# Proceedings of the 23^rd^ Paediatric Rheumatology European Society Congress: part one: Genoa, Italy. 28 September–01 October 2016

**DOI:** 10.1186/s12969-017-0141-9

**Published:** 2017-05-30

**Authors:** F. De Benedetti, J. Anton, M. Gattorno, H. Lachmann, I. Kone-Paut, S. Ozen, J. Frenkel, A. Simon, A. Zeft, E. Ben-Chetrit, H. M. Hoffman, Y. Joubert, K. Lheritier, A. Speziale, J. Guido, Roberta Caorsi, Federica Penco, Alice Grossi, Antonella Insalaco, Maria Alessio, Giovanni Conti, Federico Marchetti, Alberto Tommasini, Silvana Martino, Romina Gallizzi, Annalisa Salis, Francesca Schena, Francesco Caroli, Alberto Martini, Gianluca Damonte, Isabella Ceccherini, Marco Gattorno, Marie-Louise Frémond, Carolina Uggenti, Lien Van Eyck, Isabelle Melki, Darragh Duffy, Vincent Bondet, Yoann Rose, Bénédicte Neven, Yanick Crow, Mathieu P. Rodero, Yvonne Kusche, Johannes Roth, Katarzyna Barczyk-Kahlert, Giovanna Ferrara, Annalisa Chiocchetti, Silvio Polizzi, Josef Vuch, Diego Vozzi, Anna Mondino, Erica Valencic, Serena Pastore, Andrea Taddio, Flavio Faletra, Umberto Dianzani, Ugo Ramenghi, Alberto Tommasini, Qing Zhou, Xiaomin Yu, Erkan Demirkaya, Natalie Deuitch, Deborah Stone, Wanxia Tsai, Amanda Ombrello, Tina Romeo, Elaine F. Remmers, JaeJin Chae, Massimo Gadina, Steven Welch, Seza Ozen, Rezan Topaloglu, Mario Abinun, Daniel L. Kastner, Ivona Aksentijevich, Donatella Vairo, Rosalba Monica Ferraro, Giulia Zani, Jessica Galli, Micaela De Simone, Marco Cattalini, Elisa Fazzi, Silvia Giliani, Ebun Omoyinmi, Ariane Standing, Dorota Rowczenio, Annette Keylock, Sonia Melo Gomes, Fiona Price-Kuehne, Sira Nanthapisal, Claire Murphy, Thomas Cullup, Lucy Jenkins, Kimberly Gilmour, Despina Eleftheriou, Helen Lachmann, Philip Hawkins, Nigel Klein, Paul Brogan, Anita Dhanrajani, Mercedes Chan, Stephanie Pau, Janet Ellsworth, Jaime Guzman, Florence A. Aeschlimann, Marinka Twilt, Simon W. Eng, Shehla Sheikh, Ronald M. Laxer, Diane Hebert, Damien Noone, Christian Pagnoux, Susanne M. Benseler, Rae S. Yeung, Christoph Kessel, Katrin Lippitz, Toni Weinhage, Claas Hinze, Helmut Wittkowski, Dirk Holzinger, Niklas Grün, Dirk Föll, Pieter Van Dijkhuizen, Federica Del Chierico, Clara Malattia, Alessandra Russo, Denise Pires Marafon, Nienke M. ter Haar, Silvia Magni-Manzoni, Sebastiaan J. Vastert, Bruno Dallapiccola, Berent Prakken, Alberto Martini, Fabrizio De Benedetti, Lorenza Putignani, Berna Eren Fidanci, Kenan Barut, Serap Arıcı, Dogan Simsek, Mustafa Cakan, Ezgi D. Batu, Sezgin Şahin, Ayşenur Kısaarslan, Ebru Yilmaz, Özge Basaran, Ferhat Demir, Kubra Ozturk, Zübeyde Gunduz, Betül Sozeri, Balahan Makay, Nuray Ayaz, Onder Yavascan, Ozlem Aydog, Yelda Bilginer, Zelal Ekinci, Dilek Yıldız, Faysal Gök, Muferret Erguven, Erbil Unsal, Ozgur Kasapcopur, Seza Ozen, Erkan Demirkaya, For the FMF Arthritis Vasculitis and Orphan Disease Research in Paediatric Rheumatology (FAVOR), Hafize E. Sönmez, Ezgi D. Batu, Betül Sözeri, Yonatan Butbul, Yelda Bilginer, Seza Özen, Claudia Bracaglia, Giusi Prencipe, Manuela Pardeo, Geneviève Lapeyre, Emiliano Marasco, Antonella Insalaco, Walter Ferlin, Robert Nelson, Cristina de Min, Fabrizio De Benedetti, N. Ruperto, H. I. Brunner, P. Quartier, T. Constantin, E. Alexeeva, I. Kone-Paut, K. Marzan, N. Wulffraat, R. Schneider, S. Padeh, V. Chasnyk, C. Wouters, J. B. Kuemmerle-Deschner, T. Kallinich, B. Lauwerys, E. Haddad, E. Nasonov, M. Trachana, O. Vougiouka, K. Leon, E. Vritzali, K. Lheritier, A. Martini, D. Lovell, PRINTO/PRCSG, Francesca Schena, Stefano Volpi, Roberta Caorsi, Federica Penco, Claudia Pastorino, Francesca Kalli, Alessia Omenetti, Sabrina Chiesa, Arinna Bertoni, Paolo Picco, Gilberto Filaci, Ivona Aksentijevich, Alice Grossi, Isabella Ceccherini, Alberto Martini, Elisabetta Traggiai, Marco Gattorno, Isabelle Melki, Yoann Rose, Carolina Uggenti, Marie-Louise Fremond, Lien Van Eyck, Naoki Kitabayashi, Marco Gattorno, Stefano Volpi, Olivero Sacco, Isabelle Meyts, Marie-Anne Morren, Carine Wouters, Eric Legius, Isabelle Callebaut, Christine Bodemer, Frederic Rieux-Laucat, Mathieu Rodero, Yanick Crow, Marie-Louise Frémond, Mathieu P. Rodero, Nadia Jeremiah, Alexandre Belot, Eric Jeziorski, Darragh Duffy, Didier Bessis, Guilhem Cros, Gillian I. Rice, Bruno Charbit, Anne Hulin, Nihel Khoudour, Consuelo Modesto Caballero, Christine Bodemer, Monique Fabre, Laureline Berteloot, Muriel Le Bourgeois, Philippe Reix, Thierry Walzer, Despina Moshous, Stéphane Blanche, Alain Fischer, Brigitte Bader-Meunier, Frédéric Rieux-Laucat, Yanick Crow, Bénédicte Neven, K. Annink, N. ter Haar, S. Al-Mayouf, G. Amaryan, J. Anton, K. Barron, S. Benseler, P. Brogan, L. Cantarini, M. Cattalini, A. Cochino, F. De Benedetti, F. Dedeoglu, A. De Jesus, O. Dellacasa, E. Demirkaya, P. Dolezalova, K. Durrant, G. Fabio, R. Gallizzi, R. Goldbach-Mansky, E. Hachulla, V. Hentgen, T. Herlin, M. Hofer, H. Hoffman, A. Insalaco, A. Jansson, T. Kallinich, I. Koné-Paut, A. Kozlova, J. Kuemmerle-Deschner, H. Lachmann, R. Laxer, A. Martini, S. Nielsen, I. Nikishina, A. Ombrello, S. Ozen, E. Papadopoulou-Alataki, P. Quartier, A. Ravelli, D. Rigante, R. Russo, A. Simon, M. Trachana, Y. Uziel, M. Gattorno, J. Frenkel, Nienke ter Haar, Jerold Jeyaratnam, Helen Lachmann, Anna Simon, Paul Brogan, Matteo Doglio, Marco Cattalini, Jordi Anton, Consuelo Modesto, Pierre Quartier, Esther Hoppenreijs, Silvana Martino, Antonella Insalaco, Luca Cantarini, Loredana Lepore, Maria Alessio, Inmaculada Calvo Penades, Christina Boros, Rita Consolini, Donato Rigante, Ricardo Russo, Jana Pachlopnik Schmid, Thirusha Lane, Alberto Martini, Nicolino Ruperto, Joost Frenkel, Marco Gattorno, Chiara Passarelli, Elisa Pisaneschi, Virginia Messia, Manuela Pardeo, Antonio Novelli, Fabrizio Debenedetti, Antonella Insalaco, P. A. Brogan, M. Hofer, J. B. Kuemmerle-Deschner, B. Lauwerys, A. Speziale, X. Wei, R. Laxer, Antonella Insalaco, Denise Pires Marafon, Martina Finetti, Manuela Pardeo, Silvana Martino, Marco Cattalini, Maria Alessio, Francesca Orlando, Andrea Taddio, Serena Pastore, Elisabetta Cortis, Angela Miniaci, Nicola Ruperto, Alberto Martini, Fabrizio De Benedetti, Marco Gattorno, Charlotte Eijkelboom, Nienke ter Haar, Luca Cantarini, Martina Finetti, Paul Brogan, Pavla Dolezalova, Isabelle Koné-Paut, Antonella Insalaco, Marija Jelusic-Drazic, Liliana Bezrodnik, Mari Carmen Pinedo, Valda Stanevicha, Marielle van Gijn, Silvia Federici, Nicolino Ruperto, Joost Frenkel, Marco Gattorno, Hermann Girschick, Martina Finetti, Francesca Orlando, Antonella Insalaco, Gerd Ganser, Susan Nielsen, Troels Herlin, Isabelle Koné-Paut, Silvana Martino, Marco Cattalini, Jordi Anton, Sulaiman Mohammed Al-Mayouf, Michael Hofer, Pierre Quartier, Christina Boros, Jasmin Kuemmerle-Deschner, Susanne Schalm, Maria Alessio, Nicolino Ruperto, Alberto Martini, Annette Jansson, Marco Gattorno, on behalf of PRINTO and Eurofever registry, Martina Finetti, Marta Marchi, Chiara Marini, Matteo Doglio, Clara Malattia, Angelo Ravelli, Alberto Martini, Alberto Garaventa, Marco Gattorno, Arinna Bertoni, Sonia Carta, Enrica Balza, Patrizia Castellani, Caterina Pellecchia, Federica Penco, Francesca Schena, Silvia Borghini, Maria Libera Trotta, Claudia Pastorino, Isabella Ceccherini, Alberto Martini, Marco Gattorno, Anna Rubartelli, Sabrina Chiesa, Jaime Guzman, Andrew Henrey, Thomas Loughin, Roberta Berard, Natalie Shiff, Roman Jurencak, Susanne Benseler, Lori Tucker, on behalf of ReACCh-Out Investigators, Charalampia Papadopoulou, Ying Hong, Petra Krol, Yiannis Ioannou, Clarissa Pilkington, Hema Chaplin, Stephania Simou, Marietta Charakida, Lucy Wedderburn, Paul Brogan, Despina Eleftheriou, Lynn R. Spiegel, Sara Ahola Kohut, Jennifer Stinson, Paula Forgeron, Miriam Kaufman, Nadia Luca, Khush Amaria, Mary Bell, J Swart, F. Boris, E. Castagnola, A. Groll, G. Giancane, G. Horneff, H. I. Huppertz, D. Lovell, T. Wolfs, M. Hofer, E. Alekseeva, V. Panaviene, S. Nielsen, J. Anton, F. Uettwiller, V. Stanevicha, M. Trachana, F. De Benedetti, L. M. Ailioaie, E. Tsitami, S. Kamphuis, T. Herlin, P. Dolezalova, G. Susic, F. Sztajnbok, B. Flato, A. Pistorio, A. Martini, N. Wulffraat, N. Ruperto, Stephanie J. W. Shoop, Suzanne M. M. Verstappen, Janet E. McDonagh, Wendy Thomson, Kimme L. Hyrich, CAPS, Maarit Tarkiainen, Pirjo Tynjala, Pekka Lahdenne, Janne Martikainen, Acute-JIA Study Group, Meredyth Wilkinson, Christopher Piper, Georg Otto, Claire T. Deakin, Stefanie Dowle, Stefania Simou, Daniel Kelberman, Yiannis Ioannou, Claudia Mauri, Elizabeth Jury, David Isenberg, Lucy R. Wedderburn, Kiran Nistala, I. Foeldvari, N. Ruperto, D. J. Lovell, G. Horneff, H. I. Huppertz, P. Quartier, G. Simonini, M. Bereswill, J. Kalabic, A. Martini, H. I. Brunner, Kiem Oen, Jaime Guzman, Brian M. Feldman, Brenden Dufault, Jennifer Lee, Natalie Shiff, Karen Watanabe Duffy, Lori Tucker, Ciaran Duffy, ReACCh-Out Investigators, N. Ruperto, D. J. Lovell, N. Tzaribachev, G. Vega-Cornejo, I. Louw, A. Berman, I. Calvo, R. Cuttica, G. Horneff, F. Avila-Zapata, J. Anton, R. Cimaz, E. Solau-Gervais, R. Joos, G. Espada, X. Li, M. Nys, R. Wong, S. Banerjee, A. Martini, H. I. Brunner, For Pediatric Rheumatology International Trials Organization (PRINTO)/Pediatric Rheumatology Collaborative Study Group (PRCSG), Rebecca Nicolai, Denise Pires Marafon, Margherita Verardo, Adele D’Amico, Luisa Bracci-Laudiero, Fabrizio De Benedetti, Gian Marco Moneta, Alexandre Belot, Gillian Rice, Anne-Laure Mathieu, Sulliman O. Omarjee, Brigitte Bader-Meunier, Thierry Walzer, Tracy A. Briggs, James O’Sullivan, Simon Williams, Rolando Cimaz, Eve Smith, Michael W. Beresford, Yanick J. Crow, GENIAL Investigators, UK JSLE Study Group, Madeleine Rooney, Nick Bishop, joyce davidson, Clarissa pilkington, Michael Beresford, Jacqui Clinch, Rangaraj Satyapal, Helen Foster, Janet Gardner Medwin, Janet McDonagh, Sue Wyatt, On Behalf of the British Society for Paediatric and Adolescent Rheumatology, Valentina Litta Modignani, Francesco Baldo, Stefano Lanni, Alessandro Consolaro, Angelo Ravelli, Giovanni Filocamo, Alessia Omenetti, Joost Frenkel, Helen J. Lachmann, Seza Ozen, Nicolino Ruperto, Marco Gattorno, on behalf of Eurofever Registry, Antonella Insalaco, Gianmarco Moneta, Manuela Pardeo, Chiara Passarelli, Camilla Celani, Virginia Messia, Fabrizio De Benedetti, Bilade Cherqaoui, Linda Rossi-Semerano, Perrine Dusser, Véronique Hentgen, Isabelle Koné-Paut, Claire Grimwood, Perrine Dusser, Linda Rossi, Isabelle Kone Paut, Veronique Hentgen, Denise Lasigliè, Denise Ferrera, Giulia Amico, Marco Di Duca, Roberta Caorsi, Loredana Lepore, Antonella Insalaco, Marco Cattalini, Laura Obici, Rita Consolini, Roberto Ravazzolo, Alberto Martini, Isabella Ceccherini, Ryuta Nishikomori, Juan Arostegui, Marco Gattorno, Silvia Borghini, Federica Penco, Andrea Petretto, Chiara Lavarello, Elvira Inglese, Alessia Omenetti, Martina Finetti, Claudia Pastorino, Arinna Bertoni, Marco Gattorno, Federica Vanoni, Silvia Federici, Seza Ozen, Joost Frenkel, Helen Lachmann, Alberto Martini, Nicola Ruperto, Marco Gattorno, Michaël Hofer, on behalf of EUROFEVER PROJECT, J. B. Kuemmerle-Deschner, H. M. Hoffman, P. N. Hawkins, T. van der Poll, U. A. Walker, A. Speziale, Y. Joubert, H. H. Tilson, Jasmin Kuemmerle-Deschner, Seza Ozen, Pascal N. Tyrrell, Isabelle Koné-Paut, Raphaela Goldbach-Mansky, Helen Lachmann, Norbert Blank, Hal M. Hoffman, Elisabeth Weissbarth-Riedel, Boris Huegle, Tilmann Kallinich, Marco Gattorno, Ahmet Gul, Nienke M. ter Haar, Marlen Oswald, Fatma Dedeoglu, Susanne M. Benseler, Aki Hanaya, Takako Miyamae, Manabu Kawamoto, Yumi Tani, Takuma Hara, Yasushi Kawaguchi, Satoru Nagata, Hisashi Yamanaka, Almira Ćosićkić, Fahrija Skokić, Belkisa Čolić, Sanimir Suljendić, Anna Kozlova, Irina Mersiyanova, Mariya Panina, Lily Hachtryan, Vasiliy Burlakov, Elena Raikina, Alexey Maschan, Anna Shcherbina, Banu Acar, Meryem Albayrak, Betul Sozeri, Sezgin Sahin, Kenan Barut, Amra Adrovic, Nese Inan, Serhan Sevgi, Ozgur Kasapcopur, Caroline M. Andreasen, Anne Grethe Jurik, Mia B. Glerup, Christian Høst, Birgitte T. Mahler, Ellen-Margrethe Hauge, Troels Herlin, Cecilia Lazea, Laura Damian, Calin Lazar, Rodica Manasia, Chloe M. Stephenson, Vimal Prajapati, Paivi M. Miettunen, Dilek Yılmaz, Yavuz Tokgöz, Yasin Bulut, Harun Çakmak, Ferah Sönmez, Elif Comak, Gülşah Kaya Aksoy, Mustafa Koyun, Sema Akman, Yunus Arıkan, Ender Terzioğlu, Osman Nidai Özdeş, İbrahim Keser, Hüseyin Koçak, Ayşen Bingöl, Aygen Yılmaz, Reha Artan, F. De Benedetti, J. Anton, M. Gattorno, H. Lachmann, I. Kone-Paut, S. Ozen, J. Frenkel, A. Simon, A. Zeft, E. Ben-Chetrit, H. M. Hoffman, Y. Joubert, K. Lheritier, A. Speziale, J. Guido, X. Xu, Fatemeh F. Mehregan, Vahid Ziaee, Mohammad H. Moradinejad, Giovanna Ferrara, Serena Pastore, Antonella Insalaco, Manuela Pardeo, Alberto Tommasini, Francesco La Torre, Clotilde Alizzi, Rolando Cimaz, Martina Finetti, Marco Gattorno, Pio D’Adamo, Andrea Taddio, H. Lachmann, A. Simon, J. Anton, M. Gattorno, I. Kone-Paut, S. Ozen, J. Frenkel, E. Ben-Chetrit, H. Hoffman, A. Zeft, Y. Joubert, K. Lheritier, A. Speziale, G. Junge, J. Gregson, F. De Benedetti, Hasmik Sargsyan, Hasmik Sargsyan, Hulya Zengin, Berna E. Fidanci, Cagla Kaymakamgil, Dilek Konukbay, Dogan Simsek, Ezgi D. Batu, Dilek Yildiz, Faysal Gok, Seza Ozen, Erkan Demirkaya, Iris Stoler, Judith Freytag, Banu Orak, Christine Seib, Lars Esmann, Eva Seipelt, Faekah Gohar, Dirk Foell, Helmut Wittkowski, Tilmann Kallinich, Ismail Dursun, Sebahat Tulpar, Sibel Yel, Demet Kartal, Murat Borlu, Funda Bastug, Hakan Poyrazoglu, Zubeyde Gunduz, Kader Kose, Mehmet E. Yuksel, Abdullah Calıskan, Ahmet B. Cekgeloglu, Ruhan Dusunsel, Katerina Bouchalova, Jana Franova, Marcel Schuller, Marie Macku, Katerina Theodoropoulou, Raffaella Carlomagno, Annette von Scheven-Gête, Claudia Poloni, Michael Hofer, Laura O. Damian, Dan Cosma, Amanda Radulescu, Dan Vasilescu, Liliana Rogojan, Calin Lazar, Simona Rednic, Mihaela Lupse, Lien De Somer, Pierre Moens, Carine Wouters, Rocio Galindo Zavala, Laura Martín Pedraz, Esmeralda Núñez Cuadros, Gisela Díaz-Cordovés Rego, Antonio L. Urda Cardona, Rocio Galindo Zavala, Laura Martín Pedraz, Esmeralda Núñez Cuadros, Gisela Díaz-Cordovés Rego, Antonio L. Urda Cardona, Ilaria Dal Forno, Sara Pieropan, Ombretta Viapiana, Davide Gatti, Gloria Dallagiacoma, Paola Caramaschi, Domenico Biasi, Daniel Windschall, Ralf Trauzeddel, Hartwig Lehmann, Gerd Ganser, Rainer Berendes, Maria Haller, Manuela Krumrey-Langkammerer, Antje Nimtz-Talaska, Philipp Schoof, Ralf Felix Trauzeddel, Christine Nirschl, Estefania Quesada-Masachs, Carla Aguilar Blancafort, Sara Marsal Barril, Consuelo Modesto Caballero, Francisca Aguiar, Rita Fonseca, Duarte Alves, Ana Vieira, Alberto Vieira, Jorge A. Dias, Iva Brito, Gordana Susic, Vera Milic, Goran Radunovic, Ivan Boricic, Pauline Marteau, Catherine Adamsbaum, Linda Rossi-Semerano, Michel De Bandt, Irène Lemelle, Chantal Deslandre, Tu Anh Tran, Anne Lohse, Elisabeth Solau-Gervais, Pascal Pillet, Brigitte Bader-Meunier, Julien Wipff, Cécile Gaujoux-Viala, Sylvain Breton, Valérie Devauchelle-Pensec, Sandra Gran, Olesja Fehler, Stefanie Zenker, Michael Schäfers, Johannes Roth, Thomas Vogl, Severine Guillaume Czitrom, Dirk Foell, Dirk Holzinger, Stefano Lanni, EH Pieter Van Dijkhuizen, Silvia Magni Manzoni, Denise Pires Marafon, Francesca Magnaguagno, Laura Tanturri de Horatio, Nienke M. Ter Haar, Annemieke S. Littooij, Sebastiaan J. Vastert, Fabrizio De Benedetti, Angelo Ravelli, Alberto Martini, Clara Malattia, Vitor A. Teixeira, Raquel Campanilho-Marques, Ana F. Mourão, Filipa O. Ramos, Manuela Costa, Wafa A. Madan, Orla G. Killeen, Adriana Rodriguez Vidal, Diana Sueiro Delgado, Maria Isabel Gonzalez Fernandez, Berta Lopez Montesinos, Inmaculada Calvo Penades, Aleksey Kozhevnikov, Nina Pozdeeva, Mikhail Konev, Evgeniy Melchenko, Vladimir Kenis, Gennadiy Novik, Betul Sozeri, Aysenur Pac Kısaarslan, Zubeyde Gunduz, Hakan Poyrazoglu, Ruhan Dusunsel, Butsabong Lerkvaleekul, Suphaneewan Jaovisidha, Witaya Sungkarat, Niyata Chitrapazt, Praman Fuangfa, Thumanoon Ruangchaijatuporn, Soamarat Vilaiyuk, Dan Ø. Pradsgaard, Arne Hørlyck, Anne H. Spannow, Carsten W. Heuck, Troels Herlin, Talia Diaz, Fernando Garcia, Lorenia De La Cruz, Nadina Rubio, Joanna Świdrowska-Jaros, Elzbieta Smolewska, Mirta Lamot, Lovro Lamot, Mandica Vidovic, Edi Paleka Bosak, Ivana Rados, Miroslav Harjacek, Nikolay Tzaribachev, Polymnia Louka, Romiesa Hagoug, Chiara Trentin, Olga Kubassova, Mark Hinton, Mikael Boesen, Olena A. Oshlianska, Illya A. Chaikovsky, G. Mjasnikov, A. Kazmirchyk, Umberto Garagiola, Irene Borzani, Paolo Cressoni, Fabrizia Corona, Eszter Dzsida, Giampietro Farronato, Umberto Garagiola, Paolo Cressoni, Fabrizia Corona, Antonella Petaccia, Eszter Dzsida, Giampietro Farronato, Alenka Gagro, Agneza Marija Pasini, Goran Roic, Ozren Vrdoljak, Lucija Lujic, Matija Zutelija-Fattorini, Monika M. Esser, Deepthi R. Abraham, Craig Kinnear, Glenda Durrheim, Mike Urban, Eileen Hoal, Yanick Crow, Olena A. Oshlianska, Victoria B. Nikolayenko, Kubilay Şahin, Yasar Karaaslan, Adele Civino, Giovanni Alighieri, Sergio Davì, Roberto Rondelli, Silvana Martino, Giovanni Filocamo, Andrea Magnolato, Francesca Ricci, Romina Gallizzi, Alma Olivieri, Valeria Gerloni, Bianca Lattanzi, Francesca Soscia, Alessandro De Fanti, Silvia Magni Manzoni, Stefania Citiso, Lorenzo Quartulli, Francesco La Torre, Donato Rigante, Maria Cristina Maggio, Manuela Marsili, Maria Antonietta Pelagatti, Valentino Conter, Franca Fagioli, Loredana Lepore, Andrea Pession, Angelo Ravelli, Alessandro Consolaro, Nicolino Ruperto, Marco Garrone, Mariangela Rinaldi, Jaime De Inocencio, Erkan Demirkaya, Stella Garay, Dirk Foell, Daniel J. Lovell, Calin Lazar, Susan Nielsen, Berit Flato, Alberto Martini, Angelo Ravelli, EPOCA Study Group, Emiliano Marasco, Angela Aquilani, Simona Cascioli, Ivan Caiello, Gian Marco Moneta, Denise Pires-Marafón, Silvia Magni-Manzoni, Rita Carsetti, Fabrizio De Benedetti, Emily Robinson, Salvatore Albani, Michael W. Beresford, Wilco de Jager, Sytze de Roock, Trang Duong, Justine Ellis, Kimme Hyrich, Laetitia Jervis, Daniel Lovell, Lucy Marshall, Elizabeth D. Mellins, Kirsten Minden, Jane Munro, Peter A. Nigrovic, Jason Palman, Johannes Roth, Nicolino Ruperto, Sunil Sampath, Laura E. Schanberg, Susan D. Thompson, Wendy Thomson, Richard Vesely, Chris Wallace, Chris Williams, Qiong Wu, Nico Wulffraat, Rae S. M. Yeung, Berent Prakken, Lucy R. Wedderburn, G. Horneff, M. B. Seyger, D. Arikan, J. Kalabic, J. K. Anderson, A. Lazar, D. A. Williams, C. Wang, R. Tarzynski-Potempa, J. S. Hymans, Gabriele Simonini, Erika Scoccimarro, Irene Pontikaki, Giovanna Ferrara, Teresa Giani, Alessandro Ventura, Pier Luigi Meroni, Rolando Cimaz, Gaetana Minnone, Marzia Soligo, Ivan Caiello, Giusi Prencipe, Denise Pires Marafon, Silvia Magni-Manzoni, Luigi Manni, Fabrizio De Benedetti, Luisa Bracci Laudiero, Noortje Groot, I. Grein, N. M. Wulffraat, R. Schepp, G. Berbers, C. C. Barbosa Sandoval de Souza, V. Paes Leme Ferriani, G. Pileggi, S. de Roock, Ingrid H. R. Grein, Silvia Scala, Elisa Patrone, Casper Schoemaker, on behalf of Dutch JIA patient organization, Wendy Costello, on behalf of ENCA, Nico Wulffraat, Suzanne Parsons, Janet McDonagh, Wendy Thomson, Jean-David Cohen, Damien Bentayou, Marc-Antoine Bernard Brunel, Sonia Trope, Jens Klotsche, Miriam Listing, Martina Niewerth, Gerd Horneff, Angelika Thon, Hans-Iko Huppertz, Kirsten Mönkemöller, Ivan Foeldvari, Dirk Föll, Kirsten Minden, ICON study group, Achille Marino, Stefano Stagi, Niccolò Carli, Federico Bertini, Teresa Giani, Gabriele Simonini, Rolando Cimaz, Adriana S. Díaz-Maldonado, Sally Pino, Pilar Guarnizo, Alfonso Ragnar Torres-Jimenez, Berenice Sanchez-Jara, Eunice Solis-Vallejo, Adriana Ivonne Cespedes-Cruz, Maritza Zeferino-Cruz, Julia Veronica Ramirez-Miramontes, Ankur Kumar, Anju Gupta, Deepti Suri, Amit Rawat, Nandita Kakkar, Surjit Singh, Balahan Makay, Özge A. Gücenmez, Erbil Ünsal, Bo Magnusson, Karina Mördrup, Anna Vermé, Christina Peterson, Board of the Swedish Pediatric Rheumatology Registry, Caroline Freychet, Jean Louis Stephan, Michael Hofer, Alexandre Belot, Cathryn E. Harkness, Madeleine Rooney, Leanne Foster, Emma Henry, Pauline Taggart, Dogan Simsek, Coskun F. Ozkececi, Esra Kurt, Gokalp Basbozkurt, Faysal Gok, Erkan Demirkaya, Daiva Gorczyca, Jacek Postępski, Aleksandra Czajkowska, Bogumiła Szponar, Mariola Paściak, Anna Gruenpeter, Iwona Lachór-Motyka, Daria Augustyniak, Edyta Olesińska, Emediong S. Asuka, Tatyana Golovko, Samuel U. Aliejim, Emilio Inarejos Clemente, Estibaliz Iglesias Jimenez, Joan Calzada Hernandez, Sergi Borlan Fernandez, Clara Gimenez Roca, David Moreno Romo, Natalia Rodriguez Nieva, Juan Manuel Mosquera Angarita, Jordi Anton Lopez, Esmeralda Nuñez-Cuadros, Gisela Diaz-Cordovés, Rocío Galindo-Zavala, Antonio Urda-Cardona, Antonio Fernández-Nebro, Estefania Quesada-Masachs, Daniel Álvarez de la Sierra, Marina Garcia Prat, Mónica Martínez Gallo, Ricardo Pujol Borrell, Sara Marsal Barril, Ana M. Marín Sánchez, Consuelo Modesto Caballero, Etienne Merlin, Sylvain Breton, Sylvie Fraitag, Jean-Louis Stephan, Carine Wouters, Christine Bodemer, Brigitte Bader-Meunier, Francesco Baldo, Federico Annoni, Giancarla Di Landro, Sofia Torreggiani, Marta Torcoletti, Antonella Petaccia, Fabrizia Corona, Giovanni Filocamo, Georgina Tiller, Jo Buckle, Jane Munro, Angela Cox, Peter Gowdie, Roger C. Allen, Jonathan D. Akikusa, Hayde G. Hernández-Huirache, Edel R. Rodea-Montero, Jean-David Cohen, Alexandre Belot, William Fahy, Pierre Quartier, Christelle Sordet, Sonia Trope, Karin B. Berggren, Johanna T. Kembe, Joyce Bos, Wineke Armbrust, Nico Wulffraat, Marco van Brussel, Jeanette Cappon, Pieter Dijkstra, Jan Geertzen, Elizabeth Legger, Marion van Rossum, Pieter Sauer, Otto Lelieveld, Kubra Ozturk, Levent Buluc, Gur Akansel, Bahar Muezzinoglu, Zelal Ekinci, Ljubov Rychkova, Tatyana Knyazeva, Anna Pogodina, Tatyana Belova, Tamara Mandzyak, Ekaterina Kulesh, Alessandro Cafarotti, Manuela Marsili, Cosimo Giannini, Roberta Salvatore, Giuseppe Lapergola, Caterina Di Battista, Maria Loredana Marcovecchio, Raffaella Basilico, Piernicola Pelliccia, Francesco Chiarelli, Luciana Breda, Beverley Almeida, Sarah Tansley, Stefania Simou, Harsha Gunawardena, Neil McHugh, Lucy Wedderburn, Juvenile Dermatomyositis Research Group (JDRG), Jessie Aouizerate, Brigitte Bader-Meunier, Marie De Antonio, Christine Bodemer, Christine Barnerias, Guillaume Bassez, Isabelle Desguerre, Pierre Quartier, Romain Gherardi, Jean-Luc Charuel, François-Jérôme Authier, Cyril Gitiaux, C. H. Spencer, Rabheh Abdul Aziz, Chack-Yung Yu, Brent Adler, Sharon Bout-Tabaku, Katherine Lintner, Melissa Moore-Clingenpeel, Christina Boros, Liza McCann, Nicola Ambrose, Mario Cortina-Borja, Stefania Simou, Clarissa Pilkington, Lucy Wedderburn, Juvenile Dermatomyositis Cohort and Biomarker Study (JCDBS), Claas Hinze, Prasad T. Oommen, Fabian Speth, Johannes-Peter Haas, Working Group “Juvenile Dermatomyositis” of the German Society for Paediatric and Adolescent Rheumatology (GKJR), Claas Hinze, Prasad T. Oommen, Fabian Speth, Johannes-Peter Haas, Working Group “Juvenile Dermatomyositis” of the German Society for Paediatric and Adolescent Rheumatology (GKJR), Fabian Speth, Johannes-Peter Haas, Claas Hinze, Claudio Lavarello, Gabriella Giancane, Angela Pistorio, Lisa Rider, Rohit Aggarwal, Sheila K. Oliveira, Ruben Cuttica, Michel Fischbach, Gary Sterba, Karine Brochard, Frank Dressler, Patrizia Barone, Ruben Burgos-Vargas, Elizabeth Candell Chalom, Marine Desjonqueres, Graciela Espada, Anders Fasth, Stella Maris Garay, Rose-Marie Herbigneaux, Claire Hoyoux, Chantal Job Deslandre, Frederick W. Miller, Jiri Vencovsky, Angelo Ravelli, Alberto Martini, Nicolino Ruperto, Erdal Sag, Seza Ozen, Gulsev Kale, Haluk Topaloglu, Beril Talim, Gabriella Giancane, Claudio Lavarello, Angela Pistorio, Francesco Zulian, Bo Magnusson, Tadej Avcin, Fabrizia Corona, Valeria Gerloni, Serena Pastore, Roberto Marini, Silvana Martino, Anne Pagnier, Michel Rodiere, Christine Soler, Valda Stanevicha, Rebecca Ten Cate, Yosef Uziel, Jelena Vojinovic, Angelo Ravelli, Alberto Martini, Nicolino Ruperto, Ana V. Villarreal, Nydia Acevedo, Talia Diaz, Yuridiana Ramirez, Enrique Faugier, Rocio Maldonado, Bita Arabshahi, John H. Lee, Ian Leibowitz, Lawrence O. Okong’o, Jo Wilmshurst, Monika Esser, Christiaan Scott, Ezgi Deniz Batu, Nagehan Emiroglu, Hafize Emine Sonmez, Gokcen Dilsa Tugcu, Zehra Serap Arici, Ebru Yalcin, Deniz Dogru, Ugur Ozcelik, Yelda Bilginer, Mithat Haliloglu, Nural Kiper, Seza Ozen, Masato Yashiro, Mutsuko Yamada, Toshihiko Yabuuchi, Tomonobu Kikkawa, Nobuyuki Nosaka, Yosuke Fujii, Yukie Saito, Hirokazu Tsukahara, Sulaiman M. Al-Mayouf, Nora AlMutiari, Mohammed Muzaffer, Rawiah shehata, Adel Al-Wahadneh, Reem Abdwani, Safia Al-Abrawi, Mohammed Abu-shukair, Zeyad El-Habahbeh, Abdullah Alsonbul, Aleksandra Szabat, Monika Chęć, Violetta Opoka-Winiarska, Ankur Kumar, Anju Gupta, Amit Rawat, Biman Saikia, Ranjana W. Minz, Deepti Suri, Surjit Singh, Christine Arango, Clara Malagon, Maria D. P. Gomez, Angela C. Mosquera, Ricardo Yepez, Tatiana Gonzalez, Camilo Vargas, GRIP study group, Francesco Zulian, Marta Balzarin, Biagio Castaldi, Elena Reffo, Francesca Sperotto, Giorgia Martini, Alessandra Meneghel, Ornella Milanesi, Ivan Foeldvari, Jens Klotsche, Ozgur Kasapçopur, Amra Adrovic, Valda Stanevicha, Maria Teresa Terreri, Ekaterina Alexeeva, Maria Katsicas, Rolando Cimaz, Mikhail Kostik, Thomas Lehman, W.-Alberto Sifuentes-Giraldo, Vanessa Smith, Flavio Sztajnbok, Tadey Avcin, Maria Jose Santos, Dana Nemcova, Cristina Battagliotti, Despina Eleftheriou, Liora Harel, Mahesh Janarthanan, Tilmann Kallinich, Jordi Anton Lopez, Kirsten Minden, Susan Nielsen, Kathryn Torok, Yosef Uziel, Nicola Helmus, Ivan Foeldvari, Eileen Baildem, Michael Blakley, Christina Boros, Kim Fligelstone, Antonia Kienast, Dana Nemcova, Clare Pain, Amanda Saracino, Gabriele Simoni, Kathryn Torok, Lisa Weibel, Nicola Helmus, Ivan Foeldvari, Jens Klotsche, Ozgur Kasapçopur, Amra Adrovic, Valda Stanevicha, Maria Teresa Terreri, Ekaterina Alexeeva, Maria Katsicas, Rolando Cimaz, Mikhail Kostik, Thomas Lehman, W.-Alberto Sifuentes-Giraldo, Vanessa Smith, Flavio Sztajnbok, Tadey Avcin, Maria Jose Santos, Dana Nemcova, Cristina Battagliotti, Despina Eleftheriou, Liora Harel, Mahesh Janarthanan, Tilmann Kallinich, Jordi Anton Lopez, Kirsten Minden, Susan Nielsen, Kathryn Torok, Yosef Uziel, Nicola Helmus, Maria K. Osminina, Nathalia A. Geppe, Olga V. Niconorova, Olesya V. Karashtina, Oksana V. Abbyasova, Olga V. Shpitonkova, Amra Adrovic, Sezgin Sahin, Kenan Barut, Sinem Durmus, Hafize Uzun, Ozgur Kasapcopur, Ivan Foeldvari, Jens Klotsche, Ozgur Kasapçopur, Amra Adrovic, Valda Stanevicha, Maria Teresa Terreri, Ekaterina Alexeeva, Maria Katsicas, Rolando Cimaz, Mikhail Kostik, Thomas Lehman, W.-Alberto Sifuentes-Giraldo, Vanessa Smith, Flavio Sztajnbok, Tadey Avcin, Maria Jose Santos, Dana Nemcova, Cristina Battagliotti, Despina Eleftheriou, Liora Harel, Mahesh Janarthanan, Tilmann Kallinich, Jordi Anton Lopez, Kirsten Minden, Susan Nielsen, Kathryn Torok, Yosef Uziel, Nicola Helmus, Angela Mauro, Eleonora Fanti, Fabio Voller, Franca Rusconi, Rolando Cimaz, Fernando Garcia-Rodriguez, Ana V. Villarreal-Treviño, Angel J. Flores-Pineda, Paola B. Lara-Herrea, Diego R. Salinas-Encinas, Talia Diaz-Prieto, Maria R. Maldonado-Velazquez, Sarbelio Moreno-Espinosa, Enrique Faugier-Fuentes, Romina Gallizzi, Martina Finetti, Mirella Crapanzano, Luca Cantarini, Marco Cattalini, Giovanni Filocamo, Antonella Insalaco, Angela Mauro, Donato Rigante, Francesco Zulian, Maria Alessio, Ilaria Parissenti, Nicolino Ruperto, Marco Gattorno, Rolando Cimaz, Man S. Parihar, Surjit Singh, Pandiarajan Vignesh, Anju Gupta, ManojKumar Rohit, Kavitha Gopalan, Surjit Singh, Pandiarajan Vignesh, Anju Gupta, ManojKumar Rohit, Savita V. Attri, Ying Hong, Despina Eleftheriou, Sira Nanthapisal, Alan Salama, David Jayne, Mark Little, Paul Brogan, Yulia Kostina, Galina Lyskina, Olga Shpitonkova, Alena Torbyak, Galina Lyskina, Olga Shirinsky, Angela Mauro, Maria Francesca Gicchino, Maria Cristina Smaldone, Mario Diplomatico, Alma Nunzia Olivieri, C H. Spencer, Rabheh Abdul Aziz, Richard McClead, Sharon Bout-Tabaku, Hiren Patel, Chung-Yung Yu, Coskun F. Ozkececi, Gokalp Basbozkurt, Dogan Simsek, Esra Kurt, Faysal Gok, Erkan Demirkaya, Dita Cebecauerová, Tomáš Dallos, Edita Kabíčková, Martin Kynčl, Daniela Chroustová, Jozef Hoza, Dana Němcová, Vladimír Tesař, Pavla Doležalová, Ezgi Deniz Batu, Hafize Emine Sonmez, Tuncay Hazirolan, Fatih Ozaltin, Yelda Bilginer, Seza Ozen, Fabiola Almeida, Isabela H. Faria de Paula, Maíra M. Sampaio, Fernando N. Arita, Andressa G. Alves, Maria Carolina Santos, Eunice M. Okuda, Silvana B. Sacchetti, Fernanda Falcini, Marini Francesca, Stefano Stagi, Donato Rigante, Gemma Lepri, Marco Matucci-Cerinic, Maria Luisa Brandi, Giancarla Di Landro, Sofia Torreggiani, Antonella Petaccia, Marta Torcoletti, Fabrizia Corona, Giovanni Filocamo, Hakan Kisaoglu, Sema Misir, Selim Demir, Yuksel Aliyazicioglu, Mukaddes Kalyoncu, Isabela H. Faria de Paula, Carlos Eduardo Ramalho, Fabiola D. Almeida, Andressa G. Alves, Maria Carolina Santos, Silvana B. Sacchetti, Eunice M. Okuda, Joan Calzada-Hernández, Rosa Bou, Estíbaliz Iglesias, Judith Sánchez-Manubens, Fredy Hermógenes Prada Martínez, Clara Giménez Roca, Sergi Borlan Fernández, Juan Manuel Mosquera Angarita, Jordi Anton, Marek Bohm, Kamran Mahmood, Valentina Leone, Mark Wood, Ken-Ichi Yamaguchi, Satoshi Fujikawa, Working Group of Behçet’s Disease, Pediatric Rheumatology Association of Japan (PRAJ), Kyu Yeun Kim, Do Young Kim, Dong Soo Kim, Maka Ioseliani, Ivane Chkhaidze, Maia Lekishvili, Nana Tskhakaia, Shorena Tvalabeishvili, Aleksandre Kajrishvili, Maiko Takakura, Masaki Shimizu, Natsumi Inoue, Mao Mizuta, Akihiro Yachie, Clotilde Alizzi, Giovanni Corsello, Maria Cristina Maggio, Maryam Piram, Carla Maldini, Sandra Biscardi, Nathalie Desuremain, Catherine Orzechowski, Emilie Georget, Delphine Regnard, Isabelle Kone-Paut, Alfred Mahr, Mihaela Sparchez, Laura Damian, Zeno Sparchez, Nydia Acevedo Silva, Ana V. Villarreal Treviño, Yuridiana Ramirez Loyola, Talia Diaz Prieto, Enrique Faugier Fuentes, Maria D. R. Maldonado Velazquez, Pilar Perez, Angela C. Mosquera, Clara Malagon, Sagar Bhattad, Amit Rawat, Biman Saikia, Ranjana Minz, Jitendra Shandilya, Surjit Singh, Pediatric Allergy and Immunology Unit, PGIMER, Chandigarh, Man S. Parihar, Surjit Singh, Pandiarajan Vignesh, Anju Gupta, ManojKumar Rohit, Rocio Maldonado, Enrique Faugier, Ana Villarreal, Nydia Acevedo, Yuridiana Ramírez, Talia Diaz, Yulia Kostina, Galina Lyskina, Olga Shpitonkova, Kubra Ozturk, Zelal Ekinci, Zeynep Birsin Özçakar, Suat Fitoz, Fatos Yalcinkaya, Annacarin Horne, Francesca Minoia, Francesca Bovis, Sergio Davi, Priyankar Pal, Jordi Anton, Kimo Stein, Sandra Enciso, Ozgur Kasapcopur, Michael Jeng, Despoina Maritsi, Randy C. Cron, Angelo Ravelli, Anne Thorwarth, Sae Lim von Stuckrad, Angela Rösen-Wolff, Hella Luksch, Patrick Hundsdoerfer, Kirsten Minden, Peter Krawitz, Tilmann Kallinich, Betul Sozeri, Nuray Aktay Ayaz, Ezgi Deniz Batu, Balahan Makay, Sezgin Şahin, Doğan Simsek, Şebnem Sara Kılıc, Kubra Ozturk, Emine Sonmez, Aysenur Pac Kisaarslan, Ozge Altug Gucenmez, Mustafa Cakan, Z. Serap Arıcı, Amra Adrovic, Fatih Kelesoglu, Yelda Bilginer, Erkan Demirkaya, Zelal Ekinci Ekinci, Ruhan Dusunsel, Erbil Unsal, Ozgur Kasapcopur, Seza Ozen, Butsabong Lerkvaleekul, Soamarat Vilaiyuk, Maria Miranda-Garcia, Carolin Pretzer, Hans-Iko Huppertz, Gerd Horneff, Johannes-Peter Haas, Gerd Ganser, Jasmin Kuemmerle-Deschner, Helmut Wittkowski, Michael Frosch, Johannes Roth, Dirk Foell, Dirk Holzinger, F. Gohar, Angela McArdle, Niamh Callan, Belinda Hernandez, Miha Lavric, Christoph Kessel, Dirk Holzinger, Oliver FitzGerald, Stephen R. Pennington, Dirk Foell, Gerd Horneff, Joachim Peitz, Joern Kekow, Ariane Klein, Gerd Horneff, Anna C. Schulz, Kirsten Minden, Frank Weller-Heinemann, Anton Hospach, J-Peter Haas, BIKER collaborative group, Karen Put, Jessica Vandenhaute, Anneleen Avau, Annemarie van Nieuwenhuijze, Ellen Brisse, Tim Dierckx, Omer Rutgeerts, Josselyn E. Garcia-Perez, Jaan Toelen, Mark Waer, Georges Leclercq, An Goris, Johan Van Weyenbergh, Adrian Liston, Lien De Somer, Patrick Matthys, Carine H. Wouters, Mao Mizuta, Masaki Shimizu, Natsumi Inoue, Yasuo Nakagishi, Akihiro Yachie, Masaki Shimizu, Natsumi Inoue, Mao Mizuta, Akihiro Yachie, Michael J. Ombrello, Victoria Arthur, Elaine F. Remmers, Anne Hinks, Daniel L. Kastner, Patricia Woo, Wendy Thomson, International Childhood Arthritis Genetics (INCHARGE) Consortium, Barbara Stanimirovic, Biljana Djurdjevic-Banjac, Olivera Ljuboja, Boris Hugle, Fabian Speth, Johannes-Peter Haas, Despoina Maritsi, MArgarita Onoufriou, Olga Vougiouka, Despina Eleftheriou, Gerd Horneff, Joachim Peitz, Joern Kekow, Dirk Foell, Kenza Bouayed, Sanae El Hani, Imane Hafid, Nabiha Mikou, Maka Ioseliani, Maia Lekishvili, Nunu Shelia, Shorena Tvalabeishvili, Aleksandre Kajrishvili, Mari Laan, Jaanika Ilisson, Chris Pruunsild

**Affiliations:** 1 IRCCS Ospedale Pediatrico Bambino Gesú, Rome, Italy; 2 Hospital Sant Joan de Déu, Barcelona, Spain; 3 Pediatric Rheumatology, G. Gaslini Institute, Genoa, Italy; 4 UK National Amyloidosis Centre, University College London Medical School, London, UK; 5 Hôpital Kremlin Bicetre, University of Paris SUD, Paris, France; 6 Department of Pediatrics, Hacettepe University, Ankara, Turkey; 7 University Medical Center Utrecht, Utrecht, Netherlands; 8 Radboud University Medical Centre, Nijmegen, Netherlands; 9 Pediatrics Rheumatology, Cleveland Clinic, Cleveland, USA; 10 Rheumatology Unit, Hadassah—Hebrew University Medical Center, Jerusalem, Israel; 11 University of California, San Diego, La Jolla USA; 12 Novartis Pharma AG, Basel, Switzerland; 13 Second Division of Pediatrics, G. Gaslini Institute, Genova, Italy; 14 Division of Human Genetics, G. Gaslini Institute, Genova, Italy; 15 Division of Rheumathology, Bambino Gesù Children Hospital, Rome, Italy; 16 Department of Pediatrics, Federico II Hospital, Napoli, Italy; 17 Depatment of Pediatric Rheumthology and Nefrology, Policlinico di Messina, Messina, Italy; 18 Department of Pediatrics, S. Maria delle Croci Hospital, Ravenna, Ravenna, Italy; 19 Division of Rheumathology, Burlo Garofalo Children Hospital, Trieste, Italy; 20 Department of Pediatrics, Regina Margherita Hospital, Torino, Italy; 21 Department of Pediatrics, Policlinico di Messina, Messina, Italy; 22 Department of Cellular Biochemistry, University of Genova, Genova, Italy; 23 Scientific Director, G. Gaslini Institute, Genova, Italy; 24 Laboratory of Neurogenetics and Neuroinflammation, Imagine Institute, Paris, France; 25 Center for Human Immunology, Institut Pasteur, Paris, France; 26 Pediatric Hematology-Immunology and Rheumatology Department, Hôpital Necker, Paris, France; 27 Institute of Immunology, Münster, Germany; 28 Department of Pediatrics, University of Trieste, Trieste, Italy; 29 Amedeo Avogadro University of Eastern Piedmont, Novara, Italy; 30 Ophthalmology Department, University of Florence, A.O.U.C., Florence, Italy; 31 Department of Pediatrics, IRCCS Burlo Garofolo, Trieste, Italy; 32 Department of Pediatric Sciences, University of Torino, Torino, Italy; 33 Inflammatory Disease Section, National Human Genome Research Institute, Bethesda, USA; 34 Genetics and Pathogenesis of Allergy Section, National Institute of Allergy and Infectious Diseases, Bethesda, USA; 35 FMF Arthritis Vasculitis and Orphan disease Research Center (FAVOR), Gulhane Military Medical Academy, Ankara, Turkey; 36 Translational Immunology Section, National Institute of Arthritis and Musculoskeletal Diseases, Bethesda, USA; 37 Heart of England NHS Foundation Trust, Birmingham, UK; 38 Department of Pediatric Nephrology and Rheumatology, Hacettepe University Faculty of Medicine, Ankara, Turkey; 39 Institute of Cellular Medicine, Newcastle University, Newcastle, UK; 40 Translational Molecular Medicine Department, University of Brescia, Brescia, Italy; 41 Clinical and Expreimental Sciences, Pediatric Clinic, Brescia, Italy; 42 Unit of Child Neurology and Psychiatry, Spedali Civili, Brescia, Italy; 43 Clinical and Experimental Sciences, University of Brescia, Brescia, Italy; 44 IIIP, UCL, Institute of Child Health, London, UK; 45 NAC, Royal Free Hospital, London, UK; 46 NE Thames Genetics Laboratory, London, UK; 47 Immunology, GOSH, London, UK; 48 Pediatric Rheumatology, BC Children’s Hospital, University of British Columbia, Vancouver, Canada; 49 Pediatric Rheumatology, Stollery Children’s Hospital, University of Alberta, Edmonton, Canada; 50 Pediatrics, Stollery Children’s Hospital, Edmonton, Canada; 51 Rheumatology, The Hospital for Sick Children, Toronto, Canada; 52 Rheumatology, Alberta Children’s Hospital, Calgary, Canada; 53 Nephrology, The Hospital for Sick Children, Toronto, Canada; 54 Rheumatology, Mount Sinai Hospital, Toronto, Canada; 55 Pediatric Rheumatology & Immunology, University Childrens’ Hospital, Münster, Germany; 56 Paediatric Rheumatology, Istituto Giannina Gaslini, Genoa, Italy; 57 Paediatric Rheumatology, University Medical Centre Utrecht, Wilhelmina Children’s Hospital, Utrecht, Netherlands; 58 Human Microbiome Unit, Ospedale Pediatrico Bambino Gesù, Rome, Italy; 59 Università degli Studi di Genova, Genoa, Italy; 60 Paediatric Rheumatology, Ospedale Pediatrico Bambino Gesù, Rome, Italy; 61 Scientific Direction, Ospedale Pediatrico Bambino Gesù, Rome, Italy; 62 Gulhane Military Medical Academy, Ankara, Turkey; 63 Istanbul University, Istanbul, Turkey; 64 Hacettepe University, Ankara, Turkey; 65 Kanuni Sultan Suleyman Research Hospital, Istanbul, Turkey; 66 Erciyes University, Kayseri, Turkey; 67 Tepecik Training Hospital, Izmir, Turkey; 68 Dıskapı Training Hospital, Ankara, Turkey; 69 Karadeniz Technical University, Trabzon, Turkey; 70 Kocaeli University, Kocaeli, Turkey; 71 9 Eylul University, Izmir, Turkey; 72 Sami Ulus Children Hospital, Ankara, Turkey; 73 Goztepe Training Hospital, Istanbul, Turkey; 74 Division of Rheumatology, Hacettepe University Faculty Of Medicine, Ankara, Turkey; 75 Division of Rheumatology, Erciyes University Faculty of Medicine, Kayseri, Turkey; 76 Division of Rheumatology, Rambam Medical Center, Haifa, Israel; 77 Division of Rheumatology, Ospedale Pediatrico Bambino Gesù IRCCS, Roma, Italy; 78 Novimmune SA, Geneva, Switzerland; 79 PRINTO-Istituto Gaslini, Genoa, Italy; 80 PRCSG, Cincinnati, OH USA; 81 Necker-Enfants Malades Hospital, Paris, France; 82 Cliniques Universitaires Saint-Luc and Université Catholique de Louvain, Brussels, Belgium; 83 Novartis Pharmaceuticals Corporation, East Hanover, NJ USA; 84 Novartis Pharma AG, Basel, Switzerland; 85 Second Pediatric Division, Giannina Gaslini Institute, Genova, Italy; 86 CEBR, Università, Genova, Italy; 87 National Human Genome Research Institute, National Institute of Health, Bethesda, MD USA; 88 Medical Genetics, G. Gaslini Institute, Genova, Italy; 89 Novartis Institute for Research in Biomedicine, Basel, Switzerland; 90 Laboratory of Neurogenetics and Neuroinflammation, Imagine Institute, Paris, France; 91 General Pediatrics - Internal Medicine - Infectious diseases, Robert Debré Hospital - APHP, Paris, France; 92 Pediatric Immuno-Haematology and Rheumatology Unit, Necker Hospital - APHP, Paris, France; 93 UO Pediatria 2 Reumatologia, Genova, Italy; 94 UO Pneumologia, G. Gaslini, Genova, Italy; 95 Immunology, UZLeuven, Leuven, Belgium; 96 Dermatology, UZLeuven, Leuven, Belgium; 97 Rheumatology, UZLeuven, Leuven, Belgium; 98 Genetics, UZLeuven, Leuven, Belgium; 99 MPMC, Sorbonne University — UMR CNRS 7590, UPMC Sorbonne Paris Cité, Muséum National d’Histoire Naturelle, Paris, France, IRD UMR 206, Paris, France; 100 Dermatology, Necker Hospital - APHP, Paris, France; 101 INSERM UMR 1163, Laboratory of Immunogenetics of Pediatric Autoimmunity, Imagine Institute, Paris, France; 102 Laboratory of Neurogenetics and Neuroinflammation, Paris, France; 103 Laboratory of Immunogenetics of Pediatric Autoimmunity, Imagine Institute, Paris, France; 104 Pediatric Rheumatology, Nephrology and Dermatology Department, Hospices Civils de Lyon, Lyon, France; 105 Pediatrics Department, Centre Hospitalier Universitaire de Montpellier, Montpellier, France; 106 Laboratory of Dendritic Cell Immunobiology, Institut Pasteur, Paris, France; 107 Dermatology Department, Centre Hospitalier Universitaire de Montpellier, Montpellier, France; 108 Pediatric Hematology-Immunology and Rheumatology Department, Hôpital Necker-Enfants Malades, Paris, France; 109 Manchester Centre for Genomic Medicine, University of Manchester, Manchester, UK; 110 Pharmacology and Toxicology Laboratory, Hôpital Universitaire Henri Mondor, Créteil, France; 111 Pediatric Rheumatology Department, Hospital Universitari Vall d’Hebron, Barcelona, Spain; 112 Dermatology Department, Hôpital Necker-Enfants Malades, Paris, France; 113 Pathology Department, Hôpital Necker-Enfants Malades, Paris, France; 114 Pediatric Radiology Department, Hôpital Necker-Enfants Malades, Paris, France; 115 Pediatric Pneumology Department, Hôpital Necker-Enfants Malades, Paris, France; 116 Pneumology Department, Hospices Civils de Lyon, Lyon, France; 117 Centre International de Recherche en Infectiologie, Université Lyon 1, Lyon, France; 118 UMC Utrecht, Utrecht, Netherlands; 119 The ADDI consortium, Genua, Italy; 120 Autoinflammatory Alliance, San Fransisco, CA USA; 121 Eurofever Project, University Medical Center Utrecht, Utrecht, Netherlands; 122 Division of Medical Genetics, Roma, Italy; 123 Division of Rheumatology, Ospedale Pediatrico Bambino Gesù IRCCS, Roma, Italy; 124 UCL Institute of Child Health and Great Ormond Street Hospital NHS Foundation Trust, Department of Paediatric Rheumatology, London, UK; 125 Unité Romande de Rhumatologie Pédiatrique, Hospitalier Universitaire Vaudois, Lausanne, Switzerland; 126 University Hospital Tuebingen, Tuebingen, Germany; 127 Cliniques Universitaires Saint-Luc and Université Catholique de Louvain, Brussels, Belgium; 128 Novartis Pharma AG, Basel, Switzerland; 129 Novartis Pharma, Beijing, China; 130 University of Toronto, The Hospital for Sick Children, Ontario, Canada; 131 Division of Rheumatology, Ospedale Pediatrico Bambino Gesù IRCCS, Roma, Italy; 132 Pediatria II Reumatologia PRINTO, Istituto Giannina Gaslini, Genova, Italy; 133 Division of Rheumatology, Ospedale Pediatrico Bambino Gesù, Roma, Italy; 134 Paediatrics Departments, Clinica Pediatrica Universita’ di Torino, Torino, Italy; 135 Unità di immunologia e Reumatologia Pediatrica, Clinica Pediatrica Università Brescia, Spedali Civili, Brescia, Italy; 136 Dipartimento di Pediatria, Università di Napoli Federico II, Napoli, Italy; 137 Scienze della Riproduzione e dello Sviluppo, Università di Trieste IRCCS Burlo Garofalo, Trieste, Italy; 138 Struttura Complessa Pediatria, Ospedale Santa Maria della Stella, Orvieto, Italy; 139 Ambulatorio di Reumatologia, Aziensa Ospedaliera Universitaria Sant’Orsola Malpighi, Bologna, Italy; 140 UMCU, Utrecht, Netherlands; 141 University of Siena, Siena, Italy; 142 IGG, Genova, Italy; 143 GOSH, London, UK; 144 General University Hospital, Prague, Czech Republic; 145 Bicêtre Hospital, Le Kremlin-Bicêtre, France; 146 OPBG, Rome, Italy; 147 University Hospital Centre, Zagreb, Croatia; 148 Hospital de Ninos Ricardo Gutierrez, Buenos Aires, Argentina; 149 Hospital de Cruces, Barakaldo, Spain; 150 Stradins University, Riga, Latvia; 151 Perinatal Centre of the Vivantes Klinikum, Clinic for Paediatric and Adolescent Medicine, Berlin, Germany; 152 Pediatria II, Reumatologia; PRINTO, Istituto Giannina Gaslini, Genoa, Italy; 153 Università di Napoli Federico II, Naples, Italy; 154 Ospedale Pediatrico Bambino Gesù, Rome, Italy; 155 St. Josef-Stift Hospital, Sendenhorst, Germany; 156 Rigshospitalet, Copenhagen, Denmark; 157 Skejby Sygehus, Aarhus University Hospital, Aarhus, Denmark; 158 Le Kremlin-Bicêtre University Hospital, Paris-Sud University, Paris, France; 159 Dipartimento di Scienze Pediatriche e dell’Adolescenza, University of Torino, Torino, Italy; 160 Unita’ di Immunologia e Reumatologia Pediatrica, Clinica Pediatrica dell’Universita’ di Brescia, Spedali Civili, Brescia, Italy; 161 Hospital Sant Joan de Déu, Barcelona, Spain; 162 King Faisal Specialist Hospital and Research Center, Riyadh, Saudi Arabia; 163 Centre Multisite Romand de Rhumatologie Pediatrique / Centre Hospitalier Universitaire Vaudois (CHUV), Lausanne, Switzerland; 164 Paris-Descartes University, IMAGINE Institute, Necker Children’s Hospital, Paris, France; 165 Women’s and Children’s Hospital, Adelaide, Australia; 166 University Children’s Hospital Tuebingen, Tuebingen, Germany; 167 Klinikum der Univeristat, Munchen, Germany; 168 Dipartimento di Pediatria, Università di Napoli Federico II, Naples, Italy; 169 Pediatria II, Reumatologia; PRINTO, Istituto Giannina Gaslini, Genoa, Italy; 170 University Medical Centre, Munich, Germany; 171 Pediatria II, Reumatologia, Istituto Giannina Gaslini, Genoa, Italy; 172 Università di Genova, Genoa, Italy; 173 Pediatria IV, Oncologia, Istituto Giannina Gaslini, Genoa, Italy; 174 Laboratorio di Immunologia delle Malattie Reumatiche, Giannina Gaslini Institute, Genova, Italy; 175 Unità di Biologia Cellulare, IRCCS San Martino-Ist, Genova, Italy; 176 Genetica Medica, Giannina Gaslini Institute, Genova, Italy; 177 Università degli Studi di Genova, Genova, Italy; 178 Pediatric Division, Giannina Gaslini Institute, Genova, Italy; 179 Pediatrics, University of British Columbia, Vancouver, Canada; 180 Statistics, Simon Fraser University, Burnaby, Canada; 181 Pediatrics, Western University, London, Canada; 182 Pediatrics, University of Florida, Gainesville, FL USA; 183 Pediatrics, University of Ottawa, Ottawa, Canada; 184 Pediatrics, University of Calgary, Calgary, Canada; 185 Department of Paediatric Rheumatology, Great Ormond Street Hospital and UCL Institute of Child Health, London, UK; 186 Arthritis Research UK, Centre for Adolescent Rheumatology, London, UK; 187 Institute of Cardiovascular Sciences, University College London, London, UK; 188 Rheumatology, The Hospital for Sick Children, Toronto, Canada; 189 Pediatrics, University of Toronto, Toronto, Canada; 190 Psychology, University of Toronto, Toronto, Canada; 191 Child Health Evaluative Sciences, The Hospital for Sick Children, Toronto, Canada; 192 Child and Evaluative Health Sciences, The Hospital for Sick Children, Toronto, Canada; 193 Lawrence S. Bloomberg Faculty of Nursing, University of Toronto, Toronto, Canada; 194 School of Nursing, University of Ottawa, Ottawa, Canada; 195 Adolescent Medicine, The Hospital for Sick Children, Toronto, Canada; 196 Rheumatology, Alberta Children’s Hospital, Calgary, Canada; 197 Rheumatology, Sunnybrook Research Institute, Toronto, Canada; 198 PRINTO, Genova, Italy; 199 Arthritis Research UK Centre for Epidemiology, The University of Manchester, Manchester, UK; 200 NIHR Manchester Musculoskeletal Biomedical Research Unit, Central Manchester University Hospitals NHS Foundation Trust and University of Manchester Partnership, Manchester, UK; 201 Centre for MSK Research, Faculty of Medical and Human Sciences, Manchester, UK; 202 Arthritis Research UK Centre for Genetics and Genomics, The University of Manchester, Manchester, UK; 203 Institute of Clinical Medicine, University of Helsinki, Helsinki, Finland; 204 Children’s Hospital, Helsinki, Finland; 205 Poison Centre, Helsinki, Finland; 206 Helsinki University Central Hospital, Helsinki, Finland; 207 University of Eastern Finland, Kuopio, Finland; 208 Centre for Adolescent Rheumatology, UCL, London, UK; 209 Centre of Rheumatology, UCL, London, UK; 210 UCL Institute of Child Health, UCL, London, UK; 211 PRINTO-IRCCS, Genova, Italy; 212 PRCSG, Cincinnati, OH USA; 213 Hôpital Necker-Enfants Malades, Paris, France; 214 AbbVie Deutschland GmbH & Co. KG, Ludwigshafen, Germany; 215 Pediatrics, University of Manitoba, Winnipeg, Canada; 216 Pediatrics, University of British Columbia, Vancouver, Canada; 217 Pediatrics, University of Toronto, Toronto, Canada; 218 Statistics, University of Manitoba, Winnipeg, Canada; 219 Pediatrics, University of Ottawa, Ottawa, Canada; 220 Pediatrics, University of Florida, Gainesville, USA; 221 Istituto G. Gaslini Pediatria II Reumatologia, Genova, Italy; 222 Cincinnati Children’s Hosp. Medical Center, Cincinnati, OH USA; 223 Pediatric Rheumatology, Bad Bramstedt, Germany; 224 CREA (Clinica de Reumatología y Enfermedades Autoinmunes) Hosp. México Americano, Guadalajara, Mexico; 225 Panorama Medical Centre, Cape Town, South Africa; 226 Univ. Nacional de Tucuman and Centro Médico Privado de Reumatología, Tucuman, Argentina; 227 Univ. La Fe, Valencia, Spain; 228 Hosp. General de Niños Pedro de Elizalde, Buenos Aires, Argentina; 229 Asklepios Clinic Sankt Augustin, Sankt Augustin, Germany; 230 Star Medica Hosp., Merida, Mexico; 231 Hosp. Sant Joan de Déu, Barcelona, Spain; 232 Ospedale Pediatrico Anna Meyer, Firenze, Italy; 233 Hôpital de la Milétrie, Poitiers, France; 234 University Hosp. Gent, Gent, Belgium; 235 Hosp. De Ninos Dr. Ricardo Gutierrez, Buenos Aires, Argentina; 236 Bristol-Myers Squibb, Princeton, USA; 237 Bristol-Myers Squibb, Braine L’Alleud, Belgium; 238 Istituto G. Gaslini Pediatria II Reumatologia and Univ. of Genova, Genova, Italy; 239 Division of Rheumatology, Ospedale Pediatrico Bambino Gesù IRCCS, Roma, Italy; 240 Unit of Neuromuscular and Neurodegenerative Disease, Department of Neuroscience, Ospedale Pediatrico Bambino Gesù IRCCS, Roma, Italy; 241 Institute of Translational Pharmacology, CNR, Roma, Italy; 242 INSERM U1111& Pediatric Rheumatology Unit, Lyon, France; 243 Manchester Academic Health Science Centre, Universtity of Manchester, Manchester, UK; 244 INSERM U1111, Lyon, France; 245 Pediatric Immunology and Rheumatology, IMAGINE Institute, Necker Hospital, Paris, France; 246 Pediatric Rheumatology, Meyer Children Hospital, Firenze University, Firenze, Italy; 247 Paediatric Rheumatology, Alder Hey Children’s NHS Foundation Trust, Liverpool, UK; 248 Neuroinflammation, Institut Imagine & Manchester Academic Health Science Centre, Paris, France; 249 Experimental Medicine, Queens University Belfast, Belfast, UK; 250 Academic Unit of Child Health, Sheffield University, Sheffield, UK; 251 Medicine, Royal Hospital for Sick Children (Yorkhill) Glasgow, Glasgow, UK; 252 Great Ormond Street Hospital for Children, NHS Trust, London, UK; 253 Institute Transnational medicine, University of Liverpool, Liverpool, UK; 254 Royal National Hospital for Rheumatic Diseases, Bath, UK; 255 Queen’s Medical Centre - Nottingham Childrens Hospital, Nottingham, UK; 256 Institute of Cellular Medicine, Newcastle University, Newcastle, UK; 257 Child Health, University of Glasgow, Glasgow, UK; 258 Institute of Inflammation and Repair, University of Manchester, Manchester, UK; 259 Rheumatology, Leeds Teaching Hospitals, Leeds, UK; 260 Dipartimento della Donna, del Bambino o del Neonato, Fondazione IRCCS cà Granda Ospedale Maggiore Policlinico, Milano, Italy; 261 IRCCS G Gaslini and Università di Genova, Genova, Italy; 262 DINOGMI, University of Genoa, Genoa, Italy; 263 University Medical Center Utrecht, Utrecht, Netherlands; 264 Royal Free Campus, London, UK; 265 Hacettepe University Children’s Hospital, Ankara, Turkey; 266 Giannina Gaslini Institute, Genoa, Italy; 267 Division of Rheumatology, Ospedale Pediatrico Bambino Gesù IRCCS, Roma, Italy; 268 Division of Medical Genetics, Ospedale Pediatrico Bambino Gesù IRCCS, Roma, Italy; 269 Pediatrics, CHU Estaing, Clermont-Ferrand, France; 270 Pediatric Rheumatology, CEREMAI, CHU, Bicêtre, France; 271 Pediatrics, CEREMAI, CH, Versailles, France; 272 French Reference Centre for Autoinflammatory Diseases, Pediatric Unit, Versailles Hospital, Le Chesnay, France; 273 French Reference Centre for Autoinflammatory Diseases, Pediatric Unit, CHU Kremlin Bicetre, Kremlin Bicetre, France; 274 IRCCS G. Gaslini, Genova, Italy; 275 IRCCS Burlo Garofalo, Trieste, Italy; 276 IRCCS Bambino Gesu’, Roma, Italy; 277 Spedali Civili, Brescia, Italy; 278 IRCCS San Matteo, Pavia, Italy; 279 Università di Pisa, Pisa, Italy; 280 Universita’ di Kyoto, Kyoto, Japan; 281 Ospedale di Barcelona, Barcelona, Spain; 282 Laboratorio di Immunologia delle Malattie Reumatiche, Genova, Italy; 283 Core Facilities-Laboratorio Proteomica, Genova, Italy; 284 Pediatria II, Istituto G. Gaslini, Genova, Italy; 285 Pediatric Rheumatology Unit, CHUV, University of Lausanne, Lausanne, Switzerland; 286 Pediatrics, ORBV - Bellinzona, Bellinzona, Switzerland; 287 Pediatria II - Reumatologia, Istituto G. Gaslini, Genoa, Italy; 288 Department of Pediatric Nephrology and Rheumatology, Hacettepe University, Ankara, Turkey; 289 Department of Paediatrics, UMC Utrecht, Utrecht, Netherlands; 290 National Amyloidosis Centre, University College Medical School, London, UK; 291 University Hospital Tuebingen, Tuebingen, Germany; 292 University of California, San Diego, CA USA; 293 University College London Medical School, London, UK; 294 Academic Medical Center, University of Amsterdam, Amsterdam, Netherlands; 295 University Hospital, Basel, Switzerland; 296 Novartis Pharma AG, Basel, Switzerland; 297 University of North Carolina, Chapel Hill, NC USA; 298 Department of Pediatrics, Division of Pediatric Rheumatology, University Hospital Tuebingen, Tuebingen, Germany; 299 Pediatric Nephrology and Rheumatology, Hacettepe University, Ankara, Turkey; 300 Department of Medical Imaging, University of Toronto, Toronto, ON Canada; 301 Pediatric Rheumatology, CHU Bicêtre, Le Kremlin Bicêtre, France; 302 Translational Autoinflammatory Disease Section, NIAMS/NIH, Bethesda, MD USA; 303 UK National Amyloidosis Centre, University College London Medical School, London, UK; 304 Haematologie, Onkologie und Rheumatologie, Universitaetsklinikum Heidelberg, Heidelberg, Germany; 305 Division of Allergy, Immunology and Rheumatology, Department of Pediatrics, UC San Diego/Rady Children’s Hospital, San Diego, CA USA; 306 Kinderrheumatologische Ambulanz, Universitaetsklinikum Eppendorf, Hamburg, Germany; 307 German Center for Pediatric and Adolescent Rheumatology, Garmisch-Partenkirchen, Germany; 308 Rheumatology, Charité, University Medicine Berlin, Berlin, Germany; 309 UO Pediatria 2, G. Gaslini Institute, Genoa, Italy; 310 Department of Internal Medicine, Rheumatology Division, Istanbul University, Istanbul, Turkey; 311 Laboratory for Translational Immunology, University Medical Center Utrecht, Utrecht, Netherlands; 312 Division of Immunology, Boston Children’s Hospital, Boston, MA USA; 313 Rheumatology, Alberta Children’s Hospital, University of Calgary, Calgary, AB Canada; 314 Department of Pediatrics, Tokyo, Japan; 315 Institute of Rheumatology, Tokyo Women’s Medical University, Tokyo, Japan; 316 Reumatology, Allergology and Immunology, University Clinical Center, Bosnia and Herzegovina, Tuzla, Bosnia and Herzegovina; 317 Neonatology, University Clinical Center, Bosnia and Herzegovina, Tuzla, Bosnia and Herzegovina; 318 Childens Surgery, Childrens Hospital, University Clinical Center, Bosnia and Herzegovina, Tuzla, Bosnia and Herzegovina; 319 Immunology, Federal Research and Clinical Center of Pediatric Hematology, Oncology and Immunology, Moscow, Russian Federation; 320 Department of Pediatric Rheumatology, Kırıkkale University, School of Medicine, Kırıkkale, Turkey; 321 Department of Pediatric Hematology, Kırıkkale University, School of Medicine, Kırıkkale, Turkey; 322 Pediatric Rheumatology, Erciyes University Faculty of Medicine, Kayseri, Turkey; 323 Pediatric Rheumatology, Cerrahpasa Medical Faculty, Istanbul University, Istanbul, Turkey; 324 Novartis Pharmaceutical Corporation, Istanbul, Turkey; 325 Department Rheumatology, Aarhus University Hospital, Aarhus, Denmark; 326 Department Radiology, Aarhus University Hospital, Aarhus, Denmark; 327 Department Paediatrics, Aarhus University Hospital, Aarhus, Denmark; 328 Pediatrics, University of Medicine and Pharmacy Cluj-Napoca, Cluj-Napoca, Romania; 329 Rheumatology, County Emergency Hospital, Cluj-Napoca, Romania; 330 University of Calgary, Calgary, Canada; 331 Paediatrics, University of Calgary, Calgary, Canada; 332 Pediatric Nephrology, Adnan Menderes University Faculty of Medicine, Aydın, Turkey; 333 Pediatric Gastroenterology, Adnan Menderes University Faculty of Medicine, Aydın, Turkey; 334 Pediatrcis, Adnan Menderes University Faculty of Medicine, Aydın, Turkey; 335 Ophtalmology, Adnan Menderes University Faculty of Medicine, Aydın, Turkey; 336 Pediatric Nephrology - Rheumatology, Akdeniz University, Antalya, Turkey; 337 Medical Genetics, Akdeniz University, Antalya, Turkey; 338 Rheumatology, Akdeniz University, Antalya, Turkey; 339 Nephrology, Akdeniz University, Antalya, Turkey; 340 Immunology, Akdeniz University, Antalya, Turkey; 341 Pediatric Gastroenterology, Hepatology & Nutrition, Akdeniz University, Antalya, Turkey; 342 IRCCS Ospedale Pediatrico Bambino Gesú, Rome, Italy; 343 Hospital Sant Joan de Déu, Barcelona, Spain; 344 Pediatric Rheumatology, G. Gaslini Institute, Genoa, Italy; 345 UK National Amyloidosis Centre, University College London Medical School, London, UK; 346 Hôpital Kremlin Bicetre, University of Paris SUD, Paris, France; 347 Department of Pediatrics, Hacettepe University, Ankara, Turkey; 348 University Medical Center Utrecht, Utrecht, Netherlands; 349 Radboud University Medical Centre, Nijmegen, Netherlands; 350 Pediatrics Rheumatology, Cleveland Clinic, Cleveland, OH USA; 351 Rheumatology Unit, Hadassah—Hebrew University Medical Center, Jerusalem, Israel; 352 University of California, San Diego, La Jolla, CA USA; 353 Novartis Pharma AG, Basel, Switzerland; 354 Novartis Pharmaceuticals Corporation, East Hanover, NJ USA; 355 Pediatric Rheumatology, Shahid Beheshti University of Medical Sciences, Tehran, Islamic Republic of Iran; 356 Pediatric Rheumatology, Tehran University of Medical Science, Tehran, Islamic Republic of Iran; 357 University of Trieste, Trieste, Italy; 358 Institute of Child and Maternal Health – IRCCS “Burlo Garofolo”, Trieste, Italy; 359 IRCCS Ospedale Pediatrico Bambino Gesù, Rome, Italy; 360 Antonio Perrino Hospital, Brindisi, Italy; 361 Ospedale Pediatrico G. Di Cristina di Palermo, Palermo, Italy; 362 Anna Meyer Children Hospital, Florence, Italy; 363 UOC Pediatria 2, Istituto Gaslini, Genova, Italy; 364 UK National Amyloidosis Centre, University College London Medical School, London, UK; 365 Radboud University Medical Centre, Nijmegen, Netherlands; 366 Hospital Sant Joan de Déu, Barcelona, Spain; 367 Pediatric Rheumatology, G. Gaslini Institute, Genoa, Italy; 368 Hôpital Kremlin Bicetre, University of Paris SUD, Paris, France; 369 Hacettepe University Children’s Hospital, Ankara, Turkey; 370 University Medical Center Utrecht, Utrecht, Netherlands; 371 Rheumatology Unit, Hadassah—Hebrew University Medical Center, Jerusalem, Israel; 372 University of California, La Jolla, USA; 373 Pediatrics Rheumatology, Cleveland Clinic, Cleveland, OH USA; 374 Novartis Pharma AG, Basel, Switzerland; 375 IRCCS Ospedale Pediatrico Bambino Gesú, Rome, Italy; 376 Yerevan State Medical University, Yerevan, Armenia; 377 Yerevan State Medical University, Yerevan, Armenia; 378 Child Health Nursing, Gülhane Military Medical Academy, Ankara, Turkey; 379 Pediatric Rheumatology, Gülhane Military Medical Academy, Ankara, Turkey; 380 Pediatric Rheumatology, Hacettepe University, Ankara, Turkey; 381 Pediatric Nephrology, Gülhane Military Medical Academy, Ankara, Turkey; 382 Pediatric Rheumatology, FAVOR, Ankara, Turkey; 383 Department of Pediatric Rheumatology, Charité Berlin, Berlin, Germany; 384 Charité Berlin, Berlin, Germany; 385 Immanuel Berlin Buch, Berlin, Germany; 386 University Hospital Muenster, Albert-Schweitzer-Campus 1, Gebäude W30, Münster, Germany; 387 Pediatric Nephrology, Erciyes University, Faculty of Medicine, Kayseri, Turkey; 388 Dermatology and Venereology, Erciyes University, Faculty of Medicine, Kayseri, Turkey; 389 Biochemistry, Erciyes University, Faculty of Medicine, Kayseri, Turkey; 390 Department of Biomedical Engineering, Faculty of Engineering, Kayseri, Turkey; 391 Department of Pediatrics, Palacky University and Faculty Hospital, Olomouc, Czech Republic; 392 Institute of Molecular and Translational Medicine, Palacky University and Faculty Hospital, Olomouc, Czech Republic; 393 Pediatric Department, University Hospital, Brno, Czech Republic; 394 Pediatric Rheumatology, CHUV, Lausanne, Switzerland; 395 Pediatric Neurology, CHUV, Lausanne, Switzerland; 396 Rheumatology, Emergency Clinical County Hospital Cluj, Cluj-Napoca, Romania; 397 Pediatric Surgery, University of Medicine and Pharmacy Cluj, Cluj-Napoca, Romania; 398 Infectious Diseases, University of Medicine and Pharmacy Cluj, Cluj-Napoca, Romania; 399 Radiology, University of Medicine and Pharmacy Cluj, Cluj-Napoca, Romania; 400 Pathology, Emergency Clinical County Hospital Cluj, Cluj-Napoca, Romania; 401 1st Pediatric, University of Medicine and Pharmacy Cluj, Cluj-Napoca, Romania; 402 Rheumatology, University of Medicine and Pharmacy Cluj, Cluj-Napoca, Romania; 403 Department of Pediatric Rheumatology, UZ Leuven, Leuven, Belgium; 404 Department of Orthopedic Surgery, UZ Leuven, Leuven, Belgium; 405 Paediatric Rheumatology, Universitary Regional Malaga Hospital, Malaga, Spain; 406 Paediatrics, Universitary Regional Malaga Hospital, Malaga, Spain; 407 Rheumatology, Universitary Regional Malaga Hospital, Malaga, Spain; 408 Paediatric Rheumatology, University Regional Hospital of Malaga, Malaga, Spain; 409 Paediatrics, Malaga University Regional Hospital, Malaga, Spain; 410 Paediatric Rheumatology, Malaga University Regional Hospital, Malaga, Spain; 411 Paediatrics, University Regional Hospital of Malaga, Malaga, Spain; 412 Rheumatology, Policlinico G.B.Rossi, Verona, Italy; 413 Asklepios Hospital Weissenfels, Weissenfels, Germany; 414 Helios Kliniken Berlin Buch, Berlin, Germany; 415 University of Giessen and Marburg, Giessen, Germany; 416 St. Joseph Stift, Sendenhorst, Germany; 417 Kinderkrankenhaus St. Marien, Landshut, Germany; 418 Kinderärztliche Praxis Gundelfingen, Gundelfingen, Germany; 419 German Center for Pediatric Rheumatology, Garmisch-Partenkirchen, Germany; 420 Kinderärztliche Praxis Frankfurt/Oder, Frankfurt/Oder, Germany; 421 Kinderärztliche Praxis, München, Germany; 422 Department of Anesthesiology and Intensive Care Medicine, Campus Charité Mitte and Campus Virchow-Klinikum, Berlin, Germany; 423 Paediatric Rheumartology Unit, Vall d’Hebron University Hospital, Barcelona, Spain; 424 Clinical Research Support Unit, Vall d’Hebron Research Institute, Barcelona, Spain; 425 Rheumatology, Vall d’Hebron University Hospital, Barcelona, Spain; 426 Paediatric Rheumatology Unit, Vall d’Hebron University Hospital, Barcelona, Spain; 427 Rheumatology, Centro Hospitalar São João, Oporto, Portugal; 428 Faculty of Medicine of Porto University, Porto, Portugal; 429 Radiology, Centro Hospitalar São João, Porto, Portugal; 430 Pediatric Gastroenterology, Centro Hospitalar São João, Porto, Portugal; 431 Pediatric Rheumatology Unit, Centro Hospitalar São João, Porto, Portugal; 432 Institute of Rheumatology, University of Belgrade, Belgrade, Serbia; 433 Medical faculty, University of Belgrade, Belgrade, Serbia; 434 Institute of pathology, Belgrade, Serbia; 435 Rheumatology, CHU Cavale Blanche, Brest, France; 436 Paediatric Radiology, AP-HP, Bicêtre, France; 437 Paediatric Rheumatology, AP-HP, Hôpital Bicêtre, Paris, France; 438 Rheumatology, CHU Martinique, Fort de France, France; 439 Rheumatology, University Hospital, Nancy, France; 440 Paediatrics, Hôpital Robert Debré, Paris, France; 441 Paediatrics, University Hospital, Nîmes, France; 442 Rheumatology, Hospital Centre of Belfort Montbeliard, Belfort, France; 443 Rheumatology, University Hospital of Poitiers, Poitiers, France; 444 Paediatrics, Hôpital Pellegrin, Bordeaux, Bordeaux, France; 445 Paediatric Rheumatology, Hôpital Necker, Paris, France; 446 Rheumatology, Hôpital Cochin, Paris, France; 447 Rheumatology, University Hospital, Nîmes, France; 448 Paediatric Radiology, Hôpital Necker, Paris, France; 449 Rheumatology, Hôpital Cavale Blanche, Brest, France; 450 Institute of Immunology, Münster, Germany; 451 European Institute for Molecular Imaging, Münster, Germany; 452 Service de Medecine des Adolescents, Chu de Bicetre, Le Kremlin Bicetre Cedex, France; 453 Klinik für Pädiatrische Rheumatologie, Universitätsklinikum Münster, Münster, Germany; 454 Istituto Giannina Gaslini, Genova, Italy; 455 University Medical Center Utrecht, Utrecht, Netherlands; 456 Ospedale Pediatrico Bambino Gesù, Roma, Italy; 457 Università degli Studi di Genova, Genova, Italy; 458 Serviço de Reumatologia e Doenças Ósseas Metabólicas, Hospital de Santa Maria, Centro Hospitalar Lisboa Norte, Lisbon, Portugal; 459 Unidade de Reumatologia Pediátrica, Serviço de Reumatologia e Departamento de Pediatria, Hospital de Santa Maria, Centro Hospitalar Lisboa Norte, Lisbon, Portugal; 460 Instituto de Medicina Molecular, Faculdade de Medicina de Lisboa, Lisbon, Portugal; 461 National Centre for Paediatric Rheumatology, Our Lady’s Childrens Hospital, Dublin, Ireland; 462 National Centre for Paediatric Rheumatology, Our Lady’s Children Hospital Crumlin, Dublin, Ireland; 463 Pediatric Rheumatology, Hospital La Fe, Valencia, Spain; 464 Children’s Orthopedic and Rheumatologic Department, FSBI “The Turner Scientific and Research Institute for Children’s Orthopedics” under the Ministry of Health of the Russian Federation, Saint-Petersburg, Russian Federation; 465 Department of Foot Pathology, Neuroothopedics and Systemic Diseases, FSBI “The Turner Scientific and Research Institute for Children’s Orthopedics” under the Ministry of Health of the Russian Federation, Saint-Petersburg, Russian Federation; 466 Pediatric department n.a. I. M. Voroncov, Saint-Petersburg State Pediatric Medical University under the Ministry of Health of the Russian Federation, Saint-Petersburg, Russian Federation; 467 Pediatric Rheumatology, Erciyes University, Faculty of Medicine, Kayseri, Turkey; 468 Pediatrics, Faculty of Medicine Ramathibodi Hospital, Mahidol University, Bangkok, Thailand; 469 Radiology, Faculty of Medicine Ramathibodi Hospital, Mahidol University, Bangkok, Thailand; 470 Pediatrics, Aarhus University Hospital, Aarhus, Denmark; 471 Radiology, Aarhus University Hospital, Aarhus, Denmark; 472 Paediatrics, Hospital San José Tec de Monterrey, Monterrey, Mexico; 473 Paediatric Rheumatology, Hospital Universitario “Dr. José Eleuterio González”, Monterrey, Mexico; 474 Rheumatology, Hospital Universitario “Dr. José Eleuterio González”, Monterrey, Mexico; 475 Department of Pediatric Cardiology and Rheumatology, Medical University of Lodz, Lodz, Poland; 476 Clinical Hospital Center Sestre Milosrdnice, Zagreb, Croatia; 477 University of Zagreb School of Medicine, Zagreb, Croatia; 478 PRI - Pediatric Rheumatology Research Institute, Bad Bramstedt, Germany; 479 Image Analysis, London, UK; 480 Department of Radiology, Bispebjerg and Frederiksberg Hospital Copenhagen University, Copenhagen, Denmark; 481 State Institute of Pediatrics Obstetrics and Gynecology NAMS, Kyiv, Ukraine; 482 V.M. Glushkov Institute of Cybernetics of NAS of Ukraine, Kyiv, Ukraine; 483 National Military Medical Center of Ukraine, Kyiv, Ukraine; 484 Department of Biomedical, Surgical and Dental Sciences, University of Milan, Milano, Italy; 485 Department of Biomedical, Surgical and Dental Sciences, University of Milan, Milan, Italy; 486 Children’s Hospital Zagreb, Zagreb, Croatia; 487 Pathology, Stellenbosch University, National Health Laboratory Services, Cape Town, South Africa; 488 Stellenbosch University, Cape Town, South Africa; 489 University of Manchester, Manchester, UK; 490 Connective Children Diseases in Children, State Institute of Pediatrics Obstetrics and Gynecology NAMS of Ukraine, Kyiv, Ukraine; 491 Rheumatology, Mersin State Hospital; Keçiören Education and Research Hospital, Ankara, Turkey; 492 Rheumatology, Hitit University Medical Faculty, Ankara Numune Education and Research Hospital, Ankara, Turkey; 493 Pediatric Rheumatology, Tricase (LE), Italy; 494 Pediatric Rheumatology, Genova, Italy; 495 Pediatric Oncology, Bologna, Italy; 496 Pediatric Rheumatology, Torino, Italy; 497 Pediatric Rheumatology, De Marchi, Milano, Italy; 498 Pediatric Rheumatology, Trieste, Italy; 499 Pediatric Rheumatology, Brescia, Italy; 500 Pediatric Rheumatology, Messina, Italy; 501 Pediatric Rheumatology, Napoli, Italy; 502 Pediatric Rheumatology, G. Pini, Milano, Italy; 503 Pediatric Rheumatology, Ancona, Italy; 504 Pediatric Rheumatology, Orvieto, Italy; 505 Pediatric Rheumatology, Reggio Emilia, Italy; 506 Pediatric Rheumatology, Bambino Gesù, Roma, Italy; 507 Pediatric Rheumatology, Brindisi, Italy; 508 Pediatric Rheumatology, Cattolica, Roma, Italy; 509 Pediatric Rheumatology, Palermo, Italy; 510 Pediatric Rheumatology, Chieti, Italy; 511 Pediatric Rheumatology, Monza, Italy; 512 Pediatric Oncology, Monza, Italy; 513 Pediatric Oncology, Torino, Italy; 514 Pediatria II, Istituto Giannina Gaslini, Genova, Italy; 515 PRINTO, Istituto Giannina Gaslini, Genova, Italy; 516 Division of Rheumatology, Ospedale Pediatrico Bambin Gesù, Roma, Italy; 517 Immunology Diagnostic Unit, Ospedale Pediatrico Bambin Gesù, Roma, Italy; 518 Institute of Child Health, University College London, London, UK; 519 SingHealth Translational Immunology and Inflammation Centre, Singhealth and Duke-NUS Graduate Medical School, Singapore, Singapore; 520 Institute of Translational Medicine, University of Liverpool, Liverpool, UK; 521 Department of Paediatric Immunology, University Medical Centre Utrecht, Utrecht, Netherlands; 522 Division of Rheumatology, Hospital for Sick Children, University of Toronto, Toronto, Canada; 523 Murdoch Children’s Research Institute, Royal Children’s Hospital, Melbourne, Australia; 524 Arthritis Research UK Centre for Epidemiology, Centre for Musculoskeletal Research, University of Manchester, Manchester, UK; 525 Division of Rheumatology, Cincinnati Children’s Hospital Medical Center, Cincinnati, OH USA; 526 Department of Pediatrics, Standford University School of Medicine, Stanford, CA USA; 527 Paediatric Rheumatology, German Rheumatism Research Centre, Berlin, Germany; 528 Division of Immunology, Boston Children’s Hospital, Boston, MA USA; 529 Institute of Immunology, University of Munster, Munster, Germany; 530 Pediatria II Reumatologia, Instituto Giannina Gaslini, Genova, Italy; 531 Arthritis Research UK Centre for Genetics and Genomics, Centre for Musculoskeletal Research, University of Manchester, Manchester, UK; 532 Department of Pediatrics, Duke University Medical Center, Durham, NC USA; 533 Rheumatology, Immunology, Respiratory and Gastroenterology office, European Medicines Agency, London, UK; 534 Department of Medicine, University of Cambridge and MRC Biostatistics Unit, Cambridge, UK; 535 UCL Business PLC, University College London, London, UK; 536 Asklepios Clinic, Sankt Augustin, Germany; 537 Radboud University Nijmegen Medical Center, Nijmegen, Netherlands; 538 AbbVie Inc., North Chicago, IL USA; 539 AbbVie Deutschland GmbH & Co. KG, Ludwigshafen, Germany; 540 Connecticut Children’s Medical Center, Hartford, CT USA; 541 Rheumatology Unit-Dpt of Paediatrics University of Florence-Anna Meyer Children Hospital, Firenze, Italy; 542 Gaetano Pini Institute, Milano, Italy; 543 Institute for Maternal and Child Health - IRCCS “Burlo Garofolo”, University of Trieste, Trieste, Italy; 544 Gaetano Pini Institute, University of Milano, Milano, Italy; 545 Division of Rheumatology, Bambino Gesu’ Children’s Hospital, Rome, Italy; 546 Institute of Translational Pharmacology, Consiglio Nazionale delle Ricerche (CNR), Rome, Italy; 547 Department of Pediatric Rheumatology, Sophia Children’s Hospital, Erasmus University Medical Centre, Rotterdam, Netherlands; 548 Department of Pediatric Immunology and Rheumatology, Wilhelmina Children’s Hospital, University Medical Centre, Utrecht, Netherlands; 549 Department of Pediatric Rheumatology, Pequeno Príncipe Hospital, Curitiba, Brazil; 550 National Institute of Public Health and the Environment (RIVM), Bilthoven, Netherlands; 551 Department of Pediatrics, University of Medicine of Ribeirão Preto, São Paulo, Brazil; 552 Wilhelmina Children’s Hospital, University Medical Centre, Utrecht, Netherlands; 553 Pediatric Rheumatology Department, Hospital Pequeno Príncipe, Curitiba, Brazil; 554 Pediatric Immunology and Rheumatology Department, Wilhemina Children’s Hospital/University Medical Centre Utrecht, Utrecht, Netherlands; 555 Research Department, Istituto Giannina Gaslini, Genoa, Italy; 556 Dutch JIA patient organization, Wilhemina Children’s Hospital/University Medical Centre Utrecht, Utrecht, Netherlands; 557 Chair of European Network for Children with Arthritis (ENCA), Tipperary, Ireland; 558 Public Programmes Team, Central Manchester University Hospitals’ NHS Foundation Trust, Manchester, UK; 559 Centre for Musculoskeletal Research, University of Manchester, Manchester, UK; 560 Public Hospital, Montpellier, France; 561 CG Artists, Bordeaux, France; 562 ANDAR Patient Organization, Paris, France; 563 Epidemiology Unit, German Rheumatism Research Centre, Berlin, Germany; 564 Kinderklinik, Asklepios, Sankt Augustin, Germany; 565 Kinderklinik, Medizinischen Hochschule Hannover, Hannover, Germany; 566 Prof.-Hess-Kinderklinik, Klinikum Bremen-Mitte, Bremen, Germany; 567 Klinik für Kinder- und Jugendmedizin, Kinderkrankenhauses der Stadt Köln, Köln, Germany; 568 Hamburger Zentrum für Kinder- und Jugendrheumatologie, am Klinikum Eilbek, Hamburg, Germany; 569 Klinik für pädiatrische Rheumatologie und Immunologie, Universitätsklinikum Münster, Münster, Germany; 570 Department of Paediatrics, Rheumatology Unit, Anna Meyer Children’s Hospital, University of Florence, Florence, Italy; 571 Department of Paediatrics, Endocrinology Unit, Anna Meyer Children’s Hospital, University of Florence, Florence, Italy; 572 Pediatric Rheumatology, Care for Kids - Hospital La Misericordia - Instituto Roosevelt, Bogotá, Colombia; 573 Pediatric Rheumatology, Care for Kids - Hospital Infantil de San José, Bogotá, Colombia; 574 Pediatric Rheumatology, Care for Kids - Fundación Cardio Infantil - CAYRE, Bogotá, Colombia; 575 Reumatologia Pediatrica, Imss Hospital General Centro Medico Nacional La Raza, Azcapotzalco, Mexico; 576 Hematologia Pediatrica, Imss Hospital General Centro Medico Nacional La Raza, Azcapotzalco, Mexico; 577 Pediatrics, PGIMER, Chandigarh, India; 578 Histopathology, PGIMER, Chandigarh, India; 579 Pediatric Rheumatology, Dokuz Eylül University Hospital, İzmir, Turkey; 580 Paediatric Rheumatology Unit, Astrid Lindgren Children’s Hospital, Karolinska University Hospital, Stockholm, Sweden; 581 Hälsohögskolan, Jönköping, Sweden; 582 Pediatrics, University Hospital of St Etienne, St Etienne, France; 583 Pediatrics, University Hospital of St Etienne, Saint Etienne, France; 584 Pediatrics, University Hospital of Lausanne, Lausanne, Switzerland; 585 Pediatrics, University Hospital of Lyon, Lyon, France; 586 Occupational Therapy Musgrave Park Hospital, Belfast, UK; 587 Belfast Health & Social Care Trust, Belfast, UK; 588 Pediatric Rheumatology, Gulhane Military Medical Academy, Ankara, Turkey; 589 Pediatrics, Gulhane Military Medical Academy, Ankara, Turkey; 590 Third Department and Clinic of Paediatrics, Immunology and Rheumatology of Developmental Age, Wroclav Medical University, Wroclaw, Poland; 591 Department of Paediatric Pulmonology and Rheumatology, Medical University of Lublin, Lublin, Poland; 592 Hirszfeld Institute of Immunology and Experimental Therapy, Polish Academy of Sciences, Wroclaw, Poland; 593 Department of Paediatric Rheumatology, John Paul II Paediatric Centre, Sosnowiec, Poland; 594 Department of Pathogen Biology and Immunology, Institute of Genetics and Microbiology, Wroclav University, Wroclaw, Poland; 595 Department of Paediatric Pulmonology and Rheumatology, Children’s University Hospital, Lublin, Poland; 596 Pediatrics Department, Vasyl Karazin Kharkiv National University, Kharkiv, Ukraine; 597 Radiology, Hospital Sant Joan de Deu, Barcelona, Spain; 598 Rheumatology, Hospital Sant Joan de Deu, Barcelona, Spain; 599 Orthopedics, Hospital Sant Joan de Deu, Barcelona, Spain; 600 Rehabilitation, Hospital Sant Joan de Deu, Barcelona, Spain; 601 Pediatric Rheumatology Unit, Regional Universitary Hospital of Málaga, Málaga, Spain; 602 Pediatric Department, H. Materno-Infantil. HRU de Málaga, Málaga, Spain; 603 Rheumatology Department, Regional Universitary Hospital of Málaga, Málaga, Spain; 604 Paediatric Rheumatology Unit, Vall d’Hebron University Hospital, Barcelona, Spain; 605 Immunology, Vall d’Hebron University Hospital, Barcelona, Spain; 606 Rheumatology, Vall d’Hebron University Hospital, Barcelona, Spain; 607 CHU Clermont-Ferrand, Clermont-Ferrand, France; 608 APHP, Paris, France; 609 CHU Saint-Etienne, Saint-Priest en Jarrez, France; 610 University Hospital Leuven, Leuven, Belgium; 611 Dipartimento della Donna, del Bambino o del Neonato, Fondazione Irccs Cà Granda Ospedale Maggiore Policlinico, Milano, Italy; 612 Dipartimento di Fisiopatologia Medico Chirurgica e dei Trapianti, Fondazione Irccs Cà Granda Ospedale Maggiore Policlinico and Università di Milano, Milano, Italy; 613 Rheumatology, Royal Childrens Hospital, Melbourne, Australia; 614 Pediatric Rheumatology, Hospital Regional de Alta Especialidad del Bajío, Leon, Guanajuato Mexico; 615 Research, Hospital Regional de Alta Especialidad del Bajío, Leon, Guanajuato Mexico; 616 Public Hospital, Montpellier, France; 617 Public Hospital, Lyon, France; 618 KOURIR Patient Organization, Paris, France; 619 Public Hospital Necker, Paris, France; 620 Public Hospital, Strasbourg, France; 621 ANDAR Patient Organization, Paris, France; 622 Occupational Therapy Clinic, Children’s Occupational Therapy Ward, Stockholm, Sweden; 623 Center for Rehabilitation, UMCG, Groningen, Netherlands; 624 Department of Pediatric Rheumatology, Beatrix Children’s Hospital, UMCG, Groningen, Netherlands; 625 Department of Pediatric Immunology, Wilhelmina Children’s Hospital, UMCU, Groningen, Netherlands; 626 Child Development & Exercise Center, Wilhelmina Children’s Hospital, UMCU, Utrecht, Netherlands; 627 Center for Rehabilitation and Rheumatology, Reade, Amsterdam, Netherlands; 628 Center for Rehabilitation and Dept. of Oral and Maxillofacial Surgery, UMCG, Utrecht, Netherlands; 629 Beatrix Children’s Hospital, UMCG, Groningen, Netherlands; 630 Department of Pediatrics Rheumatology, Kocaeli University Faculty of Medicine, Kocaeli, Turkey; 631 Department of Orthopaedics and Traumatology, Kocaeli University Faculty of Medicine, Kocaeli, Turkey; 632 Department of Radiology, Kocaeli University Faculty of Medicine, Kocaeli, Turkey; 633 Department of Pathology, Kocaeli University Faculty of Medicine, Kocaeli, Turkey; 634 Scientific Center Of The Family Health And Human Reproduction Problems, Irkutsk, Russian Federation; 635 Department of Pediatrics, University of Chieti, Chieti, Italy; 636 Department of Radiology, University of Chieti, Chieti, Italy; 637 Rheumatology, Institute of Child Health, University College London, London, UK; 638 Rheumatology, Royal National Hospital For Rheumatic Diseases, Bath, UK; 639 Clinical and Academic Rheumatology, North Bristol NHS Trust, Southmead Hospital, Bristol, UK; 640 Department of Pharmacy and Pharmacology, University of Bath, Bath, UK; 641 Hôptal H.Mondor, Creteil, Paris, France; 642 Hôpital Necker, Paris, France; 643 Hôpital La Pitié, Paris, France; 644 Rheumatology, Nationwide Childrens/Ohio State, Columbus, USA; 645 Radiology, Nationwide Childrens/Ohio State, Columbus, USA; 646 Research Institute, Nationwide Childrens/Ohio State, Columbus, USA; 647 University of Adelaide, Adelaide, Australia; 648 University College London Institute of Child Health, London, UK; 649 Alder Hey Childrens Hospital, Liverpool, UK; 650 University College Hospital, London, UK; 651 Great Ormond Street Hospital for Children, London, UK; 652 Pediatric Rheumatology and Immunology, University Hospital Münster, Münster, Germany; 653 Department of Pediatric Oncology, Hematology and Immunology, University Children’s Hospital, Medical Faculty, Heinrich-Heine-University, Düsseldorf, Germany; 654 German Center for Paediatric Rheumatology and Immunology, Garmisch-Partenkirchen, Germany; 655 Pediatric Rheumatology and Immunology, University Hospital Münster, Münster, Germany; 656 Department of Pediatric Oncology, Hematology and Immunology, University Children’s Hospital, Medical Faculty, Heinrich-Heine-University, Düsseldorf, Germany; 657 German Center for Paediatric and Adolescent Rheumatology, Garmisch-Partenkirchen, Germany; 658 German Center for Paediatric and Adolescent Rheumatology, Garmisch-Partenkirchen, Germany; 659 Pediatric Rheumatology and Immunology, University Hospital Münster, Münster, Germany; 660 Pediatria II, Reumatologia; PRINTO, Istituto Giannina Gaslini, Genoa, Italy; 661 Department of Pediatrics, Hacettepe University, Faculty of Medicine, Ankara, Turkey; 662 Division of Pediatric Rheumatology, Hacettepe University, Ankara, Turkey; 663 Division of Pediatric Neurology, Hacettepe University, Faculty of Medicine, Department of Pediatrics, Ankara, Turkey; 664 Pediatria II, Reumatologia; PRINTO, Istituto Giannina Gaslini, Genoa, Italy; 665 Pediatrics Rheumatology, Hospital Infantil de Mexico Federico Gomez, Ciudad de Mexico, Mexico; 666 Pediatric Rheumatology, Pediatric Specialists of Virginia, Fairfax, USA; 667 Radiology, Fairfax Radiological Consultants PC, Fairfax, USA; 668 Pediatric Gastroenterology, Pediatric Specialists of Virginia, Fairfax, USA; 669 Paediatric Rheumatology, University of Nairobi, Nairobi, Kenya; 670 Paediatric Neurology, University of Cape Town, Cape Town, South Africa; 671 Paediatric Immunology and Rheumatology, University of Stellenbosch, Cape Town, South Africa; 672 Paediatric Rheumatology, University of Cape Town, Cape Town, South Africa; 673 Department of Pediatrics, Division of Rheumatology, Hacettepe University Faculty of Medicine, Ankara, Turkey; 674 Department of Pediatrics, Division of Pulmonology, Hacettepe University Faculty of Medicine, Ankara, Turkey; 675 Department of Radiology, Hacettepe University Faculty of Medicine, Ankara, Turkey; 676 Pediatrics, Okayama University Hospital, Okayama, Japan; 677 King Faisal Specialist Hospital & Research Center, Riyadh, Saudi Arabia; 678 King Faisal Specialist Hospital, Riyadh, Saudi Arabia; 679 King Abdulaziz University, Jeddah, Saudi Arabia; 680 Maternity & Children Hospital, Jeddah, Saudi Arabia; 681 Queen Rania Children Hospital, Amman, Jordan; 682 Qaboos Sultan University, Muscat, Oman; 683 Royal Hospital, Muscat, Oman; 684 Department of Pediatric Pulmonology and Rheumatology, Medical University of Lublin, Poland, Lublin, Poland; 685 Pediatrics, PGIMER, Chandigarh, India; 686 Immunopathology, PGIMER, Chandigarh, India; 687 Pediatric Rheumatology, El Bosque University, Bogota, Colombia; 688 Pediatric Rheumatology, Universidad Libre, Cali, Colombia; 689 Pediatric Rheumatology, Universidad de Cartagena, Cartagena, Colombia; 690 Pediatric Rheumatology, Universidad Del Valle, Cali, Colombia; 691 Pediatric Rheumatology Unit, University of Padua, Padua, Italy; 692 Pediatric Cardiology Unit, University of Padua, Padua, Italy; 693 Am Schön Klinik Eilbek, Hamburg, Germany; 694 Pediatric Rheumatology, Berlin, Germany; 695 Pediatric Rheumatology, Istanbul, Turkey; 696 Pediatric Rheumatology, Riga, Latvia; 697 Pediatric Rheumatology, Sao Paulo, Brazil; 698 Pediatric Rheumatology, Moskva, Russian Federation; 699 Pediatric Rheumatology, Buenos Aires, Argentina; 700 Pediatric Rheumatology, Florence, Italy; 701 Pediatric Rheumatology, St. Petersburg, Russian Federation; 702 Pediatric Rheumatology, New York, USA; 703 Pediatric Rheumatology, Madrid, Spain; 704 Pediatric Rheumatology, Gent, Belgium; 705 Pediatric Rheumatology, Rio de Janeiro, Brazil; 706 Pediatric Rheumatology, Ljubljana, Slovenia; 707 Pediatric Rheumatology, Almada, Portugal; 708 Pediatric Rheumatology, Prague, Czech Republic; 709 Pediatric Rheumatology, Santa Fee, Argentina; 710 Pediatric Rheumatology, London, UK; 711 Pediatric Rheumatology, Nettnja, Israel; 712 Pediatric Rheumatology, Chennai, India; 713 Pediatric Rheumatology, Barcelona, Spain; 714 Pediatric Rheumatology, Copenhagen, Denmark; 715 Pediatric Rheumatology, Pittsburgh, USA; 716 Pediatric Rheumatology, Kfar Saba, Israel; 717 Am Schön Klinik Eilbek, Hamburg, Germany; 718 Pediatric Rheumatology, Liverpool, UK; 719 Pediatric Rheumatology, Indianapolis, USA; 720 Pediatric Rheumatology, London, UK; 721 Pediatric Rheumatology, Prague, Czech Republic; 722 Pediatric Rheumatology, Firenze, Italy; 723 Pediatric Rheumatology, Pittsburgh, USA; 724 Pediatric Rheumatology, Zürich, Switzerland; 725 Am Schön Klinik Eilbek, Hamburg, Germany; 726 Pediatric Rheumatology, Berlin, Germany; 727 Pediatric Rheumatology, Istanbul, Turkey; 728 Pediatric Rheumatology, Riga, Latvia; 729 Pediatric Rheumatology, Sao Paulo, Brazil; 730 Pediatric Rheumatology, Moskva, Russian Federation; 731 Pediatric Rheumatology, Buenos Aires, Argentina; 732 Pediatric Rheumatology, Florence, Italy; 733 Pediatric Rheumatology, St. Petersburg, Russian Federation; 734 Pediatric Rheumatology, New York, USA; 735 Pediatric Rheumatology, Madrid, Spain; 736 Pediatric Rheumatology, Gent, Belgium; 737 Pediatric Rheumatology, Rio de Janeiro, Brazil; 738 Pediatric Rheumatology, Ljubljana, Slovenia; 739 Pediatric Rheumatology, Almada, Portugal; 740 Pediatric Rheumatology, Prague, Czech Republic; 741 Pediatric Rheumatology, Santa Fee, Argentina; 742 Pediatric Rheumatology, London, UK; 743 Pediatric Rheumatology, Nettnja, Israel; 744 Pediatric Rheumatology, Chennai, India; 745 Pediatric Rheumatology, Barcelona, Spain; 746 Pediatric Rheumatology, Copenhagen, Denmark; 747 Pediatric Rheumatology, Pittsburgh, USA; 748 Pediatric Rheumatology, Kfar Saba, Israel; 749 Pediatric Department, I.M.Sechenov First Moscow State Medical University, Moscow, Russian Federation; 750 Pediatric Department, Pediatric Outpatien Clinic of Public Joint-stock Company Gazprom, Moscow, Russian Federation; 751 Pediatric Rheumatology, Istanbul University, Cerrahpasa Medical School, Istanbul, Turkey; 752 Biochemistry, Istanbul University, Cerrahpasa Medical School, Istanbul, Turkey; 753 Am Schön Klinik Eilbek, Hamburg, Germany; 754 Pediatric Rheumatology, Berlin, Germany; 755 Pediatric Rheumatology, Istanbul, Turkey; 756 Pediatric Rheumatology, Riga, Latvia; 757 Pediatric Rheumatology, Sao Paulo, Brazil; 758 Pediatric Rheumatology, Moskva, Russian Federation; 759 Pediatric Rheumatology, Buenos Aires, Argentina; 760 Pediatric Rheumatology, Florence, Italy; 761 Pediatric Rheumatology, St. Petersburg, Russian Federation; 762 Pediatric Rheumatology, New York, USA; 763 Pediatric Rheumatology, Madrid, Spain; 764 Rheumatology and Internal Medicine, Gent University Hospital, Gent, Belgium; 765 Pediatric Rheumatology, Rio de Janeiro, Brazil; 766 Pediatric Rheumatology, Ljubljana, Slovenia; 767 Pediatric Rheumatology, Almada, Portugal; 768 Pediatric Rheumatology, Prague, Czech Republic; 769 Pediatric Rheumatology, Santa Fee, Argentina; 770 Pediatric Rheumatology, London, UK; 771 Pediatric Rheumatology, Nettnja, Israel; 772 Pediatric Rheumatology, Chennai, India; 773 Pediatric Rheumatology, Barcelona, Spain; 774 Pediatric Rheumatology, Copenhagen, Denmark; 775 Pediatric Rheumatology, Pittsburgh, USA; 776 Pediatric Rheumatology, Kfar Saba, Israel; 777 Rheumatology Unit, Second University of Naples, Naples, Italy; 778 Epidemiology Unit, Regional Health Agency of Tuscany, Florence, Italy; 779 Epidemiology Unit, Anna Meyer Children’s University Hospital, Florence, Italy; 780 Rheumatology Unit, Anna Meyer Children’s Hospital, Florence, Italy; 781 Pediatric Rheumatology, Hospital Infantil De Mexico Federico Gomez, Mexico City, Mexico; 782 Pediatric Infectology, Hospital Infantil De Mexico Federico Gomez, Mexico City, Mexico; 783 Aou G.Martino, Messina, Italy; 784 Istituto Gaslini, Genova, Italy; 785 Policlinico Le Scotte, Siena, Italy; 786 Clinica Pediatrica, Brescia, Italy; 787 Ospedale Maggiore, Clinica Pediatrica II De Marchi, Milano, Italy; 788 Ospedale Bambin Gesù, Roma, Italy; 789 II Università degli Studi di Napoli, Napoli, Italy; 790 Policlinico Gemelli, Roma, Italy; 791 Ospedale Salus Pueri, Padova, Italy; 792 Ospedale Federico II, Napoli, Italy; 793 Ospedale Meyer, Firenze, Italy; 794 Allergy Immunology Unit, Advanced Pediatrics Centre, Chandigarh, India; 795 Cardiology, Post Graduate Institute of Medical Education and Research, Chandigarh, India; 796 Allergy immunology Unit, Advanced Pediatrics Centre, Post Graduate Institute of Medical Education and Research, Chandigarh, India; 797 Cardiology, Post Graduate Institute of Medical Education and Research, Chandigarh, India; 798 Division of Biochemistry, Advanced Pediatrics Centre, Chandigarh, India; 799 Infection, Immunity, Inflammation and Physiological Medicine, Institute of Child Health, UCL, London, UK; 800 UCL Centre for Nephrology, Royal Free Hospital, London, UK; 801 Vasculitis and Lupus Clinic, Addenbrookes Hospital, Cambridge, UK; 802 School of Medicine, Trinity College Dublin, Dublin, UK; 803 Pediatric Department, I.M. Sechenov First Moscow State Medical University, Moscow, Russian Federation; 804 Department of Childhood Diseases, I.M.Sechenov First Moscow State Medical University, Moscow, Russian Federation; 805 Rheumatology Unit, Second University of Naples, Naples, Italy; 806 Rheumatology, Nationwide Childrens/Ohio State, Columbus, OH USA; 807 Rheumatology, Nationwide Childrens/Ohio State, Columbus, USA; 808 Nephrology, Nationwide Childrens/Ohio State, Columbus, USA; 809 Pediatrics, Gulhane Military Medical Academy, Ankara, Turkey; 810 Pediatric, Gulhane Military Medical Academy, Ankara, Turkey; 811 Department of Pediatrics and Adolescent Medicine, First Faculty of Medicine, Charles University and General University Hospital, Prague, Czech Republic; 812 Pediatric Rheumatology Service, 2nd Department of Pediatrics, Comenius University Medical School and University Children’s Hospital, Bratislava, Slovakia; 813 Department of Pediatric Hematology and Oncology, Charles University and University Hospital Motol, Prague, Czech Republic; 814 Department of Radiology, Second Faculty of Medicine and University Hospital Motol, Prague, Czech Republic; 815 Department of Nuclear Medicine, First Faculty of Medicine, Charles University and General University Hospital, Prague, Czech Republic; 816 Department of Nephrology, First Faculty of Medicine, Charles University and General University Hospital, Prague, Czech Republic; 817 Department of Pediatrics, Division of Rheumatology, Hacettepe University Faculty of Medicine, Ankara, Turkey; 818 Department of Radiology, Hacettepe University Faculty of Medicine, Ankara, Turkey; 819 Department of Pediatrics, Division of Nephrology, Hacettepe University Faculty of Medicine, Ankara, Turkey; 820 Pediatric Rheumatology, Irmandade da Santa Casa de Misericórdia de São Paulo- Brasil, São Paulo, Brazil; 821 Pediatric Neurology, Irmandade da Santa Casa de Misericórdia de São Paulo- Brasil, São Paulo, Brazil; 822 Department of Internal Medicine, Section of Rheumatology, Transition Clinic, Florence, Italy; 823 Department of Surgery and Translational Medicine, Florence, Italy; 824 Health Sciences Department, Anna Meyer Children’s University Hospital, University of Florence, Florence, Italy; 825 Institute of Pediatrics, Università Cattolica Sacro Cuore University of Rome, Rome, Italy; 826 Department of Internal Medicine, Section of Rheumatology, Transition Clinic, University of Florence, Florence, Italy; 827 Dipartimento della Donna, del Bambino o del Neonato, Fondazione Irccs Cà Granda Ospedale Maggiore Policlinico, Milano, Italy; 828 Department of Pediatrics, Karadeniz Technical University, Faculty of Medicine, Trabzon, Turkey; 829 Department of Biochemistry, Cumhuriyet University Faculty of Pharmacy, Sivas, Turkey; 830 Department of Medical Biochemistry, Karadeniz Technical University, Faculty of Medicine, Trabzon, Turkey; 831 Department of Nutrition and Dietetics, Karadeniz Technical University, Faculty of Health Sciences, Trabzon, Turkey; 832 Department of Pediatric Rheumatology, Karadeniz Technical University, Faculty of Medicine, Trabzon, Turkey; 833 Pediatric Rheumatology, Irmandade Santa Casa de Misericordia de São Paulo, São Paulo, Brazil; 834 Pediatric, Irmandade Santa Casa de Misericordia de São Paulo, São Paulo, Brazil; 835 Pediatric Rheumatology Unit, Department of Pediatrics, Hospital Sant Joan de Déu, Esplugues de Llobregat, Barcelona Spain; 836 Department of Pediatric Cardiology, Hospital Sant Joan de Déu, Esplugues de Llobregat, Barcelona Spain; 837 Paediatric Rheumatology, Leeds General Infirmary, Leeds Teaching Hospitals Trust, Leeds, UK; 838 Paediatric Rheumatology, Leeds General Infirmary, Leeds Teaching Hospitals Trust, Prague, Czech Republic; 839 Immuno-Rheumatology Center, St. Luke’s International Hospital, Tokyo, Japan; 840 Fujikawa Pediatric Clinic, Tokyo, Japan; 841 Pediatrics, Division of Rheumatology, College of Medicine, Yonsei University, Severance Children’s Hospital, Seoul, Republic of Korea; 842 Department of Dermatology, College of Medicine, Yonsei University, Seoul, Republic of Korea; 843 M. Iashvili Children’s Central Hospital, Tbilisi, Georgia; 844 Department of Pediatrics, School of Medicine, Institute of Medical Pharmaceutical and Health Sciences, Kanazawa University, Kanazawa, Japan; 845 University Department Pro.Sa.M.I. “G. D’Alessandro”, University of Palermo, Palermo, Italy; 846 Inserm 1018, CESP, Kremlin Bicêtre, Paris, France; 847 Pediatric Rheumatology, CHU de Bicêtre, Paris, France; 848 Université Paris Sud, Le Kremlin Bicêtre, Paris, France; 849 Internal Medecine, CHU Saint Louis, Paris, France; 850 Pediatrics, CHIC, Créteil, Paris, France; 851 Pediatrics, CHU Trousseau, Paris, France; 852 Pediatrics, CH Sainte Camille, Bry sur Marne, Paris, France; 853 Pediatrics, CHIV, Villeneuve Saint Georges, Paris, France; 854 Pediatrics, CHU de Bicêtre, Paris, France; 855 Pediatric Rheumatology, CHU de Bicêtre, Paris, France; 856 Université Paris Sud, Kremlin Bicêtre, Paris, France; 857 Université Paris 7-Diderot, ECSTRA, Paris, France; 858 2nd Department of Paediatrics, Iuliu Hatieganu University of Medicine and Pharmacy, Cluj-Napoca, Romania; 859 Rheumatology Department, Emergency Clinical County Hospital, Cluj-Napoca, Romania; 860 3rd Department of Internal Medicine, Iuliu Hatieganu University of Medicine and Pharmacy, Cluj-Napoca, Romania; 861 Pediatric Rheumatology, Hospital Infantil de Mexico Federico Gomez, Mexico City, Mexico; 862 Pediatric Rheumatology, El Bosque University, Bogota, Colombia; 863 Pediatrics, PGIMER, Chandigarh, India; 864 Immunopathology, PGIMER, Chandigarh, India; 865 Allergy immunology Unit, Advanced Pediatrics Centre, Chandigarh, India; 866 Cardiology, Post Graduate Institute of Medical Education and Research, Chandigarh, India; 867 Paediatric Rheumatology, Hospital Infantil de México Federico Gómez, Mexico City, Mexico; 868 Pediatric Department, I.M. Sechenov First Moscow State Medical University, Moscow, Russian Federation; 869 Department of Pediatrics Rheumatology, Kocaeli Univertsity Faculty of Medicine, Kocaeli, Turkey; 870 Pediatric Rheumatology, Ankara University, Ankara, Turkey; 871 Pediatric Radiology, Ankara University, Ankara, Turkey; 872 Peadiatric Rheumatology, Karolinska Institutet, Stockholm, Sweden; 873 Peadiatric rheumatology, Instituto Giannina Gaslini, Genoa, Italy; 874 Peadiatric Rheumatology, Institute of Child Health, Kolkata, India; 875 Peadiatric rheumatology, Hospital Sant Joan de Deu, Barcelona, Spain; 876 Pediatric oncology, Arkansas Children’s Hospital, Little Rock, USA; 877 Pediatric Oncology, Hospital Infantil de Mexico City, Mexico City, Mexico; 878 Pediatric Rheumatology, University of Istanbul, Istanbul, Turkey; 879 Pediatric Oncology, Stanford, Palo Alto, USA; 880 Pediatric Rheumatology, Athens Medical School, Athens, Greece; 881 Pediatric Rheumatology, University of Alabama at Birmingham, Birmingham, USA; 882 Pediatric Rheumatology, Instituto Giannina Gaslini, Genoa, Italy; 883 Pediatric Pneumology and Immunology, Charité University Medicine Berlin, Berlin, Germany; 884 Department of Pediatrics, University Clinic Carl Gustav Carus, Dresden, Germany; 885 Pediatric Oncology and Hematology, Charité University Medicine Berlin, Berlin, Germany; 886 Department of Rheumatology and Clinical Immunology, Charité University Medicine Berlin, Berlin, Germany; 887 Institute of Medical Genetics and Human Genetics, Charité University Medicine Berlin, Berlin, Germany; 888 Pediatric Rheumatology, Erciyes University Faculty of Medicine, Kayseri, Turkey; 889 Pediatric Rheumatology, Istanbul Kanuni Sultan Suleyman Education and Research Hospital, Istanbul, Turkey; 890 Pediatric Rheumatology, Hacettepe University Faculty of Medicine, Ankara, Turkey; 891 Pediatric Rheumatology, Dokuz Eylul University Faculty of Medicine, Izmir, Turkey; 892 Pediatric Rheumatology, Cerrahpasa Medical Faculty, Istanbul University, Istanbul, Turkey; 893 Pediatric Rheumatology, Gulhane Military Medical Faculty, FMF Arthritis Vasculitis and Orphan Disease Research Center, Ankara, Turkey; 894 Pediatric Rheumatology, Uludag University Faculty of Medicine, Bursa, Turkey; 895 Pediatric Rheumatology, Kocaeli University Faculty of Medicine, Kocaeli, Turkey; 896 Pediatric Rheumatology, Capa Medical Faculty, Istanbul University, Istanbul, Turkey; 897 Pediatrics, Faculty of Medicine Ramathibodi Hospital, Mahidol University, Bangkok, Thailand; 898 Department of Pediatric Rheumatology and Immunology, University Children’s Hospital Muenster, Muenster, Germany; 899 Prof.-Hess Children’s Hospital and Gesundheit Nord Klinikverbund Bremen, Bremen, Germany; 900 Asklepios Clinic Sankt Augustin, Sankt Augustin, Germany; 901 German Center for Pediatric and Adolescent Rheumatology, Garmisch-Partenkirchen, Germany; 902 St. Josef-Stift Sendenhorst Hospital, Sendenhorst, Germany; 903 Department of Pediatrics, Division of Pediatric Rheumatology, University Children’s Hospital Tuebingen, Tuebingen, Germany; 904 German Pediatric Pain Centre, Children’s and Adolescents’ Hospital, Datteln, Germany; 905 Institute of Immunology, University Hospital Muenster, Muenster, Germany; 906 Paediatric Rheumatology and Immunology, University of Muenster, Muenster, Germany; 907 UCD Conway Institute of Biomolecular and Biomedical Research, University College Dublin, Dublin, Ireland; 908 ASKLEPIOS, Sankt Augustin, Germany; 909 Helios Clinic, Vogelsang, Germany; 910 ASKLEPIOS, Sankt Augustin, Germany; 911 Charite, Berlin, Germany; 912 Prof. Hess Children’s Hospital, Bremen, Germany; 913 Olgahospital, Stuttgart, Germany; 914 German Centre Pediatric and Adolescent Rheumatology, Garmisch-Partenkirchen, Germany; 915 Laboratory of Immunobiology, Rega Institute, University of Leuven, Leuven, Belgium; 916 Autoimmune Genetics Laboratory, VIB, Ghent, Belgium; 917 Laboratory of Clinical and Epidemiological Virology, Rega Institute, University of Leuven, Leuven, Belgium; 918 Laboratory of Experimental Transplantation, KU Leuven - University of Leuven, Leuven, Belgium; 919 Department of Pediatrics, University Hospitals Leuven, Leuven, Belgium; 920 Department of Clinical Chemistry, Ghent University, Ghent, Belgium; 921 Laboratory of Neuroimmunology, KU Leuven - University of Leuven, Leuven, Belgium; 922 Pediatric Rheumatology, University Hospitals Leuven, Leuven, Belgium; 923 Laboratory of Pediatric Immunology, KU Leuven - University of Leuven, Leuven, Belgium; 924 Department Of Pediatrics, School Of Medicine, Institute of Medical Pharmaceutical and Health Sciences, Kanazawa University, Kanazawa, Japan; 925 Department of Pediatric Rheumatology, Hyogo Prefectural Kobe Children’s Hospital, Kobe, Japan; 926 Department of Pediatrics, School of Medicine, Institute of Medical Pharmaceutical and Health Sciences, Kanazawa University, Kanazawa, Japan; 927 Tranlational Genetics and Genomics Unit, National Institute of Arthritis and Musculoskeletal and Skin Diseases, Bethesda, USA; 928 Inflammatory Disease Section, National Human Genome Research Institute, Bethesda, USA; 929 Arthritis Research UK Centre for Genetics and Genomics, Manchester Academic Health Science Centre, Manchester, UK; 930 Center of Paediatric and Adolescent Rheumatology, University College London, London, UK; 931 NIHR Manchester Musculoskeletal Biomedical Research Unit, Central Manchester NHS Foundation Trust, Manchester Academic Health Science Centre, Manchester, UK; 932 Pediatric Rheumatology, University Clinical Centar Republic of Srpska, Banjaluka, Bosnia and Herzegovina; 933 Pediatric Hematology, University Clinical Centar Republic of Srpska, Banjaluka, Bosnia and Herzegovina; 934 Pediatric Pulmology, University Clinical Centar Republic of Srpska, Banjaluka, Bosnia and Herzegovina; 935 German Center for Pediatric and Adolescent Rheumatology, Garmisch-Partenkirchen, Germany; 936 Pediatrics, P & A Kyriakou Children’s Hospital, Athens, Greece; 937 Pediatrics, Makarios III Children’s Hospital, Nicosia, Cyprus; 938 Pediatrics, P & A Kyriakou Children’s Hospital, Athens, Greece; 939 Paediatric and Adolescent Rheumatology, Great Ormond Street Hospital, London, UK; 940 ASKLEPIOS, Sankt Augustin, Germany; 941 Helios, Vogelsang, Germany; 942 University, Muenster, Germany; 943 Pediatrics, Children hospital A. Harouchi, CHU Ibn Rochd, Casablanca, Morocco; 944 M. Iashvili Children’s Central Hospital, Tbilisi, Georgia; 945 Tallinn Childrens’Hospital, Tallinn, Estonia; 946 Children’s Clinic, Tartu University Hospital, Tartu, Estonia; 947 Children’s Clinic, Institute of Clinical Medicine, Tartu University, Tartu, Estonia

## Recurrent fevers

### O1 Efficacy and safety of Canakinumab in patients with periodic fever syndromes (colchicine-resistant fmf, hids/mkd and traps): results from a phase 3, pivotal, umbrella trial

#### F. De Benedetti^1^, J. Anton^2^, M. Gattorno^3^, H. Lachmann^4^, I. Kone-Paut^5^, S. Ozen^6^, J. Frenkel^7^, A. Simon^8^, A. Zeft^9^, E. Ben-Chetrit^10^, H.M. Hoffman^11^, Y. Joubert^12^, K. Lheritier^12^, A. Speziale^12^, J. Guido^12^

##### ^1^IRCCS Ospedale Pediatrico Bambino Gesú, Rome, Italy; ^2^Hospital Sant Joan de Déu, Barcelona, Spain; ^3^Pediatric Rheumatology, G. Gaslini Institute, Genoa, Italy; ^4^UK National Amyloidosis Centre, University College London Medical School, London, United Kingdom; ^5^Hôpital Kremlin Bicetre, University of Paris SUD, Paris, France; ^6^ Department of Pediatrics, Hacettepe University, Ankara, Turkey; ^7^University Medical Center Utrecht, Utrecht, Netherlands; ^8^Radboud University Medical Centre, Nijmegen, Netherlands; ^9^Pediatrics Rheumatology, Cleveland Clinic, Cleveland, USA; ^10^Rheumatology Unit, Hadassah—Hebrew University Medical Center, Jerusalem, Israel; ^11^University of California, San Diego, La Jolla, USA; ^12^Novartis Pharma AG, Basel, Switzerland

###### **Presenting author:** F. De Benedetti

**Introduction:** Periodic fever syndromes (PFS) are rare auto-inflammatory conditions that include, among others, cryopyrin-associated periodic syndromes (CAPS), familial Mediterranean fever (FMF), hyper-IgD syndrome/mevalonate kinase deficiency (HIDS/MKD), TNF receptor-associated periodic syndrome (TRAPS) [1]. Canakinumab (CAN), a fully human, highly specific anti-IL-1β neutralising monoclonal antibody, is effective in CAPS [2]. IL-1β has been shown to be involved in the pathogenesis of FMF, HIDS/MKD and TRAPS, for which no or limited treatment options exist [1]. Open-label studies have suggested the efficacy of CAN in colchicine-resistant/intolerant FMF (crFMF), HIDS/MKD and TRAPS [3-5]. We report the efficacy and safety of CAN from a randomised treatment epoch of a Phase 3 pivotal study in patients (pts) with crFMF, HIDS/MKD or TRAPS.

**Objectives:** Primary objective was to demonstrate that CAN 150 mg (or 2 mg/kg for pts ≤40 kg) sc q4w is superior to placebo (PBO) in achieving a clinically meaningful response defined as resolution of the index flare at Day 15 and no new disease flares over 16 weeks (wks) of treatment. Secondary objectives included proportion of pts who achieved a physician global assessment (PGA) of disease activity <2 (minimal/none) and proportions of pts with C-reactive protein (CRP) ≤10 mg/L and serum amyloid A (SAA) ≤10 mg/L at Wk 16.

**Methods:** The study consists of 3 disease cohorts (crFMF, HIDS/MKD and TRAPS) and 4 study epochs: a screening epoch (E1) of up to 12 wks, a randomised treatment epoch (E2) of 16 wks, a randomised withdrawal epoch (E3) of 24 wks and an open-label treatment epoch (E4) of 72 wks. Pts (aged ≥2 years) with a flare during E1 were randomised (1:1) in E2 to receive CAN or PBO. Safety assessments included adverse events (AEs) and serious AEs (SAEs).

**Results:** Of 181 pts (crFMF, n = 63; HIDS/MKD, n = 72; TRAPS, n = 46) randomised in E2, 6 discontinued (5 PBO; 1 CAN). In all 3 disease cohorts, the proportion of responders for the primary outcome at Wk 16 was significantly higher with CAN vs PBO (Table). At Wk 16, a significantly higher proportion of pts achieved a PGA score <2, CRP ≤10 mg/L and SAA ≤10 mg/L in the CAN group vs PBO in all 3 cohorts. The most frequently affected system organ class across 3 cohorts was infections and infestations typically involving the upper respiratory tract. The incidence of SAEs was 8.6%, 4.7% and 11.8% in crFMF, TRAPS and HIDS/MKD cohort, respectively.

**Conclusion:** These results demonstrated superior efficacy of canakinumab after a 16-week treatment period compared with placebo. The overall safety profile was not distinct from those reported in previous controlled studies.

**References**

1. Savic S and Wood P. *Clin Med.* 2011;11(4):396–401.

2. Kuemmerle-DeschnerJB, et al. *Ann Rheum Dis*. 2015;74:850.

3. Brik R, et al. *Ann Rheum Dis*. 2013;72:75.

4. Arostegui, et al. *Ann Rheum Dis*. 2015;74: 401.

5. Lachmann, et al. *Ann Rheum Dis*. 2015;74: 852.

**Trial registration identifying number:** NCT02059291

**Disclosure of Interest**

F. De Benedetti Grant / Research Support from: Pfizer, Abbvie, Roche, Novartis, Novimmune and BMS, J. Anton Grant / Research Support from: Novartis, Consultant for: Novartis, M. Gattorno Grant / Research Support from: Novartis and SOBI, Consultant for: Novartis and SOBI, H. Lachmann Consultant for: Novartis, SOBI, Takeda and GSK, Speaker Bureau of: Novartis and SOBI, I. Kone-Paut Grant / Research Support from: Novartis, SOBI and Roche, Consultant for: Novartis, SOBI, Pfizer, Abbvie, Roche, S. Ozen Speaker Bureau of: Novartis and SOBI, J. Frenkel Grant / Research Support from: Novartis and SOBI, A. Simon Grant / Research Support from: Novartis, CSL, Behring and Xoma/Servier, Consultant for: Novartis, Takeda, Xoma and SOBI, A. Zeft Shareholder of: Merck, Opko Health, Arno therapeutics, Consultant for: Novartis, E. Ben-Chetrit Consultant for: Novartis, H. Hoffman Consultant for: Novartis, Speaker Bureau of: Novartis, Y. Joubert Employee of: Novartis, K. Lheritier Employee of: Novartis, A. Speziale Employee of: Novartis, J. Guido Employee of: Novartis

**Table 1 (abstract O1).** Proportion of responders at Week 16 by cohort

Cohort	Canakinumab (150 mg q4w), n/m (%)	Placebo n/m (%)	p-value
crFMF	19/31 (61.3)	2/32 (6.3)	<0.001*
HIDS/MKD	13/37 (35.1)	2/35 (5.7)	0.0020*
TRAPS	10/22 (45.5)	2/24 (8.3)	0.0050*

*n* number of patients who responded, *m* number of patients evaluated for response

*Statistical significance (1-sided) at the 0.025 level based on Fisher exact test/logistic regression model

### O2 An update on the Italian cohort of DADA2 patients and an assessment of a novel functional screening test

#### Roberta Caorsi^1^, Federica Penco^1^, Alice Grossi^2^, Antonella Insalaco^3^, Maria Alessio^4^, giovanni Conti^5^, Federico Marchetti^6^, Alberto Tommasini^7^, Silvana Martino^8^, Romina Gallizzi^9^, Annalisa Salis^10^, Francesca Schena^1^, Francesco Caroli^2^, Alberto Martini^11^, Gianluca Damonte^10^, Isabella Ceccherini^2^, Marco Gattorno^1^

##### ^1^Second Division of Pediatrics, G. Gaslini Institute, Genova, Italy; ^2^Division of Human Genetics, G. Gaslini Institute, Genova, Italy; ^3^Division of Rheumathology, Bambino Gesù children hospital, Rome, Italy; ^4^Department of Pediatrics, Federico II Hospital, Napoli, Italy; ^5^Depatment of Pediatric rheumthology and nefrology, Policlinico di Messina, Messina, Italy, ^6^Department of Pediatrics, S. Maria delle Croci Hospital, Ravenna, Ravenna, Italy; ^7^Division of Rheumathology, Burlo Garofalo children hospital, Trieste, Italy; ^8^Department of Pediatrics, Regina Margherita Hospital, Torino, Italy; ^9^Department of Pediatrics, Policlinico di Messina, Messina, Italy; ^10^Department of cellular biochemistry, University of Genova, Genova, Italy; ^11^Scientific Director, G. Gaslini Institute, Genova, Italy

###### **Presenting author:** Roberta Caorsi

This abstract is not included here as it has already been published.

## Mechanisms of disease

### O3 Inhibition of interferon production driving tmem173-associated auto-inflammation

#### Marie-Louise Frémond^1^, Carolina Uggenti^1^, Lien Van Eyck^1^, Isabelle Melki^1^, Darragh Duffy^2^, Vincent Bondet^2^, Yoann Rose^1^, Bénédicte Neven^3^, Yanick Crow^1^, Mathieu P. Rodero^1^

##### ^1^Laboratory of Neurogenetics and Neuroinflammation, Imagine Institute, Paris, France; ^2^Center for Human Immunology, Institut Pasteur, Paris, France; ^3^Pediatric Hematology-Immunology and Rheumatology Department, Hôpital Necker, Paris, France

###### **Presenting author:** Marie-Louise Frémond

**Introduction:** Gain-of-function mutations in *TMEM173* encoding STING (Stimulator of Interferon Genes) underlie a novel type I interferonopathy, minimally responsive to conventional immunosuppressive therapies and associated with high childhood morbidity and mortality. A newly emerging treatment strategy in STING-related inflammation aims to control interferon (IFN) signalling post-binding of the IFN receptor, by targeting JAK1/2. We hypothesized that inhibition of IFN production itself might represent an alternative therapeutic approach in this disease.

**Objectives:** To evaluate the effect of BX795, a TBK1 inhibitor, on constitutive production of type I IFN in *TMEM173*-associated vasculopathy.

**Methods:** Human embryonic kidney 293 cells (HEKs) transfected with wild-type (WT) or mutant STING, and peripheral blood mononuclear cells (PBMCs) from three patients were treated with BX795 2 μM. The effect of BX795 on TBK1 signalling cascade was assessed in HEKs by western blotting and IFNβ reporter assay. In patient cells, IFNα production was assessed by ultra-sensitive digital ELISA, STAT1 phosphorylation status by flow cytometry and RNA expression of IFN-stimulated genes (ISG) by qPCR.

**Results:** In HEKs, BX795 inhibited the phosphorylation of IRF3 and IFNβ promoter activity induced by cGAMP or mutant STING. In patient-derived PBMCs, exposure to BX795 inhibited IFNα production measured by digital ELISA, and was associated with a decrease of STAT1 phosphorylation and reduced ISG transcription. In addition, BX795 decreased the positive feedback loop of STING on the type I IFN pathway.

**Conclusion:** Our findings demonstrate that inhibition of IFNα secretion is a potential approach to control disease-associated inflammation in patients with gain-of-function mutations in *TMEM173*, and may also be relevant in other monogenic type I interferonopathies.

**Disclosure of Interest**

None Declared

### O4 NLRP3 inflammasome activity in monocytes is regulated by 12/15-lipoxygenase

#### Yvonne Kusche^1^, Johannes Roth^1^, Katarzyna Barczyk-Kahlert^1^

##### ^1^Institute of Immunology, Münster, Germany

###### **Presenting author:** Yvonne Kusche

This abstract is not included here as it has already been published.

## Other autoinflammatory diseases

### O5 PLAS: a novel autoinflammatory and lymphoproliferative syndrome associated with PIM-1 mutations

#### Giovanna Ferrara^1^, Annalisa Chiocchetti^2^, Silvio Polizzi^3^, Josef Vuch^4^, Diego Vozzi^4^, Anna Mondino^4^, Erica Valencic^4^, Serena Pastore^4^, Andrea Taddio^4^, Flavio Faletra^4^, Umberto Dianzani^2^, Ugo Ramenghi^5^, Alberto Tommasini^4^

##### ^1^Department of Pediatrics, University of Trieste, Trieste, Italy; ^2^Amedeo Avogadro University of Eastern Piedmont, Novara, Italy; ^3^Ophthalmology Department, University of Florence, A.O.U.C., Florence, Italy; ^4^Department of Pediatrics, IRCCS Burlo Garofolo, Trieste, Italy; ^5^Department of Pediatric Sciences, University of Torino, Torino, Italy

###### **Presenting author:** Giovanna Ferrara

**Introduction:** We present a novel syndrome characterised by both lymphoproliferative and autoinflammatory features, due to a *PIM-1* kinase gene mutation (PIM-1 associated Lymphoproliferative Autoinflammatory Syndrome, PLAS).

**Objectives:** To describe clinical and genetic features in two cases with PLAS.

**Methods:** Whole exome sequencing (WES) analysis with trio based strategy in the first case and direct sequencing of candidate gene in the second one.

**Results:** A 35-year-old man was referred to ophthalmologist’s evaluation for blurry vision in his left eye. The fundus examination showed confluent, yellowish choroidal lesions in both eyes. During childhood, his past medical history was relevant for a diagnosis of celiac disease, recurrent episodes of protracted unexplained fever and skin rashes. He also referred unexplained inflammatory lesions of the osteoarticular and muscular system, one episode of aseptic meningitis, an intracranial granuloma with features of vascular proliferation and two episodes of anterior uveitis. In adulthood, his medical history included episodes of fever with urticaria and subcutaneous nodules. The histological examination of two skin lesions showed a reactive angioendotheliomatosis and a leukocytoclastic vasculitis, respectively. At the age of 30 he underwent splenectomy because of car accident and the subsequent biopsy revealed the incidental finding of non-caseating granulomas. Brain TC also founded multiple lytic and sclerotic skull lesions. He was diagnosed with atypical sarcoidosis (normal thorax X-ray, ACE) and started oral steroid and methotrexate.

Since childhood its laboratory data ever showed persistent elevated erythrocyte sedimentation rate (100–112 mm/h), strong positive C-reactive protein (60–80 mg/l) and polyclonal gammopathy (IgG 3600 mg/dl; IgM 126 mg/dl), elevated serum amyloid A levels (160 mg/ml) and decreased CD4/CD8 T cell ratio. Autoantibody (ANA, ENA, anti-DNA) and HLA B 27 were negative.

WES analysis showed the *de novo* heterozygous missense variation c.C1132A (p.H378N) in *PIM-1* gene (NM_001243186). This variation was never described in on-line database (HGMD, Exac, 1000 g, ESP6500).

The second patient was an adult female with a clinical history of persistent microcytic anemia, splenomegaly, striking hypergammaglobulinemia (IgG > 3000 mg/dL) and recurrent protracted fever episodes during childhood. At 13 years, she underwent splenectomy because of hypersplenism. At 28 years she presented a cerebral oligoastrocytoma that was removed by surgery. No mutations were found in genes associated with autoimmune lymphoproliferative syndromes and histiocytosis (*FAS*, *FASLG*, *XIAP*, *TNFRSF13B*, *UNC13D*, *CASP9*, *CASP10*).

The direct sequencing of PIM-1 revealed again the above described *de novo* mutation in PIM-1.

**Conclusion:** We propose that these patients represent two cases of a novel syndrome associated with a specific mutation in *PIM-1* gene (PLAS). First, the two cases share significant overlap of autoinflammatory and proliferative features involving the endothelial and the immune systems. Second, the gene mutation identified was never described in healthy people or in patients with other disorders and is predicted to be pathogenic based on all the bionformatic tools. Finally, previous functional studies showed that PIM-1 pathway is relevant to immune activation and indeed somatic mutations in this gene have been reported in association with lymphomas, and it is thought to contribute to survival of cancerous cells.

**Disclosure of Interest**

None Declared

### O6 Biallelic hypomorphic mutations in a linear deubiquitinase define otulipenia, an early-onset systemic autoinflammatory disease

#### Qing Zhou^1^, Xiaomin Yu^2^, Erkan Demirkaya^3^, Natalie Deuitch^1^, Deborah Stone^1^, Wanxia Tsai^4^, Amanda Ombrello^1^, Tina Romeo^1^, Elaine F. Remmers^1^, JaeJin Chae^1^, Massimo Gadina^4^, Steven Welch^5^, Seza Ozen^6^, Rezan Topaloglu^6^, Mario Abinun^7^, Daniel L. Kastner^1^, Ivona Aksentijevich^1^

##### ^1^Inflammatory Disease Section, National Human Genome Research Institute, Bethesda, USA; ^2^Genetics and Pathogenesis of Allergy Section, National Institute of Allergy and Infectious Diseases, Bethesda, USA; ^3^FMF Arthritis Vasculitis and Orphan disease Research Center (FAVOR), Gulhane Military Medical Academy, Ankara, Turkey; ^4^Translational Immunology Section, National Institute of Arthritis and Musculoskeletal Diseases, Bethesda, USA; ^5^Heart of England NHS Foundation Trust, Birmingham, United Kingdom; ^6^Department of Pediatric Nephrology and Rheumatology, Hacettepe University Faculty of Medicine, Ankara, Turkey; ^7^Institute of Cellular Medicine, Newcastle University, Newcastle, United Kingdom

###### **Presenting author:** Qing Zhou

**Introduction:** Autoinflammatory diseases are caused by mutations in genes regulating innate immune responses. More than 20 genes have been associated with various monogenic autoinflammatory disorders, however there is a significant number of undiagnosed patients including familial cases.

**Objectives:** To identify a disease-causing gene in two inbred families presenting with a neonatal-onset systemic inflammatory disease. We expected to find homozygous pathogenic mutations and subsequently to screen other undiagnosed patients who present with similar disease.

**Methods:** We performed whole-exome and candidate gene sequencing in the patients and their unaffected family members. NFκB luciferase assay and overexpression experiments in HEK 293 cells were used to confirm the causality of the mutations. Patient samples were analyzed using immunoprecipitation, immunoblotting, gene expression and cytokine profiling.

**Results:** We studied 3 unrelated patients, one of Pakistani and two of Turkish ancestry, with early-onset systemic inflammation and generalized erythematous rash diagnosed as neutrophilic dermatitis/panniculitis, lipodystrophy, failure to thrive, and without obvious primary immunodeficiency.

We identified three novel homozygous mutations in the *FAM105B* gene, which encodes OTULIN, the deubiquitinase that specifically hydrolyzes Met1-linked ubiquitin chains (Ub) from target proteins. Post-translational modifications by ubiquitination are important for the regulation of many signaling complexes including the NF-kB pathway. Met1-Ub chains are generated by the linear ubiquitin assembly complex (LUBAC). OTULIN is an evolutionarily highly conserved protein and in mice complete deficiency is embryonically lethal.

The p.Leu272Pro and p.Tyr244Cys mutations are located near the linear ubiquitination binding region, while the p.Gly174Aspfs*2 mutation truncates the protein. Cells transfected with mutated versus wildtype OTULIN had decreased enzyme activity and showed a substantial defect in the linear deubiquitination of target molecules NEMO, RIPK1, TNFR1 and ASC. Stimulated patients’ cells, fibroblasts and PBMCs, showed increased phosphorylation of IKKα/β, IκBα, JNK, P38 and a higher linear-ubiquitinaton level of the target proteins. These results indicate that inefficient deubiquitination of OTULIN target proteins might explain increased NF-κB activity in mutant cells. Levels of many proinflammatory cytokines including IL-1β, TNF, IL-6, IL-18, IL-12, and IFN-γ were substantially increased in patient serum samples and stimulated cells. Cytokine profiling in serum samples was consistent with disease activity. Transcriptome profiling of patient whole blood samples and stimulated fibroblasts showed similar results. One patient had excellent response to treatment with the TNF inhibitor, infliximab. Two other patients have been treated with either etanercept or anakinra, but they still have an active disease requiring steroids.

**Conclusion:** Loss-of-function mutations in OTULIN result in increased linear ubiquitination of signaling molecules and lead to enhanced TNFR1, NF-κB, and ASC-dependent inflammasome activity. This new disease, which we named otulipenia, adds to the emerging spectrum of human diseases caused by defects in the ubiquitin pathway and suggests a role for targeted cytokine therapies.

**Disclosure of Interest**

None Declared

## Diagnostic challenges

### O7 Discrimination among monogenic and acquired disease with inflammatory and autoimmune component through the in vitro study of signal transducers of type I interferon

#### Donatella Vairo^1^, Rosalba Monica Ferraro^1^, Giulia Zani^2^, Jessica Galli^3^, Micaela De Simone^3^, Marco Cattalini^4^, Elisa Fazzi^3^, Silvia Giliani^1^

##### ^1^Translational Molecular Medicine Department, University of Brescia, Brescia, Italy; ^2^Clinical and Expreimental Sciences, Pediatric Clinic, Brescia, Italy; ^3^Unit of Child Neurology and Psychiatry, Spedali Civili, Brescia, Italy; ^4^Clinical and Experimental Sciences, University of Brescia, Brescia, Italy

###### **Presenting author:** Donatella Vairo

This abstract is not included here as it has already been published.

### O8 A targeted next-generation sequencing gene panel for autoinflammation

#### Ebun Omoyinmi^1^, Ariane Standing^1^, Dorota Rowczenio^2^, Annette Keylock^1^, Sonia Melo Gomes^1^, Fiona Price-Kuehne^1^, Sira Nanthapisal^1^, Claire Murphy^1^, Thomas Cullup^3^, Lucy Jenkins^3^, Kimberly Gilmour^4^, Despina Eleftheriou^1^, Helen Lachmann^2^, Philip Hawkins^2^, Nigel Klein^1^, Paul Brogan^1^

##### ^1^IIIP, UCL, Institute of Child Health, London, United Kingdom; ^2^NAC, Royal Free Hospital, London, United Kingdom; ^3^NE Thames Genetics Laboratory, London, United Kingdom; ^4^Immunology, GOSH, London, United Kingdom

###### **Presenting author:** Ebun Omoyinmi

**Introduction:** Monogenic autoinflammatory diseases (AID) are severe lifelong systemic inflammatory disorders with dysregulated innate immunity, causing significant morbidity, mortality, and economic burden. The number of AID is rapidly expanding. Securing a molecular diagnosis is of major importance for treatment, prognosis, and genetic counselling. Routine genetic screening is time-consuming, costly, and lacks sensitivity since only common disease harbouring exons of a minority of the known AID genes are currently tested using Sanger sequencing. Next-generation sequencing (NGS) offers the ability to rapidly and cost-effectively screen all exons of gene panels containing hundreds of genes. This approach has not yet been routinely introduced in the UK for AID.

**Objectives:** To develop and evaluate the performance of a NGS gene panel for AID.

**Methods:** The Agilent SureDesign tool was used to design an NGS panel targeting 113 genes, grouped into 9 broad clinical phenotypes: AID; monogenic vasculitis/vasculopathy; complement defects; monogenic lupus; hemophagocytic lymphohistiocytosis (HLH); early-onset inflammatory bowel disease; autoimmune lymphoproliferative syndromes; monogenic stroke; and hereditary amyloidosis. The targeted region includes coding exons, conserved non-coding exons, upstream promoter regions, and splice sites. Captured and indexed libraries (QXT Target Enrichment System) were sequenced as a multiplex of 16 samples on an Illumina MiSeq sequencer in paired-end mode. Positive controls for panel validation comprised 21 DNA samples from patients with confirmed mutations in a variety of genes on the panel. We then applied the panel to test 27 prospective samples with suspected but unconfirmed monogenic inflammation. Read alignment, variant calling, and annotation were performed using 2 pipelines; the Galaxy online bioinformatics tool (https://galaxyproject.org/) and Agilent SureCall v3.0 software.

**Results:** In the validation stage, our targeted panel detected all known mutations in the 19 control samples with an average read depth of 244X (+/- 29X) across the captured genes. The panel was effective at detecting different types of variants, including rare and common single nucleotide variants (SNVs), insertion/deletions, splice-junction variants, upstream promoter region variants, and somatic mosaicism. The Agilent SureCall software was more sensitive and able to call mosaic mutation with allele frequency as low as 3%. Prospective testing of the panel in 27 patients revealed potential pathogenic variants in 12 of the 27 patients giving a detection rate of 44%. These included atypical patients with novel mutations in specific exons/genes, not currently routinely available for testing by Sanger sequencing in the NHS.

**Conclusion:** This study demonstrates the clinical utility of a comprehensive NGS-based targeted gene panel which was both highly sensitive and specific in detecting different sequence variants of clinical significance. We are currently integrating this panel into a routine diagnostic laboratory testing strategy for monogenic inflammation. This will facilitate accurate and timely molecular diagnoses, more targeted treatment, and ultimately better outcomes for our patients.

**Disclosure of Interest**

None Declared

## Vasculitis

### O9 Optimal aspirin dose in Kawasaki disease - the ongoing battle

#### Anita Dhanrajani^1^, Mercedes Chan^2^, Stephanie Pau^3^, Janet Ellsworth^2^, Jaime Guzman^1^

##### ^1^Pediatric Rheumatology, BC Children’s Hospital, University of British Columbia, Vancouver, Canada; ^2^Pediatric Rheumatology, Stollery Children’s Hospital, University of Alberta, Edmonton, Canada; ^3^Pediatrics, Stollery Children’s Hospital, Edmonton, Canada

###### **Presenting author:** Anita Dhanrajani

**Introduction:** Kawasaki disease (KD) is an acute systemic vasculitis of childhood with potential cardiac morbidity in the form of coronary aneurysms. Pediatric Rheumatologists play a key role in the diagnosis and management of difficult cases of KD. Guidelines for KD have been published by American Heart Association (AHA). Standard use of 2 g/kg intravenous immunoglobulin (IVIG) is well supported by randomized trials, however, there is controversy about the best dose of acetyl salicylic acid (ASA). Consequently, there is worldwide difference in the current ASA dose used. Concerns regarding gastrointestinal side effects of ASA have prompted use of moderate doses (30-50 mg/kg) in some parts of the world, supported by evidence from meta analyses. The use of high dose ASA (80-100 mg/kg/day) in the initial phase of KD, at one Canadian Centre, as opposed to low dose ASA (3-5 mg/kg) at another centre with a similar patient population, led to this retrospective study

**Objectives:** To determine if low dose ASA in initial phases of KD at one Canadian centre leads to worse short term outcomes compared to higher doses of aspirin used at another centre. Primary Outcome: IVIG resistance Secondary outcomes:1. Duration of hospital stay 2. Incidence of coronary artery abnormalities

**Methods:** Retrospective chart review of patients admitted with a diagnosis of Kawasaki disease at 2 Canadian centres. 150 charts from centre 1 and 178 charts from centre 2 were reviewed. Inclusion criteria:1. Definitive diagnosis of KD as per AHA guidelines2. Received IVIG at AHA recommended dose 3. Received ASA (3–5 mg/kg at centre 1 and 80–100 mg/kg in initial phase at centre 2)Charts with incomplete information were excluded. Data from both centres was entered into the REDCAP database and exported to SPSS version 22.0 for analysis. Data analysis: Baseline characteristics were compared with descriptive statistics. Chi square test was used to compute p values for the primary outcome. Multiple logistic regression analysis was used to adjust for potential confounders. Duration of hospital stay was compared with ANOVA to adjust for confounders.p value of <0.05 was considered significant.

**Results:** 122/150 and 127/178 patients from centre 1 and 2 respectively were included for the final analysis. There were no significant differences in baseline characteristics between the two groups. IVIG resistance was seen in 28/122 patients in Centre 1 vs 11/127 in centre 2 (p = 0.030). There was no significant difference in the duration of hospital stay between the two groups (p: 0.375) The differences in coronary artery abnormalities between the two groups did not reach a significant p value after adjusting for confounders (p = 0.075).

**Conclusion:** In this retrospective study, a lower dose of ASA led to an increased risk of IVIG resistance without a significant difference in coronary artery outcomes. The 154% increase in the use of IVIG translates to potential increase in health care costs. Given that IVIG is a scarce resource worldwide, this justifies conducting larger multi centric trials to explore cost benefit ratio of high versus low dose aspirin.

**Disclosure of Interest**

None Declared

**Table 2 (abstract O9).** See text for description

	Unadjusted				Adjusted			
Variable names	ODDS RATIO	95% CI lower	95% CI upper	P -value	ODDS RATIO	95% CI lower	95% CI upper	P- value
Aspirin dose (HIGH vs LOW)	0.318	0.151	0.673	0.003	0.313	0.110	0.892	0.030
Platelet count <300000 IN WEEK 1	1.585	0.889	2.827	0.119	1.734	0.889	3.384	0.107
CRP value	1.852	0.305	11.258	0.503	1.003	0.998	1.008	0.267
Age categories (<=12 MONTHS, >12 MONTHS)	1.138	0.444	2.916	0.788	1.096	0.379	3.173	0.865
Sodium < 133 mmol/L	1.368	0.711	2.631	0.348	1.752	0.842	3.646	0.134

### O10 Childhood Takayasu Arteritis: disease course and response to therapy

#### Florence A. Aeschlimann^1^, Marinka Twilt^2^, Simon W. Eng^1^, Shehla Sheikh^1^, Ronald M. Laxer^1^, Diane Hebert^3^, Damien Noone^3^, Christian Pagnoux^4^, Susanne M. Benseler^2^, Rae S. Yeung^1^

##### ^1^Rheumatology, The Hospital for Sick Children, Toronto, Canada; ^2^Rheumatology, Alberta Children’s Hospital, Calgary, Canada; ^3^Nephrology, The Hospital for Sick Children, Toronto, Canada; ^4^Rheumatology, Mount Sinai Hospital, Toronto, Canada

###### **Presenting author:** Susanne M. Benseler

**Introduction:** Takayasu Arteritis (TA) is a devastating large vessel vasculitis commonly associated with systemic inflammation and resulting in severe ischemic end organ dysfunction. To date, treatment efficacy, disease course and flare risk in children with TA are widely unknown.

**Objectives:** To describe clinical presentation, treatment regimens and outcome of childhood TA and to identify risk factors for adverse outcome.

**Methods:** A single-center cohort study of consecutive children fulfilling the EULAR/PRINTO/PReS criteria for childhood TA between 1986 and 2015 was performed. Clinical, laboratory and imaging features, treatment, disease course and outcome were documented. Disease activity was assessed by the Pediatric Vasculitis Disease Activity Score (PVAS), damage by the Pediatric Vasculitis Damage Index (PVDI). Active disease was defined as PVAS ≥ 1 and/or increased inflammatory markers not explained by other causes and/or active disease on imaging (new lesions and/or evidence of vessel wall inflammation). Inactive disease was defined as PVAS = 0 and normal inflammatory markers or inactive disease on imaging. Adverse outcome was defined as disease flare (new TA symptoms and/or increased inflammatory markers necessitating therapy escalation and/or new angiographic lesions) or death. Analyses: Kaplan-Meier statistics compared treatment regimens. Logistic regression analyses tested putative predictors for adverse outcome.

**Results:** Twenty-seven children (74% female) were included; median age at diagnosis was 12.4 years (range 4.2–17.7); median follow-up duration was 2.1 years. The most common presenting symptoms were malaise (48%), headaches (33%), weight loss (30%) and claudication of the extremities (22%), the most common clinical findings blood pressure discrepancy between limbs (67%), decreased/absent pulse (59%), hypertension and bruits (56%). Median ESR was 35 mm/h (range 1–109), CRP 31.9 mg/dl (range 0.5–140). Twenty-two (81%) children presented with active disease at diagnosis and received immunosuppressive treatment (Table 3). Adverse outcome was seen in 14/27 (52%) children: 13 (48%) experienced flares after a median of 9.2 months after diagnosis and 2 (7%) died within months of diagnosis. The two-year flare-free survival was 80% with biologic therapies compared to 43% in conventional therapies (p = 0.03). At last follow-up, biologic therapies resulted in significantly higher rates of inactive disease (p = 0.02). None of the other tested variables were associated with an increased flare risk.

**Conclusion:** Childhood TA carries a dramatic burden: half of the children experienced flares and 7% died. Biologic treatment regimens were associated with significantly better control of disease activity.

**Disclosure of Interest**

None Declared

**Table 3 (abstract O10).** Treatment regimens and adverse outcome in the childhood TA cohort

Induction therapy	No. of patients (Total N = 27)	Median PVAS at diagnosis (range)	Inactive disease at 6 months	Therapy for maintenance of remission/ during disease course	No. of patients with flares	Death	Inactive disease at last follow up
CS only	4 (15)	16 (8–17)	1/3	CS, Cyclo, Aza, MMF, MTX	3/4	1/4	1/3
CS + MTX	10 (37)	15.5 (6–21)	2/10	CS, Cyclo, Aza, Lef, MTX, Biologics	7/10	0/10	5/10
CS + Cyclo	5 (19)	7 (6–35)	2/4	CS, Cyclo, Aza, Lef, MTX, Biologic	3/5	1/5	3/4
CS + MTX + Biologic	3 (11)	10 (6–25)	3/3	CS, Biologic + Lef/MTX	0/3	0/3	3/3
No treatment	5 (19)	10 (6–17)	5/5	None	0/5	0/5	5/5

*Aza* azathioprine, *CS* corticosteroids, *Cyclo* cyclophosphamide, *Lef* leflunomide, *MTX* methotrexate, *MMF* mycophenolat mofetil

## Immune aspects of JIA

### O11 Dysbalanced serum factor environments can drive IL-17 over-expression by gamma delta T cells in systemic juvenile idiopathic arthritis

#### Christoph Kessel^1^, Katrin Lippitz^1^, Toni Weinhage^1^, Claas Hinze^1^, Helmut Wittkowski^1^, Dirk Holzinger^1^, Niklas Grün^1^, Dirk Föll^1^

##### ^1^Pediatric Rheumatology & Immunology, University Childrens’ Hospital, Münster, Germany

###### **Presenting author:** Christoph Kessel

**Introduction:** Systemic-onset juvenile idiopathic arthritis (sJIA) is considered to follow a biphasic course with an initial systemic disease phase driven by innate immune mechanisms and interleukin (IL)-1β as a key cytokine, while a second chronic arthritic phase may be dominated by adaptive immunity and cytokines such as IL-17A.

**Objectives:** A recent sJIA mouse model points to a critical implication of IL-17A expressing γδT cells (Tγδ17) in disease pathology [1]. In this model, IL-17 expressing γδT cells drive a phenotype that resembles arthritis-prominent sJIA in an environment of low interferon (IFN)γ. However, human data on the role of IL-17 expressing γδT cells in sJIA are lacking.

**Methods:** Paired serum samples of sJIA patients (n = 12) during active and inactive disease as well as sera from healthy children (n = 10) were analyzed for expression of IL-1β, IL-6, IL-17A, IL-23, IFNγ and S100A12 by bead array assay. *Ex vivo* T cellular cytokine expression was assayed in whole peripheral blood samples collected from active and inactive sJIA (n = 12) as well as healthy children (HC, n = 13). Cells in whole peripheral blood were stimulated with phorbol 12-myristate 13-acetate (PMA) and ionomycin and were stained for expression of CD3, CD4, γδTCR, IL-17A and IFNγ before analysis by flow cytometry. Finally, CD4^+^ and γδTCR^+^ T cells were isolated from sJIA patients’ as well as healthy control PBMCs (both n = 5) and co-cultured with healthy control monocytes under different stimulating conditions. IL-17A and IFNγ expression was quantified.

**Results:** Particularly in active sJIA patients’ serum IL-17A expression was significantly increased. *Ex vivo* T cellular cytokine expression analysis revealed sJIA γδT cells to over-express IL-17A compared to cells in whole blood obtained from healthy donors. This was not observed with CD4^+^ T cells. During active disease peripheral Tγδ17 cell frequencies were decreased and appeared to recover in patients with no clinical disease activity. IL-17A over-expression by sJIA γδT cells was confirmed in *in vitro* studies on isolated cell level and found to occur particularily upon stimulation of monocyte:γδT cell co-cultures with TLR4 ligands such as LPS or S100A12. On the contrary to γδT cellular IL-17A over-expression, IFNγ expression by sJIA CD3^+^CD4^+^ cells was strikingly low, both in *ex vivo* analysis of patients’ whole blood as well as *in vitro* T cell cultures. Finally, in our assays particularly therapeutic IL-1 blockade appeared to expand peripheral IFNγ^+^CD4^+^ cells and restore intracellular IFNγ levels, while γδT cellular IL-17A expression and release normalized to that of healthy donors.

**Conclusion:** An sJIA serum factor environment with elevated levels of endogenous TLR4 ligands such as S100 proteins and increased expression of cytokines like IL-1β can result in IL-17 over-expression by sJIA γδT cells. As already suggested from the sJIA mouse model, this may be promoted by impaired IFNγ expression as we observed with CD4^+^ T cells. During active disease Tγδ17 cells may migrate from the periphery to inflamed tissues to drive local inflammation and promote downstream differentiation and expansion of Th17 cells. As a cell-type bridging innate and adaptive immunity, γδT cells may thus play a key pathological role in tilting systemic to arthritis-prone disease in men and mice.

**References**

1. Avau A et al. Systemic juvenile idiopathic arthritis-like syndrome in mice following stimulation of the immune system with freund’s complete adjuvant: Regulation by interferon-γ. Arthritis and Rheumatology. 2014; 66(5):1340–1351.

**Disclosure of Interest**

None Declared

### O12 The composition of the gut microbiota differs between children with JIA and healthy controls

#### Pieter Van Dijkhuizen^1,2^, Federica Del Chierico^3^, Clara Malattia^1,4^, Alessandra Russo^3^, Denise Pires Marafon^5^, Nienke M. ter Haar^2^, Silvia Magni-Manzoni^5^, Sebastiaan J. Vastert^2^, Bruno Dallapiccola, Berent Prakken^2^, Alberto Martini^1,4^, Fabrizio De Benedetti^5^, Lorenza Putignani^3^

##### ^1^Paediatric rheumatology, Istituto Giannina Gaslini, Genoa, Italy; ^2^Paediatric rheumatology, University Medical Centre Utrecht, Wilhelmina Children’s Hospital, Utrecht, Netherlands; ^3^Human Microbiome Unit, Ospedale Pediatrico Bambino Gesù, Rome, Italy; ^4^Università degli Studi di Genova, Genoa, Italy; ^5^Paediatric rheumatology, Ospedale Pediatrico Bambino Gesù, Rome, Italy; ^6^Scientific direction, Ospedale Pediatrico Bambino Gesù, Rome, Italy

###### **Presenting author:** Pieter Van Dijkhuizen

**Introduction:** The human body is home to a myriad of non-pathogenic bacteria, the so-called microbiota. It has been hypothesised that the gut microbiota plays a role in the pathogenesis of autoimmune diseases, such as RA. Its role in the pathogenesis of JIA needs further investigation.

**Objectives:** To study differences in the composition of the gut microbiota in patients with JIA compared to healthy controls.

**Methods:** A prospective follow up study was initiated in October 2013, in which untreated patients were enrolled with a new onset of JIA according to ILAR criteria and a duration of symptoms of at most 6 months. Patients were followed for 2 years. At baseline, and if ever a patient presented with inactive disease according to Wallace criteria or a disease flare, a faecal sample was collected and stored at -80 °C. Operational taxonomic units were pyrosequenced targeting 16S ribosomal RNA. Additionally, faecal samples were collected from age-, gender- and geography-matched healthy controls and processed as above.

The microbial composition of the faecal samples was analysed using compositional data analysis. The relative abundance was visualised using ternary plots. Differences between healthy controls and patients, as well as between Italian and Dutch patients were tested using MANOVA. Paired baseline, inactive disease and flare samples were analysed using Hotelling’s T^2^ paired sample test. Association with various clinical parameters was tested using MANOVA. Finally, a random forest algorithm was used to distinguish Italian patients and healthy controls based on their microbiota composition.

**Results:** Overall, 307 samples were available for analysis. Of these, 100 belonged to patients at baseline (72 Italian samples and 28 Dutch samples). These were matched 1:1 with 107 healthy control samples. Moreover, 100 samples were obtained during follow-up: 70 in inactive disease and 30 in persistent activity or disease flare.

There was strong evidence the composition of the microbiota at the phyla level was different in patients at baseline with respect to healthy controls, when analysing Italian and Dutch patients both separately and jointly, taking into account the different age at onset of Italian and Dutch patients (p ≤ 0.003). There was no evidence of differences between patients at baseline and in inactive disease, or between patients at baseline and in persistent activity (p ^3^ 0.048). There was evidence that the composition of the microbiota at the phyla level was different in ANA positive patients with respect to ANA negative patients, even when taking the nationality of patients into account (p = 0.02). There was no evidence for an association with uveitis at baseline (p = 0.18) and only weak evidence for an association with the number of active joints (p = 0.07).

Visualisation of the results, as well as compositional Principal Components Analysis showed that the relative abundance of Proteobacteria and Tenericutes contributed the most to the differences between the groups in all comparisons.

The random forest algorithm separated patients and healthy controls well, using only the microbiota composition at the species level. The AUC of the model was 0.99 (95% CI 0.98- > 0.99).

**Conclusion:** This study provides strong evidence for differences in the composition of the microbiota, and especially the relative abundance of Proteobacteria and Tenericutes, between patients with JIA and healthy controls. Future research should be directed to elucidate the effects of the microbiota on JIA.

**Disclosure of Interest**

None Declared

## Vasculitis and Behcet disease

### O13 A new approach to assessment of juvenile vasculitis: the juvenile vasculitis multidimensional assessment report (J-VAMAR)

#### Berna Eren Fidanci^1^, Kenan Barut^2^, Serap Arıcı^3^, Dogan Simsek^1^, Mustafa Cakan^4^, Ezgi D. Batu^3^, Sezgin Şahin^2^, Ayşenur Kısaarslan^5^, Ebru Yilmaz^6^, Özge Basaran^7^, Ferhat Demir^8^, Kubra Ozturk^9^, Zübeyde Gunduz^5^, Betül Sozeri^5^, Balahan Makay^10^, Nuray Ayaz^4^, Onder Yavascan^6^, Ozlem Aydog^11^, Yelda Bilginer^3^, Zelal Ekinci^9^, Dilek Yıldız^1^, Faysal Gök^1^, Muferret Erguven^12^, Erbil Unsal^10^, Ozgur Kasapcopur^2^, Seza Ozen^3^, Erkan Demirkaya^1^, and For the FMF Arthritis Vasculitis and Orphan Disease Research in Paediatric Rheumatology (FAVOR)

##### ^1^Gulhane Military Medical Academy, Ankara, Turkey; ^2^Istanbul University, Istanbul, Turkey; ^3^Hacettepe University, Ankara, Turkey; ^4^Kanuni Sultan Suleyman Research Hospital, Istanbul, Turkey; ^5^Erciyes University, Kayseri, Turkey; ^6^Tepecik Training Hospital, Izmir, Turkey; ^7^Dıskapı Training Hospital, Ankara, Turkey; ^8^Karadeniz Technical University, Trabzon, Turkey; ^9^Kocaeli University, Kocaeli, Turkey; ^10^9 Eylul University, Izmir, Turkey; ^11^Sami Ulus Children Hospital, Ankara, Tukey; ^12^Goztepe Training Hospital, Istanbul, Turkey

###### **Presenting author:** Berna Eren Fidanci

**Introduction:** There are many effects of vasculitis (fatigue, reduced energy level, school absenteeism, physical appearance) that were quite important to patients but not measured with the outcome instruments currently included in clinical trials of vasculitis.

**Objectives:** To develop and test a new multidimensional questionnaire for assessment of children with juvenile vasculitis in standard clinical care.

**Methods:** The Juvenile VAsculitis Multidimensional Assessment Report (J-VAMAR) includes 15 parent or patient-centered measures or items that assess well-being, pain, functional status, health-related quality of life, problems about school, disease activity, disease status and course, symptoms of vasculitis, use of medications, side effects of medications, therapeutic compliance. The J-VAMAR is proposed for use as both a proxy-report and a patient self-report, with the suggested age range of 7-18 years for use as a self-report. The Outcome Measures in Rheumatology Clinical Trials (OMERACT) filter for outcome measures in rheumatology was applied to validate the J-VAMAR.

**Results:** The analysis data set was collected between 2013 to 2016, the questionnaire was completed by 164 children with vasculitis in 429 visits. The diagnoses of the patients were as follows: 46.6% HSP, 19% Behcet vasculitis, 9.2% CPAN, 9.2% Takayasu, 6.6% Kawasaki, 5.5% Wegener, 3.2% Unclassified and 0.5% Churg-Strauss. The mean disease onset age of children with vasculitis was 8,36 ± 4,61. The J-VAMAR was found to be feasible and to possess face,content, criterion and construct validity. All parents and children reported that the questionnaire was simple and easy to understand. Completion and scoring appeared to be quick, requiring less than15 minutes. The Cronbach’s alpha coefficient for internal consistency for the J-VAMAR dimensions was between 0.735–0.963. Between the test-retest scale scores, there is a significant and a positive correlation (0.326–1.000). When the criterion validity is considered, we would say that the correlation level between the each subscale and the related scale spanned from medium (r = 0.282, p < 0.05) to large (r =0.694, p < 0.001). Parents’ proxy-reported and children’s self-reported data were remarkably concordant.

**Conclusion:** The J-VAMAR provided thorough information for the study patients about recent medical history and current health status. It performed similarly across different children’s ages and characterized the level of disease activity and disability well. The development of the J-VAMAR introduces a new and a multi-dimensional approach in pediatric rheumatology practice. This new questionnaire is a valid tool may help enhance the quality of care of children with vasculitis.

**Disclosure of Interest**

None Declared

### O14 Behçet’s disease in children: Eastern Mediterranean experience

#### Hafize E. Sönmez^1^, Ezgi D. Batu^1^, Betül Sözeri^2^, Yonatan Butbul^3^, Yelda Bilginer^1^, Seza Özen^1^

##### ^1^Division of Rheumatology, Hacettepe University Faculty Of Medicine, Ankara, Turkey; ^2^Division of Rheumatology, Erciyes University Faculty of Medicine, Kayseri, Turkey; ^3^Division of Rheumatology, Rambam Medical Center, Haifa, Israel

###### **Presenting author:** Hafize E. Sönmez

**Introduction:** Behçet’s disease (BD) is a variable vessel vasculitis characterized by recurrent oral and/or genital aphthous ulcers accompanied by cutaneous, ocular, articular, vascular, gastrointestinal, and/or central nervous system inflammatory lesions. The most widely used diagnostic criteria for adult onset disease is the International Behçet’s Study Group (ISG) critera. An international expert consensus group (the pediatric BD [PEDBD] group) has recently proposed a new set of criteria for the classification of Behçet’s disease (BD) in children. To evaluate the disease activity in the follow-up; there are mainly two severity scores Behçet’s disease dynamic activity measure (IBDDAM) and Behçet’s disease current activity form (BDCAF).

**Objectives:** Our aim was to test the performance of the PEDBD criteria compared to the ISG criteria. Our second aim was to check the correlation between the severity score systems and physician global assessment (PGA) in pediatric BD patients.

**Methods:** Two centers from Turkey and one center from Israel participated in this study. The disease onset was ≤16 years of age. As controls, pediatric patients with other diseases such as primary vasculitides, periodic fever syndrome, or systemic lupus erythematosus were included.

**Results:** Sixty eight BD (44.1% male) patients and 93 control patients were included. The median age at symptom onset was 132 (14–191) months and the median time to diagnosis from symptom onset was 12 (0–108) months. Acneiform lesions were significantly associated with female gender (18.4% in females versus 0% in males; p = 0.015). The sensitivity and specificity of the PEDBD and the ISG criteria and revised ICBD criteria were 73.5%/52.9% and 98.9%/100% respectively. Thirty two (47%) patients with BD failed to fulfill the ISG BD criteria. However, almost all of these patients met the PEDBD criteria. For systemic therapy, 62 (91.2%) patients received oral colchicine, 15 (22.1%) prednisolone, 16 (23.5%) azathioprine, 6 (8.8%) methotrexate, 5 (7.4%) anti-tumor necrosis factor alpha treatment, and one patient received cyclophosphamide. The median (min-max) IBDDAM and BDCAF scores at diagnosis were 6 (1–23) and 4 (1–7) and significantly decreased to 1 (0–8) and 1 (0–4) respectively at latest follow-up (p < 0.001 for both). The median (min-max) physician global assessment score at diagnosis was 5 (2–9) and significantly decreased to 1 (0–7) at latest follow-up (p < 0.001). IBDDAM score had a strong positive correlation with BDCAF score (r = 0.637; p < 0.001). PGA positively correlated with BDCAF and IBDDAM scores (r = 0.502; p < 0.001 and r = 0.624; p < 0.001, respectively).

**Conclusion:** In our pediatric series, the PEDBD criteria showed better sensitivity (73.5%) than the ISG criteria and the specificity was close. The higher sensitivity of the PEDBD criteria set is a big advantage for pediatric patients since early diagnosis and timely treatment is very important. Currently, BD patients are treated according to the physician’s judgement of disease activity. Our study demonstrated that the severity scores were positively correlated with each other and PGA and thus may be used in clinical practice.

**Disclosure of Interest**

None Declared

## Macrophage activation syndrome

### O15 Immune abnormalities leading to exaggerated production of IFN-gamma and the therapeutic response to an anti-IFN-gamma antibody in a patient with NLRC4 mediated disease

#### Claudia Bracaglia^1#^, Giusi Prencipe^1#^, Manuela Pardeo^1^, Geneviève Lapeyre^2^, Emiliano Marasco^1^, Antonella Insalaco^1^, Walter Ferlin^2^, Robert Nelson^2^, Cristina de Min^2^, Fabrizio De Benedetti^1^

##### ^1^Division of Rheumatology, Ospedale Pediatrico Bambino Gesù IRCCS, Roma, Italy; ^2^Novimmune SA, Geneva, Switzerland

###### **Presenting author:** Claudia Bracaglia

^**#**^ **contributed equally to this study**

**Introduction:** Animal and human data suggest that IFNγ plays a pathogenic role in HLH. A phase 2 trial with the anti-IFNγ monoclonal antibody NI-0501 in primary HLH provides encouraging preliminary data [1]. Gain-of-function mutations in *NLRC4* are associated with a distinct autoinflammatory syndrome, with recurrent HLH, fever episodes, and enterocolitis [2].

**Objectives:** To report a patient with severe early onset progressive HLH carrying a *de novo* missense mutation in *NLRC4* (T337N), the immune abnormalities leading to abnormal production of IFNγ and the response to treatment with NI-0501.

**Methods:** We evaluated cytokine levels by multiplex assay and by specific ELISAs and expression of IFNγ in freshly isolated PBMCs by cytometry.

**Results:** LR, caucasian male, presented, at 20 days of age, fever and rash and progressively developed clinical and laboratory features of HLH leading rapidly to liver failure and subsequent multiorgan failure. Genes causing primary-HLH and functional tests were negative. High-dose glucocorticoids and cyclosporine-A led to partial improvement. Development of sepsis triggered HLH reactivation. Measurable IFNγ levels (6 pg/ml) and high levels of the IFNγ inducible chemokines CXCL9 (5670 pg/ml) and CXCL10 (4400 pg/ml) were found, the latter demonstrating activation of the IFNγ pathway. NI-0501 was started (compassionate use) on background of dexamethasone (13.6 mg/m2) and cyclosporine-A. After 3 months, the child was discharged in excellent conditions (prednisone 0.3 mg/kg). Markedly elevated production of IFNγ was revealed by the administration of NI-0501 through measurement of total IFNγ bound to circulating NI-0501. This IFNγ was fully neutralized, as shown by rapidly undetectable levels of CXCL9 and CXCL10. The percentage of CD4+, CD8+ and CD56+ cells expressing IFNγ was significantly increased in the NLRC4 patient without (0.95%, 0.88%, 0.80%, respectively) and with stimulation with PMA/ionomycin (6.44%, 10.20%, 25,00%). Serum levels of IL-18 were markedly higher (as expected in NLRC4-mediated disease) at >300 ng/ml before treatment and remained elevated throughout treatment (ranging between 25 and 35 ng/ml), with the patient showing no symptoms. Incubation of PBMCs from healthy controls with NLRC4 patient’s sera in the presence of IL-12 (known costimulus for IFNγ synthesis) led to a marked hyperproduction of IFNγ (>20 ng/ml), compared to sera from patients with other autoinflammatory diseases (0.59 ± 0.47 ng/ml). This increase in IFNγ production was neutralized by incubation with an anti-IL18 receptor antibody. After 7 months of NI-0501 treatment, all therapies, including NI-0501, were discontinued, without any clinical or laboratory sign of HLH reactivation, even in the presence of occasional increases in IL-18 levels.

**Conclusion:** Our data suggest that in NLRC4-related disease overproduction of IL-18, induced by an hyperfunctional NLRC4 inflammasome, may be one of the contributors to the up-regulation of IFNγ production that appears to be driving HLH. In this patient, neutralization of IFNγ allowed control of all disease features, enabling withdrawal of all treatments, including glucocorticoid and cyclosporyn-A. No safety concern emerged.

**References**

1. Jordan ML. A novel targeted approach to the treatment of HLH with an anti-IFNγ-monoclonal antibody: first results from a pilot phase 2 study in children with primary HLH. 2015 American Society of Hematology.

2. Canna SW. An activating NLRC4 inflammasome mutation causes autoinflammation with recurrent macrophage activation syndrome. Nat Genet 2014

**Disclosure of Interest**

C. Bracaglia: None Declared, G. Prencipe: None Declared, M. Pardeo: None Declared, G. Lapeyre Employee of: Novimmune, E. Marasco: None Declared, A. Insalaco: None Declared, W. Ferlin Employee of: Novimmune, R. Nelson Employee of: Novimmune, C. de Min Employee of: Novimmune, F. De Benedetti Grant / Research Support from: Novartis, Novimmune, Hoffmann- La Roche, SOBI, AbbVie, Pfizer

### O16 Long-term efficacy and safety of Canakinumab in patients with active systemic juvenile idiopathic arthritis (SJIA): results from a phase III extension study

#### N. Ruperto^1^, H.I. Brunner^2^, P. Quartier^3^, T. Constantin^1^, E. Alexeeva^1^, I. Kone-Paut^1^, K. Marzan^2^, N. Wulffraat^1^, R. Schneider^2^, S. Padeh^1^, V. Chasnyk^1^, C. Wouters^1^, J.B. Kuemmerle-Deschner^1^, T. Kallinich^1^, B. Lauwerys^4^, E. Haddad^2^, E. Nasonov^1^, M. Trachana^1^, O. Vougiouka^1^, K. Leon^5^, E. Vritzali^6^, K. Lheritier^6^, A. Martini^1^, D. Lovell^2^, and PRINTO/PRCSG

##### ^1^PRINTO-Istituto Gaslini, Genoa, Italy; ^2^PRCSG, Cincinnati, OH, USA; ^3^Necker-Enfants Malades Hospital, Paris, France; ^4^Cliniques Universitaires Saint-Luc and Université Catholique de Louvain, Brussels, Belgium; ^5^Novartis Pharmaceuticals Corporation, East Hanover, NJ, USA; ^6^Novartis Pharma AG, Basel, Switzerland

###### **Presenting author:** N. Ruperto

**Introduction:** The management of SJIA with biological therapies is aimed to achieve and maintain clinical remission (CR), and accordingly taper corticosteroids (CS). Canakinumab (CAN) demonstrated high inactive disease (ID) rates in about 33% of patients (pts) in Day 15 and 30 respectively in previous studies [1]. However, little is known about high level response rates in SJIA pts using CAN long-term.

**Objectives:** To evaluate the long-term treatment response in terms of safety and efficacy in CAN treated pts with active SJIA.

**Methods:** This was an open-label, non-comparative study of CAN-naïve SJIA pts (≥2 to <20 years) receiving subcutaneous CAN 4 mg/kg every 4 weeks. Efficacy was assessed every 3 months by the aACR 30/50/70/90/100) responses compared to baseline (BL), ID or CR (ID for >6 months) and changes in JADAS10-CRP scores over time. Safety was assessed by adverse events (AEs) and serious AEs (SAEs). The results are based on the observed data with imputations to carry the last observation forward.

**Results:** Of 123 pts with active SJIA, 70 (57%) had fever and 71 (57.7%) used corticosteroids at BL. Mean CRP was 117.8 mg/L (normal: 0-10 mg/L), and, on average, pts had 9.9 active joints and 8.9 joints with limited motion. A rapid response was observed at Day 15: 59 (51%) and 27 (26%) pts had aACR ≥70 and aACR 100 responses, respectively. These responses were maintained at subsequent time points (Table). At Month 6, CR was achieved in 52 (42.3%) pts. Overall, 33 (26.8%) pts had CR for at least 12 months. At BL, the median CRP score was 22.3, with median changes from BL of −12.0 at Day 15 and −16.8 at last assessment, respectively. At the last assessment, 59 (48.4%) pts had ID (JADAS10 ≤ 1); 14 (11.5%) had low disease activity (JADAS10 > 1 and ≤3.8), while 14 (11.5%) had moderate and 35 (28.7%) had high disease activity. Overall, 24 (33.8%) pts were steroid-free at last assessment. In total, 108 (87.8%) pts had at least 1 AE. Overall, exposure adjusted AE and SAE rate was 8.22 and 54.8 events/pt-years (pyr) respectively, with 183.56 pyr exposure and 40 (32.5%) pts had SAEs; most commonly reported SAEs were disease flares or worsening of SJIA in 13 (10.6%) pts, macrophage activation syndrome in 6 (4.9%) pts, and fever in 4 (3.3%) pts. No deaths occurred in this study.

**Conclusion:** CAN treatment was associated with rapid response and sustained therapeutic effect over the long-term in the pts with active SJIA. The safety profile is consistent with other CAN studies.

**References**

1. Ruperto et al. N Engl J Med. 2012;367:2396–406.

**Trial registration identifying number:** NCT00891046

**Disclosure of Interest**

N. Ruperto Grant / Research Support from: Abbott, BMS, “Francesco Angelini”, GlaxoSmithKline (GSK), Hoffman-La Roche, Italfarmaco, Janssen, Novartis, Pfizer, Sanofi Aventis, Schwarz Biosciences, Sobi, Xoma and Wyeth, Speaker Bureau of: Abbott, AbbVie, Amgen, Biogenidec, Astellas, Alter, AstraZeneca, Boehringer, BMS, CDPharma, Celgene, CrescendoBio, EMD Serono, Hoffman-La Roche, Italfarmaco, Janssen, MedImmune, Medac, Novartis, Novo Nordisk, Pfizer, Sanofi Aventis, Servier, Takeda and Vertex, H. Brunner Consultant for: Novartis, Roche, Pfizer, UCB, Celgene, Regeneron, Amgen, Astrazeneca, GSK and BMS, Speaker Bureau of: Novartis and Roche, P. Quartier Grant / Research Support from: Abbvie, BMS, Chugai-Roche, Novartis, Pfizer, Consultant for: : Abbvie, Novartis, Servier and SOBI, Speaker Bureau of: Abbvie, BMS, Chugai-Roche, Medimmune, Novartis, Pfizer and SOBI, T. Constantin: None Declared, E. Alexeeva Grant / Research Support from: Roche, Abbott, Novartis, Pfizer, Bristol-Myers Squibb and Centocor, Speaker Bureau of: Roche, Novartis, Merck Sharp Dohme, Bristol-Myers Squibb, Medac and Pfizer, I. Kone-Paut Grant / Research Support from: SOBI, Novartis and Roche, Consultant for: Novartis, SOBI, Pfizer, Abbvie and Roche/Chugai, K. Marzan Grant / Research Support from: Novartis, Abbvie, N. Wulffraat Grant / Research Support from: Novartis, R. Schneider: None Declared, S. Padeh: None Declared, V. Chasnyk: None Declared, C. Wouters Grant / Research Support from: GSK, Novartis and Roche, J. Kuemmerle-Deschner Grant / Research Support from: Novartis, Consultant for: Novartis, SOBI and Baxalta, T. Kallinich Grant / Research Support from: Novartis, Speaker Bureau of: Sobi, Novartis, BMS and Roche, B. Lauwerys: None Declared, E. Haddad: None Declared, E. Nasonov: None Declared, M. Trachana Grant / Research Support from: Novartis, Abbvie, Bristol Meyers, Consultant for: Novartis, Roche and Pfizer, O. Vougiouka: None Declared, K. Leon Employee of: Novartis, E. Vritzali Employee of: Novartis, K. Lheritier Shareholder of: Novartis, Employee of: Novartis, A. Martini Grant / Research Support from: Abbott, BMS, “Francesco Angelini”, GlaxoSmithKline (GSK), Hoffman-La Roche, Italfarmaco, Janssen, Novartis, Pfizer, Sanofi Aventis, Schwarz Biosciences, Sobi, Xoma and Wyeth, Speaker Bureau of: Abbott, Abbvie, Amgen, Biogenidecm Bristol MyersSquibb, Astellas, Behringer, Italfarmaco, Janssen, MedImmune, Novartis, NovoNordisk, Pfizer, Sanofi, Roche, Servier and Takeda, D. Lovell Grant / Research Support from: National Institutes of Health, Consultant for: Astra-Zeneca, Bristol Meyers Squibb, AbbVee, Pfizer, Roche, Novartis, UCB, Forest Research Institute, Horizon, Johnson & Johnson, Biogen, Takeda, Genentech, Glaxo Smith Kline, Boehringer Ingelheim, Celgene and Jannsen, Speaker Bureau of: Genentech, Roche and Novartis

**Table 4 (abstract O16).** ACR responses achieved in the cohort by time point

Time point	CAN		
	N = 123		
	Minimum adapted ACR pediatric response	n (n/m%)	Patients with inactive disease (n/m)%
Month 12	m	85	(52/88) 59.1
	Non-Responders	4 (4.7)	
	aACR ≥30	81 (95.3)	
	aACR ≥50	77 (90.6)	
	aACR ≥70	73 (85.9)	
	aACR 100	49 (57.6)	
Month 21	m	65	(48/65) 73.8
	Non-Responders	3 (4.6)	
	aACR ≥30	62 (95.4)	
	aACR ≥50	58 (89.2)	
	aACR ≥70	54 (83.1)	
	aACR 100	39 (60.0)	
Last assessment	m	121	(62/122) 50.8
	Non-Responders	28 (23.1)	
	aACR ≥30	93 (76.9)	
	aACR ≥50	89 (73.6)	
	aACR ≥70	81 (66.9)	
	aACR 100	62 (51.2)	

*n* number of patients who satisfy the criteria, *m* number of patients with an assessment in the time period

## Oral presentations 1

### O17 Defect of adaptive immunity in ADA2 deficiency patients

#### Francesca Schena^1^, Stefano Volpi^1^, Roberta Caorsi^1^, Federica Penco^1^, Claudia Pastorino^1^, Francesca Kalli^2^, Alessia Omenetti^1^, Sabrina Chiesa^1^, Arinna Bertoni^1^, Paolo Picco^1^, Gilberto Filaci^2^, Ivona Aksentijevich^3^, Alice Grossi^4^, Isabella Ceccherini^4^, Alberto Martini^1^, Elisabetta Traggiai^5^, Marco Gattorno^1^

##### ^1^Second Pediatric Division, Giannina Gaslini Institute, Genova, Italy; ^2^CEBR, Università, Genova, Italy; ^3^National Human Genome Research Institute, National Institute of Health, Bethesda, MD, USA; ^4^Medical Genetics, G. Gaslini Institute, Genova, Italy; ^5^Novartis Institute for Research in Biomedicine, Basel, Switzerland

###### **Presenting author:** Francesca Schena

**Introduction:** ADA2 Deficiency is a new autoinflammatory disease characterized by systemic vasculopathy and episodes of strokes, but some patients can present mild immunodeficiency. The defect is due to a loss of function mutation of *CECR1* gene, coding for Adenosine Deaminase 2 protein. This protein regulates the catabolism of extracellular adenosine, which we have shown is an important regulator of Class Switch Recombination in B lymphocytes.

**Objectives:** As some DADA2 patients show hypogammaglobulinemia and recurrent infections, we investigated the role of CECR1 mutation on adaptive immunity. Therefore we decided to characterize peripheral B and T lymphocytes of DADA patients to directly address if ADA2 mutation affects B-cell and T cell function.

**Methods:** 9 patients carrying mutations in *CECR1* were examined. They showed clinical history with livedo reticularis, fever, vasculitis and neurological symptoms. 2 patients presented hypogammaglobulinemia, whereas others 2 patients present recurrent infections. We analyzed peripheral B and T cell phenotype by flow cytometry. B cells isolated from healthy donors and DADA2 patients have been cultured alone or in coculture with CD4+ T cells and *in vitro* B cell proliferation has been evaluated by CFSE dilution. *In vitro* B cell differentiation to Immunoglobulin secreting cells in response to TLR9 agonist and T cell help has been evaluated by ELISA/ELISPOT assay. Moreover ex vivo and in vitro cytokines production from T cells has been evaluated.

**Results:** Flow cytometric analysis showed a significant reduction of total B cells in adult population compared with age matched controls. Intriguingly, a significant decrease in the proportion of memory B cell compartment (CD19+CD27+) was observed in DADA2 patients. Moreover we observed a reduced frequency of CD4+ and CD8+ cells, whereas the subpopulations of T cells resulted normal. To investigate B cell function in DADA2 patients, B cells were isolated from patient’s peripheral blood and cultured in vitro with TLRs and CD40L stimulation. Under these conditions, the rate of proliferation of B cells has resulted strongly abrogated as compared to normal controls. We addressed the interaction of B and T cells, essential for generation of memory B and for T cell dependent response of B cells. To this end, we isolated simultaneously from DADA2 patients CD19+ B cells and CD4+ T cells. Analysis of proliferation and differentiation showed that in vitro proliferation of B cells is not sustained from patient’s T cells and IgM and IgA secretion is significantly reduced with respect to HD B lymphocytes in the presence of CD4 helper T cells obtained from DADA2 patients. Moreover intracellular IFNg, TNFa and IL17 did not differ between controls and DADA2 patients, whereas the evaluation of IL4 and IL21 are ongoing.

**Conclusion:** Our findings suggest that CECR1 mutation could lead to a defect in B cell function and to a reduced T cell help of B cells.

**Disclosure of Interest**

None Declared

### O18 Previously undescribed SAVI-associated mutations identify a novel region in human sting necessary for the control of type I interferon signalling

#### Isabelle Melki^1,2,3^, Yoann Rose^1^, Carolina Uggenti^1^, Marie-Louise Fremond^1^, Lien Van Eyck^1^, Naoki Kitabayashi^1^, Marco Gattorno^4^, Stefano Volpi^4^, Olivero Sacco^5^, Isabelle Meyts^6^, Marie-Anne Morren^7^, Carine Wouters^8^, Eric Legius^9^, Isabelle Callebaut^10^, Christine Bodemer^11^, Frederic Rieux-Laucat^12^, Mathieu Rodero^1^, Yanick Crow^1^

##### ^1^Laboratory of Neurogenetics and Neuroinflammation, Imagine Institute, Paris, France; ^2^General Pediatrics - Internal Medicine - Infectious diseases, Robert Debré Hospital - APHP, Paris, France; ^3^Pediatric Immuno-Haematology and Rheumatology Unit, Necker Hospital - APHP, Paris, France; ^4^UO Pediatria 2 Reumatologia, Genova, Italy; ^5^UO Pneumologia, G. Gaslini, Genova, Italy; ^6^Immunology, UZLeuven, Leuven, Belgium; ^7^Dermatology, UZLeuven, Leuven, Belgium; ^8^Rheumatology, UZLeuven, Leuven, Belgium; ^9^Genetics, UZLeuven, Leuven, Belgium; ^10^MPMC, Sorbonne University — UMR CNRS 7590, UPMC Sorbonne Paris Cité, Muséum National d’Histoire Naturelle, Paris, France, IRD UMR 206, Paris, France; ^11^Dermatology, Necker Hospital - APHP, Paris, France; ^12^INSERM UMR 1163; Laboratory of Immunogenetics of Pediatric Autoimmunity, Imagine Institute, Paris, France

###### **Presenting author:** Isabelle Melki

**Introduction:** Gain-of-function mutations in *TMEM173* encoding STING (stimulator of interferon genes) underlie a very recently described type I interferonopathy referred to as SAVI (STING Associated Vasculopathy in Infancy). Thirteen patients belonging to ten different families have been published so far, all carrying heterozygous substitutions involving one of three residues at positions 147, 154 and 155, located in a small linker region connecting the N-terminal transmembrane domain of STING to the C-terminal cyclic dinucleotide-binding domain.

It has been shown that the constitutive activation driven by these mutants is mediated by exit of STING from the endoplamic reticulum (ER) and stable sequestration within the ER-Golgi intermediate compartment (ERGIC)/Golgi compartment. This triggers the downstream protein kinase TBK1, which then phosphorylates IRF3 and induces the transcription of interferon stimulated genes (ISGs).

**Objectives:** To report three new individuals variably exhibiting the core features of SAVI including systemic inflammation, destructive skin lesions and interstitial lung disease.

**Methods:** One patient was studied by exome sequencing and all three by Sanger sequencing of the candidate gene *TMEM173*. Interferon alpha titres were assessed by Single Molecule Array (Simoa™) technology and ISGs were measured by qPCR on PBMCs. Functional studies were performed using site-directed mutagenesis, studying the response to cGAMP in a HEK 293 T transfection system. TBK1, IRF3 and STAT1 phosphorylation were also studied.

**Results:** Although all three patients demonstrated characteristic features of SAVI, in two individuals the disease was essentially limited to the skin. Furthermore, none of the three experienced recurrent fevers, and bacterial infections were seen, thus perhaps broadening the phenotypic spectrum.

All three patients demonstrated high interferon alpha titres in serum and / or increased expression of ISGs.

Molecular and *in vitro* data revealed that the pathology in these patients is due to missense substitutions at positions 206, 281 and 284 of the human STING protein. These mutations confer cGAMP independent constitutive activation of type I interferon signalling through TBK1, and independent from the alternative STING pathway triggered by membrane fusion of enveloped RNA viruses. Structural analysis indicates that these three amino acids lie in a discrete region of the protein - thereby providing a physical explanation for the human genetic findings, and implicating a novel cluster of amino acids in STING as functionally important in the regulation of type I interferon signalling.

**Conclusion:** Our data clearly implicate a novel region of STING as important in type I interferon signalling, leading us to speculate that the surface-exposed arginine residues at 281 and 284 may be involved in ER retention or in binding to a negative regulator of STING signalling. Thus, the findings presented here suggest a previously unappreciated aspect of the control of STING.

**Disclosure of Interest**

None Declared

### O19 Efficacy of the Janus kinase 1/2 inhibitor ruxolitinib in the treatment of vasculopathy associated with TMEM173-activating mutations in three children

#### Marie-Louise Frémond^1^, Mathieu P. Rodero^1^, Nadia Jeremiah^2^, Alexandre Belot^3^, Eric Jeziorski^4^, Darragh Duffy^5^, Didier Bessis^6^, Guilhem Cros^7^, Gillian I. Rice^8^, Bruno Charbit^5^, Anne Hulin^9^, Nihel Khoudour^9^, Consuelo Modesto Caballero^10^, Christine Bodemer^11^, Monique Fabre^12^, Laureline Berteloot^13^, Muriel Le Bourgeois^14^, Philippe Reix^15^, Thierry Walzer^16^, Despina Moshous^7^, Stéphane Blanche^7^, Alain Fischer^7^, Brigitte Bader-Meunier^7^, Frédéric Rieux-Laucat^2^, Yanick Crow^1^, Bénédicte Neven^7^

##### ^1^Laboratory of Neurogenetics and Neuroinflammation, Paris, France; ^2^Laboratory of Immunogenetics of Pediatric Autoimmunity, Imagine Institute, Paris, France; ^3^Pediatric Rheumatology, Nephrology and Dermatology Department, Hospices Civils de Lyon, Lyon, France; ^4^Pediatrics Department, Centre Hospitalier Universitaire de Montpellier, Montpellier, France; ^5^Laboratory of Dendritic Cell Immunobiology, Institut Pasteur, Paris, France; ^6^Dermatology Department, Centre Hospitalier Universitaire de Montpellier, Montpellier, France; ^7^Pediatric Hematology-Immunology and Rheumatology Department, Hôpital Necker-Enfants Malades, Paris, France; ^8^Manchester Centre for Genomic Medicine, University of Manchester, Manchester, United Kingdom; ^9^Pharmacology and Toxicology Laboratory, Hôpital Universitaire Henri Mondor, Créteil, France; ^10^Pediatric Rheumatology Department, Hospital Universitari Vall d’Hebron, Barcelona, Spain; ^11^Dermatology Department, Hôpital Necker-Enfants Malades, Paris, France; ^12^Pathology Department, Hôpital Necker-Enfants Malades, Paris, France; ^13^Pediatric Radiology Department, Hôpital Necker-Enfants Malades, Paris, France; ^14^Pediatric Pneumology Department, Hôpital Necker-Enfants Malades, Paris, France; ^15^Pneumology Department, Hospices Civils de Lyon, Lyon, France; ^16^Centre International de Recherche en Infectiologie, Université Lyon 1, Lyon, France

###### **Presenting author:** Marie-Louise Frémond

**Introduction:** Gain-of-function mutations in *TMEM173* encoding STING underlie a novel type I interferonopathy, minimally responsive to conventional immunosuppressive therapies and associated with high childhood morbidity and mortality.

**Objectives:** We hypothesized that inhibition of interferon (IFN) signaling could be used as a therapeutic approach in this disease. We describe here the medical and molecular consequences of treatment with ruxolitinib, an oral JAK1/2 inhibitor.

**Methods:** During follow-up of three children over a period of between 12 to 24 months, clinical data were collected and disease activity scores were determined for systemic, skin and lung manifestations. The effect of ruxolitinib on JAK-STAT signaling was assessed *in vitro* and *ex vivo* by transcriptomic analysis and STAT1 phosphorylation status.

**Results:** We observed marked clinical improvement in all three children in each of the central aspects of the phenotype – namely, destructive skin lesions, interstitial lung disease and systemic inflammation. This clinical effect was mirrored by *in vitro* and *ex vivo* data, where we recorded decreased STAT1 phosphorylation and reduced expression of both IFN-stimulated genes and a subset of genes associated with fever and vasculopathy. Tolerance of treatment was good without an obvious infectious risk.

**Conclusion:** These results suggest that JAK1 inhibition represents a promising therapeutic approach in *TMEM173-*mutated patients and may be also relevant to the treatment of other type I interferonopathies.

**Disclosure of Interest**

None Declared

### O20 Development of the autoinflammatory disease damage index (ADDI)

#### K Annink^1^, N ter Haar^1^, S Al-Mayouf^2^, G Amaryan^2^, J Anton^2^, K Barron^2^, S Benseler^2^, P Brogan^2^, L Cantarini^2^, M Cattalini^2^, A Cochino^2^, F De Benedetti^2^, F Dedeoglu^2^, A De Jesus^2^, O Dellacasa^2^, E Demirkaya^2^, P Dolezalova^2^, K Durrant^3^, G Fabio^2^, R Gallizzi^2^, R Goldbach-Mansky^2^, E Hachulla^2^, V Hentgen^2^, T Herlin^2^, M Hofer^2^, H Hoffman^2^, A Insalaco^2^, A Jansson^2^, T Kallinich^2^, I Koné-Paut^2^, A Kozlova^2^, J Kuemmerle-Deschner^2^, H Lachmann^2^, R Laxer^2^, A. Martini^2^, S Nielsen^2^, I Nikishina^2^, A Ombrello^2^, S Ozen^2^, E Papadopoulou-Alataki^2^, P Quartier^2^, A Ravelli^2^, D Rigante^2^, R Russo^2^, A Simon^2^, M Trachana^2^, Y Uziel^2^, M Gattorno^2^, J Frenkel^1^

##### ^1^UMC Utrecht, Utrecht, Netherlands; ^2^The ADDI consortium, Genua, Italy; ^3^Autoinflammatory Alliance, San Fransisco, CA, USA

###### **Presenting author:** N ter Haar

**Introduction:** Autoinflammatory diseases cause systemic inflammation which can result in damage to multiple organs. A validated instrument to measure damage is essential to quantify damage in individual patients, and to compare disease outcomes in clinical studies. At this moment, there is no instrument to qualify damage in autoinflammatory diseases.

**Objectives:** Our objective was to develop a common autoinflammatory disease damage index (ADDI) for Familial Mediterranean Fever (FMF), Cryopyrin Associated Periodic Syndromes (CAPS), Tumor Necrosis Factor Receptor Associated Periodic Syndrome (TRAPS) and Mevalonate Kinase Deficiency (MKD).

**Methods:** We have developed the ADDI by consensus building based on the Delphi method. The top 40 enrollers of patients in the Eurofever registry and nine important experts from the Americas participated in multiple rounds of online surveys to suggest and select items and definitions. Patients fulfilled a role in this project in order to develop a damage index that represents what is important for patients. Further, 22 patients and parents of patients rated damage items and suggested new items.

A consensus meeting was held to finalize the items and definitions. Lastly, we decided the scoring of each item based on the results of a decision making survey (1000minds), which was completed by experts and (parents of) patients.

**Results:** More than 80% of the 49 experts and 22 patients completed the online surveys. At the consensus meeting, 31 experts and a patient representative reached consensus on the inclusion and definition of the items. The scoring of each item was based on the 1000minds program, which was completed by 37 experts and fourteen patients. The preliminary ADDI contains eighteen relevant damage items, categorised per organ system. The damage items are considered relevant by the experts and all cause an important burden for patients according to the experts and (parents) of patients. Examples of damage items of the preliminary ADDI are hearing loss, amyloidosis, infertility and serosal scarring. It will take approximately five to ten minutes to complete the ADDI.

**Conclusion:** An instrument to measure damage caused by autoinflammatory diseases is developed based on consensus building with multiple rounds of online surveys and a consensus meeting. Patients fulfilled a significant role in this process.

**Disclosure of Interest**

K. Annink: None Declared, N. ter Haar: None Declared, S. Al-Mayouf: None Declared, G. Amaryan: None Declared, J. Anton: None Declared, K. Barron: None Declared, S. Benseler: None Declared, P. Brogan: None Declared, L. Cantarini: None Declared, M. Cattalini: None Declared, A. Cochino: None Declared, F. De Benedetti: None Declared, F. Dedeoglu: None Declared, A. De Jesus: None Declared, O. Dellacasa ✝ : None Declared, E. Demirkaya: None Declared, P. Dolezalova: None Declared, K. Durrant: None Declared, G. Fabio: None Declared, R. Gallizzi: None Declared, R. Goldbach-Mansky: None Declared, E. Hachulla: None Declared, V. Hentgen: None Declared, T. Herlin: None Declared, M. Hofer: None Declared, H. Hoffman: None Declared, A. Insalaco: None Declared, A. Jansson: None Declared, T. Kallinich: None Declared, I. Koné-Paut: None Declared, A. Kozlova: None Declared, J. Kuemmerle-Deschner: None Declared, H. Lachmann: None Declared, R. Laxer: None Declared, A. Martini: None Declared, S. Nielsen: None Declared, I. Nikishina: None Declared, A. Ombrello: None Declared, S. Ozen: None Declared, E. Papadopoulou-Alataki: None Declared, P. Quartier: None Declared, A. Ravelli: None Declared, D. Rigante: None Declared, R. Russo: None Declared, A. Simon: None Declared, M. Trachana: None Declared, Y. Uziel: None Declared, M. Gattorno: None Declared, J. Frenkel Grant / Research Support from: Novartis provided an unrestricted grant for the consensus meeting. They were not present at the meeting and did not have influence on the selection of experts/patients, the content of the meeting or the ADDI itself.

### O21 The phenotype and genotype of mevalonate kinase deficiency: a series of 114 cases from the Eurofever registry

#### Nienke ter Haar^1^, Jerold Jeyaratnam^1^, Helen Lachmann^1^, Anna Simon^1^, Paul Brogan^1^, Matteo Doglio^1^, Marco Cattalini^1^, Jordi Anton^1^, Consuelo Modesto^1^, Pierre Quartier^1^, Esther Hoppenreijs^1^, Silvana Martino^1^, Antonella Insalaco^1^, Luca Cantarini^1^, Loredana Lepore^1^, Maria Alessio^1^, Inmaculada Calvo Penades^1^, Christina Boros^1^, Rita Consolini^1^, Donato Rigante^1^, Ricardo Russo^1^, Jana Pachlopnik Schmid^1^, Thirusha Lane^1^, Alberto Martini^1^, Nicolino Ruperto^1^, Joost Frenkel^1^, Marco Gattorno^1^

##### ^1^Eurofever Project, University Medical Center Utrecht, Utrecht, Netherlands

###### **Presenting author:** Jerold Jeyaratnam

This abstract is not included here as it has already been published.

### O22 Autoinflammatory diseases: role of next-generation sequencing

#### Chiara Passarelli^1^, Elisa Pisaneschi^1^, Virginia Messia^2^, Manuela Pardeo^2^, Antonio Novelli^1^, Fabrizio Debenedetti^2^, Antonella Insalaco^2^

##### ^1^Division of Medical Genetics, Roma, Italy; ^2^Division of Rheumatology, Ospedale Pediatrico Bambino Gesù IRCCS, Roma, Italy

###### **Presenting author:** Chiara Passarelli

**Introduction:** Autoinflammatory disorders (AIDs) are an expanding group of complex diseases marked by periodic or chronic systemic inflammation. Mutations in approximately 25 genes have been associated with autosomic recessive or dominant autoinflammatory AIDs. Genetic analysis of based on the candidate gene has a low efficiency and it is expensive and time consuming. Next Generation Sequencing (NGS) has emerged as a new diagnostic tool in this field, with the goals of allowing for faster genetic diagnosis and of providing a better comprehension of the phenotype/genotype correlation in AID patients.

**Objectives:** To evaluate the results obtained by the application of NGS in a cohort of patients affected by undefined AIDsevaluated at our center

**Methods:** In this study we enrolled 242 patients evaluated at our center from 2010 to march 2016 affected by undefined AIDs. In the first 4 years of the study, we performed Targeted resequencing on 179 patients using a panel including 11 AIDs related genes (MVK, MEFV, NRLP12, NRLP3, NOD2, TNFRSF1A, PSTPIP1, IL1RN, LPIN2, IL36RN, PSMB8). The customized panel was subsequently implemented, adding 14 genes more recently demonstrated to as involved in AIDs, used to analyse 63 additional patients. Sequencing analysis were performed on MiSeq® sequencing platform (Illumina, San Diego, CA) and all variants identified have been confirmed by Sanger sequencing. Validated variants were studied by *in silico* analysis using SIFT and PolyPhen softwares in order to predict their possible functional impact.

**Results:** NGS analysis led to the identification of 80 patients with different variants in the typically AIDs-associated genes: MVK, MEFV, NRLP12, NRLP3, NOD2, TNFRSF1A, PSTPIP1, with a detection rate of 31%. We took into account only variants with a frequency in the global population <1%, as reported in dbSNP and Ensembl databases. 25% of patients showed monogenic variants, both novel or already known, while variants in two or more genes were found in 5% of patients. In details, 33% of total variants was found in NOD2 gene, 19% in MEFV, 16% in NLRP12, 12% in NLRP3, 9% in PSTPIP1 and 5% in both TNFRSF1A and MVK. Several of these variants do not have a clear functional significance yet, while some of them are described as risk factors. We performed 19 familial study to unravel the segregation of some variants.

**Conclusion:** NGS allowed the identification of a large amount of genetic variants in different genes. The major challenge is represented by the interpretation of the clinical relevance of the identified variants, particularly of variants that are found at low, but >1% frequency in various populations. These may indeed function as susceptibility alleles to inflammation rather than disease associated mutations.

Some patients show variants in different genes that may cooperate to cause a clinical phenotype or, on the other hand, may be irrelevant. Therefore large-scale population studies, in vitro functional assays and careful correlation between genetic information and phenotypic data are needed. Cooperations with clinicians as well as collaborations with different laboratories using NGS is crucial in order to generate information that are scientifically rigorous and relevant for patient management.

**Disclosure of Interest**

C. Passarelli: None Declared, E. Pisaneschi: None Declared, V. Messia: None Declared, M. Pardeo: None Declared, A. Novelli: None Declared, F. Debenedetti Grant / Research Support from: Novartis, Novimmune, Hoffmann-La Roche, SOBI, AbbVie, Pfizer, A. Insalaco: None Declared

### O23 Effectiveness of childhood vaccinations in CAPS patients treated with Canakinumab: results from an open-label phase 3 extension study

#### P.A. Brogan^1^, M. Hofer^2^, J.B. Kuemmerle-Deschner^3^, B. Lauwerys^4^, A. Speziale^5^, X. Wei^6^, R. Laxer^7^

##### ^1^UCL Institute of Child Health and Great Ormond Street Hospital NHS Foundation Trust, Department of Paediatric Rheumatology, London, United Kingdom; ^2^Unité Romande de Rhumatologie Pédiatrique, Hospitalier Universitaire Vaudois, Lausanne, Switzerland; ^3^University Hospital Tuebingen, Tuebingen, Germany; ^4^Cliniques Universitaires Saint-Luc and Université Catholique de Louvain, Brussels, Belgium; ^5^Novartis Pharma AG, Basel, Switzerland; ^6^Novartis Pharma, Beijing, China; ^7^University of Toronto, The Hospital for Sick Children, Ontario, Canada

###### **Presenting author:** P.A. Brogan

**Introduction:** Canakinumab (CAN) has been shown not to impair antibody production following vaccination in children in an open-label phase 3 study (NCT01302860) [1]. Here we present the results from the extension of this study.

**Objectives:** To evaluate the presence of protective antibody levels following immunisation with inactivated vaccines in CAPS patients during extension study.

**Methods:** Patients who completed the core study were allowed to continue into the extension study on the standard dosing regimen of 2 mg/kg subcutaneous CAN every 8 weeks or on last dose/dosing regimen received in the core study. Vaccination response was evaluated using post-vaccination antibody titres at 4 and 8 weeks after immunisation. Patients were considered assessable for an antibody response to a specific vaccination if they had a measurement of antibody titre 0-14 days post-vaccination (pre-vaccination assessment) and at least 1 subsequent measurement of antibody titre at 4 weeks and/or 8 weeks post-vaccination. However, for patients with adequate pre-dose antibody titres and maintained during the trial, the specific patient vaccination was deemed non-assessable.

**Results:** During the extension phase, of 17 patients (≤6 years), 4 received 8 types of vaccinations against *Corynebacterium diphtheria*, *Bordetella pertussis*, *Neisseria meningitidis*, *Clostridium tetani*, influenza type A and type B, *Haemophilus influenza* B, *Streptococcus pneumoniae*, or hepatitis B. Of 20 unique patient-vaccination cases, 17 were assessable for a vaccination response, whereas for the remaining 3, pre-dose antibody titre was not available. For 16 (94.1%) assessable cases, post-vaccination antibody titres increased above protective levels. For one patient who received Tetravec formulation (diphtheria, tetanus and acellular pertussis combination), the response observed for 1 (vaccination against *Clostridium tetani*) of the 3 vaccines included in Tetravac represented optical density rather than antibody concentrations and hence considered non-evaluable. For 19/20 patient-vaccinations, including those without pre-dose antibody titres, protective levels were observed during the study, which were maintained throughout the extension.

**Conclusion:** Canakinumab appeared to have no effect on post-vaccination antibody production following the administration of non-live vaccines in CAPS patients.

**References**

1. Brogan P, et al. *Arthritis Rheumatol*. 2015;67:(S10).

**Trial registration identifying number:** NCT01576367

**Disclosure of Interest**

P. Brogan Consultant for: Roche Pharmaceuticals and SOBI, M. Hofer Consultant for: Abbvie and Novartis, Speaker Bureau of: Abbvie, J. Kuemmerle-Deschner Grant / Research Support from: Novartis, Consultant for: Novartis, Sobi and Baxalta, B. Lauwerys: None Declared, A. Speziale Employee of: Novartis, X. Wei Employee of: Novartis, R. Laxer Grant / Research Support from: Novartis and Abbvie, Consultant for: Novartis and Abbvie

### O24 Baseline characteristics of 116 patients with cronic non-bacterial osteomyelitis (CNO) included in a national longitudinal cohort study

#### Antonella Insalaco^1^, Denise Pires Marafon^1^, Martina Finetti^2^, Manuela Pardeo^3^, Silvana Martino^4^, Marco Cattalini^5^, Maria Alessio^6^, Francesca Orlando^7^, Andrea Taddio^7^, Serena Pastore^7^, Elisabetta Cortis^8^, Angela Miniaci^9^, Nicola Ruperto^2^, Alberto Martini^2^, Fabrizio De Benedetti^1^, Marco Gattorno^2^

##### ^1^Division of Rheumatology, Ospedale Pediatrico Bambino Gesù IRCCS, Roma, Italy; ^2^Pediatria II Reumatologia PRINTO, Istituto Giannina Gaslini, Genova, Italy; ^3^Division of Rheumatology, Ospedale Pediatrico Bambino Gesù, Roma, Italy; ^4^Paediatrics Departments, Clinica Pediatrica Universita’ di Torino, Torino, Italy; ^5^Unità di immunologia e Reumatologia Pediatrica, Clinica Pediatrica Università Brescia, Spedali Civili, Brescia, Italy; ^6^Dipartimento di Pediatria, Università di Napoli Federico II, Napoli, Italy; ^7^Scienze della Riproduzione e dello Sviluppo, Università di Trieste IRCCS Burlo Garofalo, Trieste, Italy; ^8^Struttura Complessa Pediatria, Ospedale Santa Maria della Stella, Orvieto, Italy; ^9^Ambulatorio di Reumatologia, Aziensa Ospedaliera Universitaria Sant’Orsola Malpighi, Bologna, Italy

###### **Presenting author:** Antonella Insalaco

**Introduction:** Chronic nonbacterial osteomyelitis (CNO) is one of the most common autoinflammatory bone disorders in childhood. Until now few data are available about the treatment and outcome of these patients. Several studies, all uncontrolled, have suggested the efficacy of non-steroidal anti-inflammatory drugs (NSAIDs), glucocorticoids, methotrexate, bisphosphonates and tumour necrosis factor α (TNF-α) inhibitors. These drugs are differently used with variable degree of response

**Objectives:** To evaluate demographics, clinical features, laboratory and radiological assessment, therapeutic response in a large cohort of Italian CNO patients

**Methods:** Since 2015 CNO patients from 8 Italian centers were included in the new longitudinal section of the Eurofever registry. Demographic data, clinical manifestations and response to treatment were analysed.

**Results:** baseline data from 116 patients are so far available. 72 (61%) females, 111 (95.7%) Caucasian with a median age at disease onset of 10 years (IQR 7.8–11.7). Disease duration at diagnosis was 7.1 months (IQR 3.1–18.5). Disease course was continuous in 73 patients (62.9%), recurrent in 35 (30.2%) and continuous and recurrent in 8 (7%). All patients presented with musculoskeletal involvement at the onset of disease (bone pain 91.4%, arthralgia 84.5%, bone alteration 55.2%, osteitis 55%, osteolytic lesions 48.3%, myalgia and arthritis were found in a minority of the patients). Other features include constitutional symptoms in 64 patients (55.2%), muco-cutaneous manifestations in 17 (14.7%), gastrointestinal involvement in 10 (8.6%) and neurological manifestations in 4 (3.4%). At diagnosis erythrocyte sedimentation rate (ESR) and C-reactive protein (CRP) were elevated respectively in 84 (72.4%) and 62 (53.4%), with 26 (22.4%) patients having ESR and CRP both normal. Serum amyloid A was elevated in 34 out of the 50 patients tested (68%). MRI/CT was performed in 107 patients (56 Whole Body MRI, 52 local MRI, 19 CT), while bone scintigraphy in 90 patients, leading to the detection of a median of 4 lesions (range 1-21). A bone biopsy was performed in 52 patients. At the baseline visit, thirty-one (26.7%) patients had received NSAIDs, 8 (7%) were treated with pamidronate, 29 (25%) with glucocorticoids, 8 with anakinra (7%), 7 (6%) with sulfasalazine. Etanercept was used in 5 patients, Methotrexate was used in 4, adalimumab, colchicine in 1 patient

**Conclusion:** In this study we describe the baseline demographic, clinical, laboratory and radiological findings and therapy in a cohort of patients affected by CNO. Treatment used at baseline is extremely variable. Analysis of the outcome of bone lesions and of the efficacy and safety of the various treatments is ongoing on the data available from follow-up visits

**Disclosure of Interest**

A. Insalaco: None Declared, D. Pires Marafon: None Declared, M. Finetti: None Declared, M. Pardeo: None Declared, S. Martino: None Declared, M. Cattalini: None Declared, M. Alessio: None Declared, F. Orlando: None Declared, A. Taddio: None Declared, S. Pastore: None Declared, E. Cortis: None Declared, A. Miniaci: None Declared, N. Ruperto: None Declared, A. Martini: None Declared, F. De Benedetti Grant / Research Support from: Novartis, Novimmune, Hoffmann-La Roche, SOBI, AbbVie, Pfizer, M. Gattorno: None Declared

### O25 Analyses of 247 patients with recurrent inflammation of unknown cause

#### Charlotte Eijkelboom^1^, Nienke ter Haar^1^, Luca Cantarini^2^, Martina Finetti^3^, Paul Brogan^4^, Pavla Dolezalova^5^, Isabelle Koné-Paut^6^, Antonella Insalaco^7^, Marija Jelusic-Drazic^8^, Liliana Bezrodnik^9^, Mari Carmen Pinedo^10^, Valda Stanevicha^11^, Marielle van Gijn^1^, Silvia Federici^3^, Nicolino Ruperto^3^, Joost Frenkel^1^, Marco Gattorno^3^

##### ^1^UMCU, Utrecht, Netherlands; ^2^University of Siena, Siena, Italy; ^3^IGG, Genova, Italy; ^4^GOSH, London, United Kingdom; ^5^General University Hospital, Prague, Czech Republic; ^6^Bicêtre hospital, Le Kremlin-Bicêtre, France; ^7^OPBG, Rome, Italy; ^8^University Hospital Centre, Zagreb, Croatia; ^9^Hospital de Ninos Ricardo Gutierrez, Buenos Aires, Argentina; ^10^Hospital de Cruces, Barakaldo, Spain; ^11^Stradins University, Riga, Latvia

###### **Presenting author:** Charlotte Eijkelboom

**Introduction:** Auto-inflammatory diseases (AIDs) are a heterogeneous group of disorders caused by dysfunction of the innate immune system. Although many genetic mutations causing AIDs have been discovered, there is still a large group of patients with recurrent inflammation of unknown cause.

**Objectives:** Our objective is to describe the clinical features and available genetic data of patients with an undefined AID.

**Methods:** Clinical and genetic data from patients with an undefined AID were retrospectively collected in the Eurofever registry. Clinical classification criteria for FMF, MKD, TRAPS and CAPS as defined by Federici et al. were applied on the dataset [1].

**Results:** Out of the 268 patients in the Eurofever database registered with an undefined AID, 21 were excluded from further analyses because of incomplete clinical data (20) or a positive mutation for TRAPS (1). The median age of disease onset was 4.2 years (interquartile range 1.3–12.8). Patients had a median of 12 (5–15) episodes per year with a median duration of 4 (3–7) days. In 47 patients relatives were affected as well. In 204 patients analysis of one or multiple genes was performed. In 31 patients genetic variants, excluding benign polymorphisms, were found in the *MEFV* (12), *TNFRSF1A* (8), *NOD2* (9), *NLRP3* (6), *MVK* (3) and *NLRP12* gene (3). None of these variants were confirmative for diagnosis of a known AID. Patients with positive genetic screening more often had relatives affected (p < 0.001, Chi-squared) and had more episodes per year (p = 0.035, Mann-Whitney U).

Clinical manifestations are shown in the table below. NSAIDs were beneficial in 108/128 patients, but rarely completely effective. Corticosteroids were beneficial in 109/118 patients (51 complete, 58 partial response). Of 50 patients treated with colchicine, 10 had a complete response and 22 had a partial response. Of 15 patients treated with anakinra, 7 had a complete response and 6 a partial response. There was no correlation between the response to medication and presence of genetic variants (Mann-Whitney U).

When applying the clinical classification criteria [1], 22 patients were classified as CAPS, 37 as TRAPS, 15 as MKD and 12 as FMF. Seven patients scored positive for multiple diagnoses. Patients classified as TRAPS had a better response to anakinra (p = 0.036, Mann-Whitney U). There were no correlations between the disease classified by the clinical criteria and the presence of specific gene variants (Fisher’s exact).

**Conclusion:** This study describes the clinical features of a group of patients with undefined periodic inflammation. Almost one third of the patients (32%) had a positive clinical classification score for a hereditary AID.

**References**

1. Federici S, Sormani MP, Ozen S, Lachmann HJ, Amaryan G, Woo P, et al. Evidence-based provisional clinical classification criteria for autoinflammatory periodic fevers. Ann Rheum Dis. England; 2015 May;74(5):799–805.

**Disclosure of Interest**

None Declared

**Table 5 (abstract O25).** Clinical manifestations during episodes

	Always^a^	Sometimes/often^a^	Most common complaints^a^
Muco-cutaneous	22	47	Aphthous stomatis (27)
Gastro-intestinal	14	41	Abdominal pain (47)
Musculoskeletal	21	46	Arthralgia (61), myalgia (44)
Ocular	2	12	Conjunctivitis (8)
Lymphoid	33	14	Enlarged cervical lnn (38)
Cardio-respiratory	3	15	Chest pain (13)
Neurological	15	26	Headache (32)
Genito-urinary	1	5	Urethritis/cystitis (3)
Constitutional	25	40	Fatigue (55), malaise (49)

^a^% of cohort

### O26 The multifaceted presentation of chronic recurrent multifocal osteomyelitis: a series of 466 cases from the Eurofever international registry

#### Hermann Girschick^1^, Martina Finetti^2^, Francesca Orlando^2,3^, Antonella Insalaco^4^, Gerd Ganser^5^, Susan Nielsen^6^, Troels Herlin^7^, Isabelle Koné-Paut^8^, Silvana Martino^9^, Marco Cattalini^10^, Jordi Anton^11^, Sulaiman Mohammed Al-Mayouf^12^, Michael Hofer^13^, Pierre Quartier^14^, Christina Boros^15^, Jasmin Kuemmerle-Deschner^16^, Susanne Schalm^17^, Maria Alessio^18^, Nicolino Ruperto^2^, Alberto Martini^19^, Annette Jansson^20^, Marco Gattorno^2^, on behalf of PRINTO and Eurofever registry

##### ^1^ Perinatal Centre of the Vivantes Klinikum, Clinic for Paediatric and Adolescent Medicine, Berlin, Germany; ^2^Pediatria II, Reumatologia; PRINTO, Istituto Giannina Gaslini, Genoa, Italy; ^3^Università di Napoli Federico II, Naples, Italy; ^4^Ospedale Pediatrico Bambino Gesù, Rome, Italy; ^5^St. Josef-Stift Hospital, Sendenhorst, Germany; ^6^Rigshospitalet, Copenhagen, Denmark; ^7^Skejby Sygehus, Aarhus University Hospital, Aarhus, Denmark; ^8^Le Kremlin-Bicêtre University Hospital, Paris-Sud University, Paris, France; ^9^Dipartimento di Scienze Pediatriche e dell’Adolescenza, University of Torino, Torino, Italy; ^10^Unita’ di Immunologia e Reumatologia Pediatrica, Clinica Pediatrica dell’Universita’ di Brescia, Spedali Civili, Brescia, Italy; ^11^Hospital Sant Joan de Déu, Barcelona, Spain; ^12^King Faisal Specialist Hospital and Research Center, Riyadh, Saudi Arabia; ^13^Centre Multisite Romand de Rhumatologie Pediatrique / Centre Hospitalier Universitaire Vaudois (CHUV), Lausanne, Switzerland; ^14^Paris-Descartes University, IMAGINE Institute, Necker Children’s Hospital, Paris, France; ^15^Women’s and Children’s Hospital, Adelaide, Australia; ^16^University Children’s Hospital Tuebingen, Tuebingen, Germany; ^17^Klinikum der Univeristat, Munchen, Germany; ^18^Dipartimento di Pediatria, Università di Napoli Federico II, Naples, Italy; ^19^Pediatria II, Reumatologia; PRINTO, Istituto Giannina Gaslini, Genoa, Italy; ^20^University Medical Centre, Munich, Germany

###### **Presenting author:** Hermann Girschick

**Introduction:** Chronic Recurrent Multifocal Osteomyelitis (CRMO) is a rare autoinflammatory disorder featured by sterile bone osteolytic lesions.

**Objectives:** To evaluate the demographic data and the clinical, instrumental and therapeutic features at baseline in a large series of CRMO patients enrolled in the Eurofever registry.

**Methods:** A web-based registry collected retrospective data on patients affected by CRMO. Participating hospitals included paediatric and adult centers with a specific interest in autoinflammatory diseases.

**Results:** Complete baseline information on 466 patients were available (M:F = 171/295, pediatrics/adults = 439/27). 441 patients were Caucasian, 2 Hispanic, 4 Afro-American, 4 Asian, 8 Arabian and 7 of unknown ethnicity. Mean onset age was 11 years (range 3 months-62 years). An adult onset (after the 18 years) was observed in 27 (5,8%) patients. The mean time from disease onset to final diagnosis was 1 year (range 0–15 years). Disease course was described as continuous in 197 (42%), recurrent in 244 (52%), continuous and recurrent in 25 patients (5%). At baseline, 464 patients (99,5%) displayed musculoskeletal symptoms (431 bone pain, 301 arthralgia, 58 myalgia, 72 monoarthritis, 54 oligoarthritis); 87 patients (19%) mucocutaneous manifestations (25 acne, 24 palmo-plantar pustolosis, 21 psoriasis, 16 papulo-pustular lesions, 9 urticarial rash); 37 patients (8%) displayed gastrointestinal symptoms (27 abdominal pain, 15 diarrhea). Among imaging techniques, whole body magnetic resonance was performed at baseline in 141 patients (30%), revealing a mean number of 7 lesions (range 1-26). Overall, 174 patients (37%) displayed metaphyseal, 105 (23%) epiphyseal, 72 (15%) diaphyseal, 116 (25%) pelvic, 105 (23%) vertebral, 45 (10%) thoracic, 72 (15%) tarsal, 12 (3%) carpal, 13 (3%) cranial and 89 (19%) clavicular lesions. Biopsy was performed in 281 patients (60%). Complete information concerning the treatment in the period between disease onset and the enrollment in the registry were available for 386 patients. 361 (94%) patients have been treated with NSAIDs (142 complete response); 111 (29%) with steroids (42 complete response), 63 (16%) with Bisphosphonates (31 complete response), 57 (15%) with Methotrexate (13 complete response), 46 (12%) with Sulfasalazine (18 complete response), 17 (4%) with Etanercept (7 with complete response), 8 (2%) with Infliximab (3 complete response) and 4 (1%) with Anakinra (2 with complete response). Eighty-one patients have recently entered in the longitudinal phase of the registry that is specifically devoted to the evaluation of long-term efficacy and safety of the different treatments performed during their follow-up.

**Conclusion:** In conclusion, this is the largest reported case series of CRMO patients. The study shows that the disease can present with a late onset and that the range of clinical manifestations is heterogeneous. At least in the early phases of the disease NSAIDs are the most widely used drugs with a complete response in almost 40%of the patients.

**Disclosure of Interest**

H. Girschick: None Declared, M. Finetti: None Declared, F. Orlando: None Declared, A. Insalaco: None Declared, G. Ganser: None Declared, S. Nielsen: None Declared, T. Herlin: None Declared, I. Koné-Paut: None Declared, S. Martino: None Declared, M. Cattalini: None Declared, J. Anton: None Declared, S. M. Al-Mayouf: None Declared, M. Hofer: None Declared, P. Quartier: None Declared, C. Boros: None Declared, J. Kuemmerle-Deschner: None Declared, S. Schalm: None Declared, M. Alessio: None Declared, N. Ruperto Grant / Research Support from: The G. Gaslini Hospital, which is the public Hospital where I work as full time public employee, has received contributions from the following industries for the coordination activity of the PRINTO network: BMS, GlaxoSmithKline (GSK), Hoffman-La Roche, Novartis, Pfizer, Sanofi Aventis, Schwarz Biosciences, Abbott, Francesco Angelini S.P.A., Sobi, Merck Serono. This money has been reinvested for the research activities of the hospital in fully independent manners besides any commitment with third parties., Speaker Bureau of: I received speaker’s bureaus and consulting fees from the following pharmaceutical companies: AbbVie, Amgen, Biogenidec, Alter, AstraZeneca, Baxalta Biosimilars, Biogenidec, Boehringer, BMS, Celgene, CrescendoBio, EMD Serono, Hoffman-La Roche, Italfarmaco, Janssen, MedImmune, Medac, Novartis, Novo Nordisk, Pfizer, Sanofi Aventis, Servier, Takeda, UCB Biosciences GmbH, A. Martini Grant / Research Support from: The G. Gaslini Hospital, which is the public Hospital where I work as full time public employee, has received contributions from the following industries for the coordination activity of the PRINTO network: BMS, GlaxoSmithKline (GSK), Hoffman-La Roche, Novartis, Pfizer, Sanofi Aventis, Schwarz Biosciences, Abbott, Francesco Angelini S.P.A., Sobi, Merck Serono. This money has been reinvested for the research activities of the hospital in fully independent manners besides any commitment with third parties., Speaker Bureau of: I received speaker’s bureaus and consulting fees from the following pharmaceutical companies: Abbvie, Boehringer, Celgene, CrescendoBio, Janssen, Meddimune, Novartis, NovoNordisk, Pfizer, Sanofi Aventis, Vertex, Servier., A. Jansson: None Declared, M. Gattorno Grant / Research Support from: Unrestricted grant from SOBI and NOVARTIS

### O27 Clinical features distinguishing patients with Chronic Recurrent Multifocal Osteomyelitis (CRMO) from patients with tumoral osteolytic lesions: a single center experience

#### Martina Finetti^1^, Marta Marchi^2^, Chiara Marini^2^, Matteo Doglio^2^, Clara Malattia^1^, Angelo Ravelli^1^, Alberto Martini^1^, Alberto Garaventa^3^, Marco Gattorno^1^

##### ^1^Pediatria II, Reumatologia, Istituto Giannina Gaslini, Genoa, Italy; ^2^Università di Genova, Genoa, Italy; ^3^Pediatria IV, Oncologia, Istituto Giannina Gaslini, Genoa, Italy

**Introduction:** Chronic Recurrent Multifocal Osteomyelitis (CRMO) is a rare autoinflammatory disorder featured by sterile bone osteolytic lesions. The differential diagnosis includes bone malignancies, benign tumors, bacterial subacute or chronic osteomyelitis.

**Objectives:** The aim of our study is to analyze the possible differences in the clinical presentation, laboratory and instrumental examinations between patients with CRMO and patients presenting osteolytic lesions secondary to malignancies.

**Methods:** 48 pediatric patients affected by CRMO (M:F = 33/15, mean onset age 9,6 years, range 4-14 years), 21 patients affected by Langerhans cell histiocytosis (LCH, M:F = 12/9, mean onset age 4,3 years, range 3 months -12 years) and of 10 patients affected by hemato-oncological disorder (lymphoma, sarcoma and neuroblastoma, M:F = 4/6, mean onset age 7 years, range 2-11 years) presenting with osteolytic lesions and followed by a single centre since 1999 to 2015 were enrolled in the study. Clinical features, acute phase reactants values and radiological investigations (whole-body magnetic resonance) were obtained at diagnosis and at last available follow-up. Onset features of CRMO patients have been compared to those of LCH and hemato-oncological patients. A univariate analysis was performed comparing first CRMO, LCH and hemato-oncological patients and then only CRMO and LCH patients. Nominal variables were analyzed using the *χ*2 test and Fisher’s exact test. Continuous variables were analyzed with the nonparametric Mann- Whitney *U* test and the Wilcoxon test.

**Results:** Comparison among the three categories of patients (CRMO, LCH and hemato-oncological), showed that patients with bone malignancies (but not Langerhans cell histiocytosis patients) displayed a significant higher incidence of systemic symptoms: elevated fever (>38 °C) and malaise in fact resulted prevalent in hemato-oncological cohort than in the other two categories (P respectively: <0,0001 and <0,01). CRP and LDH levels were significantly higher and hemoglobin levels lower in patients with bone malignancies than in patient with CRMO or LCH, whilst arthritis signs (pain, swelling and functional limitation) were more frequent in CRMO patients (P: <0,007). Comparing only CRMO with LCH patients, some differences in the onset variables were observed: onset age was higher in CRMO than in LCH patients (P <0,001). Some differences have also been detected in the radiological distribution of bone localizations: clavicle, distal fibula and foot were exclusive for CRMO. Moreover, patients with CRMO displayed a greater number of lesions than LCH. (Table)

**Conclusion:** Our study shows that some clinical manifestations and laboratory data could be useful for differential diagnosis between CRMO and bone malignancies. However, LCH represents the most difficult disease implicated in differential diagnosis with CRMO. Biopsy still represents the gold standard examination for a correct diagnosis.

**Disclosure of Interest**

None Declared

**Table 6 (abstract O27).** Comparison between CRMO and LCH cohort of patients

	Variables	CRMO	LCH	P	Odds ratio (OR)
Demographic manifestations	Mean onset age (years)	9,6	4,3	<0,001	
Clinical Manifestations	Musculoskeletal pain	48/48 (100%)	10/21 (48%)	<0,0001	0,06 (0,01/0,26)
	Swelling and/or functional limitations	31/48 (65%)	7/21 (33%)	<0,02	0,27 (0,09/0,81)
MR lesions site	Clavicle	11/48 (22,9%)	0/21 (0%)	<0,01	
	Distal Fibula	9/48 (19%)	0/21 (0%)	<0,05	
	Foot	10/48 (20,8%)	0/21 (0%)	<0,03	
	Mean number of MR lesions	6,5	2	<0,03	

### O28 Cryopyrin associated periodic syndromes (CAPS): immunological characterization of knock-in mouse model to exploit novel approaches for the modulation of the NLRP3 inflammasome

#### Arinna Bertoni^1^, Sonia Carta^2^, Enrica Balza ^2^, Patrizia Castellani^2^, Caterina Pellecchia^2^, Federica Penco^1^, Francesca Schena^1^, Silvia Borghini^3^, Maria Libera Trotta^4^, Claudia Pastorino^1^, Isabella Ceccherini^3^, Alberto Martini^5^, Marco Gattorno^5^, Anna Rubartelli^2^, Sabrina Chiesa^1^

##### ^1^Laboratorio di Immunologia delle Malattie Reumatiche, Giannina Gaslini Institute, Genova, Italy; ^2^Unità di Biologia Cellulare, IRCCS San Martino-Ist, Genova, Italy; ^3^Genetica Medica, Giannina Gaslini Institute, Genova, Italy; ^4^Università degli Studi di Genova, Genova, Italy; ^5^2° Pediatric Division, Giannina Gaslini Institute, Genova, Italy

###### **Presenting author:** Arinna Bertoni

This abstract is not included here as it has already been published.

## Oral presentations 2

### O29 Predicting which children with juvenile idiopathic arthritis will have a severe disease course: results from the ReACCh-Out cohort

#### Jaime Guzman^1^, Andrew Henrey^2^, Thomas Loughin^2^, Roberta Berard^3^, Natalie Shiff^4^, Roman Jurencak^5^, Susanne Benseler^6^, Lori Tucker^1^, on behalf of ReACCh-Out Investigators

##### ^1^Pediatrics, University of British Columbia, Vancouver, Canada; ^2^Statistics, Simon Fraser University, Burnaby, Canada; ^3^Pediatrics, Western University, London, Canada; ^4^Pediatrics, University of Florida, Gainesville, FL, USA; ^5^Pediatrics, University of Ottawa, Ottawa, Canada; ^6^Pediatrics, University of Calgary, Calgary, Canada

###### **Presenting author:** Jaime Guzman

**Introduction:** We previously reported that the disease course of children with juvenile idiopathic arthritis (JIA) could be described in four groups, based on five variables prioritized by patients, parents and clinicians (Ann Rheum Dis 2015;74:Suppl 2 609 doi:10.1136/annrheumdis-2015-eular.2396). The four groups were labeled as Mild (43.8% of children), Moderate (35.6%), Severe Controlled (9%) and Severe Persisting (11.5%).

**Objectives:** To develop a risk stratification method to estimate the probability of a severe disease course for each individual child with JIA at diagnosis.

**Methods:** We used data from 609 children followed in the Research in Arthritis in Canadian Children emphasizing Outcomes (ReACCh-Out) prospective inception cohort who attended at least 6 of 8 planned visits in the five years after diagnosis. We used information available at study enrolment to predict disease course in the subsequent 5 years. We divided the cohort at random into a training sample (75%) to develop candidate risk stratification methods and a testing sample (25%) to test the accuracy of stratification. This process was repeated 50 times for each of 100 candidate models derived from logistic regression, random forest, neural net or nearest neighbor methods. The 50 random splits allowed estimation of the stability and accuracy of the models in subjects that were not used in the development of the model, to select the most reliable, accurate and parsimonious method. Model performance was measured with c-index, Maximum Likelihood and Pearson statistics (higher values reflect better performance).

**Results:** For prediction of a severe disease course (Severe Controlled and Severe Persisting combined) the best performing model was derived using logistic regression. It had a c-index of 0.85 (95% CI 0.80, 0.90) and 91% of children assigned to the highest decile of risk actually had a severe disease course in the test sample (Table 7), compared to 5% of those assigned to the lowest decile of risk. The model used a combination of JIA category, active joint count, pattern of joint involvement and routine tests available at the first study visit. The model’s performance was better than using JIA category alone (c-index of 0.71; 95% CI 0.65, 0.79). For reference, the widely used Framingham’s Cardiovascular Risk Score has c-index values of 0.75 to 0.80 depending on the cohort.

**Conclusion:**

In this cohort, a risk stratification method that used variables usually available at diagnosis accurately estimated the risk of a severe JIA course during the following 5 years. Knowing the probability of a severe disease course at diagnosis will help parents and physicians better tailor the optimal aggressiveness of treatment for each child with JIA.

**Disclosure of Interest**

None Declared

**Table 7 (abstract O29).** Proportion of children with a severe disease course (severe controlled and severe persisting combined) in the test data across 50 re-samples, according to their decile of risk

Decile of risk	1) Logistic	2) Random forest	3) K-nearest neighbor	4) Neural network	5) JIA category alone
First (low)	0.05	0.04	0.16	0.11	0.05
Third	0.05	0.05	0.05	0.09	0.06
Sixth	0.10	0.13	0.17	0.10	0.32
Ninth	0.43	0.45	0.29	0.41	0.36
Tenth (high)	0.91	0.88	0.51	0.74	0.37
C-Index (95% CI)	0.85 (0.80, 0.90)	0.85 (0.82, 0.88)	0.67 (0.59, 0.75)	0.75 (0.71, 0.79)	0.71 (0.65, 0.79)
Maximum likelihood (95% CI)	−51 (−43, −59)	−58 (−49, −66)	−81 (−61, −100)	−107 (−99, −116)	−69 (−60, −77)
Pearson (95% CI)	47 (34, 61)	45 (37, 53)	12 (3, 20)	26 (18, 33)	13 (7, 19)

### O30 The vasculopathy of JDM; evidence of persistent endothelial injury, hypercoagulability, subclinical inflammation and increased arterial stiffness

#### Charalampia Papadopoulou^1^, Ying Hong^1^, Petra Krol^1^, Yiannis Ioannou^2^, Clarissa Pilkington^1^, Hema Chaplin^2^, Stephania Simou^1^, Marietta Charakida^3^, Lucy Wedderburn^1,2^, Paul Brogan^1^, Despina Eleftheriou^1,2^

##### ^1^Department of Paediatric Rheumatology, Great Ormond Street Hospital and UCL Institute of Child Health, London, United Kingdom; ^2^Arthritis Research UK, Centre for Adolescent Rheumatology, London, United Kingdom; ^3^Institute of Cardiovascular Sciences, University College London, London, United Kingdom

###### **Presenting author:** Charalampia Papadopoulou

**Introduction:** Vasculopathy is considered central to the pathogenesis of Juvenile Dermatomyositis (JDM). Moreover, the interplay between persistent JDM-vasculopathy, traditional cardiovascular risk factors, exposure to corticosteroids, and chronic inflammation could create a perfect storm for early atherogenesis. One major hurdle to the study and detection of the vasculopathy of JDM, monitoring of its trajectory over time and contribution to excess cardiovascular disease has been a lack of non-invasive biomarkers. Recently, we described a number of methods for detecting endothelial cell components in blood which allow non-invasive assessment of vascular injury: circulating endothelial cells (CEC), and endothelial microparticles (EMP).

**Objectives:** To explore the vasculopathy of JDM by assessing: (i) biomarkers of endothelial injury (CEC and EMP), subclinical inflammation (cytokines) and hypercoagulability (MP-mediated thrombin generation); and (ii) structural arterial changes (indicative of premature atherosclerosis), and their relation to disease activity and treatment in children with JDM.

**Methods:** 64 patients recruited to the UK JDM Cohort & Biomarker Study were included; median age 10.5 (range 6.9–13.7) years with median disease duration of 1.5 (0.3–4.7) years. 40 (62.5%) were females. Inactive disease was defined as per modified PRINTO criteria: no skin rashes, CK ≤ 150, CMAS ≥ 48, MMT8 ≥ 78, Physician’s global assessment ≤0.2 on a visual analogue scale. CECs and MPs were identified with immunomagnetic bead extraction and flow cytometry, respectively. MP function as assessed by thrombin generation was determined using a fluorogenic assay. Cytokines and chemokines were measured by electrochemiluminiscence. Arterial stiffness was assessed using pulse wave velocity (PWV). Results are expressed as median and range.

**Results:** CECs were higher in JDM patients at 68 (32-128) cell/ml compared to 12 (8-21) cells/ml in 66 age-sex matched healthy controls, p < 0.0001. Patients with active JDM had higher CEC than those with inactive JDM, p = 0.02. Patients with calcinosis had higher CEC compared to patients without calcinosis, p = 0.03. CEC counts significantly correlated with levels of inflammatory cytokines/chemokines implicated previously in JDM disease pathogenesis: interferon regulated Monocyte Chemoattractant Protein-1 (MCP-1; r = 0.63, p = 0.02) and interleukin-8 (IL-8; r = 0.65 and p = 0.01). Total circulating MP counts were also significantly higher in active JDM, 1781 (981–2616) ×10^3^/ml compared to inactive JDM, 1116 (263–1393) ×10^3^/ml, p = 0.02; and healthy controls 89 (25–236) ×10^3^/ml, p = 0.0001. These circulating MPs were predominantly of platelet and endothelial origin. Enhanced MP mediated thrombin generation was demonstrated in active compared to inactive JDM (p = 0.03) and controls (p = 0.001). Lastly, children with JDM had increased carotid-radial PWV adjusted for age compared to healthy controls (p = 0.005).

**Conclusion:** Our data demonstrate: 1. Increased endothelial damage in children with active JDM, possibly driven by pro-inflammatory cytokines; 2. High levels of circulating MP with propensity to drive thrombin generation and hence occlusive vasculopathy; and 3. Increased arterial stiffness, suggestive of accelerated atherosclerosis in patients with JDM. Validation of these biomarkers in multicentre prospective studies will provide data regarding their prognostic relevance.

**Disclosure of Interest**

None Declared

### O31 Online peer mentoring for adolescents with juvenile idiopathic arthritis: the iPeer2Peer Program

#### Lynn R. Spiegel^1,2^, Sara Ahola Kohut^3,4^, Jennifer Stinson^5,6^, Paula Forgeron^7^, Miriam Kaufman^2,8^, Nadia Luca^9^, Khush Amaria^3^, Mary Bell^10^

##### ^1^Rheumatology, The Hospital for Sick Children, Toronto, Canada; ^2^Pediatrics, University of Toronto, Toronto, Canada; ^3^Psychology, University of Toronto, Toronto, Canada; ^4^Child Health Evaluative Sciences, The Hospital for Sick Children, Toronto, Canada; ^5^Child and Evaluative Health Sciences, The Hospital for Sick Children, Toronto, Canada; ^6^Lawrence S. Bloomberg Faculty of Nursing, University of Toronto, Toronto, Canada; ^7^School of Nursing, University of Ottawa, Ottawa, Canada; ^8^Adolescent Medicine, The Hospital for Sick Children, Toronto, Canada; ^9^Rheumatology, Alberta Children's Hospital, Calgary, Canada; ^10^Rheumatology, Sunnybrook Research Institute, Toronto, Canada

###### **Presenting author:** Lynn R. Spiegel

This abstract is not included here as it has already been published.

### O32 Adjudication of infections in the pharmacovigilance in juvenile idiopathic arthritis patients (Pharmachild) treated with biologic agents and/or methotrexate

#### J Swart, F Boris, E Castagnola, A Groll, G Giancane, G Horneff, H-I. Huppertz, D Lovell, T Wolfs, M Hofer, E Alekseeva, V Panaviene, S Nielsen, J Anton, F Uettwiller, V Stanevicha, M Trachana, F De Benedetti, L M Ailioaie, E Tsitami, S Kamphuis, T Herlin, P Dolezalova, G Susic, F Sztajnbok, B Flato, A Pistorio, A Martini, N Wulffraat, N Ruperto

##### PRINTO, Genova, Italy

###### **Presenting author:** J Swart

**Introduction:** The Pharmachild study is aimed at observing children with JIA undergoing treatment primarily with biologics ± methotrexate (MTX). It is conducted by the participating centres of over 60 countries belonging to PRINTO and PReS.

**Objectives:** Primary endpoint of the study is safety in JIA patients undergoing immunosuppressive therapy. A preliminary analysis of infections has been performed by an independent Safety Ajudication Committee of 6 experts (3 pediatric rheumatologists and 3 pediatric infectious disease specialists).

**Methods:** The centres were asked to report all infections encountered by the patients followed for JIA. Data were checked by PRINTO and the medical monitor based on MedDRA dictionary. Upon completion of these steps, all eligible cases for adjudication, represented by serious/severe/very severe infections, were submitted to the expert Committee. The experts were asked to answer 6 questions. The events with consensus of at least 4/6 experts on more than 3/6 questions were considered. An additional analysis was performed on opportunistic infections, with referral to the recommendations by Withrop *et al*.[1], and on the correlation between infections and 3 treatment phases: MTX only, first biologic ± MTX and at least 2 sequential biologics ± MTX after failure of the first biologic.

**Results:** 7817 patients were enrolled in Pharmachild at the time of preliminary analysis, with 27% of total safety events reported as infections. 360 events were submitted to the Safety Adjudication Committee. 94 infections (26.1%) achieved maximum consensus among the experts (6/6 questions), 237 (65.8%) consensus on 4/6 or 5/6 questions, the remaining events were discarded. 330 infections were finally retained for the analysis, mostly represented by serious infections of moderate/severe intensity. With regard to the other questions, 74.2% infections were adjudicated as common, appropriately treated, with no clear correlation to the immunosuppressive therapy. The experts achieved consensus on 18 infections adjudicated as opportunistic. Referring to the list of opportunistic infections provided by Winthrop *et al.*[1], we identified 89/330 (27%) opportunistic infections, mostly represented by EBV and herpes zoster infections. 98.8% of events were not recoded. A doubled risk of infections was found in the group of patients who were started on their first biologic ± MTX or who had been treated with at least 2 sequential biologics ± MTX (1.3 and 1.9 infectious events per 100 py, respectively), compared to the MTX only group (0.8 infectious events per 100 py). The same was found for serious and opportunistic infections.

**Conclusions:** Our preliminary analysis showed a significant number of infections in JIA patients on immunosuppressive therapy, with one third classified as opportunistic infection. The introduction of biologics in therapy increased the risk of opportunistic and serious infection.

**Disclosure of Interest**

None Declared

### O33 The influence of early achievement of clinically inactive disease on long-term disability outcomes in juvenile idiopathic arthritis

#### Stephanie J. W. Shoop^1,2^, Suzanne M. M. Verstappen^1^, Janet E. McDonagh^3^, Wendy Thomson^2,4^, Kimme L. Hyrich^1,2^, and CAPS

##### ^1^Arthritis Research UK Centre for Epidemiology, The University of Manchester, Manchester, United Kingdom; ^2^NIHR Manchester Musculoskeletal Biomedical Research Unit, Central Manchester University Hospitals NHS Foundation Trust and University of Manchester Partnership, Manchester, United Kingdom; ^3^Centre for MSK Research, Faculty of Medical and Human Sciences, Manchester, United Kingdom; ^4^Arthritis Research UK Centre for Genetics and Genomics, The University of Manchester, Manchester, United Kingdom

###### **Presenting author:** Stephanie J. W. Shoop

**Introduction:** Published definitions for clinically inactive disease (CID) in juvenile idiopathic arthritis (JIA) have recently been shown to identify different groups of children. Wallace’s preliminary criteria captures children with no objective inflammatory signs, whereas the clinical Juvenile Arthritis Disease Activity Score (cJADAS) cut-off identifies children with little objective inflammation and, since a parent global assessment is included, little chronic pain and fatigue. It is unclear whether long-term outcomes, such as functional ability, differ between those who, early in disease, achieve inactive inflammatory disease but have ongoing subjective symptomatology versus those who achieve CID on both aspects.

**Objectives:** To assess whether five-year functional outcome using the Childhood Health Assessment Questionnaire (CHAQ) differs according to disease activity state at one year following JIA diagnosis.

**Methods:** Children were recruited from the Childhood Arthritis Prospective Study (CAPS), a multicentre UK inception cohort. Children with a physician’s diagnosis of JIA (limited to oligoarticular and polyarticular subtypes) and recruitment prior to 1^st^ January 2011 were selected.

Children were categorised into three disease states at one year following presentation: i) CID on both tools, ii) CID on Wallace’s preliminary criteria only and iii) No CID. Mean CHAQ score and percent of children with CHAQ ≤0.5 were assessed at five years and compared descriptively between each disease state group. Multiple imputation accounted for missing data.

**Results:** Of 832 children included, 70% were female and median symptom duration at diagnosis was five months (IQR 3 to 11 months). The most common subtype was oligoarticular (68%), followed by 27% RF-negative (27%) and RF-positive polyarticular JIA (5%). At one year, 22% had achieved CID according to both Wallace’s preliminary criteria and cJADAS, 7% according to only Wallace’s preliminary criteria and 56% to neither.

Compared to those who had not achieved CID, children who had achieved CID on both tools at one year showed a trend to improved CHAQ scores at five years. However, those who had achieved CID only according to Wallace’s preliminary criteria developed similar CHAQ scores to the no CID group (Table 8). This difference may have been driven by the parental global assessment, with mean one year scores of 0.4 cm for the children in ID on both tools, but a similar scores for children in CID only according to Wallace’s preliminary criteria and those not in CID, (2.8 cm and 2.5 cm respectively).

**Conclusion:** Absence of clinically evident inflammation in JIA does not in itself predict better long-term functional ability. Better long-term functional ability is only observed to increase when both objective measures of inflammation and subjective measures of the global picture of disease are taken into account. Children in CID according to Wallace’s preliminary criteria but not the cJADAS may require alternative interventions to manage their ongoing non-inflammatory symptoms.

**Disclosure of Interest**

None Declared

**Table 8 (abstract O33).** Functional ability at five years in children who achieved different disease states at one year following diagnosis

CID state at one year following diagnosis	Mean CHAQ at five years following diagnosis (95% CI)	Percent with CHAQ ≤ 0.5 at five years following diagnosis (95% CI)
CID on Wallace’s preliminary criteria and cJADAS	0.7 (0.5, 0.8)	51 (40, 63)
CID only on Wallace’s preliminary criteria	1.0 (0.8, 1.3)	33 (16, 50)
No CID	0.9 (0.8, 1.0)	37 (29, 44)

### O34 Cost-effectiveness of first year treatment for juvenile idiopathic arthritis (JIA) in the era of biosimilars

#### Maarit Tarkiainen^1,2^, Pirjo Tynjala^3^, Pekka Lahdenne^4^, Janne Martikainen^5^, and Acute-JIA Study Group

##### ^1^Institute of Clinical Medicine, University of Helsinki, Helsinki, Finland; ^2^Children’s Hospital, Helsinki, Finland; ^3^Poison Centre, Helsinki, Finland; ^4^Helsinki University Central Hospital, Helsinki, Finland; ^5^University of Eastern Finland, Kuopio, Finland

###### **Presenting author:** Maarit Tarkiainen

**Introduction:** Biologics are costly, but effective in JIA. Little is known of the cost-effectiveness of non-biologic treatment of JIA.

**Objectives:** We aimed to compare the cost-effectiveness of three treatment strategies of JIA during the first year after onset.

**Methods:** In Acute-JIA study^1^, 60 patients were randomized to receive either infliximab (IFX) plus methotrexate (MTX); combination (COMBO) of MTX, hydroxychloroquine, and sulphasalazine; and MTX alone. Efficacy was measured at eight study visits, and data on both health care and non-health care costs were collected. Costs were assessed at 2012 level, despite for biologics, which were assessed at 2016 level. Societal perspective was used. Health utility was produced from CHAQ using the NICE quadratic mapping algoritm.

**Results:** COMBO seemed more cost-effective over aTNF and MTX. (Table 9). Cost per additional months spent in inactive disease state when using prices for original molecules were 729, 446, and 1429 for aTNF, COMBO, and MTX, respectively. When using the prices for biosimilars, this cost was 543, 436, and 1372, respectively.

**Conclusion:** COMBO seemed a dominant treatment for JIA, over MTX and aTNF.

**Disclosure of Interest**

None Declared

**Table 9 (abstract O34).** Incremental cost-effectiveness ratio (ICER) using prices of biosimilars in treatment of JIA

Treatment	Incremental Costs, €	Incremental QALYs	ICER estimate €/Qaly
COMBO vs MTX	-2918	0.072	dominant^a^
aTNF vs combo	7627	0.023	331 609
aTNF vs MTX	4926	0.095	51 853

*QALY* Quality-Adjusted Life-Year

^a^less costly and more effective treatment option

### O35 Peripheral blood B cells are expanded and their cytokine expression is dysregulated in juvenile dermatomyositis

#### Meredyth Wilkinson^1^, Christopher Piper^2^, Georg Otto^3^, Claire T. Deakin^3^, Stefanie Dowle^3^, Stefania Simou^3^, Daniel Kelberman^3^, Yiannis Ioannou^1^, Claudia Mauri^2^, Elizabeth Jury^2^, David Isenberg^2^, Lucy R. Wedderburn^3^, Kiran Nistala^3^

##### ^1^Centre for Adolescent Rheumatology, UCL, London, United Kingdom; ^2^Centre of Rheumatology, UCL, London, United Kingdom; ^3^UCL Institute of Child Health, UCL, London, United Kingdom

###### **Presenting author:** Meredyth Wilkinson

**Introduction:** Juvenile dermatomyositis (JDM) is a rare form of childhood autoimmune myositis that presents with proximal muscle weakness and heliotrope rash. B cells are strongly implicated in the pathogenesis of the disease and are responsible for the generation of myositis specific antibodies, that are closely associated with specific clinical subgroups. B cells have been detected in inflamed muscle suggesting another pathological function. We propose that B cells may also contribute to the disease by producing pro-inflammatory cytokines, triggered by type I interferons.

**Objectives:** To characterise B cell subsets and B cell production of interleukin 6 (IL-6) and interleukin 10 (IL-10) in JDM patients at presentation and after treatment with corticosteroids and methotrexate.

**Methods:** Samples and data were collected from children with JDM recruited to the UK JDM Cohort and Biomarker Study. Peripheral blood mononuclear cells (PBMC) samples from JDM patients before treatment and on treatment were selected for analysis. PBMC from child healthy controls (CHC) (n = 25) and JDM patients (n = 61) were incubated in the presence of phorbol 12-myristate 13-acetate (PMA), Ionomycin and Golgi Plug for 4 hrs and cytokine expression was analysed by flow cytometry. PBMC and sorted B cells were also stimulated for 72 hr with CD40L or 48 hr with R848 (TLR7/8) and assessed for intracellular IL-6 and IL-10 by flow cytometry and protein levels were measured by ELISA. PBMC were also stained *ex-vivo* to detect B cell subsets and Ki67 expression. Differences between groups were analysed using paired/unpaired t tests or one-way ANOVA (Prism 6 software).

**Results:** The B cell frequency was significantly higher in pre-treatment JDM patient compared to CHC samples (25.72% of total B cells Vs. 22.69% (ns) (<6 months treatment) or 16.26% in CHC; p < 0.01). This difference was most marked in the immature B cell subset (36.05% of total B cells Vs. 3.66% (<6 months on treatment) or 12.4% in CHC; p < 0.01). The immature B cell subset was more proliferative before treatment (mean Ki-67% of 17.63% Vs. 8.86% (>6 months on treatment) or 7.18% in CHC; p < 0.01). This difference was specific to the immature B cell subset. Whole transcriptome RNA sequencing of B cells from paired pre and on treatment patient samples showed an upregulation of the type I interferon signature. Of particular interest Toll-like receptor 7 (TLR7) which was up-regulated in B cells from patients’ pre-treatment. When B cells were stimulated with TLR7 agonist (R848) there was reduced expression of IL-10 detected by ELISA from JDM compared to CHC samples (10.61 pg/ml Vs. 75.01 pg/ml). However, IL-6 expression responded the same in JDM compared to CHC (486.18 pg/ml Vs. 414.29 pg/ml). When B cells were stimulated with CD40L there was equivalent expression of IL-10 from JDM pre-treatment and CHC samples (25.49% of total B cells Vs. 13.26% (<6 months on treatment)(p < 0.01) or 20.07% in CHC).

**Conclusion:** We have identified a subset of immature B cells that are expanded, more proliferative and express both IL-6 and IL-10 in pre-treatment patients. The up-regulated type I interferon signature in JDM could contribute to the expansion of immature B cells with an imbalance of IL-6 and IL-10 production.

**Disclosure of Interest**

None Declared

### O36 Uveitis associated to polyarticular juvenile idiopathic arthritis (PJIA): data from strive registry

#### I Foeldvari^1^, N Ruperto^1^, DJ Lovell^2^, G Horneff^1^, H-I Huppertz^1^, P Quartier^3^, G Simonini^1^, M Bereswill^4^, J Kalabic^4^, A Martini^1^, HI Brunner^2^

##### ^1^PRINTO-IRCCS, Genova, Italy; ^2^PRCSG, Cincinnati, OH, USA; ^3^Hôpital Necker-Enfants Malades, Paris, France; ^4^AbbVie Deutschland GmbH & Co. KG, Ludwigshafen, Germany

###### **Presenting author:** I Foeldvari

**Introduction:** Juvenile idiopathic arthritis (JIA) is one of the most common of all systemic diseases associated with childhood uveitis. Approximately 10–15% of JIA patients (pts) experience comorbid uveitis^1^.

**Objectives:** To explore events of uveitis and associated safety in pts with moderately to severely active polyarticular or polyarticular-course JIA (pJIA) who were prescribed and treated with adalimumab (ADA) and/or methotrexate (MTX) in routine clinical practice.

**Methods:** STRIVE is an ongoing, multicenter, non-interventional, observational registry of up to 10 years duration in pts with moderately to severely active pJIA who are treated with either ADA ± MTX or MTX alone as part of their routine clinical care. Pts could initiate ADA and/or MTX within 24 months prior to registry entry. Pts that completed ADA studies (DE038, M10-444) had an option to roll-over into this registry. Ophthalmologists performed slit-lamp examination for uveitis at registry entry and specified visits in 3-6 month intervals through 5 yrs. Beyond 5 yrs, uveitis events were collected solely through adverse event (AE) reporting. Observational ocular AEs (e.g. cataract, glaucoma) were recorded from registry entry through yr 6.

**Results:** As of 1 June 2015, a total of 21/303 (6.9%) and 68/543 (12.5%) enrolled pts reported at least 1 case of JIA-associated uveitis at any visit in the MTX and ADA ± MTX groups, respectively. In the JIA-associated uveitis population, 10 (47.6%) in the MTX and 42 (61.8%) in the ADA ± MTX group presented with documented uveitis at registry entry. In the population without uveitis at registry entry, 11/293 (3.8%) pts and 26/501 (5.2%) pts in MTX and ADA ± MTX arms, respectively had first documentation of uveitis post-enrollment. Most pts in the JIA-associated uveitis sub-population were female (73%), white (96%), with a mean age of 8.1 yrs; mean pJIA disease duration was 1.8 and 4.8 yrs for the MTX and ADA ± MTX groups at registry entry, respectively. Nine (42.9%) MTX and 48 ADA ± MTX (72.7%) pts were positive for antinuclear antibodies at registry enrollment. Uveitis in the vast majority of pts was localized to the anterior layer. In the ADA group, 45 (66.2%) pts with documented uveitis received concomitant MTX during the course of the registry. Through Month 42, the majority of the JIA-associated uveitis sub-population had either no new manifestation of uveitis or stabilized uveitis. A higher proportion of MTX vs ADA ± MTX pts discontinued the registry drug (15/21 [71.4%] vs. 21/68 [30.9%]), but continued to be monitored for safety follow-up. Of these, 2 (9.5%) and 1 (1.5%) in the MTX and ADA ± MTX group, respectively, discontinued the registry drug due to an AE, and 4 of the 15 pts in the MTX group discontinued MTX group and switched to the ADA ± MTX registry group. Two (0.4%) pts with glaucoma and 1 (0.2%) pt with cataract were reported in the ADA ± MTX group and none in the MTX group; 2 of these patients had documented uveitis at registry enrollment.

**Conclusion:** Among pJIA pts with uveitis documented at registry entry, a higher percentage of pts were enrolled in the ADA ± MTX group as per investigator judgment. No new safety signals for adalimumab were observed in the JIA-associated uveitis sub-population treated per standard of care. Based on this interim analysis, JIA-associated uveitis appeared well-controlled during the course of this registry for pJIA pts with existing uveitis.

**Trial registration identifying number:** NCT00783510

**Reference**

1) Rabinovich CE, 2007; 19(5): 482–486.

**Disclosure of Interest**

I. Foeldvari Consultant for: AbbVie and Novartis, N. Ruperto Grant / Research Support from: AbbVie Inc., AstraZeneca, Bristol-Myers Squibb, Janssen Biologics B.V., Eli Lilly and Co., “Francesco Angelini”, GlaxoSmithKline, Italfgroupaco, Novartis, Pfizer, Roche, Sanofi Aventis, Schwarz Biosciences GmbH, Xoma, and Wyeth Phgroupaceuticals., Employee of: GASLINI Hospital, Speaker Bureau of: Astellas, AstraZeneca, Bristol-Myers Squibb, Italfarmaco, Janssen Biologics B.V., MedImmune, Roche, and Wyeth/Pfizer, D. Lovell Consultant for: AbbVie Inc., AstraZeneca, Centocor, Bristol-Myers Squibb, Boehringer-Ingelheim, Pfizer, Regeneron, Hoffman La-Roche, Novartis, UCB, and Genentech, G. Horneff Grant / Research Support from: AbbVie, Pfizer, and Roche, Speaker Bureau of: AbbVie, Novartis, Pfizer, and Roche, H.-I. Huppertz Consultant for: Wyeth Pharmaceuticals, Essex Pharmaceuticals, and Abbott Laboratories, Speaker Bureau of: Wyeth Pharmaceuticals, Essex Pharmaceuticals, and Abbott Laboratories, P. Quartier Consultant for: AbbVie, Novartis, Pfizer, BMS, Chugai-Roche, Medimmune, Servier, and Swedish Orphan Biovitrum, Speaker Bureau of: Member of a data monitoring board for a Sanofi trial., G. Simonini: None Declared, M. Bereswill Shareholder of: AbbVie Inc, Employee of: AbbVie Deutschland GmbH & Co. KG, J. Kalabic Shareholder of: AbbVie Inc, Employee of: AbbVie Deutschland GmbH & Co. KG, A. Martini Grant / Research Support from: Abbott, BMS, Francesco Angelini S.P.A., GSK, Janssen Biotech, Novartis, Pfizer, Roche, Sanofi-Aventis, Schwarz Biosciences GmbH, Consultant for: BMS, Janssen Biotech, GSK, Novartis, Pfizer, Roche, Sanofi-Aventis, Schwarz Biosciences GmbH, Employee of: GASLINI Hospital, Speaker Bureau of: Abbott, AbbVie, Amgen, Baxalta Biosimilars, Biogenidec, BMS, Astellas, Boehringer, Italfarmaco, Janssen, MedImmune, Novartis, NovoNordisk, Pfizer, Sanofi, Roche, Servier, Takeda, UCB Biosciences GmbH, H. Brunner Consultant for: AbbVie Inc., AstraZeneca, Centocor, Bristol-Myers Squibb, Boehringer-Ingelheim, Pfizer, Regeneron, Hoffman La-Roche, Novartis, UCB, and Genentech, Speaker Bureau of: Genentech Pharmaceuticals.

### O37 Changes in quality of life over time in children with juvenile idiopathic arthritis: results from the ReACCh-Out cohort

#### Kiem Oen^1^, Jaime Guzman^2^, Brian M. Feldman^3^, Brenden Dufault^4^, Jennifer Lee^5^, Natalie Shiff^6^, Karen Watanabe Duffy^5^, Lori Tucker^2^, Ciaran Duffy^5^, and ReACCh-Out Investigators

##### ^1^Pediatrics, University of Manitoba, Winnipeg, Canada; ^2^Pediatrics, University of British Columbia, Vancouver, Canada; ^3^Pediatrics, University of Toronto, Toronto, Canada; ^4^Statistics, University of Manitoba, Winnipeg, Canada; ^5^Pediatrics, University of Ottawa, Ottawa, Canada; ^6^Pediatrics, University of Florida, Gainesville, USA

###### **Presenting author:** Jaime Guzman

**Introduction:** Health-related quality of life (HRQOL) measures provide a more holistic view of the impact of health conditions than single outcome measures. Recent studies suggest a dissociation of HRQOL and traditional Juvenile Idiopathic Arthritis (JIA) disease outcomes, with suboptimal HRQOL reported in children whose disease was inactive or minimally active.

**Objectives:** To describe changes in HRQOL from diagnosis in a large prospective inception cohort of children with JIA.

**Methods:** We analyzed data from the Research in Arthritis in Canadian Children Emphasizing Outcomes cohort (ReACCh-Out). Families (the child and a parent) of children newly diagnosed with JIA between January 2005 and December 2010 were asked to complete the Juvenile Arthritis Quality of Life Questionnaire (JAQQ) and the Quality of My Life visual analogue scale (QoML) at 0, 6, 12, 18, 24, 36, 48 and 60 months after enrolment; data reported here was extracted on May 30^th^, 2012. The JAQQ is composed of 72 items in four domains: gross motor function, fine motor function, psychosocial function and systemic symptoms (e.g. stiffness, vomiting, headache, etc.). Each domain was scored using the 5 most affected items. The total score is the mean of the 4 domains, from1 = best to 7 = worst. The QoML scale varies from 0 = worst to 10 = best HRQOL. We analyzed median values over time and the time required for reaching the best possible score. We compared HRQOL to other JIA outcome measures using Kaplan Meier methods.

**Results:** 1,249 families provided 4,968 HRQOL scores for a median follow-up of 34.2 (IQR 19.2, 48.1) months after enrolment. The initial JAQQ score, measured a median of 0.5 months after diagnosis, was 2.7 (IQR 1.9, 4.0). It varied from 2.4 (IQR 1.6, 3.1) for persistent oligoarthritis to 3.9 (IQR 2.4, 4.7) for RF-positive polyarthritis, respectively (p < 0.0001 by Kruskal Wallis test). JAQQ and QoML scores improved after diagnosis but the improvement was slower and less marked in children with RF-positive polyarthritis. The probability of attaining a JAQQ of 1 within 2 years of diagnosis, varied from 0 for RF-positive polyarthritis to 0.35 for persisting oligoarthritis. For the whole cohort, the median times to attain best scores in HRQOL were substantially delayed relative to other JIA outcomes measures (Table). The JAQQ fine motor domain achieved best scores first and the systemic symptoms domain last.

**Conclusion:** HRQOL is mildly to moderately impaired in children with JIA shortly after diagnosis, and it improves over time at different rates across JIA categories. Achievement of best possible HRQOL scores lagged behind improvements in other outcome measures. This may explain the apparent dissociation of HRQOL from these measures in cross-sectional studies.

**Disclosure of Interest**

None Declared

**Table 10 (abstract O37).** See text for description

	Total JAQQ	Gross motor	Fine motor	Psycho-social	Systemic Symptoms	QoML	Active joints^a^	Physician global^b^	CHAQ^c^
Median score first visit (IQR)	2.7 (2, 4)	3.2 (2, 5)	1.4 (1, 3)	2.4 (1, 3)	3.6 (2, 5)	7.8 (5, 9)	2 (1, 6)	2.8 (1, 5)	0.4 (0.1, 1)
Median months to best score	59.3	21.2	7.2	21.8	40.1	34.5	11.4	14.9	14
Probability of best score within 1 year	0.10	0.32	0.61	0.33	0.15	0.25	0.53	0.38	0.44
Probability of best score within 2 years	0.24	0.52	0.79	0.53	0.32	0.42	0.81	0.68	0.68

Best possible scores were a JAQQ = 1, a QoML = 10

^a^an active joint count = 0

^b^a physician global = 0 in a 100 mm visual analogue scale

^c^a Childhood Health Assessment Questionnaire (CHAQ) = 0

### O38 Subcutaneous abatacept in patients with polyarticular-course juvenile idiopathic arthritis and inadequate response to biologic or non-biologic disease-modifying antirheumatic drugs: pharmacokinetics, efficacy and safety

#### N Ruperto^1^, DJ Lovell^2^, N Tzaribachev^3^, G Vega-Cornejo^4^, I Louw^5^, A Berman^6^, I Calvo^7^, R Cuttica^8^, G Horneff^9^, F Avila-Zapata^10^, J Anton^11^, R Cimaz^12^, E Solau-Gervais^13^, R Joos^14^, G Espada^15^, X Li^16^, M Nys^17^, R Wong^16^, S Banerjee^16^, A Martini^18^, HI Brunner^2^, and For Pediatric Rheumatology International Trials Organization (PRINTO)/Pediatric Rheumatology Collaborative Study Group (PRCSG)

##### ^1^Istituto G. Gaslini Pediatria II Reumatologia, Genova, Italy; ^2^Cincinnati Children’s Hosp. Medical Center, Cincinnati, OH, USA; ^3^Pediatric Rheumatology, Bad Bramstedt, Germany; ^4^CREA (Clinica de Reumatología y Enfermedades Autoinmunes) Hosp. México Americano, Guadalajara, Mexico; ^5^Panorama Medical Centre, Cape Town, South Africa; ^6^Univ. Nacional de Tucuman and Centro Médico Privado de Reumatología, Tucuman, Argentina; ^7^Univ. La Fe, Valencia, Spain; ^8^Hosp. General de Niños Pedro de Elizalde, Buenos Aires, Argentina; ^9^Asklepios Clinic Sankt Augustin, Sankt Augustin, Germany; ^10^Star Medica Hosp., Merida, Mexico; ^11^Hosp. Sant Joan de Déu, Barcelona, Spain; ^12^Ospedale Pediatrico Anna Meyer, Firenze, Italy; ^13^Hôpital de la Milétrie, Poitiers, France; ^14^University Hosp. Gent, Gent, Belgium; ^15^Hosp. De Ninos Dr. Ricardo Gutierrez, Buenos Aires, Argentina; ^16^Bristol-Myers Squibb, Princeton, USA; ^17^Bristol-Myers Squibb, Braine L’Alleud, Belgium; ^18^Istituto G. Gaslini Pediatria II Reumatologia and Univ. of Genova, Genova, Italy

###### **Presenting author:** N Ruperto

**Introduction:** IV abatacept (ABA) 10 mg/kg every 4 weeks is well tolerated and effective in reducing the signs and symptoms of polyarticular-course juvenile idiopathic arthritis (pJIA).^1^ SC ABA 125 mg weekly has equivalent efficacy and comparable safety to IV ABA in adult RA.

**Objectives:** To evaluate SC ABA treatment in patients with active pJIA.

**Methods:** Patients with pJIA and an inadequate response/intolerance to ≥1 DMARD were enrolled in two age cohorts (2–5 years and 6–17 years) in this single-arm, open-label (OL), Phase III study and received SC ABA weekly OL for 4 months based on body weight tier (10– < 25 kg [50 mg ABA]; 25–50 kg [87.5 mg ABA]; >50 kg [125 mg ABA]). JIA-ACR criteria 30 (JIA-ACR30; ACR Pediatric 30) responders at 4 months could enter a 20-month OL extension. Primary endpoint was ABA steady-state blood trough concentration (C_minss_) at 4 months in the 6–17-year cohort.

**Results:** For the 2–5-year (n = 32; interim analysis) and 6–17-year (n = 173; complete analysis) cohorts, respectively: median (min, max) age: 5.0 (2.0, 5.0) and 13.0 (6.0, 17.0) years; number of active joints: 7.0 (2.0, 27.0) and 10.0 (2.0, 42.0). The target therapeutic C_minss_ was achieved in both cohorts (mean [SD] C_minss_ at 4 months: 2–5 years: 50.1 [14.2] μg/mL; 6–17 years: 42.1 [14.7] μg/mL^2^). Robust JIA-ACR30, 70 and inactive disease (no active joints, physician’s global assessment of disease activity <10 mm, CRP <0.6 mg/dL) responses were seen at 4 months in the 2–5-year cohort: 86.7, 70.0 and 51.7%, respectively, and in the 6–17-year cohort:^2^ 80.9, 52.6 and 29.5%, respectively. No new or unexpected safety concerns were reported (Table).

**Conclusion:** Target therapeutic exposure for SC abatacept was achieved in patients with pJIA aged 2–17 years; exposures were within the range observed for IV abatacept in pJIA. Robust efficacy was seen after 4 months of treatment with no new safety concerns.

1. Ruperto N, et al. *Lancet* 2008;**372**:383–91.

2. Ruperto N, et al. EULAR Annual European Congress of Rheumatology 2016: Abstract OP0215.

**Trial registration identifying number:** ClinicalTrials.gov NCT01844518

**Disclosure of Interest**

N. Ruperto Consultant for: AbbVie, Amgen, Alter, AstraZeneca, Baxalta Biosimilars, Biogen, Boehringer, BMS, Celgene, CrescendoBio, EMD Serono, Hoffman-La Roche, Italfarmaco, Janssen, MedImmune, Medac, Novartis, Novo Nordisk, Pfizer, Sanofi Aventis, Servier, Takeda, UCB Biosciences GmbH, Speaker Bureau of: AbbVie, Amgen, Alter, AstraZeneca, Baxalta Biosimilars, Biogen, Boehringer, BMS, Celgene, CrescendoBio, EMD Serono, Hoffman-La Roche, Italfarmaco, Janssen, MedImmune, Medac, Novartis, Novo Nordisk, Pfizer, Sanofi Aventis, Servier, Takeda, UCB Biosciences GmbH, D. Lovell Grant / Research Support from: National Institutes of Health, Consultant for: AstraZeneca, Bristol-Myers Squibb, AbbVie, Pfizer, Roche, Novartis, UBC, Forest Research Institute, Horizon, Johnson & Johnson, Biogen, Takeda, Genentech, GlaxoSmithKline, Boehringer Ingelheim, Celgene, Janssen, N. Tzaribachev Grant / Research Support from: UCB, Pfizer, Janssen, Roche, G. Vega-Cornejo: None Declared, I. Louw: None Declared, A. Berman: None Declared, I. Calvo Grant / Research Support from: Novartis, Speaker Bureau of: Pfizer, AbbVie, Novartis, Sobi, Roche, R. Cuttica: None Declared, G. Horneff Grant / Research Support from: Pfizer, AbbVie, Roche, F. Avila-Zapata: None Declared, J. Anton Grant / Research Support from: Pfizer, Novartis, Speaker Bureau of: Pfizer, AbbVie, Novartis, Sobi, Roche, R. Cimaz: None Declared, E. Solau-Gervais Consultant for: BMS, UCB, Pfizer, AbbVie, Roche, MSD, R. Joos: None Declared, G. Espada: None Declared, X. Li Employee of: Bristol-Myers Squibb, M. Nys Employee of: Bristol-Myers Squibb, R. Wong Employee of: Bristol-Myers Squibb, S. Banerjee Shareholder of: Bristol-Myers Squibb, Employee of: Bristol-Myers Squibb, A. Martini Grant / Research Support from: The Gaslini Hospital, which is the public Hospital where I work as full time public employee, has received contributions from the following industries: Abbott, Bristol-Myers Squibb, “Francesco Angelini”, GlaxoSmithKline, Hoffman-La Roche, Italfarmaco, Janssen, Novartis, Pfizer, Sanofi-Aventis, Schwarz Biosciences, Sobi, Xoma, Wyeth, Consultant for: Abbott, AbbVie, Amgen, Biogen, Bristol-Myers Squibb, Astellas, Boehringer, Italfarmaco, Janssen, MedImmune, Novartis, Novo Nordisk, H. Brunner Consultant for: Pfizer, Bristol-Myers Squibb, UCB, Janssen, Amgen, Celgene, AstraZeneca, Novartis, Genentech, Speaker Bureau of: Novartis, Genentech

**Table 11 (abstract O38).** Summary of AEs during the combined initial 4-month and 20-month extension period (All Treated Patients)

	2–5-year cohort (n = 32)	6–17-year cohort (n = 173)
Deaths	0	0
All AEs	26 (81.3)	127 (73.4)
Related AEs	11 (34.4)	45 (26.0)
AEs leading to discontinuation	0	4 (2.3)^a^
SAEs	0	8 (4.6)
Related SAEs	0	1 (0.6)
SAEs leading to discontinuation	0	2 (1.2)^b^
AEs of special interest		
Malignancies	0	1 (0.6)
Autoimmune disorders	0	3 (1.7)
Local injection-site reactions	0	10 (5.8)
Infections	22 (68.8)	90 (52.0)

Data are n (%)

*SAE* serious adverse event

^a^Exanthema (n = 1) and fatigue (n = 1), both related to study drug (as well as the two SAEs leading to discontinuation)

^b^Sepsis (n = 1), related to study drug; stage III ovarian germ cell teratoma (n = 1), not related to study drug

### O39 Expression of type I and type II interferons is increased in muscle biopsies of juvenile dermatomyositis patients and related to clinical and histological features

#### Rebecca Nicolai^1^, Denise Pires Marafon^1^, Margherita Verardo^2^, Adele D’Amico^2^, Luisa Bracci-Laudiero^1,3^, Fabrizio De Benedetti^1^, Gian Marco Moneta^1^

##### ^1^Division of Rheumatology, Ospedale Pediatrico Bambino Gesù IRCCS, Roma, Italy; ^2^Unit of Neuromuscular and Neurodegenerative Disease, Department of Neuroscience, Ospedale Pediatrico Bambino Gesù IRCCS, Roma, Italy; ^3^Institute of translational Pharmacology, CNR, Roma, Italy

###### **Presenting author:** Rebecca Nicolai

**Introduction:** Juvenile dermatomyositis (JDM) is the most common juvenile inflammatory myopathy, with a still not fully clarified immunopathogenesis. However, there is relevant evidence for an involvement of interferons (IFNs) in the chronic inflammation that characterizes JDM, and a better characterization of their role may provide promising targets for new therapies.

**Objectives:** The aim of this study was to investigate muscle expression of type I (IFNα/β) and type II (IFNγ) IFN inducible genes in muscle biopsies of JDM patients and their correlations with clinical and histological aspects of the disease.

**Methods:** In a retrospective cohort of patients diagnosed with JDM (n = 22), expression of specific genes induced by IFNα/β (IFI27, IFI44L, IFIT1, ISG15, RSAD2, SIGLEC1), the so called “type I IFN signature”, by IFNγ (CXCL9, CXCL10, CXCL11, CIITA), and IFNγ itself, were analysed by real-time PCR on snap frozen muscle biopsies and compared with samples from Duchenne muscular dystrophy (DMD) patients (n = 24). We also analysed mRNA expression of pro-inflammatory cytokines such as interleukin-1β (IL-1β), tumor necrosis factor-α (TNFα) and interleukin-6 (IL-6). For each patient charts were reviewed to record clinical features at diagnosis, physician’s global assessment of the patient’s overall disease activity, serum levels of muscle enzymes (CK, ALT, AST, LDH), erythrocyte sedimentation rate (ESR), C-reactive protein level, antinuclear antibodies status, time to inactive disease, number of immunosuppressants used over disease course and relapses. We also evaluated typical histological aspects of JDM (inflammatory infiltrate, necrosis, perifascicular atrophy and fibrosis) on tissue sections of the muscle biopsies.

**Results:** Since glucocorticoid therapy strongly reduced muscle expression of cytokines, JDM patients treated before biopsy were excluded from analysis. The mRNA expression of type I IFN signature genes (type I-IFN score) was significantly higher in untreated JDM patients (n = 16) compared with DMD patients (*p* <0.0001). Expression of IFNγ and IFNγ related genes (CXCL9, CXCL10, CXCL11, CIITA) were significantly higher in biopsies of untreated JDM patients compared with those of DMD patients (*p* <0.01, *p* <0.01, *p* <0.01, *p* <0.0001, *p* <0.0001, *p* <0.01, respectively). Expression of TNFα, but not of IL-1β and IL-6, was significantly higher in untreated JDM muscles compared with those of DMD patients (*p* <0.01). IFNγ expression significantly correlated with CIITA, CXCL9, CXCL10 and CXCL11 mRNA levels (*p* <0.05, *p* <0.0001, *p* <0.01, *p* <0.01, respectively). Type I-IFN score significantly correlated with ERS, CK, time to inactive disease and number of immunosuppressants (*p* <0.05, *p* <0.05, *p* <0.05, *p* <0.01, respectively). IFNγ mRNA levels significantly correlated with time to inactive disease and relapse after remission (*p* <0.05, *p* <0.01, respectively). We also found that type I-IFN score correlated with inflammatory infiltrate and necrosis (*p* <0.05), while IFNγ correlated with inflammatory infiltrate, perifascicular atrophy and fibrosis (*p* <0.01, *p* <0.05, *p* <0.05 respectively).

**Conclusion:** The increased expression of IFN related genes in muscle biopsies of JDM patients and their association with clinical and histological features suggest a pathogenic role of IFNs in muscle damage and inflammation in JDM. Thus, both type I and type II IFNs pathways may represent therapeutic targets in JDM.

**Disclosure of Interest**

None Declared

### O40 Next generation sequencing reveals complement deficiencies are the most frequent causes of monogenic lupus in children

#### Alexandre Belot^1^, Gillian Rice^2^, Anne-Laure Mathieu^3^, Sulliman O. Omarjee^3^, Brigitte Bader-Meunier^4^, Thierry Walzer^3^, Tracy A. Briggs^2^, James O'Sullivan^2^, Simon Williams^2^, Rolando Cimaz^5^, Eve Smith^6^, Michael W. Beresford^6^, Yanick J. Crow^7^ and GENIAL Investigators, UK JSLE Study Group

##### ^1^INSERM U1111& Pediatric Rheumatology Unit, Lyon, France; ^2^Manchester Academic Health Science Centre, Universtity of Manchester, Manchester, United Kingdom; ^3^INSERM U1111, Lyon, France; ^4^Pediatric Immunology and Rheumatology, IMAGINE Institute, Necker Hospital, Paris, France, ^5^Pediatric Rheumatology, Meyer Children Hospital, Firenze University, Firenze, Italy; ^6^Paediatric Rheumatology, Alder Hey Children's NHS Foundation Trust, Liverpool, United Kingdom; ^7^Neuroinflammation, Institut Imagine & Manchester Academic Health Science Centre, Paris, France

###### **Presenting author:** Alexandre Belot

This abstract is not included here as it has already been published.

## Various topics

### O41 Prevention and treatment of steroid induced osteopaenia in children and adolescents with rheumatic diseases: the pops study

#### Madeleine Rooney^1^, Nick Bishop^2^, joyce davidson^3^, Clarissa pilkington^4^, michael Beresford^5^, Jacqui Clinch^6^, Rangaraj Satyapal^7^, Helen Foster^8^, Janet Gardner Medwin^9^, Janet McDonagh^10^, Sue Wyatt^11^, and On Behalf of the British Society for Paediatric and Adolescent Rheumatology

##### ^1^Experimental Medicine, Queens university Belfast, Belfast, UK; ^2^Academic unit of Child Health, Sheffield University, Sheffield, UK; ^3^medicine, Royal Hospital for Sick Children (Yorkhill) Glasgow, Glasgow, UK; ^4^Great Ormond Street Hospital for Children, NHS Trust, London, UK; ^5^Institute Transnational medicine, University of Liverpool, Liverpool, UK; ^6^Royal National Hospital for Rheumatic Diseases, Bath, UK; ^7^Queen's Medical Centre - Nottingham Childrens Hospital, Nottingham, UK; ^8^Institute of Cellular Medicine, Newcastle University, Newcastle, UK; ^9^Child Health, University of Glasgow, Glasgow, UK; ^10^Institute of Inflammation and Repair, University of Manchester, Manchester, UK; ^11^Rheumatology, Leeds Teaching Hospitals, Leeds, UK

###### **Presenting author:** Madeleine Rooney

**Introduction:** Children with rheumatic diseases have reduced bone density and increased vertebral fracture rate compared to controls and that children on high dose steroids have twice the fracture risk of those on low dose. Despite the widespread use of biologics some 30% of children with JIA for example remain on steroids. There is ample evidence base for the treatment of adults with low bone density (BMD); there is none for children and adolescents.

**Objectives:** To investigate whether the bisphosphonate risedronate was superior to 1-alfacalcidol or placebo, in increasing BMD as a surrogate for reducing fracture risk

**Methods:** Children and adolescents with JIA, JDM, JSLE or vasculitis, commencing or receiving steroids were eligible. Patients were stratified using a minimisation technique according to: disease type; Tanner score: and low /high steroid dose. They were randomised to receive: Group 1. Risedronate 1 mg/kg per week; Group 2 1-alfacalcidol 15 ng/kg/day; Group 3 Placebo. Groups 1,2 and 3 all received nutritional supplements of Calcium 500 mg /day as well as Vit D 400 IU/day. All were followed for 1 year. Clinical and biochemical measurements were performed every 3 months; DXAs every six months; X rays at T0 and one year. Statistical analysis was undertaken using ANOVA. Primary Outcome. Change in lumbar spine BMD Z score (LSBMDz) at one year.

**Results:** At analysis there were 58 in Group 1; 67 in Group 2 and 72 in Group 3. There were no significant differences in SAEs between the groups. There were highly significant differences in the changes in LSBMDz at one year between the three groups; risedronate vs 1-alfacalcidol (difference 0.34; p < 0.001) and risedronate vs Placebo (difference 0.27; P < 0.01). There was no significant difference in LSBMDz between the 1-alfacalcidol and placebo groups (0.07; p = NS).

**Conclusion:** The bisphosphonate risedronate results in significant improvement in LSBMDz at one year and likely associated reduction in vertebral fracture risk. Clinicians should be advised to consider using risedronate in young people with rheumatic diseases on steroids. In contrast 1-alfacalcidol should not be used as it has no impact on improving LSBMDz

**Trial registration identifying number: EuDRACT No: 2005-003129-23**

**ISRCTN66814619**

**Ethics Reference No 06/NIR03/18**

**Disclosure of Interest**

M. Rooney: None Declared, N. Bishop Grant / Research Support from: I received grant and research support from Sanofi Aventis during the time the study was being designed, Consultant for: I received consultant fees from Sanofi-Aventis during the time the study was being designed, J. davidson: None Declared, C. pilkington: None Declared, M. Beresford: None Declared, J. Clinch: None Declared, R. Satyapal: None Declared, H. Foster: None Declared, J. Gardner Medwin: None Declared, J. McDonagh: None Declared, S. Wyatt: None Declared

**Table 12 (abstract O41).** Base line characteristics

Variables	Placebo	One alpha	Risedronate	All patients
N	77	71	69	217
Age (years), Mean (SD)	12.1 (3.5)	12.1 (3.7)	12.0 (3.4)	12.1 (3.5)
Female, n (%)	55 (71.4)	48 (67.6)	53 (76.8)	156 (71.9)
Male, n (%)	22 (28.6)	23 (32.4)	16 (23.2)	61 (28.1)
Tanner Score, Median (IQR)	2 (1 to 4)	2 (1 to 4)	2 (1 to 3)	2 (1 to 4)
Steroid Dose, n (%) <= 0.2 mg/kg	37 (48.1)	30 (42.3)	32 (46.4)	99 (45.6)
Steroid Dose, n (%) >0.2 mg/kg	40 (52.0)	41 (57.8)	37 (53.6)	118 (54.4)
Etanercept; Infliximab; Anakinra; Tocilizumab	8 (10.5%)	17 (23.9%)	7 (10.1%)	32 (14.8%)
Prior fracture History (Yes), n (%)	13 (17.1)	9 (12.7)	8 (11.6)	30 (13.89)

### O42 Reduced joint count for ultrasound assessment in juvenile idiopathic arthritis

#### Valentina Litta Modignani^1^, Francesco Baldo^1^, Stefano Lanni^2^, Alessandro Consolaro^2^, Angelo Ravelli^2^, Giovanni Filocamo^1^

##### ^1^Dipartimento della Donna, del Bambino o del Neonato, Fondazione IRCCS cà Granda Ospedale Maggiore Policlinico, Milano, Italy; ^2^IRCCS G Gaslini and Università di Genova, Genova, Italy

###### **Presenting author:** Valentina Litta Modignani

**Introduction:** Juvenile idiopathic arthritis (JIA) is a heterogeneous group of arthritides with different prevalence and distribution of joint involvement. Ultrasound (US) is a powerful tool for the assessment of joint disease and has been shown to be more accurate than clinical examination in detecting synovitis. However, assessment of a large number of joints is tedious and time consuming in daily practice, making the feasibility of US questionable.

**Objectives:** To evaluate the prevalence of clinically active joints in different JIA categories, to propose a core set for reduced joint assessment for US (rUS), and to provide preliminary evidence of validity of rUS.

**Methods:** The data collected in a large multinational study (the EPOCA study) were analyzed to evaluate the prevalence of active joints in different ILAR categories of JIA. For each patient, the visit with the highest number of active joints was selected. The 10 most frequently affected joints in the different JIA subsets, and the Juvenile Arthritis Disease Activity Score (JADAS) were calculated. A core set of 6, 8 and 10 joints was considered for inclusion in the rUS and the construct validity of the three rUS was assessed by calculating their Spearman’s correlations with the clinical (3-item) JADAS and with the other JIA outcome measures.

**Results:** 8,269 JIA patients seen in pediatric rheumatology centers worldwide were included in the study. The table shows the Spearman’s correlations of the three versions of the rUS with the clinical JADAS, the physician’s global assessment of overall disease activity, and the active joint count.

**Conclusion:** The reduced joint count revealed the ability to serve as surrogate for the whole joint count. The version that includes 6 joints performed similarly to the 8- and 10-joint counts in the assessment of severity of joint disease in all ILAR categories and is proposed as a core set for standard screening in US assessment.

**Disclosure of Interest**

None Declared

**Table 13 (abstract O42).** See text for description

Clinical JADAS							
	Systemic arthritis	RF + polyarthritis	RF - polyarthritis	Oligoarthritis	Psoriatic arthritis	ER A	Undifferentiated arthritis
	N 880	N 346	N 1914	N 3516	N 282	N 871	N 457
rUS 6	0.77	0.82	0.77	0.73	0.70	0.65	0.69
rUS 10	0.79	0.86	0.82	0.77	0.78	0.70	0.76
Physician’s global assessment							
rUS 6	0.74	0.73	0.72	0.75	0.67	0.62	0.69
rUS 10	0.75	0.76	0.76	0.77	0.74	0.65	0.75
Active joint count							
rUS 6	0.95	0.95	0.92	0.92	0.89	0.89	0.89
rUS 10	1	1	1	1	1	1	1

## Poster Session: Autoinflammatory diseases I

### P1 The impact of gastrointestinal clinical manifestations in autoinflammatory diseases (AIDS): lessons from the international Eurofever registry

#### Alessia Omenetti^1^, Joost Frenkel^2^, Helen J. Lachmann^3^, Seza Ozen^4^, Nicolino Ruperto^5^, Marco Gattorno^5^; on behalf of Eurofever Registry

##### ^1^DINOGMI, University of Genoa, Genoa, Italy; ^2^University Medical Center Utrecht, Utrecht, Netherlands; ^3^Royal Free Campus, London, United Kingdom; ^4^Hacettepe University Children’s Hospital, Ankara, Turkey; ^5^Giannina Gaslini Institute, Genoa, Italy

###### **Presenting author:** Alessia Omenetti

**Introduction:** The spectrum of autoinflammatory diseases (AIDs) is expanding and delay in diagnosis may occur if those rare entities are not promptly identified.

**Objectives:** To assess the impact and significance of gastrointestinal (GI) clinical manifestations in a broad spectrum of patients with primary diagnosis of AIDs.

**Methods:** The Eurofever registry containing data retrospectively collected about patients with primary diagnosis of AIDs enrolled by 108 partecipating centres (Nov 2009-Apr 2016) was evaluated. Demografic, genetic and clinical features were analyzed by filtering for GI symptoms including vomiting, abdominal pain, constipation, diarrhoea, GI ulcers, anal/perianal ulcers, GI bleeding and aseptic peritonitis. When present, each manifestation was ranked by its clinical significance and classified as grade 1 or grade 2 (i.e. occasionally or persistently present during flares, respectively). Development of complications such as gut perforation, peritoneal adhesions, occlusions/sub-occlusions or other, if any, were also assessed.

**Results:** Total 3322 patients affected by AIDs including Bechet (n = 208, 6.26%), Blau (n = 49, 1.47%), CANDLE (n = 1, 0.03%), CAPS (n = 263, 7.91%), CRMO (n = 486, 14.62%), DADA2 (n = 2, 0.06%), DIRA (n = 3, 0.09%), FMF (n = 905, 27.24%), Majeed (N = 3, 0.09%) MKD (n = 192, 5.77%), NLRP12 (n = 13, 0.39%), PAPA (n = 28, 0.84%), PFAPA (n = 631, 18.99%), Majeed (n = 3, 0.09%), SAVI (n = 1, 0.03%), TRAPS (n = 261, 7.85%), undefined (n = 276, 8.30%) were evaluated. At least 1 GI manifestation was reported in 1570 patients (47.2%)(M:F = 1:1) including 83 Bechet, 7 Blau, 1 CANDLE, 61 CAPS, 38 CRMO, 1 DADA2, 2 DIRA, 676 FMF, 1 Majeed, 168 MKD, 2 NLRP12, 4 PAPA, 229 PFAPA, 160 TRAPS, and 137 undefined AIDs. Among patients displaying GI manifestations, symptoms were reported as grade 2 in 731 cases (46.5%) and severe GI complications occurred in 107 cases (6.81%). Namely, 20 patients (1.27%) suffered from gut perforation (i.e. 1 CAPS, 7 FMF, 3 MVK, 1 PFAPA, 1 DIRA, 1 CRMO, 3 Bechet, 1 PAPA, 2 undefined), while 49 subjects (3.1%) were affected by peritoneal adhesions (i.e. 3 CAPS, 27 FMF, 2 TRAPS, 6 MVK, 1 PFAPA, 1 DIRA, 1 CRMO, 3 Bechet, 1 PAPA, 4 undefined), and 38 patients (2.4%) developed occlusions/sub-occlusions (i.e. 1 CAPS, 17 FMF, 4 TRAPS, 5 MVK, 1 PFAPA, 1 DIRA, 1 CRMO, 3 Bechet, 1 PAPA, 4 undefined). Finally, secondary diagnosis of GI disease was found in 5 subjects (0.3%) (i.e. gastroduodenitis, IBD, coeliac disease, eosinofilic colitis) and hepatomegaly was reported in 154 patients (9.8%).

**Conclusion:** Although descriptive, these data unveiled the unforeseen significance of GI involvement in AIDs. These findings have threefold implications in the daily clinical management of AIDs. 1) GI specialists should be aware of these rare diseases in order to avoid delayed AID diagnosis in case patients come to their attention first; 2) pediatric rheumatologists and GI specialists should develop standardized work-up protocols in order to monitor these signs and prevent potential complications; 3) these data strongly address the need of a prospective analysis in order to delineate the disease history and potential evolutions (if any) into more defined GI diseases in the contest of autoinflammation.

**Disclosure of Interest**

A. Omenetti: None Declared, J. Frenkel Grant / Research Support from: unrestricted grant from Novartis and SOBI, H. Lachmann Grant / Research Support from: unrestricted grant from Novartis and SOBI, S. Ozen Grant / Research Support from: unrestricted grant from Novartis and SOBI, N. Ruperto Grant / Research Support from: unrestricted grant from Novartis and SOBI, M. Gattorno Grant / Research Support from: unrestricted grant from Novartis and SOBI

### P2 Variable clinical phenotypes and relation of interferon signature with disease activity in ADA 2 deficiency

#### Antonella Insalaco^1^, Gianmarco Moneta^1^, Manuela Pardeo^1^, Chiara Passarelli^2^, Camilla Celani^1^, Virginia Messia^1^, Fabrizio De Benedetti^1^

##### ^1^Division of Rheumatology, Ospedale Pediatrico Bambino Gesù IRCCS, Roma, Italy; ^2^Divion of Medical Genetics, Ospedale Pediatrico Bambino Gesù IRCCS, Roma, Italy

###### **Presenting author:** Antonella Insalaco

**Introduction:** The deficiency of adenosindeaminase 2 (DADA2) is a recently described autosomal recessive autoinflammatory disease, caused by mutations of *CECR1* and characterized by early onset vasculopathy with livedoid skin associated to systemic inflammation. In some patients, the disease is mild and skin-limited, in others is severe, with multi-organ involvement including ischemic or hemorrhagic strokes. In some DADA2 patients a mild immunodeficiency was detected involving adaptive immunity. TNF inhibitors are very efficacious. Recently, an upregulation of type I interferon-stimulated gene transcripts, so called interferon signature, was described also in two DADA2 patients.

**Objectives:** To describe the clinical course of a 4 patients with *CECR1* mutations and to assess the role of interferon type I signature as marker of disease’s activity

**Methods:** Molecular analysis of *CECR1* was performed using next generation sequencing and confirmed by sanger sequencing. Blood was collected into PAXgene tubes and expression levels of IFI-27 IFI-44 L IFIT-1, ISG-15, RSAD-2 and SIGLEC- determined. The IFN score was derived as described [1]. The mean interferon score of the controls plus two SD was calculated: 1.62 and scores higher than this value were considered positive

**Results:** Four Caucasian patients (2 brothers males and 2 unrelated females) were identified carrying *CECR1* mutations (compound eterozygous Leu249Pro/Pro344Leu in the two brothers, compound heterozygosity T360A/R49Gfs*4 deletion in one and homozygous Y453C mutation in one). Mean age was 12.2 ± 2.0 years. Three of the described patients presented with clinical features consistent with the phenotype of DADA2 already reported including recurrent fever, livedo reticularis, persistent elevation of inflammatory markers, arthralgia/ arthritis. Unusual/undescribed manifestation included in 1 patient: early onset gastrointestinal involvement with a biopsy consistent with unspecific inflammation, posterior reversible encephalopathy with seizures, deafness and malignant hypertension. The latter is due to nephrogenic hypertension secondary to kidney infarction. His younger brother showed a very mild phenotype characterized by a single episode of prolonged fever with abdominal pain and arthralgia. He is the 1 patient showing hypogammaglobulinemia. All patients showed a complete normalization of clinical and laboratory findings with no recurrences after treatment with etanercept (follow-up ranging from 8 months to 20 months). The interferon score before treatment was elevated in 3 (ranging from 3.6 to 10.4) out of 4 patients (except for the younger brother), and rapidly normalized after treatment

**Conclusion:** Our data confirm the highly variability of DADA2 regarding age of onset, severity and organ involvement, even within families and, for unknown reasons, among patients with the same mutations. Furthermore, these data suggest that type I interferon score could be used in DADA2 patients as a useful biomarker of disease’s activity. The relation between type I IFN hyperactivity, deficiency of ADA2 and response to TNF inhibition remain elusive

**References**

[1] Rice GI et al . Assessment of interferon-related biomarkers in Aicardi-Goutières syndrome associated with mutations in TREX1, RNASEH2A, RNASEH2B, RNASEH2C, SAMHD1, and ADAR: a case-control study. Lancet Neurol. 2013 Dec;12(12):1159–6

**Disclosure of Interest**

A. Insalaco: None Declared, G. Moneta: None Declared, M. Pardeo: None Declared, C. Passarelli: None Declared, C. Celani: None Declared, V. Messia: None Declared, F. De Benedetti Grant / Research Support from: Novartis, Novimmune, Hoffmann-La Roche, Sobi, Abvie

### P3 Association between familial Mediterranean fever and spondyloarthritis. Clinical characteristics of the French ceremai pediatric cohort

#### Bilade Cherqaoui^1^, Linda Rossi-Semerano^2^, Perrine Dusser^2^, Véronique Hentgen^3^, Isabelle Koné-Paut^2^

##### ^1^Pediatrics, CHU Estaing, Clermont-Ferrand, France; ^2^Pediatric Rheumatology, CEREMAI, CHU Bicêtre, France; ^3^Pediatrics, CEREMAI, CH Versailles, France

###### **Presenting author:** Bilade Cherqaoui

**Introduction:** Spondyloarthritis (SpA) is more frequently observed in familial Mediterranean fever (FMF), and possibly related to *MEFV* mutations in these patients. No studies to date report this association in other region than Middle East, and only few studies describe precisely the pediatric features.

**Objectives:** To estimate the frequency and to describe FMF and juvenile SpA (J-SpA) association in a French pediatric patient’s cohort.

**Methods:** We screened FMF patients seen at Bicêtre hospital, a tertiary referral center. FMF was defined according to either Tel-Hashomer or Turkish pediatric criteria, with at least 1 exon 10 mutation in *MEFV* gene. J-SpA was defined according to ILAR criteria of enthesitis-related/psoriatic juvenile arthritis; we also assessed the performance of ASAS criteria. FMF patients with SpA (FMF-SpA) were then compared to those with typical FMF and to 20 patients with J-SpA.

**Results:** Among 125 screened files, 99 fulfilled criteria of FMF. Among FMF patients, 16 fulfilled criteria of J-SpA (16%). The demographic and genetic features of FMF-SpA patients were: 1.6 girl/1 boy, median age 7.5 years, frequency of homozygous and heterozygous M694V mutation in *MEFV* gene 50% and 31.2% respectively, and frequency of HLA-B27 17%. SpA symptoms started mostly with peripheral arthritis (87.5%), unilateral (47%), involving the lower limbs (16.6% in upper limbs); it evolved with enthesitis (50%) and inflammatory back pain (56.3%). Extra-articular manifestations (psoriasis, inflammatory bowel disease) were observed in 18.7%. Axial imaging was mostly not contributive; but enthesitis imaging revealed ultrasound or MRI abnormalities in 44.4%. Two patients were efficiently treated with anti-TNF drugs (etanercept, adalimumab). When comparing the 16 FMF-SpA patients with the 83 typical FMF patients, we observed: higher frequency of FMF and psoriasis in family (OR = 8, p = 0.04 and 9.3, 0.02 respectively), lower frequency of fever (OR = 0.1, p = 0.008), higher frequency of crisis with arthralgia/arthritis (OR = 23, p = 0.03 and 6.1, 0.02 respectively), higher frequency of talalgia/plantalgia (OR = 34.3 and 44.4, p < 0.001) in FMF-SpA group. No significant biological or *MEFV* genotypes differences were observed. FMF-SpA patients received more frequently anti-IL1 drugs after failure of colchicine (OR = 4.2, p = 0.04). Articular features seemed to respond at least partially to anti-IL1 drugs. When comparing the 16 FMF-SpA patients with 20 typical J-SpA patients, we observed: lower age at onset of articular signs (4.5+/-1.1 vs 9+/-0.6 years respectively, p = 0.001), higher frequency of plantar pain (OR = 31.6, p < 0.001), less frequent synovitis on ultra-sound (p = 0.03) in FMF-SpA group. Non-steroidal anti-inflammatory and anti-TNF drugs were less frequently used in FMF-SpA patients (p = 0.008, <0.001 respectively). ILAR criteria were more sensitive and less specific than ASAS’s in FMF-SpA and J-SpA groups (92.3/57.1% and 100/60% respectively).

**Conclusion:** A non-incidental association of FMF and J-SPA was observed in this French cohort, in accordance with data observed in Middle East (Barut K, *et al.* Articular involvement in childhood FMF. *Pediatr Rheumatol* 2015). Frequency of this association reached 16%, a bit higher than previously defined. Compared to typical FMF, SpA-FMF patients present a predominant articular/enthesitis pattern, less febrile crises, and more frequent resistance to colchicine. Except earlier age at onset, more frequent plantar involvement and less frequent ultra-sound synovitis, SpA-FMF features were virtually similar to J-SpA’s. Thus SpA-FMF related arthritis should be followed and treated as J-SpA, including anti-TNF drug use.

**Disclosure of Interest**

None Declared

### P4 Health-related quality of life (HRQOL) of children with PFAPA syndrome

#### Claire Grimwood^1^, Perrine Dusser^2^, Linda Rossi^2^, Isabelle Kone Paut^2^, Veronique Hentgen^1^

##### ^1^French reference centre for Autoinflammatory diseases, pediatric unit, Versailles Hospital, Le Chesnay, France; ^2^French reference centre for Autoinflammatory diseases, pediatric unit, CHU Kremlin Bicetre, Kremlin Bicetre, France

###### **Presenting author:** Claire Grimwood

**Introduction:** PFAPA syndrome (Periodic Fever, Aphtous stomatis, Pharyngitis and Adenitis) is generally considered to be a benign disease compared to other autoinflammatory syndromes because it normally disappears before adulthood. However fever episodes may have a huge impact on daily activities, schooling and family functioning.

**Objectives:** To describe and compare the physical and psychosocial HRQOL of children with PFAPA compared to FMF peers using a multidimensional, well validated, and reliable HRQOL instrument.

**Methods:** Thirty three voluntary PFAPA patients attending the French reference centre for autoinflammatory diseases (CeRéMAI) during the year 2015 and having an active disease were included in the study. The control group consisted of 27 FMF patients age matched, attending the autoinflammatory clinic during the same period. All the subjects and/or their parents of the study were asked to complete the age appropriate questionnaire of the Pediatric Quality of Life Inventory TM 4.0 (PedsQL™ 4.0) Generic Core Scale. Patients and controls were grouped according to their ages as follows: pre-school age children (2-7 years) and school-age children and youth (8–18 years).

**Results:** PedsQL™ self-report scores of pre-school age children (2–7 years) with PFAPA were significantly lower than FMF peers for general quality of life, physical and psychosocial functioning (Table). The parent proxy-report did not find a significant difference even though scores were systematically lower in PFAPA patients. The parent proxy-report and child self-reported PedsQL™ scores of school age children and adolescent patients (8–18 years) with PFAPA were lower than the FMF group for general quality of life, physical and psychosocial functioning; however the difference was not significant.

**Conclusion:** HRQOL in PFAPA children seems to be lower than in FMF peers while the latter are known to have impaired QOL if compared to the general population.

**Disclosure of Interest**

None Declared

**Table 14 (abstract P4).** Parent proxy-reported and child self-reported PedsQL™ scores of pediatric patients with

Scale	PFAPA patients		FMF controls		P
	Number	Mean	Number	Mean	
Pre-school age children (2–7 years)					
Self report : Total score	15	66,6	8	80,3	0,01
Proxi-report : Total score	23	68,9	14	76,9	0,12
School age children and youth (8–18 years)					
Self report : Total score	8	58	13	74	0,06
Proxi-report : Total score	10	57,4	13	72,3	0,08

### P5 Cryopyrin associated periodic syndromes in Italian patients: evaluation of the rate of NLRP3 mosaicism and search for novel genes

#### Denise Lasigliè^1^, Denise Ferrera^1^, Giulia Amico^1^, Marco Di Duca^1^, Roberta Caorsi^1^, Loredana Lepore^2^, Antonella Insalaco^3^, Marco Cattalini^4^, Laura Obici^5^, Rita Consolini^6^, Roberto Ravazzolo^1^, Alberto Martini^1^, Isabella Ceccherini^1^, Ryuta Nishikomori^7^, Juan Arostegui^8^, Marco Gattorno^1^, Silvia Borghini^1^

##### ^1^IRCCS G. Gaslini, Genova, Italy; ^2^IRCCS Burlo Garofalo, Trieste, Italy; ^3^IRCCS Bambino Gesu’, Roma, Italy; ^4^Spedali Civili, Brescia, Italy; ^5^IRCCS San Matteo, Pavia, Italy; ^6^Università di Pisa, Pisa, Italy; ^7^Universita’ di Kyoto, Kyoto, Japan; ^8^Ospedale di Barcelona, Barcelona, Spain

###### **Presenting author:** Denise Lasigliè

**Introduction:** Most patients affected with Cryopyrin associated periodic syndromes (CAPS) bear a heterozygous mutation in NLRP3 gene, a little proportion of which in mosaic status at level not evaluable by Sanger sequencing. Nevertheless, a proportion of patients with a clinical phenotype of CAPS, particularly Muckle Wells Syndrome and Familial Cold Autoinflammatory Syndrome, remain without a genetic diagnosis.

**Objectives:** (1) Evaluation of the rate of NLRP3 mosaicism in Italian patients previously described as mutation negative (2) Identification of novel genes responsible for CAPS symptoms (3) Providing patients with a correct genetic diagnosis and a risk recurrence

**Methods:** Patients with a clinical and immunological phenotype resembling CAPS phenotypes and resulted negative to the screening of the total coding sequence of NLRP3 gene by Sanger screening have been enrolled in this study. Patients DNA have been subjected to (i) Next generation sequencing at high coverage of NLRP3 gene to identify mutations in mosaic status and, in negative cases, to (ii) whole exome sequencing (WES) for novel genes discovery.

**Results:** Massively parallel DNA sequencing revealed somatic NLRP3 mosaicism in four patients: three affected with CINCA (100%) and one with MWS (12,5%). Identified nucleotide substitutions are all located in the exon three of NLRP3 and encode for four different aminoacid changes, two of them being novel (p.G564S and p.Y563C) and remaining already reported (p.R260P as full mutation and p.T433I in mosaic state). None of the detected variants was reported in about 63000 genomes at Exac Browser Consortium. Compared to wild type NLRP3, mutant proteins rapidly induce necrotic cell death when transiently transfected in human monocytic THP-1 cells and cause enhanced NF-kB reporter activity after co-expression with its partner ASC in HEK293FT. In almost all cases, mutated alleles have been identified not only in all the cell population from the blood but also in ectodermal tissues, suggesting that the event has been established very early in development.

DNA from the remaining patients have been subjected to WES in search of novel candidate genes. Unfortunately, same SNPs or different variants at the same genes have not been identified in at least two different patients. Subsequent analysis has been conducted considering single family or trio. A *de novo* mutation has been identified in a transcription factor belonging to the FOX gene family. Preliminary functional tests confirm a causative role of this amino acid substitution. In a family, the disease segregates with a novel variant in a protein sensor of viral RNA. Finally, in a patient an immune receptor displays impaired membrane expression in white blood cells, due to the coupling of an allele with low frequency from the mother and a non-functional one from the father.

**Conclusion:** CAPS are widely associated with mutations in the NLRP3 gene, being mutated in mosaic status in the most severe patients, and only a small percentage to private mutations that arise in individual patients. Although these changes have apparently a very rare frequency, only an analysis of the genes identified in this study in more patients with CAPS resembling symptoms can definitively assess their actual impact.

**Disclosure of Interest**

None Declared

### P6 Monocytes proteomic profile of patients with different autoinflammatory diseases: an approach to identify new biomarkers

#### Federica Penco^1^, Andrea Petretto^2^, Chiara Lavarello^2^, Elvira Inglese^2^, Alessia Omenetti^3^, Martina Finetti^3^, Claudia Pastorino^1^, Arinna Bertoni^1^, Marco Gattorno^1^

##### ^1^Laboratorio di Immunologia delle Malattie Reumatiche, Genova, Italy; ^2^Core Facilities-Laboratorio Proteomica, Genova, Italy; ^3^Pediatria II, Istituto G. Gaslini, Genova, Italy

###### **Presenting author:** Federica Penco

**Introduction:** Autoinflammatory diseases are a group of inherited diseases characterized by early onset and systemic inflammation, often manifesting with unexplained fevers. These pathologies are usually caused by mutations in genes involved in the regulation of innate immune response with consequent inflammatory phenotype driven by activation of monocytes, macrophages and granulocytes. Part of this pathologies are however genetically undefined.

**Objectives:** Our aim is to evaluate the differences in the expression of proteins or pathway in monocytes, untreated or treated with LPS, of patients with autoinflammatory diseases and healthy subjects in order to clusterize the different diseases and better characterize the genetically undefined pathologies.

**Methods:** Monocytes, purified form peripheral blood of patients and healthy subjects with CD14 micrebeads positive selection, were collected and incubated for 4 hours with or without LPS stimulation. Cells were lysed and prepared to be analyzed at MS. The samples were processed by iST protocol and protein expression evaluated by an High Resolution/Mass Accuracy Liquid Chromatography Tandem Mass Spectrometry (HR/MA LC MS/MS). Each sample was run at least in triplicate. PCA analysis and Person’s correlation were used as quality control of experimental design, while the statistical analysis was performed with the Perseus software.

**Results:** We analyze the monocytes of patients with CAPS, TRAPS and FMF (each group compared with healthy subjects). We have identified about 4000 proteins of each 3500 are quantified by LFQ approach. PCA analysis and Person’s correlation show a good reproducibility of data and a good separation between the different groups. In synchronous way using a cluster analysis and heatmap, based on a machine learning protein selection, we observe a protein signature specific for each group of pathology and for each condition of monocytes treatment.

**Conclusion:** Here, we addressed how an high resolution proteomics approach could be used to better understand the biology of autoinflammatory diseases. The characterization of a broad spectrum of proteins and their interaction network will allow us to identify new biomarkers for the different pathologies and to better comprehend and recognize the genetically undefined disorders.

**Disclosure of Interest**

None Declared

### P7 Results of the Eurofever Delphi survey for the classification criteria of PFAPA syndrome

#### Federica Vanoni^1,2^, Silvia Federici^3^, Seza Ozen^4^, Joost Frenkel^5^, Helen Lachmann^6^, Alberto Martini^3^, Nicola Ruperto^3^, Marco Gattorno^3^, Michaël Hofer^1^, and on behalf of EUROFEVER PROJECT

##### ^1^Pediatric Rheumatology Unit, CHUV, University of Lausanne, Lausanne, Switzerland; ^2^Pediatrics, ORBV - Bellinzona, Bellinzona, Switzerland; ^3^Pediatria II - Reumatologia, Istituto G. Gaslini, Genoa, Italy; ^4^Department of Pediatric Nephrology and Rheumatology, Hacettepe University, Ankara, Turkey; ^5^Department of Paediatrics, UMC Utrecht, Utrecht, Netherlands; ^6^National Amyloidosis Centre, University College Medical School, London, United Kingdom

###### **Presenting author:** Federica Vanoni

**Introduction:** Diagnosis of Periodic fever, aphthous stomatitis, pharyngitis and cervical adenitis (PFAPA) is currently based on a set of criteria proposed in 1999 modified from Marshall’s criteria. Nevertheless no validated evidence based set of classification criteria for PFAPA has been established so far.

**Objectives:** To identify evidence based candidate classification criteria for PFAPA syndrome using the consensus formation techniques (Delphi and Nominal Group Techniques).

**Methods:** Two following Delphi surveys were sent to health professionals working in the field of autoinflammation. In the first open survey 124 experts belonging to the PRINTO Network were asked to list as many variables they wanted they consider as important for the diagnosis of patients with PFAPA. The variables may be of any type (clinical, laboratory, instrumental variables, genetic tests etc). In the second survey 162 experts (also American experts were involved in this phase) were asked to select, from a list of items coming from the first survey, the 10 top variables and to rank them by assigning a score from 10 to 1 in order of importance. There was no possibility to assign the same rank to multiple variables. This process has been conducted in parallel with an analogous process for the monogenic periodic fevers.

**Results:** The overall rate of response to the first and second Delphi, including monogenic periodic fevers, was respectively 86% (107/124 experts) and 87%(141/162 experts). For PFAPA, the rate of response to the first and second Delphi was respectively 55% (68/124 experts) and 70% (101/162 experts). We obtained 92 variables from the first survey among which 62 were selected in the second survey. Table 15 shows the total score obtained and the rate of selection of the top variables (3^rd^ quartile).

**Conclusion:** Our process led to the identification of those features that are considered to be the most important as candidate variable to be included in a new set of evidence based classification criteria for PFAPA. The next step of this project (now ongoing) is the evaluation by a panel of experts of 360 real patients affected by AIDs (PFAPA, monogenic periodic fever and undefined periodic fever) randomly selected from EUROFEVER Registry. Those patients reaching the 80% of consensus among experts in the evaluation process will be used for the subsequent statistical analysis aimed at identified the best evidence-based classification criteria (in term of sensitivity and specificity) for PFAPA. The process will end with a consensus conference that will be held in Genoa on March 16-18, 2017.

**Disclosure of Interest**

None Declared

**Table 15 (abstract P7).** See text for description

Rank	Variable	Global Score	Votes (n)
1	Regular periodicity	436	56
2	Aphthous stomatitis	431	77
3	Response to steroid	401	66
4	Cervical adenitis	368	72
5	Well being between flares	299	57
6	Pharyngitis (exudative or not)	288	47
7	Increase of acute phase reactants and serum amyloid A during fever episodes	271	44
8	Normal growth/development	236	51
9	Pharingotonsillitis	228	35
10	Periodic fever 3–5 days	202	24
11	Periodic fever 3–6 days	202	23
12	Self limiting episodes	183	35
13	Response to tonsillectomy	182	33
14	Improvement with age	160	40
15	Exclusion cyclic neutropenia/immunodeficiency	150	34
16	Normalization of acute phase reactants in well-being	146	33
17	Recurrence every 3–6 weeks	145	21

### P8 Safety and efficacy of long-term canakinumab therapy in patients with caps: final results from β-confident registry

#### J.B. Kuemmerle-Deschner^1^, H.M. Hoffman^2^, P.N. Hawkins^3^, T. van der Poll^4^, U.A. Walker^5^, A. Speziale^6^, Y. Joubert^6^, H.H. Tilson^7^

##### ^1^University Hospital Tuebingen, Tuebingen , Germany; ^2^University of California, San Diego, CA, USA; ^3^University College London Medical School, London, United Kingdom; ^4^Academic Medical Center, University of Amsterdam, Amsterdam, Netherlands; ^5^University Hospital, Basel, Switzerland; ^6^Novartis Pharma AG, Basel, Switzerland; ^7^University of North Carolina, Chapel Hill, NC, USA

###### **Presenting author:** J.B. Kuemmerle-Deschner

This abstract is not included here as it has already been published.

### P9 Development and validation of diagnostic criteria for cryopyrin associated periodic syndromes

#### Jasmin Kuemmerle-Deschner^1^, Seza Ozen^2^, Pascal N. Tyrrell^3^, Isabelle Koné-Paut^4^, Raphaela Goldbach-Mansky^5^, Helen Lachmann^6^, Norbert Blank^7^, Hal M. Hoffman^8^, Elisabeth Weissbarth-Riedel^9^, Boris Huegle^10^, Tilmann Kallinich^11^, Marco Gattorno^12^, Ahmet Gul^13^, Nienke M. ter Haar^14^, Marlen Oswald^1^, Fatma Dedeoglu^15^, Susanne M. Benseler^16^

##### ^1^Department of Pediatrics, Division of Pediatric Rheumatology, University Hospital Tuebingen, Tuebingen, Germany; ^2^Pediatric Nephrology and Rheumatology, Hacettepe University, Ankara, Turkey; ^3^Department of Medical Imaging, University of Toronto, Toronto, ON, Canada; ^4^Pediatric Rheumatology, CHU Bicêtre, Le Kremlin Bicêtre, France; ^5^Translational Autoinflammatory Disease Section, NIAMS/NIH, Bethesda, MD, USA; ^6^UK National Amyloidosis Centre, University College London Medical School, London, United Kingdom; ^7^Haematologie, Onkologie und Rheumatologie, Universitaetsklinikum Heidelberg, Heidelberg, Germany; ^8^Division of Allergy, Immunology and Rheumatology, Department of Pediatrics, UC San Diego/Rady Children’s Hospital, San Diego, CA, USA; ^9^Kinderrheumatologische Ambulanz, Universitaetsklinikum Eppendorf, Hamburg, Germany; ^10^German Center for Pediatric and Adolescent Rheumatology, Garmisch-Partenkirchen, Germany, ^11^Rheumatology, Charité, University Medicine Berlin, Berlin, Germany; ^12^UO Pediatria 2, G. Gaslini Institute, Genoa, Italy; ^13^Department of Internal Medicine, Rheumatology Division, Istanbul University, Istanbul, Turkey; ^14^Laboratory for Translational Immunology, University Medical Center Utrecht, Utrecht, Netherlands;^15^Division of Immunology, Boston Children's Hospital, Boston, MA, USA; ^16^Rheumatology, Alberta Children's Hospital, University of Calgary, Calgary, AB, Canada

###### **Presenting author:** Jasmin Kuemmerle-Deschner

This abstract is not included here as it has already been published.

### P10 Application of Gaslini Diagnostic Score in Japanese children with periodic fever

#### Aki Hanaya^1^, Takako Miyamae^2^, Manabu Kawamoto^2^, Yumi Tani^1^, Takuma Hara^2^, Yasushi Kawaguchi^2^, Satoru Nagata^1^, Hisashi Yamanaka^2^

##### ^1^Department of Pediatrics, Tokyo, Japan; ^2^Institute of Rheumatology, Tokyo Women’s Medical University, Tokyo, Japan

###### **Presenting author:** Aki Hanaya

**Introduction:** The Gaslini Diagnostic Score (GDS) is a differential diagnostic score intended to determine a patient’s risk of having genetic factors contributing to PFAPA syndrome (periodic fevers with aphthous stomatitis, pharyngitis, and adenitis). The GDS is calculated based on the following criteria: age at onset, positive family history of periodic fever, chest pain, abdominal pain, and diarrhea. While the presence of any of the clinical symptoms is associated with an increased likelihood of a genetic basis, a child with a GDS of 1.32 or higher is considered likely to be at high risk of having a hereditary periodic fever syndrome. When applied in European countries, the GDS showed very high sensitivity (>90%) and a specificity of 59% to 82% .

**Objectives:** To investigate the utility of GDS in Japanese children diagnosed with PFAPA.

**Methods:** Children 5 years old or younger who had been diagnosed with periodic fever were enrolled in this study. The observed clinical symptoms for each subject were used in determining his or her GDS, in order to compare the score to the presence or absence of polymorphisms in 10 genes that are frequently implicated in autoinflammatory diseases. Each patient’s genomic DNA was isolated from peripheral blood and subjected to polymerase chain reaction (PCR) to amplify selected exons of the genes of interest. The three genes most frequently reported as being associated with PFAPA (and the exons amplified for each in this study) include: pyrin/marenostrin (MEFV, exons 1 to 10), tumor necrosis factor receptor superfamily member 1A (TNFRSF1A, exons 2, 3, 4), and mevalonate kinase (MVK, exons 9, 10, 11). The PCR products were purified, sequenced using the BigDye® Terminator v3.1 Cycle Sequencing Kit (ThermoFisher Scientific, Waltham, Mass. USA), and analyzed using an ABI 3130xl Prism Genetic Analyzer (Applied Biosystems, Inc., Foster City, Calif. USA).

**Results:** Of the 35 children enrolled (23 boys and 12 girls, with an average age at onset of 36.2 ± 18.4 months), 42.8% developed aphthous stomatitis, 34.3% reported stomachache, 11.4% had diarrhea, and 11.4% reported pectoralgia. Thirteen (37.1%) had a family history of periodic fever. A GDS score higher than 1.32 was seen in only 5 patients. However, 2 of those 5 had MEFV E148Q hetero, a fairly common polymorphism, found in 20% to 30% of healthy Japanese people. Of the 5, 2 other patients had MEFV polymorphisms (E148Q/P369S/R408Q hetero, E148Q homo). Of the 30 subjects with a GDS lower than 1.32, 13 showed no polymorphisms, while the other 17 patients showed MEFV polymorphisms (6 E148Q heteros, 4 E148Q/P369S/R408Q heteros, 3 E148Q/L110P heteros, 2 R202 heteros, 1 P369S/R408Q homo, 1 E84K hetero). Two patients with high GDS improved after administration of cimetidine or prednisolone during an acute attack. Four patients with low GDS were clinically diagnosed with Familial Mediterranean fever, based on efficacy of colchicine.

**Conclusion:** Application of the GDS in Japanese children with PFAPA revealed low sensitivity (15%) and comparatively high specificity (86%), excluding E148Q heteros. Therefore, we concluded that the GDS is not an informative diagnostic tool for Japanese PFAPA patients.

**Disclosure of Interest**

None Declared

### P11 Our experience in the treatment of PAPA syndrome

#### Almira Ćosićkić^1^, Fahrija Skokić^2^, Belkisa Čolić^1^, Sanimir Suljendić^3^

##### ^1^Reumatology, Allergology and Immunology, University Clinical Center, Bosnia and Herzegovina, Tuzla, Bosnia and Herzegovina; ^2^ Neonatology, University Clinical Center, Bosnia and Herzegovina, Tuzla, Bosnia and Herzegovina; ^3^Childens Surgery, Childrens Hospital, University Clinical Center, Bosnia and Herzegovina, Tuzla, Bosnia and Herzegovina

###### **Presenting author:** Almira Ćosićkić

**Introduction:** PAPA syndrome, is a rare autosomal dominant autoinflammatory disorder and its management still represents a challenge

**Objectives:** Our goal is to present our only experience in the treatment of PAPA syndrome.

**Methods:** We report the case of a 13 year old boy who was referred to our hospital with two weeks history of swollen and painful left knee and right ankle. Since the ages of 2 he was treated in another hospital for recurrent sterile arthritis and skin ulcer; last two years he was treated with Etanercept. His first relative suffers from recurrent sterile arthritis, skin ulcers and acnae. Although genetic analysis not made, arthritis of boy was suspected associated with PAPA syndrome. On examination, left knee, right ankle were swollen, painful with limitation of movement, right elbow with the flexion contracture. A rough scars on the skin above elbows, ankles, right knee, lower legs and on the back were present. Laboratory findings were abnormal with the ESR of 110 mm/h, CRP of 90.0 mg/l, mild anemia, and sterile culture of an aspirate from left knee.

**Results:** The boy responded well to start treatment with Infliximab, pulses and intra-articular steroides, in terms that after a few days the swelling and pain diminished, movements was better also; in addition, laboratory results returned to normal. In the following months the disease was partially controlled considering that after 7 months of treatment the boy had skin manifestations (ulcer and milde acne), so tetracycline were added. After 16 months achived complete response but he developed generalized urticaria during of administration of Infliximab. In coming weeks he had flares of arthritis and skin manifestations, elevated values of ESR, CRP, along with sterile cultures of an aspirate. We started treatment with pulses of steroids, Methotrexate and Adalimumab 40 mg every 14 days. After 2 months of treatment skin manifestation was significant improved, while arthritis was partiali resolved. At last vitis, after 10 months of treatment the boy was without skin manifestations but with occasional manifestations of arthritis who were treated by intra-articual steroids.

**Conclusion:** Our observations indicate that treatment with infliximab, if tolerated, may be more effective than adalimumab, although adalimumab had better effects on the skin manifestations.

**Disclosure of Interest**

None Declared

### P12 Enterocolitis and recurrent macrophage activation syndrome in a patient with G786V mutation of NLRC4 gene

#### Anna Kozlova, Irina Mersiyanova, Mariya Panina, Lily Hachtryan, Vasiliy Burlakov, Elena Raikina, Alexey Maschan, Anna Shcherbina

##### Immunology, Federal Research and Clinical Center of Pediatric Hematology, Oncology and Immunology, Moscow, Russian Federation

###### **Presenting author:** Anna Kozlova

**Introduction:** Autoinflammatory disorders comprise heterogeneous group of diseases characterized by chronic or recurrent systemic sterile inflammation as a result of genetic defects of regulators of inflammation. The NLRC4 is a member of the NOD-like receptor family of intracellular sensors that has a pivotal role in antimicrobial defense via intracellular sensing of a wide range of pathogens. NLRC4 is activated by NAIP-mediated recognition of flagellin or components of bacterial type 3 secretion systems (TTSS) in the host cell cytosol, leading to the assembly of inflammasome that mediates proteolytic cleavage of immature pro-IL-1β and pro-IL-18 and release of their bioactive forms into the extracellular space. Heterozygous mutations of NLRC4 gene lead to autoinflammation with infantile enterocolitis (AIFEC) syndrome. AIFEC symptoms include early onset growth retardation, enterocolitis, splenomegaly and fatal or near fatal episodes of systemic inflammation with macrophage activation.

**Objectives:** We report a patient with clinical picture typical of NLRC4 defect.

**Methods: Case report.**

**Results:** An index case is a 3 years old female with symptoms similar to AIFEC syndrome: episodes of fever, rash, enterocolitis, arthritis, hepatosplenomegaly, lymphoadenopathy and laboratory changes (elevated ESR, CRP, hypergammaglobulinemia, anemia, thrombocytosis/thrombocytopenia, leukocytosis/leukopenia, high level of ferritin and LDG) since infancy. Mutations in the genes typical for some autoinflammatory disorders (MEFV, MVK, TNFRSF1A, NLRP3), familiar hemaphagocytosis (SH2D1A, PRF1, STX11, STXBP2), as well as FAS, KRAS, NRAS, PTPN11, CBL were not found. The mutation c. 2357G > C (G786V) of NLRC4 gene, which has been described as polymorphism, but also described in an AIFEC-like patient, was found. Healthy father is a carrier of the same mutation. Prednison therapy didn’t alleviate symptoms. Treatment according to HLH-2004 protocol (dexamethasone, etoposide, cyclosporin A) and rituximab led to a temporary remission, with relapse of symptoms upon protocol completion. IL1 inhibitor (Anakinra, 5 mg/kg) therapy led to complete resolution of the symptoms.

**Conclusion:** In the light of patient’s clinical phenotype consistent with AIFEC the pathogenic role the NLRC4 c. 2357G > C variant requires further investigation. The patient is currently submitted to WES to rule out other genetic mechanisms of her disease.

**Disclosure of Interest**

None Declared

### P13 Decreased vitamin B12 levels in children with familial Mediterranean fever

#### Banu Acar^1^, Meryem Albayrak^2^

##### ^1^Department of Pediatric Rheumatology, Kırıkkale University, School of Medicine, Kırıkkale, Turkey; ^2^Department of Pediatric Hematology, Kırıkkale University, School of Medicine, Kırıkkale, Turkey

###### **Presenting author:** Banu Acar

**Introduction:** Familial Mediterranean fever (FMF), the most common autoinflammatory hereditary fever disease, is characterized by paroxysmal attacks of serosal inflammation. Colchicine is highly effective in preventing these attacks but it may also disrupt the intestinal absorption of vitamin B12.

**Objectives:** We hypothesized that patients treated with colchicine for a prolonged period could develop deficiency of the vitamin. Symptoms of B12 deficiency are confused with some findings of FMF if the elusiveness to investigate vitamin B12 deficiency.

**Methods:** Twenty FMF patients on regular colchicine treatment for at least 1 year and age and sex-matched 20 healthy controls were enrolled. Weakness, tiredness, a loss of appetite, nerve problems (numbness or tingling, muscle weakness, and problems walking) mental problems like memory loss, or behavioral changes were recorded. Complete blood count, peripheral blood smear, vitamin B12, homocysteine and folic acid were measured in each child. FMF patients diagnosed with vitamin B12 deficiency were given regular vitamin B12 treatment for a month. At the end of treatment complaints of patients were re-evaluated.

**Results:** Demographic, clinical, and laboratory features of the patients are shown in Table 16. The median hemoglobin, folic acid levels were not significantly different between the groups. Prevalence of B12 deficiency among patients with FMF was 35% compared with 15% in healthy controls. The median vitamin B12 values were not significantly different between the groups 310.41 (SD = 158.22) pg/mL vs. 351.83 (SD = 170.41) pg/mL, p = 0.67). At the beginning of vitamin B12 treatment, the median hemoglobin level was 11.3 g/dl, which increased to 12.4 g/dl at the end of the first month of vitamin B12 treatment. After the treatment of vitamin B12, arthralgia (25%), tiredness (10%) and memory loss (10%) declined in patients with FMF.

**Conclusion:** Vitamin B12 deficiency is common in patients with FMF. Although chronic arthralgia and fatigue are classic symptoms of FMF, they can also be the result of a vitamin B12 deficiency. The decision on an inadequate dosage of colchicine should not be drawn too hastily, reasons such as vitamin B12 deficiency, which can cause the patient’s symptoms should be considered.

**Disclosure of Interest**

None Declared

**Table 16 (abstract P13).** Demographic, clinical, and laboratory features of the patients

Characteristics	FMF	Control group
	n (%)	n (%)
Age (years)	8.2 ± 2,72	8,5 ± 2,85
Arthralgia	8 (40)	3 (15)
Limb pain with exercise / Heel pain	1 (5)/2 (10)	0 /0
Myalgia / Weakness	0/0	1 (5)/0
Tiredness	4 (20)	2 (10)
A loss of appetite,	3 (15)	1 (5)
Mental problems like memory loss, or behavioral changes	4 (20)	1 (5)
Vitamin B12 level (median-pg/ml)	310.41	351.83
Folic acid (median-ng/ml)	8,9 ± 7.2	10,3 ± 5.6

### P14 Canakinumab-treatment in autoinflammatory disease patients younger than 2 years old

#### Betul Sozeri^1^, Sezgin Sahin^2^, Kenan Barut^2^, Amra Adrovic^2^, Nese Inan^3^, Serhan Sevgi^3^, Ozgur Kasapcopur^2^

##### ^1^Pediatric Rheumatology, Erciyes University Faculty of Medicine, Kayseri, Turkey; ^2^Pediatric Rheumatology, Cerrahpasa Medical Faculty, Istanbul University, Istanbul, Turkey; ^3^Novartis Pharmaceutical Corporation, Istanbul, Turkey

###### **Presenting author:** Betul Sozeri

**Introduction:** The proinflammatory cytokine IL-1β has significantly increased the understanding of the pathogenesis of many autoinflammatory diseases (AID), including cryopyrin-associated periodic syndromes (CAPS), hyper IgD syndrome (HIDS), familial Mediterrenan fever (FMF), and systemic onset Juvenile idiopathic arthritis (sJIA). Canakinumab is a fully humanized monoclonal antibody (mAb) specific for IL-1β that has been indicated for a wide range of inflammatory disorders. Based on data from the treatment-withdrawal phase III trial, canakinumab was approved for use for the treatment of children aged >4 years for CAPS and >2 years for SJIA indications.

**Objectives:** The aim of this case report is to verify the efficacy and safety of canakinumab in patients younger than 2 years old.

**Methods:** Eight unrelated AID patients (4 sJIA, 2 NOMID/CINCA, 1 HIDS and 1 FMF and inflammatory bowel disease) are reported from two pediatric rheumatology centers. All patients were younger 24 months old and obtained their drugs off-label by approval of Ministry of Health.

**Results:** The results of eight patients (5 female, 3 male) are analyzed in this study. All patients were younger 24 months old (9 to 23 months; mean age = 18,6 months), and had canakinumab (3 mg/kg up to 6 mg/kg) s.c. every 4-8 weeks. Six out of eight patients were previously treated with anakinra for a median period of 55 days (range 1 to 150 days). The median follow up time was 20 months. Seven patients achieved a complete response while one patient was switched due to adverse event. Of the seven patients with the sJIA, HIDS and NOMID/CINCA, four (patients 1,2,4 and 5) did not require any adjustment of the therapy due to a persistent optimal control of both clinical and laboratory parameters during the whole follow-up period. Three others required at least one modification of the treatment schedule due to a persistent elevation of acute phase reactants. Compliance with treatment was good, and no reactions at the site of the injection were recorded. A patient (number 2) had pneumonia at month 20 who treated ambulatory with antibiotic. The administration of canakinumab was postponed until the complete resolution of the infection, which occurred without complications.

**Conclusion:** In this case series, we report the response to canakinumab in a AID pediatric patients. canakinumab was an effective treatment for patients with ADIs younger than 24 months old and well tolerated.

**Disclosure of Interest**

None Declared

### P15 Response of pamidronate treatment assessed by whole body magnetic resonance imaging in chronic non-bacterial osteomyelitis

#### Caroline M. Andreasen^1^, Anne Grethe Jurik^2^, Mia B. Glerup^3^, Christian Høst^3^, Birgitte T. Mahler^3^, Ellen-Margrethe Hauge^1^, Troels Herlin^3^

##### ^1^Dept. Rheumatology, Aarhus University Hospital, Aarhus, Denmark; ^2^Dept. Radiology, Aarhus University Hospital, Aarhus, Denmark; ^3^Dept. Paediatrics, Aarhus University Hospital, Aarhus, Denmark

###### **Presenting author:** Caroline M. Andreasen

**Introduction:** Pamidronate (PAM) may be effective to diminish pain and bone changes in chronic non-bacterial osteomyelitis (CNO). Whole body magnetic resonance imaging (WB MRI) can be used to assess bone involvement.

**Objectives:** To evaluate clinical and radiographic responses to PAM treatment as assessed by WB MRI and clinical response one and two year post-treatment.

**Methods:** We did a retrospective evaluation of children diagnosed with CNO according to Bristol criteria (ref.) and who had an insufficient response to NSAID treatment. Children were treated with i.v. PAM 1 mg/kg (max 60 mg/day) on 3 consecutive days every 3 months.

Seventeen children were treated for one year (8 girls and 9 boys) median age 11 years (range 5-13). All children were scheduled for two years of treatment. Data were available in 17 children after one year of treatment and nine children after two years of treatment.

WB MRI (1,5 Tesla) short tau inversion recovery (STIR) and T1-weighted images were performed at baseline (median -1 month, range -6-0), after one year (median 12 months, range 9-19) and two years (median 24 months, range 22-26). Clinical assessments, medical history and inflammatory biochemistry were obtained. For data not following a normal distribution Wilcoxon rank signed tests were used to assess change in number of bone lesions.

The study was performed according to Danish law after approval by institutional innovation board.

**Results:** Six children had elevated sedimentation rate at baseline, all normalized during the first year. Comorbidities were JIA (n = 3) and IBD (n = 1) no children had psoriasis. All children were treated with NSAID as first line treatment and further medical history was antibiotics 6%, methotrexate 41%, steroids 41%, anti-TNFα treatment before PAM 18% and 18% had anti-TNFα treatment added during PAM due to persistent symptoms and progression on WB MRI. The total number of lesions pre-treatment was 112, median 6 (range 2-14) and median number of clinical symptomatic lesions was 3 (range 1-8). Seven children had axial lesions median 1 (range 1-4) and six had lesions in relation to SI-joint median 1 (range 1-2). There were no erosive changes in the SI- joints. After one year the total number of lesions was 71, median 2 (range 0-21), seven had no lesions. Ten children had new or persisting lesions median 4 (range 1-15). One active spinal lesion and one SI- joint lesion persisted. Two children had deformation of vertebrae without persistent sign of inflammation on STIR-imaging. None of the data followed a normal distribution. There was a statistically significant change in number of bone lesions from baseline to year one (p = 0.047), particularly change in number of spinal lesions from baseline to year one (p = 0.016).

The clinical response after one year was complete pain relief 41%, partial pain relief 48% and no pain relief 11%. In six children the lesions resolved completely as assessed by WB MRI, but pain was still reported.

We evaluated preliminary data of 9 children after two years treatment. Five children remained without bone lesions and four children progressed during treatment with a median value of 3 new or worsening of lesions (range 1-10) as assessed by MRI.

**Conclusion:** PAM is a potent second-line treatment for CNO after one year. Axial lesions and SI- joint lesions respond well to PAM. Resolution of CNO lesions does not always correspond to clinical response and persistent pain may exist despite total resolution as assessed by WB MRI.

**References**

Roderick M et al. Efficacy of pamidronate therapy in children with chronic non-bacterial osteitis: disease activity assessment by whole body magnetic resonance imaging. Rheumatology 2014 Nov;53(11):1973–1976

**Disclosure of Interest**

None Declared

### P16 Clinical and laboratory characteristics in Marshall syndrome

#### Cecilia Lazea^1^, Laura Damian^2^, Calin Lazar^1^, Rodica Manasia^1^

##### ^1^Pediatrics, University of Medicine and Pharmacy Cluj-Napoca, Cluj-Napoca, Romania; ^2^Rheumatology, County Emergency Hospital, Cluj-Napoca, Romania

###### **Presenting author:** Cecilia Lazea

**Introduction:** Marshall syndrome (PFAPA) is characterized by recurrent episodes of fever associated with aphthous stomatitis, cervical adenitis or pharyngitis. It is relatively frequent in children, but the diagnosis is rarely established.

**Objectives:** The aim of this study was to establish the clinical and laboratory characteristics in Marshall syndrome.

**Methods:** Methods included: anamnesis, clinical examination, determination of ESR, PCR, TNF-α and other laboratoty tests. The treatment consisted in Prednison 1 mg/kg p.o.

**Results:** The age at the onset was 1.9 years and at diagnosis was 4.8 years. The mean interval between episodes was 4.1 weeks and the mean duration per febrile episode was 3.6 days. The patients presented: pharyngitis (100%), adenitis (100%) and aphthous lesions (60%). The mean value of ESR was 35 mm/hour, PCR was 9.2 mg/dL and leucocytes were 15.500/mm^3^. TNF-α was elevated during the febrile episodes (14.35 pg/mL) and remained high between them (11.3 pg/mL). Procalcitonin has normal values during flares. Family history revealed the presence of autoimmune diseases in 20% of patients. The patients were treated with Prednison with favorable evolution. Tonsillectomy was performed in three patients with remission of the symptoms.

**Conclusion:** Marshall syndrome should be suspected in children with periodic fever associated with pharyngitis, cervical adenitis and aphthous stomatitis. Although this is the most common cause of recurrent fever in children, the diagnosis is rarely established.

**Disclosure of Interest**

None Declared

### P17 Effective treatment of severe juvenile pustular psoriasis with cyclosporine and etanercept: a case report utilizing autoinflammatory diseases activity index (AIDAI) to document treatment response

#### Chloe M. Stephenson^1^, Vimal Prajapati^1^, Paivi M. Miettunen^2^

##### ^1^University of Calgary, Calgary, Canada; ^2^Paediatrics, University of Calgary, Calgary, Canada

###### **Presenting author:** Chloe M. Stephenson

**Introduction:** Pustular psoriasis is uncommon in children. The presentation is characterized by sudden flares of skin and extra-cutaneous manifestations, which is suggestive of an auto-inflammatory syndrome. Adult case reports have suggested use of topical corticosteroids, phototherapy, dapsone, methotrexate, cyclosporine, retinoids, and biologics (TNF-alpha antagonists) but there is no standardized or approved course of treatment.

**Objectives:** To describe a clinical course of a pediatric patient with severe pustular psoriasis with extracutaneous manifestations to sequential treatment course with topical and systemic therapies, utilizing Auto-Inflammatory Diseases Activity Index (AIDAI) to document treatment response.

**Methods:** A 3-year old Caucasian male was diagnosed with pustular psoriasis and prospectively followed from 2014 to 2016. He initially presented with a 6-month history of recurrent generalized pustules which were accompanied by high fever, conjunctivitis, myalgia, headache, anorexia and malaise. There was associated mild dactylitis, but no overt arthritis. HLA-B27 and ANA were negative. During flares, CBC revealed normal hemoglobin with leukocytosis and thrombocytosis and elevated serum C-reactive protein (CRP). The patient had several inpatient hospital admissions for suspected bacteremia with the febrile episodes but septic work-up was consistently negative. Family history included psoriasis, uveitis, and rheumatoid arthritis. Due to severity of skin disease and systemic features, genetic screening for an auto-inflammatory syndrome was considered but declined by the family as a skin biopsy confirmed psoriasis and did not reveal features suggestive of a hereditary recurrent fever syndrome. He was treated with numerous topical and systemic therapies as well as phototherapy. The response to treatment was assessed according to visual skin score for psoriasis along with sequential clinical photographs and an AIDAI score. AIDAI contains 12 items as follows: (1) fever ≥ 38.5 °C;(2) overall symptoms; (3) abdominal pain; (4) nausea/vomiting; (5) diarrhea; (6) headaches; 7) chest pain; (8) painful nodes; (9) arthralgia or myalgia;(10) swelling of the joints; (11) eye manifestations; (12) skin rash. The validated modified scoring system was used for each item with no (0) = absence of symptoms or yes (1) = presence of symptoms, with a total maximum score of “12”.

**Results:** Pre-treatment, he had 80% of his skin surface area involved with AIDAI score of 7/12. The following medications were ineffective: topical and oral corticosteroids (oral pednisone 1 mg/kg/day) and UVB phototherapy, followed by oral methotrexate (1 mg/kg once weekly) and colchicine (0.6 mg/day). Oral cyclosporine (3 mg/kg/day) was added to colchicine and methotrexate and resulted in approximately 50% improvement in cutaneous disease between flares, but systemic flares with skin involvement persisted with AIDAI score unchanged at 7/12. Due to incomplete response, subcutaneous etanercept was added (0.8 mg/kg once weekly). Within 2 weeks of initiation, the patient experienced complete remission of skin and extracutaneous manifestations with AIDAI score of 0/12. Methotrexate, colchicine and cyclosporine were subsequently tapered over 4 weeks. He remains in complete remission with etanercept monotherapy at one year follow-up.

**Conclusion:** Pustular psoriasis is challenging due to its rareness, severity and lack of uniformly effective treatment. In this case report, the patient’s severe skin and extracutaneous manifestations resolved once Etanercept was added to existing therapy, allowing rapid discontinuation of other therapies. AIDAI score was helpful in documenting treatment response.

**Disclosure of Interest**

None Declared

### P18 The prevelance of Fabry disease in familial Mediterranean fever children from Turkey

#### Dilek Yılmaz^1^, Yavuz Tokgöz^2^, Yasin Bulut^3^, Harun Çakmak^4^, Ferah Sönmez^1^

##### ^1^Pediatric Nephrology, Adnan Menderes University Faculty of Medicine, Aydın, Turkey; ^2^Pediatric Gastroenterology, Adnan Menderes University Faculty of Medicine, Aydın, Turkey; ^3^Pediatrcis, Adnan Menderes University Faculty of Medicine, Aydın, Turkey; ^4^Ophtalmology, Adnan Menderes University Faculty of Medicine, Aydın, Turkey

###### **Presenting author:** Dilek Yılmaz

**Introduction:** Fabry disease (FD) is a lysosomal storage disorder, caused by the deficiency of alpha-galactosidase A (AGALA) enzyme and can cause renal failure.

**Objectives:** We aimed to evaluate the prevalence of FD in familial mediterranean fever (FMF) children.

**Methods:** Between July 2009 and July 2015, a total of 76 children with diagnosis of FMF by new pediatric criteria were included in this study. Lochal Ethics Committee approval was given by the Clinical Research Ethics Committee of the Adnan Menderes University before starting this study. Signed informed consent was obtained from all participants for genetic analysis. A questionnaire was administered to all children for the symptoms of FD. And also, all participants are examined by the same ophthalmologist for FD. The urine analysis and FMF gene mutations of the patients were recorded. 24-hour urine protein was evaluated for each child. The renal ultrasound was done for renal cysts.

For the measurement of AGALA enzyme activity in FMF children, the blood drops were smeared onto a dry filter paper, and the meausrement of AGALA activity was performed by the dried blood spot method, with minor modifications. The cut- off value for the lysosomale enzyme activity was 1.2 μmol/l/h. Childrens who have low AGALA enzyme activity were screened for mutation analysis of the GLA gene.

**Results:** The patients’ clinical and demographic dates are given in Table 17. The female/male ratio was found to be 1.3. The mean age of FMF was 12.3 ± 3.9 years. Clinically positive patients with/without MEFV mutation were defined as phenotype I. All of the children were receiving colchicine treatment. The heterozygous mutation was detected in the MEFV gene was 51.3% (39/76), this rate was 19.7% (15/76) for the homozygous mutation and 14.5% (11/76) for both negative and combined heterozygotes mutation. Childrens were questioned for clinical signs of Fabry disease.51.3% of patients had long-term abdominal pain, 19.7% of patients had acroparesthesia and 9.2% of patients had skin angiokeratoma like lesion. In the eye examination, corneal opacity is not diagnosed in any of participitants. In the renal examination, 11.8% of patients had proteinuria and 5.3% of patients had renal cyst in the ultrasound. A total of 4 children (2 males) had decreased AGALA enzyme activity (<1.2 μmol/l/h). In those four cases, we determined M694V heterozygous mutation in one patient, M94I/V726A combined heterozygotes mutation in one patient and also negative mutations in two patients. Sequence anlysis of whole exons of GLA gene was performed for patients who have low enzyme activity, but any mutation was not found.

**Conclusion:** Fabry disease is a rare disorder, in which the clinical findings are confused with various systemic and rheumatic disorders, including FMF. Our study includes the most number of cases in the literature which the incidence of Fabry Disease in Familial Mediterranean Fever childrens are investigated. Fabry disease can cause serious complications and renal failure in adult life for the diagnosis of these disorders, we think the risk groups must be scan in childhood.

**Disclosure of Interest**

None Declared

**Table 17 (abstract P18).** Demographic and clinical parameters of the FMF children

Variables	Patients (n = 76)
Female (n, %)	43 (56.5)
Male (n, %)	33 (43.5)
Age (mean ± SD), years	12.3 ± 3.9
Alpha-galactosidase A (mean ± SD), μmol/l/h	2.95 ± 1.09
Clinical parameters	
Fever (%)	68.4
Abdominal pain (%)	86.6
Chest pain (%)	30.3
Arthritis (%)	72.4
Family history of FMF (%)	76.3

### P19 Is R202Q alteration of MEFV gene a disease-causing mutation?

#### Elif Comak^1^, Gülşah Kaya Aksoy^1^, Mustafa Koyun^1^, Sema Akman^1^, Yunus Arıkan^2^, Ender Terzioğlu^3^, Osman Nidai Özdeş^2^, İbrahim Keser^2^, Hüseyin Koçak^4^, Ayşen Bingöl^5^, Aygen Yılmaz^6^, Reha Artan^6^

##### ^1^Pediatric Nephrology - Rheumatology, Akdeniz University, Antalya, Turkey; ^2^Medical Genetics, Akdeniz University, Antalya, Turkey; ^3^Rheumatology, Akdeniz University, Antalya, Turkey; ^4^Nephrology, Akdeniz University, Antalya, Turkey; ^5^Immunology, Akdeniz University, Antalya, Turkey; ^6^Pediatric Gastroenterology, Hepatology & Nutrition, Akdeniz University, Antalya, Turkey

###### **Presenting author:** Elif Comak

**Introduction:** Familial Mediterranean Fever (FMF) is an autosomal recessive, inherited, autoinflammatory disease characterized by recurrent, self-limited febrile episodes of inflammation of serous membranes, which was caused by mutations in MEditerraneanFeVer gene (MEFV). To date over 300 alterations have been reported in MEFV genes. But, it is not clear whether all these alterations are disease-causing mutations.

**Objectives:** The aim of this study was to evaluate the clinical significance of homozygous R202Q alteration of the MEFV Gene.

**Methods:** Medical records of patients with homozygous R202Q alteration were screened retrospectively. Information including demographic features, clinical and laboratory findings, type of MEFV mutation were collected from the hospital’s computerized database. The patients having another MEFV mutations were excluded from the study.

**Results:** A total of 128 patients, 93 child (72.7%), 38 adult (27.3%) and 66 female (51.6%), 62 male (48.4%) were included in the study. Mean age at the onset of symptoms were 7.75 ± 4.10 years (1-17) and 30.09 ± 8.9 years (19-54) in children and adults, respectively. Three patients were excluded from the study, as their medical records were not available. The patients were classified according to the final diagnosis as FMF in 39 patients (30.6%), Henoch-Schonlein purpura in 10 patients (7.8%), PFAPA (periodic fevers with aphthous stomatitis, pharyngitis, and adenitis) syndrome in 6 patients (4.7%), juvenil idiopathic arthritis (2 oligoarticular onset, 1 enthesitis-related arthritis) in 3 patients (2.3%), Chron’s disease in 2 patients (1.6%), celiac disease, ulcerative colitis and mixed connective tissue disease in one patient each. 57 patients (44.6%) had FMF-like symptoms, without a diagnosis of FMF. Four asymptomatic patients (3.2%) were identified during family screening. A female adult patient was dignosed as FMF at the age of 22 and needed biological treatment (anakinra) at 27 years old due to colchicine resitance. Five patients had a history of appendectomy. In the study population, two patients developed chronic renal failure (CRF). One of them was diagnosed as CRF at 15 years old, when he had 3.2 gram/day proteinuria, without a spesific etiology. Another patient had CRF secondary to nephrolithiasis at the age of 42.

**Conclusion:** R202Q alteration of MEFV gene may lead to symptoms consistent with FMF in some cases. Also, this alteration may present with varied distinct clinical conditions other than FMF.

**Disclosure of Interest**

None Declared

### P20 Pharmacokinetics and pharmacodynamics of canakinumab in patients with periodic fever syndromes (colchicine-resistant FMF, HIDS/MKD and TRAPS): results from a phase III pivotal umbrella trial

#### F. De Benedetti^1^, J. Anton^2^, M. Gattorno^3^, H. Lachmann^4^, I. Kone-Paut^5^, S. Ozen^6^, J. Frenkel^7^, A. Simon^8^, A. Zeft^9^, E. Ben-Chetrit^10^, H.M. Hoffman^11^, Y. Joubert^12^, K. Lheritier^12^, A. Speziale^12^, J. Guido^12^, X. Xu^13^

##### ^1^IRCCS Ospedale Pediatrico Bambino Gesú, Rome, Italy; ^2^Hospital Sant Joan de Déu, Barcelona, Spain; ^3^Pediatric Rheumatology, G. Gaslini Institute, Genoa, Italy; ^4^UK National Amyloidosis Centre, University College London Medical School, London, United Kingdom; ^5^Hôpital Kremlin Bicetre, University of Paris SUD, Paris, France; ^6^ Department of Pediatrics, Hacettepe University, Ankara, Turkey; ^7^University Medical Center Utrecht, Utrecht, Netherlands; ^8^Radboud University Medical Centre, Nijmegen, Netherlands; ^9^Pediatrics Rheumatology, Cleveland Clinic, Cleveland, OH, USA; ^10^Rheumatology Unit, Hadassah—Hebrew University Medical Center, Jerusalem, Israel; ^11^University of California, San Diego, La Jolla, CA, USA; ^12^Novartis Pharma AG, Basel, Switzerland; ^13^Novartis Pharmaceuticals Corporation, East Hanover, NJ, USA

###### **Presenting author:** F. De Benedetti

**Introduction:** The pharmacokinetics (PK) of canakinumab (CAN) and total interleukin (IL)-1β kinetics have been well investigated in CAPS patients (pts) [1]. Here we present the PK and pharmacodynamics (PD) of CAN from the randomised treatment epoch (primary analysis up to Week 16) of a Phase III study in colchicine-resistant/intolerant familial Mediterranean fever (crFMF), hyper-IgD syndrome/mevalonate kinase deficiency (HIDS/MKD) and TNF receptor-associated periodic syndrome (TRAPS) pts.

**Objectives:** To evaluate the PK and PD of CAN (solution for injection-liquid in vial [LIVI]) in crFMF, HIDS/MKD and TRAPS pts at Week 16.

**Methods:** The study comprised 3 disease cohorts (crFMF, HIDS/MKD and TRAPS). Each cohort followed the same study design across 4 epochs (screening epoch [up to 12 weeks], randomised treatment epoch [16 weeks], randomised withdrawal epoch [24 weeks] and open-label treatment epoch [72 weeks]). Pts (age, ≥2 years) with crFMF, HIDS/MKD or TRAPS who had a flare during Epoch 1 were randomised (1:1) in Epoch 2 to receive subcutaneous (sc) CAN 150 mg (or 2 mg/kg for pts weighing ≤40 kg) every 4 weeks (q4w) or placebo. Blinded uptitration (up to 300 mg) was allowed for pts not resolving the index flare by Day 15. Serum samples for CAN concentrations and total IL-1β were collected at baseline (Day 1), and trough samples, at weeks 2, 4, 8, 12 and 16.

**Results:** In crFMF, HIDS/MKD and TRAPS pts, the serum clearance and steady-state volume of distribution of CAN varied according to body weight, and were estimated to be 0.14 ± 0.04 L/day and 4.96 ± 1.35 L, respectively. The estimated half-life of CAN was 25.6 ± 6.4 days. CAN minimal concentration at Week 16 following 150 mg sc q4w dosing was estimated to be 15.3 ± 6.6 μg/mL. The estimated steady-state area under the serum concentration-time curve from time zero to the end of the dosing interval tau (AUCtau) was 648 ± 202 μg.day/mL. Similar results were obtained in all 3 diseases. CAN binding to circulating IL-1β was demonstrated by increase in total IL-1β following CAN dosing in all 3 diseases. In pts requiring uptitration to 300 mg, levels of total IL-1β were higher, suggesting higher production of IL-1β, and therefore the need for a higher dose.

**Conclusion:** This was the first study to evaluate the PK of canakinumab given in the LIVI form. The results observed in crFMF, HIDS/MKD and TRAPS pts were similar to those observed in other indications (CAPS and SJIA) using the lyophilisate form. These data suggested that the new formulation did not affect the PK/PD of canakinumab and similar to CAPS, patients with higher levels of IL-1β require canakinumab uptitration to achieve optimal disease control.

**References**

1. Chakraborty A, et al. *Clin Pharmacokinet*. 2012;51:e1–18.

**Trial registration identifying number:** NCT02059291

**Disclosure of Interest**

F. De Benedetti Grant / Research Support from: Pfizer, Abbvie, Roche, Novartis, Novimmune and BMS, J. Anton Grant / Research Support from: Novartis, Consultant for: Novartis, M. Gattorno Grant / Research Support from: Novartis and SOBI, Consultant for: Novartis and SOBI, H. Lachmann Consultant for: Novartis, SOBI, Takeda and GSK, Speaker Bureau of: Novartis and SOBI, I. Kone-Paut Grant / Research Support from: Novartis, SOBI and Roche, Consultant for: Novartis, SOBI, Pfizer, Abbvie, Roche, S. Ozen Speaker Bureau of: Novartis and SOBI, J. Frenkel Grant / Research Support from: Novartis and SOBI, A. Simon Grant / Research Support from: Novartis, CSL, Behring and Xoma/Servier, Consultant for: Novartis, Takeda, Xoma and SOBI, A. Zeft Shareholder of: Merck, Opko Health, Arno therapeutics, Consultant for: Novartis, E. Ben-Chetrit Consultant for: Novartis, H. Hoffman Consultant for: Novartis, Speaker Bureau of: Novartis, Y. Joubert Employee of: Novartis, K. Lheritier Employee of: Novartis, A. Speziale Employee of: Novartis, J. Guido Employee of: Novartis, X. Xu Employee of: Novartis

### P21 Gene mutation of familial Mediterranean fever (FMF) registry in Iran

#### Fatemeh F. Mehregan^1^, Vahid Ziaee^2^, Mohammad H. Moradinejad^2^

##### ^1^Pediatric Rheumatology, Shahid Beheshti University of Medical Sciences, Tehran, Iran, Islamic Republic Of; ^2^Pediatric Rheumatology, Tehran University of Medical Science, Tehran, Iran, Islamic Republic Of

###### **Presenting author:** Fatemeh F. Mehregan

**Introduction:** Familial Mediterranean Fever (FMF) is autoinflammatory periodic disease. FMF is an autosomal recessive disorder characterized by recurrent febrile attacks accompanied by serosal and synovial membrane inflammation.

**Objectives:** FMF is caused by mutation in the MEFV gene are found usually among Mediterranean population, Armenians, Turks, Arabs and Jews.

We have a lot of different ethnicities in Iran like Armenians, Turks, Arabs and Jews and also consanguineous marriage is common among Iranian people. These lead to more frequent diagnosis of genetic disorders like FMF in our country.

We aim to identify the distribution and the frequency of the Mediterranean Fever (MEFV) gene mutation among FMF patient in the pediatric population in Tehran - Iran.

**Methods:** This study is a prospective cross sectional study of patients diagnosed with Familial Mediterranean Fever (FMF) who was registered in the autoinflammatory computer database registration through periodic fever clinic, which was established for this reason, in the medical center of children in 23.09.2012 in Tehran- Iran and data were collected with standardized forms. Of the total 102 FMF pediatric patients (68 male, 34 females) with a mean age of onset of disease 4.2 years, were screened for mutation in four exons (2, 3, 5 and 10) of MEFV gene. Genomic DNA was extracted from whole blood and entered in ARMS-PCR and PCR-RFLP reactions. When cases were negative in ARMS-PCR and PCR-RFLP, the exons were amplified and subjected to direct sequencing. The tested individuals were screened for the most common 12 MEFV mutations.

**Results:** we detected mutant gene in 88.37% of patients .thirty-three (38.4%) patients had heterozygote mutation for single gene, twenty-two (25.6%) had compound heterozygote mutations, fourteen (16.3%) had homozygote mutation and eleven (11.62%) patients had none of studied mutations. M694V was the most frequent mutation [n = 25, (28.40%)] followed by E148Q[n = 22,(25%)],M694I [n = 14 (15.90%)], M680I [n = 9,(10.22%)],V726A [n = 4,(4.54%)]mutations. V726I [n = 2, (2.27%)] and R761H [n = 2.(2.27%)] were more frequent than the others mutations gene.

**Conclusion:** In our study M694V, E148Q, M694I, M680I and V726A are the five most common mutations.

**Disclosure of Interest**

None Declared

### P22 Observational clinical survey and investigation of autoinflammatory candidate genes role in an Italian cohort of pediatric patients with chronic non-bacterial osteomyelitis

#### Giovanna Ferrara^1^, Serena Pastore^2^, Antonella Insalaco^3^, Manuela Pardeo^3^, Alberto Tommasini^2^, Francesco La Torre^4^, Clotilde Alizzi^5^, Rolando Cimaz^6^, Martina Finetti^7^, Marco Gattorno^7^, Pio D’Adamo^1^, Andrea Taddio^1,2^

##### ^1^University of Trieste, Trieste, Italy; ^2^Institute of Child and Maternal Health – IRCCS “Burlo Garofolo”, Trieste, Italy; ^3^IRCCS Ospedale Pediatrico Bambino Gesù, Rome, Italy; ^4^Antonio Perrino Hospital, Brindisi, Italy; ^5^Ospedale Pediatrico G. Di Cristina di Palermo, Palermo, Italy; ^6^Anna Meyer Children Hospital, Florence, Italy; ^7^UOC Pediatria 2, Istituto Gaslini, Genova, Italy

###### **Presenting author:** Giovanna Ferrara

**Introduction:** Chronic Non-Bacterial Osteomyelitis (CNO) is a rare inflammatory disorder that primarily affects children, characterized by insidious onset of pain, local bone expansion and radiological findings suggestive of osteomyelitis at single or multiple sites. The aetiology is unclear. Recent evidence strongly supports a genetic susceptibility, considering CNO a disease in the spectrum of autoinflammatory disorders.

**Objectives:** The aims of the study are to investigate the role of autoinflammatory candidate genes in a subgroup of patients of the Italian Cohort of CNO patients and to find possible correlation with clinical phenotype.

**Methods:** Blood samples were collected from 51 CNO patients of the cohort upon informed consensus. We selected 112 genes involved in autoinflammation and we used an Ion Torrent platform to sequence the coding regions. All the annotations and pathogenic prediction were performed with VEP (http://www.ensembl.org/info/docs/tools/vep/index.html) and all the potential pathogenic variants will be confirmed using Sanger sequencing. Laboratory data, diagnostic imaging, histological features and clinical course are also collected and all the data are stored in a relational database.

**Results:** Clinical data of 116 patients with CNO diagnosis and included in the Italian Cohort were collected. DNA samples from 51 patients were available. They were the only patients included in the present study. The patients were 33 females (65%) and 18 males (35%). Median age of first complaint was 10 years (range 4-16). Median sites number was 5 (range 1-26 sites). The majority of bone lesions was located in the long bones 63%, followed by pelvis (59%), column (43%), thorax (26%), clavicle (24%), extremities (17%), skulls (6%) and mandible (4%). Blood examination revealed increased erythrocyte sedimentation rate and C-reactive protein in 82% and 65% of cases respectively. In 36/51 (70%) patients the diagnosis was formalized after biopsy. At diagnosis X-ray was performed in 32 (63%) of patients and resulted positive for lesions in 17 of them (53%). Computer tomography was used in 12 patients (23%) and was negative only in one. Magnetic resonance (MRI) imaging was performed in 47 patients (90%) and was always positive. Radionuclide bone scanning was performed in 30 patients (59%) and was negative only in one case. All patients received non-steroidal anti-inflammatory drugs (NSAIDs) therapy, only 12 (23%) reached remission. About the other therapies: 9/18 reached remission with oral steroids, 3/11 with methotrexate, 4/8 with sulfasalazine, 10/17 with biologic therapy and 14/24 with bisphosphonates. Currently 43 patients (84%) are in clinical remission (16 on medications, 27 out of medications). All the 51 patients underwent sequencing of the selected genes. In the majority of cases, one or both parents were available for the analysis and were sequenced in order to exclude inherited variants. We are currently evaluating each variant with Sanger sequencing in order to assess their pathogenicity and have a clearest picture of CNO genetics.

**Conclusion:** This study confirms some findings already known of the disease: the prevalence of lesions in long bones, the high sensitivity of MRI and the high clinical remission rate. We have also sequenced the selected genes involved in autoinflammation. If the variants found and selected for big impact on the protein structure, will be confirmed with Sanger sequencing, we will try to find a correlation with clinical phenotypes.

**Disclosure of Interest**

None Declared

### P23 Effect of canakinumab on health-related quality of life in patients with periodic fever syndromes

#### H. Lachmann^1^, A. Simon^2^, J. Anton^3^, M. Gattorno^4^, I. Kone-Paut^5^, S. Ozen^6^, J. Frenkel^7^, E. Ben-Chetrit^8^, H. Hoffman^9^, A. Zeft^10^, Y. Joubert^11^, K. Lheritier^11^, A. Speziale^11^, G. Junge^11^, J. Gregson^11^, F. De Benedetti^12^

##### ^1^UK National Amyloidosis Centre, University College London Medical School, London, United Kingdom; ^2^Radboud University Medical Centre, Nijmegen, Netherlands; ^3^Hospital Sant Joan de Déu, Barcelona, Spain; ^4^Pediatric Rheumatology, G. Gaslini Institute, Genoa, Italy; ^5^Hôpital Kremlin Bicetre, University of Paris SUD, Paris, France; ^6^Hacettepe University Children’s Hospital, Ankara, Turkey; ^7^University Medical Center Utrecht, Utrecht, Netherlands; ^8^Rheumatology Unit, Hadassah—Hebrew University Medical Center, Jerusalem, Israel; ^9^University of California, La Jolla, USA; ^10^Pediatrics Rheumatology, Cleveland Clinic, Cleveland, OH, USA; ^11^Novartis Pharma AG, Basel, Switzerland; ^12^IRCCS Ospedale Pediatrico Bambino Gesú, Rome, Italy

###### **Presenting author:** H. Lachmann

**Introduction:** Open label studies have shown the efficacy of canakinumab (CAN), a fully human and highly specific anti-IL-1β monoclonal antibody, in patients (pts) with Periodic Fever Syndromes (PFS), which include colchicine-resistant Familial Mediterranean Fever (crFMF), Hyper-IgD Syndrome/Mevalonate Kinase Deficiency (HIDS/MKD) and TNF-Receptor Associated Periodic Syndrome (TRAPS). Currently, no data is available on the effect of CAN on Health-Related Quality of Life (HRQoL) in pts with PFS.

**Objectives:** To assess the effect of CAN on HRQoL using Child Health Questionnaire-Parent Form 50 (CHQ-PF50) and Short Form-12 Health Survey (SF-12) in pts with PFS.

**Methods:** In this Phase 3, randomised, placebo-controlled study of CAN in pts with PFS (NCT02059291), SF-12 Physical Component Summary (PCS) and Mental Component Summary (MCS) scores were assessed in adults, whereas CHQ-PF50 Physical Summary (PhS) and Psychosocial Summary (PsS) scores were assessed in children (>5– < 18 years).

**Results:** Of the 181 pts (63 crFMF, 72 MKD/HIDS, 46 TRAPS) randomised to CAN or placebo, 71 were adults (age ≥18 years) and 110 were children (age ≥2– < 18 years). Pts who initially received placebo and did not respond were switched to CAN. Treatment with CAN was associated with an early clinically meaningful improvement in SF-12 PCS scores reported at Week (Wk) 5, which were sustained and increased to a large effect size by Wk 16 for all indications (Table). Similarly, clinically meaningful improvement in SF-12 MCS, CHQ-PF50 PhS and CHQ-PF50 PsS scores was observed for all indications, except for PsS in HIDS/MKD and TRAPS pts.

**Conclusion:** Canakinumab showed rapid improvement by Wk 5 in pt-reported outcomes in adults and children with PFS, which was sustained through Wk 16.

**References**

1. User’s manual for the SF-12v2 Health Survey, 3rd ed.; 2012

2. Cohen J, et al. Statistical power analysis for the behavioural sciences, 2nd ed.; 1988

3. HealthActCHQ. The CHQ Scoring and Interpretation Manual (Boston, MA: HealthActCHQ, 2013)

4. Maruish, M.E. ed. User’s manual for the SF-12v2 Health Survey, 3rd ed. (Lincoln, RI: QualityMetric Inc., 2012)

**Trial registration identifying number:** NCT02059291

**Disclosure of Interest**

H. Lachmann Consultant for: Novartis, SOBI, Takeda and GSK, Speaker Bureau of: Novartis and SOBI, A. Simon Grant / Research Support from: CSL Behring, Novartis, Xoma/Servier, J. Anton Grant / Research Support from: Novartis, Pfizer, Abbvie, Roche and SOBI, Consultant for: Novartis, M. Gattorno Grant / Research Support from: Novartis and SOBI, Consultant for: Novartis and SOBI, Speaker Bureau of: Novartis and SOBI, I. Kone-Paut Grant / Research Support from: SOBI, Roche and Novartis, Consultant for: Novartis, SOBI, Pfizer, Abbvie and Chugai, S. Ozen Consultant for: Novartis, Speaker Bureau of: SOBI, J. Frenkel Grant / Research Support from: Novartis and SOBI, E. Ben-Chetrit Consultant for: Novartis, H. Hoffman Grant / Research Support from: Bristol Myers Squibb, Consultant for: Novartis, Sob Biovitrum, Regeneron, Speaker Bureau of: Novartis, A. Zeft: None Declared, Y. Joubert Employee of: Novartis, K. Lheritier Employee of: Novartis, A. Speziale Employee of: Novartis, G. Junge Employee of: Novartis, J. Gregson Employee of: Novartis, F. De Benedetti Grant / Research Support from: Pfizer, Abbvie, Roche, Novartis, Novimmune and BMS

**Table 18 (abstract P23).** Patient reported outcomes

Outcome measures	Mean change from baseline (n/N)^a,c^					
	crFMF		HIDS/MKD		TRAPS	
	Week 5	Week 16	Week 5	Week 16	Week 5	Week 16
SF-12 PCS	7.9 (29/30)	9.55 (30/31)	13.81 (15/15)	13.81 (14/14)	9.63 (16/17)	11.64 (13/14)
SF-12 MCS	4.83 (29/30)	4.27 (30/31)	6.41 (15/15)	8.14 (14/14)	5.65 (16/17)	5.51 (13/14)
CHQ-PF50 PhS	13.2 (21/24)	20.1 (18/21)	5.5 (32/34)	9.9 (27/29)	7.4 (16/18)	14.9 (13/14)
CHQ-PF50 PsS	4.1 (21/24)	7.2 (18/21)	1.8^b^ (32/34)	5.2 (27/29)	0.9^b^ (16/18)	1.2^b^ (13/14)

*N* total number of pts, *n* pts who received at least one dose of CAN

^a^An increase in mean score from baseline is an improvement

^b^Minimal important difference [1,2] from baseline was not achieved

^c^In the general U.S. population, mean summary scores are 50 for CHQ-PF50 and 50 for SF-12 (50.01 and 49.98, respectively for PCS and MCS). [3,4]

### P24 Recurrent hyperbilirubinemia as a rare feature of familial Mediterranean fever

#### Hasmik Sargsyan

##### Yerevan State Medical University, Yerevan, Armenia

**Introduction:** Familial Mediterranean Fever (FMF) is worldwide the most common autoinflammatory disease. It is inherited as an autosomal recessive trait und usually affects people living in or originating from the Mediterranean basin, including Armenians, Sephardi Jews, Arabs, Turks. Clinical feature of FMF are characterized by attacks of fever and painful serositis. There are described some case reports of FMF with hyperbilirubinemia.

**Objectives:** We present FMF Armenian patient with a single MEFV gene mutation and hyperbilirubinemia during the attacks, with the interesting family history. Nevertheless high incidence of FMF in Armenia, the case with hyperbilirubinemia in Armenian children have not yet been described.

**Methods:** Clinical and laboratory findings are presented.

**Results:** 16 years old boy admitted to the “Arabkir” Medical Center with complains of acute abdominal and chest pain, fever and jaundice. Manifestation of disease from the 4 years, frequency of attacks 2-3 months one time, lasting 1-2 days. The family history was: brother had FMF (MEFV-M694/N) with splenomegaly and gallbladder stone, sister had FMF(MEFV-M694V/V726A) with arthropathy and obesity. Examination of patient during the attack: slight yellowish coloration of eyes (which was disappear after the attack), with abdominal tenderness, distension. Chest X- ray reviled plural effution, echocardiography- pericarditis. Simple blood test reviled mild leukocytosis (11.8/mm3), mild elevation of erythrocyte sedimentation rate (25 mm/lst hour). In the biochemical investigations all indices was without distractions besides: CRP (6 time more than normal), mild elevation of bilirubin – total 26,0(N < 21mkmoll/l),direct-10.0(N < 3,4mkmol/l), indirect – 16.0 (N < 17,6mkmol/l). All conditions due to direct hyperbilirubinemia was excluded. After 2-3 days of FMF attack, had normalization all indices. His upper ultrasound reviled inhomogeneous gallbladder and splenomegaly. Some other investigation (liver biopsy, coproporfirin level and etc,) could not be performed for the parents refused of further investigations. Genetic investigation of the patient reviled M694V/N mutation. We established the diagnosis of FMF according Tel-Hashomer criteria, at the same time excluding other conditions accompanying with hyperbilirubinemia.

**Conclusion:** This is a rare case of mild hyperbilirubinemia with family history of FMF with interesting family tree. There is no doubt, that display of heperbilirubinemia during the attack is mediated by the effect of cytokines on the liver. As evidenced by a number of literature these cytokines decrease bile excretion and increase serum level of bile acids. It is not excluded that these mechanism operates mainly in cases, when there are changes of bile excretion and some changes of metabolism of lipids in the family history of FMF patients. For more detail evaluation of hyperbilirubinemia in FMF, should be necessary to have large number of group with investigations their family tree.

**Disclosure of Interest**

None Declared

### P25 Is the Gilbert syndrome coincidence of familial Mediterranean fever: case report

#### Hasmik Sargsyan

##### Yerevan State Medical University, Yerevan, Armenia

**Introduction:** Familial Mediterranean Fever (FMF) is an autosomal recessive disease (MEFV gen) characterized by recurrent fever and inflammatory serositis. It is reaching populations which ancestors are from Mediterranean countries. The gen of FMF is located in 16 chromosome. Gilbert syndrome is inherited as an autosomal recessive (UGT1A1 gen) trait which gene is located on chromosome 2. Although Gilbert syndrome may become apparent shortly after birth, it may not be recognized for many years. People with Gilbert syndrome have a unconjugated hyperbilirubinemia. Most affected individuals asymptomatic or may only exhibit mild yellowing of the skin, eyes, mucous membranes.

There are rarly data about coexistence of FMF with Gelbert syndrome.

**Objectives:** We present FMF patient coexistence with Gilbert syndrome.

**Methods:** Clinical and laboratory findings are presented.

**Results:** 16 years old boy admitted to the “Arabkir” medical Center with complains of: fever, abdominal pain, chest pain. Manifestation of disease began from the infancy period only with fever, but from the 8-9 years it was accompany with abdominal and chest pains. Family history: father has FMF with M694V/M694V mutation and gold bladder stone. There are too some cases of FMF from the mother and father relatives, including secondary amyloidosis also which whom conducted transplantation of kidney. The attacks of patient was 1-3 times per month each lasting typically for 3-4 days. Examinations; skin with yellow coloration. RBC results showed leucocytosis with elevated CRP and ESR. Chest X-ray reviled exudates in the costophrenic angle from the two sites, ultrasound investigation - splenomegaly. Genetic investigation of MEFV- M694V/N mutations. We established the diagnosis of FMF (according criteria Tel-Hashomer) and Gilbert syndrome (according clinical feature and genetic homozygotes mutations (UGT1A1). At the same time we rule out other conditions with similar symptoms.

**Conclusion:** According the literature data and our long work in this field, we can conclude, that liver has very important role in the development of FMF attack which is trough hepatomegaly and biochemical deviations in some cases. We can presume, that coexistence of two diseases due to the affection of cytokines which are more producing during the attacks of FMF and which are decreasing the activity of uridine 5-diphospho- glucuronosyltransferase (mutation in this enzyme cause Gilbert’s disease), It is not excluded some interaction of genes of above diseases.

**References**

1. Y. Yazgan et all. Is Hyperbilirubinemia a component or just a coincidence of familial Mediterranean fever: A case report and review of the literature//Turk J Gastroenterol 2003:14(1);71–73

2. M. Gattorno. Familial Mediterranean Fever – 2015-Medical

**Disclosure of Interest**

None Declared

### P26 Evaluation of biopsychosocial aspects of patients with juvenile Behçet’s disease: a qualitative study

#### Hulya Zengin^1^, Berna E. Fidanci^1^, Cagla Kaymakamgil^1^, Dilek Konukbay^1^, Dogan Simsek^2^, Ezgi D. Batu^3^, Dilek Yildiz^1^, Faysal Gok^4^, Seza Ozen^3^, Erkan Demirkaya^5^

##### ^1^Child Health Nursing, Gülhane Military Medical Academy, Ankara, Turkey; ^2^Pediatric Rheumatology, Gülhane Military Medical Academy, Ankara, Turkey; ^3^Pediatric Rheumatology, Hacettepe University, Ankara, Turkey; ^4^Pediatric Nephrology, Gülhane Military Medical Academy, Ankara, Turkey; ^5^Pediatric Rheumatology, FAVOR, Ankara, Turkey

###### **Presenting author:** Hulya Zengin

**Introduction:** Behçet’s disease is a chronic, recurrent and multisystemic disease that can involve the skin, mucosal surfaces, eyes, joints and other organs. Behçet’s disease, which progresses with flare and remissions, can limit individuals’ daily activities, disturb their psychological well-being and ruins the quality of life.

**Objectives:** In this qualitative part of the study, it is primarily aimed to enrich the item generation for the multidimensional assessment of juvenile Behçet’s disease.

**Methods:** The research was conducted in the Pediatric Rheumatology Department of Gulhane Military Medical Academy and Hacettepe University in Ankara, Turkey. Twenty children with Behçet’s disease and fourteen of their parents were enrolled in this qualitative study. In this study, we used a qualitative study design and a grounded theory approach. The first step was data collection, through individual in-depth interviews. From the collected data, the key points were marked with a series of codes, which are extracted from the text. The codes were grouped into similar concepts. From these concepts, categories were produced, which were the basis for the creation of a theory. The data were collected using both a demographic data form and a semi-structured interview form. The interview form was developed by the authors based on the literature search, the theoretical framework, expert opinion, and included open-ended items to gather data regarding the experiences of the participants with respect to their diseases. The study was performed on individual patient face-to face interview. Data were analyzed by grounded theory and the N Vivo 9 software program.

**Results:** Four categories were obtained. These categories were (i) physical effects of the illness, (ii) emotional effects of the illness, (iii) social effects of the illness and (iv) experienced challenges related to treatment process. In the physical effect category there are two themes; symptoms and the limitations about the illness. In the symptoms theme there are aphthous lesions, genital lesions, pain, visual problems, and in the limitations about the illness theme there are speaking and feeding difficulties related to aphthous lesions and walking difficulties related to genital lesions. The emotional effect category includes two themes, psychological impacts, coping and support. In the psychological impacts theme there are anger, disability to cope, rebellion against disease and emotional exhaustion. In the social effects category there are three themes; school-related problems, problems with peer relations and problems with social life. In the school related problems theme, decrease in academic performance, absenteeism to school and concealing the sickness from friends; in the problems with peer relations theme, fighting with friends, reduction in peer relationships; in the problems with social life theme, social isolation, inability to adapt to their peers, hide the illness were the most common features. The fourth category includes three themes; lack of knowledge about the illness, problems with medication and reaching the health facility.

**Conclusion:** These results provide us evidence-based data and necessity for the assessment of children with Behçet’s disease with multidimensional approach including physical, emotional and social aspects as well as treatment process. Our study also contributes the basis and/or justification for selecting the domains that the developing multidimensional instrument should include.

**Disclosure of Interest**

None Declared

### P27 Comparison of neutrophilic activation and secretion of cytokines derived from patients with familial Mediterranean fever and other acute and chronic inflammatory diseases

#### Iris Stoler^1^, Judith Freytag^1^, Banu Orak^1^, Christine Seib^1^, Lars Esmann^2^, Eva Seipelt^3^, Faekah Gohar^4^, Dirk Foell^4^, Helmut Wittkowski^4^, Tilmann Kallinich^1^

##### ^1^Department of Pediatric Rheumatology, Charité Berlin, Berlin, Germany; ^2^Charité Berlin, Berlin, Germany; ^3^Immanuel Berlin Buch, Berlin, Germany; ^4^University Hospital Muenster, Albert-Schweitzer-Campus 1, Gebäude W30, Münster, Germany

###### **Presenting author:** Iris Stoler

**Introduction:** In Familiar Mediterranean Fever (FMF), the most common monogenic autoinflammatory disorder, gain-of-function mutations lead to an increased activation of the inflammasome. Neutrophils play a key role in the pathogenesis of FMF.

**Objectives:** (i) To identify a FMF-specific genotype-dependent activation of neutrophilic surface markers and (ii) to describe an ex vivo activation of the inflammasome and the secretion of cytokines and S-100 molecules. (iii) To compare the observed activation patterns to changes in neutrophils from patients with other inflammatory diseases.

**Methods:** 6 FMF patients, 6 healthy heterozygous carriers and 4-6 patients with active Crohn’s disease, cystic fibrosis, rheumatoid arthritis / spondylarthritis, immundeficiencies with autoinflammation, other autoinflammatory diseases and acute infections as well as healthy controls were analyzed. CRP, IL-18, S100A12 und Caspase-1 were determined in serum. After neutrophil seperation by density gradient, neutrophils were cultivated. The kinetics of the surface expression of CD62L and CD11b was measured at 0 h, 1/2 h, 1 h, 2 h, 3 h, 4 h and 5 h. Furthermore, the cells from the FMF patients were stimulated (LPS 4 h + ATP 30 min) and treated with colchicine. S100A12, IL-18 and Caspase-1 were measured in the cell culture supernatants at the correspondent time-points. The effect of an IL-1 inhibition on the secretion of the specified parameters was evaluated by treatment with Anakinra.

**Results:** Compared to healthy controls, neutrophil granulocytes from FMF patients show a spontaneous hypersecretion of IL-18, Caspase-1, S100A12 and S100A8/9. Neutrophils from homozygous FMF patients showed a strong spontaneous activation measured by means of CD62L expression (MIF 1736 [SD 453 before cultivation; MIF 22 [SD 33] after 5 hours). Neutrophils from healthy heterozygous FMF subjects exhibited lower activation levels (MIF 1216 [382] vs. 709 [208]). In contrast, neutrophils from patients with other inflammatory diseases (2093 [661] vs. 1734 [579]) and healthy controls (1766 [512] vs. 1478 [345]) showed low activation levels. Patients with acute infections showed an inhomogenous activation pattern. Blockade of IL-1 did not alter spontaneous neutrophilc activation.

**Conclusion:** The spontaneous activation of neutrophils from patients with FMF is a disease specific genotype-dependent phenomenon.

**Disclosure of Interest**

None Declared

### P28 Nail fold capillary abnormality and insulin resistance in children with familial Mediterranean fever: is there any relationship between vascular changes and insulin resistance?

#### Ismail Dursun^1^, Sebahat Tulpar^1^, Sibel Yel^1^, Demet Kartal^2^, Murat Borlu^2^, Funda Bastug^1^, Hakan Poyrazoglu^1^, Zubeyde Gunduz^1^, Kader Kose^3^, Mehmet E. Yuksel^4^, Abdullah Calıskan^4^, Ahmet B. Cekgeloglu^4^, Ruhan Dusunsel^1^

##### ^1^Pediatric Nephrology, Erciyes University, Faculty of Medicine, Kayseri, Turkey; ^2^Dermatology and Venereology, Erciyes University, Faculty of Medicine, Kayseri, Turkey; ^3^Biochemistry, Erciyes University, Faculty of Medicine, Kayseri, Turkey; ^4^Department of Biomedical Engineering, Faculty of Engineering, Kayseri, Turkey

###### **Presenting author:** Ismail Dursun

**Introduction:** Familial Mediterranean Fever (FMF) is an auto-inflammatory disease causing overt or subclinical inflammation likely associated with insulin resistance. Various nail fold capillary changes were described and used to identify several rheumatic disorders such as scleroderma and dermatomyositis. In adults, nail fold capillary abnormality has been described in FMF. however, there is no enough study in children with FMF

**Objectives:** The aim of this study: 1) to investigate whether children with FMF have nail fold capillary abnormalities in both active or/and remission period or not. 2) To assess the insulin resistance in children with FMF in both active or/and remission period of the disease and compare to control group.

**Methods:** Ninety-two patients with FMF, including 25 patients in active period and 67 patients in remission period and 33 apparently healthy children were enrolled in the study. Nail-fold capillary examination was done by dermatologist who was unaware of *subject’s details such as patient or control group.* Groups were compared for inflammatory markers (ESR, CRP and SAA), insulin resistance (HOMA-IR) and nailfold capillary abnormalities. The relationship among the nail fold capillary abnormalities, insulin resistance and inflammatory markers were evaluated.

**Results:** Overt nail fold capillary abnormality was found in 20% of patients at the attack period and in %2.9 of patients in remission. There was no nail fold capillary abnormality in control group. HOMA-IR was higher at the attack period than attack free period and control. But this did not reach statistical significance. However, HOMA-IR was positively correlated with serum AA. Nail fold capillary abnormality was significantly positively correlated with increased levels of acute phase reactants and was significantly negatively correlated with serum HDL levels.

**Conclusion:**

- There are apparent nail fold capillary abnormalities in children with FMF especially in the active period of the disease.

- There is insulin resistance which is likely associated with inflammation.

- Nail fold capillary abnormality become overt in active period of disease as insulin resistance.

**Disclosure of Interest**

None Declared

### P29 Chronic recurrent multifocal osteomyelitis (CRMO): a single centre experience

#### Katerina Bouchalova^1,2^, Jana Franova^3^, Marcel Schuller^3^, Marie Macku^3^

##### ^1^Dpt. of Pediatrics, Palacky University and Faculty Hospital, Olomouc, Czech Republic; ^2^Institute of Molecular and Translational Medicine, Palacky University and Faculty Hospital, Olomouc, Czech Republic; ^3^Pediatric Department, University Hospital, Brno, Czech Republic

###### **Presenting author:** Katerina Bouchalova

**Introduction:** Chronic recurrent multifocal osteomyelitis (CRMO) is a rare autoinflammatory disorder, affecting predominantly girls. To date, the incidence and precise immunological basis of this disorder are unknown. Further, no validated international diagnostic criteria or definition of remission exist. The bisphosphonate, pamidronate is an effective treatment for CRMO. However, there are no markers for predicting either remission or time to terminate this treatment.

**Objectives:** Retrospective analysis of patients with CRMO treated in one pediatric rheumatology centre with respect to diagnostic parameters, therapy and outcome. The specific aim was to assess treatment with pamidronate.

**Methods:** The data on CRMO patients treated in the Pediatric Rheumatology Unit, Pediatric Department of the University Hospital Brno, Czech Republic between 2011 to May 2016 were analysed. Inclusion criteria were the presence of at least one lesion consistent with chronic osteomyelitis found from imaging techniques, the absence of detectable infection and onset before 18 years of age. Clinically inactive disease was defined as the absence of pain, local or systemic signs of inflammation, including laboratory inflammatory markers. Epidemiological, clinical, laboratory, and radiological data at the time of diagnosis and during follow up were analysed.

**Results:** In total, 20 patients (pts) (15 girls, 5 boys) with CRMO were treated. Median age at symptom onset was 8.9 years (range 2.8 to 14.7 years), median age at diagnosis was 10.4 years (range 4.5 to 17.6 years). The median time from symptom onset to diagnosis was 0.4 years (range 0.1 to 8.0 years). Areas of clinically painful localization(s) were predominantly the lower extremities, followed by clavicle, spine, upper limbs and sternum. There were no other autoinflammatory conditions preceding CRMO diagnosis in the pts history, Crohn’s disease was later diagnosed in one boy. Whole body MRI and biopsy was performed in 7/20 (35%) and 14/20 (70%) patients, respectively. Treatment with NSAIDs monotherapy had been sufficient in 5/20 (25%) pts. Of the remainder, prednisone and MTX had not been effective in terms of the long term outcome. Intravenous pamidronate and etanercept showed good efficacy. Pamidronate was used in 14/20 pts (70%). Overall, substantial improvement was observed in 13/14 (93%) of these patients, leading to termination of pamidronate therapy in 9/14 (64%) due to sustained clinical inactivity to this date. Etanercept was successfully used in two patients (one boy with insufficient effect of pamidronate, and one girl without pamidronate treatment history).

**Conclusion:** Based on short time therapeutic effects and side effects of corticosteroid and MTX treatments, CRMO is currently treated in our centre using NSAIDs and following this treatment failure, pamidronate is used with good results and without serious side effects. The data on therapy outcome should be analysed in a prospective study. Further research should be focused on markers of remission, and an appropriate pamidronate therapy regimen.

**Disclosure of Interest**

None Declared

### P30 A case of R92Q positive traps patient with aseptic meningitis and intracranial hypertension

#### Katerina Theodoropoulou^1^, Raffaella Carlomagno^1^, Annette von Scheven-Gête^1^, Claudia Poloni^2^, Michael Hofer^1^

##### ^1^Pediatric Rheumatology; ^2^Pediatric Neurology, CHUV, Lausanne, Switzerland

###### **Presenting author:** Katerina Theodoropoulou

**Introduction:** Tumor necrosis factor receptor-associated periodic syndrome (TRAPS), formerly known as familial Hibernian fever, is an autosomal dominant autoinflammatory disease, resulting from mutations in the *TNFRSF1A* gene, encoding tumor necrosis factor receptor. Its most common clinical features include recurrent long-lasting fever episodes usually persisting 1–3 weeks, periorbital edema, conjunctivitis, a migratory erythematous skin rash with underlying fasciitis and myalgia, arthralgia or arthritis and polyserositis. Even if headaches are frequently described, no case has been reported until now with documented inflammatory neurological involvement. However, an interesting association has been hypothesized with multiple sclerosis and the R92Q mutation. The R92Q is a TNFRSF1A variant of unclear significance, which has been associated with cases of TRAPS but its actual pathogenic contribution should always be interpreted in the appropriate clinical context.

**Objectives:** To describe a case of R92Q positive TRAPS patient with documented inflammatory neurological involvement.

**Methods:** Case report.

**Results:** 4-year-old girl, originated from Switzerland, presenting with recurrent episodes of fever and gastrointestinal symptoms beginning within the first 2 weeks of life, later on associated with arthralgia, pharyngitis, periorbital lilaceous discoloration and atypical febrile seizures. The patient had a normal growth and development; the family history was unremarkable. Laboratory findings showed intermittent increased levels of acute phase reactants. Imaging studies revealed normal cerebral MRI and abdominal ultrasound. After exclusion of an infectious origin as well as an immunodeficiency, an autoinflammatory disease was suspected. Genetic analysis was performed in the 4 major genes associated with periodic fever syndromes (NLRP3, MEFV, TNFRSF1A, MVK) and the R92Q variant was found in the TNFRSF1A gene suggesting the diagnosis of TRAPS. A treatment with Canakinumab, an IL-1 blocking agent, was introduced with partial response. In spite of the uptitration of its dosage to 6 mg/kg, arthralgia and monthly episodes of fever persisted. Moreover, the patient developed progressive headaches with vomiting and fluctuant level of consciousness. Cerebral MRI was repeated and was normal. Lumbar puncture showed high opening pressure suggesting intracranial hypertension. Analysis of the cerebrospinal fluid reveled pleocytosis with sterile cultures, indicating the presence of aseptic meningitis. The patient experienced resolution of the headaches after the evacuating lumbar puncture, but neurological symptoms rebounded two days later. Canakinumab treatment was, therefore, replaced with Anakinra, another IL-1 blocking agent which seems to be more efficient in treating autoinflammatory neurological symptoms following previous experience in CAPS patients. Acetazolamide therapy was also started to lower intracranial pressure.

**Conclusion:** We report here the first case of TRAPS with documented inflammatory CNS involvement. This observation could suggest that thorough cerebral investigations should be performed in TRAPS patients presenting with persistent headaches or other neurological symptoms. Anakinra, a smaller molecule inhibitor of IL-1 with a better blood brain barrier permeability than Canakinumab, might be considered in these patients.

**Disclosure of Interest**

None Declared

## Poster Session: Bone in rheumatic diseases

### P31 Chronic recurrent multifocal osteitis and Coxiella Burneti infection mimicking adult-onset Still’s disease

#### Laura O. Damian^1^, Dan Cosma^2^, Amanda Radulescu^3^, Dan Vasilescu^4^, Liliana Rogojan^5^, Calin Lazar^6^, Simona Rednic^7^, Mihaela Lupse^3^

##### ^1^Rheumatology, Emergency Clinical County Hospital Cluj, Cluj-Napoca, Romania; ^2^Pediatric Surgery, University of Medicine and Pharmacy Cluj, Cluj-Napoca, Romania; ^3^Infectious Diseases, University of Medicine and Pharmacy Cluj, Cluj-Napoca, Romania; ^4^Radiology, University of Medicine and Pharmacy Cluj, Cluj-Napoca, Romania; ^5^Pathology, Emergency Clinical County Hospital Cluj, Cluj-Napoca, Romania; ^6^1st Pediatric, University of Medicine and Pharmacy Cluj, Cluj-Napoca, Romania; ^7^Rheumatology, University of Medicine and Pharmacy Cluj, Cluj-Napoca, Romania

###### **Presenting author:** Laura O. Damian

**Introduction:** The infectious etiology of chronic recurrent multifocal osteitis (CRMO) is controversial. *Coxiella burneti*, the agent of Q fever, may result in a chronic granulomatous osteomyelitis with a relapsing, multifocal course, but also in reactive osteitis.

**Objectives:** To present a difficult diagnosis of osteitis case in an adult patient.

**Methods:** A 52-yrs lady, general practitioner working in the countryside, presented for prolonged fever with daily spikes, joint pain affecting the wrists and shoulders and discrete evanescent rash on the trunk. She was intaken in the Infectious Diseases department and extensively investigated, with negative results. Echocardiography identified no valvular disease or vegetations.

**Results:** Laboratory showed leukocytosis (12,000 WBC/mcL) with neutrophilia (79%), high CRP (9.6 mg/dL, normal <0.6 mg/dL) liver enzymes and ferritin (760, normal 11-307 mcg/L), and mild thrombocytopenia (124 × 10^3^ Plt, normal 150-400 × 10^3^). She was diagnosed with adult-onset Still’s disease and received colchicine and low-dose prednisone. The fever subsided, but she started to complain of hip pain, and a right hip bone cyst was seen on pelvic radiographs. The alkaline phosphatase, parathormon, vitamin D and quantiferon for tuberculosis yielded normal results. A blood test for *Coxiella burneti* was positive suggesting convalescence after acute Q fever (IgG phase II > 256, with phase I IgM and IgG <128). She received doxycycline and hydroxychloroquine, with general improvement. However, after three months she presented with a dull, persistent shoulder pain. Radiography showed an irregular localised condensing bone lesion on the right humerus, with a sclerotic rim. Bone biopsy revealed non-specific inflammation, with negative cultures. A whole-body MRI showed 3 cm- osteocondensating lesions (T1 and T2 hyposignal) on the right humerus and on the right femur respectively, with no contrast uptake. Coxiella serology was rather compatible to a subsided acute Q fever than to a chronic Q fever (phase II IgM and IgG <32, with IgG phase I <64, IgM phase I <32). A thorough history revealed that her maternal grandmother had been repeatedly treated with antibiotics for osteomyelitis. Sulphasalazine was added to antibiotic therapy, with good results

**Conclusion:** In predisposed individuals *Coxiella* may trigger CRMO. Q fever can mimic many diseases, including adult-onset Still’s disease, and mild thrombocytopenia can be a clue to the diagnosis.

**Disclosure of Interest**

None Declared

### P32 Limping as presenting symptom of osteopetrosis in children

#### Lien De Somer^1^, Pierre Moens^2^, Carine Wouters^1^

##### ^1^Department of Pediatric Rheumatology, UZ Leuven, Leuven, Belgium; ^2^Department of Orthopedic Surgery, UZ Leuven, Leuven, Belgium

###### **Presenting author:** Lien De Somer

**Introduction:** Osteopetrosis is an extremely rare inherited disorder, characterized by an increased bone density and caused by a defect in osteoclasts formation and function. Clinical presentation varies greatly ranging from very early onset bone marrow failure, neurological problems, hearing or vision problems to the incidental finding of osteopetrosis on classical X-ray at a later age.

**Objectives:** To create awareness that osteopetrosis need to be included in the differential diagnosis of a limping child and to demonstrate the usefulness of classical X-ray in the differential diagnosis of limping.

**Methods:** Retrospective review of pediatric cases with limping and on X ray diffuse sclerosis.

**Results:** Two patients of 3 and 5 years old, were referred to our pediatric rheumatology outpatient clinic with limping since 2 months.

The 1^st^ patient is a 5y old girl from consanguineous parents. She presented with persistent limping without fever or previous trauma. Clinically we saw a girl with macrocrania, frontal bossing and hypertelorism. Mobilisation of the left hip was slightly painful and limited in endorotation and abduction. Previously this girl was followed at the neurology department with a discontinue nystagmus and a bombing fontanel where additional imaging revealed a triventricular hydrocephaly with a coronal synostosis. Decompressive craniectomy and the placing of a ventriculo-peritoneal drainage was done before the age of 1y. Afterwards she had several revisions of the ventriculo-peritoneal drain because of recurrence of increased intracranial pressure.

The 2^nd^ patient is a 3y old boy, born from consanguineous parents, that consulted because of painful limping since 2 months without any trauma. Symptomatic treatment partially improved pain symptoms. Clinically we saw a limping boy with macrocrania and hypertelorism, bilateral genu valga and a painful mobilization of the right hip, limited in exo- and endorotation. This boy had a normal psychomotor development and no hearing or vision problems.

Classical X-ray of the hip in both patients showed sclerotic bone with extreme coxa vara and the impression of sliding of the left and right femoral head respectively. Further imaging confirmed diffuse sclerosis of the skull, the pelvis and several long bones and “sandwich” vertebrae. None of the patients had signs of bone marrow suppression, nor hypocalcemia. Genetic analysis in the 1^st^ patient revealed a missense substitution in the CLCN7 gene, which is compatible with an intermediate form of osteopetrosis. Genetic analysis of the 2^nd^ patient is ongoing.

**Conclusion:** Limping is a frequent presenting symptom at the pediatric rheumatology outpatient clinic with a broad differential diagnosis. Classical X-ray in the present patients revealed extreme coxa vara with increased bone density in the context of osteopetrosis. Limping may be the only symptom or accompanied by small stature, neurological manifestations, signs of cranial nerve compression, bone marrow failure and/or renal tubular acidosis. This is the first report that describes limping in pediatric patients as a first presentation of osteopetrosis. Diagnosis is important as the prognosis (development of haematological problems, blindness, hearing problems and neurological complications) is determined by the underlying genetic defect and therefore also the treatment strategy. Treatment varies from merely symptomatic to haematopoeitic stem cell transplantation (HSCT) in case of bone marrow failure.

**Disclosure of Interest**

None Declared

### P33 Markers of bone metabolism: are they useful to predict risk of low bone mineral density in juvenile idiopathic arthritis paediatric patients?

#### Rocio Galindo Zavala^1^, Laura Martín Pedraz^2^, Esmeralda Núñez Cuadros^1^, Gisela Díaz-Cordovés Rego^3^, Antonio L. Urda Cardona^2^

##### ^1^Paediatric Rheumatology, Universitary Regional Malaga Hospital, Malaga, Spain; ^2^Paediatrics, Universitary Regional Malaga Hospital, Malaga, Spain; ^3^Rheumatology, Universitary Regional Malaga Hospital, Malaga, Spain

###### **Presenting author:** Rocio Galindo Zavala

**Introduction:** Serum type I collagen N-propeptide (P1NP) and crosslinked C-terminal telopeptide (CTX) are formation and resorption bone markers respectively. Multiple studies have demonstrated that they are not useful for osteopenia screening in adults. Nevertheless, bone modeling (formation without resorption) is a children exclusive process that could justify a different behaviour of these markers in Paediatrics.

Juvenile idiopathic arthritis (JIA) paediatric patients are known to have higher low bone mineral density (LBMD) prevalence than healthy controls. Bone markers could be indicators of bone involvement in them and contribute to detect children at risk for developing such disease.

**Objectives:** - Analyse serum P1NP and CTX usefulness in the screening of JIA paediatric patients at risk for developing LBMD.

- Evaluate the relationship between these two bone markers, checking if there are some differences from children to adults.

**Methods:** Observational cross-sectional study in JIA Spanish patients from 5 to 16 years old, monitored by a Pediatric Rheumatology Unit between July 2014 and July 2015. Monoarticular forms and patients with other chronic diseases or toxic-to-bone treatments different from those indicated for JIA were excluded.

We considered LBMD when bone mineral density (BMD) Z-score adjusted for age (ZAH) was lower than -2 without pathological fractures.

Anthropometric, clinical and treatment data were recorded. DXA and serum P1NP and CTX were obtained. BMD Z-score was adjusted for sex, age and height in all patients.

**Results:** 94 children participated. Their characteristics are included in Table 19. Only 1 patient suffered from LBMD (1.06%) None of them had osteoporosis.

High positive correlation was found between P1NP and CTX values (Rho 0.75; p < 0.001)

P1NP correlated negatively with HAZ (Rho -0.212; p 0.046), but no relation was found between CTX and HAZ parameters

For a cut point of P1NP of 512.6 ng/ml, we obtained a result of 72% for sensibility and 49% for specificity for the detection of HAZ lower than -1.

**Conclusion:** Bone metabolism markers do not seem to be useful in the detection of JIA paediatric patients at risk for developing LBMD. The low prevalence of LBMD in our sample could contribute to these results, thus making necessary more studies to confirm them. The connection between P1NP and CTX values shows that their behaviour could be similar for children and adults.’ > score adjusted for age (ZAH) was lower than -2 without pathological fractures.

**Disclosure of Interest**

None Declared

**Table 19 (abstract P33).** Anthropometric, clinical and treatment characteristics

Patient’s characteristics (N = 94)		
Gender (Male), n (%)		33 (35,1)
Age (years), median (IR)		11,42 (8,58–13,67)
Z-score BMD adjusted for height, mean (±SD)		0,04 (±0,94)
DISEASE CHARACTERISTICS (N = 94)		
JIA subtype, n (%)	Systemic	10 (10,6)
	Oligoarticular	53 (56,4)
	Polyarticular	22 (23,4)
	Others	9 (9,6)
Disease duration (years), median (IR)		6,12 (3,22–9,10)
RECEIVED TREATMENTS (N = 94)*		
Systemic GC, n (%)		84(89,4)
Synthetic/biological DMARDs treatment, n (%)		42 (44,7)/29 (30.9)

### P34 Low bone mineral density in juvenile idiopathic arthritis: prevalence and related factors

#### Rocio Galindo Zavala^1^, Laura Martín Pedraz^2^, Esmeralda Núñez Cuadros^3^, Gisela Díaz-Cordovés Rego^1^, Antonio L. Urda Cardona^4^

##### ^1^Paediatric Rheumatology, University Regional Hospital of Malaga, Malaga, Spain; ^2^Paediatrics, Malaga University Regional Hospital, Malaga, Spain; ^3^Paediatric Rheumatology, Malaga University Regional Hospital, Malaga, Spain; ^4^Paediatrics, University Regional Hospital of Malaga, Malaga, Spain

###### **Presenting author:** Rocio Galindo Zavala

**Introduction:** Dual energy X-ray absorptiometry (DXA) is the preferred method for assessing bone mineral density (BMD). Since 2013 International Society for Clinical Densitometry has suggested that Z-score BMD in children should be adjusted for height.

Studies have demonstrated that juvenile idiopathic arthritis (JIA) patients have lower BMD than healthy controls. Factors that negatively influence BMD in JIA are not completely understood.

At present there are no researches that evaluate the prevalence of low BMD in JIA Spanish pediatric patients following current recommendations.

**Objectives:** To evaluate low BMD in JIA pediatric patients in Spain following the last recommendations and to asses involved factors.

**Methods:** Observational cross-sectional study in JIA Spanish patients from 5 to 16 years old, monitored by a Pediatric Rheumatology Unit between July 2014 and July 2015. Monoarticular forms and patients with other chronic diseases or toxic-to-bone treatments different from those indicated for JIA were excluded. Anthropometric, clinical and treatment data were recorded. DXA (measuring bone and fat mass), bone metabolism parameters and diet and exercise interview were obtained. BMD Zscore was adjusted for sex, age and height in all patients.

**Results:** 94 children participated. Their characteristics are recorded in Table 20. None of them had osteoporosis. The population prevalence estimation of low BMD was lower than 3%, with a confidence interval of 95%

In multiple linear regression analysis we found a model with R^2^ = 0.4, with significant correlation between BMD Z-score adjusted for height (ZAH) and lean mass index (LMI) (g/m^2^) (B:0.14; p:0.03), body mass index (BMI) percentile (B: 0.024; p < 0.01); height percentile (B:-0.09; p < 0.01) and fat mass index (FMI) (g/m^2^) (B:-0.135; p < 0.01). We found no relation between BMD ZAH and other studied parameters.

**Conclusion:** Low BMD prevalence in our population is lower than previously described in other studies. Different dietary and cultural habits and an unequal access to therapeutic resources could explain these results.

Higher values of LMI and BMI seems to be a protector factor to the bone, while a higher FMI acts as a risk factor for the development of low BMO for chronological age in JIA paediatric patients as it has been described in similar research in other countries.

Influence of diet, physical exercise, inflammatory activity and treatment with glucocorticoids and synthetic and biologic disease-modifying antirheumatic drugs (DMARDs) could not be demonstrated, probably because of a low sample size, because their effects might be scarce and because of the low prevalence of low BMD in our sample.

**Disclosure of Interest**

None Declared

**Table 20 (abstract P34).** Anthropometric, clinical and treatment characteristics

Patient’s characteristics (N = 94)		
Gender (Male), n (%)		33 (35,1)
Age (years), median (IR)		11,42 (8,58–13,67)
Z-score BMD adjusted for height, mean (±SD)		0,04 (±0,94)
Disease characteristics (N = 94)		
JIA subtype, n (%)	Systemic	10 (10,6)
	Oligoarticular	53 (56,4)
	Polyarticular	22 (23,4)
	Others	9 (9,6)
Received treatments (N = 94)*		
Systemic GC, n (%)		84(89,4)
Synthetic/biological DMARDs treatment, n (%)		42 (44,7)/29 (30.9)

### P35 Evaluation of bone metabolism in juvenile idiopathic arthritis: biochemical bone turnover markers and bone density parameters

#### Ilaria Dal Forno, Sara Pieropan, Ombretta Viapiana, Davide Gatti, Gloria Dallagiacoma, Paola Caramaschi, Domenico Biasi

##### Rheumatology, Policlinico G.B.Rossi, Verona, Italy

###### **Presenting author:** Sara Pieropan

**Introduction:**

Juvenile Idiopathic Arthritis (JIA) is a heterogeneous group of diseases characterised by arthritis of unknown origin with onset before age of 16 years. Osteoporosis is characterized by skeletal fragility and microarchitectural deterioration with subsequent increased incidence of fractures. Osteoporosis in pediatric age is diagnosed in the presence of a low BDM score (a z- score of -2 or less) together with at least one clinical rilevant fracture.

**Objectives:**

Evaluate bone turnover markers, calcium-phosfate metabolism, WNT system control, bone mineral density with DXA in a population of children affected by JIA followed at Verona’s Univerity Hosptial in comparison with a normal healthy control population

**Methods:**

37 children (F:M = 25:12, mean age 13,3 ± 4,7 years) affected by JIA (diagnosis according to ILAR classification) underwent evaluation of complete bone metabolism with lombar/femoral DXA and blood analysis (calcium, phosfate, albumin, PTH, 25OH-vitD, ALP, PINP, CTX, DKK1 and Sclerostin). Blood results were compared with the control group (49 children) matched according to age, sex and auxologic parameters. Physical examination and medical records were compared including drug administration.

**Results:**

Vit D levels had an inverse correlation with PTH, ALP and CTX. Patients treated in the past with DMARDs had lower levels of Vit.D. There is statistical significant correlation between NSAIDs and either lower levels of ALP/PINP or higher levels of CTX. Previous systemic steroidal therapy correlates with higher levels of DKK1.

A positive correlation statistically significant at the upper limits was found between DKK, PINP and CTX. Either vertebral or femoral Z-score correlated with Vit. D levels (p = 0,026; 0,026 e 0,038 respectively at the lumbar vertebral site, at femoral bone and at the head of the femoral bone). Femoral z-score at both sites is correlated with duration of illness and with PTH levels. No statistically significant bioumoral and densitometric differences between the poli-oligo articular JIAs were found.

**Conclusion:**

Low BMD is present in JIA children who have a higher prevalance of hypovitaminosos D and higher levels of DKK1.hypovitaminosos D should promptly be treated. More data are needed to provide recommendations for the evaluation and treatment of osteoporosis in these patients

**Disclosure of Interest**

None Declared

## Poster Session: Imaging

### P36 Pediatric musculoskeletal ultrasound – age- and sex-related normal B-mode findings of the achilles tendon

#### Daniel Windschall^1^, Ralf Trauzeddel^2^, Hartwig Lehmann^3^, Gerd Ganser^4^, Rainer Berendes^5^, Maria Haller^6^, Manuela Krumrey-Langkammerer^7^, Antje Nimtz-Talaska^8^, Philipp Schoof^9^, Ralf Felix Trauzeddel^10^, Christine Nirschl^1^

##### ^1^Asklepios Hospital Weissenfels, Weissenfels, Germany; ^2^Helios Kliniken Berlin Buch, Berlin, Germany; ^3^University of Giessen and Marburg, Giessen, Germany; ^4^St. Joseph Stift, Sendenhorst, Germany; ^5^Kinderkrankenhaus St. Marien, Landshut, Germany; ^6^Kinderärztliche Praxis Gundelfingen, Gundelfingen, Germany; ^7^German Center for Pediatric Rheumatology, Garmisch-Partenkirchen, Germany; ^8^Kinderärztliche Praxis Frankfurt/Oder, Frankfurt/Oder, Germany; ^9^Kinderärztliche Praxis, München, Germany; ^10^Department of Anesthesiology and Intensive Care Medicine, Campus Charité Mitte and Campus Virchow-Klinikum, Berlin, Germany

###### **Presenting author:** Daniel Windschall

**Introduction:** Musculoskeletal ultrasound (MSUS) is an important tool for evaluating disease activity, therapeutic progress, and remission status of rheumatic diseases in children. Knowledge of age-related normal findings is essential when interpreting pathological findings such as those seen in juvenile idiopathic arthritis. In juvenile spondyloarthritis and in the JIA category enthesitis-related arthritis the affection of the Achilles tendon is very common.

**Objectives:** To evaluate normal findings and sizes of the Achilles tendon (AT), we recorded age-related grey-scale findings in 438 healthy children between 1 and 18 years of age using high-resolution B-mode MSUS

**Methods:** We determined approximate age- and sex-related norms for the AT (sagittal and transverse diameter) at two predefined positions (middle part at the end of the tibia and distal part at the beginning of the calcaneal back). Ultrasound measurements were performed in the longitudinal and tranversal plane.

**Results:** 438 probands (243 girls) were bilaterally examined. 6 age groups were created for analyses. In the youngest age group (1-3 years) the mean sagittal diameter was 3.4 mm (middle part boys), 3.3 mm (middle part girls), 2.8 mm (distal part boys) and 2.9 mm (distal part girls). In the oldest age group (16-18) the sagittal diameter was 4.4 mm (middle part boys and girls), 4.2 mm (distal part boys) and 4.1 mm (distal part girls). Tendon thicknesses (sagittal and tranverse diameter) showed positive correlations with age (p < 0.01).

**Conclusion:** We report age and sex-related normative data of the Achilles tendon analyzed by a high sample size of healthy children.

**Disclosure of Interest**

None Declared

### P37 Usefulness of ultrasound joint score in the assessment of clinical activity in children with juvenile idiopathic arthritis

#### Estefania Quesada-Masachs^1^, Carla Aguilar Blancafort^2^, Sara Marsal Barril^3^, Consuelo Modesto Caballero^4^

##### ^1^Paediatric Rheumartology Unit, Vall d’Hebron University Hospital, Barcelona, Spain; ^2^Clinical Research Support Unit, Vall d’Hebron Research Institute, Barcelona, Spain; ^3^Rheumatology, Vall d’Hebron University Hospital, Barcelona, Spain; ^4^Paediatric Rheumatology Unit, Vall d’Hebron University Hospital, Barcelona, Spain

###### **Presenting author:** Estefania Quesada-Masachs

**Introduction:** Juvenile idiopathic arthritis (JIA) is a disease with a prognosis that has greatly improved in recent years due to the introduction of new therapies and guidelines. Musculoskeletal ultrasound (US) evaluation could be a useful tool for a comprehensive evaluation of these patients.

**Objectives:** To compare clinical and US evaluation in the assessment of joint synovitis in children with JIA.

**Methods:** The study included 64 children with JIA according to the ILAR criteria, all of them receiving biologic antiTNF therapy +/- methotrexate (MTX) or MTX alone. Clinical and US examination was performed by 2 different paediatric rheumatologists at baseline, 6 and 12 months. Demographic and disease characteristics were recorded at baseline. All joints were clinically assessed for swelling, tenderness and limited range of motion. JADAS10/27/71, cJADAS, JADAS-PCR, disease activity state, parent’s VAS, patient’s VAS, physician’s VAS, CHAQ, ESR, and CRP were recorded every 6 months. A previously validated US score was used. The score included 10 joints:knee, ankle, elbow, wrist, and 2nd metacarpophalangeal (MCP) joint bilaterally. Each joint was scored for greyscale (GS-US) synovitis and power Doppler signal (PD-US) according to a 4-point semi-quantitative scale (range 0-3). The sum of all GS values was transformed into a new US-score:GS score (range 0-30). The same method was used to calculate the PD score (range 0-30). The P value was calculated using the Fisher exact test or Kruskal-Wallis test, and the correlation was analyzed using Spearman coefficient.

**Results:** A total of 12,496 joints were clinically assessed:41 were found to be active (swollen or tender and limited), 34 were swollen, 27 tender and 37 limited. Applying the ultrasound score, 1,710 joints were assessed both clinically and by US. On GS-US evaluation, 57 joints scored ≥ 1 (3.33%) and on PD-US, 10 joints scored ≥ 1 (0.58%). A total of 1,675 joints were clinically inactive: 34 (2.02%) had subclinical synovitis by GS-US and 3 (0.18%) by PD-US. Most of the 34 joints with GS-US subclinical synovitis were judged to have mild effusion/hypertrophy (79.41%). The joint mostly commonly affected, both clinically and by US, was the knee (4.39% vs 6.43% of the explored knees, respectively). Interestingly, the joint with the greatest clinical-US discordance was also the knee (5.55% discordance), followed by the wrist and ankle. The joint with the lowest discordance (0.58%) was the 2nd MCP. The GS score and PD score were significantly higher in patients with active disease compared with patients with inactive disease (p = 0.000 and p = 0.017, respectively). The GS score was significantly changed (p < 0.05) with the following variables:physician’s VAS, parent’s VAS, CHAQ, ESR, number of limited, swelling, tender, and active joints, JADAS10/27/71, cJADAS, JADAS-PCR, and active disease status. The PD score was significantly changed (p < 0.05) with the same variables, except for CHAQ and ESR. The GS score was moderately correlated with physician’s VAS, number of active, swelling, limited and tender joints, JADAS10/27/71, cJADAS and JADAS-PCR, but the PD score showed no correlation with any of the clinical variables studied.

**Conclusion:** Subclinical synovitis detected by US is not uncommon in children with JIA. However, US scores changed significantly, mirroring most of the clinical variables. US scores could help physicians to determine the inflammatory activity of JIA more accurately. However, GS and PD US of the joints previously involved seem to be more useful than the comprehensive US score.

**Disclosure of Interest**

None Declared

### P38 The prevalence of sacroiliitis assessed by magnetic resonance imaging in a pediatric population with inflammatory bowel disease

#### Francisca Aguiar^1^, Rita Fonseca^1^, Duarte Alves^2^, Ana Vieira^3^, Alberto Vieira^2,3^, Jorge A. Dias^2,4^, Iva Brito^2,5^

##### ^1^Rheumatology, Centro Hospitalar São João, Oporto, Portugal; ^2^Faculty of Medicine of Porto University, Porto, Portugal; ^3^Radiology, Centro Hospitalar São João, Porto, Portugal; ^4^Pediatric Gastroenterology, Centro Hospitalar São João, Porto, Portugal; ^5^Pediatric Rheumatology Unit, Centro Hospitalar São João, Porto, Portugal

###### **Presenting author:** Francisca Aguiar

**Introduction:** The axial articular involvement in pediatric inflammatory bowel disease (IBD) is frequent and often asymptomatic. The magnetic resonance imaging (MRI) is an important instrument in evaluating disease activity in these patients. Occasionally, in these exams, inflammatory lesions suggesting sacroiliitis are identified incidentally. However, no studies have evaluated the sacroiliac joints assessed by MRI colonography or enterography performed in children with IBD.

**Objectives:** To study the prevalence of asymptomatic sacroiliitis in a population of children diagnosed with IBD. As a secondary aim, we intended to identify demographic and clinical variables associated with the presence of sacroiliitis in this population.

**Methods:** A cross-sectional study, including pediatric patients diagnosed with juvenile IBD that had previously undergone MRI colonography or enterography. Two independent radiologists proceeded, independently and blindly, to MRI analysis and identification of lesions suggestive of sacroiliitis. Parametric and non-parametric tests were used in statistical analysis in comparison of clinical and demographical variables in patients with or without sacroiliitis in MRI.

**Results:** 64 patients were included, 24 (37.5%) females, with a mean age of 15.1 ± 2.8 years. 54 (84.4%) had Crohn’s disease and 10 (15,6%) with ulcerative colitis. Mean age at IBD diagnosis was 12.1 ± 3.6 years and median disease duration 2.77 [0-10.5] years. The prevalence of abnormalities suggestive of sacroiliitis in MRI was 31.2% (n = 20). 17 of those patients (26.6%) presented periarticular hypersignal, which was bilateral in 12 (18.8%) of them and unilateral in 5 (7.8%). Among 3 (4.7%) patients, the MRI abnormalities included, not only periarticular hypersignal, but also cortical irregularities and subchondral cysts. Regarding the prevalence of sacroiliitis, statistically significant differences were found associated with female gender (45.8% vs 22.5% of positive MRI in females and males, respectively, p = 0.048) and those with positive MRI had shorter disease duration (1.7 [0-10.5] vs 3.2 [1-9.7] years, p = 0.001). Other factors such as the type of IBD, surgical history, age and age at diagnosis were not associated with the presence of sacroiliitis.

**Conclusion:** Our study showed that there is a high prevalence of asymptomatic sacroiliitis in children with IBD (31.2%). MRI colonography or enterography, held under the monitoring of the underlying disease, may contribute to the early identification of these patients.

**Disclosure of Interest**

None Declared

### P39 Specificity and sensitivity of salivary glands ultrasonography in juvenile Sjőgren’s syndrome

#### Gordana Susic^1^, Vera Milic^1,2^, Goran Radunovic^1,2^, Ivan Boricic^2,3^

##### ^1^Institute of Rheumatology, University of Belgrade, Belgrade, Serbia; ^2^Medical faculty, University of Belgrade, Belgrade, Serbia; ^3^Institute of pathology, Belgrade, Serbia

###### **Presenting author:** Gordana Susic

**Introduction:** Juvenile Sjőgren’s syndrome (JSS) is uncommon systemic connective tissue disease (SCTD) in childhood, with unic clinical features and various extraglandular manifestation, which differs from adult form. In last several years salivary glands ultrasound (SGUS) seems to be helpful diagnostic tool.

**Objectives:** was to assess sensitivity and specificity of salivary glands ultrasound (SGUS) for diagnosis of JSS. Gold standard for diagnosis od JSS was consensus of experienced pediatric rheumatologist and ophthalmologist. In all suspected cases confirmatory biopsy of minor salivary glands (MSGB) was performed.

**Methods:** We conducted cross-sectional study which included 33 consecutive pts. with suspected JSS: 9 pts. with primary JSS (8 F, 1 M), 15 pts. with secondary JSS (11 pts with JIA, 1 SLE, 1 MCTD, 2 with undifferentiated CTD), 4 pts. with keratoconjuncivitis sicca, 5 pts. with chronic reccurent parotitis, and 12 healthy controls. Diagnosis of primary JSS was establiched according to the American-European Consensus Group (AECG), confirmed in all with MSGB (grade IV by Mason Chisholms). Ultrasonograhy examination was performed by one clinician with more then 10 years expirience in US, blindeed to the clinical examination. Ultrasound scoring system (De Vita et al.1992.) was used for grading abnormalities of salivary gland (both pair parotid and submandibular). The following ultrasound parameters were analized: glands size, parenhymal echogenicity, parenchymal inhomogenicity, posterior gland border, and focal changes.

**Results:** Mean age of patients with pJSS was 15,3 yrs (9-20), disease duration 4,47 (1,25-10) yrs. None of patients with JSS had normal parotid homogenicity, 3/9 had mild, 1/9 evident and 5 (55,6%) gross inhomogenicity (p < 0,001 comparing with other goups). Submandibular inhomogenicity was more prevalent in JSS group – 66,7% of patients had evident or marked parenchymal abnormality (p > 0,01).

Results of receiver operating characteristic (ROC) curve analysis showed that area under the curve (AUC) was 0,853 [95% confidence interval (CI) 0,730-0,977] for inhomogenicity of parotid glands and 0,745 (CI 0,527-0,964) for inhomogenicity of submandibular glands. The cutt-of score ≥2 for parameters mentioned above allowed us to obtain the best ratio between sensitivity and specificity for diagnosis of JSS. The parotid glands inhomogenicity achieved sensitivity 66,7% and specificity 77,2%. In a case of the submandibular inhomogenicity, sensitivity and specificity were 66,7% and 83,3%, respectively. Glands size, posterior border, and focal changes showed poor diagnostic performance.

**Conclusion:** Salivary glands ultrasonography showed good accuracy as a non-invasive method in diagnosis of JSS. It is necessary to expand our research to a larger group of patients for obtaining its exact position in diagnostic algorithm.

**Disclosure of Interest**

None Declared

### P40 Conventional radiography in juvenile idiopathic arthritis: joined recommendations from the French societies for rheumatology, radiology, paediatric rheumatology

#### Pauline Marteau^1^, Catherine Adamsbaum^2^, Linda Rossi-Semerano^3^, Michel De Bandt^4^, Irène Lemelle^5^, Chantal Deslandre^6^, Tu Anh Tran^7^, Anne Lohse^8^, Elisabeth Solau-Gervais^9^, Pascal Pillet^10^, Brigitte Bader-Meunier^11^, Julien Wipff^12^, Cécile Gaujoux-Viala^13^, Sylvain Breton^14^, Valérie Devauchelle-Pensec^15^

##### ^1^Rheumatology, CHU Cavale Blanche, Brest, France; ^2^Paediatric Radiology, AP-HP Bicêtre, France; ^3^Paediatric Rheumatology, AP-HP, Hôpital Bicêtre, Paris, France; ^4^Rheumatology, CHU Martinique, Fort de France, France; ^5^Rheumatology, University Hospital, Nancy, France; ^6^Paediatrics, Hôpital Robert Debré, Paris, France; ^7^Paediatrics, University Hospital, Nîmes, France; ^8^Rheumatology, Hospital Centre of Belfort Montbeliard, Belfort, France; ^9^Rheumatology, University Hospital of Poitiers, Poitiers, France; ^10^Paediatrics, Hôpital Pellegrin, Bordeaux, Bordeaux, France; ^11^Paediatric Rheumatology, Hôpital Necker, Paris, France; ^12^Rheumatology, Hôpital Cochin, Paris, France; ^13^Rheumatology, University Hospital, Nîmes, France; ^14^Paediatric Radiology, Hôpital Necker, Paris, France; ^15^Rheumatology, Hôpital Cavale Blanche, Brest, France

###### **Presenting author:** Pauline Marteau

**Introduction:** Juvenile idiopathic arthritis (JIA) is a group of erosive inflammatory rheumatisms, responsible of joint damage. General guidelines concerning imaging in JIA were recently published by the EULAR-PReS task force. Though, specific recommendations about conventional radiography (CR) at diagnosis and follow-up in each subtype of JIA are still lacking.

**Objectives:** To provide guidelines concerning CR, at diagnosis and follow-up, in every subtype of JIA (exlusion of systemic JIA), that can be used in clinical practice.

**Methods:** A multidisciplinary task force of 14 French rheumatologists, paediatricians, radiologists, and two patients representatives was convened. Following the GRADE method for the elaboration of recommendations, they formulated a series of research questions according to the PICO process. Systemic JIA, as a specific entity whose course and prognosis differ from the other categories of JIA, was ruled out. An exhaustive literature review was performed by one junior investigator (MP) using MEDLINE and EMBASE. The keywords “juvenile idiopathic athritis”, “juvenile chronic arthritis”, “juvenile rheumatoid arthritis”, “juvenile psoriatic arthritis”, “enthesitis-related arthritis”, “juvenile spondyloarthritis/spondylarthropathy”, OR “radiography”, “X Ray” OR “radiographic assessment” were used, and completed by a manual search. We considered articles in English describing structural damage (ca, joint space narrowing, erosions, growth abnormalities). Then, several assessments were proposed, following evidence-based data when available, and expert opinion. The draft was submitted for evaluation to a group of 14 independant french-speaking reviewers. Final guidelines then underwent a second round of reviewing by the two groups of experts, with evaluation by a numeric scale according to a Delphi process.

Results:

Of over 900 publications identified initially, 117 underwent full-text review, and 72 were included. One poster was also considered along with one online national recommendation. For sructural damage, most studies considered JIA as only one entity or are based on polyarticular forms of JIA. The task force produced four general statements, and twenty-seven recommendations about CR at diagnosis and follow-up in the different subsets of JIA . The Delphi process was under progress at submission of this abstract. We recommend systematic CR of hands, wrists and feet at diagnosis and follow-up when the structural threat is high. In the oligoarticular form, CR should be based on clinical justification. CR plays a minor part in axial imaging.

**Conclusion:** Few publication have evaluated structural damage in JIA. Polyarticular and extensive oligoarthritis were the most studied. CR are frequently neglected in JIA, however, they are accessible and the less expensive imaging procedure. Hoowever they are of prime nterest in polyarticular subtype. Ultra sound and magnetic resonnance imagery will probably take an increasing part in diagnostic and therapeutic decisions but studies are also needed.

**Disclosure of Interest**

None Declared

### P41 Monitoring disease severity in experimental arthritis by imaging of phagocyte migration in vivo

#### Sandra Gran^1^, Olesja Fehler^1^, Stefanie Zenker^1^, Michael Schäfers^2^, Johannes Roth^1^, Thomas Vogl^1^

##### ^1^Institute of Immunology, Münster, Germany; ^2^European Institute for Molecular Imaging, Münster, Germany

###### **Presenting author:** Sandra Gran

**Introduction:** Recruitment of phagocytes into infected or inflamed tissue is the very first and crucial event occurring in the onset of an inflammation. Therefore, targeting and modulation of phagocyte extravasation seems to be a promising new approach to fight inflammatory disorders and diseases, such as rheumatoid arthritis. Directed non-invasive tracking of phagocyte migration to the site of inflammation would furthermore extend our knowledge about pathomechanisms of arthritis and allow monitoring of local disease activity *in vivo*.

**Objectives:** The aim of this study was to establish a system to visualize and analyze migration properties of different types of leukocytes under inflammatory conditions *in vivo*.

**Methods:** Murine myeloid progenitor cells were differentiated to neutrophils or monocytes using the ER-HoxB8 system for generation of stable progenitor cell lines (Wang et al., Nat Meth., 2006). Cells were labeled with the membrane-selective fluorescent dyes DIR or DID (Eisenblätter et al., J Nucl Med., 2009). We analyzed viability and functionality of stained phagocytes *in vitro* and investigated their ability to migrate to sites of inflammation *in vivo* in a subcutaneous granuloma mouse model (CG) and in a collagen induced arthritis mouse model (CIA) via fluorescence reflectance imaging (FRI).

**Results:** Labeling of monocytes or neutrophils with DIR or DID did not affect viability or cellular functions like phagocytosis, ROS production, adhesion or transwell migration *in vitro*. Furthermore, FRI allowed the quantitative visualization of directed migration of phagocytes towards an inflammatory stimulus, i.e. endotoxin in the cutaneous granuloma model, *in vivo* with high sensitivity and specificity. In addition, in CIA the amount of immigrated monocytes showed a close correlation to disease score and severity.

Using genome editing techniques we knocked out the adhesion receptors CD18 and VLA-4 (knockout of the α4 subunit of VLA-4). Differential cell labeling and parallel injection allowed direct quantitative comparison of two distinct phagocyte populations within the same animal. This method revealed significant differences in recruitment of wildtype and CD18 and VLA-4 knockout phagocytes *in vivo*.

**Conclusion:** Specific and distinguishable labeling of diverse phagocyte populations (e.g. wildtype versus specific knockouts) allows *in vivo* tracking and subsequent quantification of migrated cells within the same animal. Targeted deletion of specific genes and correlation of the amount of immigrated cells to disease severity offers new opportunities to investigate and monitor inflammatory mechanisms of arthritis at a cellular and molecular level *in vivo*.

**Disclosure of Interest**

None Declared

### P42 Impact of S100A12 proteins quantification and MSUS in treatment management of JIA patients with complete remission on treatment; a pilot study in 13 patients

#### Severine Guillaume Czitrom^1^, Dirk Foell^2^, Dirk Holzinger^2^

##### ^1^Service de Medecine des Adolescents, Chu de Bicetre, Le Kremlin Bicetre Cedex, France; ^2^Klinik für Pädiatrische Rheumatologie, Universitätsklinikum Münster, Münster, Germany

###### **Presenting author:** Severine Guillaume Czitrom

**Introduction:** Measurement of S100A12 proteins in serum might be able to identify JIA children with complete remission (CR), who are at high risk of relapse. However, its sensibility is less than 70%.

**Objectives:** We aimed to test whether musculoskeletal ultrasonography (MSUS) would bring more information on the predictability of the relapse risk.

**Methods:** JIA patients with complete remission on medication (CRM) according to Wallace, and about to interrupt all, or part, of their systemic treatment, were identified in SGC’s cohort and prospectively followed. At day 0 (D0), active joint count, medications, MSUS of former inflammatory joints and their grading [B-mode (BM) and Power Doppler (PD)] according to Szkudlarek, were performed by SGC. S100 A12 proteins quantifications at D0 were done by the DH-DF team. Treatment modifications were adapted to clinical and MSUS results at D0. Clinical exam and MSUS were repeated at Mo 3 and Mo 6. Informed consent were signed for this study.

**Results:** There were 13 JIA patients (9 F/4 M) with CRM including 3 ERA, 6 extended-oligos, 4 polys (1 RF+). Mean age at D0 was 10 years [4-15], mean disease duration was 5.3 years [2.0-9.8]. One patient had a history of uveitis. Four were on MTX, 6 on ETN, 3 on MTX + ETN, for 2 years meanly. All children were in CRM for a mean of 1.1 years. Last ETN injection before D0 was done beyond 14 days meanly.

At D0, 11 patients were in CRM but 2 had clinically flared up. D0 MSUS showed (i) synovitis with BM/PD signals ≤ grade 1 in 7 CRM patients in one or several joints (group1); (ii) BM grade 2/PD grade 0-2 in 4 CRM patients in one or several joints (group2); (iii) BM grade ≥ 2 /PD grade 2 in one or several joints in the 2 clinically relapsing patients (group3). S100A12 levels were meanly of 28,15 ng/ml [3-63] at D0, all below the positivity threshold of 75 ng/ml, including in the 2 relapsing patients. Therapeutic management was decided on clinical and MSUS grading: (i) D0-group1: decrease (n = 2) or interruption of systemic treatment (n = 5); (ii) D0-group2: maintenance of systemic treatment without modification; (iii) D0- group3: intensification and drop out of the study.

Follow-up in the remaining 11 patients showed (i) group1: sustained clinical CR and MSUS BM/PD signals ≤1 at Mo 3 in all 7 patients; treatments were stopped in the last 2 CRM patients of group1. At Mo 6, all 7 patients were still in clinical CR, with MSUS showing sustained BM/PD signals ≤1 in 3/7 patients; further follow-up is on-going; (ii) group2: 3 patients/4 relapsed by Mo 3 precisely on the joints indicated by MSUS signals grade2; they required therapeutic intensification and dropped out of the study; 1 patient had sustained CRM with MSUS BM grade2 /PD grade0 signals at Mo 3 and MRI proved that the lesions were degenerative. Systemic treatment was reduced in this patient; at Mo 6, she was still in CRM and MSUS signals were unchanged, allowing systemic treatment interruption.

**Conclusion:** At the individual level, MSUS can detect BM/PD grade2 synovitis in patients with clinical CRM, which seems to announce a short-term inflammatory relapse in joints. Moreover, this pilot study indicates that MSUS might be more sensitive than S100A12 proteins quantification in identifying individual patients at high risk of relapse. It suggests that MSUS is a excellent tool for JIA patients’ follow-up and therapeutic management.

**Disclosure of Interest**

None Declared

### P43 Sublinical synovitis on us in patients with new-onset juvenile idiopathic arthritis does not predict early appearance of clinical disease except for the ankle joint

#### Stefano Lanni^1^, EH Pieter Van Dijkhuizen^1,2^, Silvia Magni Manzoni^3^, Denise Pires Marafon^3^, Francesca Magnaguagno^1^, Laura Tanturri de Horatio^3^, Nienke M Ter Haar^2^, Annemieke S Littooij^2^, Sebastiaan J Vastert^2^, Fabrizio De Benedetti^3^, Angelo Ravelli^1,4^, Alberto Martini^1^, Clara Malattia^1,4^

##### ^1^Istituto Giannina Gaslini, Genova, Italy; ^2^University Medical Center Utrecht, Utrecht, Netherlands; ^3^Ospedale Pediatrico Bambino Gesù, Roma, Italy; ^4^Università degli Studi di Genova, Genova, Italy

###### **Presenting author:** Stefano Lanni

**Introduction:** Ultrasound (US) is becoming a useful adjunctive tool to clinical evaluation for the assessment of children with juvenile idiopathic arthritis (JIA). Disagreement between clinical and US examination has been shown in some studies. However, the clinical meaning of US-detected subclinical disease still needs to be clarified.

**Objectives:** The aim of the study was to investigate whether subclinical synovitis detected on US at disease onset in JIA is frequent and whether it may predict appearance of clinical synovitis in an early stage of the disease course.

**Methods:** All consecutive patients with new-onset JIA were recruited in this multicenter study**.** The following joints were scanned with US at baseline: elbows, wrists, II and III metacarpophalangeal and proximal interphalangeal (PIP) joints of both hands, knees and ankles. For each joint, synovial hypertrophy (SH), joint effusion (JE) and power Doppler signal inside the area of SH were recorded. Synovitis was defined as the presence of both or either JE and SH. The US examinations were carried out by sonographers experienced in the assessment of children with chronic inflammatory arthritis who were blinded to clinical findings. Patients were assessed clinically at baseline and after 6 months from the enrollment.

**Results:** Overall 50/133 (37.6%) patients had subclinical disease on US at baseline, for a total of 96 joints. The median number of joints per patient with subclinical disease on US was 1 (IQR 1-3). Thirty-eight/50 (76.0%) patients started a systemic medication at study entry. At the follow-up visit 3/38 (7.9%) patients developed clinical synovitis in joints which presented with subclinical disease on US at baseline. This occurred in 4 ankles. The remainder 12/50 (24%) patients were not given any systemic drug nor were injected in the joint showing subclinical disease on US. Of the 16 joints (3 elbows, 1 PIP joint, 4 knees and 8 ankles) of this group of patients with subclinical disease detected on US at baseline, only 2 ankles developed disease activity on clinical grounds within the 6-month follow-up visit.

**Conclusion:** Subclinical disease on US is frequently detected in patients with new-onset JIA. In patients who are not given any systemic drug nor are injected in the joint showing subclinical disease on US, this finding is unlikely to predict the development of clinical involvement over the first 6 months of disease. The change from a subclinical to a clinical disease status may occur in case of detection of subclinical disease on US in the ankle joint, irrespective of the beginning of systemic treatment. Further longitudinal studies are warranted in order to better understand whether the detection of subclinical synovitis on US in patients with new-onset JIA predicts the appearance of clinical disease in a later stage of the disease.

**Disclosure of Interest**

None Declared

### P44 Musculoskeletal ultrasound usefulness in the diagnosis and treatment in pediatric rheumatology

#### Vitor A. Teixeira^1^, Raquel Campanilho-Marques^1,2^, Ana F. Mourão^2,3^, Filipa O. Ramos^1,2,3^, Manuela Costa^1^

##### ^1^Serviço de Reumatologia e Doenças Ósseas Metabólicas, Hospital de Santa Maria, Centro Hospitalar Lisboa Norte, Lisbon, Portugal; ^2^Unidade de Reumatologia Pediátrica, Serviço de Reumatologia e Departamento de Pediatria, Hospital de Santa Maria, Centro Hospitalar Lisboa Norte, Lisbon, Portugal; ^3^Instituto de Medicina Molecular, Faculdade de Medicina de Lisboa., Lisbon, Portugal

###### **Presenting author:** Vitor A. Teixeira

**Introduction:** Paediatric Musculoskeletal Ultrasound (MSK-US) is an evolving diagnostic modality that is changing the perspective of patient care and follow-up in paediatric rheumatology, with important repercussions in the way physicians treat their patients.

**Objectives:** We present the impact of MSK-US in the clinical practice of a Pediatric Rheumatology outpatient clinic in recent years regarding diagnosis, monitoring of inflammatory rheumatic diseases activity and therapeutic decisions.

**Methods:** We included all patients followed in a Pediatric Rheumatology Unit who were assigned to a MSK-US evaluation between May 2013 and December 2015. We reviewed the clinical information regarding reasons for MSK-US request, that were divided into 1) confirmation of joint disease activity in children with a known inflammatory rheumatic disease; 2) local diagnosis at the joint level in patients with either mechanical disorders or for confirmation of arthritis in children without a defined diagnosis; 3) evaluation of response to a specific treatment. We also reviewed the diagnostic and therapeutic changes that were taken by the pediatric rheumatologist following the result of the MSK-US.

**Results:** A total of 67 patients were included and 82 MSK-USs were performed, completing a total of 185 anatomic regions assessed (median: 2). Fifty-five patients (82%) were female. Ages ranged between 12 months and 17 years old (mean age: 10.5 years; median: 10 years). Seventeen children (25.4%) had no diagnosis previous to the MSK-US evaluation. Twenty-six patients (38.8%) had juvenile idiopathic arthritis (JIA), 5 (7.5%) had undifferentiated connective tissue disease (CTD), one had juvenile dermatomyositis, one had juvenile systemic lupus erythematosus and the other patient had morphea. Of the patients with a diagnosis of JIA prior to the MSK-US assessment, 7 (26.9%) were classified by the pediatric rheumatologist has polyarticular JIA (pJIA), all of whom were rheumatoid factor negative, 15 (57.7%) as persistent oligoarticular JIA (oJIA), 3 (11.5%) as entesitis related JIA (ERA) and 1 (3.8%) as undifferentiated JIA. In patients with JIA and CTD the main reason to ask for a MSK-US was to evaluate subclinical synovitis.

In 3 patients with no previous diagnosis the confirmation of subclinical synovitis led to the diagnosis of JIA and was followed by: introduction of DMARDs in one patient, NSAIDs in another, and joint injection with triamcinolone hexacetonide in the last other.

Three (7.9%) patients with persistent oJIA were reclassified as polyarticular JIA (pJIA) and all of these patients underwent treatment changes following MSK-US: two patients (66.6%) started a new DMARD and one (33.3%) was submitted to intra-articular injection of triamcinolone hexacetonide. MSK-US allowed the confirmation of inflammatory joint disease activity in 21 (55.3%) of the JIA patients which resulted in alterations in DMARD treatment in 9 (42.9%) of the patients, consisting of either introduction or increase of DMARD dose or change from oral to subcutaneous methotrexate; 7 (33.3%) were submitted to intra-articular injection of corticosteroid and 28.6% did not have any treatment change. Subclinical synovitis was detected in 4 patients with undifferentiated CTD but in only 1 there was introduction of DMARD and another patient was treated with NSAIDs.

**Conclusion:** Despite some lack of validation and standardization of the pediatric MSK-US examination, the results showed that in our center there is a high reliability of pediatric rheumatologists in this imaging method which has largely contributed to the diagnosis and therapeutic decisions in our clinical practice.

**Disclosure of Interest**

None Declared

### P45 MRI imaging of the temperomandibular joint in children with JIA: a 3 year retrospective study

#### Wafa A. Madan^1^, Orla G. Killeen^2^

##### ^1^National Centre for Paediatric Rheumatology, Our Lady’s Childrens Hospital, Dublin, Ireland; ^2^National Centre for Paediatric Rheumatology, Our Lady’s Children Hospital Crumlin, Dublin, Ireland

###### **Presenting author:** Wafa A. Madan

**Introduction:** The Temporomandibular joint (TMJ) is estimated to be involved in up 70% of children with JIA (Juvenile Idiopathic Arthritis). Pain or reduced oral aperture may represent active synovitis of the joint, but it can be clinically asymptomatic in many. Undetected it can result in undergrowth of the mandible and with bilateral involvement, potentially disfiguring micrognathia. MRI with contrast (MRIc) is the gold standard for detecting active synovitis of the TMJ. Localised intra-articular Injections (IAI) corticosteroid are a well recognised therapeutic option.

**Objectives:**

**1.** To estimate the percentage of active TMJ disease in our JIA population.

**2.** Do plain film Xray changes of TMJ in patients with JIA correlate with MRI findings of active TMJ disease?

**Methods:** All TMJ MRIc reports of JIA patients were retrospectively reviewed from January 2013 to January 2016. Age, gender and subtype of JIA were documented. Time waiting for MRIc to be performed was calculated in months. Active TMJ disease was defined as synovial enhancement/synovial hypertrophy post contrast administration. Plain film reports of any abnormalities were recorded. Sensitivity and specificity figures were evaluated to establish if these changes are predictive of active TMJ disease on MRI and the validity of plain film Xray as a screening test.

**Results:** A total of 32 MRIc TMJ were performed on our patient cohort over this 3 year period. The average age was 12.4 years (range 7-18 yrs), 22(71%) were females and 9(29%) were males. Active TMJ disease was reported in 22(73%) on MRIc. The average waiting time for MRIc was 5.8 months range (0-14 months). 73% (16/ 22) who has had active TMJ disease on MRI had plain film of TMJs. 56% (9 /16) of these patients had plain film abnormalities, including condylar flattening in 78%(7/9), erosion(s) in 11% (1/9) and sclerosis in 11% (1/9). 38% (6/16) had a normal X-ray and in 6% (1/16) the appropriate views were not obtained. The estimated sensitivity of plain TMJ X-rays for active TMJ disease on MRI is 60%, with a positive predictive value of 90%. The specificity of normal TMJ X-ray is 83.3%, with a negative predictive value of 45.5%. This study aimed to evaluate TMJ X-ray as an intiial screening test for active TMJ disease given the average waiting time of almost 6 months for a MRIc. Based on this data : with abnormal TMJ x-ray findings only, 42.9% of the subjects would receive TMJ steroid IAI earlier hence reducing progression to potenially erosive disease, 28.6% of subjects would receive TMJ steroid IAI as is the current practice after the gold standard test of MRIc. 23.8% would not receive steroid IAI as it is not required and 4.8% of subjects (one subject) would receive steroid IAI unnecessarily. One subject was excluded from this estimation as she has had steroid IAI prior to the MRIc and hence was identified as receiving unnecessary treatment (negative MRIc post treatment and positive abnormal X-ray finding).

**Conclusion:** The percentage of active TMJ disease on MRI is 73% in our retrospective study of JIA patients which is comparable to other studies in the literature. Given the delay in accesss to MRIc imaging, plain film TMJ is a reasonable initial screening test for predicating active TMJ disease (sensitivity 60%, PPV =90%) in JIA patients, potentially identifying patients suitable for earlier therapeutic interventions and hence reducing irreversible erosive disease.

**Disclosure of Interest**

W. Madan Employee of: clinical trainee, O. Killeen: None Declared

### P46 Temporomandibular signs ands symptons associated with MRI findings in patients with juvenil idiopathic arthritis

#### Adriana Rodriguez Vidal, Diana Sueiro Delgado, Maria Isabel Gonzalez Fernandez, Berta Lopez Montesinos, Inmaculada Calvo Penades

##### Pediatric Rheumatology, Hospital La Fe, Valencia, Spain

###### **Presenting author:** Adriana Rodriguez Vidal

**Introduction:** Arthritis of the temporomandibular joint (TMJ) can be a manifestation of Juvenile Idiopathic Arthritis (JIA), leading to pain, dysfunction and growth disturbances of the mandible. Contrast enhanced magnetic resonance imaging (MRI) is considered the gold standard to reliably diagnose both acute arthritis and deformation of the TMJ.

**Objectives:** To investigate the association between clinical manifestations and MRI findings in TMJ of patients with JIA.

**Methods:** The data of 84 patients with JIA who had performed a MRI of the TMJ between January 2006 and March 2016 were retrospectively reviewed. Signs and symptoms of the TMJ were collected according the screening protocol suggested by *Wulffraat et al.* The following MRI features were evaluated: TMJ synovial enhancement, effusion and bone marrow edema (acute inflammation); condylar deformity, bone erosions and damaged cartilage (long-standing disease).

The association between JIA subtype, disease duration, age at diagnosis and TMJ signs and symptoms with MRI findings was evaluated using a logistic regression model. Statistical analysis was performed using R software (version 3.2.3). Values of p ≤ 0.05 were considered statistically significant.

**Results:** 84 patients (64% females) were included: 38.1% had polyarticular JIA, 23.6% oligoarticular JIA, 20.2% enthesitis related arthritis, 10.7% systemic JIA, 4.8% psoriatic arthritis and 2.4% undifferentiated JIA. Mean age at disease onset was 7.2 (±4.2) years. The mean disease duration was 5.6 (±4.9) years.

At the time of the TMJ evaluation, 77.4% were being treated with disease modifying antirheumatic drugs and 35.7% with biologic drugs.

TMJ involvement was present on MRI in 58.3% of the patients, and 29.8% had bilateral impairment. 9.6% of the patients without inflammatory involvement had TMJ dysfunction.

All patients included had at least one symptom or sign of TMJ involvement during examination. The most common symptom was pain while eating (38.1%). In relation to signs, we found pain with maximal mouth opening (MMO) in 65.5% of the patients, limited mouth opening in 36.9% and deviation at MMO in 28.6%.

Synovial enhancement was present in 35.7% of the patients, effusion in 23.8%, bone marrow edema in 10.7%, condylar deformity in 32.1%, bone erosions in 10.7% and damage cartilage in 13.1%.

Polyarticular JIA was significantly associated with acute TMJ arthritis (p 0.024). We did not find significant correlation between disease duration and age at diagnosis with TMJ involvement on MRI.

Pain while eating was positively associated with acute TMJ arthritis (p 0.0001), but also with chronic damage (p 0.02). 62.5% of the patients with pain had synovial enhancement and 43.5% had effusion on MRI. Mouth deviation at MMO was also significantly associated with chronic damage on MRI (p 0.0005), so that 66.6% of the patients with mouth deviation had condylar deformity on MRI. A statistical trend of significance was observed between limited MMO and acute TMJ arthritis (p 0.09).

70.5% of the patients included had al least two positive clinical or physical examination items.

**Conclusion:** TMJ involvement is highly prevalent in JIA patients. Nevertheless, in our study, not all symptomatic patients had pathological findings on MRI due to inflammation. Pain while eating was the most common symptom and significantly associated with both acute and chronic damage on MRI. During examination, pain with MMO was the most common sign, but mouth deviation predicts chronic damage on MRI. Using the *Wulffraat et al.* suggested criteria of at least 2 positive scores, we detected TMJ involvement on MRI in 70.5% of the patients. The use of this screening protocol may guide the performance of the MRI in our daily practice.

**Disclosure of Interest**

None Declared

### P47 CACP-syndrome in pediatric rheumatology. Clinical and instrumental diagnostic criteria

#### Aleksey Kozhevnikov^1^, Nina Pozdeeva^1^, Mikhail Konev^1^, Evgeniy Melchenko^2^, Vladimir Kenis^2^, Gennadiy Novik^3^

##### ^1^Children’s Orthopedic and Rheumatologic Department., FSBI “The Turner Scientific and Research Institute for Children’s Orthopedics” under the Ministry of Health of the Russian Federation, Saint-Petersburg, Russian Federation; ^2^Department of Foot Pathology, Neuroothopedics and Systemic Diseases, FSBI “The Turner Scientific and Research Institute for Children’s Orthopedics” under the Ministry of Health of the Russian Federation, Saint-Petersburg, Russian Federation; ^3^Pediatric department n.a. I. M. Voroncov, Saint-Petersburg State Pediatric Medical University under the Ministry of Health of the Russian Federation, Saint-Petersburg, Russian Federation

###### **Presenting author:** Aleksey Kozhevnikov

**Introduction:** Juvenile chronic inflammatory arthropathy are a large heterogeneous group of the joint pathology which characterized by persistence arthritis. The chronic synovitis occur primary source and secondary nature. A number of syndromes maybe have similar features with JIA (SAPHO, PAPA, NAO, MCTO, Farber, CACP and other).

**Objectives:** CACP-syndrome is a rare autosomal-recessive disorder of non-inflammatory joint pathology caused by mutations of the PRG4 (proteoglycan-4, 1q25-31) gene leading to absence or loss of function lubricin (Marcelino et al 1999). Lubricin is a mucinous glycoprotein (~227 kDa) secreted by synovial fibroblasts and superficial zone chondrocytes that provided lubricating surface of the joint (Swann et al., 1985). There are known about 15 disease-associated mutations of the gene. Clinical symptoms are characterized by flexion contractures of the interphalangeal joints (camptodactyly), polyarthropathy and variable occurrence of coxa vara and pericarditis. Onset of the disease usually in early childhood with symmetrical camptodactyly of II, III, IV fingers, which are similar to stenotic ligamentitis. Classic manifestation of the syndrome is chronic synovitis visually manifested like juvenile polyarthritis with symmetric progressive contractures (hips, knees, ankles, elbows and wrists) with absence of morning stiffness, skin hyperthermia, joint swelling and enthesopathy. Mechanism of non-inflammation progressive articular effusion is associated with deficiency of the surfactant system of the joint. This fact determines low activity and less of effectiveness of the anti-inflammatory drugs.

**Methods:** Instrumental picture at first sight meets all the criteria of juvenile arthritis (ILAR) and prescribes like the persistence chronic synovitis with osteoporosis with the absence of traumatic and destructive articular changes. But there are no such typical reaction of the bone like rapid uneven ossification of cartilage model of the epiphysis and erosion of articular surfaces usually visualized at the x-ray image at children suffering from JIA. Moreover long-term course of CACP-syndrome doesn’t lead to formation deep bone erosion and arthrosis unlike at JRA. Anatomic dystrophic changes of the bones usually occur through the progressive effusion and high intra-articular pressure (coxa vara, deformation of caput femoris with large “open” cysts of the acetabulum and other). MRI shows the presence of exudative synovitis of the extremely nature with the absence of chronic proliferative changes and pannus. Synovial fluid is straw-colored, less viscous with minimum of quantity cell (less 0,5*109/l) and level of TNF-α less 50 pg/ml.

**Results:** Infancy camptodactyly and symmetric contractures with entheso-negative non-erosive chronic exudative polyarthritis non-effective to anti-inflammatory drugs in combination with dystrophic bone deformations are main markers of CACP-syndrome.

**Conclusion:** The CACP is a non-inflammatory joint disorder of the group of monogenic diseases. Introduction of the algorithms of examination children with atypical variant course of JIA molecular genetic testing of the gene proteoglycan-4 (PRG4) would allow to optimization approaches in diagnostic and therapy of CACP-syndrome.

**Disclosure of Interest**

None Declared

### P48 Comparing articular cartilage thickness in JIA patients and healthy controls by ultrasonography

#### Betul Sozeri, Aysenur Pac Kısaarslan, Zubeyde Gunduz, Hakan Poyrazoglu, Ruhan Dusunsel

##### Pediatric Rheumatology, Erciyes University, Faculty of Medicine, Kayseri, Turkey

###### **Presenting author:** Betul Sozeri

**Introduction:** Juvenile idiopathic arthritis (JIA) is caused primarily by degeneration of the osseocartilaginous structures, due to the synovial inflammatory process. It is essential to closely monitor structural damage during the disease course. Joint cartilage is easily visualized with high-frequency ultrasonography (US).

**Objectives:** We aimed to compare ultrasound (US) measurements of joint cartilage thickness in 6 joints in children with JIA and healthy controls.

**Methods:** A cross-sectional study of bilateral grey-scale US by high frequency transducer (7.5-10) MHz cartilage thickness of the knee, ankle, and second metacarpophalangeal (MCP) joints was performed in patients with JIA and healthy children aged between 6 and 17 years. Medical records were reviewed for JIA subtype and state of disease. Clinical examination, including routine joint examination was carried. Disease activity was assessed with a parental questionnaire and physical examination by the physician

**Results:** A total of 31 patients (19 boys and 12 girls) as well as 31 healthy controls were included in the study. Nineteen patients had polyarthritis (12 had enthesitis-related arthritis, 4 had extended oligoJIA, 2 rheumatoid-factor negative pJIA, and 1 had rheumatoid-factor negative pJIA) and 12 had persistent oligoarthritis.

Joint cartilage thickness was decreased in the knee, wrist, and second PIP joint in children with JIA compared with the healthy cohort (p < 0.05 for all). Patients with oligoarticular JIA had thicker cartilage than patients with polyarticular JIA. Nine patients and 3 controls were detected active synovitis findings (synovial hyperplasia, joint effusion, and PD signal) in their scanned joints. The patients who has active synovitis had cartilage thickness thinner than others (p > 0,05). Also, we found decreasing cartilage thickness with age and thicker cartilage in boys than in girls (p < 0.05 for all).

**Conclusion:** Children with JIA have reduced cartilage thickness compared with children who do not have JIA, and children with polyarticular JIA have thinner cartilage than children with oligoarticular JIA. In the literature, few data are available about the US evaluation of structural damage in children affected by JIA. Musculoskeletal US are a rapid, safe, accurate and reproducible method for evaluating and monitoring joint changes in JIA.

**Disclosure of Interest**

None Declared

### P49 The comparisons between thermography and ultrasonography with physical examination for wrist joint assessment in juvenile idiopathic arthritis

#### Butsabong Lerkvaleekul^1^, Suphaneewan Jaovisidha^2^, Witaya Sungkarat^2^, Niyata Chitrapazt^2^, Praman Fuangfa^2^, Thumanoon Ruangchaijatuporn^2^, Soamarat Vilaiyuk^1^

##### ^1^Pediatrics, Faculty of Medicine Ramathibodi Hospital, Mahidol University, Bangkok, Thailand, ^2^Radiology, Faculty of Medicine Ramathibodi Hospital, Mahidol University, Bangkok, Thailand

###### **Presenting author:** Butsabong Lerkvaleekul

**Introduction:** There are several ways to assess joint inflammation in juvenile idiopathic arthritis (JIA). At present, physical examination (PE) is the gold standard in detecting arthritis but it is difficult to perform especially in young children. Ultrasonography (US) is another good tool for joint assessment but it is an operator dependent and time consuming. Infrared thermography (IRT), a non-invasive and radiation free imaging method, is a promising tool for arthritis detection. Previous studies showed correlation between joint inflammation and parameters of IRT, including thermographic index (TI), heat distribution index (HDI), and mean temperature (T_mean_). However, there is limited data in using IRT for wrist joint assessment in JIA patients.

**Objectives:** To compare IRT and US with wrist joint examination in JIA patients.

**Methods:** This is a cross-sectional study. JIA patients in Rheumatology Clinic, Ramathibodi Hospital between November 2014 and October 2015 were enrolled into this study. Patients with fever were excluded. There were 15 healthy controls. Wrist joint assessment were performed by IRT, PE, and US, respectively. IRT was placed one meter distance from wrist joint in anterior view with neutral position. The images were processed by ThermaCam Researcher Pro 2.10 program and calculated HDI by Matlab software. T_mean_, which obtained from the region of interest, was the mean temperature on dorsal surface of the wrist joint. US was performed by musculoskeletal radiologists. Gray scale was graded according to 4-point semi-quantitative scoring system and Power Doppler (PD) was graded by using The Outcome Measures in Rheumatoid Arthritis Clinical Trials (OMERACT) definition. Total scores of US in this study were the sum of Gray scale scores and PD scores, which were 6 points. Patients were classified into 2 groups based on clinical examination of wrist joints; non-arthritis and arthritis groups. Descriptive statistics, Mann-Whitney *U* test, Kruskal-Wallis test, ROC analysis, Spearman’s correlation, Kappa agreement were used. This abstract is original and not previously published anywhere.

**Results:** Of 46 patients, 16 patients were in non-arthritis group and 30 patients were in arthritis group. Using IRT, median (IQR) of mean temperature (T_mean_) in arthritis group [31.35 (2.20)°C] were higher than non-arthritis group [30.55 (2.40)°C] and healthy controls [30.10 (1.20)°C] with statistical significance (P < 0.05). When subgroup analysis was done, 23 of 30 patients had mild arthritis and 7 of 30 patients had moderate to severe arthritis. T_mean_ ≥ 31 °C was used as a cut-off level between controls and moderate to severe arthritis, the sensitivity and specificity were 85.7% and 80.0%, respectively. HDI median (IQR) in arthritis group, non-arthritis group, and healthy controls were 0.64 (0.42), 0.66 (0.44), and 0.52 (0.28), respectively with no statistical difference, however; HDI seemed to be higher in moderate to severe arthritis when compared to mild arthritis. US also had high sensitivity (83.3%) and specificity (81.3%) with area under the ROC curve of 0.87 in detecting wrist arthritis if US score ≥ 1. The kappa score between clinical examination and US was good (Kappa = 0.63, p < 0.01). Wrist examination also had correlation with US (0.67, p < 0.01) and T_mean_ (0.36, p = 0.01).

**Conclusion:** US is a good tool for arthritis detection, whereas T_mean_ from IRT is useful for joint inflammation assessment especially in moderate to severe arthritis.

**Disclosure of Interest**

None Declared

### P50 Radiographic evaluation of joint space width compared to cartilage thickness as assessed by ultrasonography in MCP, PIP, and knee joints of children with juvenile idiopathic arthritis

#### Dan Ø. Pradsgaard^1^, Arne Hørlyck^2^, Anne H. Spannow^1^, Carsten W. Heuck^1^, Troels Herlin^1^

##### ^1^Pediatrics, Aarhus University Hospital, Aarhus, Denmark; ^2^Radiology, Aarhus University Hospital, Aarhus, Denmark

**Introduction**: Joint space narrowing (JSN), is a measureable outcome of tissue damage caused by inflammation of the synovium in arthritis, and is a degenerative result of cartilage thinning. Usually conventional radiography is the preferred first choice modality to assess JSN. Within resent year’s ultrasonography have been validated for measurements of joint cartilage thickness in large and small joints in healthy children. Furthermore decreased joint cartilage thickness assessed by ultrasonography has been reported in children with JIA compared to a healthy cohort.

**Objectives**: To correlate measures of cartilage thickness assessed by US to the measures of joint space width (JSW) assessed by computerized radiography of the proximal cartilage site in the 2nd MCP, 2nd PIP, and knee in children with JIA.

**Methods**: Seventy-four children with JIA, aged between 5 and 15 years (median 11.3 yrs), 54 girls and 20 boys were included in the study. Cartilage measures of the MCP and PIP joints were assessed at the midline spot with a longitudinal scan with the joints in 900 flexion, whereas the knee joints were assessed by a suprapatellar transverse scan with the knee in maximal flexion measuring cartilage thickness at two spots: medial and lateral femoral condylar areas. Thus, US assessed only the proximal cartilage sites of the joints, whereas digital radiography assessed the total JSW. Readers of the JSW were blinded to the results of the US assessed cartilage thickness measurements.

**Results**: Onehoundred and thirty-six 2nd MCP, 138 2nd PIP, and 146 knee joints were assessed. We found a high level of agreement between US and radiographic measures of cartilage thickness and JSW with Rho values between r = 0.82 to 0.86 (2nd MCP), r = 0.50 to 0.55 (2nd PIP), and r = 0.52 to 0.81 (knee), p < 0.001 for all eight assessed sites).

**Conclusion**: US measurements of cartilage thickness of only the proximal site of 2nd MCP, 2nd PIP and knee joints, correlated well to radiographic measurements of JSW in finger and knee joints of children with JIA. However, the difficulty of assessing the distal part of the joint cartilage may limit the use of US as a tool in the assessment of JSN.

### P51 Study test match between musculoskeletal ultrasound and physical examination with acute phase reactants (C reactive protein and erythrocyte sedimentation rate) for subclinical synovitis detection in mexican with juvenile idiopathic arthritis

#### Talia Diaz^1^, Fernando Garcia^2^, Lorenia De La Cruz^3^, Nadina Rubio^2^

##### ^1^Paediatrics, Hospital San José Tec de Monterrey, Monterrey, Mexico; ^2^Paediatric Rheumatology, Hospital Universitario “Dr. José Eleuterio González”, Monterrey, Mexico; ^3^Rheumatology, Hospital Universitario “Dr. José Eleuterio González”, Monterrey, Mexico

###### **Presenting author:** Fernando Garcia

**Introduction:** The term JIA encompasses a group of clinically heterogeneous arthritides characterized by a chronic inflammatory process of the synovium and periarticular tissue that can lead to structural damage that is responsible for most disabilities in JIA. The primary goal of managing JIA is early detection of inflammatory activity in order to avoid disability. The implementation of musculoskeletal ultrasound for the detection of subclinical synovitis has significant effect on medical decisions and the most important purpose of their use in clinical practice is to improve the time of diagnosis and monitoring patients for a better quality of life.

**Objectives:** The aim of this study was to investigate and compare the use of musculoskeletal ultrasound versus physical examination plus Erythrocyte sedimentation rate and C reactive protein for the detection of subclinical synovitis in Mexican children with Juvenile Idiopathic Arthritis.

**Methods:** We retrospectively collected data of patients from Pediatric Rheumatology Clinic Zambrano Hellion Hospital and the University Hospital “Dr. José Eleuterio González” with diagnosis or suspicion of Juvenile Idiopathic Arthritis from January 2013 to May 2015. We used medical records, acute phase reactants, musculoskeletal ultrasound (high frequency 8-15Hz). Data were analyzed using SPSS Version 23 with Kappa test between musculoskeletal ultrasound and physical examination, musculoskeletal ultrasound and erythrocyte sedimentation rate & musculoskeletal ultrasound and C reactive protein.

**Results:** Of the 5,562 evaluated joints, 3% were reported with synovitis in musculoskeletal ultrasound, of which only 0.75% had clinical synovitis. Joints most affected: 29.1% knee, 0.25% elbow, 17.1% carpal, metacarpophalangeal and proximal interphalangeal hands 0.07%, metatarsophalangeal and proximal interphalangeal of the feet 22.8%. A low Kappa coefficent of <0.40 was found between musculoskeletal ultrasound (Positive Power Doppler finding) and clinical assessment in all joints except for the knee, in which Kappa value of 0.87 was reported.

**Conclusion:** Minimal agreement between musculoskeletal ultrasound and physical exam, erythrocyte sedimentation rate and C reactive protein was found. Musculoskeletal ultrasound was superior to physical examination to identify synovitis in our population. The knee was the only joint with agreement between musculoskeletal ultrasound versus clinical assessment.

**Disclosure of Interest**

None Declared

### P52 A new tool of disease activity evaluation through-out power doppler ultrasonography and serum vascular markers in juvenile idiopathic arthritis

#### Joanna Świdrowska-Jaros, Elzbieta Smolewska

##### Department of Pediatric Cardiology and Rheumatology, Medical University of Lodz, Lodz, Poland

###### **Presenting author:** Joanna Świdrowska-Jaros

**Introduction:** Ultrasound has been shown to be a sensitive tool for evaluating synovitis in juvenile idiopathic arthritis (JIA). One of the first steps in the course of JIA pathogenesis is synovial angiogenesis.

**Objectives:** To investigate the involvement of angiogenesis markers (VEGF, sVEGF-R1, sVEGF-R2, angiopoetin-1, angiopoetin-2) in early JIA and its possible relevance to disease activity and the degree of ultrasound-detected synovial inflammation and angiogenesis.

**Methods:** Serum levels of vascular markers were measured by ELISA in 62 patients with juvenile idiopathic arthritis (aged 1,5-17) and 30 age-matched healthy controls. Ultrasound examination of inflamed joints was performed in each JIA patient and synovial angiogenesis was assessed by means of Power Doppler ultrasonography (PDUS) and the 4^th^ grade vascularity scale.

**Results:** VEGF and its receptors’ (sVEGF-R1, sVEGF-R2) serum levels were significantly higher in JIA patients (▫▫ < 0.05). The same sequence was observed for ANG -1. We found large variation in serum ANG-2 levels. The PDUS imaging identified increased synovial microvascular blood flow in 54 (90%) examined JIA children.

**Conclusion:** In conclusion, the high correlation between synovial microvascular blood flow, serum angiogenic proteins, and symptoms of synovitis may indicate its important role in pathogenesis of JIA.

**Disclosure of Interest**

None Declared

### P53 Thermal point of care diagnostic tool for measurement of joint inflammation

#### Mirta Lamot^1^, Lovro Lamot^1,2^, Mandica Vidovic^1^, Edi Paleka Bosak^1^, Ivana Rados^1^, Miroslav Harjacek^1^

##### ^1^Clinical Hospital Center Sestre Milosrdnice, Zagreb, Croatia; ^2^University of Zagreb School of Medicine, Zagreb, Croatia

###### **Presenting author:** Lovro Lamot

**Introduction:** Since the dawn of the medicine, heat (lat. calor) is one of the four main signs of inflammation. Although it is recognized more than 2,000 years ago, there is still no reliable, simple and objective method to measure the warmth of inflamed joint, which is therefore most often assessed only by clinical examination, with very low validity and reliability.

**Objectives:** To investigate the use of smartphone attached mobile thermography for the assessment of joint inflammation.

**Methods:** Twenty participants with various forms of arthritis and/or arthralgia participated in this pilot cross-sectional observational study. Average age of the participants was 10 years (2,5 - 17,5). In all of the participants Power Doppler vascularization signal of both knee joints was graded from 0-3 by EULAR certificated instructor in MSUS. FLIR ONE smartphone connected device was used to obtain infrared thermal image of every patient’s knee joint prior to the MSUS examination, without clothes and with same environment conditions (room temperature was set to 20 °C). Acquired images were analyzed using FLIR Tools software for thermal image analysis with automatically set range of temperatures. On every image, both knee joints were selected using manual ellipse measurement tool and the data on average, minimum and maximum temperature inside the circle was collected. Subsequent statistical analysis of selected data was performed with Graphpad Prism software.

**Results:** In sixteen knee joints of nine patients a PD signal was above 0,5 (0,5-2,5), which was defined as an active inflammation, while in twenty-four knee joints of thirteen patients PD signal was 0, which was defined as no inflammation. Average value of average temperature in knee joints with inflammation was 33,72 °C (31,3 °C - 35,4 °C) and in patients without inflammation 29,81 °C (23,9 °C - 31,9 °C), average value of maximal temperature was 34,98 °C (32,7 °C - 36,9 °C) and 31,08 °C (25 °C - 33,3 °C), while average value of difference between maximal and minimal temperature was 4,73 °C (2,6 °C - 7,7 °C) and 2,96 °C (1,2 °C - 5,7 °C) respectively. Statistical analysis revealed significant difference in all of the measurements between knee joints with and without active inflammation (p < 0,05).

**Conclusion:** In our proof of concept study we investigated the usefulness of thermography with FLIR ONE, a cheap, portable and easy to use compact device, in joint inflammation assessment. For objective evaluation, MSUS with Power Doppler scoring of synovial vascularization was used. This method is almost a gold standard in joint inflammation assessment and it effectively measures increased blood flow inside the joint, which is one of the main causes of higher temperature. Preliminary results of our study suggested FLIR ONE thermography can be used as a bedside screening tool for joint inflammation, with a potential in assessing and quantifying degree of inflammation. This is in consistence with the results of a recent study which indicated the described method has a sufficiently high validity and reliability to be recommended for assessment of subclinical inflammation in patients with pressure ulcer and diabetic foot [1]. After confirmation in a larger cohort, our results could facilitate this innovative technique as a tool for joint inflammation assessment in everyday clinical practice.

**References**

1. T. Kanazawa, G. Nakagami, T. Goto, et. al. Use of smartphone attached mobile termography assesing subclinical inflammation: a pilot study. J Wound Care. 2016. Apr;25(4)177–82.

**Disclosure of Interest**

None Declared

### P54 Quantification of dynamic contrast enhanced MRI (DCE-MRI) examinations in juvenile idiopathic arthritis

#### Nikolay Tzaribachev^1^, Polymnia Louka^2^, Romiesa Hagoug^2^, Chiara Trentin^2^, Olga Kubassova^2^, Mark Hinton^2^, Mikael Boesen^3^

##### ^1^PRI - Pediatric Rheumatology Research Institute, Bad Bramstedt, Germany; ^2^Image Analysis, London, United Kingdom; ^3^Department of Radiology, Bispebjerg and Frederiksberg Hospital Copenhagen University, Copenhagen, Denmark

###### **Presenting author:** Nikolay Tzaribachev

**Introduction:** In RA, the value of quantitative DCE-MRI imaging biomarkers of the synovial and bonemarrow volume has been studied whereas similar knowledge is very limited in children with JIA.

**Objectives:** To assess diagnostic and predictive value of single joints quantitative DCE-MRI based imaging biomarkers in JIA as compared to the clinical scores.

**Methods:** In 8 Caucasian (7 girls, median age 13,6 years) polyarticular JIA patients, treated with DMARDs, axial DCE-MRI of the dominant MCP joints 2-5 were performed at months 0, 3 and 6 using 0.2 Tesla Esaote C-Scan. All scans were quantified with Dynamika^TM^ and imaging measures of the inflammatory volume (DEMRIQ-Volume) and the disease activity, reflecting the degree of perfusion/inflammation (DEMRIQ-Inflammation) were calculated. DCE-MRI data were analyzed using Region-of-Interest (ROIs) covering synovium of MCPJ 2-5. DEMRIQ scores were calculated for (ALL) MCP 2-5 together and for every single (SING) MCP joint.

Clinical scores included the total body active joint (AJ) count.

*T*-test was used for potential differences in DEMRIQ scores between the visits (p ≤ 0.05 = statistically significant, p ≤ 0.25 = clinically meaningful). Pearson’s correlation coefficient was used to show any decrease or increase in DEMRIQ over time (r^3^0.80 = very strong, r^3^0.60 = strong, r^3^0.40 = moderate, r^3^0.20 = weak).

**Results:** At 3 and 6 months all patients showed statistically significant and/or clinically meaningful changes in ALL and SING DEMRIQ scores (Table 21).

In 6 patients clinical improvement was found at 3 and 6 months. In 5 of these patients a persistently high or worsening DEMRIQ-Inflammation was correlated with a clinical flare within 2-6 months after the last DCE-MRI.

In one patient, clinical worsening was seen despite DEMRIQ-Inflammation improvement, but that was followed by the clinical improvement over time.

Overall, SING DEMRIQ scores showed a higher percentage of inflammation in the MCP joints compared to the clinical scores.

**Conclusion:** DEMRIQ scores followed clinical disease activity in JIA over time and DEMRIQ can depict subclinical disease activity and DEMRIQ-Inflammation scores seems to predict the clinical flares or improvement irrespective of the clinical presentation at the timepoint of DCE-MRI. . Thus DEMRIQ scores analysed with Dynamika^TM^ could become a supportive tool for disease management and decision making in JIA.

**Disclosure of Interest**

N. Tzaribachev Grant / Research Support from: Pfizer, UCB, Roche, Bayer, P. Louka: None Declared, R. Hagoug: None Declared, C. Trentin: None Declared, O. Kubassova: None Declared, M. Hinton: None Declared, M. Boesen: None Declared

**Table 21 (abstract P54).** See text for description

	ALL MCP (2-5) -DEMRIQ inflammation				Global clinical scores-AJ			Outcome
Patient	Baseline	3 mo	6 mo	Pearson’s correlation	Baseline	3 mo	6 mo	
1	117,83	151,14	135,66	Moderate (+)	13	5	7	Switch DMARD
2	83,16	28,96	64,89	Very strong (-)	8	3	0	Continue DMARD
3	99,28	46,16	26,35	Very strong (-)	9	2	11	Improved later
4	38,28	104,71	87,86	Strong (+)	12	6	0	Flare at 6 mo
5	113,36	102,6	118,39	Moderate (+)	12	4	0	Flare at 6 mo
6	29,28	39,09	58,12	Weak	8	6	0	Flare at 2 mo
7	38,1	19,59	56,47	Strong (+)	13	0	0	Flare at 3 mo
8	50,42	96,76	74,75	Moderate (-)	7	2	0	Flare at 3 mo

### P55 Value of magnetocardiography for diagnosis of myocardial damage in children with rheumatic diseases

#### Olena A. Oshlianska^1^, Illya A. Chaikovsky^2^, G. Mjasnikov^3^, A. Kazmirchyk^3^

##### ^1^State Institute of Pediatrics Obstetrics and Gynecology NAMS, Kyiv, Ukraine; ^2^V.M. Glushkov Institute of Cybernetics of NAS of Ukraine, Kyiv, Ukraine; ^3^National Military Medical Center of Ukraine, Kyiv, Ukraine

###### **Presenting author:** Olena A. Oshlianska

**Introduction:** According to a clinical registry frequency of cardiovascular system diseases ranged from 51% to 81% among 327 children with systemic diseases of connective tissue (SDCT). Injury of the heart is potentially life-threatening. Their initial signs cannot be detected by traditional diagnostic methods.

**Objectives:** improving early diagnosis of cardiac lesions in children with rheumatic diseases

**Methods:** There are 25 healthy and 31 sick children of 10-17 years with SDCT (SLE, JDM, MCTD), in which are absent active inflammation, endocrine pathology, and arterial hypertnsion, clinical and laboratory signs of carditis or cardiomyopathy were examined. Patients underwent magnetocardiographic examination, i.e. registration of heart magnetic field by SQUID-magnetometer at 36 points above the chest with 4 sm step, during 90 sec at single point. The inverse solution, i.e. reconstruction of current density vectors maps based on MCG-curves was done. The leading quantitative indicator of current density vectors maps is abnormal index (AI) the ratio of the total length of vectors directed in the “proper” direction to that have a different direction (%). To evaluate the processes of ventricular de- and repolarization this index was calculated during QRS complex and ST-T interval (AIQRS_total_ and AIST-T_total_. Besides, some other parameters were calculated, namely the ratio of the actual duration of each QRS phase to its “proper” duration (Duration Ratio - DR) for each of four depolarization phases (DR1-4) separately and for the whole process of depolarization (DR_total_), duration of complex QRS – DurQRS, the correlation coefficient C_cor_ between all maps during each interval, duration of ST-T curve area from its beginning to the inflection point in percentage to the overall length of the ST-T interval (A_Dur_).

**Results:** Statistically significant differences of average values of MCG indicators characterizing QRS complex were not found in the examined groups. However, individual analysis of normalized indicator of anomalous ventricular depolarization showed that it significantly deviates from the normal range in patients with active inflammatory process, mostly in front-apical area of myocardium (AI QRS2 70,15 ± 2,94% in patients, 56,4 ± 4,12% in healthy). This was accompanied by a reduction in the total duration of the QRS complex and the portion of normal complexes in the corresponding time intervals DRtotal 1,02 ± 0,04 s and in healthy 1,2 ± 0,09 s (by Dr1) respectively. DurQRS in patients with rheumatic diseases equal 0,089 ± 0,003 s (0,1 ± 0,04 s in healthy), AutoCorrQRS 80,43 ± 0,73% (84,14 ± 1,5% in healthy).

Correlation coefficient decreased in patients with rheumatic diseases insignificantly (CorrQRS 34,85 ± 1,01% in patients, 37,29 ± 7,01% in healthy). The analysis of the ST interval indicators showed reliable abnormalities in early repolarization of the ventricles (increasing Adur 19,85 ± 1,61% (11 ± 6,1% in healthy), reducing CorrST 63,85 ± 7,61% (82 ± 4,1% in healthy), downward trend in AI ST-Ttotal in patients 53,85 ± 3,35% (91 ± 0,4 in healthy children)). Comparing the results with standard ECG data it was revealed that the changes of repolarization processes are visualized up to 35% more frequently by MCG. Instant maps of current density vectors that obtained by MCG are more convenient for on-line visual assessment.

**Conclusion:** Non-invasiveness and sensitivity of MCG allow recommend it as a screening method for early detection of the heart injuries. The ability to visualize of electrophysiological active substrate makes the MCG as effective way to detect local changes into myocardium. Absolute safety of method makes possible its use for dynamical investigation.

**Disclosure of Interest**

None Declared

### P56 Early detection and follow-up of morphological alterations of temporomandibular joint in juvenile idiopathic arthritis: high-resolution ultrasound

#### Umberto Garagiola, Irene Borzani, Paolo Cressoni, Fabrizia Corona, Eszter Dzsida, Giampietro Farronato

##### Department of Biomedical, Surgical and Dental Sciences, University of Milan, Milano, Italy

**Introduction:** Ultrasonography could be an helpful screening method for the detection of TMJ involvement in children with JIA. It can be used and well tolerated even in very young children (2 years) without sedation or radiation risk.

**Objectives:** The aim of this work was to evaluate the usefulness of high-resolution ultrasound (US) in the early detection of temporomandibular joint (TMJ) alterations and to show normal and pathological imaging appearance of the TMJ in patients with Juvenile Idiopathic Arthritis (JIA). Although TMJ arthritis in JIA is frequently asymptomatic, the TMJ is particularly susceptible to damage. The TMJ is commonly involved in JIA and can lead to malocclusion, condylar alterations, facial deformity and restricted mouth opening.

**Methods:** High-resolution US examinations were performed in 130 patients with JIA. 34 patients (34%) had normal US appearance on both side. The sonograms were done using General Electric LOGIQ E9 equipment using real-time linear-array transducers with variable frequencies (9-18 MHz) by an experienced radiologist. Longitudinal and transverse view images were obtained. Dynamic video clips demonstrated condylar translation from “closed mouth” position to “open mouth” position. The movements can be recorded and compared between the left and the right side. Color Doppler US images furnish diagnostic information regarding hyperemia and synovial vascularity, thus allowed to study vascular signals in the soft tissues around the TMJ. Each joint was analyzed with regard to condylar irregularity, capsular thickness (effusion/synovial thickness), condylar disk dislocation, vascular increasing on Color-Doppler examination and symmetry in condylar translation between open- and closed-mouth position.

**Results:** In our clinical cases capsular thickness, enlargement of the intra-articular space, joint effusion, osteophytes, irregular condyle shape, disc displacement, erosions, increased color Doppler signals were detected. Limitations were especially related to the scarce accessibility of the medial part of the TMJ structures, and the need for trained operators. An important advantage especially in the case of children is that US examination does not require conscious sedation or general anesthesia.

**Conclusion:** The prevalence of TMJ involvement in patients with JIA varies widely (17–87%) and it is often asymptomatic even during the acute phase therefore consequently overlooked. Since inflammation in the TMJ can lead to joint deformity, early diagnosis and treatment of TMJ arthritis are particularly important. High-resolution US could be suggested as a screening tool to detect morphological alterations at an early stage of the JIA. Utilization of high-resolution US for TMJ disorder diagnosis in patients with JIA should increase due to relatively low costs and non-invasivity of the method, moreover it is a nonionizing tool. High-resolution US should be used for detecting inflammatory lesions before permanent TMJ destruction occurs, and monitoring disease progression and treatment response for a more effective therapy.

**Disclosure of Interest**

None Declared

### P57 Correlation between maxillary sinusitis and juvenile idiopathic arthritis: cone beam CT study

#### Umberto Garagiola^1^, Paolo Cressoni^1^, Fabrizia Corona^1^, Antonella Petaccia^1^, Eszter Dzsida^1^, Giampietro Farronato^1^

##### ^1^Department of Biomedical, Surgical and Dental Sciences, University of Milan, Milan, Italy

###### **Presenting author:** Umberto Garagiola

This abstract is not included here as it has already been published.

## Poster Session: Immunodeficiency and infection related arthritis

### P58 Attending to warning signs of primary immunodeficiency diseases in rheumatology practice: focus on juvenile idiopathic arthritis-like disease

#### Alenka Gagro, Agneza Marija Pasini, Goran Roic, Ozren Vrdoljak, Lucija Lujic, Matija Zutelija-Fattorini

##### Children’s Hospital Zagreb, Zagreb, Croatia

###### **Presenting author:** Alenka Gagro

**Introduction:** Primary immunodeficiency (PID) and autoimmune phenomena may occur concomitantly in the same individual. The most common forms of PID disease with articular involvement are those associated with humoral defects such as X-linked agammaglobulinaemia, common variable immune deficiency and selective IgA deficiency.

**Objectives:** The aim of our study was to systematically track a comprehensive array of PID in a cohort of 286 children with a presentation of chronic arthritis followed at the Immunology and Rheumatology outpatient clinic in Children’s Hospital Zagreb.

**Methods:** Patient’s charts were reviewed for the following immunological investigations performed as a part of the diagnostic algorithm for juvenile idiopathic arthritis: IgG, IgA, IgM, IgE, IgG subclasses, C3, C4, CH100, ANA, ANA, ENA-profile, antiphospholipid antibodies, rheumatoid factor (RF) and HLA-typing. Additional screening and specialized laboratory tests were performed in patients who presented with the recently revised warning signals for primary immunodeficiency.

**Results:** Six patients were identified with immunity defects compatible with classical PID. Two patients had selective IgA deficiency (sIgAD), while one patient with the following PIDs in each group was found: ataxia teleangiectasia (AT), autoimmune polyendocrinopathy-candidiasis-ectodermal dystrophy (APECED), autoimmune lymphoproliferative syndrome (ALPS) and familial Mediterranean fever (FMF). Majority of patients presented as RF-negative polyarthritis (see table).

**Conclusion:** Our findings further emphasize that PID and JIA may coexist and patients with autoimmune diseases should be carefully monitored for the presence of PID and vice versa. To the best of our knowledge, we also describe a first case of a child with typical ataxia telangiectasia who developed JIA.

**Disclosure of Interest**

None Declared

**Table 22 (abstract P58).** See text for description

PID (n)	Gender	ANA	RF	HLA association	Diagnosis of PID prior to arthritis (yrs)	Age at arthritis onset (yrs)
sIgAD (2)	F	+	-	-	No	2
	F	-	-	-	No	4
AT (1)	M	-	-	-	Yes/1	14
APECED (1)	M	-	-	-	No	6
ALPS (1)	F	-	-	-	Yes/13	15
FMF (1)	F	-	-	-	Yes/11	12

### P59 Familial Aicardi–Goutières Syndrome with SAMDH1 mutation –phenotype variation in 2 sisters from South Africa

#### Monika M. Esser^1^, Deepthi R. Abraham^2^, Craig Kinnear^2^, Glenda Durrheim^2^, Mike Urban^2^, Eileen Hoal^2^, Yanick Crow^3^

##### ^1^Pathology, Stellenbosch University, National Health Laboratory Services, Cape Town, South Africa; ^2^Stellenbosch University, Cape Town, South Africa; ^3^University of Manchester, Manchester, United Kingdom

###### **Presenting author:** Monika M. Esser

**Introduction:**

Aicardi-Goutières Syndrome (AGS) is a rare genetic disorder with broad phenotype spectrum. Access to molecular investigation confirmed AGS for different clinical manifestations in 2 sisters of non-consanguinous parents

**Objectives:** The value of a confirmed molecular diagnosis for a rare disease which would otherwise have remained undiagnosed is illustrated in our patients

**Methods:** We describe 2 sisters with unusual clinical features resembling childhood SLE and targeted molecular investigation. The index patient presented with hemiplegia and complex partial seizures in infancy, diagnosed as moyamoya syndrome on imaging. At 4 years of age she was referred to the paediatric rheumatology service with interface dermatitis, vasculitic lesions on her ears and fingertips and deteriorating gait. She tested negative for antinuclear antibody (ANA) and anti double-stranded DNA (dsDNA) but had positive antiphospholipid serology. She was anticoagulated with Warfarin, responded well to Chloroquine and Azathioprine for control of extensive vasculitic rash and Sodium Valproate for her seizure control. She was then diagnosed with multidrug resistant Tuberculosis and superficial staphylococcal abscesses of the spine which resolved on antituberculous and antibacterial treatment. The unusual combination of her clinical findings prompted molecular investigation. The older sister was found to have a longstanding history of vasculitic skin lesions and threatened gangrene of fingertips on presentation. She was severely stunted with delayed puberty, a feature not typically described with AGS. Skin biopsy showed perivascular-interface dermatitis. Antiphospholipid antibody screen and ANA were negative. She responded well to Nifedipine, Chloroquine and low dose Aspirin. She was subsequently diagnosed with glaucoma, which is associated with AGS and developed progressive contractures of interphalangeal joints with bone resorption on X- Ray.

**Results:** Blood samples from patients, their parents and unaffected brother were submitted to the Genetic Medicine Developmental Biomedicine Unit, Faculty of Medical and Human Sciences, University of Manchester. The 2 affected individuals carried a homozygous Ex15 c.1681_1682delAG; p.Ser561Phefs*61 in *SAMHD1* on chromosome 20. The parents and the brother were heterozygous for this variant. Variants were confirmed in the Division of Molecular Biology and Human Genetics, Faculty of Medicine and Health Sciences, Stellenbosch University. Since this mutation had not been described previously, 322 normal South African mixed racial population controls were genotyped by HRM and 63 of these were sequenced. No other chromosomes contained this mutation, the frequency of this ‘likely pathogenic’ mutation is <0.31% and therefore considered causative and consistent with a diagnosis of AGS.

**Conclusion:** Great intra-familial phenotypic variability of AGS, previously described in other publications, was documented in these two siblings with confirmed mutations of the SAMDH1 gene. The molecular investigation yielded a unifying diagnosis for an unusual combination of physical findings and different phenotype expression of AGS. Other benefits of a confirmed diagnosis were the genetic counseling which could be offered to the family and targeted investigation and treatment for complications such as glaucoma in the sister of the index patient. Although the parents are not related it was interesting that they were both heterozygous for the SAMDH1 mutation. Whether this AGS mutation results in increased susceptibility to tuberculosis, as a result of impaired interferon production, may need to be investigated further as a potential novel finding.

**Disclosure of Interest**

None Declared

### P60 Aseptic arthritis in children associated with primary immune deficiencies

#### Olena A. Oshlianska, Victoria B. Nikolayenko

##### Connective children diseases in children, State Institute of Pediatrics Obstetrics and Gynecology NAMS of Ukraine, Kyiv, Ukraine

###### **Presenting author:** Olena A. Oshlianska

**Introduction:** Defects of immune cells in children with primary immunodeficiency (PIDS) development may lead to formation of infectious, allergic, autoimmune and oncologic immunopathological syndromes. Were described both infectious and noninfectious arthritis.

**Objectives:** Identify the features of articular lesions in PIDS.

**Methods:** Were retrospectively analyzed clinical register data of patients with joint syndrome, hospitalized during 2000-2015.

**Results:** Among 901 children with joint syndrome in 21,97% its character at a pre-hospital stage was regarded incorrectly. Hyperdiagnostics was frequent in JIA (11,09%). In 13 cases (1,4%) the diagnosis of PIDS was established after an additional clinical examination. Most often (7 cases) joint lesions were observed at selective immunoglobulin A deficiency. Previous infectious was not revealed in these cases. Manifestation of joint syndrome 2-11 y., its features corresponded to JIA: 2 entezit-arthritis with inflammatory bowel diseases, 3 oligoarthritis, 1 sacroileitis, 1 sJIA. Immunological examinations: serum IgA ≤ 0,05 g/l, IgM, G, subclasses, B-cells within age values, was found depression of T-cytotoxic cells, in 4 cases - ANA, RF and HLA B27 weren’t taped. Were observed 3 cases of Bruton disease (3-6 y). In anamnesis occurred infectious syndrome (repeated pneumonia in 3 cases, pyelonephritis, chronic pyoderma), 2 patients had died sibs. Duration of joints lesions was 3 m -2 y, it was characterized as exudative oligoarthritis of knee joints. X-ray examination didn’t reveal osteal destructions. In 2 cases were reported movement restrictions and extremity elongations. Laboratory data: leukocytosis 12,5-17,2 × 10^9/^l, ESR 15-40 mm/h, IgG 0,2-2,5 g/l, Ig A 0-0,1 g/l, IgM 0,1-0,2 g/l, AB weren’t revealed, B-cels 0,02-0,85% (12-38 in mcl), CD19 + CD5 + 0-1,1%. In 2 cases the diagnosis was confirmed with genetic research. Dee-Georgie syndrome was established in child (4 y) with physical and psychomotorical development retardation. In his anamnesis were described cramps, repeated operations on cleft palate and interventricular septum defect. The infectious syndrome was not detected. Duration of joint syndrome was 2 y., were formed appreciable flexion contractures in small hand’s joints, all large upper and lower extremities joints, he couldn’t turn the head, the mouth opened partially. Were detected signs of exudative sinoviitis in radiocarpal, knee, ankle joints, deformations of toes. X-ray: systemic osteoporosis, multiple narrowings of joint clefts, single uzura. ESR 56 mm/h, CRP 48 ng/ml, ANA 1:80, RF, HLA B27 weren’t found, CD3+ 34,5%, CD3 + CD4 + 16,89%. RBTL FGA 28%. Cariotype 46XY, Fish 22+. In a 1 case with a HIDS during patient had aphthous stomatites, lymphadenopathy, gastroduodenal pathology, transient skin eruptions and ankle joints swelling without active sinoviitis or radiological changes. ESR– 13 mm/h, CRP 24 ng/ml, Ig, IL, phagocytosis, lymphocytic subpopulations were normal, IgD 620 IU/ml. HLA B27, ANA, AB DNA and desmoglein weren’t detected. In clinic were also examined 2 children with CINCA/NOMID syndrome. One child had dermal and fever syndromes, lymphadenopathies, bilateral congenital cataract, an optic nerves atrophy, conjunctivitis in the anamnesis, and delays of physical and psychomotor development, pathognomonic joints lesions since age of 1 year. The mutation of CIAS1 is taped in both cases. In all described PIDS cases there weren’t detected microorganisms in blood or synovial fluid, it was now rising of pro-calcitonin level.

**Conclusion:** In PIDS patients rheumatoid-like joint lesions can become perceptible. In patients with a joint syndrome the careful assessment of a phenotype and the anamnesis and they need screening immunologic assessment before establishment of the diagnosis of JIA.

**Disclosure of Interest**

None Declared

### P61 Retrospective analysis of laboratory data and treatments of 43 adult patients followed up with the diagnoses of acute rheumatic fever (ARF) and post-streptococcal reactive arthritis (PSRA)

#### Kubilay Şahin^1^, Yasar Karaaslan^2^

##### ^1^Rheumatology, Mersin State Hospital; Keçiören Education and Research Hospital, Ankara, Turkey; ^2^Rheumatology, Hitit University Medical Faculty, Ankara Numune Education and Research Hospital, Ankara, Turkey

###### **Presenting author:** Yasar Karaaslan

**Introduction:** Although rarer, ARF and PSRA are still seen in adults. There are no exact criteria to differentiate ARF from PSRA. Usually the arthritides that develop after a documented streptococcal infection, and not meeting modified Jones criteria are described as PSRA [1].

**Objectives:** Over the years, the opinions on the clinical findings of ARF and PSRA, and the clinical approach to those diseases changed. In this retrospective study, we aimed to analyze 43 adult patients that were followed up with the diagnoses of ARF or PSRA in a secondary and a tertiary hospital.

**Methods:** 29 patients that had been followed up with the diagnoses of ARF or PSRA in the Rheumatology Clinic of Ankara Numune Education and Research Hospital and 14 patients that had been followed up with the same diagnoses in Mersin State Hospital in the previous l year, and a total of 43 patients were included in the study. Patient files and their data on electronic data recording system of the hospital were examined to analyze the clinical and laboratory findings of the patients and their treatments.

**Results:** 6 (14%) patients that met the modified Jones criteria were regarded as ARF, and the remaining 37 (86%) patients were regarded as PSRA. Among ARF patients, there were 4 F and 2 M with a mean age of 28.3 ± 7 years. The patients with ARF could not be compared with PSRA patients since their number was small. However, it was noteworthy that 4 (67%) patients with ARF were recurrent patients that had their first attacks during childhood, and had cardiac valvular disease. The patients with PSRA consisted of 31 F and 6 M with a mean age of 30.4 ± 5.8 years. None of the patients had clinical cardiac involvement. None of 11 patients that had echocardiography had rheumatic valvular involvement. The most frequently involved joints were ankle (50%), knee (38%), wrist (19%), and MTP (8%) joints. Migratory arthritis was seen in only 2 (5.4%) patients. There was mono in 27%, oligo in 57%, and polyarthritis in 16% of the patients. Two (5.4%) patients had concomitant erythema nodosum. There was history of tonsillitis in 46% of the patients previous to the onset of arthritis. The mean period between tonsillitis and arthritis was 25 ± 9 days in those patients. The mean duration of arthritis at the time of admission was 21 ± 13 days. Fever was present only in 4 (10.8%) patients. None of the patients had erythema marginatum, subcutaneous nodules or chorea. The mean ESR, CRP, and ASO values of the patients on admission were 57.8 ± 28.6 mm/h, 6.34 ± 5.3 mg/dL, and 971.5 ± 505.3 IU/mL, respectively. The ESR, CRP and ASO values on follow up were 29.5 ± 21 mm/h, 1.6 ± 2.2 mg/dL, and 822.5 ± 468 IU/mL, respectively. Repository penicillin was administered to 84%, oral and then repository penicillin were administered to 11%, and macrolides were administered due to penicillin allergy to 5% of the patients. Anti-inflammatory treatment administered to the patients included 20 mg/day or more oral corticosteroids (CS) in one patient, less than 20 mg/day oral CS in 28 patients (75.7%) (prednisolone equivalent), repository CS in 2 (5.4%) patients, and NSAID alone in 6 patients (16.2%). The mean use of CS was 8.5 ± 3.8 weeks. Chronic arthritis was seen in only 3 (8.1%) patients in long term.

**Conclusion:** Currently, ARF and PSRA are not still rarely seen diseases. It was noteworthy that typical ARF findings including fever, cardiac involvement, migratory erythematous arthritis, and skin involvement were rarely seen in our adult patients with PSRA, however joint findings continued longer, the ASO titer was over 500 IU in 92% of the cases, and most of the patients required low-dose corticosteroids instead of aspirin or NSAIDs.

**References**

1- Curr Opin Rheumatol. 2002;14:562–5.

**Disclosure of Interest**

None Declared

## Poster Session: JIA (oligo, poly, psoriatic) I

### P62 Musculoskeletal symptoms at the onset of pediatric tumors and predictive features in the differential diagnosis with juvenile idiopatic arthritis: preliminary analysis of a multicenter, prospective, observational study

#### Adele Civino^1^, Giovanni Alighieri^1^, Sergio Davì^2^, Roberto Rondelli^3^, Silvana Martino^4^, Giovanni Filocamo^5^, Andrea Magnolato^6^, Francesca Ricci^7^, Romina Gallizzi^8^, Alma Olivieri^9^, Valeria Gerloni^10^, Bianca Lattanzi^11^, Francesca Soscia^12^, Alessandro De Fanti^13^, Silvia Magni Manzoni^14^, Stefania Citiso^1^, Lorenzo Quartulli^1^, Francesco La Torre^15^, Donato Rigante^16^, Maria Cristina Maggio^17^, Manuela Marsili^18^, Maria Antonietta Pelagatti^19^, Valentino Conter^20^, Franca Fagioli^21^, Loredana Lepore^6^, Andrea Pession^3^, Angelo Ravelli^2^

##### ^1^Pediatric Rheumatology, Tricase (LE), Italy; ^2^Pediatric Rheumatology, Genova, Italy; ^3^Pediatric Oncology, Bologna, Italy; ^4^Pediatric Rheumatology, Torino, Italy; ^5^Pediatric Rheumatology, Milano - De Marchi, Italy; ^6^Pediatric Rheumatology, Trieste, Italy; ^7^Pediatric Rheumatology, Brescia, Italy; ^8^Pediatric Rheumatology, Messina, Italy; ^9^Pediatric Rheumatology, Napoli, Italy; ^10^Pediatric Rheumatology, Milano - G. Pini, Italy; ^11^Pediatric Rheumatology, Ancona, Italy; ^12^Pediatric Rheumatology, Orvieto, Italy; ^13^Pediatric Rheumatology, Reggio Emilia, Italy; ^14^Pediatric Rheumatology, Roma - Bambino Gesù, Italy; ^15^Pediatric Rheumatology, Brindisi, Italy; ^16^Pediatric Rheumatology, Roma - Cattolica, Italy; ^17^Pediatric Rheumatology, Palermo, Italy; ^18^Pediatric Rheumatology, Chieti, Italy; ^19^Pediatric Rheumatology, Monza, Italy; ^20^Pediatric Oncology, Monza, Italy; ^21^Pediatric Oncology, Torino, Italy

###### **Presenting author:** Adele Civino

**Introduction:** The onset of some pediatric tumors may be characterized by musculoskeletal symptoms that can mimic rheumatic diseases like juvenile idiopatic arthritis (JIA). This could lead to inappropriate steroid treatment or immunosuppressive therapy with a delay in the diagnosis.

**Objectives:** To study prevalence and features of musculoskeletal symptoms at the onset of pediatric tumors diagnosed at the Italian Association of Pediatric Hematology and Oncology (AIEOP) Centres; to identify predictive factors of malignancy comparing the new cases of tumors with early musculoskeletal symptoms with JIA diagnosed in the same period at the Pediatric Rheumatology Centres of Italian Society of Paediatrics (SIP).

**Methods:** We analyze electronic case report form of pediatric patients (<16 years) with new diagnosis of cancer or JIA collected through a web based data system.

**Results:** 420 patients were enrolled between 01.05.2015 and 15.03.2016. 147 of them were affected by JIA and 272 by tumor: 50.4% acute leukemia (AL) and 49.6% solid tumors. Musculoskeletal symptoms were found in 81 (29.8%) patients with tumor; higher frequency of symptoms were found on patients with bone sarcoma (100%), neuroblastoma (41%) and AL (34,3%). Arthritis was observed in 29 (10.7%) patients with tumor including 18 AL and 11 solid tumors. Male/female ratio was 1.65. The mean number of involved joints was 1.66 (range 1-6). The most frequently involved joints were hips, knees and ankles. Joint involvement (arthritis and/or arthralgia) was observed in 60 (22%) patients with tumor including 38 AL and 22 solid tumors. We compared clinical features of patients with tumors and joint involvement with those of patients with JIA and we found that arthralgia, bone pain of the legs, pain of the spine, unproportioned pain and refusal to deambulation were significantly associated with neoplastic disease. Finding of arthritis, arthritis and arthralgia, morning stiffness, limp and the presence of only musculoskeletal symptoms were significantly associated with JIA.

**Conclusion:** With a preliminary analysis of data we found that musculoskeletal symptoms at the onset of tumors were present in about 30% of patients. These symptoms have features strictly comparable with JIA so they may be useful in the differential diagnosis with JIA.

**Disclosure of Interest**

None Declared

**Table 23 (abstract P62).** Comparison of clinical parameters between tumors with joint involvement and JIA

	Tumors with joint involvement (60 pts)		JIA (147 pts)		*p-*value
	n°	%	n°	%	
Arthritis	23	38,3	107	72,8	0.000
Bone pain	24	40,0	4	2,7	0.000
Nighttime pain	7	11,7	6	4,1	0.057
Pain of the spine	15	25,0	12	8,2	0.002
Unproportioned pain	8	13,3	2	1,4	0.001
Morning stiffness	1	1,7	80	54,4	0.000
Limp	25	41,7	89	60,5	0.014
Only musculoskeletal symptoms	16	26,7	116	78,9	0.000

### P63 Differences in disease phenotype, management and outcomes of children with juvenile idiopathic arthritis throughout the world – analysis of 8,325 patients enrolled in the EPOCA study

#### Alessandro Consolaro^1^, Nicolino Ruperto^2^, Marco Garrone^2^, Mariangela Rinaldi^2^, Jaime De Inocencio^2^, Erkan Demirkaya^2^, Stella Garay^2^, Dirk Foell^2^, Daniel J. Lovell^2^, Calin Lazar^2^, Susan Nielsen^2^, Berit Flato^2^, Alberto Martini^2^, Angelo Ravelli^1^, and EPOCA Study Group

##### ^1^Pediatria II, Istituto Giannina Gaslini, Genova, Italy; ^2^PRINTO, Istituto Giannina Gaslini, Genova, Italy

###### **Presenting author:** Alessandro Consolaro

**Introduction:** Several epidemiologic surveys have documented a remarkable, yet unexplained, disparity in the prevalence of juvenile idiopathic arthritis (JIA) subtypes among different geographic areas or ethnic groups. Moreover, the therapeutic approach to JIA is not standardized and the availability of the novel and costly biologic medications is not uniform throughout the world. This disparity may have significant impact on disease outcome.

**Objectives:** The multinational study of the EPidemiology, treatment and Outcome of Childhood Arthritis (EPOCA study) is aimed to obtain information on the variability of JIA phenotypes in different geographic areas, the therapeutic approaches of pediatric rheumatologists practicing in diverse countries, and the disease status and outcome of children with JIA currently followed worldwide.

**Methods:** Participation in the study was proposed to all pediatric rheumatology centers that are part of the Pediatric Rheumatology International Trials Organization (PRINTO), and to several centers in the US and Canada. Each center was asked to enroll 100 consecutive JIA patients or all consecutive patients seen within 6 months. Each patient received a retrospective and cross-sectional assessment. Parent- and child-reported outcomes were recorded through the administration of the Juvenile Arthritis Multidimensional Assessment Report (JAMAR). Participating countries were grouped into 6 geographic areas.

**Results:** Currently, 8,325 patients from 44 countries have been entered in the web database. Comparison of main epidemiology, treatment, and outcome features across the different geographic areas is presented in the table.

**Conclusion:** These results provide further evidence of the wide difference of JIA characteristics across geographic areas in terms of age at disease onset, subtype prevalence, and frequency of anterior uveitis. Overall, patients living in non-Western countries had higher levels of disease activity and cumulative damage than patients followed in North America and Western Europe. This disparity in disease outcomes may be partially due to differences in the availability or affordability of biologics, as usage of these medications was more common in Western pediatric rheumatology centers.

**Disclosure of Interest**

A. Consolaro: None Declared, N. Ruperto Grant / Research Support from: The G. Gaslini Hospital, which is the public Hospital where I work as full time public employee, has received contributions from the following industries for the coordination activity of the PRINTO network: BMS, GlaxoSmithKline (GSK), Hoffman-La Roche, Novartis, Pfizer, Sanofi Aventis, Schwarz Biosciences, Abbott, Francesco Angelini S.P.A., Sobi, Merck Serono. This money has been reinvested for the research activities of the hospital in fully independent manners besides any commitment with third parties., Speaker Bureau of: I received speaker’s bureaus and consulting fees from the following pharmaceutical companies: AbbVie, Amgen, Biogenidec, Alter, AstraZeneca, Baxalta Biosimilars, Biogenidec, Boehringer, BMS, Celgene, CrescendoBio, EMD Serono, Hoffman-La Roche, Italfarmaco, Janssen, MedImmune, Medac, Novartis, Novo Nordisk, Pfizer, Sanofi Aventis, Servier, Takeda, UCB Biosciences GmbH., M. Garrone: None Declared, M. Rinaldi: None Declared, J. De Inocencio: None Declared, E. Demirkaya: None Declared, S. Garay: None Declared, D. Foell: None Declared, D. Lovell: None Declared, C. Lazar: None Declared, S. Nielsen: None Declared, B. Flato: None Declared, A. Martini Grant / Research Support from: The G. Gaslini Hospital, which is the public Hospital where I work as full time public employee, has received contributions from the following industries for the coordination activity of the PRINTO network: BMS, GlaxoSmithKline (GSK), Hoffman-La Roche, Novartis, Pfizer, Sanofi Aventis, Schwarz Biosciences, Abbott, Francesco Angelini S.P.A., Sobi, Merck Serono. This money has been reinvested for the research activities of the hospital in fully independent manners besides any commitment with third parties., Speaker Bureau of: I received speaker’s bureaus and consulting fees from the following pharmaceutical companies: Abbvie, Boehringer, Celgene, CrescendoBio, Janssen, Meddimune, Novartis, NovoNordisk, Pfizer, Sanofi Aventis, Vertex, Servier., A. Ravelli: None Declared

**Table 24 (abstract P63).** See text for description

	Africa	Asia	Eastern Europe	Latin America	North America	Western Europe
	N = 261	N = 874	N = 2587	N = 814	N = 422	N = 3367
Median (IQR) age at onset, years	7 (3.6–10.5)	5.7 (2.9–9.2)	6.4 (2.9–10.5)	6.6 (3.6–10.3)	8.1 (3.7–11.1)	4.1 (2–8.7)
Systemic arthritis	17.2	26.5	8.1	17.7	4.7	6.9
Oligoarthritis	23	31.9	44.1	31.7	33.9	49.2
Uveitis	4.6	5.5	10	6.6	10.4	16.9
Use of biologics	22.6	23.6	28.9	34.2	47.6	39
Median (IQR) cJADAS10	7 (2–11)	2.5 (0–7.8)	4.5 (1–9.5)	2.5 (0–9.5)	2.3 (0–7)	2 (0–6)
Median (IQR) JAFS score	4 (0–8)	1 (0–5)	1 (0–4)	1 (0–5)	1 (0–4)	0 (0–3)
JADI-Articular > 0	27.6	19.2	23.7	31.9	15.2	12.2
JADI-Extraarticular > 0	24.5	16.8	14.8	14.5	5.9	9.1

Data are percentages unless otherwise indicated. cJADAS: clinical (3-item) JADAS; JAFS: Juvenile Arthritis Functionality Scale; JADI: Juvenile Arthritis Damage Index

### P64 Changes in switched memory B cells are associated to the therapeutic response to TNF inhibition in JIA

#### Emiliano Marasco^1^, Angela Aquilani^1^, Simona Cascioli^2^, Ivan Caiello^1^, Gian Marco Moneta^1^, Denise Pires-Marafón^1^, Silvia Magni-Manzoni^1^, Rita Carsetti^2^, Fabrizio De Benedetti^1^

##### ^1^Division of Rheumatology, Ospedale Pediatrico Bambin Gesù, Roma, Italy; ^2^Immunology Diagnostic Unit, Ospedale Pediatrico Bambin Gesù, Roma, Italy

###### **Presenting author:** Emiliano Marasco

**Introduction:** Both the innate and adaptive branches of the immune system have been implicated in the development of the various forms of JIA. Evidence suggests that B cells may be involved in oligoarticular and polyarticular JIA.

**Objectives:** To investigate possible abnormalities in B lymphocyte subsets in patients with oligo- and poly- JIA and their correlation with clinical features and response to treatment.

**Methods:** 107 patients diagnosed with oligo- or poly-JIA were studies and compared with 304 age-matched healthy children (HC). Peripheral Blood Mononuclear Cells (PBMCs) were isolated, stained with different sets of fluorochrome-conjugated antibodies and run on a BD LSRFortessa-X-20.

**Results:** Switched memory B cells were found to be significantly increased in JIA patients when compared to age-matched HC [% of CD19: 13.16% (±5.5) and 7.33% (±4.2) respectively, *p* < 0.0001]. The other subpopulations of B cells were not increased. Switched memory B cells were higher in JIA patients than HC at any given age. Switched memory B cells were approximately 70% of the B cells found in JIA synovial fluid. We also found that synovial B cells of ANA positive patients produced antinuclear antibodies. There was no association of the number of switched memory B cells with disease activity, number of joints with active arthritis or presence of iridocyclitis. In patients treated with methotrexate (MTX) alone there was a progressive increase in switched memory B cells with a positive correlation with the duration of treatment (Spearman r = 0.36; p = 0.0368). On the other hand, in patients treated with MTX and TNF inhibitors (TNFi) there was a negative correlation with treatment duration, however, it was not significant (Spearman r = -0.098; p = 0.27). Notably, during follow-up, in patients treated with MTX alone, the number of switched memory B cells increased significantly regardless of the presence of active (+42.4% ± 35) or inactive disease (+30% ± 34). In patients treated with MTX + TNFi who showed persistent active disease or disease flare the number of switched memory B cells increased significantly (+41.58% ± 32). In sharp contrast, in patients on MTX + TNFi with inactive disease, the increase in switched memory B cells was significantly lower (+9.8% ± 25) (Table). To take into account time as a variable, we calculated the slope of the increase in switched memory B cells over 12 months, as a measure of the rate at which switched memory B cells increased over time. The increase of the rate of switched memory B cells in patients with inactive disease on TNFi (0.09 ± 0.34) was significantly lower when compared to patients treated with TNFi who showed persistent active disease or disease flare (0.39 ± 0.32) and was comparable to that of HC (0.13 ± 0.06) (Table).

**Conclusion:** Switched memory B cells are increased in patients with oligo- and poly-JIA. Treatment with TNFi slows the increase of switched memory B cells over time in comparison to patients treated with MTX alone. Moreover, patients that responded to TNFi maintaining a persistent inactive disease during follow up showed a reduction in the rate of increase of switched memory B cells over time. Switched memory B cells may be involved in the development of oligo- and poly-JIA and their change over time is associated to the clinical response to TNFi.

**Disclosure of Interest**

None Declared

**Table 25 (abstract P64).** See text for description

	MTX with active disease (n = 4)	MTX with clinical remission (n = 10)	*t*-Test (p-value)	MTX + TNFi with active disease (n = 19)	MTX + TNFi with clinical remission (n = 14)	*t*-Test (p-value)
% change from baseline of switched memory B cells (±SD)	42%(±35)	29.9%(±34)	0.55	41%(±32)	9.8%(±25)	0.0047
Slope (indicating the mean % change of switched memory B cells in 12 months) (±SD)	0.43(±0.35)	0.42(±0.38)	0.95	0.39(±0.32)	0.09(±0.34)	0.015

### P65 Towards stratified medicine in juvenile idiopathic arthritis

#### Emily Robinson^1^, Salvatore Albani^2^, Michael W. Beresford^3^, Wilco de Jager^4^, Sytze de Roock^4^, Trang Duong^5^, Justine Ellis^6^, Kimme Hyrich^7^, Laetitia Jervis^1^, Daniel Lovell^8^, Lucy Marshall^1^, Elizabeth D. Mellins^9^, Kirsten Minden^10^, Jane Munro^6^, Peter A. Nigrovic^11^, Jason Palman^1^, Johannes Roth^12^, Nicolino Ruperto^13^, Sunil Sampath^14^, Laura E. Schanberg^15^, Susan D. Thompson^8^, Wendy Thomson^14^, Richard Vesely^16^, Chris Wallace^17^, Chris Williams^18^, Qiong Wu^1^, Nico Wulffraat^4^, Rae S. M. Yeung^5^, Berent Prakken^4^, Lucy R. Wedderburn^1^

##### ^1^Institute of Child Health, University College London, London, United Kingdom; ^2^SingHealth Translational Immunology and Inflammation Centre, Singhealth and Duke-NUS Graduate Medical School, Singapore, Singapore; ^3^Institute of Translational Medicine, University of Liverpool, Liverpool, United Kingdom; ^4^Department of Paediatric Immunology, University Medical Centre Utrecht, Utrecht, Netherlands; ^5^Division of Rheumatology, Hospital for Sick Children, University of Toronto, Toronto, Canada; ^6^Murdoch Children’s Research Institute, Royal Children’s Hospital, Melbourne, Australia; ^7^Arthritis Research UK Centre for Epidemiology, Centre for Musculoskeletal Research, University of Manchester, Manchester, United Kingdom; ^8^Division of Rheumatology, Cincinnati Children’s Hospital Medical Center, Cincinnati, OH, USA; ^9^Department of Pediatrics, Standford University School of Medicine, Stanford, CA, USA; ^10^Paediatric Rheumatology, German Rheumatism Research Centre, Berlin, Germany; ^11^Division of Immunology, Boston Children’s Hospital, Boston, MA, USA; ^12^Institute of Immunology, University of Munster, Munster, Germany; ^13^Pediatria II Reumatologia, Instituto Giannina Gaslini, Genova, Italy; ^14^Arthritis Research UK Centre for Genetics and Genomics, Centre for Musculoskeletal Research, University of Manchester, Manchester, United Kingdom; ^15^Department of Pediatrics, Duke University Medical Center, Durham, NC, USA; ^16^Rheumatology, Immunology, Respiratory and Gastroenterology office, European Medicines Agency, London, UK; ^17^Department of Medicine, University of Cambridge and MRC Biostatistics unit, Cambridge, UK; ^18^UCL Business PLC, University College London, London, United Kingdom

###### **Presenting author:** Emily Robinson

**Introduction:** Despite the efficacy of current biologic agents for the treatment of juvenile idiopathic arthritis (JIA) in many children, for others, treatment is ineffective and they continue to suffer active disease and disability. There are an increasing number of biological agents available but we have no validated biomarkers of treatment response with which to predict response and guide therapy choices. In order to develop a rational, evidenced-based approach to support choices of therapy for these children (stratified medicine), multi-center, international collaboration is vital.

**Objectives:** An international working meeting focused on stratified medicine in JIA, was held. The objectives of which were to reach consensus on how to overcome barriers and hurdles to robust, collaborative, clinical/translational research, and to foster effective and successful collaboration.

**Methods:** An international group of experts (paediatric rheumatologists, epidemiologists, geneticists, immunologists and translational scientists), from 8 countries and 17 institutions, representing major national and international networks, recently came together for two days to drive progress in stratified medicine in JIA. Prior to the meeting all investigators contributed to a priority-setting exercise to define the most important and feasible research questions, hurdles and possible solutions. These responses were grouped into themes, for both questions and hurdles, and then participants were asked to rank each theme in order of their perceived importance. Currently available tools, such as appropriate biobank protocols from previously established networks within pediatric rheumatology were collated.

**Results:** The key elements of a new framework to guide processes for working together were agreed, a new International collaborative study was proposed, and a declaration on principles of Research Collaboration for Stratified Medicine in Childhood Rheumatic Disorders – termed the London Declaration, was agreed and signed by all.

**Conclusion:** The London Declaration on Stratified Medicine Research for JIA and the principles it represents, should accelerate progress towards a stratified treatment approach not only for children with JIA but for all childhood rheumatic diseases.

**Disclosure of Interest**

E. Robinson Grant / Research Support from: The Chart consortium is supported by Pfizer, Roche and Janssen. The meeting was partially supported by AbbVie ltd., S. Albani: None Declared, M. Beresford Grant / Research Support from: The Chart consortium is supported by Pfizer, Roche and Janssen. The meeting was partially supported by AbbVie ltd., W. de Jager: None Declared, S. de Roock: None Declared, T. Duong: None Declared, J. Ellis: None Declared, K. Hyrich Grant / Research Support from: The Chart consortium is supported by Pfizer, Roche and Janssen. The meeting was partially supported by AbbVie ltd., Consultant for: Honoraria from AbbVie and Pfizer, L. Jervis: None Declared, D. Lovell: None Declared, L. Marshall: None Declared, E. Mellins : None Declared, K. Minden : None Declared, J. Munro: None Declared, P. Nigrovic: None Declared, J. Palman : None Declared, J. Roth : None Declared, N. Ruperto: None Declared, S. Sampath : None Declared, L. Schanberg Grant / Research Support from: The Chart consortium is supported by Pfizer, Roche and Janssen. The meeting was partially supported by AbbVie ltd., S. Thompson : None Declared, W. Thomson Grant / Research Support from: The Chart consortium is supported by Pfizer, Roche and Janssen. The meeting was partially supported by AbbVie ltd., R. Vesely Employee of: The views and opinions expressed in this article are those of the individual co-author and should not be attributed to the European Medicines Agency., C. Wallace : None Declared, C. Williams Grant / Research Support from: The Chart consortium is supported by Pfizer, Roche and Janssen. The meeting was partially supported by AbbVie ltd., Q. Wu : None Declared, N. Wulffraat: None Declared, R. Yeung : None Declared, B. Prakken: None Declared, L. Wedderburn Grant / Research Support from: The Chart consortium is supported by Pfizer, Roche and Janssen. The meeting was partially supported by AbbVie ltd.

### P66 Safety of adalimumab in pediatric patients with polyarticular juvenile idiopathic arthritis, enthesitis-related arthritis, psoriasis, and Crohn’s disease

#### G Horneff^1^, MB Seyger^2^, D Arikan^3^, J Kalabic^4^, JK Anderson^3^, A Lazar^4^, DA Williams^3^, C Wang^3^, R Tarzynski-Potempa^3^, JS Hymans^5^

##### ^1^Asklepios Clinic, Sankt Augustin, Germany; ^2^Radboud University Nijmegen Medical Center, Nijmegen, Netherlands; ^3^AbbVie Inc., North Chicago, IL, USA; ^4^AbbVie Deutschland GmbH & Co. KG, Ludwigshafen, Germany; ^5^Connecticut Children’s Medical Center, Hartford, CT, USA

###### **Presenting author:** G Horneff

This abstract is not included here as it has already been published.

### P67 Flares after withdrawal of biotherapies in JIA: clinical and laboratory correlates of remission duration

#### Gabriele Simonini^1^, Erika Scoccimarro^1^, Irene Pontikaki^2^, Giovanna Ferrara^3^, Teresa Giani^1^, Alessandro Ventura^3^, Pier Luigi Meroni^4^, Rolando Cimaz^1^

##### ^1^Rheumatology Unit-Dpt of Paediatrics University of Florence-Anna Meyer Children Hospital, Firenze, Italy; ^2^Gaetano Pini Institute, Milano, Italy; ^3^Institute for Maternal and Child Health - IRCCS “Burlo Garofolo”, University of Trieste, Trieste, Italy; ^4^Gaetano Pini Institute, University of Milano, Milano, Italy

###### **Presenting author:** Gabriele Simonini

**Introduction:** Information regarding the history of patients with JIA after systemic treatment withdrawal would be helpful in driving the choice of duration therapy. While some informations in this regard exist for second-line drugs (eg methotrexate), studies on biologics are lacking.

**Objectives:** Aim of our study was to assess the time on remission after discontinuing biologic therapy in a retrospective, comparative, multicenter, cohort study of patients with JIA.

**Methods:** Among 349 JIA patients followed in three tertiary care centres and treated with biologics (started before age 18 y), 135 (103 F, 32 M; median age 15 years) achieved remission and could discontinue such treatment. Only first cycle of biologics was considered for this study: 87 received Etanercept, 27 Adalimumab, 12 Infliximab, 7 Anakinra, 1 Rituximab, and 1 Abatacept. Primary outcome was to assess, once remission was achieved, the time on remission up to the first flare after discontinuing treatment. Time to achieve remission once biologic drug was started, corticosteroid usage, and time on remission on therapy before discontinuing all treatments were also considered, along with demographic, clinical, and laboratory data. Mann–Whitney *U*-test, Wilcoxon signed-rank test for paired samples, chi-square, and Fisher’s exact test, when appropriate, were used to compare data. Pearson and Spearman correlation tests were used to determine correlation coefficients for different variables. In order to identify predictors of outcome Cox regression model and Kaplan–Meier curves were constructed, each one at mean of entered covariates.

**Results:** The vast majority of enrolled patients flared after stopping treatment with biologics (102/135, 75.6%) with a median follow-up time on remission of 6 months (range 1–109). Considering the time point one year after treatment discontinuation 43/135 (31.9%) patients were still in remission. For this group, remission lasted for a median period of 53 months (range 12-109): 30/48 with polyarticular onset, 7/27 oligoarticular extended, 6/35 oligoarticular persistent, 2/6 enthesitis-related arthritis, 2/7 psoriatic arthritis, and 8/12 systemic onset JIA. A higher probability of maintaining remission after discontinuing treatment was present in systemic onset disease compared to the rest of JIA patients (Mantel-Cox *χ*2 5.48, p < 0.02). ANA positivity was associated with a higher probability of flare (Mantel-Cox *χ*2 4.17, p < 0.04). Patients who received biologics > 2 years after achieving remission had a higher probability of maintaining such remission off therapy (median 21.74 ± 3.4 months vs 13.4 ± 2.3; Mantel-Cox *χ*2 6.86, p < 0.009). No other clinical variable, including total length of treatment and type of treatment, resulted significantly associated with a long-lasting remission.

**Conclusion:** Even if in a retrospective study, we showed that patients with ANA positivity and those who stop treatment before two years of remission have a higher chance of relapsing after biologic withdrawal, and that patients with systemic onset disease are less likely to flare

**Disclosure of Interest**

None Declared

### P68 An active proNGF-p75NTR axis plays a pro-inflammatory role in chronic arthritis

#### Gaetana Minnone^1^, Marzia Soligo^2^, Ivan Caiello^1^, Giusi Prencipe^1^, Denise Pires Marafon^1^, Silvia Magni-Manzoni^1^, Luigi Manni^2^, Fabrizio De Benedetti^1^, Luisa Bracci Laudiero^2^

##### ^1^Division of Rheumatology, Bambino Gesu’ Children’s Hospital, Rome, Italy; ^2^Institute of Translational Pharmacology, Consiglio Nazionale delle Ricerche (CNR), Rome, Italy

###### **Presenting author:** Gaetana Minnone

**Introduction:** Inflammation has been associated with a marked increase in the production of nerve growth factor (NGF) in tissues of patients with a variety of chronic inflammatory diseases, including rheumatoid arthritis and juvenile idiopathic arthritis (JIA). However, the effects of NGF, its immature form proNGF, and their receptors, TrkA and p75NTR, in regulating cells and mediators during inflammatory responses and their contribution to the pathogenesis of arthritis are not yet defined.

**Objectives:** To evaluate the role of proNGF, mature NGF and their receptors in the modulation of inflammatory response in chronic arthritis patients.

**Methods:** Seventy-five JIA patients, according to the International League of Associations for Rheumatology classification criteria, were divided in persistent oligoarticular, extended oligoarticular or polyarticular subtypes. The expression of the NGF receptors, p75NTR and TrkA, in peripheral blood (PBMC) and synovial fluid (SFMC) mononuclear cells from JIA patients and healthy children was analyzed using real time-PCR and Western Blot. In ex vivo experiments JIA SFMC were treated with mature NGF or proNGF and the effects on intracellular signaling pathways and cytokine production were assessed by Western Blot, real time-PCR and ELISA

**Results:** p75NTR is the main NGF receptor expressed in JIA PBMC and SFMC while TrkA is the prevalent NGF receptor in healthy donor mononuclear cells. The increased expression of p75NTR and the concomitant decrease in TrkA, at both the mRNA and protein levels, resulted in a highly abnormal ratio between p75NTR and TrkA in JIA cells. The highest expression of, not only p75NTR, but also sortilin, the p75NTR co-receptor, was found in JIA SFMC. When patients were divided according to JIA subtypes the highest p75NTR expression levels were found in SFMC of patients with polyarticular JIA. The lowest p75NTR expression levels characterized SFMC from persistent oligoarticular patients while patients with extended oligoarticular JIA showed intermediate p75NTR levels. A significant correlation was found in JIA patients between the p75NTR mRNA expression levels in SFMC and the number of active joints at sampling and the levels of C-reactive protein. In the synovial fluid of JIA patients the concentration of proNGF, the specific ligand of p75NTR, far exceeded that of mNGF. In ex vivo experiments, LPS-stimulated SFMC of JIA patients were treated with proNGF. We found that proNGF specifically enhanced phosphorylation of p38, JNK and IkB, all involved in inflammatory cytokine production in immune cells. The addition of NGF did not induce the activation of these pathways and proNGF, but not mNGF, induced in SFMC a higher production of pro-inflammatory cytokine. This production was blocked using p75NTR inhibitors.

**Conclusion:** Our results show that high levels of p75NTR mRNA expression in the SFMC of JIA patients are associated with the most severe subtype and with disease activity. We also found that proNGF is the predominant form of NGF in the synovial fluid of JIA patients. Blocking of p75NTR in ex vivo experiments with JIA SFMC showed that proNGF acts through p75NTR. Altogether these results suggest that an active proNGF-p75NTR axis is involved in sustaining inflammatory production and may provide the rationale for specifically targeting p75NTR in chronic arthritides.

**Disclosure of Interest**

None Declared

### P69 Humoral and cellular responses to varicella vaccination of patients with pediatric rheumatic diseases – preliminary results of a vaccination study

#### Noortje Groot^1,2^, I Grein^2,3^, N.M. Wulffraat^2^, R Schepp^4^, G Berbers^4^, C. C. Barbosa Sandoval de Souza^5^, V. Paes Leme Ferriani^5^, G Pileggi^5^, S. de Roock^6^

##### ^1^Department of Pediatric Rheumatology, Sophia Children’s Hospital, Erasmus University Medical Centre, Rotterdam, Netherlands; ^2^Department of Pediatric Immunology and Rheumatology, Wilhelmina Children’s Hospital, University Medical Centre, Utrecht, Netherlands; ^3^Department of Pediatric Rheumatology, Pequeno Príncipe Hospital, Curitiba, Brazil; ^4^National Institute of Public Health and the Environment (RIVM), Bilthoven, Netherlands; ^5^Department of Pediatrics, University of Medicine of Ribeirão Preto, São Paulo, Brazil; ^6^Wilhelmina Children’s Hospital, University Medical Centre, Utrecht, Netherlands

###### **Presenting author:** I Grein

**Introduction:** Vaccination can be beneficial in children with pediatric rheumatic diseases (PRD), who are prone to infections. However, due to the disease itself as well as due to the use of immunosuppressive drugs, the vaccine-induced immune response of these patients can be impaired. Additionally, vaccines should be safe: they should not induce a disease flare, and, when the vaccine contains a live-attenuated pathogen, it should not induce an infection. One of the possibly beneficial vaccines for PRD patients is the varicella zoster virus (VZV) vaccination which can be used to prevent chickenpox and shingles. An adequate cellular immune response is especially important for effective protection against VZV.

**Objectives:** To assess the humoral and cellular responses to the live-attenuated VZV vaccination in children with juvenile idiopathic arthritis (JIA), juvenile dermatomyositis (JDM) or juvenile scleroderma (JScle). Specific B- and T-cell subsets, including regulatory T cells, are considered.

**Methods:** This study was initiated as part of a Dutch-Brazilian collaboration. Patients were included in Brazil (2005-2009), analyses of the samples were performed in the Netherlands. Before and 4-6 weeks after VZV vaccination, blood samples (serum and PBMC) of patients with PRD and healthy controls were collected. Patients who did not respond to the first vaccine were eligible for a second vaccination. VZV-specific antibody (VZV-Ab) concentrations were measured with a standardized Luminex-assay. IFN-gamma ELISPOT assays were performed to assess VZV-specific T cell responses. B-cell maturation, T-B cell interaction and T-cell subsets were assessed using flow cytometry.

**Results:** The cohort consisted of 54 patients (9 oligo-JIA, 22 poly-JIA, 11 sJIA, 7 JDM, 5 JScle) and 18 healthy controls (Table 26). There were no disease flares or overt varicella infections reported after vaccination.

Antibody concentrations of 35 patients and all healthy controls were determined. Responders were defined as having VZV-Ab levels of >0.26 IU/ml after vaccination and/or as a 4-fold or higher increase in VZV-Ab after vaccination. Responder status did not differ between patients and healthy controls (odds ratio (OR) 1,9; 95% Confidence Interval (CI) [-0.08; 1.35]). Seroconversion after vaccination (VZV-Ab levels <0.26 IU/ml prior to and >0.26 IU/mL after vaccination) was similar for patients and controls (OR 2,75; 95% > CI [1.05; 9.72]).

To this point, ELISPOT assays have been performed for 15 patients and 2 healthy controls. Analyses regarding this assay are in progress. Flow cytometry was done for 25 patients and 7 healthy controls. We observed an increase in the percentage of circulating memory B cells ind patients as well as controls. No overt differences between patients and controls were observed so far. Other variations within cell subsets are still under analysis.

**Conclusion:** In this preliminary analysis of the immune responses to VZV vaccination, the response of PRD patients seems comparable to that of healthy controls. Cellular immune responses to the VZV vaccine are under analysis.

**Disclosure of Interest**

None Declared

**Table 26 (abstract P69).** Patient and control characteristics at first dose of VZV-vaccine

	Patients (n = 54)	Healthy controls (n = 18)
Disease type		N/A
Systemic JIA	11 (20%)	
Oligo JIA	9 (17%)	
Poly JIA	22 (41%)	
JScl	5 (9%)	
JDM	7 (13%)	
Age at vaccination *(median, range)*	5 (0–20)	8.5 (3–18)
Medication use		N/A
MTX	98%	
MTX dose *(median, range)*	15 (10–27)	
Corticosteroids	43%	
Steroid dose *(median, range)*	6 (3–20)	
Biologics	6%	
Responder	17	6
Nonresponder	18	12

### P70 Share update – 508 families view on standard of care for children with Juvenile Idiopathic Arthritis in Europe and Israel

#### Ingrid H. R. Grein^1,2^, Silvia Scala^3^, Elisa Patrone^3^, Casper Schoemaker^4^, on behalf of Dutch JIA patient organization, Wendy Costello^5^, on behalf of ENCA, Nico Wulffraat^2^

##### ^1^Pediatric Rheumatology Department, Hospital Pequeno Príncipe, Curitiba, Brazil; ^2^Pediatric Immunology and Rheumatology Department, Wilhemina Children’s Hospital/University Medical Centre Utrecht, Utrecht, Netherlands; ^3^Research Department, Istituto Giannina Gaslini, Genoa, Italy; ^4^Dutch JIA patient organization, Wilhemina Children’s Hospital/University Medical Centre Utrecht, Utrecht, Netherlands; ^5^Chair of European Network for Children with Arthritis (ENCA), Tipperary, Ireland

###### **Presenting author:** Ingrid H. R. Grein

**Introduction:** Since 2013 PRES has initiated an European project called SHARE (Single Hub and Access point for Paediatric Rheumatology in Europe, PI Wulffraat). The project aimed to identify the specific needs for optimal care in pediatric rheumatic diseases (PRD) in each European country, and provide these countries with recommendations for the care of these group of children. Optimal care includes diagnosis, management of disease and providing both drug and non-drug therapies. The European countries were grouped regarding geographic and economic characteristics into West and East-Europe, in order to analyze if there are important differences in health care of these regions. Israel was the first non-European country to participate of the SHARE survey.

**Objectives:** To show an updated data about families view on standard of care for children with Juvenile Idiopathic Arthritis (JIA) in Europe and Israel.

**Methods:** In order to identify the specific needs for the optimal care in PRD, patients and doctors surveys have been performed at pediatric rheumatology centers and patient organisations involved in providing care for these children. Families of patients with diagnosis of JIA according ILAR (International League of Associations for Rheumatology) criteria answered a single time-point survey that encompasses information about specialists referral patterns and referral delays, disease diagnosis, multidisciplinary health care team follow up, access to treatment, participation in clinical research and transition of care to the adult service. Families filled in the questionnaires anonymously on PRINTO (Pediatric Rheumatology INternational Trials Organisation) website.

**Results:** Until May 2016, 508 families from 22 different countries answered the questionnaires: 350 families from West-Europe, 129 from East-Europe and 29 from Israel. The table shows the participant countries and the number of answered surveys of each country. The large difference in the number of answered questionnaires between West and East-Europe is a reflex of their population, as West is three times more populous than East. The families that replied this survey may present a bias to urban population, where the pediatric rheumatology centers are. Patients characteristics regarding gender, age of diagnosis and JIA subtypes are similar in East and West-Europe, as well as in Israel. Families view showed good access to pediatric rheumatologists, uveitis screening and high cost medications in all these regions. The main differences were found in pain and fatigue management, information about transition for the adults service, use of supportive care and knowledge about patient organizations. It seems that these items were less emphasized in East-Europe.

**Conclusion:** The families view is extremely important to identify the topics on standard of care that need improvement, in order to achieve a high quality health care in Europe. A substantial number of replies (508) have been obtained from 22 different countries. The results showed good access to pediatric rheumatology care in Europe and Israel, although tools like patient organizations and transition care were less used by East-Europe. For the future, the aim of SHARE is not only to expand the families survey to a larger number of European countries, but also to another continents.

**Disclosure of Interest**

None Declared

**Table 27 (abstract P70).** See text for description

West-Europe				East-Europe		Asia	
Netherlands	100	Germany	28	Latvia	21	Israel	29
Belgium	16	Austria	1	Lithuania	2		
Denmark	34	Switzerland	1	Czech Republic	20		
Sweden	1	Italy	26	Serbia	5		
United Kingdom	33	Portugal	5	Slovakia	33		
Ireland	26	Spain	20	Slovenia	17		
France	59			Albania	1		
				Greece	30		
TOTAL = 350				TOTAL = 129		TOTAL = 29	

### P71 Young people’s beliefs about research priorities in rheumatology research

#### Suzanne Parsons^1^, Janet McDonagh^2^, Wendy Thomson^2^

##### ^1^Public Programmes Team, Central Manchester University Hospitals’ NHS Foundation Trust, Manchester, UK; ^2^Centre for Musculoskeletal Research, University of Manchester, Manchester, United Kingdom

###### **Presenting author:** Suzanne Parsons

**Introduction:** Involving people of all ages in health-related research is now widely advocated. However, research priorities are still driven primarily by professional agendas, with evidence from the adult literature reporting a mismatch between researcher and patient generated lists of research topics. To date, there have been no studies exploring the research priorities of young people with rheumatic disease.

**Objectives:** The aim of this study was to explore young people’s research priorities for rheumatic conditions to feed into the development of the research strategy for the Barbara Ansell National Network for Adolescent Rheumatology (BANNAR).

**Methods:** Focus groups were undertaken with young people aged 11-24 years across the UK and Northern Ireland. Young people were recruited via members of the BANNAR and relevant national charities. Focus group participants discussed their understanding and beliefs about what should be researched in the following areas; Basic Science; Clinical Medicine and Science; Health Services, Psychosocial, and Public Health. Participants were asked to order these areas in terms of which should receive the most funding. All focus groups were audiotaped and transcribed for thematic analysis.

**Results:** 12 focus groups (11-15 years (n = 5) and 16-24 years (n = 7)) have been held with 58 participants. Young people’s research priorities were influenced by whether they felt that investment in a particular area was likely to: 1. Achieve benefits for all patients, 2. Achieve long term or short term goals (finding a cure versus improving quality of life). Other influences on participants’ priorities were their beliefs about whether research areas are already well funded and whether increasing funding could lead to faster results.

Basic Science was considered to be a key priority. Participants were especially interested in understanding the genetic basis of their condition and how this could affect their prognosis and treatment side effects. However, there was an appreciation that progress in this area was unlikely to happen overnight, and that for this reason a steady stream of funding was likely to be important.

Participants also felt that psychosocial research currently did not receive considerable investment. However, there were varying views on whether it should. Those who felt that the psychosocial aspects of their condition had been neglected tended to feel that Psychosocial research should be a priority. However, a number expressed uncertainty as to who could provide this support believing their doctors were already overstretched. Finally, some participants expressed a wish not to focus too much on the psychosocial aspects of their condition as part of their overall coping strategies. Health Services Research was considered by many to be a low priority, as for the majority of participants, consultant care was their preferred option. GPs were considered not to be a viable support option. However, participants were interested in transitional care arrangements between paediatric and adult care.

For many, the development of new treatments was a low priority, because they were currently happy with their treatment regime, or because they felt uncomfortable with the risk of trying an experimental treatment.

Finally, for all groups Public Health was ranked low. There was a need identified to provide education to a range of audiences about their conditions, e.g. GPs, schools, workplaces, but scepticism existed as to whether or not such educational materials would be utilised.

**Conclusion:** Understanding the research priorities of young people with rheumatic conditions is important to ensure that research strategies are developed that are in tune with their needs and wants. The findings from this work will feed into the research strategy of the BANNAR.

**Disclosure of Interest**

None Declared

### P72 Development of the first video game aimed at children suffering from Juvenile Idiopathic Arthritis

#### Jean-David Cohen^1^, Damien Bentayou^2^, Marc-Antoine Bernard Brunel^2^, Sonia Trope^3^

##### ^1^Public Hospital, Montpellier, France; ^2^CG Artists, Bordeaux, France; ^3^ANDAR patient organization, Paris, France

###### **Presenting author:** Jean-David Cohen

**Introduction:** In France, Juvenile Idiopathic Arthritis (JIA) affects between 2 000 and 4 000 children. The physical and psychological impact of this chronic condition can be significant, including difficulties in the family environment, as well as at school. Whilst the arrival of biotherapies has revolutionised the care of these children, educational support remains necessary to increase their independence and improve their quality of life.

**Objectives:** The development of e-health, in the context of the democratisation of communication tools, has led us to create a Serious Game as educational support for children.

**Methods:** The game has been developed following the ADDIE pedagogical engineering model. The game will be translatable in all languages and made available to foreign associations free of charge. A steering committee was formed, composed of the parents association that initiated the project, a paediatric rheumatologist, and a video game agency.

The National Association for the Defence against rheumatoid arthritis manages the logistics and monitoring. The rheumatologist creates the scientific context, in collaboration with the association. The developers make artistic and gaming suggestions. The committee sets the direction: format, themes, key messages, graphics, animations and game sequences. Learning is validated through multiple choice quizzes. The association KOURIR (association for parents of children with JIA) will test the beta version. Adaptations will then be made before the game’s release.

**Results:** The committee decided to create an adventure game with interactive activities. The following options were selected: The game is developed using the Unity® engine for computers, smartphones and tablets, allowing for use anywhere (Apple, Android). The engine is designed to support all languages. The topics of the hospital process, treatment, complimentary examinations and rehabilitation are all discussed with corresponding key messages.

Many themes are discussed in a single location, the hospital. Two avatars (a boy, a girl) have been created in order to facilitate identification for young players. Skill games and 16 multiple choice quizzes (used to assess knowledge and deliver pedagogical messages) are spread throughout the game. The test phase brought together 7 children of various ages (5 to 16) and parents members of the KOURIR association and led to some adjustments: enhanced interactivity in the rehabilitation part, shortening and improved ergonomics of the swimming-pool game, and simplification of some of the vocabulary.

**Conclusion:** To the best of our knowledge, this is the first serious game dedicated to JIA. Other locations (home, school) could complete this version. An English version is already under development. The warm reception it has received from a number of paediatric rheumatologists means we are hopeful many children will be able to benefit from this initiative, at home or in a structured educational context.

**Disclosure of Interest**

None Declared

### P73 JIA patients have a similar quality of life as healthy peers after three years of specialised care

#### Jens Klotsche^1^, Miriam Listing^1^, Martina Niewerth^1^, Gerd Horneff^2^, Angelika Thon^3^, Hans-Iko Huppertz^4^, Kirsten Mönkemöller^5^, Ivan Foeldvari^6^, Dirk Föll^7^, Kirsten Minden^1^, and ICON study group

##### ^1^Epidemiology unit, German Rheumatism Research Centre, Berlin, Germany; ^2^Kinderklinik, Asklepios, Sankt Augustin, Germany; ^3^Kinderklinik, Medizinischen Hochschule Hannover, Hannover, Germany; ^4^Prof.-Hess-Kinderklinik, Klinikum Bremen-Mitte, Bremen, Germany; ^5^Klinik für Kinder- und Jugendmedizin, Kinderkrankenhauses der Stadt Köln, Köln, Germany; ^6^Hamburger Zentrum für Kinder- und Jugendrheumatologie, am Klinikum Eilbek, Hamburg, Germany; ^7^Klinik für pädiatrische Rheumatologie und Immunologie, Universitätsklinikum Münster, Münster, Germany

###### **Presenting author:** Kirsten Minden

**Introduction:** The treatment of patients with juvenile idiopathic arthritis (JIA) currently aims at achieving an inactive disease state and the best possible quality of life. Whether these treatment goals are achievable in a routine clinical setting can be assessed by data from the Inception Cohort Of Newly-diagnosed patients with JIA (ICON). Herein, patients with recent-onset of JIA have been prospectively observed.

**Objectives:** To study changes in disease activity state and quality of life of patients during and after the first three years of paediatric rheumatology care.

**Methods:** Data of 597 patients with JIA and 306 healthy peers who were included in ICON and followed prospectively for at least three years were considered for this analysis. JIA disease activity was assessed by the cJADAS-10 (clinical Juvenile Arthritis Disease Activity Score). Inactive disease was defined as cJADAS-10 ≤ 1. The PedsQL 4.0 (Pediatric Quality of Life Inventory) Generic Core Scales were applied to measure health-related quality of life (HRQoL) in patients and controls and the PedsQL 3.0 Rheumatology module to assess disease-specific QoL.

**Results:** Patients (69% females) and controls (61% females) had a mean age of 9.8 and 10.1 years at the 3-year-follow-up (3-y-FU). Approximately half of the patients (48%) had oligoarthritis, 27% rheumatoid factor (RF) negative polyarthritis. During the 3rd year of observation, 56% of the patients were on MTX, 26% on biologics and 10% had been treated with systemic glucocorticoids. Patients’ disease activity significantly decreased from a mean cJADAS of 9.5 at first assessment to 2.6 at the 3-y-FU. A total of 271 (45%) patients had an inactive disease, ranging from 47% for persistent oligoarthritis and 56% for RF negative polyarthritis to 79% for systemic JIA. Another 10% reached a minimal active disease at FU. Eighty percent of the patients had received DMARDs up to the 3-y-FU. At the 3-y-FU, 27% did not receive any anti-rheumatic drug. However, only 8% were in remission off drug, another 37% had attained remission on drug.

At inclusion in ICON, patients had a significantly lower HRQoL than controls (mean total scale score 71.3 (SD 18.4) versus 90 (SD 70.5), proxy reports). After three years of rheumatology care, the patients’ HRQoL had clinically meaningful improved and did no longer differ significantly in any health domain from that of controls (total score 86.8 (SD 14.5) versus 89.5 (SD 8.6)). Patients with inactive disease had a higher HRQoL of 92.8 (SD 8.8) at 3-y-FU. Fourteen percent of patients still had high active disease (c-Jadas-10 > 4/8.5 for oligoarthritis/polyarthritis) and a significantly lower mean PedsQL score of 74.0 (SD 17.8). The patients’ disease-specific QoL also significantly improved from ICON inclusion to the 3-y-FU.

**Conclusion:** Current treatment options in paediatric rheumatology care allow achieving an inactive or minimal active disease state and a normalisation of HRQoL in the majority of patients.

**Trial registration identifying number:** ICON is supported by a grant from the Federal Ministry of Research and Education (FKZ: 01ER0812)

**Disclosure of Interest**

J. Klotsche: None Declared, M. Listing: None Declared, M. Niewerth: None Declared, G. Horneff Grant / Research Support from: Pfizer, Abbvie, Roche, A. Thon: None Declared, H.-I. Huppertz: None Declared, K. Mönkemöller: None Declared, I. Foeldvari: None Declared, D. Föll: None Declared, K. Minden Grant / Research Support from: Pfizer, Abbvie

### P74 Auxological features in patients with Juvenile Idiopathic Arthritis (JIA) treated with biologic drugs

#### Achille Marino^1^, Stefano Stagi^2^, Niccolò Carli^1^, Federico Bertini^1^, Teresa Giani^1^, Gabriele Simonini^1^, Rolando Cimaz^1^

##### ^1^Department of Paediatrics, Rheumatology Unit, Anna Meyer Children’s Hospital, University of Florence, Florence, Italy; ^2^Department of Paediatrics, Endocrinology Unit, Anna Meyer Children’s Hospital, University of Florence, Florence, Italy

###### **Presenting author:** Achille Marino

**Introduction:** Juvenile idiopathic arthritis (JIA) is an heterogeneous group of diseases associated with an increase of inflammatory cytokines that may influence child growth.

**Objectives:** To evaluate the auxological features in a cohort of patients with JIA treated with biologic drugs.

**Methods:** This single-center retrospective study evaluated 86 children with JIA (7 systemic, 32 oligoarticular, 31 polyarticular, 6 psoriatic, 10 enthesitis-related arthritis) on long-term (mean 38.5 months; range 6-134) treatment with biologics, and compared them (in a 1:2 ratio) with 172 age and sex-matched healthy controls for height and body mass index (BMI). Patients with endocrinological or other chronic comorbid conditions or on long-term steroid (>3 months) treatment were excluded.

**Results:** All patients received anti-TNFα treatment except 4 patients with systemic JIA; 21 patients received > 1 biologic (2 biologics in 15 patients, 3 biologics in 4 patients and 4 biologics in 2 patients); 13 patients received CTLA-4Ig; one patient received anti-IL6; anti-IL 1 was used in 5 patients.

The target height SDS was not statistically different (0.02 ± 0.66 *vs.* 0.05 ± 0.79 SDS) between JIA patients and controls. The median height SDS at JIA diagnosis was -0.29 ± 0.66 *vs.* 0.09 ± 0.84 SDS of controls (p < 0.005) (-0.24 ± 0.66 SDS for oligoarticular (p < 0.05), -0,17 ± 0.79 SDS for polyarticular (p = NS), -1.33 ± 0.49 SDS for systemic (p < 0.001), -0.71 ± 1.13 SDS for psoriatic (p < 0.05), and 0.30 ± 0.11 SDS for enthesitis-related arthritis (p = NS)).

During disease modifying antirheumatic drugs (DMARDs) treatment, before biologic therapy beginning, the height was: in all JIA patients -0.31 ± 0.66 SDS *vs.* 0.14 ± 0.86 in controls (p < 0.0001); -0,39 ± 0.93 SDS (p < 0.005) for oligoarticular and -0.23 ± 0.79 SDS (p < 0.05) for polyarticular, -1,00 ± 0.13 SDS (p < 0.005) for systemic, -0.65 ± 0.61 SDS for psoriatic (p < 0.05), and 0.10 ± 0.64 SDS for enthesitis-related arthritis (p = NS).

After a follow-up of 6.4 ± 2.3 yrs height SDS in all JIA patients was -0.16 ± 0.89 *vs.* 0.08 ± 0.81 SDS in controls (p < 0.05). Results were -0,08 ± 0.12, SDS (p = NS) for oligoarticular, -0,13 ± 0.89 SDS (p = NS) for polyarticular, -0,58 ± 0.14 SDS (p < 0.05) for systemic, -0.58 ± 0.33 SDS for psoriatic (p < 0.05), and 0.01 ± 0.60 SDS for enthesitis-related arthritis (P = NS).

The Δheight during biologic treatment was statistically significant considering all JIA patients, oligoarticular, systemic and psoriatic (p <0.0001) but not polyarticular and enthesitis-related arthritis. Interestingly, JIA patients showed a significative reduction of BMI SDS after biologic treatment (all patients -0.19 ± 0.47 at diagnosis *vs.* 0.07 ± 0.31 at last follow-up visit, p < 0.005; systemic -0.11 ± 0.31 vs. 0.40 ± 0.49, p < 0.05; polyarticular 0.13 ± 0.30 *vs.* -0.06 ± 0.32, p < 0.05), as opposed to controls whose BMI increased after a similar follow-up period (0.23 ± 0.34 vs. 0.45 ± 0.51, p < 0.0001).

**Conclusion:** Long-term biologic treatments are associated with a significant increase of height SDS and decrease of BMI SDS in JIA patients.

**Disclosure of Interest**

None Declared

### P75 Response to conventional DMARD treatment in a cohort of children with Juvenile Idiopathic Arthritis in Bogota, Colombia

#### Adriana S. Díaz-Maldonado^1^, Sally Pino^2^, Pilar Guarnizo^3^

##### ^1^Pediatric Rheumatology, Care for Kids - Hospital La Misericordia - Instituto Roosevelt, Bogotá, Colombia; ^2^Pediatric Rheumatology, Care for Kids - Hospital Infantil de San José, Bogotá, Colombia; ^3^Pediatric Rheumatology, Care for Kids - Fundación Cardio Infantil - CAYRE, Bogotá, Colombia

###### **Presenting author:** Adriana S. Díaz-Maldonado

**Introduction:** Different treatment options are available for juvenile idiopathic arthritis (JIA) increasing with the time. There are multiple factors, such as JIA subtype that impact in the management of medication and the escalation of the therapy (1).

**Objectives:** To describe the treatment with conventional and biological DMARDs and their response on a cohort of children with JIA in 6 health care institutions in Bogotá, Colombia.

**Methods:** Medical records were retrospectively reviewed derived from 568 patients diagnosed with JIA and attended in 6 health care institutions in Bogotá, Colombia since 2010. Demographic and clinical characteristics were collected as well as the current and previously suspended treatments due to no response. The no response was considered when a patient persists with clinical and laboratories criteria after 6 months of treatment.

The description of the current and previous treatments of patients with JIA was performed according to JIA subtype and Disease-modifying antirheumatic drugs (DMARDs). A descriptive analysis was performed of different treatments and those who required to suspend any biological (bDMARDs) or conventional DMARDs (cDMARDs).

**Results:** 471 patients (83%) received immunosuppressive therapy with DMARDs and 97 (17%) with NSAID or prednisolone. Of these 43% (244) have been received bDMARD.

Among those treated with DMARD, 51% patients not responding to cDMARDs and need bDMARDs. TNF inhibitors are the most frequent (72,2%) followed by interleukin - 6 receptor antagonist (IL-6 RA) (16,8%), co-stimulary (4%), IL-1RA (0,4%) and anti CD20 monoclonal antibodies (5,2%).

42% of patients with Polyarticular JIA rheumatoid factor (RF) positive, Polyarticular JIA rheumatoid factor (RF) negative, psoriatic arthritis and systemic arthritis have been treated with any biologics. Additionally, the 50% of patients with enthesitis related arthritis (ERA) HLA-27B positive have been treated with biological therapy.

Regarding to previous biological therapy, 73,4% had received one biologic, 22,2% two biologics, 2% three biologics and 2% four biologics. All patients mainly belong to polyarthritis and systemic arthritis.

In current treatment, 30,7% of the patients use bDMARDs in monotherapy while the other 69.3% use bDMARDs in combination with one cDMARDs. Conventional DMARDs are used in 58,99% of patients with JIA. Methotrexate is used in 88,9% patients with cDMARDs, followed by sulfasalazine (6,2%) and leflunomide(4,9%). Combination of two cDMARDs is used in 2,77% of patients with cDMARDs.

Ninety one patients achieved remission: 77 patients (84,6%) achieved remission off medications, which 28 patients presented oligoarticular JIA (36,3%), 21 polyarticular JIA (27,2%) and 20 systemic arthritis (25,9%). The highest percentages of remission was oligoarthritis. 13 patients (14,3%) are in remission on medications.

Thirty four patients dropout the treatment where administrative barriers were the mainly cause. Children with oligoarticular and polyarticular JIA were the most frequent in drop outing.

**Conclusion:** Conventional DMARDs are the most frequent drugs used in the treatment of JIA represented mainly by methotrexate. In the case of bDMARDs, the anti TNF inhibitors are the principal drugs. The bDMARDs showed a high frequency of use in positive HLA–B27 ERA patients compared to other subtypes. Polyartticular JIA patients presented the most frequent changes of bDMARDs compared to other JIA subtype

**Disclosure of Interest**

None Declared

### P76 Discrimination between Juvenile Idiopathic Arthritis and leukemic arthritis

#### Alfonso Ragnar Torres-Jimenez^1^, Berenice Sanchez-Jara^2^, Eunice Solis-Vallejo^1^, Adriana Ivonne Cespedes-Cruz^1^, Maritza Zeferino-Cruz^1^, Julia Veronica Ramirez-Miramontes^1^

##### ^1^Reumatologia Pediatrica, Imss Hospital General Centro Medico Nacional La Raza, Azcapotzalco, Mexico; ^2^Hematologia Pediatrica, Imss Hospital General Centro Medico Nacional La Raza, Azcapotzalco, Mexico

**Introduction:**

Acute lymphoblastic leukemia may occur with arthritis before the appearance of blasts in peripheral blood and can be confused with juvenile idiopathic arthritis. In this study, we identified the clinical and laboratory differences between the two entities.

**Objectives:** To determine the clinical and laboratory differences between leukemic arthritis and juvenile idiopathic arthritis at onset of symptoms

Methods:

Patients under 16 years old, both genders, initially presenting to the Pediatric Rheumatology unit, General Hospital, National Medical Center La Raza, with diagnosis of probable JIA, without blast cells in peripheral blood, where the final diagnosis was acute lymphoblastic leukemia or juvenile idiopathic arthritis. The clinical and laboratory manifestations were evaluated. Data were expressed as percentages for each analysis area. Chi square and Relative Risk (qualitative variables) and Mann-Whitney and *T* test for comparison of means between groups The data were analyzed by software using SPSS version 15.0.

**Results:** We analyzed data on 34 patients, 9 with leukemic arthritis and 25 with JIA. The mean age at diagnosis was significantly lower in the group of leukemic arthritis 7.56 vs 10.76 years (p = 0.045), female gender prevailed in both groups (66% and 76%, respectively, p = 0.586), time of onset of symptoms it was lower in the group of leukemic arthritis. Pain intensity, night sweats, weight loss, fever, lymphadenopathy, hepatosplenomegaly and night pain was higher in the group of leukemic arthritis. Remission of pain with analgesics was significantly lower in patients with leukemic arthritis As for laboratory parameters, rheumatoid factor positivity was higher in the group of AIJ without showing statistical significance. The hemoglobin level, the leukocyte and neutrophil count was lower in patients with leukemic arthritis, the lymphocyte count was similar in both groups. The platelet count was higher in JIA patients, the VSG was similar in both, the difference between the value of the PCR was not the value of the DHL was higher in the group of leukemic arthritis without showing statistical significance.

**Conclusion:** Leukemia is an important diferential diagnosis in children presenting with arthritis.

In this study it was found that the presence of pain disproportionate to the degree of arthritis, fever, night pain, which does not yield to the use of analgesics, weight loss and lymphadenopathy, help to discriminate clinically between leukemic arthritis and juvenile idiopathic arthritis.

Useful laboratory parameters are leukopenia, neutropenia, and anemia, the mean platelet count was in low normal value, but lower than in juvenile idiopathic arthritis. The laboratory parameter with most higher relative risk was neutropenia.

**Disclosure of Interest**

None Declared

**Table 28 (abstract P76).** Initial clinical and laboratory findings in patients with leukemic arthritis vs JIA

	Leukemic arthritis (n = 9)	JIA (N = 25)	*P* value	RR (IC 95%)
Pain intensity (0–10)	10	6.6 (4–10)	*0.0001	
Night pain	66%	4%	*0.0001	7.7 (2.5–23)
Fever	66%	12%	*0.001	5 (1.7–17.6)
Weight loss	78%	16%	*0.001	7.3 (1.8–29.6)
Lymphadenopathy	88%	4%	*0.0001	22 (3.2–153.7)
Anemia Hemoglobin g/dL	56%	8%	*0.002	4.8 (1.7–13.3)
	10.9 (8.3–12.9)	13.1 (10.2–16.4)		
Leukopenia Leukocytes mm^3^	67%	0%	*0.0001	9.3 (3–27)
	3555 (1610–5530)	9750 (5190–24630)		
Neutropenia Neutrophil mm^3^	88%	0%	*0.0001	25 (3.3–170)
	473 (0–1820)	6456 (2270–22130)		
Thrombocytopenia Platelets ×10^3^/mm^3^	44%	0%	*0.0001	6 (2.6–13)
	171 (53–296)	375 (207–661)		

### P77 Rheumatoid factor positive polyarticular JIA with pulmonary langerhans cell histiocytosis- a chance association or a causal relationship?

#### Ankur Kumar^1^, Anju Gupta^1^, Deepti Suri^1^, Amit Rawat^1^, Nandita Kakkar^2^, Surjit Singh^1^

##### ^1^Pediatrics, PGIMER, Chandigarh, India; ^2^Histopathology, PGIMER, Chandigarh, India

###### **Presenting author:** Ankur Kumar

**Introduction:** Langerhans cell histiocytosis is a multisystem disease of an unknown etiology characterized by infiltration and accumulation of CD1a+/ CD 207+ histiocytes in various organ systems. Association of LCH with autoimmune or inflammatory disorders has rarely been reported.

**Objectives:** To describe the case of a young girl with juvenile idiopathic arthritis and pulmonary LCH and to discuss the possible common pathogenetic mechanism for both diseases.

**Methods:** A 10 year old girl was symptomatic for past 1 year. Her illness started with left ankle swelling with pain, redness, restriction of movement and early morning stiffness. Over the next 5 months she had progressive involvement of right ankle joint, small joints of bilateral foot and bilateral wrist joints. On examination, she had swelling in bilateral ankle and wrist joints. There was redness and increased temperature over the surface associated with synovial thickening and restriction of passive range of movement. Her investigations revealed (Table 29) thrombocytosis; elevated ESR and C- reactive protein; positive antinuclear antibody (ANA) and positive rheumatoid factor (RF). Chest X-ray revealed cystic changes in bilateral lung fields which were confirmed on CT chest which showed multiple well defined cysts with variable sizes distributed throughout the parenchyma in bilateral lung fields. An open lung biopsy was performed to confirm the diagnosis. Histopathology examination of the resected lung tissue revealed lymphomononuclear inflammatory infiltrate in the interstitium with collection of large atypical cells around the peribronchiolar region which were showing oval to round nuclei and moderate to abundant vacuolated cytoplasm, these cells were positive for CD1a. Skeletal x-ray revealed no lytic lesions and bone marrow examination was also normal. There was no evidence of any other organ involvement with LCH.

**Results:**

For arthritis, she was initiated on oral naproxen, a short course of oral prednisolone and subcutaneous methotrexate; which led to marked improvement in her joint symptoms. For management of LCH, as she was asymptomatic with predominantly cystic lung changes and no nodular changes on CT chest; hence a possibility of chronic fibrotic disease was considered and a decision not to give any specific therapy for LCH was taken

**Conclusion:** Our case highlights a rare and previously unrecognized association of juvenile idiopathic arthritis with pulmonary LCH. It also supports the inflammatory origin of Langerhans cells

**Disclosure of Interest**

None Declared

**Table 29 (abstract P77).** Investigations in the index case

Investigation	Result
Haemoglobin (gm/L)	119
WBC counts (×10^9^ cells/L)	11
Differential counts	N_63_/L_23_/M_7_/E_7_
Platelet counts (×10^9^ /L)	467
ESR (mm/hr)	49
CRP (mg/dL)	18
AST (U/L)	49
ALT (U/L)	30
ALP (U/L)	190
Anti nuclear antibody	3+ homogenous
Rheumatoid factor (U/ml)	
At diagnosis	181 (normal range: 0–19)
4 months later	199

### P78 The rate of obesity and overweight among Turkish children with Juvenile Idiopathic Arthritis

#### Balahan Makay, Özge A. Gücenmez, Erbil Ünsal

##### Pediatric Rheumatology, Dokuz Eylül University Hospital, İzmir, Turkey

###### **Presenting author:** Balahan Makay

**Introduction:** The increasing rate of overweight among children and adolescents is a serious public health concern. Children with physical disabilities may have an increased risk for overweight and overweight may be a risk factor for inflammatory arthritis, as it is in adults with psoriatic and rheumatoid arthritis. However; there are only a few studies about obesity and juvenile idiopathic arthritis (JIA) in the literature.

**Objectives:** To investigate the rate of being overweight/obese among Turkish children with JIA at the time of diagnosis and to assess whether increased BMI is associated with different subgroups of JIA.

**Methods:** The hospital charts of 325 patients diagnosed as JIA according to the International League of Association of Rheumatology (ILAR) criteria in a 10-year period in a tertiary hospital were retrospectively reviewed. Demographic and clinical findings and JIA subgroups were recorded. Center for Disease Control BMI-for-age percentile charts were used. Patients were classified as having “increased weight” (BMI ≥ 85^th^ percentile), “healthy weight” (5^th^ ≤ BMI < 85^th^ percentile) or “underweight” (BMI < ^5th^ percentile) according to their BMI at baseline.

**Results:** The mean age at diagnosis was 9.1 ± 4.9 years. There were 182 girls and 143 boys. The median delay time to dignosis was 5 months (interquartile range 2-12 months). 105 patients (32.3%) had persistent oligoarthritis, 81 (24.9%) had enthesitis-related arthritis (ERA), 59 (18.2%) had RF (-) poliarthritis, 29 (8.9%) had systemic arthritis, 21 (6.5%) had undifferentiated arthritis, 16 (4.9%) had psoriatic arthritis, 9 (2.8%) had extended oligoarthritis and 5 (1.5%) had RF (+) poliarthritis. 244 patients (75%) had healthy weight, 56 (17.3%) had increased weight and 25 (7.7%) were underweight. The 46.4 percent of the patients with increased weight belonged to ERA subgroup. The rates of increased BMI according to JIA subgroups were given in Table 30. In univariate analyses; male gender, age at diagnosis and ERA subgroup were found to be significantly associated with increased BMI (p = 0.013, p < 0.001, and p < 0.001, respectively). However; multivariate backward stepwise regression analyses revealed that only age at diagnosis was significantly associated with increased BMI. Increased BMI was not significantly associated with diagnostic delay time, number of involved joints and erythrocyte sedimentation rate.

**Conclusion:** This is the first study to investigate the prevalence of overweight in children and adolescents with JIA at the time of diagnosis without any treatment. We could not find any difference between the overweight and obesity rates of patients with JIA and children from the general population. This study revealed that 17.3% of JIA patients had increased weight. A similar overweight/obese rate of 16-21% for children and adolescents in the general population in Turkey was found previously. In a recent study of Schenck et al, systemic JIA and ERA patients showed significantly higher overweight rates compared to other subgroups. We also found a significantly higher rate of overweight in ERA when compared to other subgroups. However; when it was adjusted for age and gender, increased BMI was not significantly associated with any certain subgroup.

**Disclosure of Interest**

None Declared

**Table 30 (abstract P78).** See text for description

	Healthy weight	Increased weight
Persistent oligoarthritis	95 (90.5)	10 (9.5)
ERA	55 (68)	26 (32)
Poliarthritis RF (+)	46 (78)	13 (22)
Systemic	28 (90.5)	1 (9.5)
Undifferentiated	17 (81)	4 (19)
Psoriatic	16 (100)	0 (0)
Extended oligoarthritis	8 (89)	1 (11)
Poliarthritis RF (-)	4 (80)	1 (20)

Data were presented as *n* (% within subgroup)

### P79 Patient partnership and disabkids in the Swedish Pediatric Rheumatology Registry

#### Bo Magnusson^1^, Karina Mördrup^1^, Anna Vermé^1^, Christina Peterson^2^, and Board of the Swedish Pediatric Rheumatology Registry

##### ^1^Paediatric Rheumatology Unit, Astrid Lindgren Children’s Hospital, Karolinska University hospital, Stockholm, Sweden; ^2^Hälsohögskolan, Jönköping, Sweden

###### **Presenting author:** Bo Magnusson

**Introduction:** The healthcare of today is facing higher demands on coproduction of it’s service with partnership between patients and healthcare providers. Good outcomes, most recognise, are more likely if the clinician and patient communicate effectively. Real time patient feedback facilitated by Patient Reported Measures (PRMs) can be used to enable true co-production of care. DISABKIDS is a European project that has developed with focus on health related quality of life (HRQoL). The Swedish Pedatric Rheumatology Registry started in 2009. Today over 2700 patients with JIA are included. The coverage is high. 65% of all Swedish patients with JIA (0-17 years of age) are included and above 90% of all patients with JIA on biological treatment. The registry is a tool for both patients and care providers in promoting valuabel care in many ways. National and local data are provided promoting good and equal care for all patients. DISABKIDS is selected in the registry as instrument for HRQoL.

**Objectives:** To describe the Swedish experience of the process of co-production with patients, families and patient representativs. Evaluate and describe the differences in the assessments of DISABKIDS as HRQoL and how to use the data to reveal individual problems and connect to the planning of further care in partnership with the patient.

**Methods:** During the project, an improvement-coach led group seminars and the discussions which constituted a base for prioritized areas. The sessions resulted in two defined improvement areas.

Results:

To develop a visualization platform for patient reported data as well as other biomedical aspects. Further, to describe the needs in care for children and adolescents with pediatric rheumatic diseases.

1. The visualization platform was built on a Feed Forward System (FFS) for patients, giving support for communication in clinical settings. This was based on the patient portal with a personal overview from registry entered data. Then a development of web-applications for patients with data exported to the registry of Patient Reported Measures were constructed. PRMs included pain, global assessment patient, health impairment, ability to be at school/preschool and my personal measure.

Children are positive to share their results and can illustrate their HRQOL better to their provider during consultations.

National results from the HRQoL assessment of DISABKIDS and DISABKIDS RA (impact and understanding) per dimension:

**Conclusion:** Patient partnership and co-production will enhance registry quality and give added value for both patients and care providers in promoting efficient and data based decisions in daily clinical work.

**Disclosure of Interest**

None Declared

**Table 31 (abstract P79).** See text for description

Independence	Innerstrength	Social Inclusion	Social Equality	Physical ability	Physical Treatment	Total score	Impact	Understanding
76%	47%	67%	67%	65%	64%	58%	2.7/5	2.6/5

### P80 Diagnostic duration and access to care in JIA: a pilot study in the University Hospital of Saint Etienne (France)

#### Caroline Freychet^1^, Jean Louis Stephan^2^, Michael Hofer^3^, Alexandre Belot^4^

##### ^1^Pediatrics, University Hospital of St Etienne, St Etienne, France; ^2^Pediatrics, University Hospital of St Etienne, Saint Etienne, France; ^3^Pediatrics, University Hospital of Lausanne, Lausanne, Switzerland; ^4^Pediatrics, University Hospital of Lyon, Lyon, France

###### **Presenting author:** Caroline Freychet

**Introduction:** Many studies in pediatric rheumatology show that there is a therapeutic « window of opportunity » early in the disease course to prevent joint or ocular damage in the context of juvenile idiopathic arthritis (JIA). In Europe, despite facilitated access to health care, children with JIA are referred to pediatric rheumatology centres with significant delay. Little is known about the reason for such a delay while strong efforts have to be made to identify each component of diagnostic delay.

**Objectives:** To assess in JIA patients the diagnostic pathway and to determine which parameters may influence the delay to diagnosis.

To evaluate the parents’ feelings and adjustments during this period.

**Methods:** From September 2015 to May 2016, we have carried out, in the university hospital of Saint-Etienne, a prospective explanatory research about new JIA patients. The method was both qualitative and quantitative using parents’ semi-structured individual interviews and medical data about the diagnostic pathway. In addition to clinical data, we have included social data: parents’ profession and education level. Data on the care course before the diagnosis have also been collected (date of the first symptoms, date of diagnosis, specialty of the various physicians met and date of the consultations).

**Results:** According to the ILAR classification we have included 19 new JIA cases: 7 oligoarthritis, 6 enthesitis-related arthritis (ERA), 3 polyarthritis RF +, 3 systemic arthritis (sJIA). Median time between first symptoms and the date of diagnosis was 29 weeks (2-114). Median time between the first symptom and the first medical consultation was 1 week (0-40) whereas median time between the first medical consultation and the diagnosis was 22 weeks (0-108). The median number of physician met before the consultation with the pediatric rheumatologist was 3 (2-6), among the following heath care providers: general pediatrician, general practitioner, emergency doctor, orthopedic surgeon, adult rheumatologist and rehabilitation doctor. In 15 cases the first physician was a general practitioner, in the other 4 cases he was an emergency doctor.

Patients with ERA had the longest delay to diagnosis (median 55 weeks (8-106)), whereas children with sJIA had the shortest interval (median 2 weeks (1-3)).

The delay to diagnosis did not vary depending on the parents’ socioeconomic groups or their education level.

Semi-structured individual interviews have been done with 9 families (13 parents) and pointed out that this waiting time was very stressful for each family with a fear of irreversible process with ankylosis. 8 families used complementary and alternative medicines (7 homeopathy, 2 acupuncture and 2 osteopathy). 8 families needed help in the day-to-day life, 2 mothers stopped their jobs and one got a partial time work.

**Conclusion:** In spite of the fact that patients have quickly recourse to health care, delay to diagnosis for JIA remains very long: strong efforts have to be made to organize targeted training programs for all practitioners involved in the care pathway of these children and to communicate on the on-going national program on rare diseases in France.

The identification of the various components of the delay to diagnosis may enable practitioners to elaborate care course management guidelines and public governmental bodies to set up adapted program for rare disease.

By the mean of the JIR cohort (juvenile inflammatory rheumatism cohort), we intend to carry out a broader study about delay to diagnosis and access to healthcare and treatment for JIA in France and in Switzerland.

**Disclosure of Interest**

None Declared

### P81 Effectiveness of knee splintage under general anaesthetic post interarticular injection in children suffering from JIA

#### Cathryn E. Harkness^1^, Madeleine Rooney^2^, Leanne Foster^1^, Emma Henry^1^, Pauline Taggart^2^

##### ^1^Occupational Therapy Musgrave Park Hospital, Belfast, United Kingdom, ^2^Belfast Health & Social Care Trust, Belfast, United Kingdom

###### **Presenting author:** Cathryn E. Harkness

**Introduction:** Flexion contractures, particularly of the knee, are a recognised complication of JIA. In the UK there is currently no standard protocol practiced for the treatment of such contractures. In an audit of 10 UK centres (5 respondents replied), one other centre splinted in theatre post interarticular steroid injection (IASI)

We report our audit findings of post injection splinting in children with JIA.

**Objectives:**

- To establish what the current practices are in the UK in relation to standard protocols for splinting the knee post inter articular steroid injection

- To compare the ROM measurements of the knee prior to and post IASI and establish the effectiveness of splintage while under GA

- To develop standards to enhance clinical practice

**Methods:** We reviewed the outcome of range of movement following splinting of the knee while under general anaesthetic from 2013-2015

The knees were injected with Triamcinolone Hexacetonide 20-40mgs according to size. A cylindrical fibreglass cast with a reinforced back slab was then applied under anaesthetic, with the knee held in the greatest achievable level of extension. The splints were opened, secured, and left in place for 24 hours. They were worn nightly thereafter. Parents were taught a daily physio regime for strengthening and stretching, and physio treatment was provided on a weekly basis for up to 6 months. Knee extension was measured at various time points - actively pre-theatre, passively in splint and actively post treatment.

**Results:** 9 patients and 10 joints were included in the review. Ages ranged from 1-6 years, with a mean age of 2.5 years. Of the 10 patients, 7 had a diagnosis of Oligo JIA and 3 of Poly-Articular JIA, with a mean disease duration of 2.5 years (ranging from 6 months to 6 years).

All 10 of the knees treated showed significant improvement in active extension post injection / splintage. The mean improvement was 83%, and this improvement was maintained unless the knee flared. Flexion was not compromised by splinting. Parents reported good compliance with initial continual use and subsequent nocturnal splintage.

**Conclusion:** We are one of only 2 centres who reportedly use splinting following joint injection under anaesthetic for knees with sustained flexion contractures. Our results concur with those published by GOSH that rapid and sustained correction of contractures is achieved and splintage is well tolerated by children. However we have no comparator group to evaluate the efficacy of such intervention over IASI and physiotherapy alone. Splinting in theatre prolongs the use of anaesthetic by 15-20 minutes. Furthermore the type and dose of steroid used in each centre may be a major confounder.

It is essential that we undertake further analysis of practices in the UK, and ideally undertake a prospective randomised controlled trial comparing injection plus physio alone with injection, splinting and physio where the injected steroid and dosage regime are controlled.

**Disclosure of Interest**

None Declared

**Table 32 (abstract P81).** Comparing active ROM pre-injection/splintage with best active ROM achieved

Jt 1	90%
Jt 2	80%
Jt 3	92%
Jt 4	67%
Jt 5	90%
Jt 6	67%
Jt 7	80%
Jt 8	100%
Jt 9	63%
Jt 10	100%

The mean is 82.9%

### P82 Analysis of disease activity and disease damage in patients with Juvenile Idiopathic Arthritis: single center experience

#### Dogan Simsek^1^, Coskun F. Ozkececi^2^, Esra Kurt^1^, Gokalp Basbozkurt^2^, Faysal Gok^1^, Erkan Demirkaya^1^

##### ^1^Pediatric Rheumatology, Gulhane Military Medical Academy, Ankara, Turkey; ^2^Pediatrics, Gulhane Military Medical Academy, Ankara, Turkey

###### **Presenting author:** Dogan Simsek

**Introduction:** Juvenile idiopathic arthritis (JIA) is a group of disease defined by the presence of continuous inflammation of joints with unknown etiology. JIA is the most important reason of loss of ability and the most common rheumatic disease in childhood.

**Objectives:** In this study, it was aimed to determine disease activity, articular and extra-articular damage index of patients with JIA who were followed-up in our outpatient clinic.

**Methods:** In this prospective study, patients diagnosed with JIA according to the ILAR classification criteria, who were being followed-up in pediatric rheumatology clinic at Gulhane Military Medical Academy between September 2014 and May 2016 were included. Demographics, clinical and laboratory features were recorded on a standardized form in addition to arthritis damage index and disease activity [Physician global assessment (PGA)] were calculated for each patient at each visit.

**Results:** A total of 96 patients [58 girls (60,4%), 38 boys (39,6%)] with a mean age of 9,2 ± 5,13 years were included. The distribution of subtypes of JIA were as follows; persistant oligoarticular JIA with 58,3% (n = 58), enthesitis related arthritis with 19,8% (n = 19), RF (-) polyarticular JIA with 7,3% (n = 7), RF (+) polyarticular JIA with 2,1% (n = 2), extended oligoarticular JIA with 4,2% (n = 4), psoriatic arthritis with 2,1% (n = 2).

The most commonly affected joint was knee at the diagnosis (57,3%, n = 55). Uveitis rate was 5,2% (n = 5, all the patients with persistent oligoarticular JIA). PGA of disease activity was found 3,1 ± 1,79 for all patients in the first visit, and 0.73 ± 1.219 at he last visit (p < 0.01). Highest PGA recorded was 5 ± 1,41 with psoriatic arthritis group and lowest PGA recorded was 2,69 ± 1,51 with persistant oligoarticular JIA subtype. Mean articular JADI score was 1,41 ± 4,9 for all subtypes at the first visit and 0.3 ± 1.14 at the last visit (p < 0.01), the highest score was 10,2 ± 18,66 in systemic JIA, lowest score was 0,11 ± 0,32 in enthesitis related arthritis subtype. There was no statistical significance for the extra-articular JADI scores between the first visit and the last visit of the patients with JIA.

**Conclusion:** During the follow-up of our patients improved with the various treatment modalities. Activity (PGA) and JADI scores of the patients decreased from the first visit to last visit. Evaluating damage and activity in children with JIA by using outcome scores is helpful to see the response to treatment and contribute our daily clinical practice.

**Disclosure of Interest**

None Declared

### P83 Serum levels of myeloid-related proteins 8 and 14 in oligoarticular and polyarticular onset of Juvenile Idiopathic Arthritis

#### Daiva Gorczyca^1^, Jacek Postępski^2^, Aleksandra Czajkowska^3^, Bogumiła Szponar^3^, Mariola Paściak^3^, Anna Gruenpeter^4^, Iwona Lachór-Motyka^4^, Daria Augustyniak^5^, Edyta Olesińska^6^

##### ^1^Third Department and Clinic of Paediatrics, Immunology and Rheumatology of Developmental Age, Wroclav Medical University, Wroclaw, Poland; ^2^Department of Paediatric Pulmonology and Rheumatology, Medical University of Lublin, Lublin, Poland; ^3^Hirszfeld Institute of Immunology and Experimental Therapy, Polish Academy of Sciences, Wroclaw, Poland; ^4^Department of Paediatric Rheumatology, John Paul II Paediatric Centre, Sosnowiec, Poland; ^5^Department of Pathogen Biology and Immunology, Institute of Genetics and Microbiology, Wroclav University, Wroclaw, Poland; ^6^Department of Paediatric Pulmonology and Rheumatology, Children’s University Hospital, Lublin, Poland

###### **Presenting author:** Edyta Olesińska

**Introduction:** The myeloid related protein (MRP) complex 8/14 is released from activated monocytes and phagocytes. A recently published data have shown MRP8/14 potential role as predictor of disease activity and response to treatment in rheumatic and other inflammatory diseases.

**Objectives:** To analyze serum levels of MRP8/MRP14, and correlate them with disease activity in oligoarticular and polyarticular onset of juvenile idiopathic arthritis (JIA).

**Methods:** 44 children with oligoarticular and polyarticular onset of JIA and 25 healthy children as controls were enrolled to the study. The JIA patients were categorized into subgroups according to disease activity and duration from diagnosis. Active JIA was defined as presence of at least one joint swelling or limitation of joint motion and pain, or warmth or tenderness. Inactive JIA was defined as no active arthritis, no active uveitis, normal erythrocyte sedimentation rate (ESR) or/and C-reactive protein (CRP), morning stiffness ≤ 15 min. Short-lasting disease was defined as disease duration less than 3 months from diagnosis and long-lasting disease as more than 3 months from diagnosis. The following clinical and laboratory measures were collected: duration from disease diagnosis, number of swollen joints and with limitation of motion, pain (VAS, 0-10), Physician Global Assessment (PGA) (VAS, 0-10), patient’s/parent’s disease activity (VAS, 0-10), CHAQ, duration of morning stiffness, ESR, CRP, complete blood count (CBC). ELISA was used to measure serum levels of MRP8/MRP14. Receiver oprating curve (ROC) analysis was performed.

**Results:** Median serum levels of MRP8/14 in JIA patients, and both in oligo- and poly-JIA were significantly higher compared to healthy controls. Median serum levels of MRP8/14 were significantly higher in active and lower in inactive JIA patients compared to healthy controls. Median serum levels of MRP8/14 in active JIA patients were significantly higher compared to inactive JIA patients. Median serum levels of MRP8/14 in JIA patients with short-lasting disease and with long-lasting disease were significantly higher compared to healthy controls. Median serum levels of MRP8/14 in JIA patients with short-lasting disease were significantly higher than in JIA patients with long-lasting disease. The receiver operating curve (ROC) analysis for the MRP8/14 showed that when the cutoff level of MRP8/14 was set at 3800 ng/mL, the sensitivity and specificity for the JIA patients were 61.4% and 84.0%, respectively. Median serum MRP8/14 positively correlated with PGA, VAS of patient’s/parent’s disease activity, VAS pain, CHAQ, morning stiffness and laboratory variables of inflammation such as CRP, ESR, white blood count, and platelet count. Serum MRP8/14 negatively correlated with disease duration.

**Conclusion:** Serum MRP8/14 concentrations represent a potent parameter to monitor inflammation in patients with oligo- and polyarticular onset of JIA. The elevation of serum MRP8/14 in patients with long-lasting disease may indicate persistent stimulation of immune response. Further studies are needed to confirm our pilot study. This work was supported by the Wroclaw Medical University (Grant No ST-653).

**Disclosure of Interest**

None Declared

### P84 Juvenile Idiopathic Arthritis in mono-zygotic twin

#### Emediong S. Asuka^1^, Tatyana Golovko^1^, Samuel U. Aliejim^1^

##### ^1^Pediatrics Department, Vasyl Karazin Kharkiv National University, Kharkiv, Ukraine

###### **Presenting author:** Emediong S. Asuka

This abstract is not included here as it has already been published.

### P85 Diagnostic pitfalls in Juvenile Idiopathic Arthritis: beyond inflammation

#### Emilio Inarejos Clemente^1^, Estibaliz Iglesias Jimenez^2^, Joan Calzada Hernandez^2^, Sergi Borlan Fernandez^2^, Clara Gimenez Roca^2^, David Moreno Romo^3^, Natalia Rodriguez Nieva^4^, Juan Manuel Mosquera Angarita^2^, Jordi Anton Lopez^2^

##### ^1^Radiology, Hospital Sant Joan de Deu, Barcelona, Spain; ^2^Rheumatology, Hospital Sant Joan de Deu, Barcelona, Spain; ^3^Orthopedics, Hospital Sant Joan de Deu, Barcelona, Spain; ^4^Rehabilitation, Hospital Sant Joan de Deu, Barcelona, Spain

###### **Presenting author:** Emilio Inarejos Clemente

**Introduction:** Juvenile idiopathic arthritis (JIA) is the most common connective tissue disease in the pediatric population with a prevalence of approximately 1 in 1,000 children younger than 16 in North America, and girls being more frequently affected than boys. JIA predominantly involves the large joints, most frequently the knees, wrists, and ankles. Magnetic resonance imaging (MRI) is a relatively new imaging technique to confirm the internal derangement of the joints on patients affected with JIA. Moreover, MRI has the advantage of displaying both soft tissue and bone and we found to be very accurate in the assessment of disease activity and to depict the more frequent pitfalls encountered on these patients, which can lead to a misdiagnosis.

**Objectives:** 1. To describe the more frequent diagnostic pitfalls on patients with JIA on imaging, especially on MRI.

2. To illustrate the radiographic appearance of JIA patients from head to toe, highlighting the differences between active and chronic disease.

3. To review the MR protocol and imaging techniques to study patients with JIA.

**Methods:** We retrospectively review all available hospital medical records, including Electronic Patient Chart (EPC) and clinical database of our patients with diagnosis of JIA. We also review all MR examinations and radiology reports of these patients. Clinical information collected included age, sex, clinical history, JIA subtype, disease duration, history of uveitis and ANA test result. All of our patients were examined with a 1.5 T Magnet (Signa HD; General Electric Medical Systems, Milwaukee, Wisconsin, USA). MR protocol varied depending on the joint examined, but all of them included contrast administration.

**Results:** We found out active inflammation on MR examination according with clinical examination in a significant number of patients. Nevertheless, there were patients with clinical suspicion of active inflammation and no radiological signs of active disease. These patients had other entities not related to JIA. The following joints were affected with different diseases. Temporomandibular joint: disk subluxation. Cervical spine: rotatory subluxation and Langerhans cell histiocytosis. Wrist: scapholunate dissociation and a ganglion cyst. Lumbar spine: spondylolisis with no spondylolisthesis. Hips: apophyseal injuries, enthesitis not related to arthritis and an osteoid osteoma on the acetabulum. Knee: Oschgood-Schlater disease, discoid meniscus tear, a giant cell tumor of the tendon sheath and a stress fracture. Ankle: tarsal coalition and stress fracture.

**Conclusion:** MRI with contrast is the technique of choice and the most sensitive one for the diagnosis and management of patients with JIA. MR is crucial to show the most frequent pitfalls on patients with JIA, due to the ability of displaying both soft tissue and bone.

**Disclosure of Interest**

None Declared

### P86 Insulin resitance and other cardiovascular disease risk factors in Juvenile Idiophatic Arthritis

#### Esmeralda Nuñez-Cuadros^1^, Gisela Diaz-Cordovés^1^, Rocío Galindo-Zavala^1^, Antonio Urda-Cardona^2^, Antonio Fernández-Nebro^3^

##### ^1^Pediatric Rheumatology Unit, Regional Universitary Hospital of Málaga, Málaga, Spain; ^2^Pediatric Department, H. Materno-Infantil. HRU de Málaga, Málaga, Spain; ^3^Rheumatology Department, Regional Universitary Hospital of Málaga, Málaga, Spain

###### **Presenting author:** Esmeralda Nuñez-Cuadros

**Introduction:** It is postulated that insulin resistance (IR) constitutes the key metabolic platform for the development of cardiovascular disease on adults. The role of chronic inflammation on IR has been shown in other diseases as rheumatoid arthritis. Certain subtypes of juvenile idiopathic arthritis (JIA) represent a state of systemic inflammation; it could lead to IR and other cardiovascular disease risk factors (CDRF). Paediatric studies on the subject are limited.

**Objectives:** To describe the IR prevalence in JIA paediatric patients and to assess the possible involved factors. Furthermore, it analyses the prevalence of classical RFC, as well as metabolic syndrome.

**Methods:** Observational cross-section study in Spanish children from 4 to 16 years old affected by the most frequent subtypes of JIA (oligoarticular, polyarticular and systemic) according to ILAR classification, monitored by a Pediatric Rheumatology Unit between July 2014 and July 2015. Monoarticular forms and patients with other concomitant treatments or diseases were excluded.

Anthropometric, clinical and treatment data were recorded. Blood test was performed, included every metabolic syndrome parameters and 3 adipokines (leptin, adiponectin and resistin). Blood pressure and validated surveys about Mediterranean diet (KIDMED) and physical activity (PAQ-C/PAQ-A) were made also. IR was defined as a homeostatic model assessment (HOMA-IR) on the 90th percentile of healthy children in a Spanish cohort (García Cuartero *et al* 2007).

**Results:** 88 patients were enrolled, 70,5% female with an average age of 11 years old. Their epidemiological, clinical and regarding treatment received characteristics are recorded in Table 33.

The IR prevalence was 24.4%. Regarding classical CDRF, 6 (7.5%) were obese, 12 (15%) overweight, 16 (18.3%) arterial hypertension (AH), 14 (16.8%) dyslipidemia, mainly HDL

About the diet and physical activity surveys, 41.3% followed an optimal Mediterranean diet (KIDMED score ≥ 8), being the most of the cases improvable (score 4-7). Our patients performed moderate physical activity during free time (PAQ-C/PAQ-A score > 3), however, statistical differences were not significant between JIA subtypes.

A linear multivariate regression analysis was performed. IR was associated (R^2^ = 0,512) with hip circumference (cm) (β = 0.04; p 0.008), HbA1C% (β = 2.845; p 0.008) and leptin (ng/ml) (β = 0.036; p 0.007). Clinical activity duration was the only predictor factor, not having significant correlation with the subtype, received treatment or disease activity during the study.

**Conclusion:** Our data suggest that the patients with JIA have an IR prevalence similar to the Spanish healthy children (adjusted by age, gender and pubertal stage). The only factor related to the disease which directly influenced the IR grade was the clinical activity overall duration, hence the importance of an early control of the disease to avoid this CDRF in future.

**Disclosure of Interest**

None Declared

**Table 33 (abstract P86).** Clinical and treatment characteristics

N = 88	
JIA subtype, n (%)	
Systemic 10 (11,4)	
Persistent oligoarticular 43 (48.9)	
Extended oligoarticular 14 (15.9)	
Polyarticular 21 (23.8)	
Disease duration (years), mean ± SD	6,3 ± 3,6
Inflammatory activity duration (weeks), median (IR)	53 (32–109)
Synthetic DMARDs treatment, n (%)	85 (96.5)
Biological DMARDs treatment, n (%)	40 (45.5)
Systemic corticosteroids, n (%)	79 (89.7)
Duration corticosteroids (days), median (IR)	122 (55–178)
Median corticosteroids dose (mg/kg/day), median (IR)	0.34 (0.21–1.15)

### P87 Prospective analysis of the immunogenic response in JIA patients (paediatric and adult) on antitnf treatment

#### Estefania Quesada-Masachs^1^, Daniel Álvarez de la Sierra^2^, Marina Garcia Prat^2^, Mónica Martínez Gallo^2^, Ricardo Pujol Borrell^2^, Sara Marsal Barril^3^, Ana M. Marín Sánchez^2^, Consuelo Modesto Caballero^1^

##### ^1^Paediatric Rheumatology Unit, Vall d’Hebron University Hospital, Barcelona, Spain; ^2^Immunology, Vall d’Hebron University Hospital, Barcelona, Spain; ^3^Rheumatology, Vall d’Hebron University Hospital, Barcelona, Spain

###### **Presenting author:** Estefania Quesada-Masachs

**Introduction:** There is some evidence that clinical response in patients with chronic arthritis treated with antiTNF agents can be influenced by their immunogenicity. This particular has not been fully studied in patients with juvenile idiopathic arthritis (JIA).

**Objectives:** To evaluate the immunogenic response against antiTNF agents and its possible impact on treatment efficacy in patients with JIA.

**Methods:** Seventy-three patients (40 children and 33 adults) fulfilling ILAR criteria of JIA were included. All of them were on treatment with Etanercept (ETN), Adalimumab (ADA) or Infliximab (IFX) +/- Methotrexate (MTX). Demographical and disease characteristics were recorded at baseline. Clinical outcome was prospectively assessed every 6 months (JADAS10/27/71, patient or parents VAS, physician’s VAS, CHAQ/HAQ, ESR, CRP, number of swollen, painful and limited joints). Patients were tested for the presence of both, serum drug levels and antidrug antibodies levels at baseline and every 6 months.

**Results:** Thirty-one paediatric patients (77.5%) needed combined treatment (antiTNF + MTX) *vs* 18 patients (54.4%) in the adult group. Furthermore, paediatric patients followed an intensified antiTNF therapy protocol more frequently than adult patients (54% *vs* 39.33%; p = 0.044). A total of 189 determinations for the assessment of immunogenic response against antiTNF therapy were analysed. No differences were found between children and adults in the prevalence of antidrug antibodies or in the circulating antiTNF levels. No antidrug antibodies were found neither in the 107 determinations of patients following ETN treatment nor in the 6 determinations of patients on IFX. Just 5 (17%) of the 29 patients on ADA treatment produced anti-ADA antibodies at least once during the follow-up period. Regarding the 77 ADA determinations, patients who were on monotherapy with ADA produced antidrug antibodies with a significant higher frequency than patients on combined treatment with MTX (37.5% *vs* 8.7% of them respectively; p = 0.016). Patients on monotherapy with ADA also exhibit a significant reduced serum ADA levels than patients on ADA + MTX (4.73 μg/mL *vs* 12.73 μg/mL respectively; p = 0.019). However, no significant differences were observed in the ETN concentration between patients on monotherapy or on ETN + MTX treatment (1.50 μg/mL *vs* 1.77 μg/mL respectively). No correlation was observed between drug levels or the presence of antidrug antibodies and any of the clinical or biological parameters.

**Conclusion:** JIA paediatric patients needed intensified and combined with MTX antiTNF therapy more frequently than adults. In spite of this, no differences were found regarding antiTNF levels between children and adults. This fact suggest that children need more therapy to maintain antiTNF concentrations to control the disease activity. No antibodies antiETN were detected in our patients. ADA in monotherapy seems to be more immunogenic than in combined therapy in JIA. We found no correlation between antiTNF levels or the presence of anti-antiTNF antibodies and the clinical outcome of JIA patients.

**Disclosure of Interest**

None Declared

### P88 Fibrous arthropathy associated with morphea: a new cause of diffuse acquired joint contractures in children

#### Etienne Merlin^1^, Sylvain Breton^2^, Sylvie Fraitag^2^, Jean-Louis Stephan^3^, Carine Wouters^4^, Christine Bodemer^2^, Brigitte Bader-Meunier^2^

##### ^1^CHU Clermont-Ferrand, Clermont-Ferrand, France; ^2^APHP, Paris, France; ^3^CHU Saint-Etienne, Saint-Priest en Jarrez, France; ^4^University Hospital Leuven, Leuven, Belgium

###### **Presenting author:** Etienne Merlin

**Introduction:** Etiologies for childhood-onset diffuse joint contractures encompass a large group of inherited disorders and acquired diseases, especially a subset of juvenile idiopathic arthritis called “dry polyarthritis”, dermatomyositis and systemic sclerosis.

**Objectives:** First time description of the cases of patients who presented with diffuse acquired joint contractures preceding distant superficial circumscribed morphea

**Methods:** Clinical reports

**Results:** We report 2 boys aged 5 and 8 years who developed acquired symmetric painless joint contractures preceding from 7 to 13 months the development of superficial plaques of morphea.

There was no other clinical involvement, no biological inflammation and no auto-antibodies. Search for urinary mucopolyaccharidosis was negative. In both patients, MRI of the wrist showed no joint effusion, no bone erosion, absent or mild synovial thickening with a slight enhancement after gadolinium infusion. One patient underwent a synovial biopsy which showed a dense fibrosis with a sparse inflammatory infiltrates, similar to the pathological pattern observed in the skin biopsy. With methotrexate and systemic steroids, joint contractures slowly improved in the first patient, remained stable in the second.

**Conclusion:** These two cases suggest that fibrous synovitis should be considered in children with acquired diffuse, symmetric and painless contractures, without elevation of acute phase reactants even in absence of extra-articular manifestations.

Articular MRI with gadolinium and careful cutaneous examination at onset and during the follow-up should provide clues for diagnosing this entity.

**Disclosure of Interest**

None Declared

### P89 Subclinical atherosclerosis in children with Juvenile Idiopathic Arthritis

#### Francesco Baldo^1^, Federico Annoni^2^, Giancarla Di Landro^1^, Sofia Torreggiani^1^, Marta Torcoletti^1^, Antonella Petaccia^1^, Fabrizia Corona^1^, Giovanni Filocamo^1^

##### ^1^Dipartimento della Donna, del Bambino o del Neonato, Fondazione Irccs Cà Granda Ospedale Maggiore Policlinico, Milano, Italy; ^2^Dipartimento di Fisiopatologia Medico Chirurgica e dei Trapianti, Fondazione Irccs Cà Granda Ospedale Maggiore Policlinico and Università di Milano, Milano, Italy

###### **Presenting author:** Francesco Baldo

**Introduction:** Juvenile Idiopathic Arthritis (JIA) is associated with early subclinical signs of atherosclerosis. Chronic inflammation per se may be an important driver but other known risk factors, such as dyslipidemia, hypertension. In 2006, the American Heart Association published guidelines endorsed by the American Academy of Pediatrics with a list of diseases in children with an increased risk of cardiovascular diseases (CVD) where JIA was included.

Because CV events are rare in the young, surrogate markers of atherosclerosis such as carotid intima-media thickness (IMT) are valuable to detect early subclinical atherosclerosis.

**Objectives:** To assess subclinical atherosclerosis in patients with JIA and to compare it with disease duration, subtype of arthtritis and the principal outcome measures of disease activity

**Methods:** Patients with JIA consecutively hospitalised in our center were included in the study. IMT of the common carotid artery was determined by ultrasonography.

The serum levels of atherosclerosis-related biomarkers, such as homocistein, triglicerid, cholesterol and sistemic blood pressure were measured.

The Juvenile Arthritis Disease Activity Score (JADAS) was used to measure JIA disease activity.

**Results:** 10 patients with JIA were included in the study, 3 oligoarticular, 2 systemic, 3 polyarticular, 1 psoriatic and one with enthesitis related arthritis. Mean age 5.3 yrs, mean disease duration 2.7 yrs.

The median (range) of JADAS71 in the 10 patients was 10 (0-19). The table shows the Spearman correlations of IMT with the principal outcome measures.

**Conclusion:** The preliminary results do not evidenced a clear correlation between IMT and JADAS or other outcome measures. These results need to be confirmed in a larger cohort of patients.

**Disclosure of Interest**

None Declared

**Table 34 (abstract P89).** See text for description

	IMT
Age	0,518340
Age at onset	0,380243
Disease duration	0,303138
Physician’s global assessment	-0,066940
Active joint count	0,013623
ESR	-0,107416
Cholesterol level	0,589938
Weight	0,562500
Blood pressure	-0,217043

### P90 Treatment and 1-year outcomes of an inception cohort of Australian children with JIA

#### Georgina Tiller, Jo Buckle, Jane Munro, Angela Cox, Peter Gowdie, Roger C. Allen, Jonathan D. Akikusa

##### Rheumatology, Royal Childrens Hospital, Melbourne, Australia

###### **Presenting author:** Georgina Tiller

**Introduction:** Recent studies have provided insight into the short and intermediate term outcomes of cohorts of children with JIA managed with contemporary treatments in North America and Europe. There are no such data for Australasia.

**Objectives:** To describe the treatment and 1-year clinical outcomes of an inception cohort of newly diagnosed Australian children with juvenile idiopathic arthritis (JIA) followed at a tertiary paediatric centre.

**Methods:** Retrospective review of prospectively collected clinical data from a specifically designed electronic rheumatology database on all patients newly diagnosed with JIA at the Royal Children’s Hospital, Melbourne, between October 2010 and October 2014.

**Results:** 134 patients were included. Sixty one percent were female. The mean age at diagnosis was 8.3 years. The distribution of patients by subtype was: Oligoarticular 36%; RF + Polyarticular 2%; RF – Polyarticular 25%; Systemic 7%; Enthesitis Related 9%; Psoriatic 7% and Undifferentiated 13%. Eighty nine percent of patients achieved a zero joint count at least once in the first year of follow-up, however, just 66% had a zero joint count at 1 year. The mean time to a zero joint count was 4.3 months in patients achieving this outcome in the first year of follow-up. The systemic subtype had the shortest time to zero joint count at 2.7 months, psoriatic the longest at 6.2 months. For the 11% of patients who did not achieve a zero joint count in the first year of follow-up, the mean time to this outcome was 17.4 months.

Sixty three percent of patients were commenced on a conventional DMARD (cDMARD), most commonly methotrexate. The mean time to commencement of a cDMARD was 2.2 months. 15% were commenced on a biologic DMARD (bDMARD). The mean time to commencement of a bDMARD was 6.3 months. 55% were treated with oral corticosteroids. 19% were on oral corticosteroids at 1 year. 62% were treated with intra-articular steroids, most commonly the oligoarticular subtype, of whom 94% had at least one joint injection. 7% of children developed uveitis, with the highest incidence in the oligoarticular subtype, 17%.

Of patients with inactive arthritis at 12 months, 41% were on and 25% were off treatment.

**Conclusion:** This study demonstrates that children with JIA in Australia have demographic features and short-term outcomes similar to those described in international cohorts. At twelve months, two thirds of the cohort had a zero joint count and over a third of these were on no medications. When compared to international cohorts there was some variation in management, with a higher proportion having intra-articular steroid therapy.

**Disclosure of Interest**

None Declared

### P91 Anti-cyclic citrullinated peptide antibodies, antinuclear antibodies, and rheumatoid factor in Mexican patients with Juvenile Idiopathic Arthritis

#### Hayde G. Hernández-Huirache^1^, Edel R. Rodea-Montero^2^

##### ^1^Pediatric Rheumatology, Hospital Regional de Alta Especialidad del Bajío, Leon, Guanajuato, Mexico; ^2^Research, Hospital Regional de Alta Especialidad del Bajío, Leon, Guanajuato, Mexico

###### **Presenting author:** Hayde G. Hernández-Huirache

**Introduction:** Juvenile idiopathic arthritis (JIA) worldwide affects 1 in every 1,000 children. The ILAR classification describes seven categories of JIA. The diagnosis is clinical but there are biochemical markers associated with extra-articular conditions. The rheumatoid factor (RF) is presented in patients with rheumatoid arthritis (RA) but in pediatric patients their absence does not discard JIA, the presence of antinuclear antibodies (ANA) is associated with a high risk of uveitis, and the anti-cyclic citrullinated peptide antibodies (Anti-CCPA) are useful for the early diagnosis of RA and predicts erosive forms of the disease. In children with JIA there are few reports of the presence and useful of such antibodies.

**Objectives:** To determine the prevalence of each type of AIJ and the presence of Anti-CCPA, ANA, and RF in patients of the Pediatric Rheumatology Clinic of a third-level care Mexican Hospital (HRAEB), to compare clinical, immunological and inflammatory characteristics by type of JIA, and to quantify the correlation between assesments.

**Methods:** A cross-sectional sample of 50 patients with JIA (2-15 years). We estimate the prevalence of each type of JIA. The immunological characteristics (RF, ANA and Anti-CCPA) were assesed and compared by type of JIA (Kruskal Wallis or Chi-square). Spearman correlation coefficients between age and assessments were calculated.

**Results:** The analysis included 50 patients, 29 (58%) female and 21 (42%) male, aged 10.56 ± 3.39 years old, 34% seropositive polyarthritis, 28% seronegative polyarthritis, 16% systemic arthritis, 14% oligoarthritis, and 8% enthesitis related to arthritis. Association between sex and type of JIA (p = 0.014) was detected. Only one patient had positive ANA. There was significant difference in Anti-CCPA levels by JIA type (p < 0.001); higher leveles in patients with seropositive polyarticular JIA. High correlation between RF and Anti-CCPA (r = 0.63, p < 0.001) was detected. It was identified that age correlated with RF (r = 0.40, p = 0.004), and with Anti-CCPA (r = 0.29, p = 0.041).

**Conclusion:** Our data suggest that there is a positive correlation between RF and Anti-CCPA in patients with JIA. The elevation of Anti-CCPA and RF seem to be an early form of adult RA. Patients who have positive Anti-CCPA and RF have a higher degree of severity of the disease with a poor response to the conventional treatment and early biological therapy is required. The presence of Anti-CCPA and RF in a pediatric patient may be a criterion for making a decision on treatment. Multicenter studies that support these findings are required.

**Disclosure of Interest**

None Declared

**Table 35 (abstract P91).** Characteristics of the study population grouped by JIA type

Variables	Systemic arthritis (n = 8)	Enthesitis related to arthritis (n = 4)	Oligoarthritis (n = 7)	Seronegative polyarthritis (n = 14)	Seropositive polyarthritis (n = 17)	p-value
Female, n (%)	2 (25%)	0 (0%)	4 (57%)	10 (71%)	13 (76%)	p = 0.014^a,c^
Male, n (%)	6 (75%)	4 (100%)	3 (43%)	4 (29%)	4 (24%)	p = 0.014^a,c^
Age (years)	8.76 (4.86)	13.70 (1.91)	7.66 (3.49)	9.31 (4.21)	12.9 (1.98)	p = 0.007^b,c^
RF (UI/ml)	8.28 (0.92)	8.60 (0.00)	8.47 (1.09)	8.6 (0.00)	196.82 (300.65)	p < 0.001^b,c^
Anti-CCPA (U/ml)	9.41 (9.44)	11.95 (20.45)	6.29 (7.36)	9.11 (14.17)	64.94 (84.49)	p < 0.001^b,c^
<20 (negative) n (%)	6 (75%)	3 (75%)	6 (86%)	11 (79%)	3 (18%)	p < 0.001^a,c^
≥20 (positive) n (%)	2 (25%)	1 (25%)	1 (14%)	3 (21%)	14 (82%)	p < 0.001^a,c^

Unless otherwise indicated, the values are given as the mean (S.D.)

^a^Chi-square test

^b^Kruskal Wallis test

^c^Significant p-values

### P92 Development of the “REAJI” skill framework in Juvenile Idiopathic Arthritis

#### Jean-David Cohen^1^, Alexandre Belot^2^, William Fahy^3^, Pierre Quartier^4^, Christelle Sordet^5^, Sonia Trope^6^

##### ^1^Public Hospital, Montpellier, France; ^2^Public Hospital, Lyon, France; ^3^KOURIR patient organization, Paris, France; ^4^Public Hospital Necker, Paris, France; ^5^Public Hospital, Strasbourg, France; ^6^ANDAR patient organization, Paris, France

###### **Presenting author:** Jean-David Cohen

**Introduction:** In the list of healthcare treatments and interventions-JIA » of 2009, HAS recalls that therapeutic education must ensure involvement of the patient and his parents: understandability of the disease, mastering of the technical skills, and adaptation of the lifestyle. The main aim of the ETP of the child, the adolescent, and his relatives is to take care of and prevent complications and to learn the skills involved in care.

**Objectives:** In this context, we have decided to develop a framework of the necessary skills needed for children suffering from JIA (REAJI) in order to improve their quality of life (knowledge, know-how, know-how-to-be).

**Methods:** The steering committee is composed of health professionals involved in paediatric rheumatology and patients’ associations. Different stages are scheduled:

1. Literature review

2. Setup of four focus groups (FG) with different facilitation methods: 2 patient FGs (8-12 year olds and adolescents), 1 parent FG, 1 health professional (HP)

3. Analysis by the steering committee et production of a first skills list.

4. Submission of the list to a panel of patients and to the national JIA competence centres for further information.

5. Submission of the completed list to the same participants for ranking (primary and secondary objectives).

6. Validation of the final list by the committee.

**Results:** The steering committee is made up of two rheumatologists, two paediatricians, and two patients associations, ANDAR (association of patients suffering from RA) and KOURIR (association of parents whose children suffer from JIA).

The press review highlights a number of works, mainly on adolescents and outside the French territory. The HP FG is composed of 1 rehabilitation doctor, 1 rheumatologist, 1 paediatrician, 1 psychologist, 1 occupational therapist, 1 nurse and 1 pain nurse. The parent FG is composed of 10 people (6 mothers, 1 aunt, 3 fathers). The 8-12 year olds FG had 3 children and the adolescents FG 4 teenagers. The committee gathers to establish the initial skills list and then proposes to complete it via an online questionnaire to a selection of HPs from the 38 French centres listed by the FRancophone

SOciety of Rheumatology and Internal Paediatric Medicine (SOFREMIP) and an equal number of patients/ parents. This expanded list is sent back to participants using the same method in order to rank it and lastly to be validated in its final version by the committee.

**Conclusion:** The collectively-developed REAJI framework is a consensus-based support tool that anyone will be able to call upon: not only individual families but in particular teams working on educational projects. This multidisciplinary participative, national effort is an example of health democracy, the results of which will be made available to all.

**Disclosure of Interest**

None Declared

### P93 Measuring grip strength in children and adolescents diagnosed with juvenile idiopathic arthritis (JIA) using the new computerised Grippit™. Does poor grip strength and pain affect activities in daily life?

#### Karin B. Berggren, Johanna T. Kembe

##### Occupational Therapy Clinic, Children’s Occupational Therapy Ward, Stockholm, Sweden

###### **Presenting author:** Johanna T. Kembe

**Introduction:** Clinical experience is that inflammations and pain in hand/finger joints affects grip strength in children and adolescents diagnosed with JIA which also affects their activities in daily life. Occupational therapist’s at Astrid Lindgren’s Childrens’s hosptital, has measured grip strength with Grippit^TM^ electronic hand grip measure for the last 20 years. Grippit^TM^ has now been computerised. Results from Grippit^TM^ gives objective measurment in Newton presented in maximum, average and sustained grip strength after 10 seconds. The results are compared with norms for typical children age 4-16 years^1^. It is known that grip strength for children and adolescents with JIA is generally lower compared to healthy population^2^. With medical treatment, e.g. joint injections, and hand training programs grip strength and hand function will improve and can affect the performance of daily activities.

**Objectives:** To evaluate the computerised Grippit^TM^ and analyse data from children and adolescents with JIA and compare relation between grip strength, pain and activitites in daily life.

**Methods:** A clinical pilot study on 12 participants, 10 girls and 2 boys age 10-18 years. Grip strength was measured at 2-3 occasions per individual. Grip strength data was correlated to age and/or length of hand. Pain in the hand during the grip assessment was meassured with Visual Analogue Scale (VAS 0-100). Performance in activities in daily life (ADL) was measured with Childhood Health Assessment Questionnaire (CHAQ) for items concerning hand function.

**Results:** Eight participants had a lower grip strength compare to typical children. Pain in hand/fingers during the assessment was reported to mean 40 at VAS. Eleven participants reported a negative impact on ADL. Even for those (*n* = 4) with normal grip strength, the majority (*n* = 3) reported a negative performance on ADL and 2 of those reported pain in hand/fingers during the evaluation.

**Conclusion:** It is important to measure grip strength for children and adolescents with JIA to get a better understanding how the disease affects activity functioning and in order to conduct adequate interventions. The computerised Grippit^TM^ is as good and useful instrument for measuring grip strength.

References

1. Norms for grip strength in children aged 4–16 years, Häger-Ross 2002

2. Hand Grip Strength in Juvenile Idiopathic Arthritis as Predictor of Disease Activity and Disability in Clinical Practice, Hamman-Rashed 2015

**Disclosure of Interest**

None Declared

### P94 Internet program for physical activity and exercise-capacity in children with Juvenile Idiopathic Arthritis; a multicenter randomized controlled trial

#### Joyce Bos^1^, Wineke Armbrust^2^, Nico Wulffraat^3^, Marco van Brussel^4^, Jeanette Cappon^5^, Pieter Dijkstra^6^, Jan Geertzen^1^, Elizabeth Legger^2^, Marion van Rossum^5^, Pieter Sauer^7^, Otto Lelieveld^1^

##### ^1^Center for Rehabilitation, UMCG, Groningen, Netherlands; ^2^Department of Pediatric Rheumatology, Beatrix Children’s Hospital, UMCG, Groningen, Netherlands; ^3^Department of Pediatric Immunology, Wilhelmina Children’s Hospital, UMCU, Groningen, Netherlands; ^4^Child Development & Exercise Center, Wilhelmina Children’s Hospital, UMCU, Utrecht, Netherlands; ^5^Center for Rehabilitation and Rheumatology, Reade, Amsterdam, Netherlands; ^6^Center for Rehabilitation and Dept. of Oral and Maxillofacial Surgery, UMCG, Utrecht, Netherlands; ^7^Beatrix Children’s Hospital, UMCG, Groningen, Netherlands

###### **Presenting author:** Joyce Bos

**Introduction:** Physical activity (PA), health related quality of life (HRQoL) and exercise capacity are decreased in patients with Juvenile Idiopathic Arthritis (JIA) and do not restore after clinical remission has been obtained. Considering the impact of JIA on daily life, the long-term health risks of the disease, the sedentary habits, and the potentially favorable effect of PA, it is important to promote an active lifestyle in children with JIA. Therefore, we developed a 14-week, cognitive behavioral program, Rheumates@work, which was a combination of internet-based and in-person instructions supplemented with four group sessions to improve PA in children with JIA.

**Objectives:** The aim of this multicenter study is to determine the effects of Rheumates@work, on PA, exercise-capacity, HRQoL and participation in school and physical education class in children with JIA.

**Methods:** Patients, aged 8- 13 years, were recruited from three pediatric-rheumatologic-centers in the Netherlands to participate in an observer blinded, randomized controlled multicenter trial. Rheumates@work started either in winter or in summer. Primary outcome was PA, assessed with a diary and an accelerometer (Actical) during 7 days, expressed as PA level (PAL), time spent in rest, light PA and moderate-to-vigorous PA (MVPA), before the intervention, after the intervention (intervention and control group), and after 3 and 12 months follow-up (intervention group only). Secondary outcome parameters were; exercise-capacity (assessed with a Bruce treadmill test expressed as endurance time), HRQoL (with the Pediatric Quality of Life Inventory Generic-core-scale) and participation in school and sports (assessed by questionnaires).

**Results:** Twenty-eight children (median age 9.69, 21 girls) participated in the intervention and 21 as controls (median age 10.2, 12 girls). No significant differences between groups were found. None of the PA outcomes measured with the Actical had changed significantly within groups. A significant median difference of MVPA of 31 minutes (p = .04) has been found in the intervention group. Exercise-capacity improved in the intervention group with a median of 57 seconds (p = .02) and decreased in the control group by 46 seconds (not significant). Participation in school improved significantly in the intervention group. Children that missed at least one day of school due to JIA decreased from 43% to 14% (p = .02). Participation in physical education class increased in the intervention group from 57% to 71% (p = <.01). HRQoL improved in the control group (p = .01), not in the intervention group. Improvements on PA and exercise-capacity persisted in the 12 months follow-up period. Improvements in rest (median decrease of 54.17 minutes, p = .01), MVPA (median increase of 12.72 minutes, p = .03 (Actical)) and PAL (increase of 0.07, p = .02) (Actical)) were better for the intervention cohort starting in winter compared to the cohort starting in summer.

**Conclusion:** Rheumates@work exerted no significant differences between groups. Small positive effects on PA, exercise-capacity, and participation in school and physical education classes in the intervention group were found. Improvements lasted up until 12 months of follow-up. Participants who started in winter showed most improvements. Rheumates@work showed no effect on HRQoL.

**Trial registration identifying number:** ISRCTN92733069

**Disclosure of Interest**

None Declared

### P95 A rare disease in the elbow: Lipoma Arborescens

#### Kubra Ozturk^1^, Levent Buluc^2^, Gur Akansel^3^, Bahar Muezzinoglu^4^, Zelal Ekinci^1^

##### ^1^Department of Pediatrics Rheumatology, Kocaeli University Faculty of Medicine, Kocaeli, Turkey; ^2^Department of Orthopaedics and Traumatology, Kocaeli University Faculty of Medicine, Kocaeli, Turkey; ^3^Department of Radiology, Kocaeli University Faculty of Medicine, Kocaeli, Turkey; ^4^Department of Pathology, Kocaeli University Faculty of Medicine, Kocaeli, Turkey

###### **Presenting author:** Kubra Ozturk

**Introduction:** Lipoma Arborescens, is a diffuse villous proliferation of the synovium characterized by replacement of subsynovial tissue by mature adipocytes. The etiology is not exactly known. Usual presentation is on the middle-aged men and knee joint especially on the suprapatellar bursa. A few cases in childhood have been reported in the literature.

**Objectives:** In this report, we describe an unusual case of Lipoma Arborescens in a child who was initially diagnosed as oligo articular juvenile idiopathic arthritis (JIA) in the elbow without any response to JIA treatment.

**Methods: Case:** A 16-year-old boy was admitted to our hospital with the complaint of the swelling and limitation in the movement of the right elbow for the last 5 months. He had no history for trauma. Physical examination revealed swollen and limited range of motion of the right elbow without redness and warmth. Laboratory tests showed normal acute phase reactants; negative antinuclear antibody (ANA) and Purified Protein Derivative test. MR examination of the right elbow demonstrated joint effusion and synovial hypertrophy consistent with juvenile idiopathic arthritis. He was treated with ibuprofen and then intraarticular steroid injection was performed. Upon no response, methotrexate treatment was initiated. On the third month of the treatment, since no ACR paediatric 30 response was received, etanercept was added to treatment. On the sixth month of follow up another MR was performed based on his ongoing complaints and revealed dense effusion, synovial hypertrophy and contrast involvement consistent with active synovitis and also cystic destructive fields in his elbow.

**Results:** Having these findings, the patient was referred to the orthopedics department. A synovectomy was performed to the anterior and posterior compartments of elbow joint via antero-lateral approach. A sample of biopsy was also taken from the lytic lesion in the distal humerus. Histologically, synovium and perisynovium had increased adipose tissue with occasional lymphoid aggregates. Humerus biopsy revealed an osteoblastic tumor consistent with osteoblastoma having hemorrhagic cystic areas. The first MR was revaluated and T1-weighted sequences revealed frond-like synovial proliferation with high signal density where fat-supressive sequences revealed partial suppression in this formation. The diagnosis of Lipoama Arborescens was confirmed. The limitation range of motion was resolved in time. He is now on follow up for osteoblastoma.

**Conclusion:** This case has been assessed worthy to report because it was seen in the elbow joint together with osteoblastoma. This rare disease should be kept in mind where joint is severely swollen with limited range of motion, negative ANA and acute phase reactants and unresponsiveness to the oligo JIA treatment.

**Disclosure of Interest**

None Declared

### P96 Risk factors of bone mineral density decrease in children with juvenile arthritis

#### Ljubov Rychkova, Tatyana Knyazeva, Anna Pogodina, Tatyana Belova, Tamara Mandzyak, Ekaterina Kulesh

##### Scientific Center Of The Family Health And Human Reproduction Problems, Irkutsk, Russian Federation

###### **Presenting author:** Ljubov Rychkova

**Introduction:** Juvenile arthritis often associate with bone mineral density (BMD) decrease which can lead to osteoporosis onset and fractures. Determination of risk factors of BMD decrease will help to prevent these complications.

**Objectives:** To determine risk factors of BMD decrease in children with juvenile arthritis.

**Methods:** Bone mineral density (BMD) was investigated in 32 children from 6 to 17 years with juvenile chronic arthritis by means of dual-energy X-ray absorptiometry method according to program ‘The whole body’. The duration of the disease was from 2 to 14 years. Twenty three children had polyarthritis and 9 children had oligoarthritis. Using multiple regression analysis, the relationship between BMD and idiopathic arthritis (JIA) form, therapy, the age of disease onset, disease duration and the level of C-reactive protein (CRP) at the time of investigation, was estimated. All regressive models were adjusted for the age and child growth percentiles.

**Results:** The children age (p = 00006), child growth percentiles (p = 0,005) and the age of disease onset (p = 0,029) were determined to be the most associated factors to BMD. Disease duration, JIA form, the level of CRP at the moment of investigation, history of intra-articular injection of glucocorticosteroid (GC), treatment with biologic medicines and even orally intake of small doses of GC at the present have not shown significant associations with BMD.

**Conclusion:** Early onset of juvenile arthritis can be the risk factor of systematic decrease in BMD among children.

**Disclosure of Interest**

None Declared

### P97 Renal function in children with Juvenile Idiopathic Arthritis: a case-control study

#### Alessandro Cafarotti^1^, Manuela Marsili^1^, Cosimo Giannini^1^, Roberta Salvatore^1^, Giuseppe Lapergola^1^, Caterina Di Battista^1^, Maria Loredana Marcovecchio^1^, Raffaella Basilico^2^, Piernicola Pelliccia^1^, Francesco Chiarelli^1^, Luciana Breda^1^

##### ^1^Department of Pediatrics, University of Chieti, Chieti, Italy; ^2^Department of Radiology, University of Chieti, Chieti, Italy

###### **Presenting author:** Manuela Marsili

**Introduction:** Large population-based studies have shown the association between rheumatoid arthritis (RA) and chronic kidney disease [1]. In juvenile idiopathic arthritis (JIA) renal manifestations have been rarely observed [2]. Serum cystatin C (CysC) has been proposed as a novel biomarker of the renal function and microalbuminuria is regarded as the gold standard for diagnosing the onset of pediatric diabetic nephropathy. The resistive index (RI), a measure derived from an arterial Doppler sonographic waveform, provides information about arterial impedance; it is especially important for the evaluation of the blood flow in renal parenchymal diseases [3].

**Objectives:** To investigate the presence of renal damage in a group of children with JIA and to establish the possible relationship with treatment.

**Methods:** Blood urea nitrogen (BUN), serum creatinine, cystatin C (CysC), erythrocyte sedimentation rate (ESR), C-reactive protein (CRP), microalbuminuria, glomerular filtration rate (eGFR), and renal resistive index (RI) were assessed in 46 children with JIA (9 boys and 37 girls, mean age 10.3 ± 3.8) and compared with 51 healthy controls (M/F:26/25, mean age 11.5 ± 3.5 years). 20 patients were on methotrexate (MTX group) therapy (M/F = 2/18; mean age 9.1 ± 3.1 years) and 26 were treated with biologic drugs (BD group) (M/F = 7/19; mean age 11.2 ± 4.1 years). Renal RIs were measured from pulse Doppler waveforms obtained from interlobar and arcuate arteries in three different regions from upper, middle and lower third of the kidney. Also a mean RI was calculated for each child by averaging mean RI of the right and left kidney.

**Results:** Creatinine, CysC and BUN were significantly higher in patients compared to controls; besides eGFR was lower in JIA subjects respect to healthy patients (p < 0.001) (tab 36). In details, the right, left and mean RI values were significantly increased in JIA subjects compared to controls (p < 0.001). Of note BD group showed significantly higher values of the mean RI compared to MTX group (tab 36); however disease duration in the BD group tends to be higher than the MTX group.

**Conclusion:** The results of our study suggest that eGFR is reduced in children with JIA. Moreover RI values are impaired in subjects with JIA compared to healthy controls and are significantly higher in those treated with BD compared to MTX group. IR values are directly related to indexes of inflammation suggesting a direct effect of disease.

**References**

1) Chiu HY, Huang HL, Li CH, et al. Increased Risk of Chronic Kidney Disease in Rheumatoid Arthritis Associated with Cardiovascular Complications - A National Population-Based Cohort Study. PLoS One. 2015 Sep 25;10(9):e0136508.

2) Göksel AK, Sever L, Kasapçopur O, Calişkan S, Balci H, Arisoy N. Albuminuria and tubular markers in juvenile idiopathic arthritis. Pediatr Nephrol 2005; 20 (2):154–8.

3) Murat A^1^, Akarsu S, Ozdemir H, Yildirim H, Kalender O. Renal resistive index in healthy children. Eur J Radiol. 2005 Jan;53(1):67–71**.**

**Disclosure of Interest**

None Declared

**Table 36 (abstract P97).** Renal parameters of subjects with JIA

Parameters	Controls (n = 51)	JIA MTX group (n = 20)	JIA BD group (n = 26)	p-value*†‡§
Creatinine (mg/dl)	0.42 ± 0.10	0.48 ± 0.08	0.54 ± 0.15	<0.001^†‡^
Cystatin C (mg/l)	0.66 ± 0.05	0.79 ± 0.06	0.77 ± 0.13	<0.001^†‡^
BUN (mg/dl)	11.72 ± 1.32	12.00 ± 2.59	13.61 ± 3.18	0.002^‡^
Microalbuminuria (mg/dl)	1.09 ± 0.00	1.29 ± 0.60	1.54 ± 1.55	0.08
ESR (mm/h)	1.00 ± 0.00	9.15 ± 6.64	15.00 ± 8.68	<0.001^†‡§^
CRP (mg/dl)	0.20 ± 0.00	0.34 ± 0.31	0.78 ± 084	<0.001^‡§^
eGFR (ml/min/1.73 m^2^)	121.9 ± 19.6	98.9 ± 8.3	98.1 ± 10.7	<0.001^†‡^
RI	0.60 ± 0.03	0.65 ± 0.02	0.68 ± 0.03	<0.001†‡§
Duration of disease (years)	-	3.96 ± 2.72	6.59 ± 3.18	0.35

One Way Anova Mann-Whitney *U*-Test

^†^ = p < 0.05 Controls vs MTX group

^‡^ = p < 0.05 Controls vs BD group

^§^ = p < 0.05 MTX vs BD group

## Poster Session: Juvenile dermatomyositis I

### P98 Anti-synthetase autoantibody is seen in patients with overlap myositis in the UK cohort of patients with jveunile dermatomyositis

#### Beverley Almeida^1^, Sarah Tansley^2^, Stefania Simou^1^, Harsha Gunawardena^3^, Neil McHugh^4^, Lucy Wedderburn^1^, and Juvenile Dermatomyositis Research Group (JDRG)

##### ^1^Rheumatology, Institute of Child Health, University College London, London, UK; ^2^Rheumatology, Royal National Hospital For Rheumatic Diseases, Bath, UK; ^3^Clinical and Academic Rheumatology, North Bristol NHS Trust, Southmead Hospital, Bristol, UK; ^4^Department of Pharmacy and Pharmacology, University of Bath, Bath, United Kingdom

###### **Presenting author:** Beverley Almeida

**Introduction:** The anti-synthestase autoantibody is the most common in adult onset idiopathic inflammatory myopathies (IIM). The classic clinical features include myositis, interstitial lung disease (ILD), Raynaud’s, mechanic’s hands, Gottron’s lesions, non-erosive inflammatory arthritis and characteristic pyrexia.

In the pediatric population, anti-synthetase autoantibodies are rare and found in less than 5% of patients with Juvenile DM (JDM) but occur in 9% of patients with JPM and 13% of patients with juvenile overlap myositis, with the peak age at disease onset being 14 years^2^. They appear to have a similar disease phenotype to adults and are at risk of developing ILD, which has been found to be the most common cause of death and were consequently associated with an increased risk of mortality.

**Objectives:** The aim of this study was to define the frequency and investigate the clinical associations of anti-synthestase autoantibodies in a large cohort of patients with JDM, the UK JDM Cohort and Biomarker Study (JDCBS).

**Methods:** Serum samples and clinical data were obtained from 386 patients with JDM recruited to the UK Juvenile Dermatomyositis Cohort and Biomarker Study. The presence of anti-synthestase autoantibodies was determined by immunoprecipitation. Due to the small number of patients with the autoantibody in the study, descriptions of common features are given.

**Results:** Anti-synthestase autoantibodies were identified in only 6 (1.5%) patients with JDM within the cohort. All patients were female and all bar one were considered to have JDM overlap. 50% were of black origin, they were all non-anti-Jo1 positive and had ILD. The details of their diagnoses and clinical features are demonstrated in Table 37.

**Conclusion:** Anti-synthestase autoantibodies can be identified in a small proportion of patients with JDM and identify a distinctive clinical sub-group. Screening for anti-synthetase autoantibodies especially non-anti-Jo1 antibodies may identify patients at risk of ILD. This mirrors the adult presentation where ILD is the predominant manifestation of non-anti-Jo1 anti-synthestase autoantibodies.

**References**

1. Clin Rev Allergy Immunol. 2014 Dec;47(3):264–73. The evolving spectrum of polymyositis and dermatomyositis, moving towards clinicoserological syndromes: a critical review. Tansley S, Gunawardena H.

2. J Intern Med. 2016 Mar 30, epub ahead of print. The juvenile idiopathic inflammatory myopathies: pathogenesis, clinical and autoantibody phenotypes, and outcomes. Rider LG, Nistala K.

**Disclosure of Interest**

None Declared

**Table 37 (abstract P98).** Characteristics of those patients with anti-synthetase autoantibodies

Patient	1	2	3	4	5	6
Diagnostic label	Overlap with scleroderma and arthritis	Overlap with scleroderma and arthritis	Overlap with arthritis	Initially diagnosed with PAN, then myositis and SLE	JDM	Overlap with systemic sclerosis
Age at onset (years)	9.06	9.41	15.47	13.06	12.53	14.43
Length of follow up (years)	0.67	11.5	1.33	6.47	3.5	2.76
Specific antibody	Jo1	Jo1	Jo1	PL12	PL7	PL12
Muscle	Severe myositis	Myositis	Myositis	Myositis	Myositis	Destructive necrotizing, myopathy on biopsy
Skin	Gottron’s Heliotrope rash	Gottron’s Mechanics hands	Gottron’s Heliotrope rash	Heliotrope rash Erythematous rash on forearms	Gottron’s Heliotrope rash	Gottron’s Heliotrope rash Skin ulceration and calcinosis Mechanics hand
Joint	Polyarthritis with contractures	Nil of note	Arthritis	Nil of note	Nil of note	Nil of note
Lung	Normal	Normal	Normal	ILD	ILD	ILD
Mortality to date	Alive	Alive	Alive	Alive	Alive	Alive

### P99 Positive detection of Anti-NXP2 autoantibodies correlates with muscle ischemia in juvenile dermatomyositis

#### Jessie Aouizerate^1^, Brigitte Bader-Meunier^2^, Marie De Antonio^1^, Christine Bodemer^2^, Christine Barnerias^2^, Guillaume Bassez^1^, Isabelle Desguerre^2^, Pierre Quartier^2^, Romain Gherardi^2^, Jean-Luc Charuel^3^, François-Jérôme Authier^1^, Cyril Gitiaux^2^

##### ^1^Hôptal H.Mondor, Creteil, Paris, France, ^2^Hôpital Necker, Paris, France, ^3^Hôpital La Pitié, Paris, France

###### **Presenting author:** Brigitte Bader-Meunier

**Introduction:** Myositis-specific autoantibodies (MSA) are useful biomarkers to classify juvenile dermatomyositis (JDM). Anti-NXP2 antibodies are present in 20% of JDM and has been reported to be associated with a substantially increase of the risk of calcinosis (1). Histological features of anti-NXP2 positive JDM remain unknown

**Objectives:** Myositis-specific autoantibodies (MSA) are useful biomarkers to classify juvenile dermatomyositis (JDM). Anti-NXP2 antibodies are present in 20% of JDM and has been reported to be associated with a substantially increase of the risk of calcinosis (1).

**Methods:** A retrospective study of clinical, biological and histological findings from 10 anti-NXP2 positive JDM patients diagnosed from 2013 to 2015 in both Pediatric rheumatology and dermatology centers (Necker’s hospital). Systematic auto-antibodies screening (Mi2, MDA5, TIF1-gamma, NXP2, SAE, Ro52, Jo1, PL7, PL12, EJ, SRP, Ku, PM-Scl, Scl70,) and myopathological study of deltoid muscle biopsy (including immunohistochemistry for endothelial cells (CD31/PECAM), regenerating myofibers (CD56/NCAM), inflammatory cells, anti-human major histocompatibility complex (MHC) class I (HLA-ABC), class II (HLA-DR), and C5b-9 were performed. All biopsies were reviewed using the score tool in JDM (2). Comparisons between anti-NXP2-positive patients NXP2 (group 1) and anti-NXP2 negative patients (group 2) for initial clinico-pathological variables were performed using the Fisher’s exact test for qualitative variables and the Wilcoxon and Kruskal-Wallis tests for quantitative variables.

**Results:** Autoantibodies were detected in 16/25 JDM patients, including anti-NXP2 in 10/25, anti-MDA5 in 2/25 and anti-Tif1gamma in 4/25. In group 1, patients had a greater degree of weakness at diagnosis (median MMT score 42 *versus* 63, p =0,01; median CMAS score 3 *versus* 38, p =0,001) and more frequent vasculopathy-related features, especially gastrointestinal involvement, (p = 0,007) than in group 2. Conversely, the percentages of patients who developed calcinosis did not differ between the two groups (20% in each group). At diagnosis, CPK values were > 5 000 IU/L in 88% and 43% of patients from group 1 and 2 respectively. All the patients underwent a muscle biopsy. Histological features revealed typical lesions of dermatomyositis in all cases: inflammatory and perimysial myopathy with ischemic lesions, perifascicular atrophy and necrosis, expression of HLA ABC class I, C5b9 capillary and sarcolemal deposits. Group 1 displayed more frequent ischaemic punch-out vacuoles associated with microinfarcts (p = 0,022) and capillary dropout (p = 0,01). At 6 months, 80% of the group 1 patients required more than two lines of treatment (including plasmapheresis or immunoadsorption) *versus* 45% in group 2 (p = 0,01)

**Conclusion:** Anti-NXP2-positive patients demonstrate a greater disease severity and a more prominent ischemic pattern than other JDM. Histopathological investigation can contribute to the classification of inflammatory myopathies in assocation with the MSA screening.

**References**

1 Tansley SL, Betteridge ZE, Shaddick G et al. Calcinosis in juvenile dermatomyositis is influenced by both anti-NXP2 autoantibody status and age at disease onset. Rheumatology (Oxford). 2014;53:2204–8

2 Varsani H, Charman SC, Li CK et al. Validation of a score tool for measurement of histological severity in juvenile dermatomyositis and association with clinical severity of disease. Ann Rheum Dis. 2015; 74:204–10

**Disclosure of Interest**

None Declared

### P100 The role of muscle MRI in detecting a flare-up of juvenile dermatomyositis

#### C H. Spencer^1^, Rabheh Abdul Aziz^1^, Chack-Yung Yu^1^, Brent Adler^2^, Sharon Bout-Tabaku^1^, Katherine Lintner^3^, Melissa Moore-Clingenpeel^3^

##### ^1^Rheumatology, Nationwide Childrens/Ohio State, Columbus, United States; ^2^Radiology, Nationwide Childrens/Ohio State, Columbus, United States; ^3^Research Institute, Nationwide Childrens/Ohio State, Columbus, United States

###### **Presenting author:** C H. Spencer

**Introduction:** The course of juvenile dermatomyositis has improved greatly over the last 70 years with the aggressive use of corticosteroids, immunosuppressive medication, and biologics. Yet it is often difficult to detect disease flare in these children. Symptoms may be mild, signs of the rash and muscle disease can vary widely and are often equivocal. Lab tests including muscle enzymes are often normal. EMG and muscle biopsy are invasive and not reliable. New disease outcome measures are in development. Other tools may be helpful to determine if more treatment is needed.

**Objectives:** To determine if a muscle MRI is useful in detecting a JDM disease flare-up and affecting physician decision-making.

**Methods:** Approval was obtained from the IRB of Nationwide Children’s Hospital (NCH). Subjects were identified by US ICD10 and ICD9 codes and only included if they met the modified Bohan and Peter DM criteria and were seen at NCH between 1/2005 and 6/2015. Each child had to be in remission for 6 months prior to flare-up. Data collected included demographics, clinical presentation at onset, clinical features at time of repeat MRI, lab test and imaging results at presentation and flare-up, and treatment before and after flare-up MRI. The MRI was performed on both lower extremities without contrast with the following sequences: Axial T1, axial T2 fat saturation, axial and coronal inversion recovery, and axial diffusion weighted. The physician decision that a child with JDM was in a flare-up was the gold standard, using rash, muscle weakness (CMAS), muscle enzymes, and myositis on MRI. We compared the MRI result with the physician’s decision of relapse or not and evaluated whether there was a concordance or discordance between MRI findings and the subsequent treatment decision. Cohen’s kappa test was used for chance-corrected agreement between physician decision and MRI findings, Bayes’ rule for conditional probability of whether the rheumatologist would decide to treat for a JDM flare-up given the MRI findings, and McNemar’s test for comparison of MRI findings and muscle enzymes at flare-up.

**Results:** Forty-five JDM children were identified with 32 females; median age at diagnosis was 5.8 years. Eighty percent had weakness at diagnosis, 100% typical rash, 73% typical nail fold capillary changes. At diagnosis, muscle enzymes were compatible with JDM generally (CK 52%, LDH 62%, aldolase 72%, AST 54% abnormal), EMG abnormal in 3/8, muscle biopsy typical of JDM in 10/11, and MRI abnormal for myositis in 31/40. Thirteen of the 45 patients had a repeat MRI for possible flare-up with differing indications: general proximal weakness in 4, hip, thigh or calf pain in 3, rash in 3, worsening nail fold changes in 1, elevated enzymes in 1, and to confirm remission in 1. Three of 13 repeat MRI’s were abnormal, demonstrating myositis. There was moderate agreement between the flare-up MRI findings and the physician’s treatment decision (kappa = 0.59); in each abnormal MRI case the physician decided to increase treatment (Bayes rule-100% agreement). When the MRI was negative for myositis in 10 patients, 7/10 physicians chose to taper medication (70% agreement). There was no agreement between MRI findings and muscle enzymes results.

**Conclusion:** Using a physician’s overall decision as the gold standard, our study does suggest that performance of an MRI at a time of a JDM flare-up is useful, yielding a sensitivity of 50% for flare-up and a specificity of 100%. Using Bayes rule, when an MRI shows myositis, physicians tend to treat 100% of the time and when an MRI shows no myositis, physicians tapered medications 70% of the time.

**Disclosure of Interest**

None Declared

### P101 Juvenile myositis: what happens next?

#### Christina Boros^1,2^, Liza McCann^3^, Nicola Ambrose^4^, Mario Cortina-Borja^2^, Stefania Simou^2^, Clarissa Pilkington^5^, Lucy Wedderburn^2^, and Juvenile Dermatomyositis Cohort and Biomarker Study (JCDBS)

##### ^1^University of Adelaide, Adelaide, Australia; ^2^University College London Institute of Child Health, London, UK; ^3^Alder Hey Childrens Hospital, Liverpool, UK; ^4^University College Hospital London, UK; ^5^Great Ormond Street Hospital for Children, London, United Kingdom

**Introduction:** Few studies describe the natural course and long term outcomes of myositis in childhood in large, prospectively-followed patient cohorts, treated in the modern era.

**Objectives:** To describe long term patient-reported outcomes in adolescents and young adults (≥16 yrs) who had myositis in childhood

To find outcome predictors from data in the Juvenile Dermatomyositis Cohort and Biomarker Study, UK and Ireland (JDCBS)

**Methods:** Participants in the JDCBS, previously diagnosed with an idiopathic inflammatory myopathy and now aged 16 years or over, completed three questionnaires: the SF36 v2, HAQ and a newly developed questionnaire to collect information on current disease features and damage, medication use and side effects, education and employment opportunities and difficulties, effects of myositis on growth and development, living arrangements and smoking and alcohol intake.

**Results:** Of the 205 sets of questionnaires sent, 84 (41%) were returned. Average age of respondents was 21.5 years (maximum 30.8y). Average disease duration was 11.8 y (SD 5.1), age at onset 9.2y (SD 4.3) and female to male ratio 4.25:1. Most patients were white (82.1%) and had a physician-assigned diagnosis of Definite Juvenile Dermatomyositis (69%). Of the 68 who had Myositis-Specific Autoantibody testing at diagnosis, eight (11.8%) had TIF1γ and eight had NXP2 Ab. Six (8.8%) were positive for Mi2 antibodies. Nine (13.2%) had unknown bands and 14 (20.6%) were negative on autoantibody testing.

Of note, 49 (59%) reported persistently active disease (see Table 38 for details) and 54 (65%) were still taking immunosuppressive medication for myositis. Among respondents at school or higher education, 13 out of 29 (44.8%) reported that their academic results were adversely affected by myositis, and. that time missed due to myositis, muscle weakness and fatigue were all significant contributors. Around two-thirds of all respondents found that myositis had made it difficult to study. Fourteen out of 50 (28%) respondents reported career compromise caused by myositis; of these, 10/37 (27%) were employed and 4/13 (30.8%) were unemployed. There were 14/76 respondents (18.4%) aged 17-30 years enrolled in higher education. Among 47 patients aged 18 to 24 there were 21 (44.7%) who were employed; patients in this study were twice as likely to be unemployed compared to the corresponding age group in the UK population (*p* = 0.001, OR 0.456, 95% CI 0.24, 0.84).

SF36 Physical Composite Scores were significantly better in those who did not report current myositis (*p* = 0.0003) arthritis (*p* = 0.002) or muscle weakness (*p* = 0.0001). Mental Composite Scores were also better in those who did not report current arthritis (*p* = 0.03) or muscle weakness (*p* = 0.013).

**Conclusion:** We found high patient-reported rates of persistently active disease and medication use in long term follow-up of juvenile myositis. The young people had reduced rates of employment compared to the corresponding UK general population.

**Disclosure of Interest**

None Declared

**Table 38 (abstract P101).** See text for description

Category	Patient reported outcome	*n* (%) answering yes
Specific disease features	Muscle weakness	28 (32.5)
	Arthritis	28 (32.5)
	Skin rash	21 (25.3)
	Calcinosis	18 (21.9)
Education and Employment status	At school	17 (20.5)
	Enrolled in higher education	15 (18.1)
	Employed	38 (45.8)
	Unemployed	14 (16.9)

### P102 Diagnosis of Juvenile Dermatomyositis in Germany – a survey among paediatric rheumatologists and child neurologists

#### Claas Hinze^1^, Prasad T. Oommen^2^, Fabian Speth^3^, Johannes-Peter Haas^3^, and Working Group “Juvenile Dermatomyositis” of the German Society for Paediatric and Adolescent Rheumatology (GKJR)

##### ^1^Pediatric Rheumatology and Immunology, University Hospital Münster, Münster, Germany; ^2^Department of Pediatric Oncology, Hematology and Immunology, University Children’s Hospital, Medical Faculty, Heinrich-Heine-University, Düsseldorf, Germany; ^3^German Center for Paediatric Rheumatology and Immunology, Garmisch-Partenkirchen, Germany

###### **Presenting author:** Claas Hinze

**Introduction:** Juvenile Dermatomyositis (JDM) is an extremely rare pediatric rheumatic disease. The early detection of typical clinical findings and adequate use of diagnostic testing is important in order to allow early clinical diagnosis and treatment. Diagnostic criteria for DM were established in 1975 (Bohan/Peter-criteria) but additional diagnostic modalities are available nowadays.

**Objectives:** To determine the current use of diagnostic testing for JDM in Germany.

**Methods:** We designed an online survey addressing all members of the Society for Paediatric Rheumatology (GKJR) in Germany and child neurologists with a special interest in JDM in February/March of 2016. The survey consisted of 6 case scenarios (32 questions) addressing diagnostic testing, diagnostic criteria and the treatment of JDM (covered elsewhere). Answers were given on a multiple-choice basis or based on a 5-point Likert scale. Descriptive statistics were used to analyze the findings.

**Results:** The survey was completed by 59 members of the GKJR and 7 child neurologists experienced in the diagnosis and treatment of JDM. The most important characteristics for the diagnosis of JDM in clinical practice based on a 5-point Likert scale (1 = essential, 5 = not important at all) were considered to be: typical skin findings (1.30 weighted average), proximal muscle weakness (1.51), typical MRI findings (1.61), elevated muscle enzymes (1.67), typical nailfold capillary changes (2.06), the presence of calcinosis (2.10), typical findings on muscle biopsy (2.18), dysphagia/dysphonia (2.38) and constitutional symptoms (2.47). The following diagnostic tests are used by more than 80% of respondents in all patients with probable JDM (in order of descending likelihood): CK, GOT, GPT, erythrocyte sedimentation rate, LDH, ANA, complete blood count, CRP, BUN/creatinine, EKG, pulmonary function testing, echocardiography, muscle MRI and immunoglobulin levels. Manual muscle testing and muscle biopsy are more commonly used by child neurologists whereas the childhood myositis assessment scale (CMAS), serologic testing (immunoglobulin levels, antibodies to infectious agents, anti-ENA antibodies and myositis-specific antibodies) and abdominal ultrasound more commonly by pediatric rheumatologists.

**Conclusion:** There is consensus that the diagnosis of JDM is strongly supported by typical MRI findings, in addition to classic clinical findings, and typical MRI findings are therefore accepted as a Bohan/Peter-equivalent diagnostic criterion in Germany. There is consensus on additional diagnostic testing in patients with probable JDM with some differences between paediatric rheumatologists and neurologist.

**Disclosure of Interest**

None Declared

### P103 Treatment of Juvenile Dermatomyositis in Germany – a survey among pediatric rheumatologists and child neurologists

#### Claas Hinze^1^, Prasad T. Oommen^2^, Fabian Speth^3^, Johannes-Peter Haas^3^, and Working Group “Juvenile Dermatomyositis” of the German Society for Paediatric and Adolescent Rheumatology (GKJR)

##### ^1^Pediatric Rheumatology and Immunology, University Hospital Münster, Münster, Germany; ^2^Department of Pediatric Oncology, Hematology and Immunology, University Children’s Hospital, Medical Faculty, Heinrich-Heine-University, Düsseldorf, Germany; ^3^German Center for Paediatric and Adolescent Rheumatology, Garmisch-Partenkirchen, Germany

###### **Presenting author:** Claas Hinze

**Introduction:** Data on the optimal management of juvenile dermatomyositis (JDM) are mostly lacking. Currently treatment decisions are often based on expert opinion. In order to develop consensus treatment protocols (CTP) for JDM in Germany, an understanding of the current clinical practice is essential.

**Objectives:** To determine the current pattern of treatment of JDM in Germany.

**Methods:** We designed an online survey addressing all members of the Society for Pediatric Rheumatology (GKJR) in Germany and child neurologists with a special interest in JDM in February/March of 2016. The survey consisted of 6 scenarios (32 questions) addressing diagnosis of JDM (covered elsewhere), treatment of moderate, severe or refractory JDM, and management of calcinosis associated with JDM. Answers were given either on a multiple-choice basis or based on a 5-point Likert scale for some items. Descriptive statistics was used to analyze the findings.

**Results:** The survey was completed by 59 members of the GKJR and 7 child neurologists, all of them with substantial experience in the treatment of JDM (on average 1-10 patients treated individually). There was wide variability regarding the induction treatment of moderate (severe) JDM: 28.4% (41.9%) use at least a single 3-day course of IV methylprednisolone (IVMP) pulse therapy, 68.7% (72.6%) use intermittent IVMP pulse therapy. High-dose, medium-dose and low-dose oral steroids were used by 25.4% (38.7%), 29.9% (30.7%) and 26.9% (4.8%), respectively. The following steroid-sparing drugs are used: Methotrexate 76.6% (91.3%), hydroxychloroquine 20.9% (54.8%), azathioprine 9.0% (11.3%), mycophenolate mofetil (MMF) 4.5% (8.1%), cyclosporine 1.5% (6.5%), intravenous immunoglobulins (IVIG) 14.9% (61.3%), rituximab 0% (9.7%), cyclophosphamide 0% (4.8%). Regarding JDM refractory to treatment with steroids and methotrexate, the following treatment preferences exist based on a 5-point Likert scale (1 = strongly supported, 5 = strongly rejected): IVIG (1.47 weighted average), MMF (1.86), rituximab (2.00), cyclosporine (2.28) and intensified steroid therapy (2.35).

**Conclusion:** Treatment of JDM in Germany is highly variable both in the induction and the maintenance treatment phases. IVIG is commonly used, especially in the context of severe or refractory disease. The development of CTPs for the treatment of JDM is desirable in order to harmonize treatment and allow comparative effectiveness research in the future.

**Disclosure of Interest**

None Declared

### P104 Treatment with high-dose subcutaneous immune globulins facilitated by recombinant human hyaluronidase in patients with juvenile dermatomyositis who are intolerant to intravenous immune globulins

#### Fabian Speth^1^, Johannes-Peter Haas^1^, Claas Hinze^2^

##### ^1^German Center for Paediatric and Adolescent Rheumatology, Garmisch-Partenkirchen, Germany; ^2^Pediatric Rheumatology and Immunology, University Hospital Münster, Münster, Germany

###### **Presenting author:** Claas Hinze

**Introduction:** High-dose intravenous immune globulins (IVIg) are a promising strategy in refractory juvenile dermatomyositis (JDM) but IVIg administration may be cumbersome and is often poorly tolerated. Combining recombinant human hyaluronidase to facilitate the administration of high doses of subcutaneous IgG (fSCIg) may be an alternative to IVIg.

**Objectives:** To describe the first experiences using high-dose fSCIg in patients with refractory, severe JDM intolerant to IVIg.

**Methods:** In this retrospective case series, five patients with steroid-refractory severe JDM were treated with fSCIg 2 g/kg/month due to IVIg adverse effects (including severe headaches, nausea, vomiting following IVIg in all five patients, and difficult venous access in one patient). Peak serum IgG levels, muscle enzymes, the childhood myositis assessment scale (CMAS) and adverse effects were observed for at least 6 months.

**Results:** Patients received fSCIg (2 g/kg/month [n = 4] or 1.7 g/kg/month [n = 1]). The first fSCIg schedule we used (fSCIg 1 g/kg every 14 days, i.e. days 1 and 15 of every 28-day cycle) resulted in median IgG peak levels of 1901 mg/dl (1606-2719 mg/dl), compared to median IgG peak and trough levels while previously receiving IVIg of 2758 mg/dl (2429-3560 mg/dl) and 1419 mg/dl (1325-1922 mg/dl). On this regimen, two patients maintained clinically inactive disease, one patient had mildly increased disease activity, one patient had partial improvement and one patient had persistent minimal disease activity. An alternative, “compressed” fSCIg schedule was then used in the three patients with persistent disease activity (fSCIg 1 g/kg five days apart every month, i.e. days 1 and 6 of every 28-day cycle) resulting in IgG peak levels of 2757 mg/dl, 2846 mg/dl and 2300 (previously 1606, 1901 and 1774 mg/dl on the biweekly regimen), resulting in clinically inactive disease in two of the three patients. The administration of fSCIg was well tolerated and there were no serious adverse effects.

**Conclusion:** We report for the first time that in patients with JDM fSCIg allows dosing similar to IVIg. With a “compressed” fSCIg treatment schedule consisting of two applications five days apart, high peak serum IgG-levels are achieved, possibly important for treatment success.

**Disclosure of Interest**

None Declared

### P105 Comparison of the PRINTO 2010 and PRINTO/IMACS 2016 improvement criteria in the PRINTO trial in new onset juvenile dermatomyositis

#### Claudio Lavarello, Gabriella Giancane, Angela Pistorio, Lisa Rider, Rohit Aggarwal, Sheila K. Oliveira, Ruben Cuttica, Michel Fischbach, Gary Sterba, Karine Brochard, Frank Dressler, Patrizia Barone, Ruben Burgos-Vargas, Elizabeth Candell Chalom, Marine Desjonqueres, Graciela Espada, Anders Fasth, Stella Maris Garay, Rose-Marie Herbigneaux, Claire Hoyoux, Chantal Job Deslandre, Frederick W. Miller, Jiri Vencovsky, Angelo Ravelli, Alberto Martini, Nicolino Ruperto

##### Pediatria II, Reumatologia; PRINTO, Istituto Giannina Gaslini, GENOA, Italy

###### **Presenting author:** Claudio Lavarello

**Introduction:** Juvenile dermatomyositis (JDM) is a systemic autoimmune disease characterized by chronic skeletal muscle inflammation with weakness and skin involvement. PRINTO and IMACS developed criteria to evaluate response in clinical trials which have been recently updated and validated.

**Objectives:** To compare the PRINTO 2010 (JDM 2010 response criteria) with PRINTO/IMACS 2016 response criteria (JDM 2016 criteria) for improvement in the PRINTO new onset juvenile dermatomyositis (JDM) trial.

**Methods:** New onset JDM children were randomized to receive either prednisone (PDN) alone or in combination with methotrexate (MTX) or cyclosporine A (CSA). Patients were evaluated at months 6, 12, 18 and 24 with different levels of JDM 2010 criteria (improvement of 20-50-70 or 90% in at least three of the 6 core set measures and worsening <30% in no more than one). The same patients were re-analyzed with the continuous JDM 2016 criteria which evaluate absolute percent change into three categories (total score 0-100): minimal (≥30), moderate (≥45), major (≥70) improvement. Clinical trials data were analyzed according to the intention-to-treat (ITT) principle with patients discontinuing (lack of efficacy, safety, etc.) considered as non-responders from that point onward.

**Results:** 139 children were enrolled in the trial: 47 on PDN, 46 on PDN + CSA and 46 on PDN + MTX. The two sets of criteria showed a similar capacity to recognize different levels of improvement of the disease at 6, 12, 18 and 24 months, after treatment with PDN, PDN + CSA or PDN + MTX as shown by the overlapping 95% CI intervals at various time points (Table). In particular, at month 6, 51% patients on PDN versus 72% on PDN + CSA or PDN + MTX achieved at least JDM 2010 20% improvement (p = 0.023) as compared to 55% and 74% with the JDM 2016 minimal improvement (p = 0.027). At month 24, 38% patients on PDN versus 60% on PDN + CSA or PDN + MTX achieved at least JDM 2010 20% improvement (p = 0.016) as opposed to 38% and 61% with JDM 2016 minimal improvement (p = 0.012). Kappa agreement between the 2 criteria with ITT approach were at least in the moderate range (0.61-0.8) as follows: 0.75, 0.75, 0.77, 0.79 at 6-12-18 and 24 months, respectively. The JDM 2016 criteria, similar to the 2010 criteria, confirmed the superior efficacy of combined treatment with PDN + CSA or PDN + MTX versus treatment with PDN alone.

**Conclusion:** Both response criteria have shown a similar discrimination in evaluating different levels of improvement in new onset JDM patients treated with 3 alternative treatment strategies.

**Trial registration identifying number:** ClinicalTrials.gov identifier: NCT00323960

EudraCT number: 2005-003956-37

**Disclosure of Interest**

C. Lavarello: None Declared, G. Giancane: None Declared, A. Pistorio: None Declared, L. Rider: None Declared, R. Aggarwal: None Declared, S. K. Oliveira: None Declared, R. Cuttica: None Declared, M. Fischbach: None Declared, G. Sterba: None Declared, K. Brochard: None Declared, F. Dressler: None Declared, P. Barone: None Declared, R. Burgos-Vargas: None Declared, E. Candell Chalom: None Declared, M. Desjonqueres: None Declared, G. Espada: None Declared, A. Fasth: None Declared, S. M. Garay: None Declared, R.-M. Herbigneaux: None Declared, C. Hoyoux: None Declared, C. Job Deslandre: None Declared, F. Miller: None Declared, J. Vencovsky: None Declared, A. Ravelli: None Declared, A. Martini Grant / Research Support from: The G. Gaslini Hospital, which is the public Hospital where I work as full time public employee, has received contributions from the following industries for the coordination activity of the PRINTO network: BMS, GlaxoSmithKline (GSK), Hoffman-La Roche, Novartis, Pfizer, Sanofi Aventis, Schwarz Biosciences, Abbott, Francesco Angelini S.P.A., Sobi, Merck Serono. This money has been reinvested for the research activities of the hospital in fully independent manners besides any commitment with third parties., Speaker Bureau of: I received speaker’s bureaus and consulting fees from the following pharmaceutical companies: Abbvie, Boehringer, Celgene, CrescendoBio, Janssen, Meddimune, Novartis, NovoNordisk, Pfizer, Sanofi Aventis, Vertex, Servier., N. Ruperto Grant / Research Support from: The G. Gaslini Hospital, which is the public Hospital where I work as full time public employee, has received contributions from the following industries for the coordination activity of the PRINTO network: BMS, GlaxoSmithKline (GSK), Hoffman-La Roche, Novartis, Pfizer, Sanofi Aventis, Schwarz Biosciences, Abbott, Francesco Angelini S.P.A., Sobi, Merck Serono. This money has been reinvested for the research activities of the hospital in fully independent manners besides any commitment with third parties., Speaker Bureau of: I received speaker’s bureaus and consulting fees from the following pharmaceutical companies: AbbVie, Amgen, Biogenidec, Alter, AstraZeneca, Baxalta Biosimilars, Biogenidec, Boehringer, BMS, Celgene, CrescendoBio, EMD Serono, Hoffman-La Roche, Italfarmaco, Janssen, MedImmune, Medac, Novartis, Novo Nordisk, Pfizer, Sanofi Aventis, Servier, Takeda, UCB Biosciences GmbH.

**Table 39 (abstract P105).** 2010 and 2016 improvement criteria after 6, 18 and 24 months of treatment with PDN or PDN + MTX or PDN + CSA (only few examples are reported)

2010 or 2016 criteria of improvement	PDN	PDN + CSA	PDN + MTX	P values
	N = 47	N = 46	N = 46	
	N (%) [95%CI]	N (%) [95%CI]	N (%) [95%CI]	
Month 6				
2010 ≥ 20%	24 (51%) [36–66]	32 (70%) [54–82]	33 (72%) [56–84]	0.023
2016 at least minimal	26 (55%) [40–70]	34 (74%) [59–86]	34 (74%) [59–86]	0.027
Month 18				
2010 ≥ 50%	20 (42%) [28–58]	30 (65%) [50–79]	29 (63%) [47–77]	0.015
2016 at least moderate	21 (45%) [30–60]	30 (65%) [50–79]	30 (65%) [50–79]	0.020
Month 24				
2010 ≥ 70%	18 (38%) [24–54]	26 (56%) [41–71]	26 (56%) [41–71]	0.042
2016 at least major	17 (36%) [23–51]	27 (59%) [43–73]	27 (59%) [43–73]	0.012

### P106 Inflammatory milieu of muscle biopsies and clinical features in juvenile dermatomyositis

#### Erdal Sag^1^, Seza Ozen^2^, Gulsev Kale^1^, Haluk Topaloglu^3^, Beril Talim^1^

##### ^1^Department of Pediatrics, Hacettepe University, Faculty of Medicine, Ankara, Turkey; ^2^Division of Pediatric Rheumatology, Hacettepe University, Ankara, Turkey; ^3^Division of Pediatric Neurology, Hacettepe University, Faculty of Medicine, Department of Pediatrics, Ankara, Turkey

###### **Presenting author:** Erdal Sag

This abstract is not included here as it has already been published.

### P107 Evidence based criteria for corticosteroid tapering/discontinuation. An analysis of the PRINTO trial in new onset juvenile dermatomyositis

#### Gabriella Giancane, Claudio Lavarello, Angela Pistorio, Francesco Zulian, Bo Magnusson, Tadej Avcin, Fabrizia Corona, Valeria Gerloni, Serena Pastore, Roberto Marini, Silvana Martino, Anne Pagnier, Michel Rodiere, Christine Soler, Valda Stanevicha, Rebecca Ten Cate, Yosef Uziel, Jelena Vojinovic, Angelo Ravelli, Alberto Martini, Nicolino Ruperto

##### Pediatria II, Reumatologia; PRINTO, Istituto Giannina Gaslini, Genoa, Italy

###### **Presenting author:** Gabriella Giancane

**Introduction:** Corticosteroids in juvenile dermatomyositis (JDM) alone or in association with other immunosuppressive drugs, namely methotrexate (MTX) and cyclosporine (CSA), represent the first-line treatment option for new onset JDM. No clear evidence based guidelines are actually available to standardize the tapering and discontinuation of corticosteroids in JDM.

**Objectives:** To provide an evidence-based approach for corticosteroid tapering/discontinuation through the analysis of the patients in the PRINTO new onset JDM trial.

**Methods:** New onset JDM children were randomized to receive either prednisone (PDN) alone or in combination with MTX or CSA. All children were given initially three daily pulses of intravenous methylprednisolone (30 mg/kg/pulse), and then PDN 2 mg/kg/day. After 1 month PDN was tapered to 1 mg/kg/day, from month 2 to 6 was tapered to 0.2, at month 12 to 0.1, and then discontinued at month 24. Major therapeutic changes were defined as the addition or major increase in the dose of MTX/CSA/other drugs or any other reasons for which the patient was dropped from the trial (adverse events, lost to follow-up, etc). Patients who followed the steroid tapering protocol and discontinued PDN at month 24 with no major therapeutic change (group 1) represented the reference standard for the best clinical outcome. Group 1 was compared with those following the steroid protocol, but with other major therapeutic changes (group 2), and with the group who deviated from the steroid protocol with/without major therapeutic changes (group 3). JDM core set measures (CSM) were compared in the 3 groups at 6-12-18 and 24 months (Table).

**Results:** 139 children were enrolled in the trial: 47 on PDN, 46 on PDN + CSA and 46 on PDN + MTX. We identified 57 (41%) patients for group 1, 24 (17%) for group 2 and 58 (42%) for group 3. At baseline all 3 groups had a high level of disease activity with no differences in the CSM. In group 1 (PND off no failure) there were 12 (21%) patients randomized to PDN alone, 21 (37%) to PDN + CSA and 24 (42%) to PDN + MTX. When we compared the three groups, significant differences were found in all CSM at each time point of analysis (p < 0.0001). In particular Group 1, when compared to Group 2 and 3, had the lowest level of disease activity at all time points; the decrease of disease activity was primarily within the first 6 months of treatment. Group 2 and 3 were overlapping in the levels of disease activity reached at all the time points and were globally higher when compared to group 1.

**Conclusion:** The PRINTO protocol from the trial in new onset JDM might constitute the reference evidence-based approach for corticosteroid tapering for possible use in current clinical practice.

**Trial registration identifying number:** ClinicalTrials.gov identifier: NCT00323960

EudraCT number: 2005-003956-37

**Disclosure of Interest**

G. Giancane: None Declared, C. Lavarello: None Declared, A. Pistorio: None Declared, F. Zulian: None Declared, B. Magnusson: None Declared, T. Avcin: None Declared, F. Corona: None Declared, V. Gerloni: None Declared, S. Pastore: None Declared, R. Marini: None Declared, S. Martino: None Declared, A. Pagnier: None Declared, M. Rodiere: None Declared, C. Soler: None Declared, V. Stanevicha: None Declared, R. Ten Cate: None Declared, Y. Uziel: None Declared, J. Vojinovic: None Declared, A. Ravelli: None Declared, A. Martini Grant / Research Support from: The G. Gaslini Hospital, which is the public Hospital where I work as full time public employee, has received contributions from the following industries for the coordination activity of the PRINTO network: BMS, GlaxoSmithKline (GSK), Hoffman-La Roche, Novartis, Pfizer, Sanofi Aventis, Schwarz Biosciences, Abbott, Francesco Angelini S.P.A., Sobi, Merck Serono. This money has been reinvested for the research activities of the hospital in fully independent manners besides any commitment with third parties., Speaker Bureau of: I received speaker’s bureaus and consulting fees from the following pharmaceutical companies: Abbvie, Boehringer, Celgene, CrescendoBio, Janssen, Meddimune, Novartis, NovoNordisk, Pfizer, Sanofi Aventis, Vertex, Servier., N. Ruperto Grant / Research Support from: The G. Gaslini Hospital, which is the public Hospital where I work as full time public employee, has received contributions from the following industries for the coordination activity of the PRINTO network: BMS, GlaxoSmithKline (GSK), Hoffman-La Roche, Novartis, Pfizer, Sanofi Aventis, Schwarz Biosciences, Abbott, Francesco Angelini S.P.A., Sobi, Merck Serono. This money has been reinvested for the research activities of the hospital in fully independent manners besides any commitment with third parties., Speaker Bureau of: I received speaker’s bureaus and consulting fees from the following pharmaceutical companies: AbbVie, Amgen, Biogenidec, Alter, AstraZeneca, Baxalta Biosimilars, Biogenidec, Boehringer, BMS, Celgene, CrescendoBio, EMD Serono, Hoffman-La Roche, Italfarmaco, Janssen, MedImmune, Medac, Novartis, Novo Nordisk, Pfizer, Sanofi Aventis, Servier, Takeda, UCB Biosciences GmbH

**Table 40 (abstract P107).** Core set measures values at different time points in the 3 groups of patients (only key results are reported)

	OFF PDN	OFF PDN	PDN ON	P value
	Failure: No	Failure: Yes	Failure Yes or No	
	Group 1	Group 2	Group 3	
	N = 57	N = 24	N = 58	
Month 6: MD global	0.5 (0–2)	2.6 (0.8–5)	3 (0.6–6)	<0.0001
Parent global	1 (0–1.8)	3.1 (0.5–5)	2.5 (0.8–5.8)	0.0002
CHAQ	0.1 (0–0.5)	0.1 (0–0.9)	0.5 (0–1.9)	0.0007
DAS	3 (0–5)	6.5 (1–11.5)	8 (4–12)	<0.0001
CMAS	47 (42–51)	45 (35–48.5)	35 (21.5–47)	0.0001
CHQ-PhS	51.8 (46.2–54.9)	47.5 (20.3–54.1)	36.3 (16.6–50.6)	<0.0001
MMT	77 (70–80)	70 (59–78.5)	64 (51–75)	<0.0001
Month 12: MD global	0 (0–1)	2.6 (1–5.5)	3 (0.6–6)	<0.0001
Month 18 DAS	0 (0–3)	6 (2.5–11.5)	9 (2–12)	<0.0001
Month 24: CMAS	50 (48–52)	42 (36–50)	36.5 (21.5–48)	<0.0001

### P108 Juvenile dermatomyositis, predictors of disease progression in Mexican children

#### Ana V. Villarreal, Nydia Acevedo, Talia Diaz, Yuridiana Ramirez, Enrique Faugier, Rocio Maldonado

##### Pediatrics Rheumatology, Hospital Infantil de Mexico Federico Gomez, Ciudad de Mexico, Mexico

###### **Presenting author:** Ana V. Villarreal

**Introduction:** Juvenile dermatomyositis is the most common inflammatory myopathy in children.

**Objectives:** To describe JDM clinical characteristics and course of the disease in a cohort of 40 Mexican patients diagnosed with juvenile dermatomyositis.

**Methods:** A transversal comparative study in a cohort of 40 patients with juvenile dermatomyositis over a period of 24 months. Descriptive data, parametric tests, and Kaplan - Meier graphs were used to analyse disease progression.

**Results:** The mean age at diagnosis was 8.1 years (SD = 3.7), most of them were female (57%), all patients from Hispanic ethnicity, 14 (35%) presented calcinosis at the time of JDM diagnosis, and up to 45% develop calcinosis at 6 months of disease onset. 5% found with an amiopatic patron. The median of the muscular enzymes values at the diagnosis was 1,005 mg/dL (15 - 29,130) for creatinine phosphokinase, 374 mg/dL (22-3,260) lactic dehydrogenase, aspartate aminotransferase 80 UI/L (17-1258). We also found initial dysphagia or dysphonia in 32.5%, pulmonary affection in 17.5%, and cardiac affection in 5%. Barium swallow examination was found altered in 25% at diagnosis. In terms of medication 24 patients received intravenous methylprednisolone initially, the median rate of pulse steroid usage was 6 ± 3.1, and 6 patients needs Biological therapy most of them anti-CD20. No relation between calcinosis at diagnosis and weakness of evolution before diagnosis in Kapplan Meier was found.

**Conclusion:** We found that Mexican pediatric population with JDM does not present the same course related to the presence of calcinosis, not according studies of other population.

References

1. Ravelli et al . **Long-Term Outcome and Prognostic Factors in Juvenile Dermatomyositis: A Multinational Multicenter Study of 490 patient.** Arthritis Care & Research . 2010; 62:63–72.

2. Sanner y cols. **Long-Term Muscular Outcome and Predisposing and Prognostic Factors in Juvenile Dermatomyositis: A case-Control Study** Arthritis Care & Research . 2010; 62: 1103–1111.

**Disclosure of Interest**: None Declared

### P109 Corticosteroid-associated pneumatosis intestinalis in juvenile dermatomyositis: a case report

#### Bita Arabshahi^1^, John H. Lee^2^, Ian Leibowitz^3^

##### ^1^Pediatric Rheumatology, Pediatric Specialists of Virginia, Fairfax, United States; ^2^Radiology, Fairfax Radiological Consultants PC, Fairfax, United States; ^3^Pediatric Gastroenterology, Pediatric Specialists of Virginia, Fairfax, United States

###### **Presenting author:** Bita Arabshahi

**Introduction:** Reports of pneumatosis intestinalis (PI) in Juvenile Dermatomyositis (JDM) are limited and often attribute the process to intestinal ischemia secondary to vasculitis. In patients with asymptomatic PI, however, the role for intestinal ischemia is not clear.

**Objectives:** We aimed to examine other potential causes of persistent PI in JDM, notwithstanding clinical resolution of vasculitis. Our goal was to examine the effect of immunosuppressive medications, namely corticosteroids, in pathogenesis of PI.

**Methods:** Hereby we report the case of a three year old Asian girl with anti-MDA-5 positive JDM and severe cutaneous vasculitis, who demonstrated persistent asymptomatic PI despite resolution of cutaneous lesions on cyclophosphamide and corticosteroids.

**Results:** Abdominal films taken during and after her six month course of cyclophosphamide did not reveal any improvment in PI. Patient showed gradual resolution of PI a year after completion of cyclophosphamide therapy, upon weaning her corticosteroids. Although the presence of anti MDA-5 antibodies is correlated with a higher risk of vasculitis, in our patient, there was no observed corrleation between the activity of cutaneous vasculitis and PI.

**Conclusion:** This observation suggests that PI in JDM may be multifactorial, and the role of corticosteroids should be considered in the differential diagnosis of PI in this population.

**Disclosure of Interest**

None Declared

### P110 Demographic and clinical characteristics of children with juvenile dermatomyositis in Cape Town

#### Lawrence O. Okong’o^1^, Jo Wilmshurst^2^, Monika Esser^3^, Christiaan Scott^4^

##### ^1^Paediatric Rheumatology, University of Nairobi, Nairobi, Kenya; ^2^Paediatric Neurology, University of Cape Town, Cape Town, South Aftrica; ^3^Paediatric Immunology and Rheumatology, University of Stellenbosch, Cape Town, South Africa; ^4^Paediatric Rheumatology, University of Cape Town, Cape Town, South Africa

###### **Presenting author:** Christiaan Scott

**Introduction:** Juvenile dermatomyositis (JDM) is a rare idiopathic inflammatory myopathy of childhood with an incidence of 1.9-3.2 per million. The aetiology of JDM is uncertain but may result from immune dysregulation triggered by environmental factors in genetically susceptible children. The demographic and clinical characteristics of JDM may thus differ by race and geographic regions. Few studies have described the characteristics of JDM patients from Africa. There is need for further studies for better understanding of the epidemiology, clinical characteristics and outcome of patients with JDM from the continent.

**Objectives:** To determine clinical characteristics and outcomes of patients with probable JDM seen between 2004-2013 in Cape Town, South Africa

**Methods:** We conducted a retrospective observational study to determine clinical characteristics and outcomes of patients satisfying the Bohan and Peter criteria for probable JDM seen between 2004-2013 in Red Cross, Groote Schuur and Tygerberg hospitals in Cape Town. Data was analyzed using R version 3.1.0 (2014-04-10).

**Results:** Twenty five cases were identified: 16 female and 9 male. Thirteen (52%) of the cases were of indigenous African, eleven (44%) mixed and one (4%) European ancestry. The median ages at disease onset and diagnosis were 6.75 (range 2.0-9.7) and 7.9 (range 3.4-9.75) years respectively. Muscle weakness and characteristic cutaneous manifestations occurred in all the 25 patients while 24 had elevated muscle enzymes. All the patients received corticosteroids, seventeen (73.9%) received methotrexate and four received rituximab. Eleven patients had calcinosis during the disease course [median follow up period of 50 (range 0.5-159) months]. The mortality was 2/25 (8%) while only 40% of the patients had clinically inactive disease by PRINTO criteria. There was no difference in racial distribution (p-value = 1), age at disease onset (p-value = 0.87) and disease duration prior to treatment initiation (p-value = 0.75) between patients who had clinically active and inactive disease. The demographic characteristics of children with JDM were similar to that from most other regions of the world with female predominance and similar age at onset. The median delay in diagnosis (4 months) was not longer than that reported in most other studies. However, some children had prolonged delay of up to 7 years due to misdiagnosis that denied them appropriate treatment in a timely manner. Majority (60%) of the patients also remained with clinically active disease, which put them at risk of further disease complications including calcinosis. Even though the mortality rate was low (8%) this was still more than double that reported in most recent large studies especially from the resource rich countries.

**Conclusion:** Long term follow up of JDM patients is advisable since majority of patients seem to have clinically active disease many years after disease onset despite treatment. Formulation and use of appropriate treatment guidelines and protocols may aid in the early diagnosis and appropriate management for optimum outcomes.

**Disclosure of Interest**

None Declared

### P111 Pulmonary involvement in children with rheumatic diseases

#### Ezgi Deniz Batu^1^, Nagehan Emiroglu^2^, Hafize Emine Sonmez^1^, Gokcen Dilsa Tugcu^2^, Zehra Serap Arici^1^, Ebru Yalcin^2^, Deniz Dogru^2^, Ugur Ozcelik^2^, Yelda Bilginer^1^, Mithat Haliloglu^3^, Nural Kiper^2^, Seza Ozen^1^

##### ^1^Department of Pediatrics, Division of Rheumatology, Hacettepe University Faculty of Medicine, Ankara, Turkey; ^2^Department of Pediatrics, Division of Pulmonology, Hacettepe University Faculty of Medicine, Ankara, Turkey; ^3^Department of Radiology, Hacettepe University Faculty of Medicine, Ankara, Turkey

###### **Presenting author:** Ezgi Deniz Batu

**Introduction:** The data on prevalence and type of pulmonary involvement in children with rheumatic diseases is scarce. Lung injury may be caused by the disease itself or by the biologic/nonbiologic disease modifying anti-rheumatic drugs (DMARDs).

**Objectives:** Our aim was to assess pulmonary functional status in children with connective tissue diseases (CTDs) and second, to assess the effect of DMARDs (biologic or nonbiologic) in children with rheumatic diseases.

**Methods:** Children with a diagnosis of CTD or receiving methotrexate or biologic drugs for more than two years who were followed-up at Hacettepe University Department of Pediatric Rheumatology and referred to the Department of Pediatric Pulmonology were included into this study. Medical history, physical examination, chest radiography (high resolution computerized tomography [HRCT] in selected cases), and pulmonary function tests such as spirometry, whole-body plethysmography and carbon monoxide (CO) diffusion test were evaluated in all patients.

**Results:** There were 33 children with CTDs (21 with juvenile dermatomyositis [JDM], 2 polymyositis [PM], 5 systemic lupus erythematosus, 4 systemic sclerosis, 1 mixed CTD). Female-to-male ratio was 2/1 and the median (min-max) age at diagnosis was 96 (11-186) months. CO diffusion capacity was low (<80%) in two patients (one with PM and one with JDM) and spirometry showed restrictive changes in these patients. HRCT was normal in the patient with PM. The patient with JDM had features suggesting interstitial lung disease (ILD) in HRCT and she was positive for anti-MDA5 autoantibody.

As for the second part of the study, there were 50 children who received biologic/nonbiologic DMARDs (44 methotrexate, 12 etanercept, 4 adalimumab, 4 anakinra, 1 tocilizumab, 1 canakinumab, 2 abatacept, 1 infliximab) for more than two years. The median (min-max) duration of treatment was 36 (24-180) months. During the routine evaluation for tuberculosis in 25 patients receiving biologics, 12 (48%) received isoniazid prophylaxis for nine months since their PPDs were ≥10 mm. However, no active tuberculosis was diagnosed during a follow-up of 36 (24-71) months. Only three patients had ILD who were using anakinra for ≥2 years for the treatment of systemic juvenile idiopathic arthritis. Both the disease itself and anakinra treatment might be contributing to the etiology of ILD in these children.

**Conclusion:** Pulmonary disease should be assessed carefully in children with CTD especially JDM/PM. ILD has been associated with several rheumatic diseases in children and whether this is a complication of the biologic/nonbiologic DMARD treatment. Thus, the routine follow-up of pulmonary functional status in children treated for rheumatic diseases is crucial to diagnose and treat pulmonary disease in the early phases.

**Disclosure of Interest**

None Declared

### P112 The clinical features of the juvenile dermatomyositis with anti-tif1 antibodies in Japan: report of three cases and review of the literature

#### Masato Yashiro, Mutsuko Yamada, Toshihiko Yabuuchi, Tomonobu Kikkawa, Nobuyuki Nosaka, Yosuke Fujii, Yukie Saito, Hirokazu Tsukahara

##### Pediatrics, Okayama University Hospital, Okayama, Japan

###### **Presenting author:** Masato Yashiro

**Introduction:** Recently, new autoantibodies specific for dermatomyositis (DM) have been identified. These disease-specific autoantibodies cover approximately 80% of the patients, and thus are useful for the diagnosis. Anti-transcriptional intermediary factor 1(TIF1) antibody has significant roles in oncogenesis. However, the usefulness of anti-TIF1 antibodies in juvenile dermatomyositis (JDM) is not clear.

**Objectives:** To evaluate the usefulness of Anti-transcriptional intermediary factor 1(TIF1) antibody in japanese patients with juvenile dermatomyositis (JDM).

**Methods:** We experienced three cases of the JDM with anti-TIF1 antibodies. In previous reports in Japan, 7 cases have been reported so far (Nagoya: 3 cases, Kanazawa: 4 cases). We analyze the clinical characteristics of these 10 patients in Japan categorized as JDM with anti-TIF1 antibodies.

**Results:** Three patients with active JDM with anti-TIF1 antibodies were observed in our hospital. All patients reported skin lesion. Two patients had elevated muscle enzymes aldolase. Only one patient has exhibited a severe systemic symptoms (fever, general fatigue, myositis). Ten patients with active JDM with anti-TIF1 antibodies were observed in Japan. There was no trend in the age of onset. No patients reported malignancy. Incidence of JDM with anti-TIF1 antibodies in Japan was 29% of whole JDM.

**Conclusion:** JDM with anti-TIF1 antibodies exhibited different clinical features. To establish the clinical significance of myositis-specific antibodies, the accumulation of cases is indispensable. In the near future, not only the tendency of each region, it is expected that the differences between all regions of the world will become apparent.

**Disclosure of Interest**

None Declared

### P113 Juvenile dermatomyositis in Arab children: a multi-center retrospective analysis of disease features and outcome

#### Sulaiman M. Al-Mayouf^1^, Nora AlMutiari^1,2^, Mohammed Muzaffer^3^, Rawiah shehata^4^, Adel Al-Wahadneh^5^, Reem Abdwani^6^, Safia Al-Abrawi^7^, Mohammed Abu-shukair^5^, Zeyad El-Habahbeh^5^, Abdullah Alsonbul^1^

##### ^1^King Faisal Specialist Hospital & Research Center, Riyadh, Saudi Arabia; ^2^King Faisal Specialist Hospital, Riyadh, Saudi Arabia; ^3^King Abdulaziz University, Jeddah, Saudi Arabia; ^4^Maternity & Children Hospital, Jeddah, Saudi Arabia; ^5^Queen Rania Children Hospital, Amman, Jordan; ^6^Qaboos Sultan University, Muscat, Oman; ^7^Royal Hospital, Muscat, Oman

###### **Presenting author:** Sulaiman M. Al-Mayouf

**Introduction:** Juvenile dermatomyositis (JDM) is a multisystem inflammatory disease affects primarily the skin and muscles. The clinical manifestations of JDM have been extensively described from different geographical parts of the world. The available published data from Arab countries about JDM is very limited.

**Objectives:** To describe disease characteristics and outcome of Arab children with JDM.

**Methods:** We retrospectively reviewed children with JDM seen between 1990 and 2014 in 5 rheumatology clinics from 3 Arab countries. All included patients fulfilled Bohan and Peter criteria for JDM, diagnosed before 14 years of age and were Arab ethnicity. Data were collected at the last follow up visit and comprised of age of presentation, follow up duration, clinical and laboratory features, treatment as well as the long-term outcomes including accrual disease damage and death related to JDM.

**Results:** A total of 89 JDM patients (55 girls) from Saudi Arabia (65.2%), Jordan (18%) and Oman (16.9%) were included. The mean age at onset was 6 years (±3), the mean age at diagnosis was 6.6 years (±3), and with mean follow up duration of 5 years (±4.4). Fifty patients had active follow up, 27 patients transferred to adult rheumatology service and 12 patients had lost follow up. Forty patients had polycyclic disease course, 34 had monocyclic course while 15 had continuous progressive course. Seventy-four patients had organ involvement, arthritis (48%), upper airway and dysphagia (14%), gastrointestinal (13%) and lung involvement (10%). Seven patients admitted to intensive care unit (ICU), 4 of them required mechanical ventilation. Dysphagia, upper airway and lung involvement were all statistically significant associated with ICU admission. All patients received corticosteroids; while methotrexate was the most frequently used immunosuppressive drugs (86%) and rituximab was used in 8 patients. Additionally 31 patients received IVIG. Twelve patients received pamidronate mainly for calcinosis. Most of the patients achieved complete clinical response but 16 ended with permanent skin changes and 12 had residual muscle weakness. Twenty-seven patients developed calcinosis, 15 had osteoporosis, and 3 had lipodystrophy while 37 patients had growth failure. One patient developed mucinous cystic ovarian tumor. There were two deaths due to infection during the follow up period.

**Conclusion:** Arab patients with JDM have similar characteristics to previously described cohorts. However, compared to our previous experience, a steadily improvement in outcome particularly calcinosis which probably related to better therapeutic approach.

**Disclosure of Interest**

None Declared

### P114 Nailfold capillaroscopy in children with juvenile dermatomyositis

#### Aleksandra Szabat, Monika Chęć, Violetta Opoka-Winiarska

##### Department of Pediatric Pulmonology and Rheumatology, Medical University of Lublin, Poland, Lublin, Poland

###### **Presenting author:** Violetta Opoka-Winiarska

**Introduction:** Juvenile dermatomyostis (JDM) is a systemic inflammatory microvasculopathy that primary affects skin and muscle The clinical course of JDM is variable and correlates with severity of vascular disease and vascular alternations taking place in the muscle, skin and diverse organs The microangiopathy characteristic of JDM can be confirmed by nailfold capillaroscopy(NC). NC is an important tool for noninvasive study of peripheral microcirculation in children with dermatomyositis.

**Objectives:** The aim of study was to comparison disease activity and nailfold capillaroscopy findings in children with juvenile deramatomyositis.

**Methods:** 13 children with diagnosed dermatomyositis (3 – 17 years old, 6 girls and 7 boys were included in the study. Age of the patients at the time of diagnosis was: 4 – 14 years, duration of illness at the time of study: 4 – 79 months. The evaluation of capillaries was performed using a stereoscopic microscope, the measurements were done using the program AxioVision. The capillary system was evaluated by assessesmant of number or loss of capillaries, the number of enlarged and giant capillaries, haemorrhages, neoangiogenic capillaries and their disorganization. The evaluation was made using the scoring system of the capillaroscopic parameters^1^. The score at the time of diagnosis and during improvemen or remission was compared.

**Results:** At the time of diagnosis microangiopathy with scleroderma-like pattern was detected in all patients. The most characteristic feature were reduced number of capillaries, neoangiogenic and enlarged capillaries. The capillary pattern was dependent on disease activity.

**Conclusion:** Capillaroscopy is simple and noninvasive method for the diagnosis and evaluation of disease activity in children with juvenile dermatomyositis.

**Trial registration identifying number:** ^1^Sulli A, Secchi ME, Pizzorni C, Cutolo M. Scoring the nailfold microvascular changes during the capillaroscopic analysis in systemic sclerosis patients. Ann Rheum Dis. 2008;67:885-7.

**Disclosure of Interest**

None Declared

## Poster Session: Scleroderma and related syndromes

### P115 A single centre experience of pediatric systemic sclerosis from North India

#### Ankur Kumar^1^, Anju Gupta^1^, Amit Rawat^1^, Biman Saikia^2^, Ranjana W. Minz^2^, Deepti Suri^1^, Surjit Singh^1^

##### ^1^Pediatrics, PGIMER, Chandigarh, India; ^2^Immunopathology, PGIMER, Chandigarh, India

###### **Presenting author:** Ankur Kumar

**Introduction:** Systemic sclerosis (SSc) is an inflammatory disorder with multisystem involvement characterized by progressive fibrosis affecting skin and various internal organs. It is subdivided clinically by the extent of skin involvement into diffuse and limited systemic sclerosis.

**Objectives:** We describe clinical and laboratory profile and outcome of pediatric SSc patients diagnosed and managed at a single centre in North India.

**Methods:** This was a retrospective case review of all children diagnosed as SSc based on Pediatric Rheumatology European Society/American College of Rheumatology/European League against Rheumatism provisional classification criteria for Juvenile Systemic sclerosis

**Results: A t**otal of 14 children (8 boys and 6 girls) were diagnosed as SSc between 1990 and 2014. Diffuse and limited cutaneous SSc were seen in 10 and 4 patients, respectively.

Median age at symptom onset and diagnosis was 7 years (range of 4-14 years) and 10 years (range 6-15 years), respectively. Progressive skin tightening (78%) and Raynaud phenomenon (71%) were the most common presenting manifestations. Raynaud phenomenon as the only presenting manifestation was present in 21% patients. Other common presenting features included restriction of joint movements due to skin tightening (57%), hyperpigmentation of skin (21%), arthritis (14%), dysphagia (30%) and dyspnoea on exertion (14%). Calcinosis and palpitation were the presenting manifestations in 1 patient each.

On evaluation, skin involvement [digital tip ulcers (50%), calcinosis (21%), salt-pepper pigmentation (14%), telangiectasia (14%) and acro-osteolysis (7%)] was present in all patients. Median Modified Rodnan Skin Score (MRSS) at presentation was 18. Skin biopsy was done in 8 patients and was suggestive of sclerodermatous skin changes in all. Capillaroscopy was done in 3 patients and was suggestive of capillary drop outs and tortuous loops.

Pulmonary involvement was seen in 10 patients. Only 2 of these were symptomatic at presentation. One patient developed ILD after 2 years of initial diagnosis of SSc. Pulmonary function test (PFT) was suggestive of restrictive pattern in 9/13 patients. Gastrointestinal tract involvement was seen in 30% patients with dysphagia as the most common clinical manifestation. Median juvenile Systemic Sclerosis Score (JSSS) was 6.5 at presentation (range 3- 12).

ANA was positive in 10/14 patients [speckled (4), nucleolar (4), mixed (1) and diffuse (1)]. RA factor was positive in 3/14 patients. CT chest showed features of ILD in 6/9 patients with ground glass opacity and honeycombing being common abnormalities. Mean follow up was for 2.07 years (3 months to 6 years). In majority of patients with major organ involvement, the treatment regimen included cyclophosphamide pulse therapy followed by oral azathioprine. No mortality was reported in our cohort of patients; however recent follow up was available for only 7 patients.

**Conclusion: I**n our cohort, we found a significant male preponderance. Progressive skin tightening and Raynaud phenomenon were the most common presenting manifestations

**Disclosure of Interest**

None Declared

### P116 Juvenile Localized Scleroderma (JLS): is it a benign disease?

#### Christine Arango^1^, Clara Malagon^1^, Maria D. P. Gomez^2^, Angela C. Mosquera^1^, Ricardo Yepez^2^, Tatiana Gonzalez^3^, Camilo Vargas^4^, and GRIP study group

##### ^1^Pediatric Rheumatology, El Bosque University, Bogota, Colombia; ^2^Pediatric Rheumatology, Universidad Libre, Cali, Colombia; ^3^Pediatric Rheumatology, Universidad de Cartagena, Cartagena, Colombia; ^4^Pediatric Rheumatology, Universidad Del Valle, Cali, Colombia

###### **Presenting author:** Christine Arango

**Introduction:** JLS is a polymorphic autoimmune disease. The Pediatric Rheumatology European Society (PRes) classification criteria help to identify different clinical features. The follow up of the patients allow the recognition of morbidity and complications derived from the disease.

**Objectives:** Describe demographic, clinical features, morbidity and sequela derived from JLS.

**Methods:** Multicentre descriptive retrospective study. Analysis of clinical records of patients with a definitive diagnosis of JLS in 10 pediatric rheumatology clinics. The patients had a minimum follow up of 6 months and a minimum of 12 months of diagnosis. Morbidity was defined as: unsightly effects (dyscromic, disfigurement lesions and/or alopecia). Sequela was defined as functional joint involvement and growth disturbances (longitudinal, circumferencial or mixed).

**Results:** n = 88. Mean age at diagnosis 6.9 years (0-14 years) with a sex ratio F:M 2,1:1. The mean follow up time was 43 months (6-243 months). Mean time between symptoms appearance and diagnosis was 16,5 months (1-96 months). AAN were positive in 42% of patients. According to PRES criteria the distribution was: circumscribed morphea (33%), mixed (32%), linear (22%), generalized (11%) and panesclerotic (2%). The clinical phenotypes of the predominant subtypes are described in Table 41. 67% developed multiple lesions. 22% had extra cutaneous involvement (neurological, ocular and articular) and 13% had another autoimmune disease. Circumscribed was the most common type, morbidity was related to unsightly effects. In mixed type the association between linear and circumscribed lesions account for 75%. This type had multiple complications (unsightly and functional effects). Linear lesions induced the three types of morbidity and showed the higher polyautoimmunity rate. The coup du sabre type had the longest duration between symptons and diagnosis, the higher extra cutaneous involvement and a significant morbidity. Generalized scleroderma developed important unsightly and functional effects. Pansclerotic was the least common, 2 girls being affected by a severe crippling disease. The frecuency of functional joint involvement, growth disturbance and multiple complications was higher in the mixed, linear and generalized types, being statistically significant.

**Conclusion:** JSC is a polymorphic and unpredictable disease that determines important morbidity. Late diagnosis is common and might have a negative impact on prognosis because it allows the progression of the lesions, their size, depth and number. The high number of extra cutaneous complications reflects that this is not a disease limited to the skin. Polyautoimmunity reflects an immune system dysregulation. An early diagnosis and a more dynamic immunosuppressive treatment may improve the prognosis. Patients need a regular and extended follow up.

**Disclosure of Interest**

None Declared

**Table 41 (abstract P116).** Clinical features of the most prevalent subtypes

	Circumscribed	Mixed	Linear	Generalized	CDS	Total	P value
Frequency (%)	29	28	12	10	7	86	
Mean age at diagnosis(years)	8,0	6,2	8,1	7,3	5,9	6,9	0,255
Disease duration at diagnosis	11,9	22,6	13,6	12,6	23,9	16,8	0,623
Extra cutaneous involvement n(%)	2(6,9)	7(25)	3(25)	2(20)	3(42,9)	17(19,8)	0,194
Autoimmune associated disease n(%)	4(3,8)	4(14,3)	2(16,7)	1(10)	0(0)	11(12,8)	0,854
Unsightly effects n(%)	25(86,2)	25(89,3)	12(100)	9(90)	7(100)	78(90,7)	0,607
Joint functional involvement n(%)	0(0)	15(53,6)	6(50)	6(60)	0(0)	27(31,4)	0,000
Growth disturbances n(%)	3(10,3)	17(60,7)	7(58,3)	2(20)	5(71,4)	34(39,5)	0,000
2 or more complications n(%)	3(10,3)	19(67,9)	7(58,3)	7(70)	5(71,4)	41(47,7)	0,001

### P117 Magnetic resonance and echocardiographic strain rate imaging for the early detection of cardiac involvement in Juvenile Systemic Sclerosis

#### Francesco Zulian^1^, Marta Balzarin^1^, Biagio Castaldi^2^, Elena Reffo^2^, Francesca Sperotto^1^, Giorgia Martini^1^, Alessandra Meneghel^1^, Ornella Milanesi^2^

##### ^1^Pediatric Rheumatology Unit, University of Padua, Padua, Italy; ^2^Pediatric Cardiology Unit, University of Padua, Padua, Italy

###### **Presenting author:** Francesco Zulian

This abstract is not included here as it has already been published.

### P118 Update on the Juvenile Systemic Sclerosis inception cohort project. Characteristics of the first 80 patients at first assessment. Www.juvenile-scleroderma.com

#### Ivan Foeldvari^1^, Jens Klotsche^2^, Ozgur Kasapçopur^3^, Amra Adrovic^3^, Valda Stanevicha^4^, Maria Teresa Terreri^5^, Ekaterina Alexeeva^6^, Maria Katsicas^7^, Rolando Cimaz^8^, Mikhail Kostik^9^, Thomas Lehman^10^, W.-Alberto Sifuentes-Giraldo^11^, Vanessa Smith^12^, Flavio Sztajnbok^13^, Tadey Avcin^14^, Maria Jose Santos^15^, Dana Nemcova^16^, Cristina Battagliotti^17^, Despina Eleftheriou^18^, Liora Harel^19^, Mahesh Janarthanan^20^, Tilmann Kallinich^2^, Jordi Anton Lopez^21^, Kirsten Minden^2^, Susan Nielsen^22^, Kathryn Torok^23^, Yosef Uziel^24^, Nicola Helmus^1^

##### ^1^Am Schön Klinik Eilbek, Hamburg, Germany; ^2^Pediatric Rheumatology, Berlin, Germany; ^3^Pediatric Rheumatology, Istanbul, Turkey; ^4^Pediatric Rheumatology, Riga, Latvia; ^5^Pediatric Rheumatology, Sao Paulo, Brazil; ^6^Pediatric Rheumatology, Moskva, Russian Federation; ^7^Pediatric Rheumatology, Buenos Aires, Argentina; ^8^Pediatric Rheumatology, Florence, Italy; ^9^Pediatric Rheumatology, St. Petersburg, Russian Federation; ^10^Pediatric Rheumatology, New York, United States; ^11^Pediatric Rheumatology, Madrid, Spain; ^12^Pediatric Rheumatology, Gent, Belgium; ^13^Pediatric Rheumatology, Rio de Janeiro, Brazil; ^14^Pediatric Rheumatology, Ljubljana, Slovenia; ^15^Pediatric Rheumatology, Almada, Portugal; ^16^Pediatric Rheumatology, Prague, Czech Republic; ^17^Pediatric Rheumatology, Santa Fee, Argentina; ^18^Pediatric Rheumatology, London, United Kingdom; ^19^Pediatric Rheumatology, Nettnja, Israel; ^20^Pediatric Rheumatology, Chennai, India; ^21^Pediatric Rheumatology, Barcelona, Spain; ^22^Pediatric Rheumatology, Copenhagen, Denmark; ^23^Pediatric Rheumatology, Pittsburgh, United States; ^24^Pediatric Rheumatology, Kfar Saba, Israel

###### **Presenting author:** Ivan Foeldvari

**Introduction:** Juvenile systemic sclerosis (jSSc) is an orphan autoimmune disease. Currently just retrospective data exist regarding evolvement of organ involvement. In the retrospective studies assessment of the organ involvement is not standardized. Our project is the first project, where prospectively and with a standardized assessment data of jSSc patients are collected. We present the data of the patients at the entry into the cohort.

**Objectives:** to learn about the characteristics and evolvement of jSSc

**Methods:** Patients with jSSc, according the PRES criteria, were recruited worldwide and were prospectively assessed, using the proposed standardized patient assessment protocol.

**Results:** 26 centers from 17 countries applied to participate on the project. The assent and consent forms were translated into the local native languages. Up till now 80 patients were enrolled. Sixty-six (82.5%) of the 80 patients were female. The mean age of the onset of Raynaud symptomatic was 9.4 years (0.2 – 15.9). The mean age at the onset of the non-Raynaud symptomatic were 9,9 years (0.3 -15.9). 58 (72.5%) had diffuse subtype and 22 had limited subtype (27.5%). 11 (14%) of them have an overlap symptomatic, in 6 (10%) in the diffuse and 5 (23%) in limited subtype.

At the time of the inclusion the mean modified Rodnan Skin Score was 15.7 (0-51). ANA positive were 60/77 (78%), 24/77 (31%) of them were anti-Scl 70 positive and 4/46 (9%) were anticentromere positive. 48/80 (60%) had already capillary changes and 36/78 (46%) inactive ulcerations, 13/78 (17%) had active ulceration at the time of the inclusion. 39/80 (49%) had cardiopulmonary involvement, 22/45 (49%) of had signs of interstitial lung disease on imaging, 16/36 (44%) had FVC <80% and 10/19 (52%) had DLCO < 80%. 6/44 (14%) patients had pulmonary hypertension. The mean 6 Minute Walk Test was 419.3 m (60-615). 5/80 (6%) had renal involvement, none of them had hypertension. 26/80 (32.5%) had gastrointestinal involvement, and 18/26 (69%) of them esophageal involvement. 48/79 (62%) had musculoskeletal involvement, mostly limited range of joint movement in 21/49 (43%). Isolated muscle weakness occurred in 3/46 (6.5%). 2/80 (2.5%) showed neurologic involvement.

**Conclusion:** We present the data on the first 80 patients with jSSc at the time of inclusion in our cohort. The current recruitment data confirms that pediatric patients are different from the adult patients; there is a significantly higher proportion of diffuse subset patients with 72.5%. 14% of the patients had an overlap features. The patients had significant disease burden assessed by patients and physician at the entry of the cohort.

**Disclosure of Interest**

None Declared

### P119 Proposal of assessment of the activity of Juvenile Localised Scleroderma. Results of the consensus meeting in Hamburg, Germany December 2015

#### Ivan Foeldvari^1^, Eileen Baildem^2^, Michael Blakley^3^, Christina Boros^4^, Kim Fligelstone^4^, Antonia Kienast^1^, Dana Nemcova^5^, Clare Pain^2^, Amanda Saracino^4^, Gabriele Simoni^6^, Kathryn Torok^7^, Lisa Weibel^8^, Nicola Helmus^1^

##### ^1^Am Schön Klinik Eilbek, Hamburg, Germany; ^2^Pediatric Rheumatology, Liverpool, United Kingdom; ^3^Pediatric Rheumatology, Indianapolis, United States; ^4^Pediatric Rheumatology, London, United Kingdom; ^5^Pediatric Rheumatology, Prague, Czech Republic; ^6^Pediatric Rheumatology, Firenze, Italy; ^7^Pediatric Rheumatology, Pittsburgh, United States; ^8^Pediatric Rheumatology, Zürich, Switzerland

###### **Presenting author:** Ivan Foeldvari

**Introduction:** Juvenile Localised Scleroderma (JLS) is an orphan disease which is complicated by difficulties in robust measurement of disease activity. Several outcome measures to assess disease activity have been described with some recent trials using the mLoSSI (modified localized scleroderma skin severity index) which is a validated cutaneous assessment tool in JLS. Whilst such measures are a step forward in assessment of this disease, they do not capture all aspects of the disease that are taken into consideration by clinicians when judging the degree of disease activity and therefore making decisions regarding treatment. In particular extra-cutaneous manifestations are poorly captured. With potential innovative and more effective treatment options emerging, it has become extremely important to define a validated activity index that captures skin, extra-cutaneous disease activity and patient reported outcomes in order to monitor response to treatment.

**Objectives:** To develop a proposal for an extended activity index, which includes extracutaneous involvement and the patient reported outcomes to better measure disease activity. This composite activity measure should be sensitive to change to assess the efficacy of treatment of JLS.

**Methods:** Members of the PRES Scleroderma working group and other paediatric rheumatologists and dermatologists interested in JLS met to develop an activity index using the nominal group technique in a consensus meeting in Hamburg, Germany, in December 2015. 80% agreement was chosen for the selected domains and items.

**Results:** The proposed domains and items to assess the activity of localised scleroderma:

**Skin:**

Change in skin thickening – *Modified Rodnan Skin Score (mRSS); Total Skin thickness score of mLoSSi; Research tool: A, durometer B, Ultrasound*

Degree of change in white waxy appearence since last visit – *Marked worsening /some worsening and some improvemnet/no change/ significant improvement*

Change in Erythema/violaceousness since last visit – *mLoSSI*

Change in Subcutaneous induration since last visit – *Yes or no & elements of mLoSSi*

Enlargement of the lesion since last visit – *Yes or no (captured within mLoSSi)*

New lesion per anatomical region of mLoSSI – *mLoSSI*

Worsening alopecia (eyelash, beard, body hair) – *marked worsening,/some worsening and some improvement/no change/ significant improvement/no hair in the lesion / decreased*

Increase in face atrophy – only Parry Romberg patients – *3 D photography*

Optional item: Change in blood flow in the lesion - *Ultrasound with doppler / laser doppler*

**Extracutaneous involvement:**

Change in Activity of anterior uveitis since last visit – *Yes or no*

Change in active joint count – *Number of active joints*

Worsening of lim discrepancy – *Yes or no*

Active CNS involvement – *Yes or no*

**Patient reported outcomes:**

Change in subcutaneous induration since last visit – *marked worsening /some worsening and some improvemnet/ no change/ significant improvement*

Uncomfortable feeling in the lesion (not including itch) – *VAS score 0-100*

Itching in the lesion – *VAS score 0-100*

Quality of life assessment – *PEDsQL, CHQ, CDLQII*

Functional assessment of daily life – *CHAQ/HAQ*

**Conclusion:** We propose extended domains to define an activity index for JLS which aim to capture the heterogeneity of disease activity. This may become an important instrument for evaluating treatment efficacy but requires prospective validation from the PRES and CARRA scleroderma working groups.

**Disclosure of Interest**

None Declared

### P120 Medication use in the Juvenile Systemic Sclerosis inception cohort. Are there differences in the diffuse and localised subset patients? Www.juvenile-scleroderma.com

#### Ivan Foeldvari^1^, Jens Klotsche^2^, Ozgur Kasapçopur^3^, Amra Adrovic^3^, Valda Stanevicha^4^, Maria Teresa Terreri^5^, Ekaterina Alexeeva^6^, Maria Katsicas^7^, Rolando Cimaz^8^, Mikhail Kostik^9^, Thomas Lehman^10^, W.-Alberto Sifuentes-Giraldo^11^, Vanessa Smith^12^, Flavio Sztajnbok^13^, Tadey Avcin^14^, Maria Jose Santos^15^, Dana Nemcova^16^, Cristina Battagliotti^17^, Despina Eleftheriou^18^, Liora Harel^19^, Mahesh Janarthanan^20^, Tilmann Kallinich^2^, Jordi Anton Lopez^21^, Kirsten Minden^2^, Susan Nielsen^22^, Kathryn Torok^23^, Yosef Uziel^24^, Nicola Helmus^1^

##### ^1^Am Schön Klinik Eilbek, Hamburg, Germany; ^2^Pediatric Rheumatology, Berlin, Germany; ^3^Pediatric Rheumatology, Istanbul, Turkey; ^4^Pediatric Rheumatology, Riga, Latvia; ^5^Pediatric Rheumatology, Sao Paulo, Brazil; ^6^Pediatric Rheumatology, Moskva, Russian Federation; ^7^Pediatric Rheumatology, Buenos Aires, Argentina; ^8^Pediatric Rheumatology, Florence, Italy; ^9^Pediatric Rheumatology, St. Petersburg, Russian Federation; ^10^Pediatric Rheumatology, New York, United States; ^11^Pediatric Rheumatology, Madrid, Spain; ^12^Pediatric Rheumatology, Gent, Belgium; ^13^Pediatric Rheumatology, Rio de Janeiro, Brazil; ^14^Pediatric Rheumatology, Ljubljana, Slovenia; ^15^Pediatric Rheumatology, Almada, Portugal; ^16^Pediatric Rheumatology, Prague, Czech Republic; ^17^Pediatric Rheumatology, Santa Fee, Argentina; ^18^Pediatric Rheumatology, London, United Kingdom; ^19^Pediatric Rheumatology, Nettnja, Israel; ^20^Pediatric Rheumatology, Chennai, India; ^21^Pediatric Rheumatology, Barcelona, Spain; ^22^Pediatric Rheumatology, Copenhagen, Denmark; ^23^Pediatric Rheumatology, Pittsburgh, United States; ^24^Pediatric Rheumatology, Kfar Saba, Israel

###### **Presenting author:** Ivan Foeldvari

**Introduction:** Juvenile systemic sclerosis (jSSc) is an orphan autoimmune disease. Currently there is no data regarding use of medication in jSSc patients. Our project is the first project, where prospectively and with a standardized assessment data of jSSc patients are collected and the applied medication assessed. We present the data of the medication us at the time point of entry into the cohort.

**Objectives:** to learn about the medication use in jSSc patients

**Methods:** Patients with jSSc, according the PRES criteria, were recruited worldwide and were prospectively assessed, using the proposed standardized patient assessment protocol.

**Results:** Up till now 80 patients were enrolled. Sixty-six (82.5%) of the 80 patients were female. 58 (72.5%) had diffuse subtype (djSSc) and 22 had limited subtype (27.5% (ljSSc). 88% of the patients in the djSSc and 86% in the ljSSc patients received medication.

**Conclusion:** Interestingly the most frequently used DMARD was Methotrexate, followed by Mycophenolate. Only small number of patients received biologics. Interestingly higher number of patients in the diffuse group received Bosentan. Interestingly 9 patients did not received any DMARDS /biologics or medication again pulmonary hypertension.

**Disclosure of Interest**

None Declared

**Table 42 (abstract P120).** See text for description

Number of patients	Whole group	Diffuse subtype	Limited subtype	0.945
Sex female /male	80	57 (71%)	23 (29%)	
Medication	66/14 = > 4.7:1	47/10 = > 4.7:1	20/4 = > 5:1	
	62/71 (87%)	44/50 (88%)	18/21 (86%)	
Corticosteroids	36/62 (58%)	27/44 (61%)	9/18 (50%)	0.667
Cyclophosphamide	5/62 (8%)	5/44 (11%)	0/18 (0%)	0.159
Chloroquine/Hydroxy-chlorqquine	10/62 (16%)	6/44 (14%)	4/18 (22%)	0.485
Methotrexate	35/62 (56%)	24/44 (54%)	11/18 (61%)	0.804
Mycophenolate	11/62 (18%)	8/44 (18%)	3/18 (17%)	0.905
Azathioprin	1/62 (2%)	1/44 (2%)	0/18 (0%)	0.524
Tocolizumab	1/62 (2%)	0/44 (0%)	1/18 (6%)	0.125
Rituximab	2/62 (3%)	1/4 (2%)	1/18 (6%)	0.523
Bosentan	11/62 (18%)	10/44 (23%)	1/18 (6%)	0.165
PDE5 inhibitors	4/62 (6%)	3/4 (7%)	1/18 (6%)	0.863
Prostanoids	1/62 (2%)	0/44 (0%)	1/18 (6%)	0.125

### P121 Cone beam computed tomograhy for evaluation of Craniofascial abnormalities in case of linear scleroderma en coup de sabre

#### Maria K. Osminina^1^, Nathalia A. Geppe^1^, Olga V. Niconorova^2^, Olesya V. Karashtina^2^, Oksana V. Abbyasova^2^, Olga V. Shpitonkova^1^

##### ^1^Pediatric department, I.M.Sechenov First Moscow State Medical University, Moscow, Russian Federation; ^2^Pediatric department, Pediatric outpatien clinic of Public Joint-stock company Gazprom, Moscow, Russian Federation

###### **Presenting author:** Maria K. Osminina

**Introduction:** Scleroderma en coup de sabre (SECDS) is severe disease variety, that results in hemiatrophy of skin, soft tissues, bones accompanied by aesthetical, odontostomatologic abnormalities, central nervous system, ocular involvement, deformation of skull bones . Therapeutic response to immunosuppressive therapy in SECDS is rather poor in comparison to linear scleroderma of limbs and trunk. Identification of scleroderma lesions of skull bones seems difficult, but very important for treatment efficacy estimation and planning of thurther reconstructive plastic surgery.

**Objectives:** To estimate the development of skull bones disturbances in children with SECDS under disease-modifying therapy we used cone beam computed tomography(CBCT)

**Methods:** All the images were acquired using CBTC scanner (Gendex CB-500, powered by i-cat, Germany). We measuared height of frontal, alveolar, maxillar and mandibular bones in the coronal & axiall sections; bone density in Houndsfield units(HU), thickness of soft tissues, comparing left and right parts of facial skull.

**Results:** CBTC scanning of facial skull were performed 5 girls with SECDS aged from 8-18 years, in 2- CBTC scanning was done twice in 6 months intervals. Disease duration ranged from 8 months to 10 years (mean was 8.5 years). All patients have received disease-modifying therapy-corticosterods orally 0.5-1 mg\kilo for 12 weeks plus methotrexate 10-12 mg\body sq.\weekly for 24-36moths, in 3 girls after 3 years the treatment was withdrawn, as scleroderma skin progression stopped and reversed partly. In all the cases we detected decrease of soft tissue amount on the site of scleroderma damage. In 4 girls (disease duration more than 7 years) height of maxillar and mandibular bones was reduced on envolved site of face. Frontal bones were thinned, with abnormal architectonics. Shape deformations of bones were significant. Density of underlying bones was diminished (143-148 HU) in cases with skin scleroderma changes in active phase (odema, induration), on the contrary- fibrotic skin and soft tissue- correlates with increased bone density(833-935 HU). Repeated in 6 months CBTC scanning helped to correct disease-modifying therapy.

**Conclusion:** Our pilot study of CBCT scanning of facial skull in SECDS showed the method as a helpfuii tool in visualization of facial strucrutes. Further investigations couil find out referrence points and lines for more informative scanning, give a key for interpretation of results, its’ correlation with clinical presentations and treatment efficacy

**Disclosure of Interest**

None Declared

### P122 Role of pentraxin 3 in patients with juvenile scleroderma

#### Amra Adrovic^1^, Sezgin Sahin^1^, Kenan Barut^1^, Sinem Durmus^2^, Hafize Uzun^2^, Ozgur Kasapcopur^1^

##### ^1^Pediatric Rheumatology, ^2^Biochemistry, Istanbul University, Cerrahpasa Medical School, Istanbul, Turkey

###### **Presenting author:** Amra Adrovic

**Introduction:** Juvenile scleroderma (JS) is a rarely seen chronic connective tissue disorder. Juvenile systemic scleroderma (JSS) has far worse prognosis with multi-organ involvement and possible life-threatening complications. Its main pathophysiological characteristics include microvascular abnormalities and excessive fibrosis of the skin, subcutaneous tissues and internal organs.

Pentraxin 3 (PTX 3) is a multifunctional protein produced at the inflammation site by macrophages, dendritic cells, endothelial cells, smooth muscle cells and fibroblasts. Among its other functions, PTX 3 suppresses fibroblast growth factor 2 (FGF 2) in systemic sclerosis. Previously studies reported an increased level of PTX 3 among adult patients with scleroderma. Study among patients with JS has not been provided yet.

**Objectives:** We aimed to measure the level of PTX 3 in patients with juvenile scleroderma comparing to healthy children. Thereby, we have tried to give an answer on question whether the PTX 3 could be a marker of fibrosis in patients with juvenile scleroderma.

**Methods:** We assessed patients with JSS and those with juvenile localized scleroderma (JLS) and age- and sex- matched healthy controls. A complete medical history, physical examination, and laboratory evaluation were performed for each patient at the time of enrollment. The same physical examination and laboratory investigation was performed in healthy controls, in order to exclude the coincidental disease. Circulating PTX3 levels were measured by enzyme immunoassay. The lower limits of detection for PTX3 was 0.1 ng/ml.

**Results:** We assessed 24 patients with JSS, 20 patients with JLS and 41 healthy controls. The mean age of patients was 15.38 ± 3.156, 12.44 ± 3.63 and 14.33 ± 3.48 for JSS, JLS and healthy controls, respectively. Mean disease duration was 2 years (range: 0.6 -15 years) for JSS and 1.5 years (range: 0.6 - 18 years) for JLS patients.

Mean serum level of PTX 3 was 10.63 ± 8.61 ng/ml, 11.75 ± 9.11 ng/ml and 2.76 ± 1.338 ng/ml for JSS, JLS and healthy controls, respectively. There was remarkable increased level of PTX 3 in serum of patients with both types of juvenile scleroderma. In both of patients group (systemic and localized form of the disease), PTX 3 level was significantly higher comparing to healthy children (p < 0.001).

We didn’t find statistically significant difference between JSS and JLS patients according to level of PTX3.

A mean modified Rodnan skin score of JSS patients was 19.95 ± 11.088. PTX3 level was found to be in positive correlation with modified Rodnan skin score, in patients with JSS (Rho = 0.497, p = 0.030).

**Conclusion:** The circulating PTX3 level is significantly higher in both JSS and JLS patients than in healthy control subjects. The possible explanation is that fibrosis and increased fibroblast activation represent main pathophysiological mechanism in both form of the disease. This result support relevance of PTX 3 measurement in order to determinate fibrosis activity.

Since it is produced by activated fibroblasts in the area of inflammation and fibrosis, a PTX 3 should be considered a relevant marker of fibrosis in patients with juvenile scleroderma.

**Disclosure of Interest**

None Declared

### P123 Is there a difference in the presentation of diffuse and limited subtype in childhood? Results from the juvenile scleroderma inception cohort. Www.juvenile-scleroderma.com

#### Ivan Foeldvari^1^, Jens Klotsche^2^, Ozgur Kasapçopur^3^, Amra Adrovic^3^, Valda Stanevicha^4^, Maria Teresa Terreri^5^, Ekaterina Alexeeva^6^, Maria Katsicas^7^, Rolando Cimaz^8^, Mikhail Kostik^9^, Thomas Lehman^10^, W.-Alberto Sifuentes-Giraldo^11^, Vanessa Smith^12^, Flavio Sztajnbok^13^, Tadey Avcin^14^, Maria Jose Santos^15^, Dana Nemcova^16^, Cristina Battagliotti^17^, Despina Eleftheriou^18^, Liora Harel^19^, Mahesh Janarthanan^20^, Tilmann Kallinich^2^, Jordi Anton Lopez^21^, Kirsten Minden^2^, Susan Nielsen^22^, Kathryn Torok^23^, Yosef Uziel^24^, Nicola Helmus^1^

##### ^1^Am Schön Klinik Eilbek, Hamburg, Germany; ^2^Pediatric Rheumatology, Berlin, Germany; ^3^Pediatric Rheumatology, Istanbul, Turkey; ^4^Pediatric Rheumatology, Riga, Latvia; ^5^Pediatric Rheumatology, Sao Paulo, Brazil; ^6^Pediatric Rheumatology, Moskva, Russian Federation; ^7^Pediatric Rheumatology, Buenos Aires, Argentina; ^8^Pediatric Rheumatology, Florence, Italy; ^9^Pediatric Rheumatology, St. Petersburg, Russian Federation; ^10^Pediatric Rheumatology, New York, United States; ^11^Pediatric Rheumatology, Madrid, Spain; ^12^Rheumatology and Internal Medicine, Gent University Hospital, Gent, Belgium; ^13^Pediatric Rheumatology, Rio de Janeiro, Brazil; ^14^Pediatric Rheumatology, Ljubljana, Slovenia; ^15^Pediatric Rheumatology, Almada, Portugal; ^16^Pediatric Rheumatology, Prague, Czech Republic; ^17^Pediatric Rheumatology, Santa Fee, Argentina; ^18^Pediatric Rheumatology, London, United Kingdom; ^19^Pediatric Rheumatology, Nettnja, Israel; ^20^Pediatric Rheumatology, Chennai, India; ^21^Pediatric Rheumatology, Barcelona, Spain; ^22^Pediatric Rheumatology, Copenhagen, Denmark; ^23^Pediatric Rheumatology, Pittsburgh, United States; ^24^Pediatric Rheumatology, Kfar Saba, Israel

###### **Presenting author:** Ivan Foeldvari

**Introduction:** Juvenile systemic sclerosis (jSSc) is an orphan autoimmune disease. Several adult publications looked at the differences between limited (ljSSc) and diffuse subtype (djSSc). There is few data regarding this topic in pediatric jSSc. The juvenile scleroderma inception cohort is a prospective standardized register for patients with jSSc.

**Objectives:** Comparison of the features of ljSSc and djSSc patients at the time of inclusion in the registry.

**Methods:** Patients with jSSc were included worldwide to the jSSc cohort. Patients fulfilled the PRES criteria for jSSc. We compared the demographics and clinical features of the ljSSc and djSSc patients.

**Results:** Up till now 80 patients were enrolled, 58 (72.5%) with djSSc and 22 (27.5%) with ljSSc. 10% in djSSc and 23% in ljSSc showed overlap features. Disease duration at time of inclusion in the cohort was 3.7 years in the djSSc and 3.0 years in ljSSc. 83% in the djSSc and 81% in the ljSSc group were female. The mean age at onset of Raynaud symptoms was 9.0 years in the jdSSc and 10.4 years in ljSSc group and the mean age at onset of the non-Raynaud symptoms was 9.4 in djSSc and 10.9 ljSSc.

At the time of inclusion the mean of modified Rodnan Skin Score was 18.2 in the djSSc and 9.1 in ljSSc. Anti-Scl 70 positivity was found in 30.4% of djSSc and 33.3% in ljSSc. Only 2 patients in the djSSc group and 2 in the ljSSc group presented anticentromere positive. Capillary changes occurred in 62.1% in the djSSc and 54.5% in ljSSc . History of active ulceration was present in 56% in the djSSc and in18% in ljSSc . Active ulcerations were present in 21.1% in the djSSc and 4.5% in the ljSSc. The mean of 6 Minute walk test was 392 m in the djSSc group and 504 m in the ljSSc group. 15.5% of djSSc and 41% of ljSSc had cardiac involvement. Pulmonary hypertension occurred in 8% in djSSc and 13% in ljSSc. 56% in djSSc and 31% in ljSSc group had signs of interstitial lung disease on imaging. Renal involvement occurred in 7% in djSSc and 4.5% in ljSSc. None had systemic hypertension. 38% of djSSc and 18% of ljSSc had gastrointestinal involvement. 57% in djSSc and 73% in ljSSc had musculoskeletal involvement.

**Conclusion:** Patients with djSSc were younger at onset; have more often capillary changes, active ulcerations and gastrointestinal involvement, and less pulmonary hypertension and musculoskeletal involvement. They also present more severe disease and more disease damage. Features of the pediatric subtypes of jSSc differ from adults with SSc, especially the high proportion of patients with diffuse subtype.

**Disclosure of Interest**

None Declared

## Poster Session: Vasculitides

### P124 Epidemiology of Kawasaki disease in Italy: surveillance from national hospitalization records

#### Angela Mauro^1^, Eleonora Fanti^2^, Fabio Voller^2^, Franca Rusconi^3^, Rolando Cimaz^4^

##### ^1^Rheumatology Unit, Second University of Naples, Naples, Italy; ^2^Epidemiology Unit, Regional Health Agency of Tuscany, Florence, Italy; ^3^Epidemiology Unit, Anna Meyer Children’s University Hospital, Florence, Italy; ^4^Rheumatology Unit, Anna Meyer Children’s Hospital, Florence, Italy

###### **Presenting author:** Angela Mauro

**Introduction:** Kawasaki disease is a systemic vasculitis with an acute and self-limited course. In view of the potential severity of the illness, of its possible genetic background, and its unknown etiology, it is important to obtain epidemiological data in different populations. The incidence of the disease differs widely among ethnic groups and is higher in the Asian populations. In Italy, no recent data are available.

**Objectives:** The aim of the present study was to define the epidemiology of KD in the years 2008-2013 in children aged < 15 years in Italy through administrative data collection.

**Methods:** We studied the epidemiology of Kawasaki disease in the years 2008-2013 in children < 15 years old in Italy using hospital ICD-9 discharge codes with a thorough data cleaning for duplicate in order to select the first hospital admission for the disease.

**Results:** Between January 2008 and December 2013, 2901 children and adolescents younger than 15 years had a first hospital admission for KD in Italy**.** The disease peaked in the first two years of life, with 85.5% of cases under 5 years. Male: female ratio was 1.4:1. The incidence rate was 5.7 for 100,000 children 0-14 years old and 14.7 for children younger than 5 years. The incidence rate in children younger than 5 years in different Regions was < 10 cases x 100,000 children in two small regions in Northern Italy, between 10 and 15 in the majority of the Regions, and above 15 in 5 regions in Central Italy just under the Apennines and in Sicily. The incidence rose slightly during the study period and had a seasonal distribution, with higher incidence in spring and in winter. A cardiac complication was recorded in 6.4% of the patients.

**Conclusion:** This is the first epidemiologic study on Kawasaki disease incidence in the country of Italy. Figures are in line but slightly higher than those reported for other European countries.

**Disclosure of Interest**

None Declared

### P125 Is Harada Score a useful tool in Mexican children with Kawasaki disease?

#### Fernando Garcia-Rodriguez^1^, Ana V. Villarreal-Treviño^1^, Angel J. Flores-Pineda^1^, Paola B. Lara-Herrea^1^, Diego R. Salinas-Encinas^1^, Talia Diaz-Prieto^1^, Maria R. Maldonado-Velazquez^1^, Sarbelio Moreno-Espinosa^2^, Enrique Faugier-Fuentes^1^

##### ^1^Pediatric Rheumatology, Hospital Infantil De Mexico Federico Gomez, Mexico City, Mexico; ^2^Pediatric Infectology, Hospital Infantil De Mexico Federico Gomez, Mexico City, Mexico

###### **Presenting author:** Fernando Garcia-Rodriguez

**Introduction:** Kawasaki Disease (KD) is the most frequent systemic vasculitis in children. Harada score (HS) had been proposed as a useful tool in detecting patients at risk to develop coronary arteries aneurysms (CAA) in patients with KD.

**Objectives:** To describe the usefulness of HS to predict the risk for CAA development in Mexican children with KD.

**Methods:** We retrospectively collected data from patients with diagnosis of KD between January 2004 and December 2014 at Hospital Infantil de México Federico Gómez. Demographic, clinical, laboratory results, echocardiographic reports and treatment information were reviewed and analyzed to assess the reliability of HS in KD.

**Results:** During the study 204 cases were analyzed, most (54.9%) male, median age 32.5 (6 - 120) months with hospitalization rate 96.1%. Twenty per-cent of patients presented as incomplete KD. We did not found any differences between complete and incomplete KD regarding weight, height or vital signs at diagnosis.

Fever (100%), conjunctivitis (89.2%), oral changes (84.3%), pharyngitis (88.2%), and strawberry tongue (83.3%) were the most frequent findings on physical exam. We found acute phase reactants values higher in those patients with classic presentation of KD when compared with incomplete presentation. Echocardiographic abnormal findings presented in 60 (29.4%) patients (11.8% were ectasia and 10.8% CAA).

Most of the patients (82.8%) received IVIG, of those cases, 18 (8.8%) presented resistance to IVIG, 6 (2.9%) required corticosteroids, and 2 (1%) infliximab; all patients were treated with acetylsalicylic acid.

Median HS in all the patients was 4 (0 - 6), finding significant difference between classic and incomplete KD (4 [0 - 6] vs 2.5 [0 - 5], respectively; P < .001). HS showed an OR 6.17 (IC95%: 2.82-13-5) for detecting any cardiac involvements, 4 (IC95%: 1.31-12.17) for ectasia, and 3.52 (IC95%: 1.14-10.84) for CAA.

**Conclusion:** We did not found important differences between classic and incomplete presentations of KD. HS demonstrated to be a reliable screening tool to detect patients at risk for chronic cardiac involvement in Mexican children with KD.

**Disclosure of Interest**

None Declared

### P126 Behçet disease in the pediatric age: data on 129 patients collected from an Italian cohort

#### Romina Gallizzi^1^, Martina Finetti^2^, Mirella Crapanzano^1^, Luca Cantarini^3^, Marco Cattalini^4^, Giovanni Filocamo^5^, Antonella Insalaco^6^, Angela Mauro^7^, Donato Rigante^8^, Francesco Zulian^9^, Maria Alessio^10^, Ilaria Parissenti^4^, Nicolino Ruperto^2^, Marco Gattorno^2^, Rolando Cimaz^11^

##### ^1^Aou G.Martino, Messina, Italy; ^2^Istituto Gaslini, Genova, Italy; ^3^Policlinico Le Scotte, Siena, Italy; ^4^Clinica Pediatrica, Brescia, Italy; ^5^Ospedale Maggiore, Clinica Pediatrica II De Marchi, Milano, Italy; ^6^Ospedale Bambin Gesù, Roma, Italy; ^7^II Università degli Studi di Napoli, Napoli, Italy; ^8^Policlinico Gemelli, Roma, Italy; ^9^Ospedale Salus Pueri, Padova, Italy; ^10^Ospedale Federico II, Napoli, Italy; ^11^Ospedale Meyer, Firenze, Italy

###### **Presenting author:** Romina Gallizzi

**Introduction:** Behçet’s disease (BD) most often affects young adults, but occasionally can have its onset in childhood. Large series describing the disease in the pediatric age are scarce.

**Objectives:** Aim of our study was to collect information on clinical characteristics and treatment in pediatric patients with BD in Italy.

**Methods:** Demographic, clinical and therapy data from pediatric patients with BD, enrolled in the Eurofever registry by Italian Pediatric Rheumatology Centers, have been analyzed. Patients enrolled met the international criteria (Lancet 1990) or were diagnosed by specialists as affected by Behçet’s disease. In the latter case the diagnosis was confirmed by a panel of expert who evaluated the clinical history.

**Results:** 129 patients were included in our study: 73 were males and 56 females. In about half of cases (n = 64) a follow-up visit was also recorded, in addition to the baseline. Ethnicity was Caucasian for almost all (125/129). Mean age at disease onset was 9 years, mean age at diagnosis 13 years. A positive family history of BD was reported in 14 cases. At the baseline visit 94.3% had muco-cutaneous symptoms; 41.5% ocular involvement; 35.9% musculoskeletal symptoms; 34.8% gastro-intestinal manifestations; 31.4% constitutional symptoms; 23.5% neurologic involvement. The most common muco-cutaneous symptoms were recurrent oral aphtosis (93%); genital ulcers (27%), pseudo-follicolitis (17%), maculopapular rash (16%), erythema nodosum (13%), acneic or papulo-pustular lesions (12% each). Pathergy test was positive in 9 patients, negative in 68, not done in 7. Ocular involvement occurred in 37 patients: 14 had anterior uveitis, 4 posterior uveitis 5 panuveitis, 8 retinal vasculitis, 5 papilledema, 5 papillitis, 3 episcleritis, 1 band keratopathy and keratitis. The most common musculoskeletal symptom was arthralgia (n = 30), followed by myalgia (n = 16), oligoarthritis (n = 6), polyarthritis (n = 5), and monoarthritis (n = 2). Abdominal pain (n = 30) and diarrhea (n = 11) were the most common gastrointestinal symptoms, followed by GI ulcers (n = 4), and anal ulcers (n = 2); 5 patients had gastrointestinal bleeding, one patient presented aseptic peritonitis and 2 patients gut perforation. Constitutional symptoms included recurrent fever in 22 patients, fatigue and malaise in 14. Headache was the most common neurologic symptom (n = 17); 7 patients had cranial nerve palsies, 3 presented vertigo, 1 optic neuritis and 1 aseptic meningitis. Moreover, 1 patient had ataxia and 1 presented hemiplegia and abnormal behavior. Venous thrombosis occurred in 3 patients (thrombosis of transverse sinus in one of them). Two patients also had genitourinary involvement (urethritis or cystitis). HLA-B51 was present in 39 patients, not done in 12. The main treatment used was systemic corticosteroids, followed by colchicine (n = 31) and other immunosuppressants, ie azathioprine (n = 6), metothrexate (n = 5), cyclosporine (n = 3), thalidomide (n = 2), and cyclophosphamide (n = 1). Infliximab was also used in one patient. During follow-up, other biologic agents were also used, ie Adalimumab (n = 9) and Anakinra (n = 1).

**Conclusion:** This is one of the largest pediatric BD cohorts reported so far. Our data are similar to those of other pediatric series. The performance of the new Ped-BD criteria in our series is currently being evaluated, as well as possible correlations between clinical signs or symptoms at onset with immunosuppressive treatment.

**Disclosure of Interest**

None Declared

### P127 Endothelial dysfunction in children with Kawasaki disease and transient coronary artery abnormalities

#### Man S. Parihar^1^, Surjit Singh^1^, Pandiarajan Vignesh^1^, Anju Gupta^1^, ManojKumar Rohit^2^

##### ^1^Allergy Immunology Unit, Advanced Pediatrics Centre, Chandigarh, India; ^2^Cardiology, Post Graduate Institute of Medical Education and Research, Chandigarh, India

###### **Presenting author:** Surjit Singh

**Introduction:** Evidence of premature atherosclerosis and systemic arterial stiffening in follow up patients of Kawasaki disease (KD) and coronary artery abnormalities is accumulating. Diminished flow-mediated dilatation (FMD) and increased carotid artery stiffness index (SI) have been used as surrogate markers of premature arteriosclerosis. Both of these indices have been shown to correlate with late cardiovascular events and/or mortality in adults. Because of their noninvasive nature these parameters have recently been studied in children as well. There is paucity of studies in endothelial dysfunction in children with KD and transient coronary artery abnormalities.

**Objectives:** In the present study, we assessed the risk of atherosclerosis by noninvasive methods (FMD, cIMT and carotid artery stiffness index) in children of KD with transient coronary artery abnormalities at least 1 year after resolution CAA of KD.

**Methods:** Twenty children with KD (mean age 11.5 ± 3.7 years) and transient coronary artery dilatation which had resolved within 1 year of diagnosis were studied at least 1 year of after resolution of CAA. All had received intravenous immunoglobulin except one during the acute phase of the disease. High-resolution ultrasonography was used to analyze brachial artery responses to reactive hyperemia and sublingual nitroglycerine. Flow-mediated dilatation was also studied in an equal number of healthy age- and sex-matched controls. Carotid artery intima-media thickness and stiffness index was calculated using B mode ultrasound and compared in all subjects using previously published equations.

**Results:** No statistically significant difference was noted in the percent flow mediated dilatation of the brachial arteries in response to reactive hyperemia among the cases (13.31 ± 10.41%) compared with controls (12.86 ± 7.09%; p = 0.874). Sublingual nitroglycerine-mediated dilatation in children with KD was 14.88 ± 12.03%. Mean carotid intima-media thickness was found to be almost same in both cases (0.036 ± 0.015 cms) and controls (0.035 ± 0.076 cms; p = 0.791). Also, no significant difference was noted in the carotid artery stiffness index among the cases (1.153 ± 0.34 U) when compared to controls (1.11 ± 0.40 U; p = 0.729).

**Conclusion:** No evidence of endothelial dysfunction in KD patients with transient coronary artery abnormalities was noted in the present study. This may point towards no risk of preclinical arteriosclerosis in children with KD with transient coronary artery abnormalities. However, as the number of cases were small, it would be imprudent to extrapolate these results and draw conclusions based on the small sample size.

**Disclosure of Interest**

None Declared

### P128 Carotid intima-media thickness and lipid profile in children with Kawasaki disease: a follow-up study after 5 years

#### Kavitha Gopalan^1^, Surjit Singh^1^, Pandiarajan Vignesh^1^, Anju Gupta^1^, ManojKumar Rohit^2^, Savita V. Attri^3^

##### ^1^Allergy immunology Unit, Advanced Pediatrics Centre, Post Graduate Institute of Medical Education and Research, Chandigarh, India; ^2^Cardiology, Post Graduate Institute of Medical Education and Research, Chandigarh, India; ^3^Division of Biochemistry, Advanced Pediatrics Centre, Chandigarh, India

###### **Presenting author:** Surjit Singh

**Introduction:** Kawasaki disease (KD) is the most common medium vessel vasculitis in children. It has a predilection to involve coronary arteries, leading to several long term cardiovascular sequelae. In addition to coronary artery abnormalities (CAAs), premature atherosclerosis, endothelial dysfunction and lipid abnormalities are seen in children with KD. Despite prompt treatment with intravenous immunoglobulin in the acute phase, these complications ensue.

**Objectives:** At our institute in Chandigarh, Meena et al. in 2009 studied carotid intima media thickness (cIMT) and lipid profile in 27 children with KD after 1 year of the acute episode of KD. This study was planned to follow-up the same cohort of children at least 5 years after the acute episode.

**Methods:** All the 27 children previously enrolled by Meena et al. were followed-up in the present study. cIMT, being a surrogate marker for premature atherosclerosis and fasting lipid profile were evaluated. Diagnosis of KD was based on the American Heart Association criteria. All patients had received 2 gm/kg of IVIg at the time of diagnosis. Age and sex matched healthy children were enrolled as controls from the out-patient department of the institute for cIMT. For lipid profile, comparison was made with published data on this subject. cIMT was measured in the Echocardiography lab of Advanced Cardiac Centre, PGIMER by Acuson Sequoia C- 512 Siemens Limited with 7-12 MHz linear array transducer. All children with KD were asked to fast for 8 hours overnight and 2 ml blood sample was collected. Total cholesterol, HDLC, triglycerides were estimated using standard laboratory kits manufactured by Siemens Healthcare Diagnostics. Cholesterol levels < 170 mg/dL were considered desirable and > 200 mg/dL were considered undesirable. Cholesterol levels in between these limits were considered borderline. Low-density lipoprotein cholesterol (LDL-C) levels < 110 mg/dL were considered desirable, and > 130 mg/dL were considered undesirable. LDL-C levels in between these limits were considered borderline. High-density lipoprotein cholesterol (HDL-C) levels > 35 mg/dL were considered desirable, and < 35 mg/dL were considered undesirable. For triglycerides, levels < 150 mg/dL were considered desirable and > 150 mg/dL as undesirable.

**Results:** Mean duration between onset of fever and IVIg treatment was 14.3 days with a range of one to 90 days. At the time of diagnosis, 10 patients (37.03%) had CAAs in the form of dilatation. There was significantly higher mean cIMT in children with KD (0.54 ± 0.087 mm) as compared to controls (0.42 ± 0.036 mm), however there was no significant difference in cIMT at 1 and 5 years of follow-up. There was no statistically significant difference among cholesterol, HDLC, LDL-C and triglyceride levels between cases with CAAs at diagnosis as compared to children without CAAs at diagnosis. Abnormal lipid profile was seen in 7 out of 27 children in the present study (1 child- borderline serum cholesterol levels, 4 children- undesirable serum HDL-C levels, and 2 children- undesirable serum triglyceride levels), 5 of whom had lipid abnormality at 1 year follow-up (Meena et al.), suggesting that lipid profile abnormalities in KD are perhaps persistent and long lasting.

**Conclusion:** Our findings thus presage fertile ground for cardiovascular complications in young adulthood in patients with KD. It is likely that the consequences of KD in childhood would impact the health status of young adults several years later.

**Disclosure of Interest**

None Declared

### P129 Microparticles as a therapeutic target for plasma exchange in antineutrophil cytoplasmic antibody associated vasculitis?

#### Ying Hong^1^, Despina Eleftheriou^1^, Sira Nanthapisal^1^, Alan Salama^2^, David Jayne^3^, Mark Little^4^, Paul Brogan^1^

##### ^1^Infection, Immunity, Inflammation and Physiological Medicine, Institute of Child Health, UCL, London, UK; ^2^UCL Centre for Nephrology, Royal Free Hospital, London, UK; ^3^Vasculitis and Lupus Clinic, Addenbrookes Hospital, Cambridge, UK; ^4^School of Medicine, Trinity College Dublin, Dublin, United Kingdom

###### **Presenting author:** Ying Hong

**Introduction:** Plasma exchange (PE) is a renal function preserving and potentially life-saving therapy in children and adults with anti-neutrophil cytoplasmic antibody (ANCA) associated vasculitis (AAV). The therapeutic mechanisms of PE in AAV are poorly understood, but it is likely that PE removes autoantibodies and other plasma constituents involved in AAV. We have previously shown that circulating neutrophil microparticles (MP) are key inflammatory mediators underpinning the pathogenesis of AAV.

**Objectives:** The aim of this project was to investigate whether MP are removed from the circulation of patients with AAV by PE, and therefore are potentially an important therapeutic target. This was an ancillary study of a major international clinical trial of therapeutic PE in AAV, the PEXIVAS trial (NCT #NCT00987389).

**Methods:** Patients with AAV undergoing PE for active disease measured by the Birmingham Vasculitis Activity Score (BVAS) were recruited from the Royal Free Hospital and Great Ormond Street Hospital NHS Foundation Trust. Annexin V+ MPs were isolated from platelet poor plasma, cellular origin defined (neutrophil, platelet, endothelial) and MP quantified using flow cytometry. The soluble inflammatory mediators in EDTA plasma were measured using multi-array electrochemiluminescence (MSD). Results were expressed as median and range.

**Results:** A total of seven patients undergoing PE at diagnosis of AAV were recruited: median age 64 years (range 17-22 years old); 4 male. Median BVAS was 15/63 (12-18/63); all patients studied had rapidly progressive glomerulonephritis. The number of total circulating AnV + MP reduced by ~60% after 3 cycles of PE from a median of 5.1 × 10^6^ /ml (range of 11.2 – 4.78 × 10^6^ /ml) to a median 1.97 × 10^6^/ml (0.36 × 10^6^ – 3.7 × 10^6^/ml), *p = 0.04*. Neutrophil MP identified as CD66b^+^AnV^+^ were reduced from a median of 58 × 10^3^ /ml (range of 88 × 10^3^ – 40 × 10^3^/ml) to a median of 15 × 10^3^/ml (range of 17 × 10^3^ – 11 × 10^3^/ml), *p = 0.02.* Angiogenic factors (VEGF and Ang-1) were lower in patients with active AAV, comparing with health control, but no change after PE. In patients with active AAV, significantly increased level of Ang-2 was detected, comparing with healthy control, furthermore, levels of Ang-II declined from a median of 8179 pg/ml (range of 3440-17313 pg/ml) to median of 3776 pg/ml (range of 775-12143 pg/ml), *p* = 0.03, similar to normal values (median of 2747 pg/ml, range of 1063-9296 pg/ml). Similarly, the plasma level of sICAM (a soluble marker of endothelial activation) were increased in patients with active AAV, and there was a significant reduction pre and post PE, *p* = 0.01.

**Conclusion:** Our preliminary results suggest that PE is an effective method to remove these circulating mediators of inflammation and endothelial injury. It remains to be elucidated if the removal of excess injury factors is crucial to ameliorate endothelial damage in patients with AVV.

**Disclosure of Interest**

None Declared

### P130 Takaysu arteritis in children: experience in observation and treatment of a single center

#### Yulia Kostina^1^, Galina Lyskina^1^, Olga Shpitonkova^1^

##### ^1^Pediatric Department, I.M. Sechenov First Moscow State Medical University, Moscow, Russian Federation

###### **Presenting author:** Yulia Kostina

This abstract is not included here as it has already been published.

### P131 The age features of Kawasaki disease, Moscow, Russian Federation

#### Alena Torbyak, Galina Lyskina, Olga Shirinsky

##### Department of Childhood diseases, I.M.Sechenov First Moscow State Medical University, Moscow, Russian Federation

###### **Presenting author:** Alena Torbyak

**Introduction:** Kawasaki disease (KD) is a leading cause of acquired heart disease in children in developed countries mostly because of its coronary artery complications. Still there is controversial data about the influence of patient’s age on the clinical features and outcomes of KD

**Objectives:** Medical records of 168 KD aged from 1 month 13 days to 13 years 6 months treated in the University Children’s Clinical Hospital from 2003 to 2014 were reviewed. 24 (14,3%) of them were <6 months, 31 (18,4%) - 6- < 12 months, 94 (56%) - 12 months- < 5 years, 19 (11,3%) - ≥5 years. To evaluate the contribution of patient’s age to the development of coronary artery aneurysms (CAA) clinical, laboratory data and IVIG treatment were analyzed

**Methods:** Patients’ data were compared between four groups: children <6 months of age, 6- < 12 months, 12 months - <5 years, ≥5 years at the time of the disease onset. The presence of CAA was measured using 2-DE performed at 8 weeks after disease onset. Differences were tested using the Mann-Whitney test, Fisher’s exact test, Chi-square, Student's *t*-test. The odds ratios (ORs) with 95% confidence intervals (CIs) of explanatory variables for the presence of CAA were determined by multiple logistic regression models

**Results:** The high degree of statistical significance for different age groups of children with KD was revealed for the frequency of the cervical lymphadenopathy, articular and abdominal syndromes, vomiting (as a gastrointestinal damage manifestation), meningeal and urinary syndromes, erythema of the BCG area (P < 0,05). It was shown that patient’s age influenced the clinical and laboratory phenotype of KD determining the frequency of non-basic symptoms which could be very prominent and often defined an incorrect diagnosis.

CAA were found in the group of children younger 6 months of age significantly more often even if they were treated with IVIG before the 10^th^ day of illness: 60% vs 11,6-12,5% of children treated with different doses of IVIG before the 10^th^ day of illness in three other age groups (P < 0,05); 50% vs 9-11% of children treated with IVIG 2 g/kg before the 10^th^ day of illness in groups 6- < 12 months and 12 months - <5 years and 20% in children ≥5 years (P > 0,05).

**Conclusion:** Patient’s age influences the clinical phenotype of Kawasaki disease and has to be taken into account while making the diagnosis. It also influences the outcome of the disease even if a child is treated with IVIG before the 10^th^ day of illness

**Disclosure of Interest**

None Declared

### P132 Early onset of behcet disease: a case report

#### Angela Mauro^1^, Maria Francesca Gicchino^1^, Maria Cristina Smaldone^1^, Mario Diplomatico^1^, Alma Nunzia Olivieri^1^

##### ^1^Rheumatology Unit, Second University of Naples, Naples, Italy

###### **Presenting author:** Angela Mauro

**Introduction:** Behçet Disease (BD) is a chronic inflammatory disorder with multisystemic involvement. The aetiology is unknown and it is characterized by clinical features characteristic of both autoimmune and auto-inflammatory diseases.

**Objectives:** Case report of early onset BD.

**Methods:** A 13 year-old boy was admitted to our hospital to investigate the presence of recurrent oral aphthosis, acneiform and papulo-pustular skin lesions, bronchiectasis associated to recurrent respiratory infections, arthritis, and growth retardation. His family history showed recurrent oral aphthosis in his mother. He was born after 36 weeks of gestation via vaginal delivery, with APGAR score 9/10. Birth weight was 3,450 Kg. At the third month of age, oral candidiasis associated to perioral and genital papulo-pustular skin lesions appeared. After 6 months, he developed recurrent fevers (T° max 39-40 °C), monthly. At 3 years of age, the papulo-pustolar skin lesions spread to buttocks and ears and, after one year, the boy developed anterior uveitis. Since he was affected by growth delay associated to increase of fecal calprotectin, he underwent an EGDS, that showed features of autoimmune enteropathy (mucosal architectural changes including villous atrophy, increased intraepithelial lymphocytes and 10 or more eosinophils per high power field); autoantibodies (transglutaminase ab, ANA, p-ANCA, c-ANCA, anti-dsDNA, anti-Jo1, ant-Sm, anti-RNP, anti-Scl70, anticentromere and anti-SSA) were negative. At 5 years of age, genital ulcers and erythema nodosum on legs (anterior tibial regions), hands and elbow appeared.

**Results:** He was then admitted to our hospital for further investigations. On physical examination, he presented rather good general conditions, oral aphthosis, acneiform and papulo-pustular skin lesions son perioral, ears and genital regions. Blood tests revealed an increase of inflammatory markers (ERS 35 mm/1 h, CRP 4.51 mg/L, SAA 70 mg/L). Liver and renal functions were normal. Autoantibodies (ANA, p-ANCA, c-ANCA, anti-dsDNA, anti-Jo1, ant-Sm, anti-RNP, anti-Scl70, anticentromere and anti-SSA) were performed as well, and resulted all negative except for ANA (1:1280). Genetic tests for MEFV, MVK, CIAS-1 and TMEM173 were negative. HLA-B51 was positive Cerebral MRI, MR angiography and echocardiography were performed, and resulted normal. He started Colchicine (1 mg/die), topical corticosteroids, antimicrobial prophylaxis and airway clearance techniques.

**Conclusion:** The diagnosis was confimed according to the criteria of the “International Study Group for the Diagnosis of Behçet Disease” (Recurrent oral aphthosis, genital ulceration, skin involvement, anterior uveitis). Looking at the early onset of disease and the positive family history we have also considered the monogenic disease with A20 haploinsufficiency, recently described and caused by high-penetrance germline mutations in TNFAIP3, which encodes the NF-kB regulatory protein A20, leading to a Behcet-like disease with early-onset systemic inflammation. Genetic test results are pending.

**Disclosure of Interest**

None Declared

### P133 A quality improvement initiative to improve urinalysis follow-up in henoch-schonlein purpura: retrospective review and prospective intervention

#### C H. Spencer^1^, Rabheh Abdul Aziz^2^, Richard McClead^2^, Sharon Bout-Tabaku^2^, Hiren Patel^3^, Chung-Yung Yu^2^

##### ^1^Rheumatology, Nationwide Childrens/Ohio State, Columbus OH, USA; ^2^Rheumatology, Nationwide Childrens/Ohio State, Columbus, United States; ^3^Nephrology, Nationwide Childrens/Ohio State, Columbus, United States

###### **Presenting author:** C H. Spencer

**Introduction:**

Henoch-Schonlein purpura (HSP) is the most common systemic vasculitis of childhood with an incidence of 10–20 cases per 100,000 children per year. Generally the prognosis is good, with exception of those with significant renal involvement. The principal aim of HSP follow-up is mostly to detect persistent renal inflammation that could progress to renal damage. It was our observation that the weak link in the care of these children at our institution was urinalysis (UA) follow-up after the first patient contact with the Nationwide Children’s Hospital (NCH) medical system. In the long-term, the HSP renal disease may be serious in these children and a simple cost-effective intervention may detect HSP renal disease earlier in the disease process and improve outcomes.

**Objectives:** This quality improvement (QI) project’s overall objective was to design an intervention that might improve the follow-up with UA screenings by educating families and asking care providers to do the urinalyses on schedule. The specific aim was to increase the performance of follow-up urinalyses from baseline by 21% over 6 months and by 30% over 12 months by use of this QI intervention.

**Methods:** Approval for this study was obtained from the Institutional Review Board of NCH in 2014. We identified HSP patients in our electronic medical record (EMR) EPIC using the US codes ICD 9 and ICD 10 for HSP. 105 HSP patients were identified retrospectively who had a diagnosis of HSP from July 1, 2014 to December 31, 2014. Their records were reviewed for diagnosis, date of diagnosis, location of clinic visit, new or old HSP diagnosis, dates and results of urinalyses at diagnosis and on follow-up, and diagnosis of renal disease by nephrologists and results of kidney biopsy. Similarly patients were identified and charts reviewed prospectively from January 1, 2015 to December 31, 2015 for the same data. The review of the 2014 charts helped identify the QI key drivers of provider factors, process factors, and patient factors that informed the intervention. The authors agreed on a consensus schedule for follow-up UA’s. A dot phrase was developed and inserted into the EMR of NCH which contained education about HSP, the rationale for the follow-up urinalyses, and our suggested schedule. The availability of the dot-phrase was advertised widely to all care providers in the NCH system before January 1, 2015. The data was collected month to month in 2015 on 239 HSP patients and UA results obtained from the EMR and primary care providers.

**Results:** Follow-up urinalysis performance for the HSP patients in 2014 was 21%. The AVS was given out to families in 2015 at rates from 75-95% in different outpatient sites. With the intervention during 2015, the urinalyses were performed at the rate of 61% from January 1 to June 30^th^ and 55% over the second 6 months. The changes were statistically significant (for mean change p = .0075). Four children initially had a normal urinalysis at their first visit and then developed an abnormal UA; 3 patients’ abnormal UA resolved over the next 8 months but one developed chronic renal disease. Overall, 2 children developed chronic renal disease with IgA nephropathy on kidney biopsy.

**Conclusion:** Our QI intervention was successful in achieving our goal of increasing follow-up urinalysis performance in HSP patients seen at HSP in 2015. We believe such an inexpensive QI intervention is beneficial to the HSP patients but further study in a collaborative network is needed to determine if it actually detects renal disease earlier and improves outcomes.

**Disclosure of Interest**

None Declared

### P134 Successful treatment of digital necrotizing vasculitis in patients with polyarteritis nodosa

#### Coskun F. Ozkececi^1^, Gokalp Basbozkurt^2^, Dogan Simsek^2^, Esra Kurt^2^, Faysal Gok^2^, Erkan Demirkaya^2^

##### ^1^Pediatrics, Gulhane Military Medical Academy, Ankara, Turkey; ^2^Pediatric, Gulhane Military Medical Academy, Ankara, Turkey

###### **Presenting author:** Coskun F. Ozkececi

**Introduction:** Polyarteritis nodosa is a rare necrotizing vasculitis which affects small and medium-sized arteries and characterized by multi- organ involvement. Gangrene digitale and extremity loss via necrotising vasculitis have been reported in polyarteritis nodosa. Although immunosuppressive agents, vasodilatators, anticoagulants are used, there is no definitive treatment protocol.

**Objectives:** .

**Methods:** A 15 year old male was admitted to our hospital with paleness, redness, bruising on both the fingers, toes and redness on the cheeks. The complaints of paleness, coldness, redness at fingertips began five days before admission to hospital. In a few days bruising, more prominently on toes, appeared on fingers and toes. Anamnesis and the family history was not significant. In the first examination, vital signs were stabile, growth and development were compatible with the age, 2^nd^ 3^rd^ and 4^th^ fingertips of left foot’s color were black and necrotising. Capillary refilling time was apparently prolonged. Laboratory analyses showed WBC of 15,040/mm^3^(5,000-14,500), hemoglobin of 14 gr/dL (12-16), platelet of 387,000/mm^3^ (150,000-450,000), erythrocyte sedimentation rate (ESR) of 86 mm/h (0-20), C-reactive protein (CRP) of 102 mg/L (0-6), antistreptolysin O (ASO) of 3280 IU/mL (0-200), aPTT of 34,1 sec (26-38), protrombine time 12,7 sec (11-14), INR of 1,09 (0,8-1,2), D-dimer 2,25 mg/L (0-0,55). Urinalysis, creatinin levels, serum levels of transaminase were normal and anti-HIV, anti-HAV, anti-HBV, and anti-HCV antibodies were negative. Anti-nuclear antibody (ANA) titre was 1/100. Anti-ds DNA, profile of Anti ENA, p-ANCA, c-ANCA, Anti cardiolipin IgM and IgG antibodies, lupus anticoagulant and crioglobulin were negative. C3, C4, Protein C, Protein S, antitombine III, vWF, homocysteine levels were normal. FVIII and FIX levels were increased. Enhanced CT angiography demonstrated narrowing and contour irregularities at left kidney, mesenteric and both tibialis posterior arteries. PA lung and sinus graphies were normal. Patient was screened for MEFV mutation and homozygous R202Q/R202Q and M694V/N were detected. The patient was homozygous for MTHFR C677T. With these findings, patients was diagnosed as poliarteritis nodosa (PAN). Methylprednisolone 1gr/day was given in three consecutive days and 1gr/month cyclophosphamide was started. After these treatment, 60 mg/day orally prednisolone therapy and simultaneously, 30 sessions of hyperbaric oxygene therapy was applied. Treatment of the first five days of hyperbaric oxygene therapy, ilioprost (0,5 ng/kg/min) was given with IV infusion. Low molecular weight heparin (enoxaparin) (2 mg/kg/day) was administrated for 6 months. Mycophenolate mofetil (1200 mg/m^2^/day) was added to therapy at the end of the cyclophosphamide pulse. After 30 sessions of hyperbaric oxygene therapy, the patient was discharged.

**Results:**

**Conclusion:** Vasculitis which can lead to loss of extremity, hyperbaric oxygene therapy that might be considered as a concomitant treatment with drugs has an important contribution to the preservation of tissue and extremity.

**Disclosure of Interest**

None Declared

### P135 Paediatric large vessel vasculitis presenting as idiopathic retroperitoneal fibrosis: case report and review of the literature

#### Dita Cebecauerová^1^, Tomáš Dallos^2^, Edita Kabíčková^3^, Martin Kynčl^4^, Daniela Chroustová^5^, Jozef Hoza^1^, Dana Němcová^1^, Vladimír Tesař^6^, Pavla Doležalová^1^

##### ^1^Department of Pediatrics and Adolescent Medicine, First Faculty of Medicine, Charles University and General University Hospital, Prague, Czech Republic; ^2^Pediatric Rheumatology Service, 2nd Department of Pediatrics, Comenius University Medical School and University Children’s Hospital, Bratislava, Slovakia; ^3^Department of Pediatric Hematology and Oncology, Charles University and University Hospital Motol, Prague, Czech Republic; ^4^Department of Radiology, Second Faculty of Medicine and University Hospital Motol, Prague, Czech Republic; ^5^Department of Nuclear Medicine, First Faculty of Medicine, Charles University and General University Hospital, Prague, Czech Republic; ^6^Department of Nephrology, First Faculty of Medicine, Charles University and General University Hospital, Prague, Czech Republic

###### **Presenting author:** Dita Cebecauerová

**Introduction:** Idiopathic retroperitoneal fibrosis (RPF) is a rare disease characterised by development of fibro-inflammatory mass in retroperitoneum. RPF is very rare among children and is considered a subtype of chronic periaortitis that might be associated with large-vessel vasculitis or other autoimmune disorders.

**Objectives:** To analyse an unusual case of retroperitoneal fibrosis in a teenage girl progressing towards predominantly large vessel vasculitis and to review existing paediatric literature.

**Methods:** Case report and literature review

**Results:** A 13-year-old previously healthy Caucasian girl presented with recurrent fever, elevated inflammatory markers and back pain that initially mimicked pyelonephritis. Recurrence of symptoms and later development of supraclavicular lymphadenopathy promoted imaging (ultrasound US, MRI) which confirmed presence of large lymph node (LN) packet with inflammatory rather than malignant appearance on PET-CT. Transient partial spontaneous regression of her symptoms as well as non-specific inflammatory histopathology changes of her cervical LN supported conservative watch-and-wait approach by the paediatric oncology team. The third relapse with systemic hypertension as an additional new symptom led to the open biopsy from the retroperitoneal mass which excluded lymphoma. Repeated MRI revealed new involvement of aorta at the level of renal arteries and supported suspicion of vasculitis. Review of the history, imaging and biopsy results led to the diagnosis of RPF with progression to secondary vasculitis. General symptoms improved on corticosteroid (CS) therapy, but systemic hypertension as a consequence of the right renal artery occlusion progressed and resulted in nephrectomy. Aggressive immunosuppression with CS and i.v. cyclophosphamide did not abate the process which eventually affected abdominal aorta, iliac, renal and pulmonary arteries. Post-contrast gadolinium enhancement was noticed in the wall of these large vessels corresponding to persistent inflammatory activity despite the disappearance of the fibrous/LN retroperitoneal mass. After introduction of rituximab and later also low-dose weekly methotrexate (MTX) disease activity slowly diminished. After 18 months of this therapy she has been able to reduce her CS dose to 0,1 mg/kg/day and is doing well clinically, laboratory and imaging-wise. Literature search of the PubMed database from 2003 revealed 31 cases of RPF in children with typical onset age between 10 and 15 years, but none of them presented with arterioocclusive process. Around half of the patients presented with an associated autoimmune condition, and in two cases the association with malignancy was described. Majority of patients with RPF responded well to CS therapy, in refractory disease B cell-depleting therapy with rituximab was successful.

**Conclusion:** We present an unusual paediatric case of an overlap between RPF and large vessel vasculitis. This case is in accordance with the published adult case series demonstrating that chronic periaortitis as a component of RPF is not limited to the abdominal aorta and displays the features of primary large vessel inflammatory disease. Our choice of initial cytotoxic therapy was driven by the severe clinical picture. We have learnt that rituximab time to clinical effect may by as long as 18 months and that combination with MTX has brought additional benefit. Despite that, the long-term prognosis is warranted.

**Disclosure of Interest**

D. Cebecauerová: None Declared, T. Dallos: None Declared, E. Kabíčková: None Declared, M. Kynčl: None Declared, D. Chroustová: None Declared, J. Hoza: None Declared, D. Němcová: None Declared, V. Tesař: None Declared, P. Doležalová Grant / Research Support from: AbbVie, Roche, Medac, Novartis, Pfizer, Consultant for: Roche, Speaker Bureau of: Pfizer, Novartis, Medac

### P136 Tocilizumab treatment in childhood Takayasu arteritis: case series of four patients and systematic review of the literature

#### Ezgi Deniz Batu^1^, Hafize Emine Sonmez^1^, Tuncay Hazirolan^2^, Fatih Ozaltin^3^, Yelda Bilginer^1^, Seza Ozen^1^

##### ^1^Department of Pediatrics, Division of Rheumatology, Hacettepe University Faculty of Medicine, Ankara, Turkey; ^2^Department of Radiology, Hacettepe University Faculty of Medicine, Ankara, Turkey; ^3^Department of Pediatrics, Division of Nephrology, Hacettepe University Faculty of Medicine, Ankara, Turkey

###### **Presenting author:** Ezgi Deniz Batu

**Introduction:** The mainstay of treatment for Takayasu arteritis (TAK) is high-dose corticosteroid. However, the addition of immunosuppressive agents such as methotrexate and cyclophosphamide is usually needed. Besides, several studies have shown favorable effect and good tolerance of tocilizumab (anti-interleukin 6) treatment in TAK patients.

**Objectives:** Our aim was to describe our experience with tocilizumab treatment in children with TAK and to review previous studies regarding tocilizumab use in TAK patients.

**Methods:** We reviewed the charts of all pediatric TAK patients followed between 2000 and 2015 in Department of Pediatric Rheumatology in Hacettepe University, Ankara, Turkey and we present the patients who were treated with tocilizumab. We screened PubMed and MEDLINE for articles involving TAK patients treated with tocilizumab.

**Results:** We have followed 11 pediatric TAK patients between 2000 and 2015. Four of them (36.3%) received tocilizumab. All four patients were female. The characteristics of these patients were summarized in Table 1. The median (minimum-maximum) age at diagnosis was 14.5 (4-16) years and the median duration of disease was 30 (7-68) months. The median duration of immunosuppressive treatment before tocilizumab onset was 16 (1-60) months. The median duration of tocilizumab treatment and the median duration of follow-up after the first dose of tocilizumab was both 9.5 (7-13) months since all four patients continue to receive tocilizumab. One of our patients received tocilizumab as a first line immunosuppressive treatment directly after one dose of pulse methylprednisolone because the family refused cyclophosphamide. Others were resistant to their initial immunosuppressive treatment (cyclophosphamide, methotrexate, azathioprine). All four of our patients achieved complete response to tocilizumab after three months of treatment. Repeated MRAs showed no new vascular lesions in our patients. ITAS2010-A values were decreased from a median of 11.5 (1-15) to 0.5 (0-1) after three months of treatment with tocilizumab. None of the patients reported any adverse events during follow-up. We identified 19 articles describing 75 TAK patients treated with tocilizumab during our literature search. Twelve patients received tocilizumab before age of 18 years including ours. Tocilizumab was given as the first line immunosuppressive treatment in seven patients including two children (one from our series). 11 out of 75 patients experienced relapse during tocilizumab treatment, while eight flared 2-14 months later than the withdrawal of tocilizumab.

**Conclusion:** Our small series suggests that tocilizumab may be a promising alternative for Takayasu arteritis treatment. Long-term controlled studies are warranted to provide better evidence for tocilizumab treatment in childhood Takayasu arteritis.

**Disclosure of Interest**

None Declared

### P137 Neurological manifestation in Behçet’s disease- pediatric case report

#### Fabiola Almeida^1^, Isabela H. Faria de Paula^1^, Maíra M. Sampaio^2^, Fernando N. Arita^2^, Andressa G. Alves^1^, Maria Carolina Santos^1^, Eunice M. Okuda^1^, Silvana B. Sacchetti^1^

##### ^1^Pediatric Rheumatology, Irmandade da Santa Casa de Misericórdia de São Paulo- Brasil, São Paulo, Brazil; ^2^Pediatric Neurology, Irmandade da Santa Casa de Misericórdia de São Paulo- Brasil, São Paulo, Brazil

###### **Presenting author:** Fabiola Almeida

**Introduction:** Behçet’s disease (BD) is a chronic multisystemic vasculitis that can affect arteries or/ and veins of any size and is characterized by triple –symptom complex of recurrent oral and/ or genital aphthous ulcers and uveitis. It can also involve gastrointestinal tract, pulmonary, musculoskeletal, cardiovascular and neurological systems (neuro- Behçet). Neurological involvement is one of the most devastating manifestations, with rare reports in pediatric age range

**Objectives:** Report a case of pediatric neuro- Behçet, emphasizing the importance of including vasculitis in the differential diagnosis when neurological disorders are associated with systemic manifestations.

**Methods:** Chart review, checking medical history, laboratory tests and imaging of a child with Behcet’s disease, according to the criteria of the International Study Group (ISG). A informed consent form was signed.

**Results:** M.E.S., four years old, female, admitted with headache associated with dilatation of the veins on the forehead *(capitus medusa*), erythema nodosum and history of recurrent oral ulcers. Added to this, it was observed bilateral papilledema and thrombosis of the superior sagittal sinus extending to the left transverse sinus, suggesting a diagnosis of Behçet’disease. Corticosteroid and anticoagulation were introduced. The patient progressed with completed resolution of the signs and symptoms.

**Conclusion:** Behçet’s disease should be always considered in the presence of neurological manifestations associated with systemic involvement, avoiding permanent sequelae that affect quality of life.

References

1. FulvioParentin; Loredana Lepore;Ingrid Rabah et al. Paediatric Behçet’s disease presenting with recurrent papillitis and episcleritis: a case report. Journal of Medical Case Reports 2011 5:81

2. Petty RE LaxerRM, Lindsley CB (eds). Behçet’s disease and related vasculitides. In: Textbook of Pediatric Rheumatology, 7^th^ edn. Elsevier Saunders, Philadelphia, p526–532

**Disclosure of Interest**

None Declared

### P138 Vitamin d receptor polymorphisms are not associated with the risk of Kawasaki disease (KD) in a group of Italian children

#### Fernanda Falcini^1^, Marini Francesca^2^, Stefano Stagi^3^, Donato Rigante^4^, Gemma Lepri^5^, Marco Matucci-Cerinic^1^, Maria Luisa Brandi^2^

##### ^1^Dept. of Internal Medicine, Section of Rheumatology, Transition Clinic; ^2^Dept. of Surgery and Translational Medicine; ^3^Health Sciences Department, Anna Meyer Children’s University Hospital, University of Florence, Florence, Italy; ^4^Institute of Pediatrics, Università Cattolica Sacro Cuore Universityy of Rome, Rome, Italy; ^5^Dept. of Internal Medicine, Section of Rheumatology, Transition Clinic, University of Florence, Florence, Italy

###### **Presenting author:** Fernanda Falcini

**Introduction:** Kawasaki disease (KD) is an acute multisystemic vasculitis, affecting primarily infants and children, and it is the most common cause of acquired heart disease in Western countries. The exact pathogenesis of KD has not been clearly identified, but infections caused by one or more pathogens are strongly suspected as major cause of the disease. Our previous study demonstrated that KD patients have highly reduced 25(OH)-vitamin D levels in comparison with healthy controls and also with other immunological disorders, suggesting a hypothetical role of vitamin D deficiency in the development of coronary artery damage in KD, and low vitamin D levels might contribute to the severity of chronic vascular abnormalities. Specific polymorphisms of vitamin D receptor (*VDR*) gene have recently been associated with different biologic response to vitamin D itself.

**Objectives:** 1. To analyze the possible associations of these polymorphisms in a series of Italian children affected by KD. 2. To evaluate a possible influence of VDR polymorphism in enhancing the risk of the disease.

**Methods:** We recruited 50 Italian infants and children affected by typical KD (38 males, 12 females, age ranging from 2 through to 62 months. Genomic DNA was extracted from peripheral blood leukocytes to analyze *VDR* polymorphisms by PCR-based sequencing (*CDX2* in the promoter region) and PCR-based enzymatic digestions (*FokI* in exon 2*, BsmI* and *ApaI* in intron 8, and *TaqI* in exon 9). An Italian population of 2221 unrelated individuals without KD or other immunological disorders was used as healthy controls. The distribution of polymorphism genotypes and alleles was evaluated in patients vs healthy controls.

**Results:** The distribution of all Cdx2, *FokI, BsmI, ApaI*, and *TaqI* polymorphisms (both as genotypes or alleles) did not show significant differences between affected subjects and healthy controls.

**Conclusion:** In conclusion, this pilot study evidence that *VDR* polymorphisms seem not to have any influence in determining the risk of KD. Anyway, future larger studies are needed to unravel the exact relationship between *VDR* genotype and KD development and/or clinical characteristics.

**Disclosure of Interest**

None Declared

### P139 The proteiform manifestations of Behçet disease. Description of 2 cases with prominent gastrointestinal and renal involvement

#### Giancarla Di Landro^1^, Sofia Torreggiani^1^, Antonella Petaccia^1^, Marta Torcoletti^1^, Fabrizia Corona^1^, Giovanni Filocamo^1^

##### ^1^Dipartimento della Donna, del Bambino o del Neonato, Fondazione Irccs Cà Granda Ospedale Maggiore Policlinico, Milano, Italy

###### **Presenting author:** Giancarla Di Landro

**Introduction:** Behçet’s disease (BD) is a chronic, relapsing, systemic vasculitis of unknown etiology with the clinical features of mucocutaneous lesions, ocular, vascular, articular, gastrointestinal, urogenital, pulmonary, and neurologic involvement. The clinical presentation of the disease can be very challenging.

**Objectives:** We reported two children with Behçet disease presenting with atypical presentations, abrupt onset of enterorrhagia and severe glomerulonephritis respectively.

**Methods:** We analyzed retrospectively the clinical characteristics and manifestations in two patients diagnosed with Behçet Disease according to the International Criteria for BD (ICBD) of 2013 and the EULAR/PRINTO/PRES classification for vasculitis of 2006 associated to the Chapel Hill Consensus Conference on Nomenclature of Systemic Vasculitis of 2012

**Results:** Case 1) 12 years old boy previously hospitalized in another Center because, at the age of 10, he presented with arthritis. Initially treated with oral steroids and methotrexate, he progressively presented episcleritis, recurrent oral ulcers, trombophlebitis and pathergy test positivity. The diagnosis of Behçet disease was made and he was started on colchicine with partial remission of symptoms. After 6 months he developed severe glomerulonephritis, with hematuria, nephrotic proteinuria and systemic hypertension. Blood examinations demonstrated the presence of c-ANCA positivity and lack of HLA-B51. Renal biopsy evidenced endo and extracapillary severe proliferative glomerulonephritis. The presence of moderate-high level of C3 at immunofluorescence induced to rule out the possibility of an anti-neutrophil cytoplasmic antibody (ANCA)-associated vasculitis, where the complement system was considered not to be involved since immunoglobulin deposition is generally absent in the lesions. He was treated successfully with cyclophosphamide and steroid, even if the proteinuria persists with partial reduced renal functionality.

Case 2) A 7 years old boy with unremarkable history, presented with persisting fever for 20 days, haematochezia, urticarial rash, oral ulcers and photophobia. Ophthalmologic examination revealed the presence of bilateral uveitis and papilloedema. Magnetic Resonance of brain resulted normal, colon endoscopy evidenced the presence of multiple lesions (abscesses). Thoracic X ray evidenced the presence of suspected pneumonia, persisting after prolonged antibiotic treatment. CT scan evidenced the presence of pulmonary micro nodules. The positivity for HLA B51 let us to formulate the diagnosis of Behçet disease and he was started on steroids and cyclosporine, with prompt remission of symptoms. Cyclosporin was later stopped and replaced with azathioprine. The patient is still in remission

**Conclusion:** Behçet disease is a rare disease with proteiform clinical presentation. Renal damage and colitis are relatively uncommon but should be always monitored in these children in order to start early with more aggressive treatment when needed.

**Disclosure of Interest**

None Declared

### P140 Assessment of relationship between IL-17 levels and oxidative stress in children with immunoglobulin a vasculitis

#### Hakan Kisaoglu^1^, Sema Misir^2,3^, Selim Demir^4^, Yuksel Aliyazicioglu^3^, Mukaddes Kalyoncu^5^

##### ^1^Department of Pediatrics, Karadeniz Technical University, Faculty of Medicine, Trabzon, Turkey; ^2^Department of Biochemistry, Cumhuriyet University Faculty of Pharmacy, Sivas, Turkey; ^3^Department of Medical Biochemistry, Karadeniz Technical University, Faculty of Medicine, Trabzon, Turkey; ^4^Department of Nutrition and Dietetics, Karadeniz Technical University, Faculty of Health Sciences, Trabzon, Turkey; ^5^Department of Pediatric Rheumatology, Karadeniz Technical University, Faculty of Medicine, Trabzon, Turkey

###### **Presenting author:** Hakan Kisaoglu

**Introduction:** Immunoglobulin A vasculitis (IgAV) is an IgA mediated small vessel vasculitis characterized by skin, gastrointestinal tract, kidney and joint involvement. Pathogenesis of IgAV is not clearly understood. Cytokines stimulate the production of chemokines by endothelial cells, attract inflammatory cells, induce the expression of cell adhesion molecules on endothelial cells and facilitate inflammatory cell adhesion to the vascular wall. In addition, activated cells of the immune system, could be responsible for tissue damage by producing reactive oxygen species.

**Objectives:** In this study, we aim to investigate the role of interleukin 17 (IL-17) and oxidative stress in the pathogenesis of IgAV and determine if there is a relationship between IL-17 and oxidative stress.

**Methods:** The patients, diagnosed with IgAV according to Ankara 2008 criteria were enrolled between July 2014 and August 2015. Patients who used steroid or nonsteroidal anti-inflammatory drugs in the last one month were excluded. Blood samples were obtained at active and remission phase of the disease. 8-hydroxy deoxy guanosine (oxidative DNA damage biomarker), 3-nitrotyrosine (protein oxidation biomarker) and IL-17 levels were measured via ELISA method. Malondialdehyte (lipid peroxidation biomarker) was measured by thiobarbituric acid reactive substances method. Total oxidant and antioxidant status levels were measured by colorimetric analysis.

**Results:** Forty-four patients, including 25 (56.8%) males and 19 (43.2%) females, aged between 1.91 and 15.41 years (mean: 7.71 years) were enrolled in this study. All patients had skin involvement, 39 (88.6%) had joint, 14 (31.8%) had gastrointestinal system and five (11.4%) had renal involvement. Disease severity score was one in 22 (50%), two in 17 (39%) and three in 5 (11%) patients.

Levels of IL-17 and TAS were significantly higher in active phase of the disease compared to in remission phase (p: 0.007 and p: 0.01, respectively). A moderate positive correlation was observed between IL-17 and TAS levels in both active and remission phase of the disease (r: 0.33; p: 0.03 and r: 0.34; p: 0.02, respectively).

Levels of 8-hydroxy deoxy guanosine in active phase of disease was significantly higher in patients with gastrointestinal system involvement (p:0.003).

**Conclusion:** These findings suggest that IL-17 might have a role in the pathogenesis of IgAV. Beside, higher TAS levels seen in active phase of the disease might be a compensatory response to oxidative stress, which is induced by inflammation. The positive correlation between IL-17 and TAS levels in both active and remission phase of the disease also supports this compensatory response.

**Disclosure of Interest**

None Declared

### P141 Associatiom between pediatric cutaneous polyarteritis nodosa and streptococcal infection - a single tertiary center experience

#### Isabela H. Faria de Paula^1^, Carlos Eduardo Ramalho^2^, Fabiola D. Almeida^1^, Andressa G. Alves^1^, Maria Carolina Santos^1^, Silvana B. Sacchetti^1^, Eunice M. Okuda^1^

##### ^1^Pediatric Rheumatology, Irmandade Santa Casa de Misericordia de São Paulo, São Paulo, Brazil; ^2^Pediatric, Irmandade Santa Casa de Misericordia de São Paulo, São Paulo, Brazil

###### **Presenting author:** Isabela H. Faria de Paula

**Introduction:** Polyarteritis nodosa (PAN) is a necrotizing vasculitis of the wall of small and medium-sized arteries. Cutaneous Polyarteritis nodosa (cPAN) is the most common in pediatric population. Its etiology is unknown, but the association with streptococcal infection has been described, which suggests the indication of antimicrobial prophylaxis similar to that made in rheumatic fever. There are few studies addressing this, what motivated this study.

**Objectives:** Aim to evaluate the association between cPAN and streptococcal infection.

**Methods:** Retrospective review of medical records of patients who met the diagnostic criteria for PAN between 1987 and June 2015. The evidence of streptococcal infection was evaluated by the increase in the antibody anti streptolysin O (ASO) at diagnosis and clinical relapse PAN.

**Results:** Fifteen patients were evaluated in this period. Two children were excluded because they did not have investigation about streptococcal infection. Thirteen patients had PAN diagnosis, three (23%) with systemic and 10 with cPAN (77%). Three patients out of four (75%) had clinical recurrence with streptococcal evidence. The number of reported cases in our service was similar to that found in the literature.

**Conclusion:** The evidence of streptococcal infection was found in patients with cPAN at diagnosis and relapse. If the association between streptococcal infection and patients with cPAN is confirmed, prophylaxis is suggested.

References

1- Fink CW. Polyarteritis and other diseases with necrotizing vasculitis in childhood. Arthritis Rheum 1977; 20:378–84.

2- S.H. Till & R.S. Amos. Long term Follow up of Juvenile Onset Cutaneous Polyarteritis Nodosa associated with Streptococcal Infection. Br J Rheumatol 1997; 36:909–1.

3- Tonnelier JM; Ansart S; Tilly-Gentric A et al. Juvenile relapsing periarteritis nodosa and streptococcal infection. JointBoneSpine 2000; 67:346–8.

4- Cassidy JT, Petty RE (2006) Polyarteritis nodosa and related vasculitides. In: Cassidy JT, Petty RE, LaxerRM, Lindsley CB (eds). Textbook of Pediatric Rheumatology, 5^th^ edn. Elsevier Saunders, Philadelphia, pp 512–520

5- Ozen S; Anton J; Arisoy N; Bakkaloglu A; Besbas N et al. Juvenile Polyarteritis: results of multicenter survey of 110 children. J Pediatrics 2004; 145:517–22.

6- Kawakami T. A Review of Pediatric Vasculitis with a focus on Juvenile Polyarteritis Nodosa. Am J Clin Dermatol 2012; 13(6):389–98.

7- Daoud MS; Hutton KP & Gibson LE. Cutaneous Periarteritis Nodosa: a clinicopathologial study of 79 cases. Br J Dermatol 1997; 136:706–13

8- Jones TD. The diagnosis of rheumatic fever. JAMA. 1944; 126: 481–4.

**Disclosure of Interest**

None Declared

### P142 Abciximab for treatment of coronary aneurysms in Kawasaki disease

#### Joan Calzada-Hernández^1^, Rosa Bou^1^, Estíbaliz Iglesias^1^, Judith Sánchez-Manubens^1^, Fredy Hermógenes Prada Martínez^2^, Clara Giménez Roca^1^, Sergi Borlan Fernández^1^, Juan Manuel Mosquera Angarita^1^, Jordi Anton^1^

##### ^1^Pediatric Rheumatology Unit, Department of Pediatrics, Hospital Sant Joan de Déu, Esplugues de Llobregat (Barcelona), Spain; ^2^Department of Pediatric Cardiology, Hospital Sant Joan de Déu, Esplugues de Llobregat (Barcelona), Spain

###### **Presenting author:** Joan Calzada-Hernández

**Introduction:** Kawasaki disease (KD) is a childhood acute vasculitis. The development of coronary damage is the main long-term consequence and can lead to myocardial infarction or sudden death. Reducing the incidence of coronary artery aneurysms (CAA) is the treatment cornerstone. Abciximab, a glycoprotein IIb/IIIa receptor antagonist, is indicated in acute coronary syndrome in adults. Some reports suggest a benefit in KD.

**Objectives:** To review the experience at our center in treating CAA with abciximab in patients with KD and determine its efficacy in reducing the CAA size.

**Methods:** Retrospective observational study describing all patients with KD and coronary involvement treated with abciximab in our center. Coronary maximum diameter is expressed in Body Surface Area (BSA)-adjusted Z-score. The coronary outcome is described and safety of treatment evaluated.

**Results:** Five patients have been treated with abciximab since 2012, all males with mean age of 11.5 ± 7.1 months. The left main coronary artery (LMCA) was constantly affected, the right coronary artery (RCA) in three patients and the left anterior descending (LAD) in two. The BSA-adjusted Z-score for the affected arteries at KD diagnosis and at last echocardiography were 9.8 ± 3.3 and 3.5 ± 4.9, respectively; what represents a mean decrease of 5.4 ± 3.45 SD (mean follow-up of 688 ± 369 days). Although an observed tendency to CAA reduction, statistical significance was not reached. The drug infusion was well-tolerated and no adverse effects were observed.

**Conclusion:** Although current data suggest a long-term benefit, further evidence about the effectiveness of abciximab in the treatment of CAA in patients with KD is needed. Abciximab seems a safe treatment in pediatric patients with KD.

**Disclosure of Interest**

None Declared

### P143 Cogan syndrome presented in 5 years old girl with history of hypoglycaemic episodes and with significant behavioral changes

#### Marek Bohm^1^, Kamran Mahmood^1^, Valentina Leone^1^, Mark Wood^2^

##### ^1^Paediatric Rheumatology, Leeds General Infirmary, Leeds Teaching Hospitals Trust, Leeds, UK, Leeds, United Kingdom; ^2^Paediatric Rheumatology, Leeds General Infirmary, Leeds Teaching Hospitals Trust, Leeds, UK, Prague, Czech Republic

###### **Presenting author:** Kamran Mahmood

**Introduction:** Cogan syndrome (CS) is a rare autoimmune vasculitis affecting vision by non-syphilitic interstitial keratitis and hearing by sensorineural hearing loss, tinnitus, and vertigo. Typical CS affects cornea and inner ear. However, the “atypical” variants of CS can involve kidneys, CNS and other eye structures.

**Objectives:** A case report of a young girl with the background of episodes of ketotic hypoglycaemia, who then developed typical features of CS with significant behavioural changes and sleep disturbance, is presented.

**Methods:** A 5 years old, Caucasian girl presented with a four week history of being unwell, red eyes, vomiting, reduced appetite and behavioural changes including sleep disturbance and keeping head tilted to left side. She was noted to be Ataxic on examination.

She has been under follow up by Paediatric Metabolic team from 10 months of age, for recurrent episodes of ketotic hypoglycaemia presenting in mornings. Almost a year before presenting with CS, her sleep pattern changed markedly from prolonged sleep episodes to sleeplessness. Following this, she developed vertigo and hearing difficulties which were initially felt to be related to ear wax and episodes of otitis media. Her symptoms of sleep disturbance and behavioral changes such as reduced apetite, tiredness and tendency to keep head down and tilted to a side progressed slowly.

She was originally admitted and treated as incomplete Kawasaki disease.

**Results:** Following an ophthalmology review due to persistence of red eyes, CS was considered as a differential diagnosis due to bilateral inferior corneal epitheliopathy. Diagnosis of CS was confirmed by an MRI of head showing an enhancement of the inner ear structures, abnormal FLAIR signal and a subtle enhancement of the right iris. Her audiometry revealed high frequencies hearing loss which was in keeping with CS.

The infection screen including lumbar puncture was negative except raised antistreptococcal antibody titres (ASOT). However, she did have raised inflammatory markers during her admission. Her autoantibody screen including ANA and ANCA was negative.

Treatment: She received 2 doses of intravenous immunoglobulin (2 g/kg/dose) for suspected incomplete Kawasaki disease, however, it failed to resolve her symptoms leading to further investigations. Following a diagnosis of CS, she was treated with high dose intravenous methylprednisolone (30 mg/kg/dose) for 3 days, followed by a weaning course of oral prednisolone over 3 months at 2 mg/kg/day initially. She was commenced on methotrexate subcutaneously in dosage 15 mg/kg/week. A very good clinical response has been observed on this treatment.

**Conclusion:** CS in paediatric population has been reported in the literature. However association of CS with non-ketotic hypoglycaemia has not been reported yet. Some of her symptoms associated with non-ketotic hypoglycaemia, very young age and significant procedural anxiety posed diagnostic challenge. It was classical MRI finding supported by typical ophthalmic signs which helped to prove the diagnosis. CS is usually treated by corticosteroids, followed by disease modifying agents (DMARDs) (1). The similarities between Cogan’s syndrome and Kawasaki disease should be considered in differential diagnosis (2). Use of anti TNF or Rituximab to treat resistant cases has been reported (3), however, there are no guidelines available and the treatment remains experimental. We suggest collaboration with other centres to develop a data base on such a rare disease to increase awareness and develop consensus guidelines.

**Disclosure of Interest**

None Declared

### P144 Sensitivity of four criteria for Behçet’s disease in Japanese pediatric patients

#### Ken-Ichi Yamaguchi^1^, Satoshi Fujikawa^1,2^, and Working Group of Behçet’s Disease, Pediatric Rheumatology Association of Japan (PRAJ)

##### ^1^Immuno-Rheumatology Center, St. Luke’s International Hospital, Tokyo, Japan; ^2^Fujikawa Pediatric Clinic, Tokyo, Japan

###### **Presenting author:** Ken-Ichi Yamaguchi

**Introduction:** Three criteria, International Study Group (ISG), International Criteria for Behçet’s Disease (ICBD) and Japanese Ministry of Health Labour and Welfare (MHLW), had been used in practice for pediatric Behçet’s disease (BD) in Japan. Sensitivity of these criteria were not high enough for pediatric patients, as they were made for adult patients.

**Objectives:** Paediatric Behçet’s Disease study (PEDBD) criteria was established and reported that it had high sensitivity for pediatric BD patients from 27 countries in 2015. We used PEDBD criteria for Japanese pediatric BD patients, and confirmed the sensitivity to compare with other criteria.

**Methods:** We conducted a questionnaire survey of pediatric rheumatologists about clinical symptoms and laboratory results of pediatric BD patients in Japan. Next, we examined these patients whether they meet each criteria to know the sensitivity. Finally, we compare the sensitivity of four criteria in our patients.

**Results:** Fifty-two patients of BD were collected, 0-15 years of age of onset. The sensitivity of each criteria became as follows, ISG criteria 51%, ICBD criteria 70%, MHLW criteria 60% and PEDBD criteria 46%.

**Conclusion:** The sensitivity of these criteria seemed to be relatively low, especially the one of PEDBD criteria were lower than we expected. This results may mean that we should take into account the importance of geographical variation in pediatric BD.

**Disclosure of Interest**

None Declared

### P145 Increased serum antibody titer against HPV-16 antigen in patients with Behçet’s disease

#### Kyu Yeun Kim^1^, Do Young Kim^2^, Dong Soo Kim^1^

##### ^1^Pediatrics, Division of Rheumatology, College of Medicine, Yonsei University, Severance Children’s Hospital, Seoul, Korea, Republic Of; ^2^Department of Dermatology, College of Medicine, Yonsei University, Seoul, Korea, Republic Of

###### **Presenting author:** Kyu Yeun Kim

**Introduction:** Quadrivalent Human Papillomavirus (HPV) vaccine has been reported to be significantly associated with Behçet’s disease (BD). However, no reports have described HPV infection as a possible cause for the development of BD.

**Objectives:** The objective of this study was to evaluate whether anti-HPV antibody titer is increased in BD.

**Methods:** Sera from 93 Korean BD patients, who fulfilled the diagnostic criteria of the International Study Group for BD, were used in an enzyme-linked immunosorbent assay (ELISA). The clinical activity of BD was evaluated at the time of blood sampling. HPV-16 L1 virus-like particle (VLP) antigen was used in this study for the ELISA.

**Results:** Patients with BD had significantly higher antibody titers against HPV-16 (optical density [OD]: 0.210-3.675; mean 0.992) than that of healthy controls (OD: 0.248-0.762; mean 0.517; p < 0.001). Using a receiver operating characteristic (ROC) analysis, a cut-off value of 0.578 OD for the anti-HPV antibody titer was determined that differentiated BD patients from healthy controls. When we compared the clinical features of BD between the two groups, articular involvement of BD was more likely in patients with an anti-HPV-16 antibody titer <0.578 OD (p = 0.035). In addition, patients with an anti-HPV-16 antibody titer <0.578 were significantly younger than patients with a titer ≥0.578 OD.

**Conclusion:** HPV itself may be a possible extrinsic triggering infectious agent causing the development of BD.

**Trial registration identifying number:** This research was supported by the Basic Science Research Program through the National Research Foundation of Korea (NRF) funded by the Ministry of Education (2014R1A1A2059503).

**Disclosure of Interest**

None Declared

### P146 A case of Churg-Strauss syndrome in a 12-year-old girl

#### Maka Ioseliani, Ivane Chkhaidze, Maia Lekishvili, Nana Tskhakaia, Shorena Tvalabeishvili, Aleksandre Kajrishvili

##### M. Iashvili Children’s Central Hospital, Tbilisi, Georgia

###### **Presenting author:** Maia Lekishvili

**Introduction:** Churg-Strauss syndrome, also known as allergic granulomatous angiitis, is extremely rare in children and is associated with higher cardiopulmonary disease and mortality rates compared to adult patients. Here, we report a twelve year-old girl with a diagnosis of CSS, who presented with prominent pulmonary involvement accompanied by eosinophilia, two-year history of asthma, peripheral neuropathy but without anti-neutrophil cytoplasmic antibody (ANCA).

**Objectives:** A twelve year-old girl presented to our clinic with a two months history of periodic fiver, weight loss, productive cough, breathlessness, arthralgia, myalgia, muscle weakness, paresthesias of right extremities. She has a two-year history of asthma, sinusitis, urticaria, allergic rhinitis, gastrointestinal bleeding. On examination she had skin nodules on her extremities and purpuric lesions on her uncles.

**Methods:** We present a case report of a 12 year old girl with Churg-Strauss syndrome, who were treated in a Pediatric Rheumatology outpatient center.

**Results:** Blood tests revealed eosinophilia, ESP-61 mm/h, CRP-192 mg/l. Chest-x ray and CT demonstrated cardiomegaly, pulmonary infiltrates in right lung andbilateral pleural effusion. Echocardiogram showed aortic and mitral valve insufficiency, EF-31%, bronchoscopy demonstrated 15% of eosinophils on BALF. Skin biopsy demonstrated necrotizing granulomatous inflammation with eosinophils. Electromyography has shown a gross violation of n. peroneus. She was treated with prednisolone (2 mg/kg - reducing with appropriate scheme) with good clinical response. ESP, CRP and eosinophil levels were normalized, but cardiopulmonary involvement sustained. Cyclophosphamide was not used (parents decision).

**Conclusion:** Presenting case demonstrates the importance of considering the diagnosis of CSS in children with eosinophilia, uncontrolled asthma, sinusitis, skin lesions and cardiomyopathy. Sever organ involvement and delayed diagnosis may worsen prognosis and even lead to a fatal outcome. If started early, treatment with corticosteroids is often successful.

**Disclosure of Interest**

None Declared

### P147 Successful treatment with etanercept in a patient with refractory cutaneous polyarteritis nodosa

#### Maiko Takakura, Masaki Shimizu, Natsumi Inoue, Mao Mizuta, Akihiro Yachie

##### Department of Pediatrics, School of Medicine, Institute of Medical Pharmaceutical and Health Sciences, Kanazawa University, Kanazawa, Japan

###### **Presenting author:** Maiko Takakura

**Introduction:** Cutaneous polyarteritis nodosa (cPAN) is a form of necrotizing vasculitis of small and medium sized arteries without visceral involvement. cPAN has often relapsing and chronic course. Some patients with cPAN are resistant to various immunosuppressive treatments and have repeated exacerbations over prolonged periods.

**Objectives:** To report the effectiveness of tumor necrosis factor alpha (TNF-a) blockade as a therapeutic alternative for refractory cPAN.

**Methods:** We report the patient of cPAN refractory to various immunosuppressive treatments and tonsillectomy, who was successfully treated with etanercept (ETA). We also present a review of 3 similar cases of cPAN reported in the literature.

**Results: Case presentation:** An 8-year-old girl was referred to us with fever, erythematous papules and painful subcutaneous nodules located over her extremities and arthritis of bilateral ankles and knees. Laboratory findings revealed leukocytosis (13410/mm^3^), elevated levels of CRP (1.4 mg/dl) and ESR (61 mm/hr). Antistreptolysin O (ASLO) titer was found to increase (2380 IU/ml). Magnetic resonance image of the thighs revealed multiple high signal nodular lesions throughout the subcutaneous tissue and subfascial muscle tissue of the thighs. A skin biopsy of a subcutaneous nodule revealed a necrotizing vasculitis. The diagnosis of cutaneous polyarteritis nodosa (cPAN) was made. She was treated with prednisolone and maintained on cyclosporine and monthly infusions of cyclophosphamide. However, flare of cPAN with fever, myalgia and painful subcutaneous nodules occurred 5 times with steroid withdrawal. Tonsillectomy was performed but flare occurred again with steroid withdrawal. ETA (12.5 mg 2 times/week) was started and led to a marked improvement of her symptoms within 3 weeks. The patient presented complete clinical remission of cPAN and continues to receive ETA and cyclosporine.

**Review of the literature:** Three cases of cPAN successfully treated with TNF-α blockade have been reported. Two cases were treated with ETA and one patient was treated with infliximab. ETA and infliximab attenuated their symptoms and showed a corticosteroid sparing effect and no adverse effects.

**Conclusion:** The significant improvement observed in this patient. ETA could be an effective alternative treatment for cPAN. Further larger studies is desired to establish the effectiveness of ETA as therapeutic alternatives for cPAN.

**Disclosure of Interest**

None Declared

### P148 Complicated schoenlein-hoenoch with severe organ involvement: personal records

#### Clotilde Alizzi, Giovanni Corsello, Maria Cristina Maggio

##### University Department Pro.Sa.M.I. “G. D’Alessandro”, University of Palermo, Palermo, Italy

###### **Presenting author:** Maria Cristina Maggio

**Introduction:** Henoch-Schönlein purpura (HSP) is the most common childhood systemic small-vessel vasculitis with skin, joint, gastrointestinal and renal involvement. Uncommon gastrointestinal complications are: intussusception, bowel perforation and rarely appendicitis. Other rare complications are: ureteritis, orchitis, pancreatitis, severe intestinal bleeding.

**Objectives:** We evaluated complicated HSP children and followed them until remission.

**Methods:** In the last 3 years we followed in our Paediatric Unit 90 children (age: 4-14 years) with SHP, presenting with classical signs (cutaneous vasculitis, arthralgia and/or arthritis of limbs, abdominal pain, without a complicated picture of intussusception and/or perforation and/or severe bleeding.

Among these children 10/90 patients (11%) (age: 5-10 years) showed a complicated HSP with a recurrent form and an organ involvement.

**Results:** 7/10 patients showed an intestinal involvement with severe abdominal pain and persistent bleeding; 4 patients had severe and prolonged intestinal bleeding, resistant to steroids. 2/7 patients developed a persistent nephritis with proteinuria and hypertension.

One patient with intestinal bleeding developed a pancreatitis with persistent and significant amylase and lipase increase; 4 patients had orchitis.

All the patients were treated with methylprednisolone (1-2 mg/kg/day), with a mild tapering of steroids. In the 2 children with nephritis amlodipine was associated. All the patients had a slow and progressive ameliorating outcome, with a complete remission.

Clinical presentation and biochemical parameters at the disease onset were not predictive of a poor outcome.

During the acute phase of the complications, factor XIII of the blood coagulation system was detected: it was in the normal range in 6/7; it was reduced in one.

**Conclusion:** Most of the children affected by HSP have an excellent prognosis, however a few cases develop a complicated form, witch need steroid and/or a combined treatment with anti-hypertensive and immunosuppressive drugs.

In our population, 8% showed a complicated form, however all the patients did not show long-term sequelae.

**Disclosure of Interest**

None Declared

### P149 Incidence of IgA Vasculitis in children: A population-based study using a four-source capture recapture estimate

#### Maryam Piram^1,2,3^, Carla Maldini^4^, Sandra Biscardi^5^, Nathalie Desuremain^6^, Catherine Orzechowski^7^, Emilie Georget^8^, Delphine Regnard^9^, Isabelle Kone-Paut^10,11^, Alfred Mahr^4,12^

##### ^1^Inserm 1018, CESP, Kremlin Bicêtre, Paris, France; ^2^Pediatric Rheumatology, CHU de Bicêtre, Paris, France; ^3^Université Paris Sud, Le Kremlin Bicêtre, Paris, France; ^4^Internal Medecine, CHU Saint Louis, Paris, France; ^5^Pediatrics, CHIC, Créteil, Paris, France; ^6^Pediatrics, CHU Trousseau, Paris, France; ^7^Pediatrics, CH Sainte Camille, Bry sur Marne, Paris, France; ^8^Pediatrics, CHIV, Villeneuve Saint Georges, Paris, France; ^9^Pediatrics, CHU de Bicêtre, Paris, France; ^10^Pediatric Rheumatology, CHU de Bicêtre, Paris, France; ^11^Université Paris Sud, Kremlin Bicêtre, Paris, France; ^12^Université Paris 7-Diderot, ECSTRA, Paris, France

###### **Presenting author:** Maryam Piram

**Introduction:** The etiology of Immunoglobulin A vasculitis (IgAV), the most common childhood vasculitis in western countries, remains unclear. Changes over time in its epidemiology could guide in the search of a causal pathway.

**Objectives:** We aimed to describe the epidemiological characteristics of childhood IgAV in a large French population.

**Methods:** Cases were prospectively collected in Val de Marne county, a southeastern suburb of Paris, with 263 874 residents <15 years old. Children with incident IgAV living in this area from 2012 to 2014 were identified by 4 sources of case notification (emergency departments, pediatrics departments, private practice pediatricians, general practitioners). We calculated 95% confidence intervals (95% CIs) for incidence rates under the assumption of a Poisson distribution. Four-source capture-recapture analysis was performed using log linear modeling to estimate the completeness of case finding. Socio-professional factors, ethnicity, seasonal and geographic variations in IgAV incidence were investigated.

**Results:** The survey identified 147 incident cases meeting the study criteria, including 78 (53%) boys and 69 (47%) girls with a mean age of 6.5 years (2.2–14.7 years). The overall annual incidence (per 100,000 children) was 18.6 (95% CI 13.6–24.5), with 19.3 (12.6–28.3) for boys and 17.8 (12.8–26.7) for girls. The capture recapture analysis estimate the completeness of case finding at 62% (95% CI 50-81) with an annual incidence of 29.9 (95% CI 23.7-37.3). The annual distribution of diagnoses significantly deviated from a random distribution and indicated a nadir in summer months. Neither socioeconomic factors nor environmental factors like industrialization or pollution appear to play a role.

**Conclusion:** Our annual incidence rate of 18.6 to 29.9 per 100,000 children, gender distribution and seasonal variation are in line with data reported worldwide and show no significant periodic or secular variation over time. IgAV seems to be trigger by an indeterminate infection shared by children in community. The impact of genetic, as predisposing factor, need to be more explore in particular the role of innate immunity.

**Disclosure of Interest**

None Declared

### P150 Severe systemic vasculitis associated with primary immunodeficiency in two siblings

#### Mihaela Sparchez^1^, Laura Damian^2^, Zeno Sparchez^3^

##### ^1^2nd Department of Paediatrics, Iuliu Hatieganu University of Medicine and Pharmacy, Cluj Napoca, Romania; ^2^Rheumatology Department, Emergency Clinical County Hospital, Cluj Napoca, Romania; ^3^3rd Department of Internal Medicine, Iuliu Hatieganu University of Medicine and Pharmacy, Cluj Napoca, Romania

###### **Presenting author:** Mihaela Sparchez

**Introduction:** In paediatric rheumatology practice, systemic vasculitides associated with primary immunodeficiency disorders are highly challenging conditions.

**Objectives:**

The aim of this presentation is to report the clinical course and response to treatment in two sisters suffering from systemic vasculitis associated with unclassified hypogammaglobulinaemia.

Methods:

We retrospectively evaluated the medical records of both sisters.

Results:

The first case was a girl, born from non- consanguineous parents. At the age of 1.66 years old, she suddenly presented high fever, nonspecific maculopapular rash, athralgia, myalgia with elevated levels of acute phase reactants and prompt response to steroids. One year later, the above manifestations relapsed. A skin biopsy showed interstitial granulomatous dermatitis in association with vasculopathy. At 3 years old she developed recurrent cranial nerve palsies and livedo reticularis and then two stroke events at the age of 4 (one ischemic and the other hemorrhagic). Magnetic resonance angiography showed no evidence of cerebral vasculitis and the biopsy of the brain performed as part of the surgical procedure for the hemorrhagic stroke showed only extravasation of erythrocytes and fibrin deposition around small vessels, without significant inflammation. The antibody profile (including antiphospholipid antibodies) and clotting tests were negative. Hypogammaglobulinemia with global Ig deficiency was present from the onset of the disease and persistent in time. She could not be considered common variable immunodeficiency disorder because total serum IgG levels were not constantly reduced below 500 mg/dl. As part of a research study, she was also diagnosed with complete C4B deficiency. At the age of 5 years old she developed moderate thrombocytopenia which persisted for the next 3 years. At 5.5 years old she was also diagnosed with nodular regenerative hyperplasia (NRF) of the liver. For the first 2 years of evolution, only high dose of corticosteroids was able to control the disease manifestations. Methotrexate, azathioprine, cyclosporine, cyclophosphamide were ineffective. Conversely, mycophenolate mofetil (MMF) associated with high monthly doses of IGIV provided prompt and persistent improvement of clinical symptoms and acute phase reactants and also tapering of steroid dose. Till now, under MMF, no recurrences have been observed, but the NRH was slowly progressive. No portal hypertension has been developed so far.

Case 2, the older sister of the above patient, had the first disease manifestations at 6.8 years old. Since then, she developed the following disease features: fever, myalgia, painful subcutaneous nodules, livedo reticularis, low serum IgM and IgG4, elevated acute phase reactants, negative antibody profile, recurrent abdominal pain, systemic arterial hypertension, one episode of massive intestinal bleeding which had no obvious source at total colonoscopy, upper endoscopy and computed tomography angiography. The biopsy of a subcutaneous nodule revealed leukocytoclastic vasculitis. In her case, MMF induced only a partial remission. The association of tacrolimus permitted rapid control of the disease activity. Up to now, no neurological signs were present.

The recently described genetic disorder “deficiency of ADA2” is being considered in both sisters, but yet unproven.

Conclusion:

Aggressive medical management is often necessary and therefore the rate of drug related morbidity in high. The response to the various treatments is variable, even between siblings.

**Disclosure of Interest**

None Declared

### P151 Henoch-Schönlein purpura, a general view about the more often vasculitis in pediatric population and a study of forecast factors of renal disease relapse in Mexican population

#### Nydia Acevedo Silva, Ana V. Villarreal Treviño, Yuridiana Ramirez Loyola, Talia Diaz Prieto, Enrique Faugier Fuentes, Maria D. R. Maldonado Velazquez

##### Pediatric Rheumatology, Hospital Infantil de Mexico Federico Gomez, Mexico City, Mexico

###### **Presenting author:** Nydia Acevedo Silva

**Introduction:** Henoch-Schönlein purpura is the more often vasculitis in pediatric population, it is characterized for being a small vessel leucocitoclastic vasculitis; it has an incidence between 10 to 20.4 cases per 100,000 children, with a predominance of male sex 2:1. Etiology remains unknown. Given the tendency for spontaneous resolution of disease treatment in most cases will be supportive. There is no evidence of the benefits of renal disease treatment, mainly because few patients have severe disease at baseline.

**Objectives:** Identify factors of poor renal prognosis in mexican children with Henoch-Schönlein purpura diagnosis.

**Methods:** A transversal comparative study in a cohort of 79 patients diagnosed with HPS over a 5 years period in Hospital Infantil de Mexico, Federico Gomez. We realized parametric, descriptive, comparative, correlation and likehood ratio test were performed

**Results:** In this study, they were evaluated a total of 79 files of patients with this diagnosis; 47 female patients and 32 male patients, with a 1.6:1 relation women dominate; the mean age presentation was 82 months, predominating in autum with 34.7% and spring with 27%. 100% of patients debuted with characteristic purpuric rash, gastrointestinal symptoms were presented in 35%, 76% presented joint disease during the disease onset; as to kidney disease nephritis was found during the debut in 19% (n = 15) and elevated creatinine in 5.1% (n = 4), to which were subsequently performed renal biopsy, histological lesion more frequently found was mesangial proliferation glomerulonephritis in less than 50% of glomeruli (grade IIIa) that was found in 4 of 4 patients, currently a patient is on replacement therapy with hemodialysis, with an average of 0.5 mg / dL ± 0.11 with a maximum of 13 mg found / dL.

**Conclusion:** Statistical correlation tests of our study are consistent with other studies published in the literature, finding a statistically significant correlation between the presence of elevated at diagnosis and relapse with p = 0.011 creatinine. As prognostic factors for relapse reported in the literature we found that the presence of initial nephritis is a prognostic factor p ≤ 0.001 relapse and complications with a value of p = 0.025. In that order we can extrapolate the results of international studies to our population.

**Disclosure of Interest**

None Declared

**Table 43 (abstract 151).** Differences between patients with and without nephritis at the diagnosis of PHS

Characteristics		All patients	Patients with Nephritis	Patients without Nephritis	Value of P
		N = 79	n = 17	n = 62	
Elevated creatinine	Yes	5%	22.2%	1.4%	≤0.001
	No	95%	77.8%	98.6%	
12 hrs proteinuria mg/m2/hra		4.57 ± 7.65	10.47 ± 13	2.9 ± 2.3	≤0.001
Proteinuria	Yes	63%	88%	23%	≤0.001
	No	37%	12%	76%	
Relapse	Yes	19.5%	50%	11.6%	≤0.001
	No	80.5%	50%	88%	

### P152 Prevalence of kidney involvement during the first follow-up year in patients with Henoch–Schönlein Purpura in a pediatric institution in Bogota, Colombia

#### Pilar Perez, Angela C. Mosquera, Clara Malagon

##### Pediatric Rheumatology, El Bosque University, Bogota, Colombia

###### **Presenting author:** Pilar Perez

**Introduction:** The Henoch-Schönlein Purpura (HSP) is the most common vasculitis in children, mainly involving the skin vessels, joints, gastrointestinal tract and kidneys. Kidney involvement remains the main cause of morbidity and mortality in children. Nephritis occurs in 30 – 50% of patients during the first 4 to 6 weeks, but with a risk of appearance during the first 5 years. The spectrum of kidney involvement ranges from mild manifestations until the development of a nephrotic and/or nephritic syndrome or kidney failure. Some predicting risk factors have been identified for the development of nephritis: persistent or recurring purpura, severe abdominal symptoms, older age at debut and disease relapse. Renal prognosis has been associated with kidney biopsy findings. The aim of the study is to determine the clinical features and kidney involvement during the first year of follow-up.

**Objectives:** Determine the clinic features at debut and kidney involvement of patients with a final diagnosis of HSP during the first month, 3 months and until the first year of follow-up.

**Methods:** Retrospective study of patients with a final diagnosis of HSP in a Pediatric Rheumatology service. A single form of data collection was applied in an institution of Bogota, Colombia during the period comprised between 2010 and 2016

**Results:** N = 86 patients, 42 girls and 44 boys. Median age at disease onset was 5.3 years SD 2.4 years (range 1 - 14 years). At HSP diagnosis all the patients had palpable purpura,70.9% abdominal pain and 88.4% arthritis. Kidney involvement was present in 39/86 patients (45%). 22/39 (56%) patients had renal involvement at diagnosis: Isolated proteinuria was the most frequent finding 10/22 (45.4%) and 3 patients had nephrotic syndrome.12 patients (31%) had kidney involvement within the first month (50% had isolated proteinuria and 50% proteinuria/hematuria). Between the first and third month 2 (5%) patients had renal manifestations (all isolated proteinuria). Between 3 months and 12 months follow-up 3 patients (8%) had kidney compromise: one hematuria, one isolated proteinuria and one hematuria/proteinuria. A trend was observed to kidney involvement in patients with abdominal symptoms (p = 0,053). Renal biopsy was performed in 8/39 patients with HSP nephritis. Mesangial proliferation was the most common finding in 6/8 patients, sclerosis in 3/8 patients (1 patient with nephrotic syndrome and 2 patients with proteinuria/hematuria). There was no evidence of crescents in any of the biopsies. The mean follow-up was 26.8 months SD 17 months (range 1 - 72); there was no evidence of kidney damage in the last assessment in any of the 39 patients with kidney involvement.

**Conclusion:** In this group of patients, kidney involvement was more severe and common in the first weeks of the disease onset. The active search and education of families during the first 4 weeks of HSP onset is essential. In severe forms, it is important to conduct a kidney biopsy in order to document poor prognostic factors and adjust treatment.

**Disclosure of Interest**

None Declared

### P153 T helper 17 (Th17) and T regulatory (Treg) cells in children with Kawasaki disease – experience from a tertiary care centre in North India

#### Sagar Bhattad^1^, Amit Rawat^1^, Biman Saikia^2^, Ranjana Minz^2^, Jitendra Shandilya^1^, Surjit Singh^1^ and Pediatric Allergy and Immunology Unit, PGIMER, Chandigarh

##### ^1^Pediatrics, PGIMER, Chandigarh, India; ^2^Immunopathology, PGIMER, Chandigarh, India

###### **Presenting author:** Sagar Bhattad

**Introduction:** Kawasaki disease (KD) is an acute necrotizing vasculitis with predilection for coronary arteries. Acute KD has been associated with abnormalities in T-helper 17 (Th17)/ T-regulatory (Treg) cells and a high Th17/low Treg profile predict poor outcome in acute KD

**Objectives:** To assay Th17 and Treg cells in children with acute KD

**Methods:** Ten children with acute KD diagnosed at the Advanced Pediatrics Centre of our institute during Jan-Dec 2015 were enrolled after obtaining written informed consent from parents/guardians. Diagnosis of KD was based on American Heart Association (AHA) criteria. Children were treated with 2 g/kg of intravenous immunoglobulin (IVIg) which was given over 12-24 hours. Aspirin was administered at a dose of 50 mg/kg initially until the children were afebrile for atleast 48 hours and later changed to anti-platelet dose.

Seventeen healthy children and 9 febrile patients (not suffering from KD) were enrolled as controls. Th17 and Treg cells were estimated at admission (pre-IVIg) and at 4-8 weeks of follow-up in cases. Samples for febrile controls were drawn only at the first instance. Estimation of Th17 and Treg cells was carried out by flow cytometry using the Th17/Treg phenotyping kit- ***catalog no. 560762*** (BD Biosciencies) as per manufacturer’s recommendations. This kit contains PerCP-Cy5.5 labeled anti-CD4, Phycoerythrin (PE) labeled anti-IL-17 and AlexaFluor labeled anti-FoxP3 antibody

**Results:** Male: female ratio amongst cases was 4:1. Mean age at onset of KD was 4.45 years and the youngest child was 6 months old. Mean duration of fever at presentation was 10.7 days and 60% of children presented beyond 10 days of onset of fever. Conjunctival injection and oral mucosal changes were noted in 50% and 70% respectively while cervical lymphadenopathy and rash each occurred in 60% of our cohort. Extremity edema was noted in 50% while periungual/perineal peeling of skin was noted in all. 40% of the children had incomplete KD. Coronary artery abnormalities (CAA): coronary dilatation was noted in 3 cases at admission (left anterior descending artery in 2, left main artery in 1), which resolved at follow-up of 6 weeks. Two amongst these were subjected to CT- coronary angiography during follow up, which were normal.

Proportions of Th17 and Treg cells in cases and controls have been tabulated below. Th17 cells were increased in acute phase of KD as compared to febrile controls **(2.58% vs 1.57%, P = 0.27)** and healthy controls **(2.58% vs 2%, P = 0.21**). Treg cells were decreased in children with KD during acute phase as compared to febrile controls **(0.99% vs 1.96%, P = 0.62)** and healthy children **(0.99% vs 2.37%, P = 0.45)**. Following treatment with IVIg, Th17 cells decreased **(2.58% vs 1.88%, P = 0.13)** and Treg cells showed an increase **(0.99% vs 2.64%, P = 0.07)**, however, this difference was not statistically significant. No significant difference was noted in Th17 and Treg % between cases who had CAA and those who did not.

**Conclusion:** Th17 cells were elevated and Tregs were reduced during acute KD as compared to febrile and healthy controls; the difference, however, was not statistically significant. A larger study to assess the same differences is recommended to confirm the differences of statistical significance.

**Disclosure of Interest**

None Declared

**Table 44 (abstract P153).** Proportions of Th17 and Treg cells in cases, febrile and healthy controls (values are expressed in median)

	Cases (Pre-IVIg)	Cases (Post-IVIg)	Febrile controls	Controls
Th17 cells	2.58%	1.88%	1.57%	2%
Treg cells	0.99%	2.64%	1.96%	2.37%

### P154 QT dispersion in children with Kawasaki disease with transient coronary artery abnormalities: a follow-up study

#### Man S. Parihar^1^, Surjit Singh^1^, Pandiarajan Vignesh^1^, Anju Gupta^1^, ManojKumar Rohit^2^

##### ^1^Allergy immunology Unit, Advanced Pediatrics Centre, Chandigarh, India; ^2^Cardiology, Post Graduate Institute of Medical Education and Research, Chandigarh, India

###### **Presenting author:** Surjit Singh

**Introduction:** Majority of patients with Kawasaki disease (KD) are believed to have subclinical myocardial involvement that predispose to an increased risk of developing ventricular arrhythmia resulting from inhomogeneous ventricular repolarization. This inhomogeneous ventricular repolarization manifests as increased QT dispersion on electrocardiography. Though there are a few studies on QTd in patients with KD and persistent coronary abnormalities, there is paucity of literature on QTd in children with KD and transient coronary abnormalities.

**Objectives:** The present study was undertaken to study QT dispersion in children with KD and transient coronary artery abnormalities during follow-up.

**Methods:** Twenty children with KD and transient coronary artery dilatation that resolved within 1 year of diagnosis (mean age 11.5 ± 3.7 years; range between 5.1 to 19.6 years) were studied at least 1 year after resolution of coronary artery abnormalities. Diagnosis of KD was based on standard guidelines of the American Heart Association. As per unit protocol, treatment included intravenous immunoglobulin (IVIg) infusion (2 g/kg) along with aspirin (30-50 mg/kg/day during acute stage followed by 3-5 mg/kg/day for 4-6 weeks or until resolution of CAA, whichever was later). Mean interval between onset of KD and IVIg infusion was 11.05 ± 6.24 days. 20 healthy controls, matched for age and sex, were also enrolled. 12 lead ECG was done in all cases and controls. A single blinded observer used the digital caliper to measure the intervals with least count of 0.01 mm. The difference between the maximum and minimum corrected QTc intervals was calculated as the QTc dispersion. As only two of the six extremity leads are recorded and the remaining 4 leads are mathematically derived, QTc dispersion was also calculated in 8 leads. These included the 6 precordial leads, shortest extremity lead, and median of other 5 extremity leads.

**Results:** Mean follow up of patients was 53.70 ± 22.52 months after acute episode. Mean interval between normalization of transient coronary dilatations and enrolment in this study was 50.55 ± 22.67 months (range of 12 - 79 months). Mean QT dispersion (QTd) in cases calculated from 12 leads was 0.057 ± 0.018 seconds compared to 0.059 ± 0.015 seconds in controls (*p* = 0.785). Mean QT dispersion in cases calculated from 8 leads (QTd8) was 0.052 ± 0.017 seconds in cases and 0.056 ± 0.017 seconds in controls (p = 0.442). Corrected QT dispersion in 12 leads (QTcd) was 0.079 ± 0.028 seconds and 0.081 ± 0.028 seconds in cases and controls respectively (p = 0.747). Corrected QT dispersion in 8 leads (QTcd8) was also not found to be significantly different between cases (0.072 ± 0.022 seconds) and controls (0.078 ± 0.026 seconds; p = 0.441)

**Conclusion:** QT dispersion in children with KD and transient coronary artery abnormalities was not found to be significantly affected in long term follow up.

**Disclosure of Interest**

None Declared

### P155 Infliximab treatment for a patient with giant coronary aneurysm with refractory Kawasaki disease: 8-month old male. Case report and brief review of literature

#### Rocio Maldonado^1^, Enrique Faugier^1^, Ana Villarreal^1^, Nydia Acevedo^1^, Yuridiana Ramírez^1^, Talia Diaz^1^

##### ^1^Paediatric Rheumatology, Hospital Infantil de México Federico Gómez, Mexico City, Mexico

###### **Presenting author:** Enrique Faugier

This abstract is not included here as it has already been published.

### P156 Fibromuscular dysplasia in the practice of child rheumatologist

#### Yulia Kostina, Galina Lyskina, Olga Shpitonkova

##### Pediatric Department, I.M. Sechenov First Moscow State Medical University, Moscow, Russian Federation

**Introduction:** Fibromuscular dysplasia (FMD) – hyperplastic disease, is caused by a mutation in the gene of type III collagen (135580, 2q31, COL3A1), characterized by lesions of the kidney, at least - carotid and other major arteries. According to the literature the FMD of renal arteries is second (10-15%) in the structure of renovascular hypertension (RVG). In some cases, clinically FMD may be asymptomatic. However, the possibility of RVG, the instrumental detection of the deformation of large arteries is the reason for the conversion to a rheumatologist and a cardiologist with the assumption TA the patient.

**Objectives:** Common to FMD and TA are gross vascular murmur in the projection of the large arteries, the possibility of VAG, deformation and thickening of the large arteries, detected by instrumental research. In contrast to TA, while no FMD laboratory signs of inflammatory activity. Angiography can reveal a kind of defeat of large arteries throughout the “string of pearls”, “rosary” - thick, hardened areas of the arteries with hyperplasia interspersed with less altered areas, while at TA, affects primarily the mouth of the large arteries and possibly the aorta.

**Methods:** We observed 7 children with FMD - 4 girls and 3 boys ranging in age from 11 to 17 years (mean age at the time of detection of arterial hypertension 13.5 years - 13.4 ± 1.05). Compared the results of clinical, laboratory and instrumental (Doppler ultrasound, MRI, angiography) examinations in 6 patients with FMD and 38 with a documented diagnosis of TA.

**Results:** at the onset in all children with suspected FMD TA in identifying RVG, not amenable to standard anti-hypertensive therapy, the presence of vascular noise in the projection of the large arteries and the data of instrumental examination (Doppler ultrasound - thickening of artery walls and increased linear velocity of blood flow). However, all patients were not inflamatory syndrome at the beginning, typical for the acute phase of TA and laboratory signs of activity throughout the observation period. For differential diagnosis, along with named above instrumental methods of examination, and in 4 children was used positron emission tomography, allowing to estimate objectively the absence of active inflammatory process in the walls of arteries. Surgical treatment was performed 5 of the 7 patients with FMD. In 2 patients, in marked stenosis of the renal arteries, attempt balloon angioplasty did not lead to the desired effect. Only autovenous bilateral aortorenal prosthesis has allowed everyone to stop RVG.

**Conclusion:** Deformation of the aorta and its branches with vascular dysplasia leads to difficulties of differential diagnosis with nonspecific aortoarteritis. The lack of inflammatory syndrome at disease onset and laboratory signs of activity during the whole follow-up period in the presented clinical case are not allowed to confirm the diagnosis of Takayasu arteritis. Revealing as a new differential diagnostic method of research is positron emission tomography, allowing to estimate objectively the absence of active inflammatory process in the walls of arteries.

**Disclosure of Interest**

None Declared

### P157 Is neutrophil to lymphocyte ratio valid to predict organ involvement in Henoch-Schönlein Purpura?

#### Kubra Ozturk^1^, Zelal Ekinci^1^

##### ^1^Department of Pediatrics Rheumatology, Kocaeli Univertsity Faculty of Medicine, Kocaeli, Turkey

###### **Presenting author:** Zelal Ekinci

This abstract is not included here as it has already been published.

### P158 Childhood polyarteritis nodosa: diagnosis with noninvasive imaging techniques

#### Zeynep Birsin Özçakar^1^, Suat Fitoz^2^, Fatos Yalcinkaya^1^

##### ^1^Pediatric Rheumatology, Ankara University, Ankara, Turkey; ^2^Pediatric Radiology, Ankara University, Ankara, Turkey

###### **Presenting author:** Zeynep Birsin Özçakar

**Introduction:** Childhood polyarteritis nodosa (cPAN) is a systemic inflammatory disease characterized by histopathological evidence of necrotizing vasculitis or angiographic abnormalities. The classification criteria for cPAN involve the angiographic detection of visceral aneurysms and conventional angiography remains the overall gold standard. There are limited numbers of publications about the use of non-invasive imaging modalities in the diagnosis of cPAN.

**Objectives:** The aim of this study was to present the clinical and imaging findings of the patients with cPAN who were diagnosed with non-invasive imaging techniques.

**Methods:** Files of patients who had been diagnosed as cPAN in our department from 2005 to 2015 were evaluated, retrospectively. Demographic, clinical, laboratory and imaging findings of the patients were evaluated.

**Results:** Nine patients (8 M, 1 F; age at disease onset: 12,5 years (7-16)) had been diagnosed as cPAN in our clinic with non-invasive imaging techniques within the last 10 years. Abdominal pain, fever, fatigue and myalgia were the most frequent complaints. Doppler ultrasonography (US) was used in the diagnosis of 7 patients and computerized tomography (CT) angiography was done in 4 patients. Duration between admission to our center and diagnosis was median of 5 days, including 4 patients who were diagnosed within 24 hours of admission. Approximately 80% of our patients with cPAN had MEFV mutations and 90% had elevated ASO levels. All of them had the involvement of the gastrointestinal tract. Hepatic and cystic arterial involvements were detected in 7 and 6 patients, respectively.

**Conclusion:** This report included the largest cPAN series that were diagnosed with noninvasive imaging modalities. We suggest that non-invasive modalities, especially Doppler US should be considered in first line approach in the diagnosis of these patients, particularly in children.

**Disclosure of Interest**

None Declared

## Poster Session: Systemic JIA I

### P159 Factors associated with etoposide usage in children with macrophage activation syndrome complicating systemic Juvenile Idiopathic Arthritis

#### Annacarin Horne^1^, Francesca Minoia^2^, Francesca Bovis^2^, Sergio Davi^2^, Priyankar Pal^3^, Jordi Anton^4^, Kimo Stein^5^, Sandra Enciso^6^, Ozgur Kasapcopur^7^, Michael Jeng^8^, Despoina Maritsi^9^, Randy C. Cron^10^, Angelo Ravelli^11^

##### ^1^Peadiatric Rheumatology, Karolinska Institutet, Stockholm, Sweden; ^2^Peadiatric rheumatology, Instituto Giannina Gaslini, Genoa, Italy; ^3^Peadiatric Rheumatology, Institute of Child Health, Kolkata, India; ^4^Peadiatric rheumatology, Hospital Sant Joan de Deu, Barcelona, Spain; ^5^Pediatric oncology, Arkansas Children’s Hospital, Little Rock, United States; ^6^Pediatric Oncology, Hospital Infantil de Mexico City, Mexico City, Mexico; ^7^Pediatric Rheumatology, University of Istanbul, Istanbul, Turkey; ^8^Pediatric Oncology, Stanford, Palo Alto, United States; ^9^Pediatric Rheumatology, Athens Medical School, Athens, Greece; ^10^Pediatric Rheumatology, University of Alabama at Birmingham, Birmingham, United States; ^11^Pediatric Rheumatology, Instituto Giannina Gaslini, Genoa, Italy

###### **Presenting author:** Annacarin Horne

**Introduction:** Although macrophage activation syndrome (MAS) has been reported in association with almost all rheumatic diseases, it is by far most common in systemic juvenile idiopathic arthritis (sJIA). Reported mortality rates in MAS reach 10-20%. Standardized treatment guidelines for MAS are currently lacking, but management commonly includes high-dose corticosteroids combined with another immunosuppressive agent. Etoposide is a well established therapy in primary hemophagocytic lymphohistiocytosis. A recent systematic literature review on treatments of MAS in sJIA identified only a few patients who were given etoposide.

**Objectives:** We aimed to investigate etoposide usage among pediatric patients with MAS complicating sJIA and to identify predictors of etoposide administration.

**Methods:** Retrospective collected data from 362 patients included in the multinational study of the 2016 classification criteria for MAS in sJIA were examined to identify patients treated with etoposide and record potential predictors of etoposide administration. Variables significantly associated with etoposide usage in univariate analysis were entered in a multivariate regression model. Continuous variables were dichotomized according to the cut-off value obtained through ROC curve analysis.

**Results:** Forty of the 362 patients (11%) were treated with etoposide and 17 of those had information on all variables studied. Factors significantly associated with etoposide administration in multivariate analysis included multiorgan failure (OR 7.9, 95% CI 2.2-28.5), platelet count ≤ 132 × 109/liter (OR 5.8, 95% CI 1.8-18.2), triglycerides > 270.8 mg/dl (OR 3.7, 95% CI 1.3-10.4), aspartate aminotransferase > 389 units/liter (OR 3.7, 95% CI 1.3-10.3), and fibrinogen ≤ 1.53 gm/liter (OR 2.9, 95% CI 1.1-7.5). The AUC of the model was 0.86. In univariate analysis, there was no significant difference in mortality rate between patients given or not given etoposide.

**Conclusion:** Patients treated with etoposide were sicker than patients who did not receive this medication. However, mortality rate did not differ between the two treatment groups, suggesting that etoposide may be part of aggressive therapeutic interventions for severely ill children with MAS

**Disclosure of Interest**

None Declared

### P160 A novel homozygous frameshift mutation in LACC1 associated with severe familial forms of Juvenile Idiopathic Arthritis

#### Anne Thorwarth^1^, Sae Lim von Stuckrad^1^, Angela Rösen-Wolff^2^, Hella Luksch^2^, Patrick Hundsdoerfer^3^, Kirsten Minden^4^, Peter Krawitz^5^, Tilmann Kallinich^1^

##### ^1^Pediatric Pneumology and Immunology, Charité University Medicine Berlin, Berlin, Germany; ^2^Department of Pediatrics, University Clinic Carl Gustav Carus, Dresden, Germany; ^3^Pediatric Oncology and Hematology, Charité University Medicine Berlin, Berlin, Germany; ^4^Department of Rheumatology and Clinical Immunology, Charité University Medicine Berlin, Berlin, Germany; ^5^Institute of Medical Genetics and Human Genetics, Charité University Medicine Berlin, Berlin, Germany

###### **Presenting author:** Anne Thorwarth

**Introduction:** The pathophysiological origin of juvenile idiopathic arthritis (JIA) is largely unknown. However, recently a homozygous missense mutation c.850 T > C in LACC1 has been reported in several patients with therapy-refractory systemic JIA (sJIA) from consanguineous families in Saudi Arabia.

**Objectives:** We here describe two siblings from a large consanguineous Lebanese family with severe JIA harboring a novel homozygous LACC1 mutation.

**Methods:** Whole-exome sequencing (WES) was performed on DNA samples of two index patients with severe forms of sJIA and both healthy parents. Sequence variants were filtered for a recessive mode of inheritance (homozygous and compound heterozygous) to identify candidate variants. Candidate gene variants were validated via Sanger sequencing.

**Results:** Whole exome sequencing revealed a novel, homozygous one base pair deletion in LACC1 (NM_153218.2:c.827delC) in the affected children. Both parents are heterozygous carriers; two unaffected children carry wildtype alleles. The mutation results in a frameshift p.(Thr276Lysfs*2) with a premature stop at position 278. Its location in the fourth out of 7 exons is designated to cause either nonsense-mediated mRNA decay with loss of enzymatic activity or transcription of a truncated protein, respectively.

**Conclusion:** We here present a consanguineous family with 2 affected individuals suffering of severe forms of JIA associated with a novel mutation in LACC1. Together with the previously described cases of LACC1-associated familial systemic JIA and the reported association with other inflammatory and infectious diseases, the present cases add evidence that LACC1 is a recessive disease gene and its loss of function results in a familial JIA phenotype.

**Disclosure of Interest**

None Declared

### P161 The systemic onset Juvenile Idiopathic Arthritis national registry in Turkey: a preliminary report

#### Betul Sozeri^1^, Nuray Aktay ayaz^2^, Ezgi Deniz Batu^3^, Balahan Makay^4^, Sezgin Şahin^5^, Doğan Simsek^6^, Şebnem Sara Kılıc^7^, Kubra Ozturk^8^, Emine Sonmez^3^, Aysenur Pac Kisaarslan^1^, Ozge Altug Gucenmez^4^, Mustafa Cakan^2^, Z. Serap Arıcı^3^, Amra Adrovic^5^, Fatih Kelesoglu^9^, Yelda Bilginer^3^, Erkan Demirkaya^6^, Zelal Ekinci Ekinci^8^, Ruhan Dusunsel^1^, Erbil Unsal^4^, Ozgur Kasapcopur^5^, Seza Ozen^3^

##### ^1^Pediatric Rheumatology, Erciyes University Faculty of Medicine, Kayseri, Turkey; ^2^Pediatric Rheumatology, Istanbul Kanuni Sultan Suleyman Education and Research Hospital, Istanbul, Turkey; ^3^Pediatric Rheumatology, Hacettepe University Faculty of Medicine, Ankara, Turkey; ^4^Pediatric Rheumatology, Dokuz Eylul University Faculty of Medicine, Izmir, Turkey; ^5^Pediatric Rheumatology, Cerrahpasa Medical Faculty, Istanbul University, Istanbul, Turkey; ^6^Pediatric Rheumatology, Gulhane Military Medical Faculty, FMF Arthritis Vasculitis and Orphan disease Research Center, Ankara, Turkey; ^7^Pediatric Rheumatology, Uludag University Faculty of Medicine, Bursa, Turkey; ^8^Pediatric Rheumatology, Kocaeli University Faculty of Medicine, Kocaeli, Turkey; ^9^Pediatric Rheumatology, Capa Medical Faculty, Istanbul University, Istanbul, Turkey

###### **Presenting author:** Betul Sozeri

**Introduction:** Systemic-onset juvenile idiopathic arthritis (SJIA) is characterised by spiking fever, evanescent rash, hepatosplenomegaly, serositis, lymphadenopathy and arthritis.

**Objectives:** The aim of this study was to evaluate the clinical and laboratory findings and the monitoring treatment outcome of patients with systemic juvenile idiopathic arthritis (sJIA) in the Turkey.

**Methods:** Through the nationwide registry of Turkey, 167 patients with SJIA based on ILAR criteria were recruited between January 2000 and December 2015 at nine pediatric rheumatology centers. We evaluated the demographic characteristics, and laboratory and clinical data at the time of SJIA diagnosis. Evaluation of medications included previous and concomitant treatments.

**Results:** One hundred sixty-six sJIA patients (92 females (55,4%),74 males (44,6%) were included in this analysis. The mean age of disease onset and diagnosis was 6,15 ± 4,45 and 6,48 ± 4,53 years, respectively. The mean duration in months from diagnosis to study enrolment was 51.7 ± 42.4 for the cohort.

Macrophage Activation Syndrome (MAS) occurred in 22% of patients (n = 35) at presentation or during the disease course. The all patients was distributed according to subtypes; monocyclic 42,3%, polycyclic 33,7% and persistent/polyarticular 24%.

During follow-up, 95% of all patients with JIA had received corticosteroid (methyl prednisolone or prednisolone), 64% received a conventional synthetic disease-modifying antirheumatic drug (csDMARD), and 48% received a biologic DMARD (bDMARD). The median time from diagnosis to the initiation of a biologic therapy (BT) was 8,5 months (min-max 0–11).

The first BA was anakinra in 41 patients, etanercept in 24, tocilizumab in 9, canakinumab in 3, and adalimumab and infliximab in 1 patient each. Median follow-up duration on BT was 24 months (range 0,5–73) for the patients who did not experience a switch of BT; for switchers, median follow-up duration on BA was 6 months (range 0.1–62.0).

Switching of BT was common, with 42 patients (52,5%) switching to a second, 12 (15%) to a third and 3 (6.2%) to a fourth BA. Among 41 patients on anakinra as a first BT, 19 (46,3%) were switched to a new BT. Out of 24 patients on ETA as a first BT, 17 (71%) switched.

Inactive disease was achieved in 143 patients (91.1%), 87% of these patients treated with BT. 18 (12,6%) of the patients who achieved inactive disease fulfilled the criteria of clinical remission on medication at last follow-up.

No case of cancer was recorded. Four patients (2,4%) had amyloidosis and one patient was died. Four cases of MAS were documented

**Conclusion:** This study reflects preliminary report of SJIA multicenter, national registry in Turkey. The study is still ongoing, it is planned to increase the number of patients with the participation of other centers.

**Disclosure of Interest**

None Declared

### P162 Serial serum interleukin-6 levels in systemic Juvenile Idiopathic Arthritis as a predictor of treatment response

#### Butsabong Lerkvaleekul, Soamarat Vilaiyuk

##### Pediatrics, Faculty of Medicine Ramathibodi Hospital, Mahidol University, Bangkok, Thailand

###### **Presenting author:** Butsabong Lerkvaleekul

**Introduction:** Interleukin-6 (IL-6) is the main cytokine that has many roles in pathogenesis of systemic juvenile idiopathic arthritis (SJIA). Previous studies showed that serum IL-6 levels were high in active SJIA patients when compared with inactive SJIA patients or healthy controls. However, the benefit of serial serum IL-6 levels monitoring is still unknown.

**Objectives:** To determine if serial serum IL-6 levels in SJIA patients can be a predictor of the treatment response.

**Methods:** A total of 422 serum samples were obtained from 26 SJIA patients, fulfilled the International League of Associations for Rheumatology (ILAR) criteria, in Ramathibodi hospital between January 2013 and May 2015. Serum IL-6 levels were measured during active disease and repeated measures every 2-6 weeks. In each visit, disease activity were assessed by using Juvenile Arthritis Disease Activity Score-27 (JADAS-27), including number of active joints (from total 27 joints), erythrocyte sedimentation rate (ESR), physician global assessment, parent/patient global assessment. American College of Rheumatology Pediatric (ACR Pedi) 30, 50, 70, 90 criteria were used to determine the treatment response. SJIA patients were classified into two groups based on serial serum IL-6 levels: 1) rapid decrement group, serum IL-6 levels returned to normal within 1 year after active disease; 2) persistent group, highly persistent serum IL-6 levels, which were not return to normal within 1 year. Simple descriptive statistics and survival analysis were used. For predictor of reaching ACR pedi 50, multivariate analysis by Cox’s regression model was applied. This abstract is original and previously unpublished work.

**Results:** There were 12 patients in the rapid decrement group and 14 patients in the persistent group with 20.8 months of mean follow-up period. Baseline characteristics between two groups in age, sex, and disease activity at time of enrollment were not different. In rapid decrement group, median duration for returning to normal of serum IL-6 levels was 7.4 months and median duration for reaching ACR pedi 30, 50, 70, 90 were 1.6, 2, 4.7, and 7 months, respectively. In persistent group, only 3 patients had serum IL-6 levels returned to normal in 21 months. Median duration of reaching ACR pedi 30 was 18 months while no ACR pedi 50, 70, and 90 response were reached. After multivariate analysis was done, the predictor of reaching ACR pedi 50 was SJIA patients whose serum IL-6 levels returned to normal within 1 year (Hazard ratio 38.74, 95% Confidence Interval 3.43-437.87).

**Conclusion:** Monitoring serial serum IL-6 levels is beneficial in assessment the treatment response in SJIA patients. Returning to normal levels of serum IL-6 within 1 year was the predictor of reaching ACR pedi 50 and also demonstrated a better treatment response when compared with highly persistent serum IL-6 levels.

**Disclosure of Interest**

None Declared

### P163 Validation of MRP8/14 serum levels as biomarker for the diagnosis of systemic Juvenile Idiopathic Arthritis in fever of unknown origin

#### Maria Miranda-Garcia^1^, Carolin Pretzer^1^, Hans-Iko Huppertz^2^, Gerd Horneff^3^, Johannes-Peter Haas^4^, Gerd Ganser^5^, Jasmin Kuemmerle-Deschner^6^, Helmut Wittkowski^1^, Michael Frosch^7^, Johannes Roth^8^, Dirk Foell^1^, Dirk Holzinger^1^

##### ^1^Department of Pediatric Rheumatology and Immunology, University Children’s Hospital Muenster, Muenster, Germany; ^2^Prof.-Hess Children’s Hospital and Gesundheit Nord Klinikverbund Bremen, Bremen, Germany; ^3^Asklepios Clinic Sankt Augustin, Sankt Augustin, Germany; ^4^German Center for Pediatric and Adolescent Rheumatology, Garmisch-Partenkirchen, Germany; ^5^St. Josef-Stift Sendenhorst Hospital, Sendenhorst, Germany; ^6^Department of Pediatrics, Division of Pediatric Rheumatology, University Children’s Hospital Tuebingen, Tuebingen, Germany; ^7^German Pediatric Pain Centre, Children’s and Adolescents’ Hospital, Datteln, Germany; ^8^Institute of Immunology, University Hospital Muenster, Muenster, Germany

###### **Presenting author:** Dirk Holzinger

**Introduction:** The differential diagnosis of fever of unknown origin (FUO) is a major challenge in pediatrics especially for differentiation of systemic-onset juvenile idiopathic arthritis (SJIA) and infectious diseases. In a pilot study the analysis of MRP8/14 serum has been demonstrated as an excellent tool for the diagnosis of SJIA, allowing early differentiation of patients with autoinflammatory diseases SJIA or Familial Mediterranean Fever (FMF) versus those with other diseases with a specificity of 95% (Frosch et al. A&R 2009).

**Objectives:** Based on our study published in 2009, the analysis of MRP8/14 serum levels has been offered to paediatric rheumatologists in Germany in the last years. Therefore, we aimed to validate the findings from our pilot study in samples from daily clinical practice and to evaluate their relevance in clinical practice.

**Methods:** The study was designed as a retrospective analysis. The study group comprised 984 patients from 44 centers who presented with FUO. Patients were selected from our database between January 2009 and October 2012. Data collected included signs (laboratory parameters CRP, BSG, leucocytes) and symptoms as well as the final diagnosis made by the caring physicians. In all samples, concentration of MRP8/14 was determined by sandwich enzyme-linked immunosorbent assay (ELISA) with a cut-off of 9200 ng/ml (for SJIA versus other diseases). A questionnaire about the relevance of the MRP8/14 result for the final diagnosis was completed.

**Results:** Final diagnoses made by physicians were SJIA (n = 301), FMF (n = 135) and other inflammatory diseases (including infections, vasculitis and other autoinflammatory diseases) (n = 548). MRP8/14 serum levels of patients with SJIA or FMF (10.090 ± 1.930 ng/ml, mean ± SEM) were elevated compared to other diagnoses (3.140 ± 570 ng/ml) irrespectively of the presence of fever and anti-inflammatory treatment. In the group of untreated patients with fever (n = 213) MRP8/14 levels of SJIA patients (18.685 ± 4.130 ng/ml) were even higher compared to other diagnoses (5.285 ± 1.535 ng/ml). In this group, the sensitivity and specificity of MRP8/14 to differentiate between patients with SJIA vs. other diseases (excluding FMF) were 75% and 89% respectively. The sensitivity of the test increased to 83% in the presence of fever, joint pain and CRP > 1 mg/dl. There was a significant correlation (p < 0,001) between MRP8/14 and CRP or leucocyte counts in the total group of patients with SJIA. The clinicians reported that MRP8/14 results were helpful (56%), decisive (11%), not important (32%) and distracting (1%) in the total cohort.

**Conclusion:** Measurement of MRP8/14 levels is a helpful tool for the diagnosis of SJIA in FUO. In daily clinical practice the marker has a sensitivity of 75% and specificity of 89% to detect SJIA in untreated patients with fever. Additional markers might be useful to improve the detection of SJIA in patients presenting with FUO.

**Disclosure of Interest**

None Declared

### P164 Proteomic discovery of SJIA diagnostic and phenotypic biomarkers

#### F Gohar^1^, Angela McArdle^2^, Niamh Callan^2^, Belinda Hernandez^2^, Miha Lavric^1^, Christoph Kessel^1^, Dirk Holzinger^1^, Oliver FitzGerald^2^, Stephen R. Pennington^2^, Dirk Foell^1^

##### ^1^Paediatric Rheumatology and Immunology, University of Muenster, Muenster, Germany; ^2^UCD Conway Institute of Biomolecular and Biomedical Research, University College Dublin, Dublin, Ireland

###### **Presenting author:** F Gohar

**Introduction:** Systemic juvenile idiopathic arthritis (SJIA) is the most severe form of childhood arthritis. At onset, it resembles an autoinflammatory disease. Later in the clinical course, subphenotypes of SJIA are recognised, which may require different therapeutic strategies. However, SJIA specific diagnostic and phenotypic biomarkers are still lacking.

**Objectives:** We aimed to identify putative serum biomarkers that could distinguish patients with two subphenotypes of SJIA (classical SJIA with predominant systemic features ‘sys_SJIA’ or articular dominant ‘artic_SJIA’) from patients with infection.

**Methods:** All SJIA patients selected were diagnosed according to ILAR criteria, and those with infection had a confirmed bacterial or viral illness. Crude serum was first affinity-depleted of the 14 highest abundant proteins (*MARS14*, Agilent Technologies). In-solution trypsin digestion was performed on depleted serum samples. Samples were then desalted and eluted using C18 resin pipette stage tips. Label-free liquid chromatography mass spectrometry (LC-MS/MS) was performed on two platforms, the quadruple time-of-flight (QTOF) and the Q-Exactive for comparison. The false discovery rate (FDR) was set at 0.01. Both sample and technical replicates were included to minimise biological and technical variability. Software packages were used for qualitative (using Mass Hunter) and quantitative (PEAKS Studio, Spectrum Mill and Max Quant) analyses and a range of statistical analyses, which included Random Forest and principal component analysis, was undertaken.

**Results:** Patients with sys_SJIA (n = 10), artic_SJIA (n = 10) and infection (n = 10) had clinical features as follows: fever present (10 vs 0 vs 10), joint pain (7 vs 5 vs 4), exanthema (6 vs 0 vs 3), serositis (1 vs 0 vs 1) and hepatosplenomegaly (3 vs 1 vs 4). Biologic therapy was only used by n = 2 in the artic_SJIA group, while steroids were used by 3 and 2 patients in the sys_ and artic_SJIA groups respectively.

The results of univariate and multivariate analyses are shown in the Table. Between 31 and 41 differentially expressed proteins were identified in the group comparison analyses. In multivariate analyses, this translated to high area under the curve (AUC) for all SJIA (n = 20) versus infection (0.94). However, as anticipated, the sys_SJIA was not as easy to discriminate from the infection group (AUC: 0.58) as the artic_SJIA group (AUC: 0.97). However, the AUC for sys_SJIA versus artic_SJIA was 0.94, with proteins related to innate immune pathways being overrepresented in sys_SJIA.

**Conclusion:** This study indicates differences in the serum proteomic signature of patients with SJIA who have different clinical courses/phenotypes, as well as in SJIA versus infection. These findings will be verified using a quantitative multiple reaction monitoring (MRM) assay on a larger cohort of patients, which is currently under development. Whilst further evaluation is still required, biomarker signatures based on this work could potentially be used in the future for improved diagnosis, classification and management of patients with SJIA.

**Trial registration identifying number:**

**Disclosure of Interest**

None Declared

**Table 45 (abstract P164).** See text for description

	Univariate analysis	Multivariate analysis: AUC	Overlapping proteins
	Differentially expressed proteins, n, p ≤ 0.05	(Top 50 differentially expressed proteins)	(n)
Sys_SJIA vs artic_SJIA	41	0.94	5
SJIA (all) vs infection	35	0.86	3
Sys_SJIA vs infection	31	0.58	2
Artic_SJIA vs infection	36	0.97	4

### P165 Anakinra for first line steroid free treatment in systemic onset Juvenile Idiopathic Arthritis

#### Gerd Horneff^1^, Joachim Peitz^1^, Joern Kekow^2^, Ariane Klein^1^

##### ^1^ASKLEPIOS, Sankt Augustin, Germany, ^2^Helios Clinic, Vogelsang, Germany

###### **Presenting author:** Gerd Horneff

**Introduction:** First line treatment with the IL-1 inhibitor Anakinra is recommended by the ACR (1) and part of the Childhood Arthritis and Rheumatology Research Alliance consensus treatment plans for systemic JIA (2).

**Objectives:** In patients naïve for corticosteroids, as a hypothesis, a corticosteroid-free treatment regimen may allow reconstitution of an impaired NK-cell function resulting into decrease of IL-18 and probably remission of the disease (3).

**Methods:** Experience with first line treatment with Anakinra for systemic JIA is presented

**Results:** 6 pts presenting with systemic JIA were treated with Anakinra after informed consent was taken for off label treatment. Two pts had received a first 3 day course of corticosteroids before referral. All pts presented with fever and rash, 3 with arthralgia and 2 with polyarthritis. After admission, spiking fever was documented for three days while infections were ruled out thoroughly. ANA/ANCA or rheumatoid factors were undetectable in all pts in the presence of highly elevated acute phase reactants, IL-18 and myeloid-related protein S100A8/A9 (table). Treatment with Anakinra was started at a dosage of 2-3 mg/kg sc. daily. Immediate complete response was observed in 4 pts (table) while virtually no effect was reached in one. In the remaining patient, a 15 year old girl, absence of fever was noted for 20-22 hours after Anakinra injection. Furthermore a marked improvement of arthritis was noted and proven by diminished Doppler-signalling. However, the patient refused to receive two daily injections and was set on oral corticosteroids. She immediately improved but deteriorated upon tapering of steroids and finally was treated successfully with tocilizumab. In one responder, recurrence of the disease was noted after discontinuation of Anakinra and the patient was set on oral corticosteroids recently. In a further responder, a disease flare upon treatment was managed with increased dosing of Anakinra to 5 mg/kg but the patient developed life threatening macrophage activation syndrome triggered by a viral infection and treatment was discontinued. Beside this event, Anakinra was tolerated uneventfully.

**Conclusion:** Experience with first line treatment with Anakinra for systemic JIA is presented. Anakinra treatment was initially successful in 4 patients while remission off drug was reached in 2 only. The presence of arthritis, especially with a polyarticular pattern might be prognostic. Of importance, one child developed MAS upon treatment with Anakinra triggered by a viral infection. Beside this, treatment with Anakinra was well tolerated and unfavourable effects of long lasting steroid application were avoided in the responders.

References

1) Beukelman T et al Arthritis Care Res 2011;63:465–82

2) DeWitt EM et al Arthritis Care Res 2012;64:1001–10.

3) Vastert SJet al. Arthritis Rheum 2014;66:1034–43

**Disclosure of Interest**

None Declared

**Table 46 (abstract P165).** See text for description

Patient	1	2	3	4	5	6
Age (years), gender	11, m	2, m	1, f	8, f	15, f	11, m
Systemic features	Spiking fever, rash,	Spiking fever, rash, hepatomegaly, splenomegaly	Spiking fever, rash, hepatomegaly, splenomegaly	Spiking fever, rash	Spiking fever, rash	Spiking fever, rash, hepatomegaly, splenomegaly
Joint involvement	Arthralgia	Arthralgia	Oligoarthritis	Poly-arthritis	Oligo-arthritis	Arthralgia
ESR, mm/h	45	68	na	na	63	na
CRP, mg/l	95.6	101.7	106.6	197.0	329.8	99.0
IL18, pg/ml	2,483	>10,000	>5,000	>10,000	>10,000	>5,000
Pretreatment	3 days corticosteroids	none	none	none	none	3 days corticosteroids
Initial response	Immediate complete response	Immediate complete response	Immediate partial response	none	Improvement of arthritis, suppression of fever	Immediate complete response
Outcome	Remission off treatment	Remission off treatment	Increase of dosage upon flare, discontinued with MAS	No response, switch to Tocilizumab	Minor response, switch to Tocilizumab	Remission, relapse after discontinuation

### P166 Experience with Tocilizumab, interleukin-1 inhibitors and etanercept for systemic Juvenile Idiopathic Arthritis

#### Gerd Horneff^1^, Anna C. Schulz^1^, Kirsten Minden^2^, Frank Weller-Heinemann^3^, Anton Hospach^4^, J-Peter Haas^5^, and BIKER collaborative group

##### ^1^ASKLEPIOS, Sankt Augustin, Germany; ^2^Charite, Berlin, Germany; ^3^Prof. Hess Children’s Hospital, Bremen, Germany; ^4^Olgahospital, Stuttgart, Germany; ^5^German Centre Pediatric and Adolescent Rheumatology, Garmisch-Partenkirchen, Germany

###### **Presenting author:** Gerd Horneff

**Introduction:** In Germany, surveillance of approved biologics for juvenile idiopathic arthritis (JIA) is performed by the Biologics Register (BiKeR) since 2001.

**Objectives:** To report on the efficacy and safety of Tocilizumab (TOC), the interleukin-1 inhibitors Anakinra and Canakinumab (IL-1i) compared to Etanercept (ETA).

**Methods:** Patients with systemic JIA treated with ETA, TOC or IL-1i were identified. Efficacy was determined using the JADAS. A 6 month treatment period was choosen as a meaningful time. Pts who discontinued prematurely due to inefficacy or intolerance were calculated as non-responders in an intention to treat analysis.

**Results:** 270 treatments with TOC (n = 72), IL-1i (Anakina n = 36, Canakinumab n = 19) or ETA (n = 143) were identified. ETA was used in 80% before 2008, all therapies with Il6i and 76% with IL1i started after 2008. Median(IQR) age (ETA 8.2 [5.3-13]; TOC 9.6 [4.9-13.3]; IL-1i 8.2 [5.1-13.3]) and disease duration (ETA 3.3 [1.1-6.6]; TOC 3.3 [0.6-7.9]; IL-1i 3.0 [0.6-7.0]) were comparable. Pre-treatment consisted of systemic corticosteroids in all patients and MTX in 88% with ETA, 83% with TOC cohort 74% with IL-1i. Concomitant treatment with systemic steroids and MTX was significantly less frequent with TOC (44%, p < 0,001/64%; p = 0.004) and IL-1i (44%, p < 0.001/44%; p < 0,0001) compared to ETA (83%/81%). Systemic manifestations were present at baseline in 8% ETA, 96% TOC and 38% IL-1i cohort. The mean (SD) baseline JADAS10 was higher in the ETA (20.7+/-9.1), than in TOC (16.2+/-10.6) or IL-1i cohort (7.8+/-8.9). A marked decrease of the JADAS was noted in all cohorts. Intentions to treat analysis revealed a significant advantage of TOC and Il-1 inhibitors over ETA (table). Significantly more patients reached a JADAS-Remission upon TOC (44.1%; OR3.85;p < 0.001) or upon IL-1 inhibitors (50%; OR 4.6;p = 0.003) than with Etanercept (18.2%). Furthermore, significantly more patients reached a JADAS-MDA upon TOC (61.8%; OR 3.6;p = 0.003) or upon IL-1 inhibitors (65.6%; OR 4.5;p < 0.0001) than with Etanercept (29.5%). ETA was more often discontinued due to inefficacy (26.1%) than TOC (11.8%) or IL-1i (21.9%).

There were 71 AE with ETA, 118 with TOC and 81 with IL-1i. Rate of AE (per patient-year) was significantly higher in the TOC (RR 5.3; p < 0.0001) and IL-1i-group (RR 3.5; p < 0.01) and serious AE were also more frequent upon TOC (RR 2.5; p = 0.01) and IL1i (2.3;p = 0.002). Of 125 infections, 13 were reported as serious. The rate of infections was significantly higher in the TOC cohort (RR 11.0; p < 0.0001), IL-1i cohort (RR 7.4; p < 0.001). Intolerance led to discontinuation of ETA in 5 (3.5%), TOC in 6 (8.3%) and IL-1i in 1 (2%). 2 patients died, one with septic shock about 2 years after discontinuation of ETA and one died upon a MAS occurring 6 months after last administration of ETA.

**Conclusion:** A high proportion of patients showed a significant response to treatment especially with Tocilizumab or with IL-1 inhibitors. After 6 months on treatment JADAS remission criteria were reached by up to 50% of patients while 60% to 65% reached JADAs minimal disease activity. Etanercept has been used in earlier times but it is clearly less effective and it’s use in systemic JIA has markedly decreased in Germany.

**Disclosure of Interest**

None Declared

**Table 47 (abstract P166).** See text for description

		Etanercept	Tocilizumab	IL-1i
		N = 143	n = 72	N = 55
Baseline	JADAS10 Median (IQR)	8.2 (5.3–13.0)	9.6 (4.9–13.3)	8.2 (5.1–13.3)
Month 6	JADAS10 Median (IQR)	6.2(1.1–15.6)	0.8 (0.1–5.3)	6.6 (0.2–2.0)
	JADAS MDA n (%)	26 (29.5%)	21 (61.8%)	21 (65.6%)
	JADAS Remission n (%)	16 (18.2%)	15 (44.1%)	16 (50%)
Discontinuations (< Month 6)	Inefficacy (n, %)	23 (26.1%)	4 (11.8%)	7 (21.9%)
	Intolerance (n, %)		1 (2.9%)	
	Remission (n, %)	1 (1.1%)		

### P167 Inflammatory gene expression profile and defective interferon-gamma and granzyme k in natural killer cells of systemic Juvenile Idiopathic Arthritis patients

#### Karen Put^1^, Jessica Vandenhaute^1^, Anneleen Avau^1^, Annemarie van Nieuwenhuijze^2^, Ellen Brisse^1^, Tim Dierckx^3^, Omer Rutgeerts^4^, Josselyn E. Garcia-Perez^2^, Jaan Toelen^5^, Mark Waer^4^, Georges Leclercq^6^, An Goris^7^, Johan Van Weyenbergh^3^, Adrian Liston^2^, Lien De Somer^8^, Patrick Matthys^1^, Carine H. Wouters^8,9^

##### ^1^Laboratory of Immunobiology, Rega Institute, University of Leuven, Leuven, Belgium; ^2^Autoimmune Genetics Laboratory, VIB, Ghent, Belgium; ^3^Laboratory of Clinical and Epidemiological Virology, Rega Institute, University of Leuven, Leuven, Belgium; ^4^Laboratory of Experimental Transplantation, KU Leuven - University of Leuven, Leuven, Belgium; ^5^Department of Pediatrics, University Hospitals Leuven, Leuven, Belgium; ^6^Department of Clinical Chemistry, Ghent University, Ghent, Belgium; ^7^Laboratory of Neuroimmunology, KU Leuven - University of Leuven, Leuven, Belgium; ^8^Pediatric Rheumatology, University Hospitals Leuven, Leuven, Belgium; ^9^Laboratory of Pediatric Immunology, KU Leuven - University of Leuven, Leuven, Belgium

###### **Presenting author:** Jessica Vandenhaute

**Introduction:** Systemic juvenile idiopathic arthritis (sJIA) is a severe immune-inflammatory childhood disease characterized by arthritis and systemic features, such as quotidian fever, rash, lymphadenopathy and serositis.

**Objectives:** The role of natural killer (NK) cells in sJIA pathogenesis remains unclear. Therefore, we investigated the phenotype, functionality and gene expression of NK cells in sJIA patients.

**Methods:** RNA-Sequencing of highly purified NK cells from six active sJIA patients and six healthy controls was performed to investigate NK cell-specific transcriptional alterations. NK cell-stimulating and other cytokines were quantified in plasma (n = 18). NK cell phenotype and cytotoxic activity against tumor cells were determined (n = 10), next to their IFN-γ-producing function (n = 8).

**Results:** NK cells of sJIA patients showed an altered gene expression profile compared to controls, with increased expression of innate genes such as *TLR4* and *S100A9* and decreased expression of immune-regulating genes like *IL10RA* and *GZMK*, indicating an enrichment of immune-inflammatory pathways in sJIA. Alterations in the balance of inhibitory and activating receptors, with decreased KLRG1 and increased NKp44 expression, were noted in sJIA NK cells. Although sJIA NK cells showed intact cytotoxicity and degranulation against tumor cells, a decreased granzyme K expression in CD56^bright^ NK cells and a defective IFN-γ-production in response to IL-18 was observed. In sJIA plasma, an increased IL-18 and decreased IFN-γ/IL-18 ratio was determined.

**Conclusion:** NK cells in sJIA display an innate inflammatory gene expression profile. Although their cytotoxic function is intact, subtle defects in NK-related regulatory pathways such as granzyme K expression and IL-18-driven IFN-γ production may underlie the immune-inflammatory dysregulation in sJIA.

**Disclosure of Interest**

None Declared

### P168 Serum IL-18 levels as a marker for disease activity in systemic Juvenile Idiopathic Arthritis during tocilizumab therapy

#### Mao Mizuta^1^, Masaki Shimizu^1^, Natsumi Inoue^1^, Yasuo Nakagishi^2^, Akihiro Yachie^1^

##### ^1^Department Of Pediatrics, School Of Medicine, Institute of Medical Pharmaceutical and Health Sciences, Kanazawa University, Kanazawa, Japan; ^2^Department of Pediatric Rheumatology, Hyogo Prefectural Kobe Children’s Hospital, Kobe, Japan

###### **Presenting author:** Mao Mizuta

**Introduction:** Tocilizumab (TCZ), a humanized anti-interleukin (IL)-6 receptor monoclonal antibody, is an effective cytokine inhibitor for the treatment of systemic juvenile idiopathic arthritis (s-JIA). However,clinical symptoms of s-JIA could be masked during TCZ therapy. even in macrophage activation syndrome (MAS) phase. Furthermore, serum CRP concentrations never increased during TCZ therapy, even in disease flare. Hence, a novel and promising biomarker for evaluating s-JIA disease activity is desired.

**Objectives:** The aim of this study is to investigate the clinical significance of serum IL-18 levels as a marker for disease activity and for predicting the prognosis in patients with s-JIA during TCZ therapy.

**Methods:** We serially measured serum IL-18 levels in 14 patients with s-JIA during TCZ therapy and compared them with the disease activity and their prognosis. Serum concentrations of IL-18 was evaluated using commercial enzyme-linked immunosorbent assay, according to the manufacturer’s instructions.

**Results:** Serum IL-18 levels in patients with s-JIA treated with TCZ during active phase were significantly higher than those in inactive and remission phase as well as those in patients with s-JIA without TCZ. Serum IL-18 levels in 8 patients without relapse decreased to the levels <1000 pg/ml in remission phase. In contrast, serum IL-18 levels in all 6 patients with relapse sustained elevated or elevated again up to the levels >1000 pg/ml even after serum IL-18 levels normalized in inactive phase.

**Conclusion:** Serum IL-18 level is a useful marker for assessing the disease activity and predicting the prognosis in patients with s-JIA during TCZ therapy. Further larger studies is desired to define the diagnostic value of serum IL-18 as a promising biomarker for evaluating s-JIA disease activity.

**Disclosure of Interest**

None Declared

### P169 Cytokine profile of Adult-Onset Still’s Disease: comparison with systemic Juvenile Idiopathic Arthritis

#### Masaki Shimizu, Natsumi Inoue, Mao Mizuta, Akihiro Yachie

##### Department of Pediatrics, School of Medicine, Institute of Medical Pharmaceutical and Health Sciences, Kanazawa University, Kanazawa, Japan

###### **Presenting author:** Masaki Shimizu

**Introduction:** A comparison of the epidemiology, genetic background, clinical presentation, and course of these diseases suggests convincingly that systemic juvenile idiopathic arthritis (s-JIA) and adult still’s disease (AOSD) are identical or similar illnesses. However, it remains unknown whether s-JIA and AOSD share the same pathogenesis. Recent studies have shown that inflammatory cytokines play pathogenic roles in the disease processes of both diseases. We hypothesized that the patterns of cytokine release in s-JIA and AOSD share common features and s-JIA and AOSD belong to the same disease category.

**Objectives:** To test our hypothesis, we analyzed serum levels of cytokines in patients with both diseases and compared them with the clinical features.

**Methods:** 33 patients with AOSD, 77 patients with s-JIA were analyzed. Serum levels of IL-18, IL-6, neopterin, sTNFRI and II were quantified by ELISA. Results were compared with clinical features of AOSD.

**Results:** Cytokine profile of both diseases was almost similar. It was characteristic that serum IL-18 levels were extremely high in active phase and remained elevated even in inactive phase. Two distinct subsets based on their serum IL-6 and IL-18 levels were identified in AOSD as well as s-JIA. The number of patients with arthritis was significantly higher in the IL-6–dominant subgroup.

**Conclusion:** AOSD and s-JIA are the same spectrum, which is based on the significant production of IL-18. Two subsets of patients with AOSD can be identified on the basis of their serum IL-6 and IL-18 levels. These two subsets appear to be characterized by certain distinct clinical features. Monitoring the cytokine profile with IL-18 and IL-6 might be useful to predict disease course.

**Disclosure of Interest**

None Declared

### P170 Genetic architecture of systemic Juvenile Idiopathic Arthritis distinguishes it from oligo- and polyarticular Juvenile Idiopathic Arthritis

#### Michael J. Ombrello^1^, Victoria Arthur^1^, Elaine F. Remmers^2^, Anne Hinks^3^, Daniel L. Kastner^2^, Patricia Woo^4^, Wendy Thomson^3,5^, and International Childhood Arthritis Genetics (INCHARGE) Consortium

##### ^1^Tranlational Genetics and Genomics Unit, National Institute of Arthritis and Musculoskeletal and Skin Diseases, Bethesda, United States; ^2^Inflammatory Disease Section, National Human Genome Research Institute, Bethesda, United States; ^3^Arthritis Research UK Centre for Genetics and Genomics, Manchester Academic Health Science Centre, Manchester, UK; ^4^Center of Paediatric and Adolescent Rheumatology, University College London, London, UK; ^5^NIHR Manchester Musculoskeletal Biomedical Research Unit, Central Manchester NHS Foundation Trust, Manchester Academic Health Science Centre, Manchester, United Kingdom

###### **Presenting author:** Michael J. Ombrello

**Introduction:** Juvenile idiopathic arthritis (JIA) is a heterogeneous group of conditions that are unified by the presence of chronic childhood arthritis without an identifiable cause. Systemic JIA (sJIA) is a rare form of JIA that is characterized by episodic systemic inflammation and chronic arthritis. Approximately half of children with sJIA go on to develop destructive, life-long arthritis that has phenotypic similarities with the oligoarticular/seronegative polyarticular (polygoJIA) or seropositive polyarticular (RFpolyJIA) forms of JIA. Importantly, therapies that are effective in other forms of JIA are much less effective in sJIA, particularly among patients with persistent arthritis. Investigating the genetic architecture of sJIA and how it compares with other forms of JIA may improve our understanding of these differences. However because of its rare nature, genomic investigations of sJIA have been limited, providing few insights into its pathophysiology and not allowing for its comparison with other forms of JIA.

**Objectives:** In the present study, we sought to identify genetic factors that influence sJIA pathogenesis and to compare the genetic architecture of sJIA with the other common forms of JIA.

**Methods:** We performed a genome-wide association study of 982 children with sJIA collected in 9 countries by participating sites of the International Childhood Arthritis Genetics (INCHARGE) Consortium. Following stringent quality control operations, over 6 million single nucleotide polymorphisms (SNPs) were tested for association with sJIA by logistic regression, adjusted for gender and ancestry, in 9 geographically-defined strata. Association results were combined by fixed-effect meta-analysis. Weighted genetic risk scores (wGRS) based on established genetic risk factors for polygoJIA or RFpolyJIA were calculated and wGRS were compared between sJIA cases and healthy controls using the Wilcoxon Rank Sum test. The correlation of wGRS with sJIA was tested by both logistic regression and by analysis of receiver operating characteristic (ROC) curves with calculation of the area under the curve. Quantile-quantile (QQ) plots were used to determine whether sJIA-associated SNPs were enriched within the set of polygoJIA-associated SNPs from the recent study by Hinks and colleagues.

**Results:** The major histocompatibility complex locus and a second locus on chromosome 1 both showed association with sJIA that exceeded the threshold for genome-wide significance (p < 5E-8), and 23 additional loci had evidence suggestive of association with sJIA (p < 5E-6). There was virtually no overlap between the sJIA susceptibility loci and the known risk loci for other forms of JIA. ROC curve analysis and logistic regression of wGRS failed to identify any correlation between sJIA and either polygoJIA or RFpolyJIA. Examination of QQ plots found no evidence for enrichment of sJIA associations among polygoJIA-associated SNPs. Collectively, we found no evidence of shared genetic architecture between sJIA and either polygoJIA or RFpolyJIA.

**Conclusion:** sJIA has a distinct genetic architecture without evidence of pleiotropy or shared genetic risk factors with other common JIA subtypes. This indicates that sJIA is a unique disease process and not a variant form of another JIA subtype. As such, the pathophysiology of sJIA must be investigated separately from the other JIA subtypes to identify therapeutic targets specific to sJIA. Moreover, the management of children with sJIA should be directed by studies of sJIA, and not by trials or observations of other subtypes of JIA.

**Disclosure of Interest**

None Declared

### P171 Bone marrow findings at children with Juvenile Idiopathic Arthritis

#### Barbara Stanimirovic^1^, Biljana Djurdjevic-Banjac^2^, Olivera Ljuboja^3^

##### ^1^Pediatric Rheumatology, University Clinical Centar Republic of Srpska, Banjaluka, Bosnia and Herzegovina; ^2^Pediatric Hematology, University Clinical Centar Republic of Srpska, Banjaluka, Bosnia and Herzegovina; ^3^Pediatric Pulmology, University Clinical Centar Republic of Srpska, Banjaluka, Bosnia and Herzegovina

###### **Presenting author:** Barbara Stanimirovic

**Introduction:** Juvenile idiopathic arthritis (JIA) involves inflammation of one or more joints lasting more than 6 weeks and the onset of the disease before the age of sixteen with the prior exclusion of other diseases and conditions in which the arthritis occurs as part of the clinical picture. Malignant diseases in children can start with musculoskeletal manifestations and prior to the diagnosis of JIA is necessary to exclude malignancy, primarily leukemia.

**Objectives:** The aim of this study is to investigate bone-marrow findings at children with JIA at the onset of disease.

**Methods:** The study included 24 children diagnosed with JIA, treated from January 2010 to January 2016 at University Clinical center of Republic of Srpska. Bone marrow aspiration (BM) was performed in all JIA patients as a part of an initial diagnostic evaluation. Control group included 24 children with immune thrombocytopenic purpura (ITP). BM smears were retrospectively reviewed by the same hematologist for the purpose of this study. Bone marrow smears were evaluated for cell populations as well as myelodysplastic features and compared to control group.

**Results:** The study included 24 patients with JIA, 16 girls and 8 boys. Median age was 5 years and two months. Ten children had systemic onset JIA, nine had oligoarthritis and five had polyarthritis. The characteristics of BM in JIA was myeloid hyperplasia and elevated plasmocyte count. There was no difference between JIA patients and control group in terms of myelodysplastic features. The patients with JIA had a significantly higher percentage of cells of neutrophil series due to increased metamyelocyte count compared to control group. Hemophagocytosis was found at one patient with systemic onset of JIA.

**Conclusion:** We emphasize the importance of including BM aspiration into the diagnostic procedures in JIA aimed of excluding malignancies. Findings of this study can be helpful in explaining hematological changes in JIA.

**Trial registration identifying number:** DEA2487D-F0FB-4B6A-8F2A-60844E3AC45A

**Disclosure of Interest**

None Declared

### P172 Inflammatory bowel disease following anti-interleukin-1-treament in systemic Juvenile Idiopathic Arthritis

#### Boris Hugle, Fabian Speth, Johannes-Peter Haas

##### German Center for Pediatric and Adolescent Rheumatology, Garmisch-Partenkirchen, Germany

###### **Presenting author:** Boris Hugle

**Introduction:** Inflammatory bowel disease (IBD) can develop with some rheumatic diseases in childhood, including juvenile idiopathic arthritis (JIA). Inflammatory bowel disease is frequently associated with other immune-mediated diseases; however, systemic onset JIA (sJIA) has not previously been connected to IBD. Treatment of sJIA has significantly changed in recent years, possibly causing changes in inflammatory patterns.

**Objectives:** We present three pediatric cases with confirmed diagnosis of sJIA who developed IBD diagnosed by histology and coloscopy during treatment with IL-1 antagonists.

**Methods:** Data from the German Center for Pediatric and Adolescent Rheumtology from 2010 until 2015 were analyzed by retrospective chart review.

**Results:** There were 82 patients with confirmed diagosis of systemic JIA. Of these, four were identified with a diagnosis of IBD. Three of these developed IBD confirmed by colonoscopy (two cases of Crohn’s disease, one case of ulcerative colitis) 0.8 – 4.3 years after diagnosis. All three were treated with IL-1 antagonists (anakinra in two cases, canakinumab in one case). The fourth patient developed transient terminal ileitis confirmed by histology on treatment with etanercept, two months after combination treatment of anakinra and etanercept for 16 months.

**Conclusion:** IBD in systemic onset JIA is a rare complication. Treatment with IL-1 antagonists might be causally related to a switch in clinical phenotype of the underlying inflammatory process.

**Disclosure of Interest**

None Declared

### P173 Complete clinical remission with tocilizumab in two toddlers with systemic Juvenile Idiopathic Arthritis-a case series

#### Despoina Maritsi^1^, MArgarita Onoufriou^2^, Olga Vougiouka^3^, Despina Eleftheriou^4^

##### ^1^Pediatrics, P & A Kyriakou Children;s Hospital, Athens, Greece; ^2^Pediatrics, Makarios III Children;s Hospital, Nicosia, Cyprus; ^3^Pediatrics, P & A Kyriakou Children’s Hospital, Athens, Greece; ^4^Paediatric and Adolescent Rheumatology, Great Ormond Street Hospital, London, United Kingdom

###### **Presenting author:** Despoina Maritsi

**Introduction:** Systemic juvenile idiopathic arthritis (SJIA) is a multisystem inflammatory disorder rarely diagnosed in infants. Tocilizumab is considered the mainstay treatment for difficult to treat SJIA in patients over four years of age. We present two infants with SJIA who failed to respond to conventional treatment and were successfully treated with tocilizumab. To our knowledge there are no previous data on the efficacy and safety of this drug in infancy.

**Objectives:** To describe the efficacy and safety of tocilizumab in two infants wth sJIA.

**Methods:** An 8 m old male presented with persistent fever and raised inflammatory markers; no source of infection was identified. He had splenomegaly and lymphadenopathy. The patient underwent major work-up for infection and malignancy and received antibiotics. Ultrasonography revealed free peritoneal and pericardiac fluid. Symptoms persisted despite the use of antibiotics. He subsequently developed a maculopapular rash on his trunk while the following day he developed arthritis of the wrists, knees, ankles and 3^rd^ and 4^rth^ PIP joints. The patient was diagnosed with SJIA and received pulses of methylprednisolone; MTX was initiated. Genetic testing for auto-inflammatory diseases (FMF, TRAPS, HIDS, CAPS) was negative; genetic and functional screening assays for primary HLH were normal. Serositis, arthritis and lymphadenopathy improved after initiation of treatment. At twelve weeks, fever spikes and rash persisted despite continuous significant doses of prednisolone (Table 48). In addition, he became cushingoid, hypertensive and extremely irritable. Inflammatory markers were high with persistent thrombocytosis. We initiated tocilizumab. A month afterwards, the patient’s condition had markedly improved; although he occasionally presented with a rash. A year later he remains asymptomatic, leading a fully normal life.

**Results:** The other patient (female 9 m) presented with pyrexia of unknown origin for 15 days with hepatosplenomegaly and serositis. She developed bilateral knee arthritis and a maculopapular rash on day 21. Following exclusion of bacterial infection, malignancy and other genetically defined autoinflammatory disease, she was diagnosed as sJIA and started on an identical regimen as patient above. Similarly we failed to decrease the steroid dose below 1 mg/kg/day; she remained on this regimen for further six months during which she had severe growth delay; tocilizumab was initiated at 18 months of age (Table 48). After six months, she remains asymptomatic and growing well. Prednisolone was tapered over three months in both cases. Clinical remission was achieved by nine and six months for the two patients respectively. None of them developed a flare. However, both patients experienced side affects potentially related to tocilizumab. The first patient had primary varicella infection and the second had recurrent upper respiratory tract infections.

**Conclusion:** Tocilizumab may be considered as a treatment option in patients less than 2 years of age with severe sJIA. A current open label study is exploring the pharmocokinetics and pharmacodynamics of tocilizumab in this age group and will provide some efficacy and safety data.

**Disclosure of Interest**

None Declared

**Table 48 (abstract P173).** Clinical and disease characteristics of the two patients at tocilizumab initiation

Patient characteristics	Patient I	Patient II
Age	11 months	18 months
Ethnicity	Caucasian	Caucasian
Gender	male	female
Disease duration	3 months	7 months
Steroid dose	2 mg/kg/day	1 mg/kg/day
Methotrexate dose (SC)	15 mg/m^2^	15 mg/m^2^
Tocilizumab dose (IV)	12 mg/kg every two weeks	12 mg/kg every two weeks
ACR Pedi 30 (+ no fever)	By week 12	By week 8
ACR Pedi 90 (+no fever)	By week 36	By week 24
Adverse events	Varicella	Recurrent upper respiratory tract infections

### P174 Canakinumab for first line steroid free treatment in a child with systemic onset Juvenile Idiopathic Arthritis

#### Gerd Horneff^1^, Joachim Peitz^1^, Joern Kekow^2^, Dirk Foell^3^

##### ^1^ASKLEPIOS, Sankt Augustin, Germany, ^2^Helios, Vogelsang, Germany, ^3^University, Muenster, Germany

###### **Presenting author:** Gerd Horneff

**Introduction:** First line treatment with the IL-1 inhibitor Anakinra has been recommended by the ACR (1) and is part of the Childhood Arthritis and Rheumatology Research Alliance consensus treatment plans for systemic JIA(2). In patients naïve for corticosteroids, as a hypothesis, a corticosteroid-free treatment may allow reconstitution of an impaired NK-cell function resulting into decrease of interleukin-18 and probably remission of the disease (3). Canakinumab is a long acting high-affinity human IL1ß monoclonal antibody that selectively bind to human IL1ß and thereby inhibiting downstream signalling pathways.

**Objectives:** In 2014, canakinumab has been added to the CARRA treatment plans (4). Thus, a single injection of canakinumab has been used as a sole treatment in a child newly diagnosed with systemic active JIA.

**Methods:** A 2 7/12 year old boy presented with fever up to 41.0 °C, rash, arthralgia, splenomegaly, refusal to stand or walk and highly elevated acute phase reactants. Infections were ruled out thoroughly. Initial bone marrow aspirate was unremarkable. ANA/ANCA or rheumatoid factors were undetectable. The patient was referred to the rheumatology clinic.

**Results:** After admission, spiking fever was documented for 3 days. Highly elevated acute phase reactants were present. There were signs of macrophage activation syndrome including leucocytopenia, neutropenia, thrombocytopenia, elevated ferritin (>1200 μg/l), lactatedehydrogenase (1028 U/l), transaminases (ALAT 110 U7l, ASAT 124 U/l) and trigycerides (497 mg/dl) but normal fibrinogen. Biomarkers, IL-18 and myeloid-related protein S10A8/A9 were highly elevated (table).

Informed consent was taken for off label treatment with canakinumab instead of routine care with corticosteroids. Fever immediately disappeared the day after a single application of canakinumab (2 mg/kg sq.). No further rash or arthralgia were noted. CRP level rapidly decreased and step by step returned to normal while the clinical condition normalized and splenomegaly completely resolved. The patient was discharged at day 3 . A rapid decline of all disease activity markers was noted. Of interest, MRP S100A8/A9 normalized after 6 weeks while serum level of IL-18 markedly decreased but were still elevated until month 3. The patient remained free from clinical symptoms for now 3 months. Thus, the patient remained in clinical remission after a single injection of canakinumab without any anti-rheumatic treatment.

**Conclusion:** First experience with first line treatment with canakinumab for systemic JIA is presented. A single sq. injection of canakinumab, a monoclonal anti-IL-1ß antibody with prolonged plasma half life, completely suppressed disease activity in a toddler with systemic JIA and normalized inflammatory parameters and biomarkers . This treatment clearly showed advantage over daily sq. injections. Early steroid free treatment with canakinumab may lead to long lasting remission via decreased inflammasome activation and reconstitution of NK-cell function, and eventually cure of the disease. Aside, unwarranted effects of long lasting steroid application was avoided.

References

1) Beukelman T et al Arthritis Care Res 2011;63:465–82.

2) DeWitt EM et al Arthritis Care Res 2012;64:1001–10.

3) Vastert SJ et al. Arthritis Rheum 2014;66:1034–43

**Disclosure of Interest**

None Declared

**Table 49 (abstract P174).** See text for description

	HB (g/dl) [11.5–13.5]	CRP (mg/l) [<5]	Leukocytes (/μl) [5,000–14,500]	Thrombo-cytes (/nl) [150–450]	ASAT (U/l) [<38]	LDH /U/l) [155–345]	Ferritin (μg/l) [7–142]	IL-18 (pg/ml) [50–300]	S100A8/A9 (ng/ml) [<2,940)
Presentation (day -20)	9.6	60	18,800	n	n	n	4,409		
Baseline	8.8	166.12	7,360	54	119	1028	>1,200	>10,000	134,000
Day 1	8.6	41.52	6,470	85	124	852			
Day 3	9.1	21.35	6,820	82	69	520			24,000
Day 22	11.3	0.11	8,720	419	38	278			1,773
Day 50	10.9	0.27	7,080	316	34	286	63	2,805	1,150
Month 3	12.1	0.06	7,650	254	34	255			

N = reported as normal

### P175 Systemic onset Juvenile Idiopathic Arthritis: epidemiological features of Moroccan cohort

#### Kenza Bouayed^1^, Sanae El Hani^1^, Imane Hafid^1^, Nabiha Mikou^1^

##### ^1^Pediatrics, Children hospital A. Harouchi, CHU Ibn Rochd, Casablanca, Morocco

###### **Presenting author:** Kenza Bouayed

**Introduction:** Systemic onset juvenile idiopathic arthritis SoJIA is a rare auto-inflammatory disease that belongs to the big group of Juvenile Idiopathic Arthritis JIA representing about 10 to 17% of cases. It is distinguished from the other types by the presence of systemic manifestations with marked inflammatory reaction beside the articular symptoms.

**Objectives:** To analyze the epidemiological, clinical, diagnostic tests, therapeutic and evolutive parameters in SoJIA.

**Methods:** This is a retrospective study of SoJIA patients followed in a pediatric rheumatology monocenter over a 16 -year period from January 2000 to December 2015. The diagnosis was based on ILAR 2004 revised criteria. The hospital charts of these patients were reviewed and information was collected.

**Results:** The study included a total of 59 cases, 27 girls and 32 boys. The mean age of disease onset was 4 years and 5 months while the mean duration delay of diagnosis was 4 months and 9 days. Consanguinity was reported in 22% of cases. Fever was objectified in all patients; it was typical in 61% of them, while arthritis was present at onset in only 76% of patients with a 6.2 mean number affected joints during the evolution whose 13% with standard radiological expression. Morning stiffness was reported in 74% of patients and rash in only in 49%. The hematopoietic involvement was represented by hepatomegaly, splenomegaly and lymphadenopathy in 8.4%, 8% and 27% of cases and serositis in 22%. NSAIDs were prescribed in all cases, oral corticosteroids in 72% and intra-articular form in 11.8%. Methotrexate was given in 49% of patients, thalidomide in 2 cases, Leflunomide in only one case. Meanwhile biological were recommended in 15 cases 2 Etanercept and 13 (22%) Tociluzumab. The study of evolution included 42 patients. The number of relapses was ≥ 3 relapses in 22 of them, 84% with systemic manifestation and 16% with exclusive joint shape. Complications were growth impact with short stature in 22% of cases, Cushingoid facies in 40%, glucocorticoid-induced cataract in 5%, osteoporosis in 5%, macrophage activation syndrome in 2 patients; twice for one of them, severe infection in 6.7% and joint destruction in 8.4% of patients.

**Conclusion:** Our study is characterized by: a slight male predominance for a sex ratio usually 1, the occurrence of macrophage activation twice in the same patient justifying a genetic test which income normal, 2 families with 2 children affected deserving genetic exploration that not have been yet realized. The clinical course was modified by biological therapy in refractory cases. Our patients underwent conventional treatment until 2006 when etanercept was tried with poor results and replaced by Tocilizumab from 2009 permitting a complete remission for almost patients treated without treatment withdrawals because of expensive cost and outside the joint squeals already existing.

**Disclosure of Interest**

None Declared

### P176 Four years of tocilizumab therapy for systemic Juvenile Idiopathic Arthritis in Georgia

#### Maka Ioseliani, Maia Lekishvili, Nunu Shelia, Shorena Tvalabeishvili, Aleksandre Kajrishvili

##### M. Iashvili Children’s Central Hospital, Tbilisi, Georgia

###### **Presenting author:** Maia Lekishvili

**Introduction:** The blockade of interleukin 6 has been shown to reduce the corticosteroid dependence in children suffering from systemic juvenile idiopathic arthritis (s-JIA).

**Objectives:** To investigate the effect of TCZ treatment in 9 patients with systemic JIA.

**Methods:** 9 patients suffering from s-JIA with inadequate response to therapy (prednisone) have received Tocilizumab (TCZ) since the year 2013 in Georgia. They were retrospectively analyzed. The data were collected in one pediatric outpatient unit.

**Results:** The mean age at diagnosis was 32.5 months (from 13 to 73 months), there were 4 boys (44.4%) and 5 girls (55.6%) (ratio4:5). All patients were treated with prednisone for 25.2 months (SD 23.8, from 6 to 72 months) before starting TCZ. Currently 6 patients are still treated with prednisone: 4 patients (66.7%) are on therapy with prednisone with appropriate reducing scheme and in 2 patients (33.3%) prednisone was restored because flare of the disease.

The mean period of time from diagnosis to TCZ therapy was 25.6 months (SD 23.5, from 7 to 72 months). Patients were treated with TCZ for 14.6 months (SD 7.5, from 2 to 23 months). There were still 9 patients (100%) treated with TCZ.

5 patients (55.6%) are in remission, 4 (44.4%) have active arthritis, without systemic signs and symptoms.

Adverse events included mild infection (2 patients) and increased aminotransferase levels (1 patient).

**Conclusion:** Our data show that TCZ was effective in severe, persistent systemic Juvenile Idiopathic Arthritis. At 12 weeks 5 (55.6%) patients have demonstrated response in ACR Pedi 70, 3 (33.3%) - in ACR Pedi 50, 1 (11.1%) – in ACR Pedi 30.

**Disclosure of Interest**

None Declared

### P177 Systemic onset juvenile idiopathic arthritis in Estonia

#### Mari Laan^1^, Jaanika Ilisson^2^, Chris Pruunsild^2,3^

##### ^1^Tallinn Childrens’Hospital, Tallinn, Estonia; ^2^Children’s Clinic, Tartu University Hospital, Tartu, Estonia; ^3^Children’s Clinic, Institute of Clinical Medicine, Tartu University, Tartu, Estonia

###### **Presenting author:** Mari Laan

**Introduction:** Juvenile idiopathic arthritis (JIA) is a heterogeneous group of childhood-onset chronic arthritis. Systemic JIA (sJIA) as one of the disease categories has prominent systemic features as well as arthritis and accounts for 5-15% of children with JIA (1). Every year 250-270 new cases of JIA are diagnosed in Estonia

**Objectives:** The aim of this study is to describe clinical aspects, methods of treatment and achievement of clinical remission in patients with sJIA in Estonia

**Methods:** Data for the study have been collected retrospectively from medical records of newly diagnosed patients with sJIA in Tallinn Children’s Hospital and Children’s Clinic Tartu University Hospital from 2000-2015.

**Results:** During years 2000-2015 sJIA was diagnosed in 26 patients, nine boys and 17 girls. Most frequent first diagnosis at hospitalisation was fever of unknown origin or unknown bacterial infection (73% of the cases). Mean age at diagnosis was 8.8 ± 4.8 years; boys were younger (7.5 ± 5.5 years) than girls (9.6 ± 4.6 years) but the age difference wasn’t statistically significant (p = 0.36). Mean duration of fever before the diagnosis of sJIA was 14.2 ± 4.0 days, 96% of patients had rash and 50% had hepato- or splenomegaly. At hospitalisation 46% of patients did not have clinical arthritis. All patients had high values of inflammatory markers (CRP 130 ± 87 mg/l and SR 58 ± 25 mm/h). Ferritin levels were determined in 57.6% of patients and the mean value was 1479.7 ± 1284.2 mg/l. All patients were treated with corticosteroids and 17 of them received intravenous pulse therapy (mean dose 12 mg/kg/dose). Mean number of pulses needed was 3.4. Oral prednisolone was given in a mean dose of 1.1 mg/kg/day. Biologic agent was started in 10 patients at a mean duration of the disease 1.5 (95% confidence interval 0.4 – 2.7) years. During the disease course three patients had documented growth suppression, three documented osteoporosis and one child developed macrophage activation syndrome. Clinical remission on treatment was achieved in 15 patients, but clinical remission off treatment only in nine patients.

Nine patients had monocyclic and 17 polycyclic course of sJIA. During the disease course active component of the disease was arthritis in three, systemic in nine and arthritis with systemic in 14 patients.

**Conclusion:** sJIA is a rare subtype of JIA. Most frequent clinical presentation at diagnosis was fever with rash and hepato- or splenomegaly, at hospitalisation arthritis was present in only half of patients. Corticosteroids were used in all patients, 1/3 received biological treatment. The disease had polycyclic course in 2/3 of patients and remission off treatment was achieved in 1/3 of cases.

1. Ravelli A, Martini A. Juvenile idiopathic arthritis. Lancet. 2007;369(9563):767–78.

**Disclosure of Interest**

None Declared

